# Transcriptome signatures associated with meningioma progression

**DOI:** 10.1186/s40478-019-0690-x

**Published:** 2019-04-30

**Authors:** Angela N. Viaene, Bo Zhang, Maria Martinez-Lage, Chaomei Xiang, Umberto Tosi, Jayesh P. Thawani, Busra Gungor, Yuankun Zhu, Laura Roccograndi, Logan Zhang, Robert L. Bailey, Phillip B. Storm, Donald M. O’Rourke, Adam C. Resnick, M. Sean Grady, Nadia Dahmane

**Affiliations:** 1 Department of Pathology and Laboratory Medicine, University of Pennsylvania Perelman School of Medicine, Philadelphia, PA USA; 2 Center for Data Driven Discovery in Biomedicine (D³b), Children’s Hospital of Philadelphia, Philadelphia, PA USA; 3 Department of Neurosurgery, University of Pennsylvania Perelman School of Medicine, Philadelphia, PA USA; 4 Department of Neurological Surgery, Weill Cornell Medicine, New York, NY USA; 5 Present Address: Children’s Hospital of Philadelphia, Philadelphia, PA USA; 6 Present Address: Massachusetts General Hospital Department of Pathology, Harvard Medical School, Boston, MA USA; 7 Present Address: Department of neurological surgery, Weill Cornell Medicine, New York, NY USA; 8 Present Address: Department of Neurosurgery, University of Pittsburgh Medical Center, Pittsburgh, PA USA

**Keywords:** Meningioma, Progression, Transcriptome, Immune infiltration, snoRNAs

## Abstract

Meningiomas are the most common primary brain tumor of adults. The majority are benign (WHO grade I), with a mostly indolent course; 20% of them (WHO grade II and III) are, however, considered aggressive and require a more complex management. WHO grade II and III tumors are heterogeneous and, in some cases, can develop from a prior lower grade meningioma, although most arise de novo. Mechanisms leading to progression or implicated in de novo grade II and III tumorigenesis are poorly understood. RNA-seq was used to profile the transcriptome of grade I, II, and III meningiomas and to identify genes that may be involved in progression. Bioinformatic analyses showed that grade I meningiomas that progress to a higher grade are molecularly different from those that do not. As such, we identify *GREM2*, a regulator of the BMP pathway, and the snoRNAs *SNORA46* and *SNORA48*, as being significantly reduced in meningioma progression. Additionally, our study has identified several novel fusion transcripts that are differentially present in meningiomas, with grade I tumors that did not progress presenting more fusion transcripts than all other tumors. Interestingly, our study also points to a difference in the tumor immune microenvironment that correlates with histopathological grade.

**Electronic supplementary material:**

The online version of this article (10.1186/s40478-019-0690-x) contains supplementary material, which is available to authorized users.

## Introduction

Meningiomas, neoplasms of mesodermal-arachnoid origin, are the most common primary intracranial and spinal tumor [18]. About 80% show benign behavior and are amenable to surgical resection alone. However, 20% can clinically recur and require multimodal treatment (repeat surgery and radiotherapy). Currently, histopathological grade is the main predictor of meningioma behavior, with most WHO grade I (called ‘benign’) tumors having a nonmalignant course; on the other hand, grade II (‘atypical’) and III (‘anaplastic’) meningiomas are often more aggressive and recur [18]. The current WHO classification relies on histomorphological features to sub-classify meningiomas into 15 subtypes, nine for grade I and three each for grades II and III [33, 44]. Currently, the major predictors for meningioma recurrence are WHO grade (with higher grades carrying a higher risk) and extent of surgical resection [7]. To complement and improve an all histology-based classification, new genomics-based approaches are emerging [33, 39, 43–45].

A recent study of epigenetic-based unsupervised clustering in a 140-patient cohort divided meningiomas into favorable and unfavorable cohorts that differ on recurrence-free survival based on a 64-CpG loci methylation predictor [39]. In accordance with previous literature, loss of 1p, 6q, 14q, and 18q, and gain of 1q were associated with worse outcomes [15]. A separate methylation-based classification identified six distinct clinically relevant classes of meningiomas that had concordance with previously published genomic profiles and a stronger association with clinical outcome than the WHO grade system. Methylation signatures predictive of prognosis in WHO grade I tumors were further identified [44]. Transcriptomic analysis also suggested that *PTTG1* and *LEPR* expression could be used as prognostic markers independent of WHO grade, with their expression associated with more aggressive and possibly recurrent tumors [46]. Furthermore, loss of chromosome 1p36, and the *CCNB1* and *CDC2* genes have been associated with progression from grade I to higher grade [4, 23, 32]. Loss of histone H3K27me3 has also been linked with an increased risk of recurrence [30]. Overall, these studies support that the current histopathological classification of meningiomas is limited at providing definitive stratified prognostic information, particularly within a certain WHO grade.

The *Neurofibromin 2* (*NF2*) gene encodes the Merlin protein and is the first gene to be characterized as a meningioma driver. *NF2* is mutated in neurofibromatosis type II, a familiar tumor predisposition syndrome where up to 70% of patients develop meningiomas [24]. In animal models, *NF2* mutations have also been shown to drive tumorigenesis [38, 41]. Further, exposure to radiation therapy, a known risk factor for meningioma development, has been shown to drive structural aberrations in *NF2* [1]. Enrichment in *NF2* mutations has also been linked to features of high-grade meningiomas over low-grade [5]. Thus far, this molecular understanding has not translated into a different clinical management or significant improvement of prognosis assessment for patients with meningioma [29]. More recently, exome and whole genome sequencing analyses have identified non-*NF2* oncogenic drivers like the *POLR2A*, *TRAF7*, *KLF4*, *AKT1*, *FOXM1, SMARCB1* and *SMARCE1*, and *SMO* genes, implicating RNA polymerase, proapoptotic E3 ubiquitin ligase, PI3K, Wnt signaling, SWI/SNF chromatin remodeling complex, and the Hedgehog pathways in tumorigenesis and progression [7, 10, 11, 48, 52]. The role of each of these genes and molecules is still being elucidated (e.g. [6]), as they represent possible therapeutic targets. *FOXM1*, for instance, has been associated with worse clinical outcome as a marker for aggressive meningioma [52].

Currently, there is an unmet need created by the absence of a comprehensive classification system with precise prognostic information. Such a necessity is reinforced by the diverse fate of patients with grade I meningiomas, some of which recur [44]. A more in-depth characterization is necessary so that genome and transcriptome features associated with risk of progression to higher grades can be identified on first intervention (biopsy or surgery), perhaps warranting a more aggressive intervention and more structured follow-up. This question has been answered, at least in part, for anaplastic meningiomas (WHO grade III), where driver mutations in SWI/SNF complex genes and increased PRC2 activity are associated with worse prognosis [12].

In the present study, bioinformatics analysis of RNA-sequenced meningiomas was used to detect signatures associated with meningioma progression. We identify *GREMLIN 2 (GREM2)*, a regulator of the BMP pathway [3], and the small nucleolar RNAs (snoRNAs) *SNORA46* and *SNORA48* as novel, previously uncharacterized, downregulated candidate genes that may be linked to meningioma progression. Further, our results suggest that WHO grade I meningiomas that did not progress tend to be molecularly different from those that progressed; they also contain more RNA fusion transcripts and a significantly higher immune infiltrate than grade II or III tumors. We believe that a further characterization of these targets may yield significant prognostic and therapeutic advantages in the treatment of meningiomas.

## Materials and methods

### Meningioma samples

Meningioma samples were obtained at the Hospital of the University of Pennsylvania (HUP) and banked after intraoperative examination under IRB protocol approved by the University of Pennsylvania. After report review, each diagnosis was verified via histopathological review by a board-certified neuropathologist (MML and/or AV). Tumors with intermediate features (incomplete atypical features) and tumors with grade-defining histology (i.e. choroid or clear cell meningioma) were specifically excluded to amplify the effect of potential pathways implicated in meningioma progression in a more homogeneous cohort. Clinical and demographic information was obtained within IRB specifications, including prior history of radiation therapy. The discovery set consisted of 25 meningioma samples from 20 patients, which included de novo tumors (WHO I = 9, WHO II = 7, WHO III = 3), and progressive tumors (*n* = 6). Table 1 shows all selected patient samples along with corresponding information on WHO grade, gender, brain invasion, tumor location, and length of follow-up. The validation cohort, used for quantitative reverse transcription (qRT)-PCR only, was selected with similar criteria and consisted of 38 samples from 38 different patients (WHO I = 20, WHO II = 12, WHO III = 6). Tables 2 and Table 3 show all selected patient samples along with corresponding information on WHO grade, gender, brain invasion, and tumor location. For a subset of these (WHO I = 7) included in Table 3, length of follow up was known and superior to 5.4 years, as shown.

**Table 1:** Characteristics of meningioma samples included in the discovery set

Patient	BTTB ID	Gender	WHO Grade	Brain Invasion	Tumor location	Follow Up Years
1^a^	3043	M	I P	N	Left cerebellopontine angle	3
	3261		II S	N	Foramen magnum	
2	2536	M	I P	N	Right temporal lobe	7.5
	4995		II S	Y	Right temporal lobe	
3	1981	F	I P	N	Left parasagittal falcine convexity	9
	4302		II S	N	Left frontal lobe	
4^a^	1818		II DN	N	Right frontal lobe	9.4
	3254	F	III S	N	Posterior right frontal lobe	
	3526		III S	N	Right parietal lobe	
5^a^	3909	F	I NP	N	Right parietal lobe	5.4
6	2960_2	F	I NP	N	Right frontal lobe	7.6
7	3402_2	F	I NP	N	Left frontal parafalcine	1.6
8	3478	F	I NP	N	Left occipital lobe	6.6
9	3861	F	I NP	N	Left occipital lobe	5.5
10	2909_2	F	I NP	N	Right frontal lobe	7.8
11	2788^b^	F	II S	N	Right cerebellopontine angle	2.9
12	4084	F	II DN	Y	Parasagittal	3.8
13	4142	M	II DN	Y	Right parietal lobe	5.2
14	2993	F	II DN	Y	Left frontal lobe	8.3
15	2771	F	II DN	N	Right parietal lobe	3.6
16	2290	F	II DN	Y	Planum sphenoidale	2.5
17	4836	F	II DN	Y	Parasagittal	3.9
18	2516	M	III DN	N	Right frontal parasagittal	3
19	2860	F	III DN	Y	Left frontal lobe	7.3
20	5231	M	III DN	N	Right frontal lobe	2.8

I P Grade I that Progressed, *S* Secondary, *DN* De Novo, I NP Grade I that Never Progressed

^a^patient has prior history of radiation

^b^grade II that progressed from a grade I tumor

**Table 2:** Characteristics of meningioma samples included in the validation set

Patient	Gender	WHO Grade	Brain Invasion	Tumor location
1	F	I	N	Right frontal lobe
2	F	I	N	Right temporal lobe
3	F	I	N	Right frontal lobe
4	F	I	N	Right skull base
5	F	I	N	Left frontal lobe
6	M	I	N	Left temporal lobe
7	F	I	N	Left cerebellopontine angle
8	F	I	N	Left frontal lobe
9	F	I	N	Spine (T2,T3)
10	F	I	N	Left middle fossa
11	F	I	N	Sella/Pituitary
12	F	I	N	Left posterior cerebellum
13	F	I	N	Left temporal lobe
14	F	II	Y	Skull base
15	F	II	N	Right frontal lobe
16	F	II	N	Right frontal lobe
17	M	II	N	Left frontal lobe
18	M	II	N	Right dural base
19	F	II	N	Left frontal lobe
20	F	II	Y	Bilateral parasagittal dural base
21	M	II	N	Right frontal lobe
22	M	II	Y	Left frontal lobe
23	F	II	N	Right frontal and parietal lobes
24	F	II	N	Left frontal lobe
25	F	II	N	Right dural base
26	M	III	N	Right frontal lobe
27	M	III	N	Right frontal and parietal lobes
28	F	III	Y	Left frontal lobe
29	F	III	N	Right parietal lobe
30	F	III	Y	Bilateral frontal lobe
31	M	III	Y	Right parietooccipital lobe

**Table 3:** Characteristics of meningioma samples included in the validation set (Grade I NP)

Patient	Gender	Follow-up years	WHO Grade	Brain Invasion	Tumor Location
32	F	8.9	I	N	Right temporal lobe
33	F	7.4	I	N	Right parietal lobe
34	F	6.1	I	N	Right posterior frontal lobe
35	M	5.4	I	N	Left frontal lobe
36	M	5.4	I	N	Left frontal lobe
37	F	6	I	N	Occipital lobe
38	F	5.4	I	N	Right ventricle

### cDNA synthesis and quantitative RT-PCR

Total RNA was prepared from frozen meningioma samples using TRIzol (Invitrogen, Carlsbad, CA, USA). RNA was reversed transcribed into cDNA using Superscript III reverse transcriptase (Invitrogen) according to manufacturer’s recommendations. Real-time PCR was performed with a SYBR Green probe using a ViiA 7 (Applied Biosystems). Each sample was run in triplicate, and the RNA level for each gene was assessed by normalization to the expression of the housekeeping gene *GAPDH* or *RN18S1*. The primer sequences for the various genes used in this study are available on request.

### RNA sequencing

The library preparation and sequencing process were performed at the Beijing Genomics Institute (BGI) facility located in the Children’s Hospital of Philadelphia (CHOP). Library construction was performed by following Illumina stranded RNA-seq workflow (TruSeq Stranded Total RNA Library Prep Kit, Cat# RS-122-2201). Briefly, 200 ng of total RNA is treated with Ribo-zero kit to remove ribosomal RNA, and then purified. RNA is then fragmented and converted to cDNA with RT reaction. Subsequent steps include end repair, addition of an “A” overhang at the 3′ end, and ligation of the indexing-specific adaptor, followed by purification with Agencourt Ampure XP beads. The library is then amplified and purified with Ampure XP beads. Size and yield of the bar-coded libraries are assessed on the LabChip GX, with an expected distribution around 260 bp. Concentration of each library is measured with real-time PCR. Pools of indexed library are then prepared for cluster generation and 100 bp by 100 bp paired end sequencing on the Illumina HiSeq 2000.

### Bioinformatics analysis of the RNA-seq data

The samples were sequenced at the sequencing core BGI@CHOP. Randomly fragmented DNA sequences were run through libraries prepared for paired end sequencing on an Illumina HiSeq 2000. The raw RNA sequencing reads were run through the QC checks by FastQC and then mapped to reference genome (h19) by aligner STAR. After that, HTSeq was applied to detect the sequencing read count for each gene. DESeq2 was applied to detect the differential expression level for each gene between different groups. Principal component analysis (PCA) was used to characterize the RNA-seq data set; Hierarchical clustering was performed using thousands of genes with the highest variability across the samples. For the discovery set, the differential expression between different groups was considered for the Gene Set Enrichment Analysis (GSEA) [49]. STAR-Fusion [21] was applied for the RNA-seq transcript fusion detection.

### Amplification and sequencing of fusion transcripts

cDNA was synthesized from total RNA as previously described and then amplified by standard PCR using Taq DNA polymerase with fusion flanking primers. After gel recovery, the fragments were sequenced with *NF2* exon primers.

### Tissue microarrays

Diagnostic slides were reviewed to ensure all tumors were graded according to the 2016 WHO classification of meningiomas [33]. A total of 71 meningiomas were used in the construction of three tissue microarrays (TMA) including 15 patients whose tumors recurred with progression to a higher grade (13 patients with progression from grade I to grade II and 2 patients with progression from grade II to grade III) and 42 patients without tumor progression (18 WHO grade I, 17 WHO grade II, and 7 WHO grade III). Representative area(s) of each tumor was/were selected and circled on H&E stained-slides, and 1 mm cores were cut from the corresponding paraffin-embedded blocks. In the majority of cases, multiple cores were taken from the same tumor. Control tissues used in the construction of the TMA were obtained from histologically unremarkable brain autopsy specimens (cortex, cerebellum and meninges controls) and from surgical tonsillectomy specimens. Sections were cut at 5 μm from each TMA and used for hematoxylin and eosin staining and immunohistochemistry.

### Immunohistochemistry and quantification

Immunohistochemical staining was performed on 5 µm sections using standard methods. Briefly, slides were deparaffinized in xylene and rehydrated through graded alcohols. Antibodies to CD45 (leukocyte common antigen) were used (1: 200, Dako), and detection of the antibodies was performed using a chromogenic substrate, diaminobenzene (Dako). Slides were counterstained with hematoxylin, dehydrated through a series of ascending concentrations of ethanol and xylene, and then coverslipped.

Immunohistochemical staining for CD45 was evaluated by counting the number of immunopositive cells within tumor areas in each 1 mm tissue core. The pathologist performing the counting was blinded to tumor grade and progression/non-progression status. A core was excluded if it contained less than 90% solid tumor. In instances where more than one core was present from a tumor, counts were averaged across all cores from the same tumor to give a single count. Counts were then averaged for grade I and grade II/III tumors; de novo tumors were grouped separately from tumors that progressed.

## Results

### Transcriptional profile across different meningioma WHO grades

The discovery set chosen for RNA-seq (Table 1) consisted of 25 meningioma samples from 20 patients, which included: samples from grade I tumors that did not progress to higher grade (grade I never progressed or “I NP”), samples from de novo grade II or III tumors, samples from patients with tumors which progressed from grade I (grade I progressed or “I P”) to grade II (secondary or “II S”) (patients #1–3) and samples from a patient with a tumor progressing from grade II to III (patient #4). The majority of patients (76%) were female, which is consistent with the well-known higher incidence of meningioma in women. Median age at surgery was 47.5 years (range 27–86). Eight tumors (38%) had brain invasion (WHO grade II or III tumors). Among the 20 patients selected, 5 (25%) had radiation therapy for a prior malignancy.

**Fig. 1:**
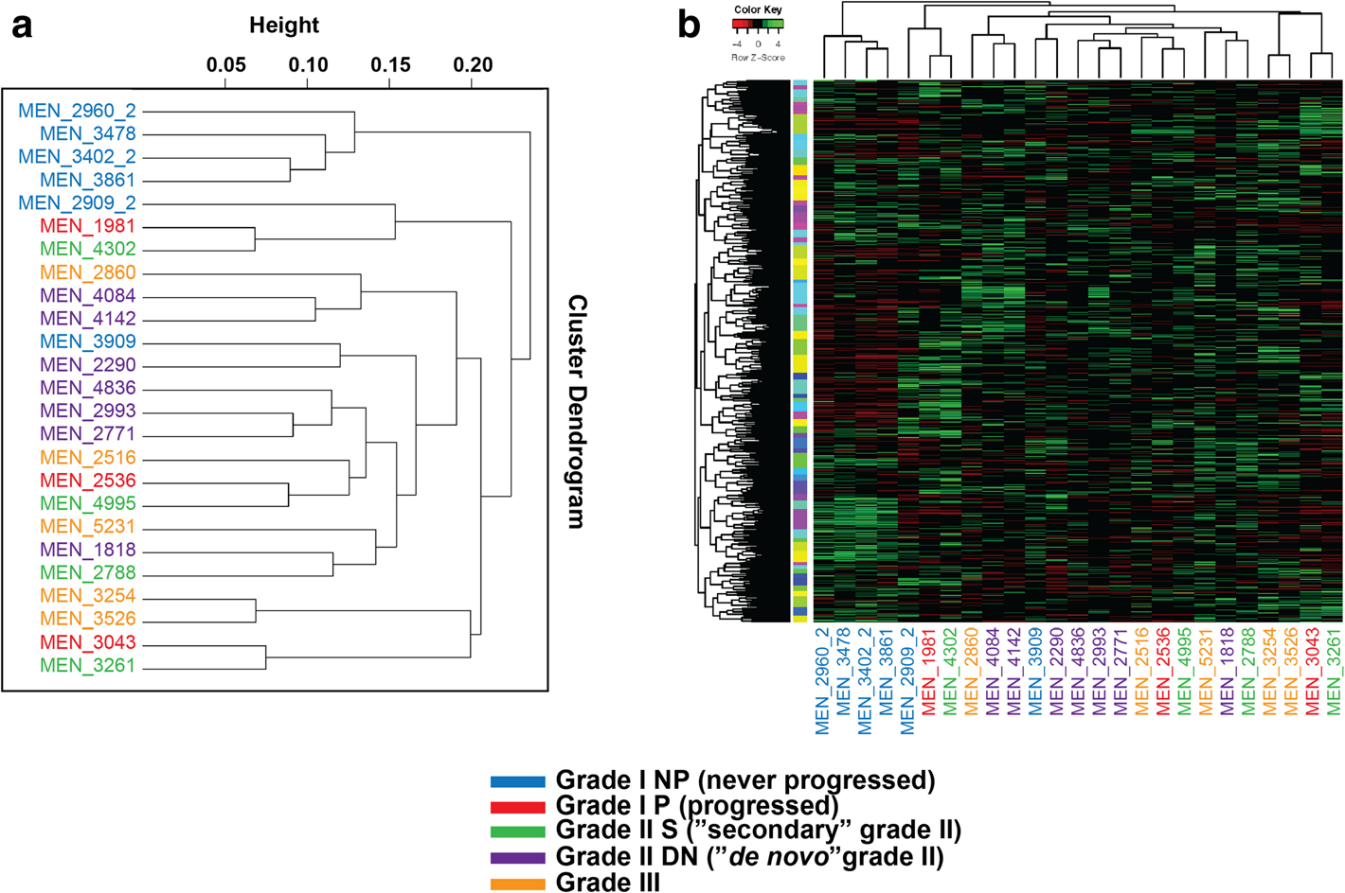
Unsupervised clustering of the RNA-seq data obtained from the 25 meningioma samples from 20 patients constituting the discovery set. **a** Cluster dendrogram for all of the meningioma samples. **b** Expression heatmap for the 25 meningioma samples ordered by unsupervised clustering

The RNA-seq data were analyzed using unsupervised clustering approaches [2, 42, 53]. Interestingly, 4 of the 6 grade I tumors that did not progress (I NP) clustered together and separately from the other tumor samples (Fig. 1a). Grade I tumors that progressed (I P) clustered together with their respective secondary grade II tumor, suggesting that secondary tumors mostly resemble their primary. Generally, grade II tumors that arose de novo (DN) tended to mix with grade II tumors that progressed from grade I (grade II S). Grade III tumors did not cluster together, suggesting that they represent a more heterogeneous class (Fig. 1a). This pattern was observed when differential expression level across 2000 genes was measured and compared (Fig. 1b). When only grade I tumors (i.e. both I P and I NP) were analyzed via unsupervised clustering to avoid possible confounding results from other grade tumors, 4/6 I NP were clearly separate from the remainder of the cohort; while 2/6 clustered more closely to I P (Additional file 1: Figure S1a). When grade II tumors were analyzed independently, a clustering pattern was harder to discern, albeit 5 of the 7 grade II DN tumors formed 2 clusters separately from the grade II S ones (Additional file 1: Figure S1b).

**Fig. 2:**
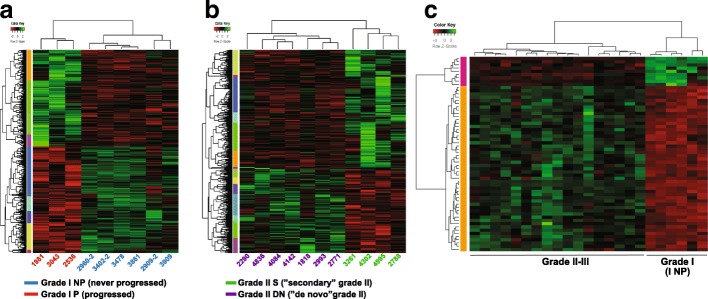
Supervised clustering of the RNA-seq data obtained from the 25 meningioma samples from 20 patients constituting the discovery set. **a** Comparison between grade I samples that progressed to higher grade and those that did not. **b** Comparison between grade II meningiomas from a previous grade I (‘secondary’) and those arising de novo. **c** Comparison between grade I meningiomas that never progressed and grade II-III meningiomas

These results suggest, at least in part, that grade I tumors that progress to higher grades are molecularly different from grade I tumors that did not progress. Therefore, we identified a set of genes (Additional file 2: Table S1, Fig. 2a) that differ between these two prelabelled subclasses of grade I tumors, supporting the findings of unsupervised clustering. We further performed Gene Set Enrichment Analysis (GSEA) [34] and found that the differentially expressed genes between I P and I NP tumors were significantly overrepresented in signatures associated with ‘Hypoxia’, ‘EGF signaling’, ‘HRAS oncogenic’ and ‘Tumor angiogenesis’ (Additional file 1: Figure S2). These results shed light on the numerous pathways that distinguish these two groups. For instance, hypoxia-associated pathways and HIF1 signaling (a downstream effector involved in the response to hypoxia [31]) are found in I P samples. On the other hand, reduced TGFβ signaling is found in I NP samples (Additional file 1: Figure S2).

Grade I NP tumors also differed from grade II or/and III meningiomas in a set of differentially expressed genes, as determined by supervised clustering (Additional file 1: Figure S2c, Additional file 3: Table S2, Additional file 4: Table S3 and Additional file 5: Table S4).

The unsupervised clustering analysis pointed towards de novo grade II tumors being potentially different from secondary grade II that arise from a prior grade I tumor, as specific transcriptome signatures for each subtype were identified with supervised clustering (Additional file 1: Figure S2b, Additional file 6: Table S5).

### Grade-specific differentially expressed genes across meningiomas

To validate the results identifying differentially expressed genes between grade I and grades II/III tumors, we selected a new set of meningioma samples that constitute a second (validation) cohort (Tables 2 and 3); q RT-PCR was performed on these samples. The validation set consisted of 38 meningioma samples, which included grade I through III tumors with no progression. A special group consisted of grade I NP meningiomas for which follow-up was at least 5.4 years (average follow-up of 6.4 years), given the importance of lengthy follow-up to exclude recurrence and/or progression (Table 3). Out of the 38 patients, 28 (74%) were female. Median age at surgery was 55 years (range 34–88). Six (16%) tumors had brain invasion, three with grade II meningiomas and three with grade III.

**Fig. 3:**
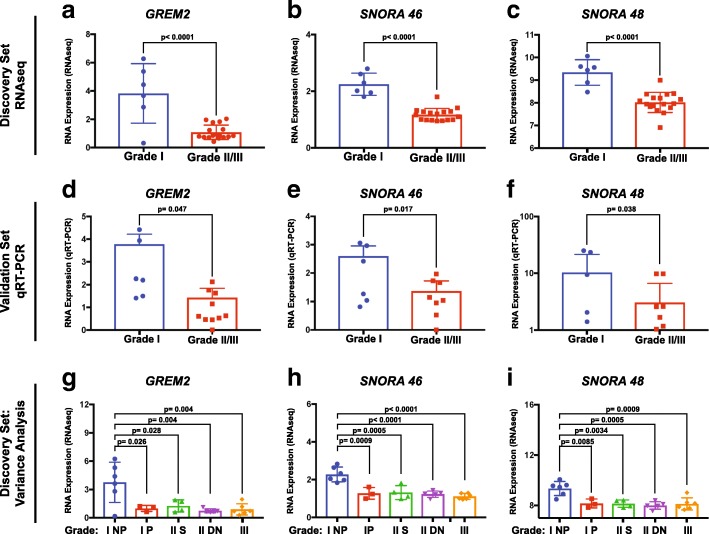
Decreased expression of *GREM2*, *SNORA46* and *SNORA48* in meningioma progression. **a-c** Box plot showing the normalized expression of *GREM2*, *SNORA46* and *SNORA48* genes in the discovery cohort by RNA-seq. **d-f** Analyses of the relative RNA levels of *GREM2*, *SNORA46* and *SNORA48* in the validation cohort (*n* = 20 grade I, *n* = 12 grade II, *n* = 6 grade III). Note that the expression of these genes is decreased from grade I to grade II-III. **g-i** Variance analysis of the normalized expression of *GREM2*, *SNORA46* and *SNORA48* genes in the discovery cohort by RNA-seq, across each tumor grade

Among the numerous genes differentially expressed across meningioma grades, we identified *GREM2*, a regulator of the BMP pathway, and the snoRNAs, *SNORA46* and *SNORA48*, as being significantly reduced in meningioma progression by RNA-seq analysis (Fig. 3a-c). In particular, *GREM2* levels in grade I tumors were significantly higher than in grades II and III (*p* < 0.0001); similar differential expression was found for *SNORA46* (*p* < 0.0001) and *SNORA48* (*p* < 0.0001). We confirmed this differential expression in the validation cohort via qRT-PCR (Fig. 3d-f). *GREM2*, *SNORA46*, and *SNORA48* were each expressed at significantly higher levels in grade I meningiomas than grade II and III (*p* = 0.047, *p* = 0.017, and *p* = 0.038, respectively; one-tailed t-test). When analyzing these genes’ expression across each meningioma grade, we observed how *GREM2*, *SNORA46*, and *SNORA48* were expressed at significantly higher levels in I NP when compared to I P, secondary or de novo grade II, and grade III. On the other hand, no difference was found among other grades (Fig. 3 g-i).

### Identification of novel *NF2* fusion transcripts

Gene fusions have been known to play a major role in tumorigenesis since the discovery of the Philadelphia chromosome in chronic myeloid leukemia. More recently, their role has been investigated in brain neoplasms as well. In ependymomas, *C11orf95*-*RELA* fusions drive oncogenic nuclear factor-κB (NF-κB) to transform neural stem cells into tumor cells [40]. In IDH-wildtype gliomas, *FGFR-TACC* fusions have been investigated as possible therapeutic targets, with inhibition of the *FGFR* fusion transcript yielding promising preliminary results [13]. The role of fusions in meningiomas, however, is still under investigation [1, 17].

**Fig. 4:**
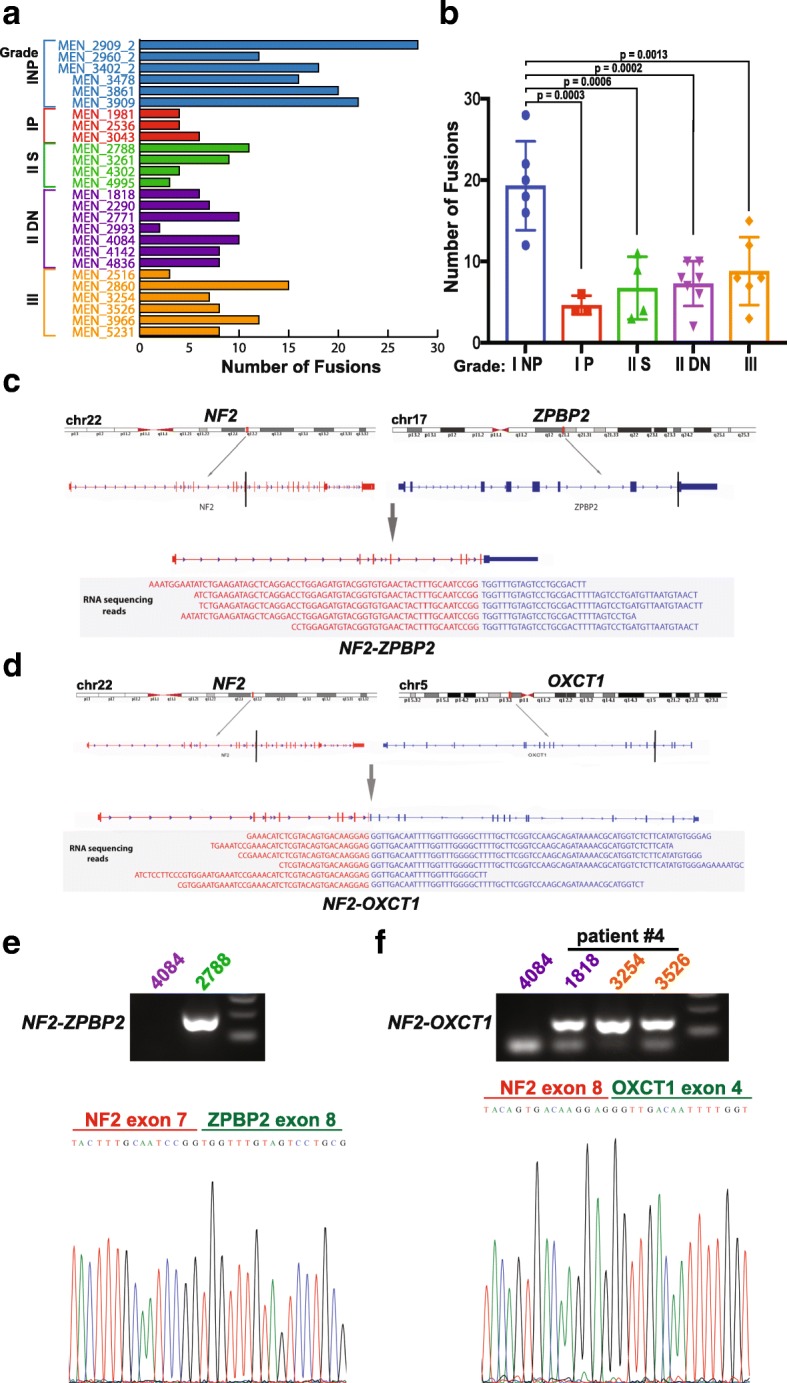
New *NF2* fusion transcripts identified in meningiomas. **a-b** Number of fusions transcripts identified through RNA-seq (**a**) and the significance across grades (**b**). **c, d** *NF2-ZPBP2* (chromosomes 22q-17q, **c**) and *NF2-OXCT1* (chromosomes 22q-5p, **d**) fusion transcripts lead to truncated and non-functional NF2 protein. Asterisks mark position of primers used for RT-PCR (**e, f**) to validate the fusion transcripts. **e, f** The *NF2–ZPBP2* (**e**) and *NF2-OXCT1* (**f**) fusion transcripts identified by RNA-seq were validated in the corresponding meningioma samples using RT–PCR and Sanger sequencing. All three tumors from patient #4 harbored the fusion *NF2-OXCT1* (**f**)

We used STAR-fusion [21] to identify potential novel fusion transcripts and to determine if fusion events were also differentially present in meningiomas of different grades [14]. We observed that grade I meningiomas that never progressed have a significantly higher number of rearrangements as identified by sequencing than grade I meningiomas that eventually did progress or grade II (both de novo and secondary) and grade III meningiomas, which had a smaller fusion burden (one-way ANOVA with Tukey’s multiple comparison test; Grade I NP vs. Grade I P, *p* = 0.0003; Grade I NP vs. Secondary grade II, *p* = 0.0006; Grade I NP vs. de novo Grade II, *p* = 0.0002; Grade I NP vs. Grade III, *p* = 0.0013) (Fig. 4a-b). No significant difference was found among grade I meningiomas that progressed, grade II (both de novo and secondary), and grade III. Among the identified fusion events (Table 4), we selected two novel *NF2*-involved fusion products not observed so far in meningioma or other tumors: *NF2*—*ZPBP2* (*Zone Pellucida Binding Protein 2*) (chromosomes 22q and 17q) and *NF2*—*OXCT1* (*3-oxoacid CoA-transferase*) (chromosomes 22q and 5p) (Fig. 4c and d, respectively) which led to a truncated and non-functional *NF2* transcript. Of note, the *NF2*—*OXCT1* fusions all occurred in meningiomas that progressed to higher grade or were secondary to progression. To validate these new fusions, we designed primer pairs specific for each fusion transcript and analyzed our samples with RT-PCR. We found that, indeed, there was clear concordance between the RNA-seq data and RT-PCR analyses (Fig. 4e and f for *NF2—ZPBP2* and *NF2—OXCT1*, respectively). Of note, we observed that the novel *NF2—OXCT1* fusion was found in case #1818 and its two instances of recurrence, i.e. cases #3254 and #3526 (Fig. 4f), suggesting the importance of this fusion event that was maintained in all 3 resected tumors from the same patient.

**Table 4:** Relevant fusion events and frequency among tumor classes

Fusion identified	Number of events and tumor class					
*NF2-ZPBP2*	II S					
*NF2-OXCT1*	II DN^a, b^	III^a, b^	III^a, b^			
*C10orf112-PLXDC2*	I NP	I P^b^	III			
*GAB1-HHIP-AS1*	I NP^b^					
*HHIP-AS1-GAB1*	I NP^b^					
*KANSL1-ARL17A*	I NP	I NP	II S	II DN	II DN	III
*MLLT3-CNTLN*	II DN^a, b^	III^a, b^	III^a, b^			
*RP11-444D3.1--SOX5*	I NP	I NP				
*SAMD5-SASH1*	I NP	I NP	I NP	I NP		

^a^fusion found across all samples (de novo and recurrent) from the same patient

^b^patient has prior history of radiation

In addition to fusions implicating the *NF2* gene, other fusion transcripts were observed in more than one meningioma sample including *C10orf112*-*PLXDC2* (found in I NP, I P, and grade III); *GAB1*-*HHIP*-*AS1* and *HHIP-AS1*--*GAB1* found in a I NP sample; *KANSL1-ARL17A* (found in two I NP, two de novo grade II, one secondary grade II, and one grade III); *MLLT3*-*CNTLN* (one de novo grade II and two secondary grade III); *RP11*-*444D3*.1-*SOX5* (two grade I NP, one grade III); and *SAMD5*-*SASH1* (found in four grade I NP samples) (as summarized in Table 4). Of note, *NF2-OXCT1* and *MLLT3i-CNTLN* fusions are found across all samples from the same patient (patient #4), i.e. for primary and secondary samples. None of these fusion transcripts was observed in pediatric brain tumors sequenced within the Children Brain Tumor Tissue Consortium (CBTTC) (data available on CAVATICA).

### The immune microenvironment is differentially activated across meningioma grades

**Fig. 5:**
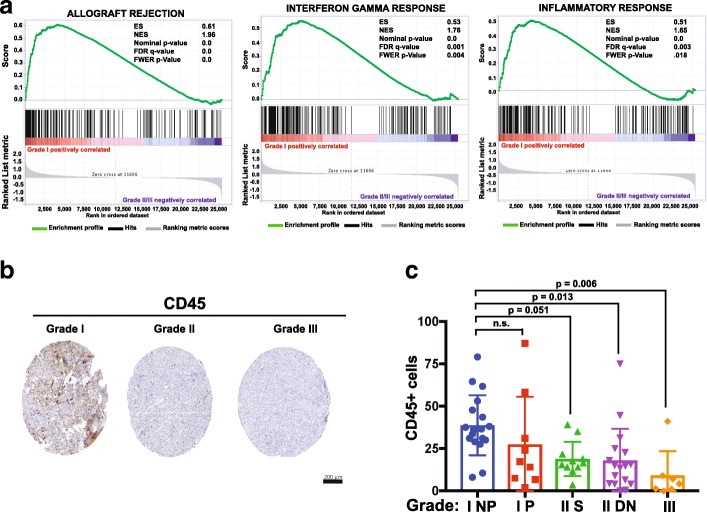
Gene expression signatures involved in immune function are positively linked to grade I meningioma tumors. **a** GSEA analysis shows the significant correlation of the genes up-regulated in grade I NP tumors with those gene sets associated with ’Allograft Rejection,’ ‘Interferon Gamma Response,’ and ‘Inflammatory Response.’ **b** Representative images from immunohistochemical analysis of CD45+ cells in tissue microarrays containing de novo meningioma samples of different grades (18 WHO grade I, 17 WHO grade II, and 7 WHO grade III). **c** Quantification of the number of CD45+ cells by tumor grade

To determine if gene signature associated with different biological processes may be linked to meningioma progression, we performed GSEA analysis on the differentially expressed genes between grade I and grade II/III tumors. Surprisingly, the genes that distinguished grade I from grade II/III tumors were significantly overrepresented in gene signatures associated with different immune responses; this association was much reduced in grade II and III meningiomas. In particular, a strong association was found with ‘allograft rejection,’ ‘interferon gamma response,’ and ‘inflammatory response’ gene sets (*p* < 0.0001) (Fig. 5a).

To further validate the finding that the host immune response varies among different meningioma grades, we set out to analyze if the number of infiltrating immune cells was different between I NP and grade II/III tumors. We therefore generated 3 tissue microarrays (TMAs) from 71 meningiomas. Included within these TMAs were 15 patients whose tumors recurred with progression to a higher grade (13 patients with progression from grade I to grade II and 2 patients with progression from grade II to grade III) and 42 patients without evidence of tumor progression at an average follow-up of 6 years (18 WHO grade I, 17 WHO grade II, and 7 WHO grade III). These TMAs were stained for CD45 (leukocyte common antigen), a marker widely used to examine general immune infiltration and inflammation [37]. Grade I NP meningiomas had a noticeable CD45 positive infiltrate, with significantly higher numbers of CD45 positive inflammatory cells compared to grade II (secondary or de novo) and III tumors. This result was consistent with the GSEA analysis indicating increased immune activity in low grade meningioma microenvironment (one-way ANOVA with Tukey’s multiple comparison test; grade I NP vs. grade I P, n.s.; grade I NP vs. secondary grade II, *p* = 0.05; grade I NP vs. de novo grade II, *p* = 0.013; grade I NP vs. grade III, *p* = 0.006) (Fig. 5b-c).

## Discussion

To the best of our knowledge, this is one of the first studies that compares grade I meningioma tumors that did not progress to grade I tumors that did progress. Results presented in this study suggest that grade I meningiomas that never progress to higher grade (grade I NP) have a transcriptome different not only from higher grade tumors but also from grade I tumors that do progress to higher grade (grade I P). Overall, grade I NPs clustered the furthest from higher grade meningiomas, which clustered closer together (Fig. 1). Admittedly, the cohort used in our study is limited and further studies with additional cases of grade I tumors that progressed are needed to better understand the fate of grade I meningiomas. Among the genes that we have identified as being differentially expressed between grade I and grade II-III tumors are *GREM2*, *SNORA46*, and *SNORA48*, found at higher levels in low grade meningiomas than in higher grade tumors.

*GREM2,* also known as *PRDC* (Protein Related to Dan and Cerberus) encodes for a member of the DAN family of secreted proteins which constitute a subgroup of inhibitors of the BMP pathway [36]; GREM2 appears to be more efficient in inhibiting BMP2 and BMP4 than TGFβ [50]. Its function during development is not well understood, although it has been associated with osteogenesis and cardiac development [35, 54]; GREM1 has been associated with increased vascular proliferation and carcinogenesis in diffuse intrinsic pediatric glioma and other gliomas [8, 20, 47]. GREM2 function in cancer biology and in meningioma is not known. Our results suggest that decrease of *GREM2* expression may be linked to acquisition of malignant behavior in meningioma. Decrease of GREM2 expression may lead to increase BMP signaling, thus suggesting a role for BMP signaling in meningioma progression. BMP2/4 expression was examined in early passage human primary meningioma cells and was found to be expressed in a majority of these cells [27]; however, the role of BMP signaling in meningioma tumors is still not known [26]. In this context, it is important to note that defects in TGFβ and/or BMPs signaling have been associated with meningioma progression and it has been suggested that a decrease of the inhibitory regulation of TGFβ may be linked to meningioma progression [26]. How the decrease of *GREM2* expression that we observed in grade II-III meningiomas may lead to defects in BMP and/or TGFβ signaling and to tumor progression remains to be determined.

The role of non-coding small nucleolar RNAs (snoRNAs) in human diseases has been increasingly appreciated. Indeed, mutations in the *SNORD118* were identified in patients with cerebral microangiopathy leukoencephalopathy, and defects in expression of several snoRNAs are linked to the Prader-Willi syndrome [9, 25]. Interestingly, snoRNAs have also been found to be involved in tumorigenesis [51, 55]. A recent global analysis of the expression of snoRNAs in 31 different cancer types showed that the snoRNA expression is generally decreased in tumors when compared to normal tissues and identified *SNORD46* as a tumor suppressor [19]. However, other snoRNAs have been shown to act more like oncogenes: indeed, *SNORD14D* or *SNORD35A* are required for leukemia development in vivo in certain models [56]. These results emphasize the complex role of snoRNAs in cancer which is most likely cell-context specific and still remains, to a large degree, to be determined. In our study, the higher levels of *SNORA46* and *SNORA48* found in low-grade meningiomas compared to higher-grade tumors point towards their role as cancer or tumor progression suppressors. Their exact mechanism of function, however, remains unclear.

In addition to these novel markers of meningioma progression, our study also identified several fusion transcripts that have not previously been described in meningioma and/or other cancers. Our data suggest that grade I NP tumors may also differ from other grade I that progressed and higher-grade tumors by their number of fusion transcripts. Among the fusion transcripts identified, several involved the *NF2* gene, the most common gene associated with meningioma tumorigenesis. Surprisingly, in our study, we found a higher burden of fusions in grade I NP meningiomas. In addition, the presence and number of *NF2* fusions were not linked to prior radiation treatment, as *NF2* fusions were also observed in patients that were naïve to radiation therapy. Others have shown that radiation therapy induces specific *NF2* mutational and structural variants [1, 44]. Of the fusions identified, we report two novel fusion products that result in a truncated and non-functional *NF2* transcript. Future analyses of the function of these transcripts will most likely help shed light on interpreting meningioma progression to higher grades.

In addition to identifying novel molecules with a potential role in meningioma progression, our study has also determined that gene signatures linked to immune response are significantly represented in grade I vs grade II-III tumors. We have validated these RNA-seq results by immunohistochemistry and demonstrated that the immune infiltration as visualized by the presence of CD45 positive cells decreases significantly between grade I and grade II-III meningiomas. These results are in agreement with a recent report also showing a decrease of CD45 positive cells between grade I and grade II-III meningiomas [16]. Other inflammatory microenvironment elements have been associated with meningioma grade – for example, the immune modulatory molecule PD-L1 (CD274, which has an immune avoidance role) has been associated with anaplastic meningiomas [16]. Similarly, others have reported an increased PD-L1 and CD163 expression in higher grade meningiomas, thus suggesting a role for immune avoidance in higher grade tumors associated with worse prognosis [16, 22, 28]. Blocking these pathways in high grade tumors may represent a novel therapeutic strategy.

## Conclusions

In conclusion, we have used RNA-seq to establish the transcriptome of a cohort of meningioma samples including samples from patients whose tumor progressed from benign to malignant. Our study identified a transcriptional signature that distinguished between grade I tumors that will progress from those that will not. In addition, we have identified novel potential regulators of tumor progression, including the GREM2 and snoRNAs genes. We also shown that the number of fusion transcripts is higher in grade I tumors that do not progress compared to all the other tumors and further identified novel NF2 fusion products. Finally, we reported how grade I tumors differ from more malignant ones in immune infiltration, significantly higher in benign samples.

## Additional files

Additional file 1: **Figure S1.** Unsupervised clustering of the RNA-seq data obtained from grade-specific tumor samples only. a) Cluster dendrogram for all the grade I meningioma samples. b) Cluster dendrogram for all the grade II meningioma samples. **Figure S2.** GSEA analysis shows the significant correlation of the genes up-regulated in grade I P vs I NP tumors with those gene sets associated with ’Hypoxia,’ ‘EGF signaling,’ ‘HRAS oncogenic,’ ’Tumor angiogenesis‘ and ‘TGFb signaling Down.’ (PDF 1858 kb)

Additional file 2: **Table S1.** List of significantly differentially expressed genes between grade I NP and I P meningiomas, as identified by RNA-seq. (PDF 540 kb)

Additional file 3: **Table S2.** List of significantly differentially expressed genes between all grade I and all grade II meningiomas, as identified by RNA-seq. (PDF 426 kb)

Additional file 4: **Table S3.** List of significantly differentially expressed genes between all grade I and all grade III meningiomas, as identified by RNA-seq. (PDF 659 kb)

Additional file 5: **Table S4.** List of significantly differentially expressed genes between all grade I and grade II-III meningiomas, as identified by RNA-seq. (PDF 581 kb)

Additional file 6: **Table S5.** List of significantly differentially expressed genes between grade II S. and II DN meningiomas, as identified by RNA-seq. (PDF 255 kb)
