# Supplementary material for: Transcriptome signatures associated with meningioma progression
Source: Acta Neuropathol Commun. 2019 Apr 30;7:67. doi: 10.1186/s40478-019-0690-x (PMC6489307; doi:10.1186/s40478-019-0690-x)
Supplement: Supplementary file 1 — Figure S1. Unsupervised clustering of the RNA-seq data obtained from grade-specific tumor samples only. a) Cluster dendrogram for all the grade I meningioma samples. b) Cluster dendrogram for all the grade II meningioma samples. Figure S2. GSEA analysis shows the significant correlation of the genes up-regulated in grade I P vs I NP tumors with those gene sets associated with ’Hypoxia,’ ‘EGF signaling,’ ‘HRAS oncogenic,’ ’Tumor angiogenesis‘ and ‘TGFb signaling Down.’ (PDF 1858 kb) [file 40478_2019_690_MOESM1_ESM.pdf]

Supplementary Material

Supplementary Figure 1: Unsupervised clustering of the RNA-seq data obtained from grade-specific tumor samples only.

a) Cluster dendrogram for all the grade I meningioma samples.

b) Cluster dendrogram for all the grade II meningioma samples.

Supplementary Figure 2: GSEA analysis shows the significant correlation of the genes up-regulated in grade IP vs INP tumors with those gene sets associated with 'Hypoxia,' 'EGF signaling,' 'HRAS oncogenic,' 'Tumor angiogenesis' and 'TGFβ signaling Down.'

Supplementary Table 1: List of significantly differentially expressed genes between grade INP and IP meningiomas, as identified by RNA-seq.

Supplementary Table 2: List of significantly differentially expressed genes between all grade I and all grade II meningiomas, as identified by RNA-seq.

Supplementary Table 3: List of significantly differentially expressed genes between all grade I and all grade III meningiomas, as identified by RNA-seq.

Supplementary Table 4: List of significantly differentially expressed genes between all grade I and grade II and III meningiomas, as identified by RNA-seq.

Supplementary Table 5: List of significantly differentially expressed genes between grade II S. and II DN meningiomas, as identified by RNA-seq.

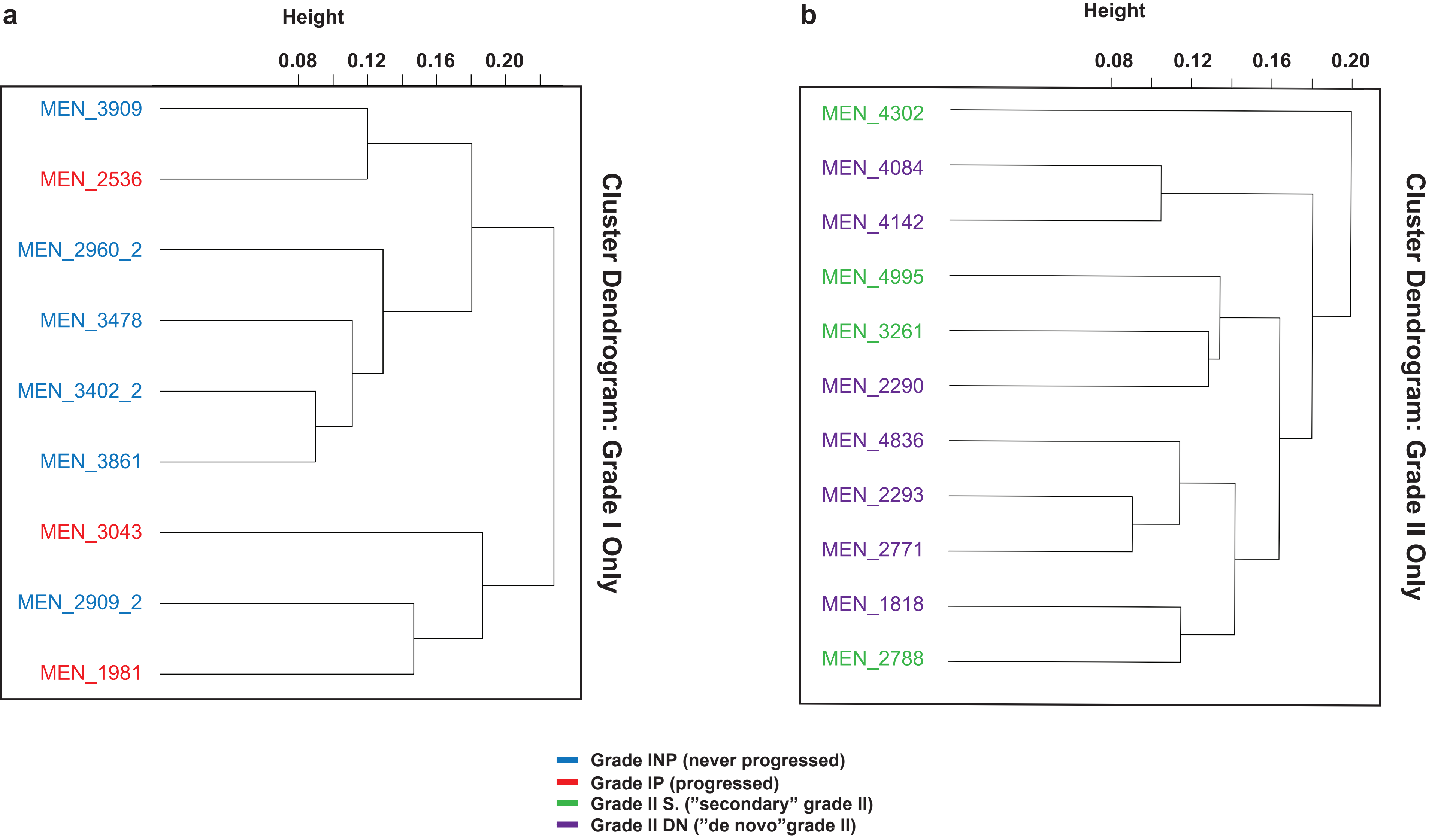

Supplementary Figure 1

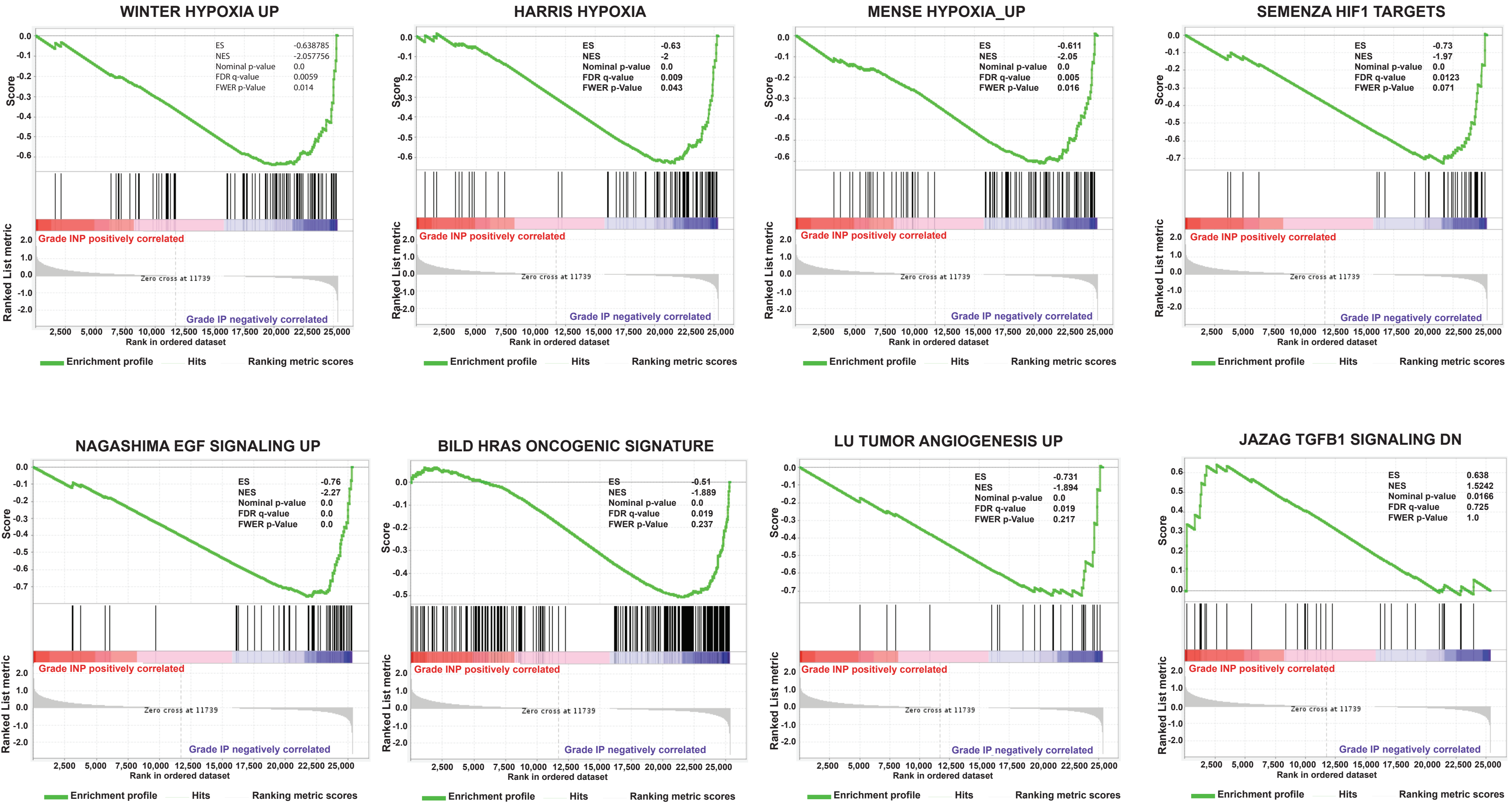

Supplementary Figure 2
