# Supplementary material for: Transcriptome signatures associated with meningioma progression
Source: Acta Neuropathol Commun. 2019 Apr 30;7:67. doi: 10.1186/s40478-019-0690-x (PMC6489307; doi:10.1186/s40478-019-0690-x)
Supplement: Supplementary file 2 — Table S1. List of significantly differentially expressed genes between grade I NP and I P meningiomas, as identified by RNA-seq. (PDF 540 kb) [file 40478_2019_690_MOESM2_ESM.pdf]

Supplementary Table 1: Differentially expressed genes between GR INP and GR IP meningiomas

Gene	baseMean	log2FoldChar	pvalue	padj
RNA5-8S5	885.336646	-9.8466041	1.52E-144	2.34E-140
DNM3OS	828.771988	6.18663848	3.43E-58	2.64E-54
TENM2	6824.41363	4.90394961	3.78E-33	1.94E-29
PLA2G2A	139.09861	-7.5651604	5.19E-23	1.99E-19
MANBAL	564.732921	-1.6452591	3.10E-20	9.51E-17
CTTNBP2	336.049479	3.11484964	2.57E-18	6.58E-15
P2RY14	160.68115	4.12019978	1.55E-16	3.39E-13
U2AF1	573.471554	-1.9691871	1.94E-16	3.73E-13
SNORA54	182.314513	3.33873547	2.38E-16	4.06E-13
CHMP4B	1711.48836	-1.7366269	1.32E-15	2.03E-12
DPYSL4	138.510643	3.99572332	3.78E-15	5.28E-12
COL9A3	4653.86591	-3.096956	1.85E-14	2.36E-11
RAPGEFL1	279.953527	-2.0366165	2.71E-14	3.20E-11
LOC1019293	143.425589	5.60726059	5.04E-14	5.17E-11
SAFB	1273.46186	-0.8654561	4.96E-14	5.17E-11
NOP56	1254.01513	-1.0083777	6.17E-14	5.92E-11
RPL28	4946.35932	-1.7121877	1.98E-13	1.79E-10
RAB3D	1463.84019	-2.2187193	2.69E-13	2.30E-10
RNA45S5	4296906.94	-5.2853054	3.09E-13	2.50E-10
PRSS30P	90.995885	5.16517859	3.90E-13	3.00E-10
LNX1	204.59076	3.48141932	4.79E-13	3.51E-10
ANP32B	1025.49234	-2.0150009	6.76E-13	4.72E-10
PLD4	399.921466	2.73818441	7.74E-13	5.17E-10
PRR13	1085.75502	-1.2511954	1.18E-12	7.57E-10
DDX3Y	693.200041	-5.9216418	3.09E-12	1.83E-09
UTY	489.024406	-5.890448	3.00E-12	1.83E-09
FKBP9	3568.7939	1.22839642	4.10E-12	2.33E-09
RPS4Y1	779.017278	-5.8649085	5.73E-12	3.14E-09
FGL2	38764.1816	4.33218652	7.23E-12	3.83E-09
PI16	293.32581	5.27608057	9.93E-12	5.08E-09
HDAC9	1058.41779	3.18043924	1.04E-11	5.13E-09
RPL23P8	54.9962623	-2.6050175	1.37E-11	6.60E-09
ZFY	169.761813	-5.5239122	1.51E-11	7.01E-09
EMD	642.711971	-1.6337243	1.76E-11	7.96E-09
TEK	795.752892	2.32010306	1.94E-11	8.53E-09
PDE1C	348.18298	4.12513748	4.14E-11	1.77E-08
CCDC124	341.5989	-1.3509962	5.95E-11	2.41E-08
COL1A2	90688.0657	2.54311016	5.95E-11	2.41E-08
PLP1	513.917414	4.5532474	6.42E-11	2.49E-08
RAB11B	1012.33599	-1.0406461	6.48E-11	2.49E-08
PPDPF	2231.22293	-1.7949783	1.06E-10	3.89E-08
ZNF300P1	82.3185336	3.68636274	1.05E-10	3.89E-08
ELN	6286.02073	4.41963246	1.10E-10	3.93E-08
TENM3	4071.81946	4.77969181	1.21E-10	4.23E-08
INMT	10896.0367	4.99677873	1.26E-10	4.29E-08
C20orf24	113.123554	-1.8776771	1.36E-10	4.44E-08
PDGFRB	7388.86748	2.07580192	1.35E-10	4.44E-08

TAF15	886.092138	-1.1173412	1.70E-10	5.43E-08
GDF10	99.1980321	4.90736834	2.47E-10	7.75E-08
CACNA1D	697.832396	4.20613414	2.58E-10	7.92E-08
SUMO3	1387.46153	-0.944086	3.50E-10	1.05E-07
EGFL6	6044.57704	4.94592072	3.76E-10	1.11E-07
MYCL	220.075451	3.20331898	4.64E-10	1.34E-07
FGF12	96.9087083	2.80060331	4.83E-10	1.37E-07
HM13	1724.67794	-1.3776734	9.86E-10	2.75E-07
TVP23C	516.118506	1.07913691	1.02E-09	2.81E-07
GPC5-AS1	421.123985	4.24756768	1.06E-09	2.86E-07
NFE2L3	144.214122	2.31342332	1.30E-09	3.44E-07
FRY	1529.70968	2.52688132	1.39E-09	3.62E-07
SNORA22	80.0833148	-2.1797756	2.14E-09	5.48E-07
NICN1	508.06881	1.36947137	2.24E-09	5.64E-07
AMT	250.531065	1.42799535	2.71E-09	6.72E-07
SPNS3	46.4543637	3.99791181	3.08E-09	7.51E-07
SCPEP1	704.424715	1.4591407	3.15E-09	7.57E-07
GINS3	70.9635736	2.28793464	3.29E-09	7.78E-07
LMCD1-AS1	30.8134375	3.71714442	3.35E-09	7.81E-07
C1orf204	63.6096836	2.25310046	3.41E-09	7.82E-07
LEPREL1	494.967462	3.57498082	3.53E-09	7.85E-07
NDRG4	123.306862	4.08955049	3.53E-09	7.85E-07
TLE3	1368.42866	-1.225536	3.58E-09	7.85E-07
HOPX	595.180428	-4.1787089	4.86E-09	1.05E-06
SNRPA1	188.715106	-1.3402391	6.04E-09	1.29E-06
C1QTNF2	75.8739055	3.51349673	7.36E-09	1.53E-06
LTBP1	5836.11589	2.66221755	7.28E-09	1.53E-06
CEBPB	764.532933	-1.6319916	1.45E-08	2.98E-06
FIBIN	5639.37572	3.43823239	1.48E-08	2.99E-06
C16orf86	70.0729878	2.48296861	1.73E-08	3.44E-06
FBL	809.767675	-1.2638973	1.97E-08	3.89E-06
LOC1019270	82.9616562	3.23750769	2.37E-08	4.60E-06
SPATA2	312.503771	-1.1193868	2.46E-08	4.72E-06
PDE1A	3175.88423	1.74106734	2.94E-08	5.50E-06
SDSL	157.468474	-1.2011107	2.93E-08	5.50E-06
NAV3	495.598348	4.14729383	3.07E-08	5.68E-06
KALRN	1381.1174	2.46532214	3.13E-08	5.73E-06
SNORA81	278.00544	-1.1230406	3.52E-08	6.36E-06
RBMS3	1712.04174	2.90068568	4.16E-08	7.43E-06
LOC1019273	23.3722114	3.65669149	4.57E-08	7.98E-06
RPRM	17.2311231	-4.2703577	4.52E-08	7.98E-06
FAM180B	130.228134	4.14284587	4.73E-08	8.16E-06
VPS26A	956.58959	-1.097936	4.93E-08	8.42E-06
H2AFX	227.186949	-1.3129785	5.11E-08	8.54E-06
RPS16	3869.45914	-1.8144526	5.09E-08	8.54E-06
SLC25A19	145.663531	-1.6623851	5.40E-08	8.92E-06
MTMR9LP	369.020284	2.90607615	6.02E-08	9.73E-06
TPD52L2	2383.91508	-0.8914758	5.99E-08	9.73E-06
LRRC37A6P	139.826718	2.30105121	6.31E-08	1.01E-05
BRSK1	144.145881	2.6541596	6.55E-08	1.02E-05

CNTN4	1554.44583	3.91239369	6.75E-08	1.02E-05
FAM127B	343.008818	-1.1354841	6.55E-08	1.02E-05
FBLN1	6312.00332	4.11687098	6.73E-08	1.02E-05
LRP2	440.386223	-4.2015537	6.74E-08	1.02E-05
PCDHA10	147.119114	3.84910393	6.62E-08	1.02E-05
CMAHP	469.81326	2.19084986	7.21E-08	1.08E-05
SSPO	397.27109	2.02558996	8.27E-08	1.22E-05
TRAF3IP2-AS	213.573488	1.91233649	8.79E-08	1.29E-05
MAB21L1	45.9403337	3.53826929	9.06E-08	1.31E-05
MRPL12	203.470859	-1.3296857	9.37E-08	1.32E-05
PIGV	304.072252	1.21836931	9.29E-08	1.32E-05
PTMS	2401.0335	-1.6174574	9.37E-08	1.32E-05
SNORA12	168.944295	-1.4760489	1.01E-07	1.42E-05
MESDC1	265.782867	-1.7359374	1.03E-07	1.43E-05
FMNL3	3179.53717	1.27751017	1.13E-07	1.55E-05
NUMBL	918.914546	-0.7433102	1.25E-07	1.70E-05
MBOAT7	1007.5	-1.2932154	1.30E-07	1.75E-05
NCKAP5	272.86085	2.16296591	1.33E-07	1.78E-05
FNDC4	43.4618769	2.53840513	1.35E-07	1.79E-05
RAB7A	3068.07544	-0.8888403	1.45E-07	1.91E-05
SRBD1	712.238016	1.0032527	1.47E-07	1.91E-05
MYO10	1703.4847	1.68710042	1.52E-07	1.97E-05
ZNF812	254.578988	2.85954176	1.66E-07	2.13E-05
LOC1001309	135.227433	2.95920106	1.77E-07	2.25E-05
KRT17	290.938555	-4.435953	2.37E-07	2.98E-05
PITPNB	298.126276	-1.5016675	2.40E-07	2.99E-05
HSPB1	1784.53833	-1.7723334	2.50E-07	3.09E-05
SMO	974.607944	1.03834039	2.51E-07	3.09E-05
NKD1	2902.84852	3.93439807	2.66E-07	3.25E-05
GFRA1	3920.27359	4.19404843	2.83E-07	3.40E-05
SYTL2	556.291234	2.29415727	2.83E-07	3.40E-05
GHRLOS	87.2710881	2.56877614	2.94E-07	3.50E-05
CLCF1	36.2708432	-2.7978217	3.01E-07	3.55E-05
LHCGR	434.184159	4.18805035	3.09E-07	3.58E-05
PDCL3P4	77.2040671	1.43628226	3.10E-07	3.58E-05
PRKY	24.7014498	-4.060611	3.10E-07	3.58E-05
GADD45GIP1	380.552251	-1.2449738	3.28E-07	3.75E-05
MARVELD1	1180.46317	1.16939191	3.44E-07	3.91E-05
DLEU7-AS1	61.4656224	3.35170418	3.54E-07	3.97E-05
ENTPD6	1974.61124	-1.2376661	3.54E-07	3.97E-05
WBP11	1139.43105	-0.778251	3.56E-07	3.97E-05
VILL	305.646666	1.11760015	3.59E-07	3.97E-05
FKBP14	1042.10996	1.29303287	3.65E-07	4.01E-05
RBPMS2	186.527187	-2.2831013	3.75E-07	4.09E-05
SCAF4	813.497526	-1.0171098	3.89E-07	4.18E-05
USP32P2	330.541727	3.83585615	3.89E-07	4.18E-05
OCRL	1303.94634	-0.7261799	4.32E-07	4.61E-05
ZNF580	440.001675	-1.1463547	4.37E-07	4.63E-05
VASH1	1337.75367	1.64124644	4.46E-07	4.70E-05
RUNX1T1	292.210185	2.9897406	4.85E-07	5.07E-05

CSRNP3	797.32837	3.17505407	4.92E-07	5.11E-05
SLC7A1	367.567272	-2.6774434	5.01E-07	5.16E-05
AXL	2355.51827	1.6597282	6.02E-07	6.16E-05
GRAMD3	3903.63772	0.82565949	6.26E-07	6.37E-05
RPS17	212.834775	-1.6053941	6.42E-07	6.49E-05
RB1	2702.07519	-0.8037136	6.63E-07	6.65E-05
EMC6	134.178027	-1.3238119	6.81E-07	6.71E-05
MMP28	437.408951	2.95091138	6.76E-07	6.71E-05
SEPHS2	472.554228	-1.4458789	6.78E-07	6.71E-05
ARG2	182.620903	-3.0902715	7.00E-07	6.85E-05
ANKRD22	290.495106	3.41402401	7.08E-07	6.88E-05
ISG20	74.463395	-1.8946225	7.35E-07	7.10E-05
DGCR9	33.2272733	3.3524116	7.52E-07	7.22E-05
RPL23AP32	241.485947	2.06140615	8.03E-07	7.67E-05
NAF1	247.329384	-1.1517838	8.65E-07	8.20E-05
ZFP90	1253.66047	1.01022447	9.27E-07	8.74E-05
BAI3	110.729087	3.19465535	9.73E-07	9.12E-05
DNTTIP1	322.258853	-1.1901271	1.08E-06	9.98E-05
GPRC5A	519.279693	-3.6804148	1.08E-06	9.98E-05
UBE2E2	466.182216	1.59103385	1.09E-06	9.98E-05
FTH1P3	49.8120427	-2.4926254	1.12E-06	0.00010264
HEG1	3843.57937	2.51314658	1.15E-06	0.00010374
ITGB2-AS1	123.128026	3.02249708	1.15E-06	0.00010374
LEPREL2	1183.3806	1.34249758	1.15E-06	0.00010374
ANO3	48.4126152	3.73465885	1.18E-06	0.00010568
PDZRN4	129.016036	3.94750504	1.20E-06	0.00010616
AEBP1	10652.7395	3.01144856	1.28E-06	0.00011226
PCDHA11	178.970131	2.91128653	1.28E-06	0.00011226
LOXL3	448.296308	2.74614142	1.34E-06	0.00011687
MGC45800	406.087808	3.95819377	1.39E-06	0.00011992
POTEE	35.0908944	-2.0705575	1.38E-06	0.00011992
KRT10	256.178088	-1.5191397	1.40E-06	0.00012018
CBFA2T2	1645.37138	-0.8953312	1.50E-06	0.00012795
PLAC9	372.926639	3.42661826	1.57E-06	0.00013343
MESTIT1	24.2645232	3.66161296	1.61E-06	0.00013604
OPRL1	82.1149488	2.1013297	1.67E-06	0.0001393
PARP3	306.994546	1.35842503	1.68E-06	0.0001393
UBAP2	901.948535	-1.1599973	1.68E-06	0.0001393
ABHD12	742.993032	-1.0558514	1.72E-06	0.00014216
C16orf47	13.043493	3.76741355	1.78E-06	0.00014618
PCDHA4	90.605321	3.08017522	1.79E-06	0.00014618
CLEC17A	29.8089691	3.38159691	1.83E-06	0.0001477
PCBP2	6997.12212	-1.4061468	1.83E-06	0.0001477
SNAI3	48.0057835	3.08889919	1.84E-06	0.0001477
PA2G4	1625.9407	-0.9009741	1.91E-06	0.00015249
FUS	2061.1438	-1.7157968	1.97E-06	0.00015669
TMEM156	76.0179844	2.93169218	1.98E-06	0.00015702
ADAMTSL2	298.408683	3.00081629	2.02E-06	0.00015869
TRPC1	1133.63864	1.39569733	2.02E-06	0.00015869
FOXC2	610.430689	-1.5446804	2.08E-06	0.00016221

DACT3	124.667194	1.95795319	2.22E-06	0.00017248
CIITA	1213.61787	2.24982852	2.39E-06	0.00018425
BAZ2A	3045.86623	-0.8526061	2.41E-06	0.00018492
CASS4	220.568663	2.07362274	2.43E-06	0.00018581
PHF19	639.152673	1.94523879	2.44E-06	0.00018581
PLCL2	877.095265	2.268075	2.46E-06	0.00018646
DTNA	234.588501	2.14701061	2.51E-06	0.00018872
COL1A1	46547.2989	2.69341312	2.67E-06	0.00020007
DPP10	9.47708917	-3.334248	2.75E-06	0.00020475
DKFZP434A0	15.1704078	3.73504972	2.80E-06	0.00020775
NRG3	89.1857951	-3.5836445	2.85E-06	0.0002105
SLC2A9	160.397648	2.20801179	2.86E-06	0.0002105
BOK	650.332749	2.78141998	2.97E-06	0.00021642
TDRP	1046.95629	-2.4301446	2.96E-06	0.00021642
C3orf18	272.584217	1.58300809	3.05E-06	0.00022116
ATIC	665.29126	-1.7657756	3.07E-06	0.00022146
FAM212A	74.8966926	2.65313236	3.20E-06	0.00022959
OSER1	660.441541	-0.7695652	3.31E-06	0.00023633
SAMD5	1329.41825	3.62360449	3.41E-06	0.00024278
RBM3	3581.23588	-0.9941426	3.47E-06	0.00024589
EDNRA	4030.8456	2.68170083	3.60E-06	0.00025283
PSMF1	1606.69109	-0.8170316	3.60E-06	0.00025283
EBF3	660.617463	3.10302186	3.69E-06	0.00025777
PRRT2	370.648442	2.63465535	3.87E-06	0.00026934
CACNA1G	151.788727	3.40596919	3.93E-06	0.00027071
FLJ46906	25.5518943	2.52309416	3.91E-06	0.00027071
GIMAP1	208.690848	1.85875753	4.00E-06	0.00027404
NLRP3	603.259282	2.16877412	4.04E-06	0.00027571
ATP5D	523.212089	-0.8587376	4.10E-06	0.00027802
FAM129A	3647.17931	-2.2618469	4.11E-06	0.00027802
IGF1	1668.89729	3.17981081	4.45E-06	0.00029977
ATOH8	523.714726	2.12681953	4.64E-06	0.00031123
BEX2	201.750508	-2.4262676	4.71E-06	0.00031431
ABCA2	1147.99246	1.78478817	4.85E-06	0.00032284
POTEJ	27.1459188	-2.0022491	4.92E-06	0.0003258
NAGA	1000.7397	0.73259291	5.00E-06	0.00032981
SLC26A6	636.221343	1.77040758	5.11E-06	0.00033568
LOC728323	66.4908291	-1.5187721	5.17E-06	0.00033797
C5	517.372327	1.88939516	5.27E-06	0.00034302
NUBPL	220.398067	1.34761257	5.48E-06	0.00035512
DAZAP2	3409.97113	-0.861041	5.98E-06	0.00038594
CDC42EP2	441.256379	-1.481863	6.22E-06	0.00039222
DFNB59	54.2502596	2.11114031	6.22E-06	0.00039222
FZD10-AS1	61.3606829	3.24557071	6.28E-06	0.00039222
KCNK10	40.0933572	3.18609796	6.12E-06	0.00039222
PID1	740.484426	2.95686207	6.23E-06	0.00039222
PRPF6	1998.68703	-0.6993764	6.24E-06	0.00039222
SGCA	27.2670295	3.37457889	6.19E-06	0.00039222
TSTD3	115.838688	1.29185602	6.26E-06	0.00039222
PNPLA2	747.766906	-1.2443794	6.43E-06	0.00039969

MRM1	87.9971763	1.28166438	6.53E-06	0.0004046
SRPX	356.122567	3.58751899	6.62E-06	0.00040819
CD24	36.6261091	-3.391969	6.72E-06	0.00041294
ATP6V0C	1099.91462	-1.6060089	6.86E-06	0.00041986
ACY3	18.0914456	3.63999651	7.00E-06	0.00042339
PCBP4	623.170356	1.55806497	6.95E-06	0.00042339
TMSB4Y	12.9252714	-3.8783832	6.99E-06	0.00042339
ANXA2P1	19.3878257	-2.252151	7.06E-06	0.00042537
NCRNA0018	11.8580033	-3.8763932	7.52E-06	0.00045149
CKLF	103.666045	1.59921196	7.80E-06	0.00045904
GPR114	75.7223284	3.06575593	7.79E-06	0.00045904
IREB2	2418.9366	-0.8966751	7.78E-06	0.00045904
RPL13P5	31.5346103	-1.5421908	7.73E-06	0.00045904
SNORA5C	25.3654668	2.49690245	7.79E-06	0.00045904
LOC1001297	22.1781494	2.48031046	7.85E-06	0.0004601
BRICD5	58.5591463	1.49361741	7.91E-06	0.00046187
C9orf139	34.6148275	3.20450578	8.00E-06	0.00046571
COL3A1	45875.1731	2.38635115	8.20E-06	0.00047536
GPSM1	564.736817	1.35297529	8.24E-06	0.00047567
PFN1	3932.01119	-0.9494174	8.82E-06	0.00050751
FAM117B	373.477159	0.99899637	8.92E-06	0.00050957
TRPV3	61.5652676	2.49661446	8.89E-06	0.00050957
SMOC2	2762.60469	2.49215556	9.06E-06	0.00051551
MAP3K3	1925.48476	0.83698734	9.50E-06	0.00053851
LOC646903	62.4677993	2.05883974	9.62E-06	0.00054274
PCSK5	520.277984	2.0240137	9.64E-06	0.00054274
DLGAP2	160.28278	3.44212149	9.74E-06	0.00054634
SYNJ1	1224.35023	-1.003242	9.83E-06	0.00054919
IGSF21	174.006579	2.18615751	9.99E-06	0.00055626
CDH5	2660.78331	1.29149036	1.01E-05	0.00056027
GAPT	167.043429	2.11081383	1.01E-05	0.00056027
ANKRD30BL	18.3395207	3.58879945	1.03E-05	0.00056545
MEX3D	239.941519	-1.2430499	1.07E-05	0.00058814
NT5M	42.3630716	1.91202362	1.10E-05	0.00060111
LOC339874	30.2693535	2.4229137	1.14E-05	0.00062061
GSN-AS1	10.6554725	3.51332889	1.16E-05	0.00062982
RCN3	741.641767	2.42407128	1.16E-05	0.00062982
KIAA0922	819.244876	-2.1760744	1.19E-05	0.00064083
PCOLCE	3790.80254	2.2688758	1.20E-05	0.00064688
USP32P1	218.199474	-3.2031267	1.21E-05	0.00065024
ADAMTS5	725.028482	-3.5477117	1.23E-05	0.00065496
DLL1	213.110559	2.67004839	1.23E-05	0.00065496
BCL3	194.56758	-2.999605	1.25E-05	0.0006613
SFRP4	8547.78065	3.28421819	1.25E-05	0.0006613
CSTB	1368.1141	-2.0482227	1.26E-05	0.00066238
SEMA4B	256.479107	-2.3581979	1.28E-05	0.00067238
MAP2K2	1083.02621	-0.5917673	1.29E-05	0.0006759
COL8A2	3103.57989	2.86190822	1.31E-05	0.00067988
TMEM42	194.662854	1.36274972	1.32E-05	0.00068285
IL27RA	65.2067573	2.12405951	1.35E-05	0.00069832

DUSP14	274.901296	-1.3437833	1.36E-05	0.00069858
TBC1D4	741.704257	1.65740987	1.36E-05	0.00069858
G6PD	924.387591	-1.9814354	1.37E-05	0.00069953
SORL1	7078.16534	2.24696935	1.39E-05	0.00071188
EMP3	751.171591	-1.3150994	1.45E-05	0.00073558
SCNN1D	165.460489	2.05435778	1.45E-05	0.00073558
MLF2	1298.17911	-0.7380505	1.48E-05	0.00075042
ADRM1	830.545069	-0.873068	1.53E-05	0.00077166
SPG7	1665.33845	0.72842755	1.55E-05	0.00077594
AACS	520.19432	-1.0013271	1.58E-05	0.0007878
B3GNT9	931.241058	1.12507271	1.58E-05	0.0007878
LSM14A	2476.81327	-0.6701903	1.60E-05	0.00079669
PSMB10	407.267588	1.01300971	1.63E-05	0.00080551
CLEC10A	83.4503085	3.16148671	1.64E-05	0.00080874
KIAA1614	252.874541	1.85226067	1.66E-05	0.00081977
YPEL2	935.706854	1.712792	1.70E-05	0.00083284
NUDT15	253.213944	-1.264223	1.71E-05	0.00083546
INHBB	243.336524	-2.953258	1.73E-05	0.00084358
C18orf32	55.0688252	-1.335495	1.76E-05	0.00085804
ADCY10P1	178.341844	2.43246047	1.79E-05	0.00086989
KCNA6	238.109519	3.41943777	1.81E-05	0.00087392
EXTL1	33.6215735	3.34612053	1.82E-05	0.00087708
PAK4	737.400849	-1.0965682	1.83E-05	0.00087708
MATN2	1894.35367	3.02135346	1.86E-05	0.00088811
P2RY13	655.801241	2.15706234	1.88E-05	0.00089876
SPATA18	194.331189	3.33239648	1.95E-05	0.00092904
AQP3	23.2898567	-2.7713416	2.01E-05	0.00093142
BTN3A1	674.446782	1.32297456	2.00E-05	0.00093142
CDC42EP3	1375.13036	1.85129749	1.99E-05	0.00093142
CMTR2	777.115538	0.60419959	1.99E-05	0.00093142
NIPSNAP3B	36.7386579	1.91701028	1.98E-05	0.00093142
PPL	496.576815	-3.2722458	1.99E-05	0.00093142
RPL21	13.7266502	2.99050945	2.01E-05	0.00093142
SPACA6P	279.309516	-1.0110515	2.01E-05	0.00093142
TUBA1C	1068.99706	-1.5457826	1.98E-05	0.00093142
SLC12A9	627.61514	1.08891131	2.04E-05	0.0009413
ALDH1A2	789.609623	-3.1726783	2.06E-05	0.00094548
TCEB1	413.386389	-1.1361297	2.06E-05	0.00094548
PTP4A1	3031.03845	-2.1514307	2.08E-05	0.00095203
NAGLU	786.025844	1.45220165	2.20E-05	0.00100239
TIA1	1941.02092	0.82168321	2.23E-05	0.00101489
ATG3	737.363781	-0.6287369	2.27E-05	0.00102919
NUDT3	223.102196	-1.2221234	2.28E-05	0.00102998
ANXA2P3	16.5254199	-2.3804876	2.33E-05	0.00104665
STARD8	824.909303	1.42353066	2.33E-05	0.00104665
SOCS2	354.459172	2.42003089	2.35E-05	0.00105056
POTEF	9.42068188	-2.2710281	2.36E-05	0.00105308
TRAF1	537.449986	1.85331871	2.39E-05	0.00106295
MAPRE1	2519.71499	-0.8688395	2.44E-05	0.00108187
RBP1	657.036329	-2.8077557	2.57E-05	0.00113718

C11orf45	58.7965193	2.33168426	2.58E-05	0.00113869
GPM6B	316.002062	-3.3951571	2.59E-05	0.00114055
PCDHA9	97.6420268	2.97508426	2.64E-05	0.00115605
YTHDF1	1057.07655	-0.7739692	2.64E-05	0.00115605
DDRGK1	782.697803	-0.8577143	2.69E-05	0.0011749
COL9A2	159.561362	2.68009768	2.74E-05	0.00119148
KIF26B	79.1806577	2.41100603	2.75E-05	0.00119148
FTH1	18270.7513	-2.1466717	2.82E-05	0.00121918
NPAS2	192.840746	-1.601458	2.83E-05	0.00122073
SRP14	2906.00717	-1.1516651	2.84E-05	0.00122073
CRMP1	169.741724	1.98897067	2.86E-05	0.00122737
USP2	223.568262	-3.2371521	2.91E-05	0.00124458
PRDX6	3120.95627	-1.0326576	2.99E-05	0.0012763
KLHL24	2248.47398	0.57706976	3.02E-05	0.00128549
TEFM	180.880394	0.73942808	3.03E-05	0.00128549
CAPZB	3022.93529	-1.2129458	3.13E-05	0.00132328
CLEC12A	39.4226214	2.96975808	3.19E-05	0.00134001
MMP2	4992.24454	2.48082863	3.18E-05	0.00134001
UBE2K	1840.93064	-0.6695776	3.19E-05	0.00134001
COL8A1	11001.4523	3.23469543	3.21E-05	0.00134064
GLYCTK	373.828557	0.78701158	3.21E-05	0.00134064
GAP43	242.498126	2.88518245	3.23E-05	0.00134351
LOC151475	26.1165518	3.20587403	3.39E-05	0.00140866
C7orf31	121.392756	1.59623974	3.41E-05	0.00140979
CNNM1	61.302933	-3.2864125	3.41E-05	0.00140979
MRAP	71.0824176	-3.2914385	3.49E-05	0.00143548
SASH1	1673.33707	1.89868226	3.50E-05	0.00143778
MFAP5	4002.13688	3.05250168	3.52E-05	0.00144037
FANCD2	590.55024	1.73347003	3.54E-05	0.00144184
LLPH	329.113395	-1.0513992	3.53E-05	0.00144184
CYS1	490.43288	2.89526334	3.55E-05	0.0014436
TLR10	52.830234	2.40890177	3.58E-05	0.00145313
BAP1	1117.4719	0.64169916	3.61E-05	0.00145819
ENTPD1	1506.12822	1.59817745	3.62E-05	0.00146005
PSRC1	97.3219219	2.26547036	3.73E-05	0.00149875
TRPC3	908.130875	-2.6420277	3.74E-05	0.00150146
STARD5	127.798	2.4475823	3.79E-05	0.00151804
CBX4	491.212535	-1.777197	3.81E-05	0.00152168
CCNYL1	521.550797	-0.8440535	3.86E-05	0.00153459
TBC1D15	1187.68109	-0.687192	3.91E-05	0.00155132
MYOZ1	107.23121	3.10473391	3.94E-05	0.00156029
KAZALD1	69.7993148	3.03092694	4.06E-05	0.00160284
UBE2M	649.500202	-1.4497353	4.11E-05	0.00161721
LIMCH1	2373.99614	1.48879031	4.12E-05	0.00161729
DEFB1	10.9585867	-3.397588	4.15E-05	0.00162615
NRXN2	270.500819	3.0509122	4.27E-05	0.00166768
WNK2	143.449626	-2.7999288	4.28E-05	0.00166768
DAPP1	89.6329615	2.12811774	4.32E-05	0.00167384
MAGI2	846.668953	0.99385094	4.31E-05	0.00167384
NS3BP	136.956891	1.2683581	4.34E-05	0.00167384

ST8SIA1	437.954012	3.40993684	4.34E-05	0.00167384
TNNI3	13.0373529	-3.3938086	4.35E-05	0.00167384
MTRNR2L2	4139.26041	-1.7904975	4.39E-05	0.00168702
FAM98B	546.426768	-1.1986605	4.44E-05	0.0017016
DMD	4379.67576	2.28392498	4.48E-05	0.0017111
ADAM19	666.126675	-2.1890629	4.69E-05	0.00178265
ASS1	3183.29883	-1.623182	4.68E-05	0.00178265
ANXA2P2	88.6323372	-1.8057475	4.86E-05	0.0018428
USP31	1200.2471	-1.6053848	4.90E-05	0.00185487
GZF1	525.226113	-0.9064211	5.09E-05	0.00192234
SERTM1	447.74258	3.38389923	5.12E-05	0.00192779
MIR3648	34.7191799	-3.2346713	5.25E-05	0.00197302
RSPO3	948.435384	3.20343153	5.30E-05	0.00197854
SLC24A3	1267.40416	-2.0781714	5.30E-05	0.00197854
SNORA74B	537.446259	-1.6272242	5.31E-05	0.00197854
FZD4	4203.62883	1.62500253	5.33E-05	0.00198413
NANP	280.173546	-0.8203749	5.35E-05	0.00198525
EDA	633.090013	-2.1647018	5.39E-05	0.00199248
ZCCHC3	399.151733	-1.134191	5.40E-05	0.00199248
LINC01268	62.4960403	2.52579039	5.67E-05	0.00208867
RPL41	4645.37108	-0.9749929	5.81E-05	0.00213602
DUSP5	769.910331	-2.8934907	5.83E-05	0.00213936
DLG2	357.030032	2.4663227	5.96E-05	0.00217027
FILIP1L	612.79453	1.53289318	5.96E-05	0.00217027
TSTA3	325.66258	-1.3037106	5.95E-05	0.00217027
SDHC	899.975198	-0.7690109	6.02E-05	0.00218474
FGF7	758.727417	3.11939453	6.06E-05	0.00219716
ATF5	212.700424	-1.4660871	6.14E-05	0.00221792
PPFIBP2	695.01129	1.96513761	6.15E-05	0.00221792
SNORD89	9.14941746	3.26535094	6.21E-05	0.0022358
F2RL2	132.372557	3.14181284	6.31E-05	0.00224867
OSGEPL1-AS	20.3555783	2.32404662	6.27E-05	0.00224867
PHF14	1607.65242	0.65722013	6.30E-05	0.00224867
RFTN2	481.74834	2.54617919	6.31E-05	0.00224867
DBNDD2	125.097459	-1.5183146	6.39E-05	0.00225275
FAM106A	66.6097201	3.36092123	6.36E-05	0.00225275
UBQLN2	1145.20624	-0.7046826	6.34E-05	0.00225275
WDR59	694.542802	0.75885425	6.37E-05	0.00225275
ZNF579	119.358316	-1.1007855	6.39E-05	0.00225275
DIEXF	634.580466	-0.5917679	6.59E-05	0.0023159
KBTBD12	31.1976024	3.05534822	6.68E-05	0.00234456
CAPNS2	16.99189	2.98308092	6.83E-05	0.00238468
RAB27B	794.268045	-3.3444782	6.83E-05	0.00238468
SNAI2	1627.09641	2.31807195	6.96E-05	0.00242301
SIN3A	1979.37122	-0.8560902	7.16E-05	0.00248694
S100A10	4118.47639	-1.2800556	7.21E-05	0.00249986
PAX6	140.908387	-3.0438182	7.24E-05	0.00250627
LAMB2	16568.7352	1.57367416	7.36E-05	0.00253505
M6PR	1605.82037	-0.8081402	7.36E-05	0.00253505
FAM150B	50.4751388	-3.3503156	7.38E-05	0.00253561

ZNF574	327.811762	-0.7010682	7.57E-05	0.00259489
ZNF69	171.856664	1.77717435	7.58E-05	0.00259489
FAR2	250.399294	1.89328825	7.71E-05	0.00263201
PBDC1	235.763314	-1.1333222	7.73E-05	0.00263201
OLFM2	336.500716	2.18306522	7.75E-05	0.00263278
EBF1	1593.5523	2.76741106	7.76E-05	0.00263293
OXNAD1	256.651065	1.15949784	7.79E-05	0.00263457
CCDC8	726.428547	1.25493425	7.82E-05	0.00263979
IGSF9B	258.839489	-1.6570858	7.85E-05	0.00263979
ITGA8	740.10579	3.10215773	7.85E-05	0.00263979
CLN6	414.055234	-0.7237682	7.92E-05	0.00265546
NHSL1	712.137943	1.5983034	7.94E-05	0.0026566
SHC4	739.491724	3.03563446	8.03E-05	0.00268132
SPON1	198.863673	2.87253632	8.26E-05	0.00275345
ANGPT1	266.416772	3.07801251	8.36E-05	0.00276139
GHRL	31.0738945	2.76585734	8.34E-05	0.00276139
PCDHA2	74.0204063	2.57374564	8.35E-05	0.00276139
ZNF865	250.043489	-1.2378553	8.34E-05	0.00276139
FURIN	2008.87231	-1.1196707	8.42E-05	0.00277616
PDPR	2046.37728	1.09939628	8.47E-05	0.00278592
ELMO1	960.182521	1.52953308	8.57E-05	0.00281017
RASSF3	838.787003	-1.4899065	8.58E-05	0.00281017
RGS10	338.520262	1.30762215	8.66E-05	0.00283048
NBN	4847.62829	2.31698943	8.84E-05	0.00288359
CSNK1D	2134.88768	-1.1492728	8.99E-05	0.00292458
SH3GL1P2	40.4998232	3.2356626	9.01E-05	0.00292644
HIST2H2BE	778.802348	-1.2223305	9.13E-05	0.00294786
PALLD	1010.69946	1.90126087	9.10E-05	0.00294786
VIPR1	122.203871	2.11114819	9.13E-05	0.00294786
ITPR1	1340.12641	1.84771374	9.22E-05	0.00297001
CTSA	2238.74009	-1.0317164	9.29E-05	0.00298472
HMCN1	1724.38962	2.99386011	9.31E-05	0.00298472
CARD9	131.056439	2.13827693	9.52E-05	0.00304855
IFT172	979.135422	1.02054312	9.62E-05	0.00307164
GRIA3	246.633827	3.12196612	9.67E-05	0.00307181
STAT6	4315.64981	-0.9160598	9.68E-05	0.00307181
TXN2	550.627764	-1.2578014	9.64E-05	0.00307181
RPL8	9046.97006	-1.2647836	9.71E-05	0.00307616
TBCE	322.839801	-0.843671	9.73E-05	0.00307635
TRIM65	417.251376	1.14782918	9.76E-05	0.00308047
SUSD3	97.6412492	2.47177495	9.82E-05	0.00309023
CALD1	9633.32965	0.97270148	9.87E-05	0.00310043
RPL17	2019.42801	-0.9426219	9.90E-05	0.00310419
PCBP1	2046.28105	-1.1999325	0.00010019	0.00313477
POLR2L	1037.31889	-1.005457	0.00010105	0.00315531
THAP11	426.758093	1.04467579	0.0001017	0.00315718
ULK1	983.130223	-0.9594494	0.00010173	0.00315718
WNT5A	903.497256	2.4963588	0.00010159	0.00315718
TRMT6	266.778985	-1.1111457	0.0001022	0.00316537
SCN11A	34.2413305	2.37601962	0.00010397	0.003214

FMR1	1526.1203	-0.7146428	0.00010685	0.00328296
SNORA76C	21.6572218	2.59623148	0.00010664	0.00328296
TMEM119	590.075282	2.62213666	0.00010647	0.00328296
RUNX2	611.338329	2.54927378	0.00010855	0.00332877
C21orf91	588.921358	-1.2390598	0.00010916	0.00334057
CSNK2A1	1904.49164	-0.9438932	0.00011034	0.00337012
ATP8B4	759.589314	1.89229228	0.00011075	0.00337435
TMEM220	64.7612617	1.61083228	0.00011092	0.00337435
SGCE	180.052518	2.4138667	0.00011127	0.00337843
BBS2	1417.22308	0.95698129	0.00011164	0.00338283
ACSM5	96.8522889	1.62923034	0.00011192	0.00338458
FZD9	11.9616586	2.99122388	0.00011218	0.00338578
TNS3	5708.79724	2.28967029	0.00011324	0.00341132
ERO1L	1364.69445	-1.3682058	0.00011378	0.003418
HIRA	706.395745	-0.9305524	0.00011391	0.003418
FKBP1A	1647.33284	-1.166668	0.00011486	0.0034399
FAM177B	22.9565675	2.18234364	0.0001173	0.00350471
LOC401052	14.6308791	2.47658232	0.00011749	0.00350471
CACNA1C	1029.5167	1.50197108	0.00011796	0.00351201
GYS1	1236.97573	-1.0228968	0.0001182	0.00351237
ARRDC4	1254.20765	-2.606078	0.00011981	0.00355325
ARAF	1096.38219	-0.6147988	0.00012265	0.00360965
ARL4C	432.738622	-2.5974308	0.0001224	0.00360965
EPHA4	909.997937	2.24844686	0.00012257	0.00360965
MASP1	125.217544	2.98178082	0.00012261	0.00360965
LIMS1	1466.99041	-0.6670824	0.00012337	0.00362399
ATP6V0E2-A	84.0713461	1.29672704	0.00012404	0.00363675
MROH8	115.927199	1.14586564	0.00012669	0.00370016
TMEM115	867.579742	0.72141179	0.00012664	0.00370016
RSRC2	1330.23928	-0.5824476	0.00012712	0.0037059
PPTC7	408.387839	-1.0221237	0.00013059	0.0037996
MAN1B1-AS	27.2382383	1.79384685	0.00013236	0.00384395
DDAH1	1396.00879	1.48702171	0.00013431	0.00388583
SEC11C	573.009719	-1.6435295	0.00013427	0.00388583
CFL2	1222.73687	-0.8106719	0.00013531	0.00390027
IPCEF1	196.584247	2.01322287	0.00013528	0.00390027
FSIP2	356.077078	3.09760331	0.00013639	0.0039239
ACKR3	1345.37309	-2.3466173	0.00013812	0.00396612
MCOLN1	346.372455	-1.0336194	0.0001398	0.00400694
MYH10	4064.02102	1.78242206	0.00014093	0.00403174
PCBP1-AS1	146.694438	1.67278117	0.00014188	0.00405157
AOAH-IT1	9.67468382	3.14085245	0.00014414	0.00410834
FAM131A	411.365615	1.62348188	0.00014468	0.00411628
SNORA74A	149.935201	-1.3867119	0.00014522	0.0041238
PALM2	63.1272044	2.76052578	0.0001456	0.00412699
DOK5	333.78819	2.75872387	0.00014806	0.00418896
EIF1	4640.81777	-1.1773505	0.00014966	0.00422656
UBA7	1315.67449	1.46550037	0.00015139	0.00426758
IL17RC	469.207089	1.06048484	0.00015205	0.00427841
PRKAR2A-AS	20.1933391	2.14972069	0.00015282	0.00429206

DLGAP1-AS1	150.996104	-1.0612229	0.00015453	0.0043322
TLR5	278.398309	1.63664517	0.00015493	0.00433542
GSK3A	647.343512	-0.9753142	0.00015587	0.00435378
ERH	874.33499	-0.9769701	0.00015741	0.00438886
NAALAD2	108.544845	2.87686735	0.00015779	0.00439123
PCDHA7	45.9968662	2.78946951	0.00015806	0.00439123
DCTPP1	194.707599	-0.675629	0.00015883	0.00439309
GAR1	98.4514306	-1.0869057	0.00015899	0.00439309
RASAL2-AS1	66.9377764	1.63185875	0.00015892	0.00439309
QDPR	235.751868	-1.1157772	0.00016461	0.00454019
RNF207	292.082755	2.05575308	0.00016663	0.00458768
SESN1	3322.38454	1.33310648	0.00016723	0.00459611
ENO2	1061.55565	-2.353907	0.0001677	0.00460057
PLD2	1008.35744	1.08372472	0.00016944	0.00464002
LOC1019280	8.67536188	2.85355949	0.00017022	0.00465306
CWC15	724.153853	-0.5933635	0.00017102	0.00465839
ENGASE	626.486568	1.0020621	0.00017094	0.00465839
LINC00312	54.5382203	2.74119969	0.00017435	0.00474086
SMYD5	311.563773	0.75687797	0.00017541	0.00475291
TBC1D5	2406.00603	1.01108105	0.00017515	0.00475291
LOC284950	19.3966837	2.22239665	0.00017598	0.00475463
MEF2C	2651.62251	1.39697619	0.0001761	0.00475463
NLRP1	731.910167	1.73847958	0.00017847	0.00481015
CNTN2	10.7852227	3.11241158	0.0001792	0.00482141
CCDC88B	523.941742	1.97466119	0.00018049	0.00484587
GGTA1P	451.984114	1.77270456	0.00018074	0.00484587
MPND	170.852401	-1.1691757	0.00018133	0.00485314
LOC283922	556.880925	0.86035315	0.0001828	0.00488422
NOP10	500.807821	-0.9863494	0.00018394	0.00490607
EMILIN1	1893.80821	1.7897165	0.00018619	0.00493763
GAB3	331.116839	1.48265017	0.00018612	0.00493763
THRB	109.527029	2.73780905	0.00018641	0.00493763
UBAP1	1375.42846	-0.872579	0.00018546	0.00493763
TMEM218	259.375198	0.73656115	0.00018679	0.00493917
KIAA1522	1135.1823	-1.9172124	0.00018849	0.00497517
MAP10	98.4568366	-1.4713819	0.00018896	0.00497517
MYH3	86.0329407	1.61745194	0.00018912	0.00497517
GLP1R	43.7250765	-3.0331997	0.00018998	0.00498922
PIGZ	169.348199	1.49184313	0.00019186	0.00502984
GSTM2	455.514939	1.61589855	0.00019268	0.00504291
NSUN5P1	589.530764	1.62388404	0.00019399	0.00506033
SNORA20	19.4358215	2.50229536	0.00019401	0.00506033
PYROXD1	513.131255	-1.3291566	0.00019435	0.00506078
G0S2	72.2470203	-2.8415676	0.00019582	0.00509037
CLPTM1	1677.44311	-0.6832993	0.00019789	0.00512865
FIZ1	147.828598	-1.1781114	0.00019796	0.00512865
DNASE1L3	868.874127	2.68518059	0.00019952	0.00515166
SHISA6	361.395124	2.59157004	0.00019947	0.00515166
MXD4	1987.32566	0.8671411	0.00020034	0.00516407
HPRT1	268.798617	-1.4893937	0.00020254	0.00521204

LOC1019270	252.904538	1.20131579	0.00020802	0.00533965
MCM5	471.793998	0.72225386	0.00020819	0.00533965
SOX9	23.2504587	-2.9220984	0.0002112	0.00540772
RABIF	263.922879	-1.1489607	0.00021233	0.00542768
MED4	785.10841	-0.5709804	0.00021303	0.00543642
CALCR	18.8226921	2.45916663	0.00021457	0.00546667
C20orf197	36.5847515	2.23582047	0.00021577	0.00547917
DHCR24	1925.64843	-2.5705388	0.00021556	0.00547917
FUT4	326.038951	1.02115279	0.00021932	0.00555955
ZNF444	188.642946	-0.6780862	0.00021966	0.00555955
EMC3-AS1	133.059711	1.89979701	0.0002202	0.00556399
IL17RB	61.4500191	2.42490092	0.00022222	0.00560136
RAC1	4455.22706	-0.5771551	0.00022241	0.00560136
CD244	15.4490828	2.6568497	0.00022537	0.00564628
HNRNPM	2038.38977	-0.7024271	0.00022556	0.00564628
NACC1	1179.95139	-1.1121407	0.00022566	0.00564628
TAL1	272.79669	2.47126209	0.0002251	0.00564628
GLT8D1	1011.94515	0.87816263	0.00022645	0.00565686
POLR2M	114.577832	-0.9627565	0.00022897	0.00571048
CCDC121	89.1345341	1.17868351	0.00022975	0.00572055
AGPAT4	529.12144	1.08075165	0.00023217	0.00577152
FMO1	1344.16604	2.804712	0.00023573	0.00584451
TXLNG	831.846009	-1.1526509	0.00023586	0.00584451
RPLP1	10798.7556	-1.2888054	0.0002414	0.00597206
MT1M	31.2152843	2.73969367	0.0002441	0.00602918
SHMT2	1060.13508	-1.2224278	0.00025051	0.00617739
ST5	1220.11172	0.73194388	0.00025169	0.00619659
ZNF628	107.03335	-1.2568553	0.00025359	0.00623356
CIB1	511.522479	-1.2605273	0.00025947	0.00636775
CCDC102A	255.324377	1.30030086	0.00026044	0.00637249
COX5A	429.782307	-1.274571	0.00026049	0.00637249
ERCC1	1473.70876	0.77806067	0.0002615	0.00638691
CCDC92	673.497711	-1.1669481	0.00026243	0.00639943
HNRNPA0	2304.32732	-0.8173607	0.00026572	0.00646946
ACD	187.789044	0.96015521	0.00026828	0.00651118
TMEM229B	63.2061408	1.43126959	0.00026827	0.00651118
NFYC-AS1	114.489439	1.28723759	0.00026901	0.00651854
ZNF331	1140.87662	-1.5215835	0.0002718	0.00657574
TBXA2R	40.5754317	2.37653074	0.00027959	0.00675362
NLGN3	400.216541	2.59191746	0.00028022	0.00675818
HOXC6	37.3483962	3.03567396	0.00028139	0.00677575
DYNLRB1	1326.49131	-1.1120844	0.00028848	0.00693563
PCBP2-OT1	55.9247458	-1.2388719	0.00028926	0.00694365
SNAP91	164.207432	2.73410439	0.00029382	0.00704208
KLHL5	1377.96127	1.18275094	0.00029609	0.00708538
ZNF213-AS1	59.0027929	1.17928156	0.00030172	0.00720883
CCDC142	337.560478	1.04723596	0.00030219	0.00720895
LINC00899	42.3066193	1.33401247	0.00030283	0.00721076
S100A14	14.9285169	-2.9356745	0.00030321	0.00721076
KLHDC4	217.833784	0.83869665	0.00030653	0.00727862

LOC1005070(	44.0152643	1.80713481	0.00031212	0.00739993
FAT1	11804.1656	1.36601671	0.00031619	0.00748479
EIF3J	874.613396	-1.0149766	0.00031732	0.00749996
ASPRV1	90.8056375	1.87331195	0.00031985	0.00753648
TSPAN32	15.1631121	2.77056904	0.00031937	0.00753648
INMT-FAM1ℓ	18.272538	3.03176381	0.0003247	0.00762952
OLFML3	2837.40513	1.83724698	0.00032528	0.00762952
YWHAB	4771.75474	-0.7788141	0.00032528	0.00762952
RBM42	743.473457	-0.6790696	0.00032627	0.00764093
ECH1	800.723944	-0.8660693	0.00032758	0.00765518
NFIL3	559.549207	-2.2427688	0.00032787	0.00765518
DNAH12	112.597017	2.08651825	0.00032957	0.00768316
RAB8A	1343.44876	-0.7279304	0.00033341	0.00776088
AHSA2	2151.95459	1.20593454	0.00033401	0.00776317
SELV	11.0631401	3.00296395	0.00033699	0.0078204
LINGO1	89.1327654	1.71982627	0.00033946	0.00786603
LGALS3	2758.87908	-2.3275068	0.00034103	0.0078718
LRRN2	104.340232	2.78930774	0.00034125	0.0078718
MYO15B	3435.33817	1.58946885	0.00034079	0.0078718
PDCD4-AS1	37.1261607	1.82495455	0.00034379	0.0079185
ATP5J	901.858027	-1.1357713	0.00034454	0.007924
NT5C1A	39.783805	2.9038844	0.00034551	0.00793423
HTR2C	11.4203673	2.99991886	0.00034619	0.00793685
TOB1	2285.526	-1.4578427	0.00034665	0.00793685
LOC284837	24.2106835	2.79231737	0.00034795	0.00795471
AKAP13	15762.1019	-0.6351438	0.00035091	0.00795652
IGFBPL1	18.272326	-2.8051793	0.00034913	0.00795652
ITIH3	35.3523426	2.82417508	0.00035133	0.00795652
NCALD	1336.70974	-2.7558345	0.00035166	0.00795652
PRKCZ	99.5698894	-2.5114033	0.00034929	0.00795652
TMOD1	5538.83732	-1.554256	0.00035124	0.00795652
TRAPPC5	334.362878	-0.8510535	0.00035144	0.00795652
ANO9	54.8499357	-2.6480902	0.00035402	0.00795964
DTNBP1	178.488673	-0.9864221	0.00035422	0.00795964
RBM45	232.651083	0.7494583	0.00035232	0.00795964
SLC12A2	9822.45764	-1.6249572	0.00035289	0.00795964
SLC7A6	1317.94558	1.14897995	0.00035438	0.00795964
PLCB2	1144.98014	1.75220463	0.00035615	0.00798765
PLAG1	78.8652914	1.99631096	0.00035701	0.00799517
CHMP2A	1131.31038	-1.0542	0.00035928	0.00803438
GTF2IRD2	149.040519	0.99658929	0.0003603	0.00804556
SCG5	264.329547	-2.838892	0.00036503	0.00813923
LUCAT1	9.22816383	-3.0262714	0.0003696	0.0082012
NKAP	387.141917	-1.0354165	0.00036928	0.0082012
PTTG2	10.102701	2.56863028	0.00036994	0.0082012
SNRPF	292.586193	-1.3201248	0.0003695	0.0082012
LINC00342	744.689092	0.8764764	0.00037793	0.00835686
PLA2G7	33.4020891	-2.6415058	0.00037805	0.00835686
WSCD2	54.7271143	2.86735748	0.00038224	0.0084373
ENO3	63.650615	2.04642941	0.00038394	0.00845049

HLA-DRB5	1552.58546	2.80126178	0.00038378	0.00845049
SNORA48	966.393958	1.53860525	0.00038999	0.00857148
CDK18	202.738754	-1.3351657	0.00039127	0.00858734
LOC1002893	14.3711941	2.22730826	0.0003928	0.00860849
LOC729603	311.237427	-1.0936389	0.000394	0.00862261
DENND4A	2411.69674	-0.9291077	0.00039754	0.00868742
ZKSCAN7	207.292828	1.00328304	0.0003981	0.00868742
ANKRD36	2110.02394	0.79689211	0.00040108	0.00874016
TUBB4B	2459.56947	-1.4930266	0.00040417	0.008795
TRMT61B	228.02174	0.80183387	0.00040846	0.00887582
TOP1MT	211.848651	-2.0004343	0.0004109	0.00891628
RNF166	465.053427	1.21736561	0.00041509	0.00898175
SGPP2	248.851816	-2.8905812	0.00041457	0.00898175
ACCS	410.051684	1.48826195	0.00042002	0.00907557
CYB561A3	882.024446	1.19620023	0.00042534	0.00917764
CAPNS1	4690.65513	-0.8133051	0.00042614	0.009182
DLGAP4	1309.42927	-1.1024314	0.00043325	0.00930908
TRIM29	457.270295	-2.7948442	0.00043272	0.00930908
PI3	11.6680324	-3.0416101	0.00043952	0.00943056
LMO3	60.8938863	2.91116516	0.00044244	0.00945998
MMRN1	354.25081	-2.3335649	0.00044273	0.00945998
ZFR2	13.6986409	2.96213228	0.00044192	0.00945998
PCGF6	158.800389	0.78756202	0.00044374	0.00946838
ADRA2A	13.2014956	2.66873848	0.00044747	0.00952153
SRXN1	777.431352	-1.1253822	0.00044736	0.00952153
ZNF664	2776.82718	-1.1288352	0.00044812	0.00952207
RALY	2036.47482	-0.9270183	0.00044878	0.00952284
MB21D2	139.219315	1.54378255	0.0004497	0.00952935
MYO1A	44.7143486	-2.3065836	0.00045182	0.009561
SLC2A12	944.894999	2.0665868	0.00045408	0.00958255
SPTSSA	421.142109	-0.9841961	0.00045394	0.00958255
SLC38A10	2836.35803	0.99946174	0.00045603	0.00961035
POLR2I	250.763643	-1.0172116	0.00045979	0.00967648
PAICS	1166.22355	-0.9719581	0.00046195	0.00970862
CNTN6	55.0477926	2.5997682	0.00046296	0.00971643
FAM91A1	5322.97057	-2.0509746	0.00046613	0.00976959
CX3CL1	462.72946	1.96911194	0.0004706	0.00984983
ADCY7	2730.03658	1.24281606	0.0004714	0.00985332
PMP22	2339.5485	1.43133474	0.00047448	0.00990409
SLC2A14	45.6463543	-2.8330244	0.00047685	0.00994011
PROM1	95.1881815	2.94861032	0.00048173	0.0100282
SNORD15A	10.8161993	2.35692686	0.00049055	0.01019796
BRINP1	68.7483317	2.88252422	0.00049171	0.01020828
ARRDC5	34.302494	2.31017883	0.00049369	0.01022173
NXT1	156.005371	-0.9029805	0.00049314	0.01022173
C21orf58	87.7394743	1.22654822	0.00049608	0.01024358
CHI3L2	106.860338	-2.7879451	0.00049591	0.01024358
SKAP2	891.470399	1.08767889	0.00050279	0.01036827
PLP2	664.618069	-1.3253636	0.00050452	0.01039005
ADAP1	130.556636	1.8302063	0.00051094	0.01050813

SH3TC1	1048.86542	1.66061068	0.00051273	0.01053086
LINC00857	28.2462493	2.20758411	0.00051406	0.01054396
LOC283335	141.036405	-1.0145273	0.00051895	0.01063014
GGNBP2	1482.79294	-0.611821	0.00052191	0.01066228
PCDH11Y	8.25868798	-3.0009378	0.0005216	0.01066228
GPR64	153.234847	-2.5472065	0.00052681	0.01074828
GPR126	613.497559	1.76380886	0.00053079	0.01081502
SEC31B	741.009322	0.96308053	0.00053339	0.01085355
SRC	710.208087	-0.9228226	0.00053553	0.0108827
NBPF8	305.11098	1.4250019	0.00053851	0.0109288
ANKRD1	10.5070028	-2.975374	0.00053991	0.01094273
FAM26E	659.546449	1.55320548	0.00054216	0.01094581
MORF4L2	3825.56092	-0.8569618	0.0005411	0.01094581
PITPNM3	265.023476	-1.8151033	0.0005422	0.01094581
CH25H	115.36178	2.2914093	0.00054446	0.01097633
RFX5	1026.69753	0.92998575	0.00054514	0.01097633
P2RY12	468.826547	2.11930653	0.0005465	0.01098938
ANKRD65	30.1232521	2.64911908	0.00054997	0.01104469
COL5A2	7569.29394	1.10113393	0.00055228	0.01107657
MED16	869.328762	-0.7420391	0.00055939	0.01118997
ZNF468	289.165284	-0.7393391	0.00055931	0.01118997
LARS2	486.762594	0.74902749	0.00056066	0.0112009
ATE1-AS1	31.9303135	2.18710526	0.0005656	0.01123571
EML1	523.020245	1.31777436	0.00056447	0.01123571
LOC554206	20.1427993	1.75056002	0.00056536	0.01123571
RHOV	18.7618804	-2.8783546	0.00056606	0.01123571
TAB3	1068.96705	-0.917986	0.00056606	0.01123571
COL4A6	67.7702959	2.47816879	0.00057152	0.01128551
L1CAM	8.97877941	-2.2330204	0.00057225	0.01128551
MAP3K7CL	133.072131	-1.449747	0.00057122	0.01128551
SLC29A1	2944.39416	1.77596797	0.00057032	0.01128551
TNN	62.1486847	2.55357225	0.00057165	0.01128551
LOC283194	20.0791799	1.99008175	0.00057311	0.01128807
APEX2	252.523917	-0.6339777	0.00057434	0.01129786
MRPL4	465.151375	-0.6475811	0.00057521	0.01130046
PLEKHH2	3748.01927	2.28258298	0.00058106	0.01140089
RAB21	1339.04178	-0.7948574	0.00058438	0.01143676
TM6SF1	344.173426	1.49557378	0.000584	0.01143676
HEXIM1	798.856244	-1.1706063	0.00059058	0.01152878
LOC1005065	70.1411182	2.13599161	0.00059044	0.01152878
ZNF708	1273.53564	-0.9973838	0.00059136	0.01152928
CLEC9A	167.584276	2.38036357	0.00059286	0.01154378
KCNJ3	36.6410769	2.89300108	0.00059625	0.01159509
CDC16	1293.64053	-0.8800938	0.00059937	0.01163289
LARP1B	917.856775	-1.5204984	0.0005997	0.01163289
KIF27	288.775451	0.90224899	0.00060106	0.0116444
C21orf15	486.85702	2.91223024	0.00060212	0.01165041
PRMT7	498.536886	0.86156564	0.00060445	0.01166611
USP42	434.393791	-0.6576492	0.00060388	0.01166611
ADSS	793.510239	-0.7198737	0.00061178	0.01179278

CD55	1995.7422	-1.8627513	0.00061559	0.01181741
CRTAM	20.0103468	2.32244284	0.00061409	0.01181741
FAM26F	89.4034197	1.73619366	0.00061574	0.01181741
NAA20	650.607461	-1.0322253	0.00061614	0.01181741
DYNLL1	1700.1442	-0.9863603	0.00062272	0.01191607
HS3ST3B1	103.954537	2.34919171	0.00062283	0.01191607
VTN	381.788056	-2.1751177	0.00062438	0.01193085
LYNX1	967.467142	1.41154922	0.00062588	0.01194467
ALDH1L1	132.396819	2.47489375	0.00062722	0.01195525
LOC1019296	34.5724581	2.78968797	0.00062987	0.011991
ITGAM	1337.93296	1.91490502	0.00063731	0.01211764
PYCR1	512.518961	1.86588997	0.00063997	0.01215304
FEM1A	819.666195	-0.7783693	0.00064142	0.01216556
CNKSR3	722.684385	1.32284133	0.00064808	0.01224658
CTXN1	41.4439617	-1.5952942	0.0006468	0.01224658
FGF20	9.08365234	2.68765234	0.00064763	0.01224658
MAP3K9	183.155383	-1.376877	0.00064926	0.01225372
MIR22HG	748.438678	-0.9396079	0.00065163	0.01228351
DTWD1	489.633382	-0.5816683	0.00065337	0.01228723
TANGO6	587.773382	0.94970647	0.00065343	0.01228723
UBQLN1	2394.5701	-0.8037117	0.00065548	0.01231066
SIRT5	372.196089	0.74999464	0.0006606	0.01239177
SNRNP200	5828.95751	-0.7243455	0.00066842	0.01252311
MYCBPAP	35.8417566	2.07086762	0.00067088	0.01255382
FBRSL1	568.410013	-1.0722577	0.00067633	0.01262924
OSBP2	67.2768678	-2.3478933	0.00067655	0.01262924
EZH2	134.918189	-1.1593346	0.00067856	0.01265143
LMCD1	503.019432	2.4211768	0.00067992	0.01266127
ACAN	113.228601	2.67576287	0.00070387	0.01304727
CD37	250.986186	1.76639296	0.00070539	0.01304727
HCCS	276.042035	-1.1963855	0.00070307	0.01304727
SAFB2	978.032607	-0.6653934	0.00070282	0.01304727
SCAND1	295.476764	-0.8983478	0.00070647	0.01304727
TNFAIP8L2	104.010358	1.82210148	0.00070659	0.01304727
YWHAE	6446.54376	-0.7054838	0.00070409	0.01304727
PGK1	5621.27847	-1.3809556	0.00072342	0.01334195
NPY1R	667.073166	-2.7639848	0.00073423	0.01352511
LOC1005064	17.0189156	2.79063123	0.00073586	0.01353889
B4GALT5	1583.03869	-1.1518194	0.00074361	0.01365152
PLAGL2	436.443136	-0.9955791	0.00074376	0.01365152
PTPN1	1253.72041	-1.0971926	0.00074757	0.01370519
CHAC1	11.961101	-2.0246988	0.00075707	0.01386283
PROSER1	583.99514	-0.9022396	0.00075838	0.01387014
LINC01197	90.6975195	2.8297986	0.00076661	0.0140041
MAPK6	1080.27006	-1.0645586	0.00077333	0.01411011
PCDHGA12	1120.5838	1.82584859	0.00077644	0.01411921
PRPS1	529.18021	-0.8550471	0.00077613	0.01411921
UBE2I	1087.45472	-0.4297189	0.00077659	0.01411921
KCND3	64.3494095	-1.3916829	0.00077954	0.01415616
TPP1	2886.47553	-0.9934917	0.00078161	0.01417686

RITA1	305.516166	-1.0321989	0.00078435	0.01420989
SRGAP1	1114.17869	-1.3567124	0.00078914	0.01427985
ZNF335	779.18713	-0.732305	0.00079078	0.01429261
BST1	328.632774	2.01882266	0.00079602	0.01433698
ITM2A	4918.93021	1.83461894	0.00079603	0.01433698
SPARCL1	13563.394	1.03601134	0.000796	0.01433698
ZNF28	437.737883	-0.7719668	0.00079804	0.01435626
RPS28	2398.57962	-0.8680456	0.00080585	0.01447987
CNKSR2	53.9463656	2.58935404	0.00081386	0.01460661
TNFRSF25	111.059158	1.66190379	0.00081578	0.0146241
NEDD9	1028.66802	1.35040323	0.00081907	0.01466594
ALMS1-IT1	21.0807911	1.91519363	0.00082503	0.01473822
CD74	27903.7822	1.55090344	0.00082474	0.01473822
FAM105A	2101.89535	1.54557377	0.0008261	0.01474024
LARP4	1938.08526	-0.7317741	0.00083383	0.0148265
P2RX1	17.5707629	2.09152965	0.00083361	0.0148265
SDC4	981.563555	-1.6015522	0.00083374	0.0148265
CELSR1	617.222911	-2.5172049	0.0008382	0.01488704
GPX7	209.97616	1.59682979	0.0008465	0.01501707
PEX6	677.137069	0.78134545	0.00086083	0.01525373
PLXDC2	6200.63632	1.5055388	0.00086349	0.01528257
TRIM6	260.984242	-1.2166999	0.00086445	0.01528257
CHN1	1133.14152	-1.3703171	0.00086916	0.01532277
PLAA	925.472586	-0.826155	0.00086907	0.01532277
PPP2R2C	49.3000361	-2.3890317	0.00086972	0.01532277
SPTA1	8.30054004	2.39940616	0.0008709	0.01532604
STAU1	2588.1545	-0.6101138	0.00087829	0.01543837
LOC1019272	34.4676324	2.81142626	0.00088224	0.01549016
TACC2	912.585772	-0.8984607	0.00088664	0.01554965
VENTX	295.629809	2.15843492	0.00088919	0.01557646
SCARA3	4138.13101	-0.8704819	0.00089081	0.01558719
PSMG1	316.84735	-0.9772474	0.00089387	0.01562293
MKS1	187.278592	0.77831039	0.00089601	0.01564256
PLXNA1	2568.02638	1.21292263	0.0008982	0.01566288
COG4	1092.30005	0.81853927	0.00090017	0.01567948
ZNF843	18.1188645	2.2196551	0.000907	0.0157805
HSP90AA1	10270.4635	-1.1153391	0.00090804	0.01578082
HSD11B1	56.2220665	2.70056952	0.00091026	0.01580157
ABHD17C	328.589086	-1.0532266	0.00092504	0.0160255
CYP4X1	1892.73979	1.6403022	0.00092653	0.0160255
IL11RA	853.990418	2.05444802	0.00092783	0.0160255
LINC01125	41.7246802	1.61641355	0.00092838	0.0160255
RBP4	296.653008	2.5277803	0.00092836	0.0160255
C8orf31	49.3788267	2.57158589	0.00093224	0.01607416
EMILIN2	944.124508	-2.5142302	0.00094157	0.0162167
FARSA	725.191125	-0.6823301	0.00094331	0.01622859
HSPG2	8969.25753	1.63771893	0.00094513	0.01624171
XAF1	1130.46438	1.00351588	0.00094711	0.01625753
CDK16	1244.51021	-0.6020316	0.00095407	0.01633035
MBTPS1	3447.43838	0.74203704	0.00095451	0.01633035

VGLL3	259.533578	-2.7300103	0.00095454	0.01633035
CDC14B	900.759176	1.43263615	0.00095825	0.0163573
PARD6B	149.72241	-1.9444067	0.00095791	0.0163573
BDH2	990.976214	0.82211549	0.000964	0.01641898
ZCCHC18	14.6955234	2.1870727	0.00096358	0.01641898
ICK	1191.90816	1.04525811	0.00097428	0.01657573
STAG3	38.5199782	1.75721961	0.00097573	0.016582
DENND6A	1330.55839	0.69142091	0.00098283	0.01667242
FAM72B	21.9865099	1.94051798	0.00098322	0.01667242
CLEC4F	7.93549843	2.58998164	0.00098882	0.01674114
YWHAQ	3649.59078	-0.6425057	0.00098945	0.01674114
SYT17	49.7533372	1.86444042	0.00099202	0.01676615
ENTPD3-AS1	41.7059566	1.45964853	0.00099315	0.01676675
IFNAR2	462.576349	-1.1813822	0.0009959	0.016791
ZPR1	493.415857	-0.791873	0.00099677	0.016791
PYGB	3199.95586	-0.7667665	0.0010055	0.0169195
INO80C	139.883804	-1.708679	0.0010067	0.01692116
ATP5G2	1876.55627	-0.6604709	0.00100866	0.01693556
PPME1	806.349812	-0.9126827	0.00100989	0.01693774
MYLK4	944.623857	2.32136327	0.00101368	0.0169828
CALM3	4527.60911	-0.9044738	0.00101805	0.01701197
CEP97	378.816246	0.61351346	0.00101718	0.01701197
NOTCH2	11985.7594	0.76224265	0.00101875	0.01701197
OR7E2P	40.6007925	2.55287914	0.00102075	0.01702697
TOB2	1005.11243	-0.6447044	0.00102316	0.01704863
HSPA12A	962.726338	2.1852973	0.00102839	0.01711724
IL15	71.7333825	1.80522795	0.0010296	0.0171188
POLR3H	730.021962	-0.6768681	0.00103125	0.01712765
ELOVL6	243.996538	-1.0141249	0.00103711	0.01720644
ZSCAN31	273.735367	1.67510051	0.00104013	0.0172379
COL6A1	16576.8415	1.16560707	0.00104136	0.01723969
PPP1R7	548.219574	-0.7162144	0.00104326	0.01725255
IRF2BP1	403.649315	-0.63473	0.0010503	0.01735031
MYL4	9.51911381	2.77439521	0.00105383	0.017388
REPS2	1602.69765	-1.8413867	0.00105485	0.017388
C19orf43	1315.33454	-0.8918381	0.00106361	0.01740175
DENND2C	382.57515	1.35017334	0.00106254	0.01740175
MGARP	103.296838	2.76563456	0.00106107	0.01740175
SIK1	3289.53546	-2.1301428	0.00105821	0.01740175
SNORA68	150.177336	1.59359503	0.00106265	0.01740175
ZBTB8B	16.1528708	2.50722832	0.00105985	0.01740175
ZC2HC1C	321.720164	2.14117091	0.00105891	0.01740175
PAF1	1016.13852	-0.9804156	0.00106591	0.01741417
UBE2R2	1325.7681	-0.7063894	0.00106664	0.01741417
CXXC1	659.209284	-0.5992859	0.00106829	0.0174227
FILIP1	274.221638	1.97538108	0.00107555	0.01752245
ARHGEF2	2060.67258	-0.6621327	0.00107777	0.01754002
CES1	80.0772939	-2.7801426	0.00108174	0.01758604
ERG	415.406522	1.18070454	0.00109126	0.01770331
NIPA1	559.243424	-0.9494003	0.00109067	0.01770331

SLC12A5	17.5516425	2.51584765	0.00109414	0.01773137
KCTD20	2717.07642	0.61376702	0.0010978	0.01773462
MALAT1	581262.618	-0.9233516	0.00109781	0.01773462
TMEM160	118.699612	-0.9214542	0.00109582	0.01773462
SEPSECS	501.07572	0.7578284	0.00110484	0.0178295
EIF3M	1470.01782	-0.4618596	0.00112034	0.01802282
LOC146880	945.6103	0.97869019	0.00112019	0.01802282
SOX5	21.2288512	2.30444436	0.00111992	0.01802282
HES4	394.058654	-1.8373378	0.00112473	0.01803122
TUBB6	2304.71152	-1.5526887	0.00112261	0.01803122
UBAP2L	2554.30174	-0.6389463	0.00112556	0.01803122
ZBTB45	151.010001	-0.7715005	0.00112404	0.01803122
MRPS17	112.858545	-0.8544914	0.00112739	0.0180418
KRT80	15.8850111	-2.8175413	0.00113038	0.01807082
ARID2	2930.95575	-0.8974984	0.0011387	0.01816599
CYTH3	1689.58608	0.87198068	0.00113799	0.01816599
SUMF1	913.374125	0.94468517	0.00114049	0.01817568
FOLR1	104.53424	2.43392974	0.00114181	0.01817778
TTTY15	144.674926	-2.7478788	0.00114879	0.01827011
ANKRD36BP2	8.94570356	-2.1411573	0.00116946	0.01855307
FAM207A	115.102646	-1.0279334	0.00116892	0.01855307
PATL2	19.9372947	1.81472094	0.00117021	0.01855307
DCUN1D4	1532.67687	-0.697135	0.00117349	0.01857648
LOC1026064	32.0980222	1.53145872	0.0011741	0.01857648
ATP6V1E2	15.645973	2.07315712	0.00118795	0.01875685
ZKSCAN2	407.084356	0.67747018	0.00118682	0.01875685
DEDD2	401.632871	-0.8293514	0.0011918	0.01875984
PCCA	1578.10228	-0.9728506	0.00119085	0.01875984
PPOX	243.424126	0.91032803	0.00119147	0.01875984
RGS6	217.673851	2.63708066	0.00119888	0.01885191
AHNAK2	6837.3737	-1.961363	0.00120714	0.01894303
NSFL1C	1134.49964	-0.9271764	0.00120702	0.01894303
ZNF789	99.9534675	1.04498206	0.00121579	0.01905938
LOC1009964	24.4573534	2.46513057	0.00122375	0.01916461
KAL1	495.14137	-2.2546643	0.00123377	0.01930179
ZBTB42	76.6851708	-0.9546743	0.00123525	0.01930533
EFNA1	365.936985	-1.6811264	0.00124279	0.01940336
TPST1	340.339045	0.96668341	0.0012488	0.01947752
ZNF770	2102.87023	-0.856476	0.00125089	0.01949032
GABARAPL1	2650.75191	-1.3568197	0.00125657	0.0195589
CCDC59	320.925621	-0.645198	0.00126225	0.01962753
DYRK1A	2813.89754	-0.5270539	0.00126802	0.01969393
PTPN2	824.162323	-0.7596321	0.00126909	0.01969393
SNORA76A	11.1004	1.93413559	0.00127051	0.01969618
HEMK1	753.040145	0.68872882	0.00129606	0.02007201
RAI14	2214.71691	1.16356585	0.00130325	0.02016296
TESK1	407.070341	-0.770995	0.00130999	0.02024691
AAR2	757.588061	-0.760975	0.00131299	0.02027283
LINC00277	33.3112644	-1.9418688	0.00131492	0.02027297
ZIC2	2932.51255	-0.984875	0.00131564	0.02027297

PALD1	1875.49291	1.42150793	0.00131903	0.02030486
RAE1	366.667449	-0.648752	0.00132179	0.02032705
HOXC4	77.8287601	2.42320267	0.00132415	0.02034177
LINC01126	49.8742347	1.47032412	0.0013254	0.02034177
HEPH	505.005289	2.47096262	0.00132937	0.02038229
QRSL1	395.587687	0.82450477	0.00133126	0.02039095
SLC22A17	939.011107	1.87596439	0.00133499	0.02042776
FICD	144.788361	-1.3889604	0.00133916	0.02047108
HAUS4	420.474973	0.99966722	0.00134053	0.02047168
FAM149A	277.037583	0.94048603	0.00134621	0.02053805
BCAR3	698.789546	1.9795024	0.00135208	0.02060717
AKIRIN2	447.940677	-0.8612352	0.00136624	0.02080235
CCDC175	37.3896108	2.72100209	0.00137234	0.02083321
RPL36	3717.45865	-0.5776176	0.00136992	0.02083321
ZNF34	152.666586	1.14012939	0.0013711	0.02083321
GLI1	412.792325	2.2365659	0.0013782	0.02090154
CCDC146	209.745735	1.25231913	0.00138584	0.02099664
C7orf55	82.4254991	1.03656787	0.00139062	0.02100834
FLJ37453	65.0233476	1.45611682	0.00139345	0.02100834
LOC1005070(	22.2442451	2.22044638	0.00139216	0.02100834
OGFRL1	2309.00431	0.84791517	0.00139278	0.02100834
PLEKHA4	1439.30554	-1.423249	0.0013906	0.02100834
CGNL1	5844.24967	2.02958787	0.00140177	0.02109248
PTGER4P2-C	9.57455564	2.40369211	0.00140073	0.02109248
DUSP27	78.5811565	2.16912083	0.00141365	0.02125045
FAM103A1	229.91134	-0.9511391	0.00141905	0.0212917
PRAM1	108.949777	2.07283419	0.00141917	0.0212917
WISP1	538.764784	2.16655897	0.00142318	0.02133107
P2RX6	28.7386804	1.87154788	0.00142615	0.02135478
LOC1019279(	30.3768074	1.70226811	0.00143466	0.02146127
HCST	123.8559	1.78479943	0.00143622	0.02146368
EML4	2825.98344	1.03088331	0.00144685	0.02158061
NCR3LG1	755.583902	-1.9758151	0.001446	0.02158061
MIR4458HG	89.4632416	-1.7328132	0.00144982	0.02160391
TBC1D25	404.375936	-0.5938191	0.00145446	0.02165203
AGTR1	25.9660885	2.52317574	0.00146019	0.02167847
CHMP1B	4055.46689	-1.7577772	0.00146098	0.02167847
ERMAP	414.849023	0.96332662	0.00146188	0.02167847
SCN2A	51.1583949	2.58723713	0.00146054	0.02167847
ANKRD20A1	201.450185	2.68231403	0.00148594	0.02200586
PLXNA3	1042.1828	-1.2239455	0.00148927	0.02200586
PRICKLE1	2894.65632	1.40949386	0.00148749	0.02200586
SCN7A	16.8861966	2.64951885	0.00148969	0.02200586
DHX37	481.49721	-0.5261796	0.00149857	0.02209452
HNRNPD	1346.3911	-1.0437069	0.00149788	0.02209452
SLC5A6	1562.17968	1.94463907	0.00150285	0.02213637
SATB1	379.981436	1.0079687	0.00150719	0.02217911
ZNF114	42.6631289	-1.9431287	0.00150911	0.02218605
FPR2	27.4219221	-2.4673975	0.00151336	0.02222728
SLC7A6OS	281.567253	0.69834976	0.00151624	0.02224839

ATL2	2019.19872	0.82423937	0.00152486	0.02228961
CYFIP2	872.968479	-1.740585	0.00152409	0.02228961
INTU	615.860217	0.94858278	0.0015244	0.02228961
NAT9	633.369841	0.88192502	0.001521	0.02228961
CENPB	1527.75931	-0.5420918	0.00152794	0.02229738
CNPY4	520.14798	1.10232806	0.00152829	0.02229738
LOC1005072	964.125649	-0.6209755	0.00153173	0.02231152
NFKBIB	191.472929	-0.997279	0.00153217	0.02231152
NCMAP	13.2149307	2.38281038	0.00154277	0.02244246
RAP1B	1787.92277	-1.0148277	0.00154408	0.02244246
SLC25A35	83.2525919	1.16282881	0.00156788	0.02276693
TSPEAR-AS1	21.2904657	2.70641589	0.00157087	0.02276724
USP9Y	627.631464	-2.6606418	0.00157011	0.02276724
LOC1019288	15.9525686	1.94544699	0.00157666	0.02278664
PKD2	2052.80445	1.13804419	0.00157379	0.02278664
TLR7	370.044623	1.49162703	0.00157637	0.02278664
WHAMM	369.998098	-0.7851674	0.0015823	0.02284672
ENKUR	331.401741	2.30071518	0.00158621	0.02287739
IL16	523.56916	1.47773037	0.0015874	0.02287739
C5orf20	7.84033378	2.6789673	0.00159302	0.02292111
PDCD5	456.762476	-1.0946706	0.00159342	0.02292111
ST8SIA3	19.9043899	-2.5429331	0.00159692	0.02294994
MESDC2	1524.80499	-0.6787367	0.00159872	0.0229544
GTF2F1	1522.33263	-0.4504496	0.0016006	0.02295988
SEMA6D	905.59212	2.0953474	0.00160783	0.02304201
EIF2S2	1633.4857	-0.7326791	0.00161091	0.02306465
ACSF3	385.111581	0.6683215	0.00161878	0.02315582
ZNF512	1346.96089	0.51494357	0.00162199	0.02318015
MTRNR2L9	97.6114117	1.32837789	0.00162414	0.02318927
LOC1019268	38.7830338	-1.9411603	0.00162605	0.02319499
CCDC61	147.520262	-0.8988594	0.0016384	0.02332578
COMMD7	443.260818	-0.825851	0.00163839	0.02332578
FAH	204.895627	-0.7818016	0.00163977	0.02332578
DISC1	104.072874	1.0882703	0.00164805	0.02342182
ROBO3	383.986556	2.09076596	0.00165638	0.02349672
SLC24A4	50.8146034	2.05828656	0.00165579	0.02349672
SMURF2	1125.35617	0.67534422	0.00165924	0.02351558
CTDSPL	1541.21757	1.39531423	0.00167223	0.02367786
YWHAH	2441.69563	-0.6462981	0.00168282	0.02380579
CSDC2	189.15081	-2.345358	0.00168535	0.0238197
NAPSB	235.78173	2.48565535	0.00168849	0.02384219
TMEM139	8.47929031	-2.5262378	0.00169202	0.02387003
LARP1	5553.41802	-0.7707082	0.00169784	0.02393024
GREM2	116.584103	2.68578046	0.00170288	0.0239793
AGAP11	566.029792	1.92462528	0.00171319	0.02410237
TECR	1044.27913	-1.3429278	0.00171624	0.0241232
GALNT16	180.311506	1.77789096	0.00172635	0.02424307
CEP41	295.582255	0.73353496	0.00173451	0.02433536
DCAF13	391.661022	-0.737234	0.00174041	0.02436914
KIAA1107	193.673064	1.05534311	0.00174034	0.02436914

TMEM70	231.989601	-0.7280152	0.00174167	0.02436914
SLC39A10	1638.81575	0.76022306	0.00174934	0.0244542
FKBP4	912.815214	-0.8029926	0.00175331	0.02448732
ZNF317	926.738309	-0.3808276	0.00175619	0.02450529
KDELC2	1509.3758	0.94064811	0.00175888	0.02452051
IDH3A	826.166776	-1.1091803	0.00176461	0.02455588
ZFPM2	80.2586042	1.90157299	0.00176414	0.02455588
LEPREL4	447.662148	1.21287678	0.00177026	0.02461225
CLEC4E	151.988292	1.88829095	0.00177272	0.02462418
CHST3	1003.64861	1.02313226	0.00177946	0.02469544
SNORA53	774.029449	-1.3365489	0.0017942	0.02487747
C9orf3	708.341618	1.02281799	0.0018032	0.02495728
TRAK1	1623.48065	1.08405707	0.00180176	0.02495728
DGKG	37.536995	1.70573493	0.00180827	0.0250049
MMP16	981.938204	2.37744302	0.00181648	0.02509588
FAM198A	3093.95957	1.5176649	0.00182854	0.02522002
SERTAD4	126.676143	1.88168479	0.00182875	0.02522002
LRRC37BP1	425.63242	0.68152019	0.00183954	0.025346
CX3CR1	1354.54383	1.7822752	0.0018471	0.02540462
LOC645434	10.7650456	2.05456366	0.00184707	0.02540462
SUDS3	1235.22502	-0.4789124	0.00185587	0.02550249
LOC1001306	152.685402	1.59490899	0.00187182	0.02569861
ASPA	96.9660532	2.31264768	0.00187373	0.02570194
PIGB	346.864652	-0.9378317	0.00188609	0.02583829
POLRMT	610.530774	-0.6588279	0.00188704	0.02583829
MED29	1470.17342	-0.7881036	0.00189036	0.02586067
PCDHA8	44.8260866	2.33015456	0.00189846	0.02594837
TFIP11	484.730838	-0.7858709	0.00190221	0.02597652
BMP2	99.4266874	-1.8305145	0.00192219	0.02622298
LUM	213.953677	2.46324605	0.00192367	0.02622298
WARS2	361.536583	1.04839629	0.00192719	0.02624777
F2RL1	137.038433	-2.2882446	0.00193033	0.02625775
SMG7	1807.45107	-0.9966611	0.00193135	0.02625775
NUBP1	271.306642	0.56558349	0.00193855	0.02633244
THSD1	125.771946	1.23923999	0.00194866	0.02644632
MYBPC1	8.60745392	2.63771571	0.00195368	0.02649106
PRR14L	1960.5573	-0.668001	0.00196157	0.02657461
LCAT	216.499952	0.91639462	0.00200209	0.02709972
TGFBR2	4434.69312	0.92881427	0.00200672	0.02713837
GLRX2	58.7991202	-1.0652653	0.00201937	0.02728542
SNAI3-AS1	74.6683418	1.28097652	0.00202232	0.02730138
JUNB	4860.31668	-1.8825828	0.00202465	0.02730874
ANP32A-IT1	128.116673	1.57407945	0.00203271	0.02731971
GRIK2	44.1997464	2.1452981	0.0020283	0.02731971
LINC00597	80.3606321	1.72245518	0.00203108	0.02731971
NBR2	99.827054	1.12911242	0.00203435	0.02731971
NUDT4	199.691984	-1.5077331	0.00203301	0.02731971
CDH6	589.051441	2.02866708	0.00203885	0.02735619
SLC39A4	43.5847743	-1.6924599	0.00204362	0.02739625
PIK3CG	623.920631	1.41059055	0.00205516	0.0275269

RDH16	8.40161798	2.30528012	0.00206768	0.02764645
SLC26A5	9.56518186	-2.6664941	0.00206733	0.02764645
NDUFA3	597.143257	-0.8448146	0.00207062	0.02766172
SLC12A1	147.267009	2.43375192	0.00207665	0.02771814
TMC6	691.022946	-0.8456189	0.00208233	0.02776977
NOC4L	158.082659	-0.7410688	0.00208439	0.02777313
ANK1	86.8498862	-2.0693744	0.00208697	0.0277835
FIGNL1	220.920114	1.06374463	0.00208988	0.02778489
SNORA46	12.4002558	2.16244431	0.00209069	0.02778489
NAA60	801.908462	-0.7459324	0.00209369	0.02780062
FRMD7	26.9549816	2.57835215	0.00209737	0.02782545
CCDC170	150.710359	1.6453972	0.0021017	0.02785885
EME1	21.1547248	1.48743528	0.00211278	0.0279575
TOB1-AS1	20.6158435	1.6665956	0.00211221	0.0279575
C8orf44	114.733302	1.15087219	0.00213244	0.02814777
DEF8	946.412146	0.64970454	0.00213632	0.02814777
KCNK5	246.646324	1.56299694	0.00213273	0.02814777
RFT1	530.72553	0.54077775	0.00213408	0.02814777
SERPINA3	966.704281	-2.5018026	0.00213477	0.02814777
GNB4	2547.69907	1.45517974	0.00214316	0.0281895
VAV2	531.413803	-0.8811246	0.00214269	0.0281895
UBE2D3	4756.57677	-0.6403991	0.00214819	0.02823154
AQR	1636.8341	-0.6146704	0.00216447	0.0283345
FMN2	52.3346632	2.14676338	0.00216438	0.0283345
GLTSCR1	206.12884	-1.3464774	0.00216187	0.0283345
RASEF	156.407997	-2.5060049	0.00216159	0.0283345
TMEM130	263.267911	2.62767026	0.00216525	0.0283345
SUSD4	68.500865	-2.4996063	0.00217387	0.02842311
MLH1	787.310091	0.65655728	0.00218455	0.02853842
SPRY1	1389.03125	1.38204559	0.00219533	0.02864879
ZFAS1	992.367458	-1.1223857	0.00219672	0.02864879
AASS	2299.40202	0.85059392	0.00220014	0.02866904
FMO5	58.5665093	1.71109784	0.00220481	0.02870555
PLCE1-AS1	11.2526362	2.40870032	0.00220678	0.02870678
SSPN	1200.74157	1.9546392	0.00221341	0.02876876
CYB561D2	185.482238	0.78714236	0.00222082	0.02882116
MVB12B	871.773486	0.91885175	0.00222252	0.02882116
SACS	2445.84281	-1.0681295	0.00222307	0.02882116
AES	7053.52727	-0.6823209	0.00223101	0.02888621
HEXDC	186.67317	0.84999933	0.00223185	0.02888621
ZNF414	105.367721	-1.0230056	0.00223635	0.0289201
SLC30A3	56.8611321	2.57093413	0.00223836	0.02892174
CHKA	944.112405	0.84378103	0.00224085	0.02892956
MUC19	21.490604	-2.1898332	0.00224439	0.02895091
CARM1	722.227175	-0.6702399	0.00225357	0.02902065
NAB2	388.180718	-1.3987572	0.00225328	0.02902065
COBL	131.169822	-2.4707455	0.00229089	0.02945179
GTF2A2	647.870294	-0.9246291	0.00228961	0.02945179
NOTCH3	7360.07864	0.8715165	0.0022964	0.02949797
PRKX	891.59412	0.73260301	0.00229844	0.02949958

NCOA5	945.505977	-0.8285477	0.00230039	0.02949987
PTGES3	2947.71041	-0.8554173	0.00231446	0.02965563
MIDN	1686.75121	-2.0868606	0.00232324	0.02974323
IL2RB	38.6178735	1.64213066	0.00233894	0.02991939
SPAG9	11053.9516	0.52079278	0.00234177	0.02993068
MAGEE2	10.0354803	2.3432663	0.00234399	0.02993408
BAHD1	841.38072	-0.7131264	0.00234762	0.02995553
MORN4	129.596338	0.84683513	0.00236005	0.03007499
RGS7BP	231.11187	2.32013684	0.0023609	0.03007499
ARL2BP	1392.31505	0.65509527	0.00236863	0.03014849
KDM5D	567.106514	-2.5433664	0.00238025	0.03027133
FUK	403.96206	0.6878782	0.00238611	0.03029574
LNX2	736.742975	-0.8333505	0.00238586	0.03029574
LRRN1	598.930697	2.29235601	0.00238893	0.03030645
C19orf53	771.343773	-0.8464022	0.00239219	0.03032279
FGF2	1813.68146	1.2599169	0.00240577	0.03046983
CA4	22.5898453	-2.429281	0.00241068	0.03050679
SLC9A8	653.453598	-0.5596605	0.00241704	0.03054519
TAF1C	806.88931	0.69719018	0.00241769	0.03054519
PAPPA	2284.29454	2.38633423	0.00242385	0.03058089
SPTBN1	50663.927	0.8920119	0.00242615	0.03058089
ZNF326	798.244245	-0.6521044	0.00242649	0.03058089
MTMR12	1733.79122	-0.7140326	0.00243204	0.03062571
MCAT	121.185324	-1.0044616	0.00244502	0.03074288
PDCD4	1528.6192	0.72600964	0.00244534	0.03074288
TBK1	827.659151	-0.4974481	0.00245103	0.03078921
NXPH2	974.071519	2.59507224	0.00246774	0.03097371
CEACAM21	35.6245493	1.59400706	0.00248571	0.03102186
DUSP2	95.4746712	-2.0833422	0.00248071	0.03102186
GNG5	526.545085	-1.0913574	0.0024782	0.03102186
LAMA5	4826.57083	-1.6444022	0.00247718	0.03102186
MIF	1923.41238	-1.9807522	0.00248171	0.03102186
RNASE1	1415.55721	-1.9303846	0.00247387	0.03102186
SYT15	844.585675	2.16719439	0.00248457	0.03102186
UGDH	880.043925	-1.3028169	0.00248981	0.03104788
MAPK1IP1L	2655.83867	-0.7297425	0.00249261	0.03105753
ABI3	353.209479	1.33856676	0.0024994	0.03106706
DDX6	5669.10851	-0.3888884	0.00249739	0.03106706
LOC642852	768.615377	0.7607117	0.00249944	0.03106706
MT1E	104.058494	2.05301969	0.0025122	0.03120041
POLM	447.883138	0.6814265	0.00251666	0.03123052
SUPT7L	1247.09205	0.73430617	0.00252454	0.03130307
CEP162	494.580211	0.69217534	0.00254058	0.03145124
JAK2	2526.85435	1.50622381	0.00253924	0.03145124
OCEL1	115.97854	-0.8711741	0.00255199	0.03156702
GPR155	531.358302	1.50440013	0.0025633	0.03166753
INSIG2	1052.04531	-1.0189911	0.00256424	0.03166753
CCL20	22.7538508	-2.6093252	0.00257665	0.03179523
BTN3A3	510.573096	0.89182269	0.00259838	0.03203769
GSTM5	448.33971	2.5923928	0.00260272	0.03206547

SNHG20	186.982338	1.17690237	0.00260505	0.0320684
CCNI	4326.35966	-0.557331	0.00260845	0.03208454
RAB11A	4199.09482	-1.1989353	0.00263512	0.03238665
DMC1	16.5008516	1.96371232	0.00263805	0.0323968
ALG2	777.328734	-0.7219269	0.00264214	0.03242103
BICD1	245.135584	-1.529787	0.00265825	0.0325927
ENTPD3	9.03316618	2.30684839	0.00266909	0.0326995
FAM167B	30.8382027	1.8444988	0.00267673	0.03276701
PGR	3606.56502	-1.7714838	0.00269004	0.03290367
BGN	5883.25621	1.36548259	0.0026986	0.03298214
COL11A1	12340.7155	1.97090477	0.00270427	0.03302523
LINC01176	44.2002676	1.74333216	0.00270983	0.03303331
LOC1027248	535.344453	1.33393445	0.00271353	0.03303331
LOC729737	134.676546	-1.6641855	0.00271127	0.03303331
RALGPS2	374.673748	-1.9058734	0.00271297	0.03303331
JADE3	307.683704	-0.8659374	0.00271759	0.03305644
TMEM129	1252.1664	1.01994766	0.00272554	0.03312691
SNURF	16.4418519	-1.4631744	0.00273022	0.03315763
CCDC144A	116.57075	-2.3105987	0.00273279	0.03316256
KCNQ1	248.312115	1.18073202	0.00273953	0.03319194
KIF3B	2836.25411	-0.8047777	0.00273851	0.03319194
FNDC1	900.611392	2.56224653	0.00274729	0.03323359
WDR92	178.622588	0.74029363	0.00274717	0.03323359
APOA1	66.3467802	2.5791763	0.00275578	0.03331
GAPDH	24534.0633	-1.1518988	0.00276705	0.03341487
MAP4K1	85.0299023	1.46468406	0.00277058	0.03341487
PPP1R16A	324.290483	-0.8944872	0.00277098	0.03341487
BTG3	486.121075	-1.2905882	0.00278156	0.03351612
BTN3A2	481.83345	1.09491261	0.00279872	0.0336965
DSE	4423.60588	0.91690096	0.00280521	0.03374816
TUBGCP3	755.627444	-0.6428948	0.00282864	0.03400342
MYEF2	1475.06915	-0.8804644	0.00283548	0.03405905
FRMD6	887.78893	1.21731073	0.00284063	0.03409416
C10orf54	2039.32541	1.21429236	0.0028485	0.03414792
FAM109B	270.541162	1.11331041	0.00284955	0.03414792
EIF4EBP3	40.8949928	-1.6640875	0.00285424	0.03417752
SULT1C2	440.956961	-1.2445951	0.00287226	0.03436648
NEAT1	26477.3615	-1.1033341	0.00287652	0.03439063
EML5	259.327692	-1.5475332	0.00288091	0.0344163
AGPAT4-IT1	62.3842889	1.28980268	0.00288867	0.03448225
NEB	1432.06959	1.76468415	0.00289947	0.03458423
SNX29	2192.05013	1.03778345	0.00290722	0.03462783
UBXN6	1803.1188	-0.6486607	0.00290763	0.03462783
MYLK3	21.1208848	2.19202006	0.00292284	0.03478204
PPIF	509.854275	-1.0524345	0.00292564	0.03478845
FCHO1	126.653419	1.97147724	0.00293228	0.0348134
TTLL12	742.399067	-1.2688766	0.00293058	0.0348134
RASSF4	962.163056	0.95361511	0.0029357	0.03482712
EXOSC4	138.639981	-0.8180304	0.00294086	0.03486149
CHRFAM7A	10.757253	2.20495847	0.00294555	0.03489011

ABHD11	163.381071	1.07573867	0.00296258	0.03506486
SNAPC1	218.756113	-1.194794	0.00297417	0.03517489
PTGER3	11.6815415	1.94352015	0.00297675	0.03517827
SNORA52	43.6429687	1.46957672	0.00297915	0.03517961
CTSLP2	9.61279122	2.54234644	0.00298805	0.03520354
EIF1AY	121.447198	-2.4805034	0.00298358	0.03520354
PRDM4	1028.31715	-1.057526	0.0029861	0.03520354
CYP4F3	34.5207099	-2.2653625	0.00299911	0.03530675
CCDC141	42.995738	1.60572402	0.00301097	0.03541923
DAAM2	1036.90317	1.81277993	0.00301747	0.03546853
MIEN1	304.130226	-0.957332	0.0030333	0.03562731
LDLRAD2	31.7595682	2.53206727	0.00303894	0.03566631
TMEM121	24.2332417	1.32999932	0.00307107	0.03601594
NUFIP1	181.420972	-0.727589	0.00307862	0.03607696
A2M	11024.7866	0.89573135	0.00308284	0.03609885
PIP4K2B	2921.8594	0.36258101	0.0030874	0.03612469
CLPP	453.664694	-0.8380521	0.00309401	0.03615087
PLEKHA1	2007.76775	1.03603042	0.00309434	0.03615087
C21orf67	30.0983617	1.42554482	0.00312416	0.03647152
HIST3H2A	65.3431676	-1.6478531	0.00313892	0.03661597
DIS3	1958.53133	-0.5323076	0.00314818	0.03669615
FBN2	2639.3145	1.45454137	0.00316191	0.03679529
GLTSCR2	2516.34579	-0.8194435	0.00316252	0.03679529
NSUN5P2	294.889188	1.16157321	0.00316387	0.03679529
CLEC4A	87.044193	1.11546116	0.00317831	0.0369352
SNORA23	254.191952	-0.881863	0.00318111	0.03693988
KIF1A	1425.01563	2.4816623	0.00318599	0.03696861
LINC00265	310.145725	1.024333	0.00318997	0.03698676
BCORL1	351.064566	-1.0842495	0.00319848	0.03699771
C15orf48	17.6294457	-2.233204	0.00319415	0.03699771
CYP2R1	97.6245406	1.28615095	0.00320295	0.03699771
HACL1	421.115987	0.73933311	0.00319952	0.03699771
RNF111	1241.47248	-0.6789769	0.00320243	0.03699771
AXIN2	298.52086	1.58466632	0.00320783	0.03701084
MSS51	175.663225	1.16534565	0.00320891	0.03701084
TAF4	332.186531	-0.6672966	0.00321239	0.03702326
GMDS	1749.98286	-0.9330498	0.0032266	0.03715909
CYP27C1	22.5397006	2.36411783	0.00323023	0.03717311
SEMA3B	3159.17399	1.25545425	0.00324006	0.03725832
COBLL1	1311.48329	1.7940981	0.00326388	0.03750409
FMO4	192.744374	1.0512608	0.00327043	0.03755125
MRPL57	673.107418	-0.8105528	0.00327638	0.03756618
PREX1	2361.47887	1.12201465	0.00327662	0.03756618
EID2	189.668263	-0.6672142	0.00328338	0.03761564
EMC7	680.935883	-0.8655107	0.00330531	0.03783866
MST1	142.173869	1.44486958	0.00331338	0.03790276
TAF11	439.261956	0.61535857	0.00331636	0.03790863
ZNF276	868.383419	0.8890331	0.00331894	0.03790998
DKC1	609.919891	-0.612702	0.00332434	0.03794341
RNASEK	265.270071	-0.806703	0.00336946	0.03842987

LRRC29	69.0018162	1.00884724	0.00337715	0.03848904
PROX1	54.9220116	-1.6996932	0.00339182	0.03862749
LINC00997	128.553747	0.87417298	0.00339733	0.0386616
NR4A3	508.131194	-2.1661651	0.00341454	0.03882873
PATL1	776.122212	-0.8452867	0.00342346	0.0389013
CHRNA6	22.3900668	1.96866726	0.00342735	0.03891673
CLN5	1229.17779	-0.5113156	0.00344631	0.03901674
NME2	270.532226	-1.118631	0.00344356	0.03901674
PGM5	837.83776	1.69233032	0.0034457	0.03901674
TFPT	217.878056	-0.9476183	0.00344585	0.03901674
GGT1	60.6529871	-1.8597008	0.00345351	0.03904067
NPR3	2402.14729	2.50674827	0.00345275	0.03904067
AKAP10	1094.53949	0.40227115	0.00346824	0.03917833
SBNO2	969.561077	-1.4362144	0.00347373	0.03920196
ZFC3H1	2498.31334	-0.8514307	0.00347543	0.03920196
ISYNA1	4591.78995	-1.5332916	0.00348235	0.0392224
TMEM200B	305.672651	1.13761154	0.00348035	0.0392224
SIDT1	47.9918842	1.25581698	0.00350051	0.03936925
WDPCP	237.527479	0.71915482	0.00349823	0.03936925
ADD2	13.1277692	2.13667542	0.00351221	0.03943645
RPL3	18145.76	-1.0242033	0.00351419	0.03943645
TMEM56-RW	18.9787406	1.54373796	0.00351172	0.03943645
HMGCR	1093.22807	-1.3336381	0.00351743	0.03944404
ABHD15	363.004441	0.8756708	0.00353313	0.03959112
XPOT	1940.28243	-0.7103625	0.00353686	0.03960409
CDK2AP2	599.566306	-0.8737238	0.00354667	0.03966218
RECK	1530.12467	0.73368406	0.00354721	0.03966218
RASA4	62.7350254	0.88012525	0.00355937	0.03976916
ERBB4	336.871525	2.50175232	0.00358161	0.03996692
PBLD	174.833086	0.84708747	0.00358227	0.03996692
CLEC7A	1139.52455	1.68084247	0.00359211	0.04001852
DDX54	996.520457	-0.5097778	0.00359471	0.04001852
ZNF385A	376.138354	-1.0409636	0.00359062	0.04001852
TPRA1	329.0878	0.72854209	0.00360165	0.04006677
C22orf34	45.2551293	1.67187886	0.00360714	0.04009875
CHAMP1	340.003827	-0.8047356	0.00361037	0.04010563
LINC00886	109.668642	1.72141406	0.00361378	0.04011452
CREB3L1	184.528637	-1.7096712	0.00362231	0.04017081
IFI44L	495.007586	1.30292805	0.00362816	0.04017081
ITGA3	749.287059	-1.2804014	0.00363715	0.04017081
PTCH2	70.1153311	1.65545912	0.0036357	0.04017081
RFX3	697.808076	0.86147805	0.003633	0.04017081
SEH1L	772.61833	-0.9556692	0.00363668	0.04017081
TMEM9B-AS	81.6060258	1.54817593	0.00362435	0.04017081
FAM78A	284.265062	1.54452995	0.00364839	0.04023707
HTRA1	2845.45436	1.37921829	0.00364733	0.04023707
CNPY3	1155.56227	0.81690497	0.00365782	0.04031208
PAGR1	779.097538	0.8384417	0.00366621	0.04034971
SLC25A26	241.52624	0.69556034	0.00366648	0.04034971
LDLRAD4	2868.40546	1.06942442	0.0036723	0.04035588

SRSF8	860.913683	-0.5349742	0.00366975	0.04035588
S100B	255.093471	2.48054249	0.00368005	0.04041219
SYNE1	6761.84362	1.56281034	0.00368455	0.04043264
ANKLE2	1667.88201	-0.6429662	0.00370932	0.04067544
SNORA4	17.5672618	-1.2423123	0.003721	0.04077443
ADAMTSL5	311.53754	-2.2826709	0.00373241	0.04087029
IMP3	765.416541	-0.7221277	0.00374922	0.04099592
PODNL1	62.6210242	2.22673689	0.00374701	0.04099592
MSANTD2	245.666955	0.69601241	0.00376508	0.04114003
KLHL41	41.1790719	1.27526374	0.003776	0.04123001
KCNIP1	14.212397	2.17392464	0.0037911	0.04136551
PWAR1	71.1655992	-1.1726945	0.00379664	0.04139654
LINC01278	230.480392	-0.684067	0.003807	0.04148005
ATP5E	901.628521	-0.6872895	0.00381015	0.04148503
LY75	69.8569402	1.45254289	0.00381865	0.04150446
NTHL1	160.247309	1.31505721	0.00382004	0.04150446
SOAT2	8.07960137	-2.1340251	0.00381494	0.04150446
EPRS	3131.76518	-0.6996161	0.00385615	0.04186715
ARNTL2	341.78845	-1.523704	0.00387352	0.04187813
ATRIP	148.757408	0.73467031	0.00386955	0.04187813
PCDHA5	62.9617406	2.07760498	0.00387003	0.04187813
SMOX	607.468264	-1.0914964	0.00387083	0.04187813
STEAP2	363.635776	-1.8292168	0.00386184	0.04187813
TMEM249	9.33933557	1.89645333	0.00386588	0.04187813
TDG	442.454261	-0.6628629	0.00388557	0.04197891
ADAT2	194.505412	1.08118957	0.00389978	0.04210283
L3MBTL4	466.955964	-1.3594502	0.00391392	0.04219616
TXNL4B	242.443092	1.44350597	0.00391361	0.04219616
TARDBP	2376.10115	-0.6563145	0.00392583	0.04229486
CD63	7763.12171	-0.9811484	0.00393437	0.0423566
TSPO	368.72756	1.27304487	0.00393707	0.0423566
BANF1	421.291428	-0.9957828	0.00397914	0.04274991
SEC22C	1303.6948	0.66662538	0.0039792	0.04274991
TFB1M	151.371265	0.52684602	0.00398315	0.04276248
AP3B2	72.2881313	-2.0226753	0.00401203	0.04304249
AKT1S1	534.860349	-0.7892534	0.0040192	0.04304493
HSPD1	3247.57027	-0.7068148	0.00402066	0.04304493
TK2	1836.74658	0.8350414	0.0040164	0.04304493
BAG2	311.561156	-1.1691903	0.00403648	0.04318412
PLB1	237.616987	1.47173207	0.00404773	0.04327433
GPD1L	1210.20225	1.4216505	0.00405738	0.04334741
LRP5	1271.63977	1.35243465	0.00406992	0.04345114
ZNF787	270.616451	-0.595875	0.00407566	0.0434822
EXOC2	834.897091	0.58776092	0.00408176	0.04350329
LPCAT2	1025.88718	1.46986571	0.0040833	0.04350329
ETF1	2367.16457	-0.8969317	0.0040967	0.04361585
CSE1L	2036.90037	-0.8349835	0.00410362	0.04365923
SF3B4	538.194434	-0.8257873	0.00411027	0.04369966
SMARCA2	4190.34138	0.66851549	0.00411867	0.04375869
SPRYD7	236.913348	-0.9671113	0.00412666	0.04378307

TAZ	291.307271	0.63490156	0.00412531	0.04378307
DUOX2	80.4563664	2.34280424	0.00414889	0.04396349
PRIMPOL	338.390164	0.7138067	0.00414939	0.04396349
NPHP3-ACAD	205.255879	0.93226007	0.0041631	0.04404803
SMNDC1	521.374888	-0.6424269	0.00416096	0.04404803
COL6A2	23773.1427	1.01173033	0.0041763	0.0441573
RNF126	280.708955	-0.8583644	0.00418965	0.044268
BACE1	1516.96102	0.75233009	0.00420358	0.04427422
CYSTM1	816.169712	-1.2067543	0.00419484	0.04427422
SEC24B	1665.40519	-0.431667	0.00420465	0.04427422
SPECC1	785.760665	1.24752297	0.00419632	0.04427422
TRIB3	87.2153927	-1.7070157	0.00420061	0.04427422
ELF4	655.485023	-0.6386217	0.00421218	0.04429276
LIF	75.4067912	-1.7039243	0.00421023	0.04429276
ZNF384	970.393564	-0.5951447	0.00421987	0.04434332
TFRC	3808.55352	-1.6979353	0.00422555	0.04437257
IPO7	3856.71136	-0.489279	0.00424169	0.04451167
PRSS12	21.0185288	2.28308138	0.00426337	0.04470867
CEBPA-AS1	18.6475724	2.2550176	0.00429784	0.04502269
LOC1005056	16.3490005	1.91734039	0.00430017	0.04502269
TOMM40L	209.589683	0.82860163	0.00430211	0.04502269
ARHGEF33	34.828765	1.39031013	0.00430787	0.04505227
ABCC1	864.913499	-1.4232937	0.00431098	0.04505409
ATXN2	1540.02207	-0.6299254	0.00431414	0.04505654
HBA1	168.99997	-2.002627	0.00432258	0.045114
TMEM189	462.128107	-1.1173413	0.00434733	0.04534147
LOC1002722	21.8485916	1.36692879	0.00436305	0.04545857
POLR2D	485.730441	-0.456986	0.00436447	0.04545857
KCTD9	807.634382	-1.0784736	0.00437752	0.0455505
MME	330.840528	2.1853328	0.00437923	0.0455505
NSDHL	219.965161	-1.0325212	0.00443829	0.04613362
PSMA3	627.013997	-0.7553206	0.00444323	0.04615366
SUCLG2-AS1	88.8195429	1.49556731	0.00445037	0.0461935
TRPC2	8.43493326	2.20632458	0.00445307	0.0461935
CHODL	83.2223357	-2.2103944	0.00446703	0.04629632
SH3YL1	678.748378	0.96438275	0.00446901	0.04629632
ZNFX1	3377.87384	-0.5750269	0.0044732	0.04630845
CD274	40.4797719	-1.8932199	0.00448197	0.04634508
FLNC	5960.55601	-1.7212581	0.00448277	0.04634508
GALNT5	81.1655502	2.29244925	0.00449224	0.04638058
PRPF19	999.997067	-1.0534254	0.00448969	0.04638058
TCAP	10.4147918	1.80494836	0.00449862	0.04641522
HIST1H2BD	595.216306	-0.927806	0.00451493	0.04655224
LOC1005060	26.8551045	1.806199	0.00452818	0.04665754
PTPRH	32.3434457	-2.4177288	0.00453841	0.0467316
MFNG	231.208167	1.29774331	0.00455069	0.04682671
EXOSC8	299.969157	-0.4883218	0.00455375	0.04682683
ANO7	31.810667	-1.4312154	0.00455984	0.04685809
CACNA2D2	608.652128	1.97659266	0.00458517	0.04705686
FAM135A	924.014718	0.67461453	0.00458963	0.04705686

FIGNL2	20.9742092	2.15120985	0.00459041	0.04705686
KMT2B	1434.14547	-0.5486376	0.00459354	0.04705686
KXD1	962.993582	-0.4971066	0.0045945	0.04705686
IRF5	421.383919	1.45364017	0.00462829	0.04730829
KLF4	1721.57795	-1.4022912	0.00462354	0.04730829
SPRED2	821.713837	0.63901366	0.00462821	0.04730829
NUDCD3	2093.1852	0.57505034	0.0046423	0.04741995
TFAP2C	7.6003886	-2.4265632	0.00465527	0.04752083
CCDC106	221.489061	-0.6257376	0.00467663	0.04765638
MAD1L1	516.581002	0.95385913	0.00468174	0.04765638
NBPF25P	51.105665	1.18095656	0.00468021	0.04765638
SEZ6L	18.8336861	2.14236424	0.00468406	0.04765638
SPATA5	576.914671	0.73228329	0.0046779	0.04765638
FGD2	665.032595	1.53879883	0.00469439	0.0477299
GOLGA6L4	13.878887	1.88268195	0.00470543	0.04781052
PFDN2	199.39352	-0.8746354	0.00471567	0.04787908
ZNF22	430.991345	0.63479798	0.00471841	0.04787908
NGRN	2147.27543	-0.6632978	0.00474103	0.04807682
DNAJA4	461.963553	-1.3101654	0.00475212	0.04815756
SDR16C5	55.9285003	-2.4336986	0.00477164	0.04832352
PTGES2	478.725272	-0.7724978	0.00478096	0.0483563
RNF146	1455.28828	0.66149803	0.00478118	0.0483563
LRRC4	209.755607	-1.4293095	0.00478814	0.04839483
POU5F2	73.7166378	1.11159135	0.00480543	0.04853773
RABL3	449.923354	-0.5600102	0.00481803	0.04863303
C1orf198	1981.53371	1.36570115	0.00482955	0.04871727
ADAMTSL3	11346.8086	1.33378161	0.00483684	0.04875882
USP51	253.518638	-0.6990367	0.00485347	0.0488943
GK	274.767826	-1.3057542	0.00485706	0.04889846
ANKMY1	183.647706	1.26971888	0.00486759	0.04890827
LOC1019275	118.514229	0.89953683	0.00486675	0.04890827
RPS21	2260.71272	-0.9531145	0.00486731	0.04890827
DRD2	76.020568	2.41637434	0.00487508	0.04895157
ASCC2	586.700311	-0.6699698	0.00491346	0.0490698
COPS2	1624.32248	-0.7564258	0.0049188	0.0490698
DDX39A	406.437537	-0.6300132	0.0049125	0.0490698
GPR108	676.720148	-0.6842188	0.00490566	0.0490698
JUND	2663.23311	-1.215925	0.00491874	0.0490698
MORF4L1	5002.28688	-0.7635909	0.00491237	0.0490698
PROSC	894.391423	-0.790867	0.00489158	0.0490698
SETMAR	296.746333	0.67956345	0.0049081	0.0490698
SNAP25	7.57294924	-2.412649	0.00491794	0.0490698
TBC1D12	716.652852	0.83285056	0.0048981	0.0490698
RAP1GAP	119.121417	-1.9770545	0.00492225	0.04907241
PRORSD1P	79.6076777	1.11038767	0.00493173	0.04913499
CASP9	272.17402	-1.0467894	0.00494747	0.04925988
ABHD3	389.812588	-1.5400805	0.00497017	0.04935081
ATP11C	1021.63946	-1.3605881	0.0049823	0.04935081
EFS	751.993317	1.62185257	0.00497751	0.04935081
FBXW4	604.714617	-0.4727689	0.00497978	0.04935081

ILF3	4679.53799	-0.4624052	0.00497863	0.04935081
KATNAL2	184.777875	1.08345051	0.00497884	0.04935081
SNTB2	2911.68422	1.08996591	0.00496356	0.04935081
TUBGCP4	434.508139	-0.7217065	0.00496759	0.04935081
PGRMC2	857.411361	-0.8960796	0.00499889	0.04948317
LOC54944	82.3704147	1.19042269	0.00500348	0.04949677
LINC00921	147.940769	1.05330492	0.00500723	0.04949893
PCSK9	14.2954902	-2.1346842	0.00501014	0.04949893
ESR1	133.791613	1.72507809	0.0050161	0.04952592
PIF1	18.0904565	1.95033385	0.00503058	0.04963696
KIAA1407	214.123935	0.90404029	0.00507595	0.05002044
MYOC	21.0768466	2.39737302	0.00507369	0.05002044
ZSWIM1	309.665711	-0.7910636	0.00507962	0.05002447
PCK2	185.746518	-1.0293677	0.00508563	0.05004825
PRMT1	1207.6115	-0.6893355	0.00508855	0.05004825
EXOG	300.305399	0.82049064	0.00509339	0.05006384
PRX	888.113092	1.58357682	0.0051008	0.05010464
MANEA-AS1	66.2514296	1.07221063	0.00511903	0.0502194
NOP2	506.598964	-0.7834196	0.00511811	0.0502194
AMICA1	202.23829	1.51204904	0.00512409	0.05023704
CHST14	442.00275	0.77402327	0.00515124	0.05046315
MYZAP	104.711389	-2.1468688	0.00515372	0.05046315
CNTNAP1	399.923518	1.26571066	0.00517312	0.05062079
NIFK-AS1	125.226642	0.78418471	0.0051958	0.05081041
PLEKHM3	1078.96716	0.60903747	0.0052222	0.05100356
PUS1	155.094306	-0.7572114	0.00522197	0.05100356
RAB3A	38.5295575	-1.033119	0.0052341	0.05108735
DUSP18	151.335418	-1.0659097	0.00524738	0.05118446
RPGRIP1	21.2342004	1.91685198	0.00525122	0.05118942
NBPF3	191.531176	1.41973751	0.00525696	0.05121283
SERTAD2	651.63665	1.25307418	0.0052628	0.05123725
GFPT2	913.193518	-2.0144945	0.00526977	0.0512402
WWC2-AS2	36.4797207	1.63994249	0.00526844	0.0512402
COPZ1	2018.90799	-0.6689963	0.00527972	0.05130448
EMILIN3	282.382772	1.66600285	0.00528369	0.0513106
SNHG17	104.338164	-1.0333303	0.00528881	0.05132787
FXR1	2337.41296	-0.4515734	0.00530512	0.05142228
PAK7	26.9370176	2.40677579	0.00530523	0.05142228
RPS24	8948.84043	-0.7393111	0.00532896	0.05161967
SNORA57	413.070802	1.24474057	0.00533522	0.05164778
RASAL3	182.950122	1.47872672	0.00534021	0.05166349
PERP	2454.5767	-1.514132	0.00535604	0.05178404
LOC1019297	29.7956737	2.33888347	0.00537	0.0518537
WWOX	413.075054	1.0181118	0.00536965	0.0518537
ATP2B4	10472.8047	-0.5719629	0.00540073	0.05192203
CRIP1	252.917061	-1.5950535	0.00539156	0.05192203
FCGR1B	66.3665783	1.74343337	0.00539462	0.05192203
LRRC8C	2328.58739	0.92948767	0.0053982	0.05192203
7-Sep	4725.21259	0.72181406	0.00539324	0.05192203
SNORA67	41.3997011	-1.1327201	0.00539545	0.05192203

TRIM45	274.794529	1.61311646	0.00539124	0.05192203
LRRC37A3	440.010854	1.08334846	0.00540616	0.05194173
LDHA	6155.82395	-1.5205028	0.00541064	0.05194824
SLC4A11	20.9424808	1.87415629	0.0054136	0.05194824
SLBP	953.213651	0.88858789	0.00542063	0.05195077
STOML3	19.6805618	2.27717339	0.00542006	0.05195077
TRAF3IP3	109.229071	1.70640459	0.00542746	0.05198386
LATS2	1630.90016	-0.5884934	0.00544545	0.0521236
SIGLEC11	129.526581	1.95359592	0.00545168	0.05215079
ARF6	1262.29643	-0.6723114	0.00545842	0.05218273
SOBP	1050.17179	-0.8192136	0.00546217	0.05218615
EYA2	1741.98559	-1.258822	0.00547011	0.05222954
S100P	7.74589596	-2.2647469	0.00547931	0.0522849
SCG3	9.47792533	-2.0631676	0.00549801	0.05243078
CPXM2	3578.98004	2.07988864	0.00550898	0.05250278
ZNF358	621.633613	-0.4538321	0.00551517	0.05252921
MLYCD	205.481454	0.68919755	0.00553146	0.05261911
MTRNR2L3	10.8170388	1.80599175	0.00553125	0.05261911
NFX1	1548.53448	-0.3729	0.00554044	0.05263929
TRAF5	2177.13821	-1.3650539	0.0055382	0.05263929
TPM4	9982.53535	-1.155501	0.00556311	0.05279166
WIPF2	1047.75443	-0.601418	0.00556335	0.05279166
MLST8	390.15046	-0.6987792	0.00558499	0.05296431
LTB4R	257.512496	0.80496163	0.00561261	0.05319341
HIST2H2BF	332.351609	-0.9893516	0.0056231	0.05325994
BYSL	147.4095	-0.8004617	0.00563383	0.05329587
TMEM106B	2398.71186	0.61822833	0.00563214	0.05329587
GBP4	473.554439	1.04031069	0.00566444	0.05348667
RBM7	451.266952	-0.5877163	0.00566349	0.05348667
TRG-AS1	20.2224238	1.78080901	0.00565978	0.05348667
HIST1H2BG	225.047871	-1.2446802	0.0056777	0.05354606
SMDT1	214.733376	-0.9828367	0.00567515	0.05354606
ALKBH5	2344.59298	-0.5702067	0.00568913	0.05355518
HMHA1	1172.00814	1.43150713	0.00568536	0.05355518
PDLIM3	65.37204	-2.3498282	0.00568788	0.05355518
FKBP9P1	26.0738365	1.29584385	0.0056947	0.05357484
LINC00987	46.5823733	1.91413452	0.00571075	0.05369291
WSB2	1378.8081	-0.9425419	0.00572698	0.05381259
FBXO46	238.186363	-0.8340805	0.00573547	0.05385941
KLF2	3528.15281	-1.5263096	0.005753	0.0539581
SLC35E2B	1177.18222	0.96783405	0.00575043	0.0539581
RPS5	4838.99549	-0.6776026	0.00576297	0.05401861
RMDN3	617.08848	-0.5361272	0.00576857	0.0540381
CNOT6	1093.36341	-0.7001025	0.00577377	0.05403933
HACE1	506.556143	0.69488713	0.00577573	0.05403933
BRCA1	228.52391	1.08560142	0.00579223	0.05409483
DUSP7	147.789695	1.31856731	0.00578878	0.05409483
GPI	4457.97874	-0.9191332	0.0057884	0.05409483
FLI1	747.148109	1.07245473	0.00584709	0.054553
SPATA9	10.5972607	2.22988883	0.00584839	0.054553

TAGLN2	2073.69851	-1.6308281	0.00586482	0.05467306
FBLL1	11.080242	1.98149134	0.0058724	0.05471052
NDUFA12	495.598842	-0.9423462	0.00587598	0.0547107
COX7B	823.269229	-0.7587049	0.00588087	0.0547231
EXT1	3330.34304	1.44839709	0.00589148	0.05478861
DCDC2	14.7736588	-2.3422376	0.00589608	0.05479821
FZD6	774.186672	-1.0040606	0.00591812	0.05493657
RSPO4	15.3483399	1.68906915	0.00591499	0.05493657
SPINT1	157.07264	-1.7526396	0.00592324	0.0549509
PTPRG	3790.15635	1.17307659	0.00593435	0.0550208
GOLGA8H	36.2222209	1.6806787	0.00594058	0.05504086
IGSF9	42.7722313	-1.7608637	0.00594726	0.05504086
MXD1	343.625657	-1.657027	0.00594375	0.05504086
CILP2	133.149458	2.26242451	0.00596176	0.05514181
PODXL2	342.028293	2.12893069	0.00598269	0.05529323
STARD7-AS1	98.4038485	0.99472777	0.00598533	0.05529323
MEST	5227.96506	2.00374498	0.00600748	0.05546448
ABAT	495.83127	1.41716875	0.00603092	0.05554736
GSN	34035.9455	0.8637443	0.00602824	0.05554736
HSPH1	1937.1675	-1.1506188	0.00602554	0.05554736
OXCT1-AS1	18.2243302	1.8466691	0.00602669	0.05554736
ADAT1	435.901932	0.71020176	0.0060659	0.05576662
BACH2	260.627005	1.35783007	0.00607595	0.05576662
ETV1	190.094043	1.87771985	0.00608013	0.05576662
GZMH	14.7094119	2.03008981	0.00606386	0.05576662
MED10	254.968107	-1.1444386	0.006078	0.05576662
MPPED1	184.714301	-1.9503509	0.00606087	0.05576662
ZNF480	661.346692	-0.774435	0.00607639	0.05576662
ROM1	49.1018086	1.53333339	0.00610593	0.05596978
MSC	57.4764036	2.03685243	0.00611361	0.05597961
PER3	1926.03401	1.31806953	0.00611429	0.05597961
NPHP1	168.948689	0.65595056	0.00615204	0.05629174
ZNF684	81.0366929	1.22905461	0.00616342	0.05636223
SCRN2	471.902037	0.80345763	0.00616785	0.05636921
ELOF1	454.820673	-0.5234879	0.00618642	0.05650529
NTN4	1046.25628	1.769466	0.00620026	0.05656452
PRKG1	865.912566	1.85246837	0.00619937	0.05656452
FOXC1	7271.62787	-0.7606055	0.00620737	0.05659572
TRIM11	417.473651	-0.7886323	0.00621563	0.05663745
CRHR1-IT1	31.5870508	1.41971432	0.00623243	0.05667495
OLA1	1208.0769	-0.7737675	0.00622667	0.05667495
PSMD12	743.841274	-0.7309243	0.0062345	0.05667495
RNF24	553.635975	-1.0721983	0.00622869	0.05667495
PSMG3-AS1	264.726927	0.95576347	0.00624236	0.05671284
COA1	549.90965	0.65763462	0.00624763	0.05672716
AHCY	1541.36753	-0.8173328	0.0062607	0.05681222
MED14	1781.55149	-0.6409135	0.00626917	0.05682228
PITPNM1	816.010078	-0.8933565	0.0062692	0.05682228
ITGA9	706.015287	1.12365643	0.00627695	0.05685898
CPEB2	1446.1123	-1.5406269	0.00629988	0.05703299

ABHD2	5238.79053	-1.1006704	0.00631989	0.05710748
ARHGEF5	272.852536	-1.2048539	0.0063241	0.05710748
CTNNBIP1	532.668721	1.01315524	0.00631777	0.05710748
ZFP36L2	4110.33204	-0.61359	0.00631907	0.05710748
ZNF25	725.74189	0.70189071	0.00632669	0.05710748
GAMT	147.018926	1.29431793	0.00635446	0.05729086
LINC00924	26.839918	2.13603408	0.00635444	0.05729086
WDR1	3814.34217	-0.6299205	0.00636342	0.05733795
LILRA1	114.698399	1.65271218	0.00638569	0.05746022
MON2	4322.31521	-0.59166	0.00638821	0.05746022
PDGFRL	536.862403	1.85651015	0.00638687	0.05746022
MURC	12.441667	1.91868483	0.00641937	0.05770675
MCF2L	585.354626	0.9842648	0.00642377	0.0577125
DDIT3	279.576202	-0.9847499	0.00645628	0.05792074
EPDR1	680.336868	1.56314778	0.00645826	0.05792074
IL18RAP	31.1864504	-2.1387476	0.00645231	0.05792074
ZDHHC12	128.534406	-0.8368496	0.006464	0.05793842
NBEAL1	2032.87816	0.3834086	0.00647103	0.0579417
NDUFB9	490.590299	-0.5530805	0.00647191	0.0579417
EZR	3754.64909	-1.1193297	0.00649062	0.05807537
ANKK1	19.8563435	1.73026759	0.00649816	0.05810901
CDK4	1158.6844	-0.6434397	0.00651447	0.05819216
SPAG8	79.956226	0.99907014	0.00651504	0.05819216
MBLAC1	25.1858643	1.23922983	0.00652449	0.05824277
TMEM200A	16.9610331	1.80814012	0.00655785	0.05850656
FAM73B	409.053904	0.60805879	0.00658038	0.05867347
SAMM50	397.921477	-0.5834757	0.00663735	0.0591471
FLJ31104	16.0476047	1.53308964	0.00664713	0.05919988
LOC1005067	11.4941136	1.68173561	0.00666483	0.05932315
AK5	22.3350981	2.26576041	0.00667436	0.05937361
HYAL2	846.671491	0.99322128	0.00668522	0.05943576
SCARNA2	6210.67929	0.96707866	0.00672466	0.05975186
RYR3	257.441492	2.27464652	0.00673995	0.05981853
SERPINB8	256.672309	-1.6571425	0.00673681	0.05981853
SPNS2	144.807339	1.23603041	0.0067511	0.05988284
AGMAT	13.8389129	1.9561846	0.00676438	0.05995177
NCK1-AS1	33.9057831	1.61558819	0.00676667	0.05995177
MICU2	946.096201	-0.7402513	0.00677234	0.05996739
B3GAT1	19.5626417	2.23558024	0.00679854	0.06014674
TXNRD3	371.833879	0.6660887	0.00680042	0.06014674
UHMK1	4054.39965	-0.4405359	0.00680981	0.06019515
CD4	1628.28617	1.32762309	0.00683116	0.06027977
TECPR1	887.768973	0.6961032	0.00682691	0.06027977
UBE2D4	502.545497	0.77887832	0.00683064	0.06027977
CASP10	407.237585	0.61954351	0.00684817	0.06036053
LOC1005061	20.3046911	1.50413516	0.00684599	0.06036053
MECR	258.4632	0.72791764	0.00687771	0.0605862
LRRC16B	25.0619005	1.3419573	0.00688893	0.06065025
ARHGDIA	3201.94333	-0.668803	0.00691101	0.06079006
LETM2	29.8523789	-1.0075349	0.0069246	0.06079006

LINC01089	333.524185	0.77953793	0.00692441	0.06079006
SRGAP2	1735.40579	1.01537806	0.0069201	0.06079006
SRP68	1080.78229	-0.8655293	0.00692221	0.06079006
IRF2BPL	1062.76523	-0.8414028	0.00693685	0.0608168
PHTF2	811.943573	0.73673762	0.00693952	0.0608168
TAS2R43	46.8945166	1.25770961	0.00693591	0.0608168
PITPNM2	430.544934	-0.8886033	0.00697158	0.06106294
TOLLIP	863.766534	-0.7899734	0.00698088	0.06107479
TP53RK	641.687689	-1.013827	0.00697866	0.06107479
ADIRF	3759.95979	1.68713721	0.00703418	0.06150604
COLGALT2	10.9106686	1.94996136	0.00704013	0.06152307
GHR	1299.0121	1.14847061	0.00704971	0.06157177
GATS	1424.40862	0.64008458	0.00706595	0.06167848
TAPT1-AS1	169.636474	1.15485247	0.007078	0.06174861
MYO18A	1638.97096	0.80806076	0.00708488	0.06177358
CABLES2	195.688057	-0.7352934	0.00713123	0.06214238
SERINC2	107.28324	-1.9427326	0.00713798	0.06216597
ESF1	628.549147	-0.5566083	0.00718524	0.0625073
TLCD1	10.3497282	-1.8555128	0.00718531	0.0625073
C12orf49	419.527339	-0.688104	0.0072094	0.06261049
CDC37L1	369.211247	-0.5691954	0.00720356	0.06261049
TJP3	29.3103814	-1.5976888	0.00720753	0.06261049
C12orf73	73.4372231	-1.0649642	0.00722328	0.06269558
ARRDC2	1072.12634	1.5708803	0.00723063	0.06272395
BOC	11113.5165	1.81491738	0.00725088	0.06279327
CHD1L	680.583377	0.52085352	0.00725024	0.06279327
SRSF10	140.595619	0.82017986	0.00725079	0.06279327
CPOX	223.129005	0.70513735	0.00726255	0.06281193
MTHFS	29.3821961	-1.2565275	0.0072653	0.06281193
NCF1C	25.7971583	1.55141004	0.00725969	0.06281193
SH3BP4	1642.1337	1.6206421	0.00727603	0.06286935
COL26A1	384.016502	2.10314801	0.00729216	0.06287821
LSM12	292.038539	-0.8405369	0.00728868	0.06287821
RAD51D	178.60773	0.78669974	0.00728955	0.06287821
RPL38	3447.95227	-0.4821361	0.00729343	0.06287821
ADH1B	26.5286855	-1.9703748	0.0073148	0.06302264
ADIPOR2	2040.47034	-0.7335326	0.00732249	0.06302264
XRN2	2153.06961	-0.5653177	0.00731844	0.06302264
SV2A	44.1864489	1.86844666	0.00733404	0.06308672
PLD1	3092.83477	0.84481974	0.00734698	0.06309202
SLMO2	882.455811	-0.9412073	0.0073464	0.06309202
SORCS2	822.57012	1.94340347	0.00734163	0.06309202
CD27	7.7957634	1.92418216	0.00737802	0.06318325
CTNND1	278.138919	0.51679977	0.00738307	0.06318325
CYBB	4938.10071	1.40462726	0.00739007	0.06318325
DCLK2	231.737116	1.16041198	0.00737963	0.06318325
HNRNPH3	1404.91045	-0.6427833	0.00738917	0.06318325
MPV17L2	140.114982	-0.8459426	0.00738823	0.06318325
NOX5	107.600461	2.1796257	0.0073905	0.06318325
PRICKLE3	88.2219394	-0.6711175	0.00736915	0.06318325

HIVEP3	722.855534	1.86532146	0.00739963	0.06322611
HUWE1	12238.7401	-0.4486838	0.00740723	0.06324559
LSM10	133.172765	-0.9496905	0.00741014	0.06324559
CLIP2	473.556028	0.81549874	0.00744286	0.06348954
TMTC2	54.5947022	-1.3659803	0.00746408	0.06359992
ZNF121	534.873851	-0.4051375	0.00746274	0.06359992
ZNF669	106.314061	-0.7531012	0.00748319	0.06372741
SMIM5	27.7284606	1.63584513	0.00749412	0.06378517
R3HDM2	1775.1684	-0.6330495	0.00750114	0.06380956
POLA2	274.386463	0.61720191	0.00750828	0.0638349
SLC25A28	480.668182	-0.556711	0.00753047	0.06398818
IL20RA	104.065319	2.12079863	0.007569	0.06425068
ZNF10	748.984149	-1.1500049	0.00756973	0.06425068
IGFBP2	7059.60828	1.41312977	0.00758304	0.06432815
UBE2S	180.095574	-1.0833865	0.00758941	0.06434661
ASPSCR1	343.047408	0.62286931	0.00759508	0.06435916
STK17B	1044.85282	1.26036067	0.00760076	0.06437182
RAB13	720.126362	-0.8574646	0.00760869	0.06440348
VPS33A	480.236178	-0.8368431	0.00761633	0.06443268
GLG1	11866.4483	0.67005036	0.00762418	0.06446356
SNORA61	11.0725563	1.66148981	0.00764631	0.0646151
MMGT1	1105.88396	-0.5987064	0.00765844	0.06464646
PCDHGB4	368.694987	-0.9565322	0.00765843	0.06464646
FRMD6-AS1	29.4816387	1.22633427	0.00770937	0.06490064
LOC1005059	12.4639537	1.76856245	0.00769407	0.06490064
RAPH1	1477.30909	0.8711342	0.00770002	0.06490064
SHROOM3	634.078267	1.98127505	0.00770967	0.06490064
VAC14-AS1	68.4376204	2.20355525	0.00770882	0.06490064
ZNF730	91.5774868	-1.436863	0.00773427	0.06507204
BNIP3	382.471684	-1.407517	0.00776275	0.06524021
GPR133	1563.15874	2.02167081	0.00776228	0.06524021
TTLL3	324.672825	0.95891928	0.00776884	0.06525567
ICA1	115.718893	1.35876553	0.007796	0.06544804
DOCK9	4787.93503	0.97142341	0.0078117	0.06554402
MAP2K5	402.377008	-0.7040252	0.00781708	0.06555334
USE1	206.902131	-0.7429111	0.00782697	0.06560053
TNFSF8	240.131628	1.4122632	0.00783127	0.06560073
TRAP1	852.620819	-0.6931946	0.00783619	0.06560622
CSTF1	599.726686	-0.7788793	0.00784259	0.06562405
TRMT112	1121.3565	-0.5825469	0.00785495	0.06565592
UBE3A	2583.90617	-0.4669294	0.00785364	0.06565592
IER2	863.165094	-1.5134806	0.00789771	0.06595175
LOC148413	202.808861	0.8526193	0.00789893	0.06595175
SPTLC3	8255.60719	-1.7586843	0.00790376	0.06595623
GSTM4	288.805546	1.19610045	0.00792069	0.06606163
DMTN	500.802834	-1.7351257	0.00798053	0.06632648
EIF2B2	377.381999	-0.587895	0.0079811	0.06632648
LINC00574	29.0388719	1.94267768	0.00797289	0.06632648
NUDT19	240.27041	-0.5004712	0.00797592	0.06632648
SSX2IP	506.087801	-1.2279517	0.00797591	0.06632648

TNFRSF12A	220.165073	-1.9473642	0.00796549	0.06632648
UCP2	329.207594	-1.159234	0.00798266	0.06632648
FOXF2	19.9680528	2.03923557	0.00798715	0.06632785
ATP1B3	1822.01232	-1.1686768	0.00802033	0.06656747
TPPP	226.629199	-1.7950416	0.00803043	0.06661532
GLYR1	1762.29186	-0.5004085	0.00805	0.06674161
CRBN	870.317965	0.6176827	0.0080596	0.06678517
KLF6	5417.8722	-0.8833639	0.00807419	0.06684706
SELPLG	725.757249	1.40830304	0.00807578	0.06684706
GOPC	1357.97961	0.57097761	0.00808504	0.06685171
NDUFB7	886.834614	-0.7556038	0.00808191	0.06685171
M1AP	14.6149004	1.64672265	0.00813201	0.06720389
PPP6R1	1191.42577	-0.6437353	0.00813688	0.06720803
RHPN2	274.223033	-1.8035016	0.00814372	0.0672284
UBE2A	979.359293	-0.8139187	0.0081537	0.06727461
ABI3BP	8614.5548	1.87934653	0.00816544	0.06733526
PDLIM2	801.183813	1.38716047	0.0081789	0.06741008
ADAM20P1	141.191103	-0.8601814	0.00818796	0.06744859
WDR11-AS1	13.8614301	1.83939513	0.00819993	0.06751097
ABHD14A	188.86038	1.17470391	0.0082625	0.06789308
EIF5AL1	8.47456302	-1.6338665	0.00826699	0.06789308
GPR153	63.233811	-1.3973248	0.00825246	0.06789308
OSBPL3	539.264774	1.07921273	0.00827285	0.06789308
RIPK2	278.009988	-0.8026875	0.00827183	0.06789308
TRIB1	675.908156	-1.5716538	0.00825756	0.06789308
DKFZP586I14	460.445177	1.12758755	0.00828062	0.06791099
TIMM50	466.137783	-0.6350487	0.00828388	0.06791099
FAM208A	3531.87339	0.50064451	0.0083109	0.06805992
ZFAND5	5739.64132	1.00927036	0.00831047	0.06805992
RDH10	756.488735	-1.7183095	0.0083491	0.0683363
VMO1	75.9393112	1.65181775	0.00836481	0.06840499
WDFY4	851.345962	1.3586931	0.0083664	0.06840499
DPM1	429.490835	-0.7262715	0.00840441	0.06867923
CORIN	57.4719522	2.07669011	0.00841677	0.06870717
GSTM3	1273.74312	1.83534267	0.00841451	0.06870717
PRR7	12.1501755	-1.4299817	0.00842284	0.06872017
GIMAP6	585.166965	1.1895216	0.0084305	0.06874619
DAAM1	804.613945	-1.136564	0.00843927	0.0687812
CCDC107	220.476268	-0.5807312	0.00846058	0.06891831
RNF10	2305.71036	-0.4929873	0.0084739	0.0689902
AUTS2	1006.03029	1.24280433	0.00848028	0.06899549
EDC3	618.821809	-0.5241197	0.00848802	0.06899549
ZMYND15	175.588981	1.38258949	0.00848451	0.06899549
EXO5	114.386721	0.77318936	0.00850303	0.06906938
GORASP1	849.635114	0.51364057	0.0085061	0.06906938
MFSD11	554.465914	0.62734648	0.00852542	0.06918969
BZRAP1-AS1	24.8855349	1.48533827	0.00853469	0.0692283
CCDC110	16.3140832	1.42820072	0.00854574	0.06926044
MYOCD	21.8451903	2.12575001	0.00854767	0.06926044
FGFR2	1292.57862	-1.7229866	0.00856767	0.06934939

PKIB	131.224848	1.5769596	0.00856506	0.06934939
TNC	1267.60577	2.19974053	0.00857855	0.06940088
STK32A	493.495778	2.12100871	0.00860102	0.06954605
MGAT4A	2165.30472	1.01776079	0.00861802	0.06964686
FAM120C	890.91617	-0.5846444	0.00863265	0.06972839
RGS9	22.7276753	1.61329407	0.00865117	0.0698413
BCL2L11	429.231012	-1.1229747	0.00866869	0.06994591
MORC2-AS1	7.45298089	-1.8220587	0.00868222	0.07001838
IER5L	192.522488	-1.6919382	0.00870923	0.07016251
PLEKHG4	363.305239	1.31250603	0.00870669	0.07016251
ARF3	3421.39134	-0.7182598	0.00872897	0.0702479
SHOC2	1528.05745	-0.4028552	0.00872754	0.0702479
ABCG2	2347.84575	1.62355872	0.00876274	0.07037604
KLF10	954.730266	-1.4360508	0.0087678	0.07037604
MUC1	197.51254	-1.1735379	0.00876276	0.07037604
PLEKHO1	626.337192	0.92719896	0.00876607	0.07037604
PPM1F	947.836107	0.44605834	0.00876655	0.07037604
PIP4K2C	794.454507	-0.5797377	0.00878999	0.0705173
MMS22L	394.180242	1.03772529	0.0087971	0.07053746
SNORA5A	13.0989972	1.42483809	0.0088044	0.07055919
CCDC126	417.714998	1.2229236	0.0088208	0.07061697
TCERG1	1821.70129	-0.5131537	0.00881853	0.07061697
PHYHIP	39.45624	-2.2308314	0.00882987	0.07065279
DYNC2H1	2220.88265	0.64436302	0.00885761	0.07083779
PPP1R12C	1537.53938	-0.908578	0.00886317	0.07084538
EI24	1191.47307	-0.4242771	0.00887195	0.07087871
DPP3	450.023904	-0.656549	0.0088995	0.07106188
IL17RE	103.604426	2.06295339	0.00890784	0.07109154
FCGBP	13309.0031	1.60690829	0.00892092	0.07115894
CHPF2	1844.74838	0.76841101	0.00893417	0.07122763
GNB1L	48.3760629	-0.9420377	0.00895878	0.07138683
ABHD14B	781.600865	0.5853844	0.0089732	0.07145073
HIST1H2AB	55.9553943	1.15641291	0.00897611	0.07145073
RASD1	176.955029	2.00831235	0.00898131	0.07145514
PDCL3	203.786746	-0.955786	0.00902998	0.07180519
HSPA5	7314.67382	-1.303964	0.00904919	0.0718888
PDGFRA	205.392105	2.16547375	0.00905454	0.0718888
PHYHD1	157.583198	1.24764913	0.00905413	0.0718888
PTP4A2	3226.84788	-0.704154	0.00906842	0.07196188
CCT8	2282.15214	-1.0036805	0.00907951	0.07201262
NMB	109.31017	-1.0868209	0.00909005	0.07205905
TMEM230	2519.72454	-0.7512751	0.00912777	0.07228345
USH1C	300.662788	-2.1684176	0.00912757	0.07228345
PTBP1	2999.19156	-0.3721825	0.00913649	0.07231526
PIN4	236.624737	-0.7418978	0.00916728	0.07250738
TLE1	380.438331	1.20454552	0.0091702	0.07250738
IPW	608.988149	-0.8046759	0.00918607	0.07255259
ITGB5	2291.28917	1.0765388	0.00918125	0.07255259
PCDHGA8	450.429286	-0.9195288	0.00919009	0.07255259
CYP2B7P	12.1955835	1.84375963	0.00919588	0.07256104

C18orf21	194.684383	-0.6465737	0.00920467	0.07259307
BATF3	24.4471352	1.48836305	0.00924088	0.07284124
UBR2	2826.24153	0.43129018	0.00926827	0.07301974
ELF3	24.3104731	-1.9276697	0.00928223	0.07309223
NUDT11	53.8892802	1.64932348	0.00930864	0.07323422
PITRM1-AS1	24.7323494	-1.7024562	0.0093098	0.07323422
RPLP0	16651.1451	-0.9123955	0.00931588	0.07324456
CRYGS	26.0362381	1.43476244	0.00932457	0.07327537
RRM1	785.594222	-0.493554	0.00935826	0.07350251
ACSBG2	34.7856684	2.20638795	0.00936866	0.07354662
NAPA	1340.13479	-0.5837446	0.00937547	0.07356252
C17orf89	166.395062	-0.7555288	0.00941333	0.07382182
TET3	1388.07738	0.58459026	0.0094184	0.07382393
LRG1	11.0903406	-1.9057267	0.00946805	0.07413745
TIMM10	176.021198	-0.6140807	0.00946724	0.07413745
CTGF	29240.038	1.77305823	0.00948921	0.07426526
DCTD	961.302125	0.45411668	0.00949577	0.07427581
LOC730102	164.40765	0.98230644	0.00950023	0.07427581
LOC1019276	17.1893707	2.22724771	0.00951563	0.074342
LOC642846	211.292025	1.27192927	0.00951837	0.074342
LOC1019280	105.479101	1.13344916	0.00953391	0.07442556
ANKRD20A5I	292.642858	2.14910169	0.00954336	0.07444666
GLP2R	119.017227	2.23541123	0.00954631	0.07444666
C19orf47	213.412892	-0.6758564	0.00955576	0.07448255
TNNC1	1434.93952	2.00321136	0.00958417	0.07466612
ADRBK2	685.189931	0.97336151	0.00959878	0.0747083
COPS6	845.753744	-0.7121558	0.00959931	0.0747083
C2orf44	214.667813	0.56028939	0.00960421	0.07470861
COPZ2	1547.46558	1.13437015	0.00961378	0.07474519
MAPK13	124.449061	-1.3098361	0.00962817	0.07481922
CSF1R	4792.8147	1.12513789	0.0096355	0.07483716
NBPF1	1849.49724	1.20044721	0.00964022	0.07483716
DHX9	3967.50242	-0.5474721	0.00965955	0.07487373
LOC1001328	228.449664	1.72970888	0.00965603	0.07487373
TRPS1	1656.79154	0.97588446	0.00965736	0.07487373
RSRP1	1287.04825	0.85255051	0.00966481	0.07487667
ZCCHC2	505.507437	-1.2631822	0.00967431	0.07491249
CNTRL	1281.15673	1.19604675	0.00971551	0.0751748
HGF	206.770217	1.66742069	0.00971797	0.0751748
CCDC9	395.367642	-0.6074914	0.00975334	0.07530916
FXYD6	774.625818	-1.8407192	0.00974884	0.07530916
HMGN1	2075.59088	-0.6085259	0.00975108	0.07530916
MAPK3	819.29063	-0.8345902	0.00975495	0.07530916
LOC729683	32.3884052	1.29470743	0.00983065	0.0758183
TUFT1	229.194848	-1.1068637	0.0098357	0.0758183
YWHAZ	8186.4971	-0.8161692	0.0098345	0.0758183
XPO7	1958.01973	-0.7139926	0.0098444	0.07584729
FAM120A	4716.95777	-0.4829917	0.0098572	0.07590788
GRIN3A	7.98485296	2.17027165	0.00987243	0.07598708
SLC39A13	978.733426	0.5668914	0.00990058	0.07616552

LAMA2	1161.97241	1.67260818	0.00990905	0.07619255
UBALD1	168.673047	-1.0363535	0.00992273	0.0762596
MRPS35	544.792212	-0.5728002	0.00993177	0.07629093
NDUFS6	395.316076	-0.7661136	0.00994946	0.07638856
GVINP1	315.86407	0.85564677	0.00997025	0.07647174
PSTPIP1	106.765793	1.70406107	0.00996804	0.07647174
MRPL42P5	8.5364222	-1.408739	0.00999016	0.07658626
TLL2	40.7624096	-1.9182298	0.01001401	0.07673082
LGALS3BP	8979.79081	1.0168013	0.01003033	0.07681754
LOC1019272	7.538106	2.22287448	0.01003555	0.07681925
CRISPLD1	195.870434	1.53510351	0.01005218	0.07685091
FAM106CP	11.0478267	-2.2255862	0.01006971	0.07685091
PA2G4P4	155.597915	1.58299366	0.0100624	0.07685091
PCOLCE2	300.082692	1.80394997	0.01006111	0.07685091
PLEKHG5	638.278767	1.39525173	0.0100651	0.07685091
SNRPB2	716.785996	-0.7125274	0.01005518	0.07685091
BRD7	1018.84323	0.64676254	0.01009611	0.07699451
MRE11A	922.537943	0.63562381	0.01009854	0.07699451
FBF1	416.282767	1.10675131	0.01011275	0.07706461
APLN	104.909337	-2.0735965	0.01014036	0.07712195
C3orf62	176.454726	0.97738588	0.01013674	0.07712195
SLC25A5	1620.39647	-0.6099555	0.01013779	0.07712195
USP35	250.749761	0.67938316	0.01013873	0.07712195
WDR4	227.411392	-0.6471639	0.01017032	0.07731152
CA13	79.1582145	1.81524665	0.01019251	0.07742688
FXYD5	2308.23454	1.33786045	0.01020445	0.07742688
LRRC24	39.1666255	1.05133243	0.01020636	0.07742688
SLC12A6	1747.88086	-0.895897	0.0102061	0.07742688
TMED2	4177.50256	-0.8144747	0.01021069	0.07742688
CCDC94	269.192415	-0.4632054	0.01025818	0.07766748
HPCAL4	105.387743	2.0673437	0.01026264	0.07766748
MFAP4	1615.57849	1.61916923	0.01025319	0.07766748
USP53	8674.43168	0.69321291	0.01026146	0.07766748
USP19	1529.99852	0.6872562	0.01027937	0.07775576
SCAMP3	800.813212	-0.7418379	0.01029352	0.0778245
LINC01004	1889.19714	0.89314914	0.01033917	0.07813119
TWF1	1433.45757	-0.7414539	0.01036173	0.07826313
LY86	187.258619	1.11458171	0.01037642	0.07833559
AEN	927.031334	-0.8849041	0.01039528	0.0783663
APBB2	7303.77704	0.91611942	0.01041619	0.0783663
KSR2	15.4158506	2.10893726	0.01039345	0.0783663
SLC9A9	789.520906	1.31532227	0.01040887	0.0783663
TNFAIP3	2568.28527	-1.2373684	0.0104079	0.0783663
TTC31	506.905955	0.56524609	0.01039747	0.0783663
USP47	2721.63689	-0.3950933	0.01041319	0.0783663
HMGCS1	900.230015	-1.0870752	0.01042449	0.07839034
LGR6	16.4692208	-1.8961528	0.01044739	0.07852412
EDEM2	452.859519	-0.4535732	0.01046285	0.07860183
CDC37	2159.73443	-0.6693254	0.01050061	0.07884694
ZNF621	1481.80059	0.56677989	0.01051545	0.07891983

GOLPH3	1883.87943	-0.6402983	0.01053625	0.07899088
KCTD18	357.170337	0.52862132	0.01053414	0.07899088
SLC25A45	260.649489	1.28324634	0.01054362	0.07899088
SLC29A2	8.74885183	1.93483039	0.01054549	0.07899088
LOC1019273	44.1568067	1.3639894	0.01056472	0.07905786
PUS10	162.07259	0.76294008	0.01056039	0.07905786
SLC16A7	114.722437	-1.5138463	0.01058619	0.07917993
ANKRD52	1715.84866	-0.8387521	0.01062619	0.0794018
LGMN	1101.51156	-0.6696233	0.0106211	0.0794018
ST7	562.864051	-0.6781433	0.01063519	0.07943046
LINC01011	42.4934814	1.55395596	0.01065076	0.07943765
MFSD7	86.0782421	1.15258906	0.01065167	0.07943765
RTKN	303.043696	0.82653677	0.01064406	0.07943765
VAPB	2235.12563	-0.8163227	0.01070048	0.07976293
TSC22D1	14388.8061	-1.3686087	0.01071664	0.07984472
ZSWIM7	187.541293	0.78890634	0.01073602	0.0799503
TP53BP2	1222.53973	-0.8074188	0.01074476	0.07997659
TUBB2A	225.197346	0.7430645	0.01076842	0.08011389
RUSC1-AS1	65.2266463	1.02257776	0.0108081	0.08037017
MRPL51	718.000253	-0.6584857	0.01082835	0.08048183
FLYWCH1	991.307237	-0.4697722	0.01084541	0.08053452
PPFIA2	89.6981074	2.07269869	0.01084592	0.08053452
ARHGEF35	56.0961588	-1.2782036	0.01086649	0.08064087
LAMTOR1	900.461166	-0.6690914	0.01087074	0.08064087
MAP3K11	744.72854	-0.6887674	0.0108822	0.08068069
MYLK-AS1	22.6225481	1.52600335	0.01088662	0.08068069
LOC1001336	8.29975271	2.04975469	0.01090874	0.08074495
LPHN2	1827.85925	1.67806256	0.01091105	0.08074495
MTERF	304.736524	0.53118603	0.01090729	0.08074495
OSMR	1571.47352	-1.018425	0.01092946	0.08076444
OTUB1	705.416723	-0.5312181	0.01092682	0.08076444
UST	1555.74884	1.01141241	0.01092792	0.08076444
LINC00925	53.193842	-2.0248828	0.01093916	0.0807973
UBE2B	1051.56531	-1.0836225	0.01095103	0.08084604
CCDC85C	580.904476	-0.7943237	0.01097555	0.08098819
COL16A1	3156.95697	1.2672934	0.0109898	0.08105442
LEPR	39697.8685	1.8634587	0.01100006	0.08109116
AGBL1	8.17003753	-2.1915483	0.01103117	0.08128147
CD9	4953.48172	-1.1247512	0.01103892	0.08129958
LOC1005071	27.404959	1.59968802	0.01106139	0.08142606
HIST1H1A	21.5826669	2.14733008	0.01108306	0.08154646
ARIH1	2245.46319	-0.6668105	0.0111065	0.08166654
CCT6P1	80.7171221	0.7403263	0.01111001	0.08166654
AK3	2762.21064	-0.8668782	0.01111623	0.08167318
CA3	200.702776	2.15902257	0.01113843	0.08179721
TMEM134	158.726788	-0.6534052	0.01114939	0.08183858
CCDC64	17.9862912	-1.5456823	0.01115587	0.08184698
PLXNC1	7517.41721	-1.4538436	0.0111616	0.08184994
ZNF280B	43.860952	1.25196015	0.01118907	0.08201224
GIMAP8	575.085061	1.18340517	0.01120783	0.0821106

TSEN54	133.781897	0.74102233	0.01121662	0.08213581
SMAD4	3558.15404	-0.4278095	0.01126486	0.08244973
NME9	11.7170292	2.11347499	0.01127976	0.0825142
POU2F2	161.246444	1.42606573	0.01128978	0.0825142
TNFRSF1A	2119.02661	-0.5942522	0.01128644	0.0825142
LOC1001333	50.5127273	1.05044832	0.01132916	0.08276269
NUP93	862.819383	1.15987996	0.0113429	0.08282369
WBP2	1255.03733	-0.7929956	0.01135879	0.08290029
NIN	2011.85083	0.52141616	0.01136458	0.08290316
CTSF	2402.81408	1.52112559	0.01137071	0.08290848
FAM102B	768.073484	0.82063776	0.01138429	0.08296814
GSPT1	3459.8283	-0.4573285	0.01143293	0.08328316
AIFM1	469.849115	-0.7409933	0.01145607	0.08337261
PIWIL4	79.829072	0.76033099	0.01145148	0.08337261
GIMAP4	685.793544	0.97827728	0.01146353	0.08338742
SNORA72	10.0270442	1.82668242	0.01151001	0.0836859
ERVK13-1	1473.22412	0.62531761	0.01152292	0.08374009
AMPD3	630.619579	-0.616751	0.01153253	0.08375
BLCAP	2168.26861	-1.1844943	0.01154063	0.08375
C17orf62	828.678965	0.5901094	0.01153653	0.08375
CTNNB1	11377.7415	0.5688304	0.01158012	0.08399685
TP53INP2	978.361523	-1.0219246	0.01159161	0.08404051
ANK3	1248.82988	1.8047666	0.01161637	0.08418033
AGPS	1410.30085	1.11768524	0.01164259	0.08425111
CYP4B1	715.09119	1.72813212	0.01164133	0.08425111
DHX38	1345.26257	0.66752792	0.0116377	0.08425111
GNAO1	48.82836	1.80652355	0.01166537	0.08436625
PTPN3	306.577474	1.71365402	0.01166948	0.08436625
MGC12916	71.115532	1.60335472	0.0117161	0.08466346
TSNAXIP1	69.0586404	1.11096723	0.01172616	0.08469631
ARPC5L	203.428083	-0.6430936	0.01173794	0.08470169
MST1P2	19.5424044	1.80811423	0.01173402	0.08470169
PRICKLE2	877.575193	1.114655	0.01175699	0.08479938
CROCCP3	469.901858	1.03061761	0.01177728	0.08484602
IQCH-AS1	233.44847	0.87185825	0.01177707	0.08484602
MRPS12	198.315599	-0.5174547	0.01178478	0.08484602
POMP	784.951265	-0.8218135	0.01179107	0.08484602
RRN3P2	156.849791	1.64856363	0.0117907	0.08484602
ZFP69B	38.8783425	1.20952777	0.01179708	0.08484948
KLKB1	51.5619803	1.92583915	0.01182339	0.08499894
ANKRD33B	2674.18333	-1.2765883	0.01184965	0.08514789
SDF2L1	134.677156	-1.5081266	0.01186205	0.08519712
OLFML1	3090.54923	1.65436313	0.01188696	0.08533618
SIGLEC16	24.2645024	1.95018173	0.0118931	0.08534037
SCNN1A	200.111516	-1.8863573	0.01191417	0.0854516
RGS12	441.940423	0.67016681	0.01193459	0.08551823
RPL36A	694.359893	0.4455443	0.01193267	0.08551823
ZFYVE20	1313.95963	0.51330526	0.01194143	0.08552738
PPP2R2A	1106.16221	-0.9367403	0.01195956	0.08560943
TMBIM4	1467.7521	-0.4024935	0.01196403	0.08560943

FAM66C	164.214065	1.28351863	0.01199847	0.08581585
MICALL1	683.835819	-0.969235	0.01201486	0.085862
ZBTB8A	433.600878	1.12257807	0.0120161	0.085862
DLEU2L	53.8957355	1.08202942	0.01203194	0.08592475
SYDE2	320.155263	1.23910636	0.01203606	0.08592475
MEG8	18.2397408	2.04495475	0.01205477	0.0860183
GPD1	15.6764621	-1.5257106	0.01209621	0.08622905
HAAO	154.742686	1.21126899	0.0120955	0.08622905
POLR2E	1221.57508	-0.575877	0.01210114	0.08622905
ANKAR	392.243931	0.91042612	0.01213796	0.08645128
PCDHGB6	352.791659	-0.9231206	0.01216471	0.08656158
ZFAND6	1145.37637	-0.7311297	0.01216001	0.08656158
BORA	95.7490588	-0.858972	0.01217093	0.08656572
ZXDB	494.603176	-0.589579	0.01220811	0.08679003
ADRA2C	11.5340166	2.00407218	0.01224067	0.08697006
LOC1019287	15.6533694	1.94674934	0.01224476	0.08697006
DCUN1D2	386.485311	-0.8753964	0.01225617	0.08698461
NISCH	7703.39504	1.11589587	0.01226379	0.08698461
POLR3K	91.3711705	-0.6557407	0.0122602	0.08698461
CC2D2B	18.2479119	1.34870788	0.01227828	0.08704719
ACTB	46107.4743	-0.5134931	0.0123292	0.08736783
RBBP8	224.52344	-1.1408577	0.01234797	0.08746048
PCED1B	87.6950483	1.17631792	0.01237363	0.08756152
TUBB3	91.4782777	-1.9705648	0.01236829	0.08756152
GATA6	124.846916	1.99321873	0.01238199	0.08758033
EIF4ENIF1	518.538129	-0.8658233	0.01242742	0.08786124
DLG3	779.808457	-1.073398	0.01244704	0.08795947
AKR1E2	20.8283005	1.6689944	0.01245544	0.08797839
INHA	36.7614782	1.48172804	0.01246346	0.08799453
NTN1	2780.96135	1.45737621	0.01247337	0.08802406
TTC9	236.113772	-1.3700688	0.01249582	0.08814199
C11orf21	29.9027709	1.60621092	0.01251679	0.08824939
BAIAP2L1	64.9057238	-1.9949339	0.01254323	0.08835472
TPGS1	62.1179234	-0.6391963	0.0125432	0.08835472
RAB5B	3766.39688	-0.7233766	0.01256487	0.08842609
WARS	1134.08013	-0.7903092	0.01256291	0.08842609
LRRTM2	40.667254	1.35685995	0.01260552	0.08867153
STRAP	1386.86214	-0.7164289	0.01264682	0.08892133
HIF1AN	1571.10543	-0.4959442	0.0126546	0.08892776
ZNHIT2	83.2112235	-0.816626	0.01265931	0.08892776
CCDC15	59.1707065	1.35384391	0.01267002	0.0889623
HIPK3	6317.4852	-0.4068309	0.01271034	0.08916393
SMARCD2	1374.61133	1.00521244	0.01270862	0.08916393
NANOS3	7.88082578	1.83248167	0.01272677	0.08922281
ZNF493	1755.87981	-0.9276973	0.01273035	0.08922281
WLS	3145.602	1.0583769	0.01275245	0.08933694
ALAS2	23.8985802	1.37490006	0.01277172	0.08936903
BAG1	762.17339	-0.5767134	0.01277448	0.08936903
RNF135	577.454322	0.62743072	0.01276445	0.08936903
LOC440300	228.284384	1.12076217	0.01278904	0.08943011

LCK	25.3099055	1.63343593	0.01279805	0.0894524
ACTA1	12.4499255	-1.7479328	0.01283234	0.08950076
CARF	722.290507	0.67013402	0.01283409	0.08950076
CDK5	80.7500806	0.84852581	0.01283329	0.08950076
GRAMD2	7.75029244	2.10342388	0.01283266	0.08950076
VRK3	530.504744	-0.6658628	0.01282424	0.08950076
TAC3	11.1270365	-2.1511168	0.01285172	0.08958304
ANXA2	21801.5297	-0.9159774	0.0128743	0.08963354
HSPBP1	284.946946	-0.6102234	0.01287647	0.08963354
JADE2	1686.74085	-0.6202711	0.01287005	0.08963354
NOD1	328.176075	0.95706637	0.01288658	0.08966329
CALCRL	1559.60948	1.79455337	0.01289983	0.08967423
XYLB	114.624916	0.95877346	0.01289506	0.08967423
SRGAP2D	201.868732	0.89247886	0.012913	0.08972522
HDDC3	129.016499	0.55557214	0.01293031	0.08976429
PIGH	277.35287	-0.5484607	0.01292672	0.08976429
RPP25	57.3610275	-1.6111925	0.01293717	0.08977134
RNF125	335.71073	0.95694575	0.01298713	0.09007734
ISCA2	213.149307	-0.9617053	0.01300152	0.09013641
SCARF1	304.125729	1.17046164	0.01301246	0.09017163
COL22A1	122.903024	2.04693	0.01306161	0.09026788
DNMT3B	24.6227202	1.3535831	0.01304581	0.09026788
MSANTD1	9.12744994	1.76035689	0.01303374	0.09026788
PRC1	324.666084	-1.220675	0.0130606	0.09026788
SPCS2	637.955961	-0.6567004	0.01304898	0.09026788
TXLNGY	211.082728	-1.9989383	0.01305426	0.09026788
HPS5	1529.46749	0.89898443	0.01307774	0.09030248
IL20RB	27.5016225	-1.2736452	0.01307837	0.09030248
RPRD1B	1018.5957	-0.4364319	0.01320727	0.09115149
C11orf63	327.785641	1.43597033	0.01323277	0.09120995
CCR2	55.9959789	1.68657571	0.01323084	0.09120995
MIEF1	980.577958	-0.7215146	0.01323355	0.09120995
ADAM15	1363.21393	0.3689438	0.01325428	0.09128132
CEBPD	3496.89097	-1.1609285	0.01325579	0.09128132
ACKR2	63.0838158	1.48618525	0.01328647	0.09132886
MYOM2	58.4315623	-1.3397811	0.01327367	0.09132886
SOX8	11.9030025	1.66906978	0.01328546	0.09132886
SSRP1	1591.02197	-0.5186456	0.01327865	0.09132886
PRKRIP1	30.5468273	-0.9071841	0.01329523	0.09134822
TTPAL	728.727823	-0.7611834	0.01332343	0.09150106
CSNK1G1	956.463879	-0.5371808	0.01334103	0.09158096
MOB2	286.880047	0.49066947	0.01336994	0.09173848
DUS3L	287.419096	-0.5995398	0.01340432	0.09185128
LIMD2	315.987836	0.96953323	0.01339561	0.09185128
TARS	1416.6938	-0.8448602	0.01339842	0.09185128
PDE7A	550.661263	-0.9402008	0.01342473	0.09195011
ZHX2	710.451805	-0.6667506	0.01344714	0.09206258
ARL8A	686.978067	-0.7876863	0.0134769	0.09216188
GLDC	113.638132	1.34333557	0.01348564	0.09216188
PEBP4	7.48676337	1.82149003	0.01348426	0.09216188

RPUSD1	223.77928	-0.7381874	0.01349164	0.09216188
TDRD5	16.8341169	-1.9148271	0.01347877	0.09216188
FAM155A-IT	33.7477966	1.64747647	0.0135059	0.09221825
FAM179A	56.0749799	1.94281465	0.0135333	0.09232328
INVS	750.58609	0.68397545	0.01353219	0.09232328
TEX264	434.229037	0.6059701	0.01354468	0.09235991
ACRBP	52.447134	1.19162126	0.01357781	0.09254477
ERBB3	45.6968625	-1.5267038	0.01359283	0.09260603
CXCR6	34.0585697	1.47363083	0.01361624	0.09268337
FAP	119.491631	1.94470741	0.01361298	0.09268337
PAX2	30.4114573	2.13300185	0.01362598	0.09270856
IMPA2	117.774648	-0.9660414	0.0137059	0.09316982
SLC10A3	502.913216	-1.0039697	0.01370081	0.09316982
ASB13	234.807771	0.71223059	0.01371602	0.09319735
COX6B1	1385.55273	-0.7302967	0.01376295	0.09343361
CYSLTR2	24.8997499	1.4794409	0.01376215	0.09343361
ZNF429	337.885104	-0.6095791	0.01378253	0.09352517
SULF1	9167.88048	1.74503943	0.01385683	0.09398783
SAMD9L	2620.5053	0.62779906	0.01386881	0.0940276
CBLN2	17.1366856	2.11873979	0.01388742	0.09407226
ZC3H12A	129.094281	-1.5575011	0.01388765	0.09407226
RNF167	1024.35642	-0.462398	0.01391011	0.09418289
MSMO1	621.820118	-1.2143866	0.01392169	0.09421978
FAM222B	757.479503	-1.1392406	0.01394026	0.09430396
RASSF9	122.276869	1.43745201	0.01395479	0.09436065
LMLN	278.776749	0.85246728	0.01396358	0.09436289
LOC1001315	1110.80075	0.78764043	0.0139674	0.09436289
HMGB2	909.140359	-0.6546948	0.01397673	0.09438441
HES1	1252.1155	-1.3831132	0.01400409	0.09452762
SSR4P1	43.2473923	1.44923888	0.01406867	0.09488812
WDR18	255.540012	-0.659385	0.01406985	0.09488812
NPEPL1	55.38818	-1.0829692	0.01409286	0.09500158
SLITRK4	58.0844232	1.87064924	0.01410916	0.09506974
LOC1019291	332.231321	-1.1048953	0.0141261	0.09514217
HNRNPU	10066.2382	-0.492643	0.01413525	0.09516204
C1orf159	76.5101019	1.18117511	0.01414766	0.09519173
LINC00893	222.568307	0.7232719	0.01415823	0.09519173
NCOA6	1661.3417	-0.5873852	0.01415824	0.09519173
AATF	636.446405	-0.6897299	0.01417102	0.09520668
VN1R1	125.802301	-1.1057423	0.01417286	0.09520668
CPEB1	60.2799211	-1.6153324	0.0142083	0.09540303
CPPED1	962.022313	-1.2218205	0.0142257	0.09547814
NECAP1	705.161736	-0.4644584	0.01426602	0.09570695
GMEB2	458.509627	-0.7002756	0.01429208	0.09581209
RFTN1	436.899241	1.35022421	0.01429417	0.09581209
TNKS1BP1	2760.76764	-0.7634318	0.01433083	0.09601592
ITGB1BP2	27.0817955	1.54011991	0.01435044	0.0961054
TRPM2	634.591586	1.21133763	0.01435939	0.09612341
YIPF4	747.767067	0.63178676	0.01437119	0.09616057
SEC23B	1287.54239	-0.7388893	0.0143885	0.09623447

ARMC12	13.6935065	1.54347963	0.01443399	0.09649672
TSPY26P	46.974596	0.97777477	0.01444733	0.09654388
SMS	508.84413	-0.7658577	0.01445988	0.09658569
GAS1	5092.35674	-1.2123916	0.01447145	0.09662098
ENPP4	1341.46199	-0.8251974	0.01448716	0.09662293
GNAS-AS1	16.8325659	1.60592963	0.01450319	0.09662293
SFRP1	3574.79057	2.05739227	0.01450251	0.09662293
UHRF2	1652.16208	0.74824174	0.01449284	0.09662293
ZNF215	32.3672383	-1.6890568	0.01449449	0.09662293
DSCR9	14.2750281	-1.1384485	0.01456239	0.09697457
GID8	1500.82094	-0.7871855	0.01456859	0.09697457
TMEM132A	216.08546	1.28063216	0.01459647	0.09711804
CLEC18B	9.41556046	1.63821886	0.0146172	0.09721389
EPN1	1288.96905	-0.5382682	0.01462412	0.09721779
ARMC5	232.198022	0.5164478	0.01468928	0.09760875
C11orf84	240.080172	-0.4447098	0.01470351	0.09766105
KDM3A	1103.2275	-0.8646169	0.01470996	0.09766168
SNORA49	236.872897	-0.9402892	0.0147183	0.09767482
SERTAD4-AS	22.825994	1.50104089	0.01474997	0.09784277
C1orf233	205.518127	1.4585719	0.01477956	0.09792047
TACC3	169.743739	0.67761934	0.01477451	0.09792047
YY1	1242.93644	-0.4409919	0.01478081	0.09792047
DGCR6	64.4855137	1.77015542	0.01480738	0.09801195
GATSL3	309.843265	1.06720081	0.01480666	0.09801195
APOBEC3C	400.852433	-0.9970985	0.01487965	0.09844795
RRBP1	3731.49843	0.84843411	0.01490323	0.09851909
SFRP5	24.5740757	2.0591115	0.01490156	0.09851909
DPP8	1413.61013	-0.5548276	0.01495331	0.09876511
ZNF483	1720.68957	-0.7657396	0.01494704	0.09876511
MYOZ3	360.944425	1.68340402	0.01499852	0.09898249
SYTL5	122.55813	2.03815683	0.0149991	0.09898249
KRCC1	548.880918	0.63093327	0.01501304	0.09898941
TDP2	484.077631	-0.8233533	0.01501097	0.09898941
ALS2CL	363.517502	1.45289365	0.01508045	0.09932724
RAD51B	164.825675	1.03109852	0.01507325	0.09932724
ZNF454	112.799088	1.31669707	0.01508367	0.09932724
ACOT4	32.373463	-1.3314319	0.01510981	0.09945674
LATS1	2190.16082	0.51039083	0.01512899	0.09948971
LOC1019295	17.4111845	1.74858824	0.01514072	0.09948971
PDE4DIP	1939.9562	1.27136074	0.015137	0.09948971
YDJC	67.5933665	-0.8193481	0.01514071	0.09948971
ITGA4	589.604759	0.99915687	0.01515216	0.09949163
RBM47	690.743006	-1.3262454	0.01515397	0.09949163
C17orf96	54.6242839	1.44251727	0.01517677	0.09951057
PCDHB1	17.6111043	-1.8519732	0.01516856	0.09951057
PNMAL2	198.037182	1.63586064	0.0151721	0.09951057
WDR11	2434.69145	0.55240604	0.01518276	0.09951057
ELOVL5	2699.13469	-0.8962891	0.01521771	0.09965458
MYOZ2	10.099699	1.5173269	0.01521195	0.09965458
ETAA1	461.188801	0.48331152	0.01524541	0.09978213

SEMA3F	977.196409	1.45500436	0.01525018	0.09978213
RHOF	71.209181	-1.2713837	0.01526806	0.09982941
TRAPPC10	1930.0163	-0.7044569	0.0152704	0.09982941
LOC1005064[illegible]	56.9642854	1.06706115	0.01527822	0.09983806
LINC00092	95.7486867	1.73452679	0.01533331	0.10015544
CCDC74A	106.229753	1.54841106	0.01538568	0.10045479
RPS27L	1506.26373	-0.9068724	0.01540659	0.10054864
MXRA8	3815.75091	1.29502172	0.01541688	0.10057303
CUX2	14.018235	1.92167028	0.01544836	0.10073565
KCNC3	455.863645	1.45314006	0.0154641	0.10079548
AARS2	811.219712	0.52262375	0.01548653	0.10087164
FAM118B	330.833158	0.52570251	0.01548891	0.10087164
ANP32E	942.797822	-0.688348	0.01551537	0.10089653
NR2C2	2173.26538	0.46535044	0.01551901	0.10089653
PRAP1	15.4491678	-2.0718359	0.01551872	0.10089653
ZNF839	264.049266	0.80709763	0.01550059	0.10089653
MMP14	3977.07157	-0.7500445	0.01554804	0.10099983
ZFHX3	5628.30455	0.80853882	0.01554404	0.10099983
LTBP2	29151.4275	1.85747611	0.01557089	0.10106277
RAB42	33.0890442	-1.6155677	0.01556909	0.10106277
BTBD11	866.409603	-1.7476269	0.01560428	0.10123673
CDK6	3100.9638	1.26868943	0.01561782	0.1012818
METTL6	301.359309	0.7415381	0.01562841	0.10128238
NAA10	439.945596	-0.754463	0.01563109	0.10128238
CCDC33	33.0576739	-2.0858739	0.01565342	0.10134157
RGS18	176.591498	1.38620249	0.01565234	0.10134157
PCDHA6	145.467382	1.74920273	0.01566931	0.1014017
GATM	791.495473	1.47757182	0.01569575	0.10153004
RASA4B	48.6626002	1.04403925	0.01573634	0.10172115
TOX4	1556.9758	-0.5334161	0.01573854	0.10172115
TIMD4	7.86490216	1.96113092	0.01575918	0.10181174
ACSF2	570.246056	1.366591	0.01579762	0.10201717
FAM13A-AS[illegible]	183.381986	0.93008842	0.01582206	0.10208915
MAVS	5753.30707	-0.598594	0.01581669	0.10208915
ATM	6741.2526	0.68674695	0.01583565	0.10209107
LILRB5	28.7387719	-1.8266485	0.01582916	0.10209107
IK	1897.79866	-0.5342209	0.01584637	0.10211734
LTBR	1271.95089	-0.6359384	0.01586045	0.10216523
DEDD	466.058148	-0.3490077	0.01587112	0.10219115
CD40	211.091448	1.20152759	0.01589857	0.10223749
ITM2C	11982.7278	-1.579015	0.01589282	0.10223749
MCTP2	172.04702	-1.4285829	0.01588648	0.10223749
RPS26	2691.9341	-1.3366432	0.01590494	0.10223749
C12orf66	160.193701	-0.755107	0.01593005	0.10231327
SLC44A1	4332.98735	1.20291311	0.01592588	0.10231327
FCF1	593.338651	-0.7710555	0.01595076	0.10240348
SYNE3	281.234203	1.09075426	0.01596692	0.10246442
DOPEY1	1878.06009	0.6332461	0.01599072	0.10257185
ORMDL2	220.119522	-0.8177929	0.01599702	0.10257185
FCGR1C	68.1521114	1.65586233	0.01601397	0.10259494

GART	1239.07743	-0.4878795	0.01600879	0.10259494
C19orf57	12.1631956	1.54923416	0.01605086	0.10271772
MKL2	4997.37727	-1.5636589	0.01604217	0.10271772
MLLT11	187.074025	-1.3039003	0.0160532	0.10271772
CRLS1	578.199048	-0.511174	0.01607147	0.1027427
FAM160A1	316.03964	-1.8518785	0.01607775	0.1027427
PVRL2	2095.43408	0.73747422	0.01609054	0.1027427
S100A1	9.09254122	-2.0662518	0.01608864	0.1027427
UBA6	1275.73413	0.44633366	0.01607392	0.1027427
BRD9	655.226428	-0.4535929	0.01610593	0.10279406
TPD52	645.30231	-1.6659038	0.01611196	0.10279406
CDC25B	2662.35502	-1.1389149	0.01612613	0.10284175
TPT1	42403.0244	-0.766658	0.01614239	0.10290269
USP14	1663.05143	-1.0197467	0.01616853	0.10302662
ALG5	383.169129	-0.688279	0.01620693	0.10305427
LRRC28	212.585104	-0.8816482	0.01621312	0.10305427
MAN2A2	1293.44736	-0.5195656	0.01621064	0.10305427
RWDD3	104.283063	0.77475097	0.01620069	0.10305427
SNX17	1821.61015	0.66052208	0.01621215	0.10305427
TMEM17	70.6345919	0.84211446	0.01618724	0.10305427
MRPL2	177.090076	-0.6768298	0.01623311	0.10313865
BLOC1S3	177.948046	-0.6983773	0.01625442	0.1032034
TOMM40	597.759726	-0.6849334	0.01625674	0.1032034
HNRNPH2	105.534331	-0.7480823	0.01626619	0.10322074
CHRNA5	10.4014375	-1.6073676	0.01630001	0.10335001
IGF2R	8561.32344	-0.9560463	0.01629994	0.10335001
PCDHA1	31.8411443	2.07829748	0.01630679	0.10335031
DUOX1	477.092194	2.03100533	0.01632352	0.10341373
HNRNPUL2	607.720681	-0.5149684	0.01633568	0.10344811
CERS5	714.925278	-0.6745811	0.01636718	0.10360487
AP4E1	971.288443	-0.4555893	0.0163806	0.10363359
SLC3A2	1481.84398	-1.2212533	0.01638521	0.10363359
POU6F2	300.221366	-1.7333269	0.01639521	0.10365416
HLA-DQB1	1605.57182	1.88326164	0.01641477	0.10371821
PCDHA3	222.378067	1.79808545	0.01641884	0.10371821
CDK17	894.151951	-0.7795287	0.01645432	0.1038996
LINC00910	76.2477269	1.34809468	0.01647493	0.103987
RBM12	2736.90808	-0.4998077	0.01649437	0.10406692
PAPOLG	673.901392	0.73285287	0.01650546	0.10409414
FAM180A	299.555183	2.03265527	0.01656366	0.10441832
PRMT5	808.657259	-0.6635385	0.01658462	0.10450757
ZCWPW1	94.0152447	1.03249044	0.01663067	0.10475483
GRIK1-AS1	11.8494554	-1.7347081	0.01664802	0.10482114
HLA-DOA	71.0383656	1.5224654	0.01669188	0.10483945
MYH15	31.1666751	1.36421172	0.01668218	0.10483945
RC3H2	1515.604	-0.4848747	0.01668116	0.10483945
SPG21	1153.49421	-0.5643957	0.01668648	0.10483945
TCFL5	438.190802	-0.8692537	0.01667747	0.10483945
XXYLT1	480.336057	1.08593745	0.01665904	0.10483945
ACOT1	42.7929938	1.20311454	0.01672174	0.10494119

EHF	540.873648	-1.6778039	0.01672052	0.10494119
NAMPT	3514.72856	-1.1875095	0.01676473	0.10516807
VWA9	735.978895	-0.5816762	0.01677418	0.10518435
SNX22	53.2387187	1.56518323	0.01678716	0.10522282
TP53I3	907.390026	1.29976265	0.01680178	0.10527149
FNDC3A	6051.09251	-0.5578392	0.0168504	0.10553312
SETD6	257.202558	0.56230975	0.01686057	0.10555375
PPFIA4	89.4711563	-1.7028656	0.01686967	0.10556774
GPR141	26.2314249	1.52304794	0.0169018	0.10568271
PWAR5	1358.37091	-0.5937338	0.01689992	0.10568271
EIF1B-AS1	37.3126507	1.31234195	0.01691515	0.10568412
LRMP	174.021413	1.11648576	0.01691579	0.10568412
AOAH	509.185123	1.3757084	0.0169432	0.10570146
BREA2	8.78559692	1.84894411	0.01696103	0.10570146
CACNA1I	9.91047336	-1.8016283	0.01693881	0.10570146
HAX1	882.017238	-0.6112323	0.01696673	0.10570146
HSPA4	2590.97852	-0.5819275	0.01695296	0.10570146
NUPR1	1106.16186	-1.1376048	0.01692795	0.10570146
SOSTDC1	13.9434895	1.81422885	0.01696234	0.10570146
BDKRB2	134.955287	-1.9468303	0.01697814	0.10572972
ENHO	11.6142473	1.9405266	0.0169965	0.10576162
KCNK1	479.412827	-1.9346367	0.01700392	0.10576162
MAP2K6	82.900999	1.36994925	0.01699759	0.10576162
DMXL2	3817.76808	-0.4863934	0.01704845	0.10588897
DPY19L2P3	10.1458381	1.53005809	0.01703751	0.10588897
LINC00641	837.90753	-0.7200326	0.01706575	0.10588897
NSUN5	279.53479	0.6092636	0.01705853	0.10588897
SYT7	30.9178799	1.83784035	0.01706456	0.10588897
ZBTB7A	663.511334	-0.5826743	0.01704094	0.10588897
C17orf97	81.6819002	1.08887299	0.01707649	0.10591282
MDM2	5765.39809	-0.694917	0.01708772	0.10593972
ZMYM1	444.207137	0.67605569	0.01715986	0.10634404
HOXC5	44.3187696	2.02352453	0.01718608	0.10644256
SYNPO2	5190.59795	1.28389608	0.01718961	0.10644256
CXorf40A	174.664401	-0.5767984	0.01722004	0.10658805
PRKAG2	2172.86576	-1.5282675	0.01726128	0.10680028
LGALS12	8.18871924	1.95141997	0.01728803	0.1069227
PIGK	854.409065	0.63263381	0.0173273	0.10712248
ERMN	12.9872297	1.72516332	0.01737509	0.10736826
IGIP	784.17666	0.6052829	0.01738803	0.10736826
LOC1001316	18.784508	-1.1498159	0.01738453	0.10736826
KLHL2	487.771184	-0.8919342	0.0174525	0.10767985
8-Sep	2133.98683	0.6110067	0.01744876	0.10767985
TXN	846.371818	-1.0094064	0.01750964	0.10798902
JMJD7	107.846653	0.85521992	0.01752194	0.10802147
CDS1	103.888239	-1.9242626	0.0175424	0.10806088
LOC1005066	13.2684915	2.00799415	0.01753555	0.10806088
NAALADL2	266.984283	0.72338814	0.01759697	0.10835364
WNT3	96.4606262	1.32765802	0.01761187	0.10840193
CHERP	683.424544	-0.6057977	0.01764409	0.10846982

DEPDC7	24.0413126	1.60189114	0.01763876	0.10846982
MRPL46	221.377496	-0.7161815	0.01763686	0.10846982
LOC646762	497.010417	1.07590512	0.0176666	0.10856477
PCID2	527.407679	-0.4088917	0.01773482	0.10894044
ERC1	3851.99036	-0.6210651	0.01775804	0.10903949
CCL26	189.646996	1.10963784	0.01777462	0.10905946
CYB5R3	2719.15546	-1.1403605	0.01777549	0.10905946
ADAM11	86.026018	1.15269996	0.01781334	0.10924806
PIK3R1	9249.4693	1.57791783	0.01785359	0.10945121
ARHGAP4	751.482319	1.08203053	0.01788838	0.10962074
MTFR2	15.1893852	-1.3731714	0.01789645	0.10962647
IFITM2	2510.02436	-1.2891174	0.01791635	0.10970461
TFDP2	2285.25676	0.79561033	0.01795102	0.10987313
CASKIN2	663.233224	0.83414167	0.01796948	0.10989855
GRB2	2589.78353	-0.3891851	0.01796617	0.10989855
SQSTM1	7666.0648	-1.1690539	0.01798788	0.1099673
IFNLR1	175.393114	1.04443762	0.01799757	0.10998274
AP3D1	3310.86145	-0.4681104	0.01802968	0.11005482
ARHGAP15	265.331212	1.21514322	0.01805284	0.11005482
BBS7	412.97689	-0.4701811	0.0180534	0.11005482
FN3K	133.264408	-1.0578053	0.01805015	0.11005482
MYO6	2719.55523	-1.1110508	0.01804499	0.11005482
PEX3	351.053285	0.40009391	0.01802264	0.11005482
PTPLAD2	216.547108	0.87297175	0.01806667	0.11005482
SH3BGRL	3162.66668	-0.596096	0.01806193	0.11005482
SMAP2	1774.2016	1.1408364	0.01808407	0.11011716
PHYH	214.523878	-0.754981	0.01809749	0.1101552
CHST5	7.71934591	1.79241923	0.01811461	0.11021576
LURAP1L	111.452853	1.56431027	0.01817958	0.11056723
RHBDF1	1052.7142	0.64823831	0.01820524	0.11067948
RNF185	964.344038	-0.8888547	0.01824765	0.11089344
FSTL3	2247.52079	-1.2326567	0.01829151	0.11111605
PRKD1	208.017319	1.61038654	0.01836032	0.11148997
PFN4	9.9336059	1.61453059	0.01837	0.11150468
TFG	1448.37134	-0.5357984	0.0183895	0.11157896
PAXBP1-AS1	99.8745753	0.94829615	0.01840841	0.11164961
CYP4F11	9.82463686	-2.0199488	0.01843712	0.11177958
OSGIN2	2376.84036	0.88036096	0.01846878	0.11188324
RCE1	142.817978	-0.6507166	0.01846374	0.11188324
DPH6-AS1	37.5492873	1.01575212	0.01848398	0.11193118
CTDSP2	5025.10553	-0.5120022	0.01852116	0.11211212
CERS4	340.247255	-0.6147702	0.0185604	0.11217286
LOC90246	56.6982056	1.88807702	0.01855721	0.11217286
SARNP	595.277205	-0.5701597	0.01855412	0.11217286
SFMBT2	1239.82318	0.96470721	0.01855854	0.11217286
MAP4K3	1020.86494	0.49814277	0.01857804	0.11223532
SMC5-AS1	28.2710094	1.00976856	0.01860254	0.11233915
LUC7L3	3725.61972	0.45491726	0.0186143	0.11236603
DBX2	21.6687839	2.02439374	0.01862909	0.11241112
ARHGEF40	1694.65917	0.77974226	0.01866896	0.11260746

HPSE	134.996763	-1.0976776	0.01871482	0.11261879
IFT140	886.237799	0.64960448	0.01868854	0.11261879
LTC4S	80.2623746	1.33421144	0.01870616	0.11261879
MEF2C-AS1	43.289974	1.62919659	0.0187035	0.11261879
PGAM5	423.959923	-0.6166309	0.01868824	0.11261879
ZNF500	454.086388	0.4432467	0.01871026	0.11261879
CPT1C	189.721343	1.60775807	0.01874691	0.11276774
CREM	828.869875	-1.289903	0.01876002	0.11280242
CCT5	2218.4658	-0.6485792	0.01884831	0.11328896
LOC1001314	13.7365415	1.40674486	0.01887072	0.11337931
METRN	212.652664	-0.6436682	0.01890771	0.11355711
HDDC2	679.767983	-0.451817	0.01892303	0.1136047
UFD1L	384.196071	-0.8524157	0.01895195	0.11373389
CPNE5	162.797174	1.8184114	0.0189646	0.11375064
TBX3	13.8467209	1.71573871	0.01896955	0.11375064
ZNF467	127.555114	-1.1121025	0.01901136	0.1139569
ZNF281	530.446291	-0.6445603	0.01904293	0.11410162
PSME3	1234.65237	-0.4738825	0.01906683	0.1142003
MYRF	13.8805263	-1.871659	0.01908123	0.11424196
PDK4	3017.45621	1.29599157	0.0190913	0.11425778
KIF26A	62.5516883	1.60588035	0.01912293	0.11440246
ABHD17A	596.034471	-0.5973279	0.01914148	0.11446888
FHL2	325.489207	-1.1915776	0.0191971	0.11471217
MACROD1	70.0647387	1.44468703	0.01919114	0.11471217
SOX4	752.516269	0.7100111	0.01924553	0.11491221
TLX1	36.9990974	2.02633139	0.01924329	0.11491221
NDUFA7	401.830626	-0.9387338	0.0192782	0.11506252
AGPAT3	1901.26566	-0.5958718	0.019309	0.1151998
KCNN3	288.808842	1.37752929	0.01931619	0.1151998
ALDH5A1	567.463857	0.71374709	0.0193341	0.11521057
CENPBD1P1	556.649701	-0.6349487	0.0193405	0.11521057
MAP2K3	383.99023	-0.970713	0.01933468	0.11521057
NUPL1	1102.44636	-0.5115862	0.01937055	0.11530018
SCML1	406.650886	-0.909573	0.01936722	0.11530018
CITED1	13.689559	-1.7115134	0.01938885	0.11534154
PTPN11	3322.92375	-0.562739	0.01939251	0.11534154
FASTKD5	419.689756	-0.4919547	0.01941145	0.11537161
ZNRF2	532.660344	0.89794022	0.01941259	0.11537161
EFTUD1P1	62.9796778	1.5865222	0.01947272	0.1156395
SF1	2775.04122	-0.6585256	0.01946633	0.1156395
TEX30	65.8379584	-0.7553945	0.01949491	0.11572654
SKP1	5469.55388	-0.930777	0.01952006	0.11583109
UBE2Z	2031.84941	-0.4271929	0.0195323	0.11585895
CC2D2A	663.799573	1.18381506	0.01960177	0.11622615
GIGYF1	2068.44673	0.68341497	0.01963132	0.11635647
GRIN2A	25.1614497	1.89327188	0.01965418	0.11644706
EVA1C	452.280783	-1.3599373	0.0196715	0.1164598
MFI2	44.2989944	-1.7873198	0.01966804	0.1164598
NFATC2	573.221371	1.00551549	0.01970786	0.11658522
NR3C1	3844.87331	0.76394753	0.01970193	0.11658522

COL25A1	68.2787325	1.06519062	0.01979154	0.11703521
DSCAM	13.9387484	1.79908382	0.01980555	0.11707299
VAMP8	601.872133	-0.9726402	0.01981681	0.11709451
XRCC1	501.393128	-0.4176425	0.0198296	0.117125
FBXL15	136.1906	-0.6950275	0.01987336	0.1173022
KLHL15	1113.89444	-0.7459721	0.01987487	0.1173022
ATP6V1G1	1495.64592	-0.5399695	0.01989637	0.11733897
MOCS3	236.595867	-0.6519233	0.01989429	0.11733897
ATP11A	1468.20036	-0.6392185	0.0199224	0.11737698
CECR6	82.5748514	1.37317465	0.01992302	0.11737698
IL18R1	133.953888	-1.2986308	0.01992574	0.11737698
ID1	3973.05023	-1.3242092	0.01995869	0.11748095
SNORA80A	48.2302411	1.54607785	0.01995558	0.11748095
ITPRIPL2	2001.03947	0.58134124	0.0200035	0.11769962
MS4A14	160.118002	1.55485524	0.02001425	0.11771779
NME3	350.275842	0.87411411	0.02002687	0.11774697
PCDHB15	188.727812	-1.5034631	0.02003796	0.11776711
PCNA	944.184213	-0.5635874	0.02006256	0.11786657
GDF11	356.718966	-1.0722812	0.02007296	0.11788258
FER1L5	8.02496467	1.98104454	0.0201091	0.11804973
LINC00896	9.30797463	1.71060673	0.02012819	0.11811663
DLEC1	225.616878	1.17392259	0.02018387	0.11839815
IRAK1BP1	280.890285	0.54850635	0.02021555	0.11853875
ADCK2	317.435371	0.88709847	0.0202644	0.11873453
PIGA	265.224778	-0.9192372	0.02025952	0.11873453
TSPAN7	2354.94957	1.60699835	0.02027828	0.11877055
ETV3	334.058149	-0.6475315	0.0202906	0.11879745
LINC00339	123.558741	0.83326347	0.02030509	0.11883699
GSPT2	320.7114	-0.7948216	0.02034706	0.11903728
C8orf59	273.969947	-0.4817147	0.02035993	0.11904473
EOMES	12.2105962	1.6671695	0.02036907	0.11904473
PDZD2	4719.03343	-0.8273532	0.02037158	0.11904473
PEAR1	1929.52453	1.16444441	0.02038468	0.11907598
DCT	18.0642221	1.39255678	0.02040212	0.11908729
LINC01094	247.813082	1.28444239	0.0204017	0.11908729
PEX5	844.333753	-0.7474566	0.02045277	0.11933758
ARID3A	196.653584	-1.3133872	0.02046278	0.11935065
C19orf38	37.6001323	1.42979946	0.02052483	0.11966717
SSBP4	732.352267	-0.4997248	0.02055082	0.11977322
CLASRP	589.316502	-0.5015486	0.02061995	0.12013054
GIT1	1190.51284	-0.5587383	0.02066395	0.12034126
LOC728989	9.00906843	1.83626312	0.02067327	0.1203499
CDK14	1055.09592	0.87282437	0.02069282	0.12041808
LOC1006309	30.7858373	1.06405456	0.02071002	0.12047256
ATP5O	1445.62674	-0.4937834	0.02072848	0.12048868
CLDN7	113.651351	-1.4776841	0.02072763	0.12048868
TLR2	1660.41209	1.12093991	0.02074071	0.12051421
AIM1	298.416953	1.13043923	0.02080081	0.12071825
BTBD8	49.3135939	1.07267731	0.02083853	0.12071825
DDO	9.42759252	1.59311243	0.02080304	0.12071825

FOXN2	824.692355	0.93207581	0.02082657	0.12071825
MRPL45P2	155.517565	0.73183662	0.02082981	0.12071825
MRPS34	400.221751	-0.4725145	0.02082959	0.12071825
PQBP1	446.6609	-0.6273778	0.02083869	0.12071825
TOMM20	3886.12541	-0.6383753	0.02078712	0.12071825
ZYX	2485.40218	-0.9632537	0.02086497	0.12082493
HSD17B11	690.713101	0.53321545	0.02090402	0.12100545
SLC46A1	775.015762	0.73417825	0.02097409	0.12136533
SRSF6	4121.44036	-0.583649	0.02099002	0.12141175
ZDHHC14	233.822705	1.11422241	0.02103052	0.12160027
CR1	52.3144431	-1.7778609	0.02105129	0.12167454
TMEM176A	36.7650852	-1.8857731	0.02106241	0.12169303
CEP112	637.845312	0.61560231	0.02107479	0.12171879
TMOD2	1530.19209	0.89081621	0.02108587	0.12173704
PWARSN	615.541864	-0.5819484	0.0211082	0.12182017
TLR1	686.664866	0.74060611	0.02112503	0.12187151
DLST	1366.77443	-0.6171577	0.0211349	0.12188271
VPS13C	12548.8627	-0.5426361	0.02114684	0.12190579
CIAPIN1	443.696344	0.53904216	0.02117915	0.12190908
SH2B1	1529.28021	0.6897217	0.02117165	0.12190908
TOE1	184.727572	-0.8821513	0.02115676	0.12190908
ZNF497	39.2820174	1.04828217	0.02117057	0.12190908
DAK	383.574353	0.6943846	0.0211991	0.12197821
ZIC5	854.836868	-0.6587822	0.02125373	0.12224675
TLR3	245.245564	0.96990856	0.02129948	0.12246403
DCK	486.320298	0.75708412	0.02131535	0.12247776
NR1D2	2157.02958	1.11440481	0.02131781	0.12247776
EGLN2	51.6995939	-0.7677047	0.02135784	0.12266188
FHOD1	639.581875	0.6066108	0.02136834	0.1226763
CDKN2D	59.3914556	-1.3393759	0.02138456	0.12269357
HECTD2	1082.06545	0.9450951	0.02138732	0.12269357
SCN3B	23.603901	-1.5682688	0.02146475	0.12309182
RABGEF1	998.948563	0.59410372	0.02148533	0.12316384
DUXAP10	9.42375137	1.83287862	0.021544	0.12340806
LAPTM4B	1486.89941	0.75350745	0.02153924	0.12340806
CIPC	764.344093	-0.5758269	0.0215901	0.12362606
LOC115110	25.1479646	1.46197733	0.02166651	0.1239712
SPG11	3813.22054	-0.3696145	0.02166417	0.1239712
TMEM104	543.636565	0.67520672	0.02169346	0.12398685
WFDC2	189.003228	-1.9101888	0.02167863	0.12398685
ZNF425	83.0097957	0.87835728	0.02169053	0.12398685
SALL1	115.825257	1.8887022	0.02170995	0.12403496
CCNI2	17.7555641	1.8582248	0.02172648	0.12408323
C6orf106	1906.79119	-0.5228805	0.02174348	0.12408819
TSPAN18	2856.54256	1.36761756	0.0217435	0.12408819
REXO1	578.781685	-0.5806988	0.02176901	0.12418764
CAMK2B	35.5629754	1.60429613	0.02179834	0.12426268
GAB2	1650.22528	0.98044959	0.02179637	0.12426268
AQP7	8.92842776	1.90491005	0.02183394	0.12441942
PWP1	901.796006	-0.5774654	0.02190457	0.12477562

SPIN4	368.573605	-0.5558366	0.02192378	0.12483876
ATOX1	356.580761	-0.697742	0.02196322	0.12500931
PSMD8	1438.7454	-0.7032121	0.02199441	0.12500931
RBL2	2889.05524	0.61838344	0.02197948	0.12500931
SSH3	824.549443	-0.8068205	0.02198718	0.12500931
TRNT1	331.810615	0.61394298	0.02198486	0.12500931
HS3ST3A1	39.6003199	1.861652	0.02201574	0.12505935
THEM6	258.966103	0.59872673	0.0220195	0.12505935
PIM3	285.950883	-1.511401	0.02203155	0.12508153
C8orf88	518.812246	-1.2137942	0.02204832	0.12508431
ENG	2770.83337	1.3916553	0.02204114	0.12508431
PKM	13319.3204	-1.1638832	0.02205648	0.1250844
KIAA0232	2116.99174	-0.4481553	0.0220772	0.1251557
FKBP10	3195.6136	0.74870077	0.02208743	0.12516753
ACTR8	623.190948	0.76840086	0.02212492	0.12533375
TMEM54	174.250765	-0.8724196	0.02213351	0.12533621
NCK1	942.179875	-0.4813067	0.02217008	0.12540404
PDGFB	593.865306	0.97805625	0.02216775	0.12540404
PIAS4	213.533252	-0.5523336	0.02217971	0.12540404
SLC25A10	62.039775	1.20427864	0.0221863	0.12540404
SLC25A12	792.261022	0.71799535	0.02217315	0.12540404
TCF12	3965.01621	-0.7402444	0.02219939	0.12543184
WIBG	292.132532	-0.4464871	0.02231957	0.12606453
ANXA2R	44.8324692	1.24547107	0.02234239	0.12608652
FIP1L1	815.318031	-0.4283037	0.02235629	0.12608652
TUBA8	31.7435341	-1.4270818	0.02235248	0.12608652
VPS9D1	318.293945	0.69126841	0.02234127	0.12608652
GIPC1	1329.16319	-0.6665048	0.02239298	0.12624713
SRGAP3	659.409658	1.056463	0.02241973	0.12635157
TFCP2	800.420015	-0.800481	0.02251572	0.12679949
ZNHIT3	296.710699	-0.372159	0.02251382	0.12679949
COX17	280.53882	-0.8425201	0.02253584	0.12682916
PFDN5	2944.52114	-0.5144822	0.0225375	0.12682916
UTP14A	393.937705	-0.5082198	0.02259695	0.12711715
HYOU1	2976.14462	-0.9878136	0.02263557	0.1272424
JRKL	449.432952	-0.4196587	0.02263578	0.1272424
WBP1	49.3830991	0.92259302	0.02266975	0.12738673
ALX4	1868.9832	1.90461895	0.02268253	0.12739474
ARL5B	580.821897	-0.5715279	0.02269152	0.12739474
TMCO3	1926.67537	-0.9296205	0.02269605	0.12739474
ASAH1	2504.37484	-0.6463629	0.02272005	0.12748288
LHFPL2	8301.58937	-1.1822467	0.02273841	0.12753913
STK11	1118.07542	-0.723061	0.02275498	0.12753913
WAC	3235.4984	-0.3587422	0.02275275	0.12753913
MTIF3	514.26367	-0.6301333	0.02280791	0.12778918
PRUNE	483.264483	-0.5331477	0.02285577	0.12796399
UBA52	4688.75229	-0.4432451	0.02284765	0.12796399
LINC00565	12.3973841	1.74358719	0.02286927	0.12797155
LOC1019278	22.8022233	1.30911455	0.02287378	0.12797155
ITGAL	294.782419	1.32561577	0.02291569	0.12808761

MGST3	891.087767	-0.7049963	0.02291326	0.12808761
PDZK1IP1	21.4435927	1.95036321	0.02291954	0.12808761
IFITM3	6019.1022	-1.1319154	0.02292918	0.12809489
BDH1	138.911621	0.91585194	0.02301117	0.12850624
TSR2	634.620133	-0.5975392	0.02302207	0.12852038
ATXN7L2	112.292685	1.03100148	0.02308011	0.12875079
PDLIM1	680.709738	-1.5875243	0.02307821	0.12875079
FAM168A	2566.74567	0.64749927	0.02314208	0.12900425
HSP90B2P	57.6637585	-1.1488154	0.02314234	0.12900425
AKAP11	6561.21252	-0.5787646	0.02315552	0.12903091
CALML3-AS1	15.4253419	-1.5275439	0.02320019	0.12923295
CALB2	11.3490871	1.95490229	0.02323676	0.12938786
PEAK1	4941.0167	1.13670197	0.02324484	0.12938786
LOC1019270	64.1227167	1.20433179	0.02326197	0.12943631
CHCHD6	281.602159	1.48326592	0.02330787	0.12959782
SLC4A3	163.448869	1.39855566	0.02330205	0.12959782
FFAR4	17.6179089	1.20320489	0.02336993	0.12978952
KCNQ3	953.035981	1.29155846	0.02336397	0.12978952
RNF114	1213.72557	-0.4361412	0.02336607	0.12978952
TICAM1	179.148551	-0.6927462	0.02337614	0.12978952
PSMC5	1008.62949	-0.5582222	0.02339272	0.12983468
SQLE	352.14097	-1.0066035	0.02340189	0.12983867
C19orf70	316.696641	-0.5643461	0.02341741	0.12987784
PPP3CA	1317.07188	-0.8208592	0.02343088	0.12990564
AAGAB	908.703384	-0.6952915	0.02344506	0.1299373
NIPBL	4625.2708	-0.5973747	0.0234535	0.1299373
CUL4A	1622.43037	-0.5048356	0.02350292	0.13016414
USP39	620.45917	-0.4011119	0.02356029	0.13043486
RPL35	4103.24173	-0.4387824	0.0235804	0.13049916
SUGT1	713.16271	-0.550977	0.0235954	0.13053515
ANKHD1-EIF4	18.7212656	-1.2278164	0.02364241	0.13074815
ZNF783	665.479949	0.52834885	0.02378988	0.13151634
TAF1D	471.148022	-0.7482337	0.02383675	0.13172806
CELF1	3749.60589	-0.5635611	0.0238865	0.13195554
ALMS1P	9.78709306	1.61005133	0.02390761	0.13202469
LOC730183	14.4564247	1.35933555	0.02396067	0.13227011
RAB5C	1804.67373	-0.6153578	0.0239843	0.132353
HECTD4	6258.34684	-0.3121226	0.02401885	0.1324485
MRPS25	1200.42824	0.78484798	0.02401826	0.1324485
SMPD1	904.05969	0.75705976	0.02405175	0.13258234
CDS2	3323.69048	-0.7365326	0.02409629	0.13278025
PPP1R26	422.070204	0.48001398	0.02418136	0.13320125
AQP1	5179.83148	1.82852963	0.02421355	0.13323521
LOC1019292	11.0181927	1.4352326	0.02420353	0.13323521
PGM1	1113.91169	1.10912918	0.02420885	0.13323521
NLGN4Y	168.816896	-1.7782691	0.02444037	0.13438706
THBD	425.204917	-1.6075592	0.02443579	0.13438706
CRAMP1L	1393.45813	0.69226922	0.02445517	0.13442029
KRI1	480.759919	-0.5576761	0.02448136	0.13443446
SLC7A9	8.64505719	1.51714066	0.024484	0.13443446

USP13	817.639962	-0.6949208	0.02446925	0.13443446
GPR162	331.808211	1.19543425	0.02454287	0.1347096
LGALS1	2694.98225	-0.9119489	0.0245582	0.13474558
CIR1	567.979129	-0.3984864	0.02457421	0.13475567
FIG4	473.186497	0.62568289	0.02460967	0.13475567
PARP9	1054.50057	-0.5040678	0.02460653	0.13475567
POLR1D	734.603778	-0.4123915	0.02459869	0.13475567
SCN4A	41.4964585	1.61877544	0.02461267	0.13475567
USP11	2374.40606	-0.4644109	0.02458836	0.13475567
CYTL1	73.2028882	1.30248743	0.02468475	0.13505406
TWIST2	64.965032	-1.5432129	0.02467648	0.13505406
EIF1B	295.84096	-0.8001429	0.02469604	0.13506775
SOCS4	1253.60324	0.79542186	0.02476442	0.13539352
HDC	23.7486109	1.58544064	0.02477377	0.13539648
GRAMD1A	1107.92437	-0.6764896	0.02479506	0.13546462
FAM210A	400.577375	-0.6449817	0.02484559	0.13567263
TXNL1	741.434014	-0.6572734	0.0248508	0.13567263
LAS1L	640.429954	-0.5293885	0.02486943	0.13572613
DUSP6	1601.02636	-1.1305003	0.02490271	0.13581128
UNC119B	1094.70057	-0.584868	0.02489681	0.13581128
BIRC2	1821.4047	0.36972935	0.02493723	0.13587578
ITGA9-AS1	13.9395909	1.20294873	0.02494531	0.13587578
LINC00222	12.2590905	1.50849684	0.02494321	0.13587578
MGC16142	29.6668718	0.95549318	0.02495711	0.13587578
NUDT16P1	86.1828391	1.25966242	0.02495876	0.13587578
IGSF3	606.454895	1.21236251	0.02499621	0.13603145
CHST7	163.474418	1.52645501	0.02500713	0.13604268
BEND7	151.407877	-0.8301409	0.02502066	0.13606811
PRR11	94.9431878	0.68975287	0.02503246	0.13608411
NAPB	584.088491	-0.5594754	0.02511728	0.13649693
CHD2	5245.37835	-0.3512035	0.02522311	0.13688046
CYP4F35P	170.04847	1.92944327	0.02523437	0.13688046
INHBA	73.5082307	1.50966179	0.02521056	0.13688046
MMP17	139.691634	1.66702709	0.02523612	0.13688046
NOVA1	340.676401	-1.438718	0.02521873	0.13688046
RAB28	363.818196	0.36764787	0.02524132	0.13688046
EIF6	906.779519	-0.6003719	0.02526126	0.13694029
GNAI1	237.277063	0.83198347	0.02532241	0.13722334
CCDC151	29.0256241	-1.4890374	0.02533401	0.13723778
PAQR4	53.9711874	0.88211081	0.02539263	0.13750685
CCT6B	66.2303661	0.91117774	0.02544907	0.13775218
MEF2A	4051.88562	0.62254951	0.02545586	0.13775218
GTPBP4	658.294923	-0.591871	0.02549988	0.13785539
TMEM248	4145.56495	0.50475242	0.02549623	0.13785539
TTC39B	524.037279	0.75087169	0.02550186	0.13785539
CPNE2	284.525791	0.90339463	0.02552581	0.13793633
PPARGC1A	1310.58229	1.55299608	0.0255393	0.1379607
S100A16	1172.00941	-1.1394498	0.02556843	0.13806952
FARS2	308.171871	0.569961	0.02565078	0.13846557
IL12RB1	63.6630678	1.26176827	0.02570601	0.13871496

SCN2B	477.14748	1.81299843	0.02575838	0.13894873
ALB	22.2603861	-1.5947434	0.02577108	0.13896555
CBX3P2	48.2902561	1.25080696	0.02580146	0.13896555
FEM1B	2844.80358	-0.6075755	0.02581577	0.13896555
KRTAP5-7	10.6380022	-1.3257052	0.02578456	0.13896555
MIOS	599.056645	0.36062398	0.02580812	0.13896555
PSMA6	975.505719	-0.4745068	0.02581113	0.13896555
ME2	1083.97426	-0.8021605	0.02589615	0.13934943
LINC00311	12.6824216	1.80885043	0.02601113	0.13991911
HIST2H2AC	661.757307	-0.5646789	0.02608446	0.14023398
ZNF433	203.416717	-0.9217537	0.02608792	0.14023398
SAP18	2170.33789	-0.7661203	0.02611545	0.14033285
NPIPB3	248.203671	-0.8870136	0.02615059	0.14047257
CDC42SE2	1161.35795	-0.6061718	0.02616214	0.1404855
CCDC86	204.106214	-0.8451507	0.02617303	0.14049486
TNFSF9	17.953004	-1.6991761	0.0262229	0.1407134
MXRA5	3509.22913	1.65456395	0.02625387	0.1408304
CLASP2	1723.49202	0.71944771	0.02630847	0.14106118
TRIP6	1375.72532	0.38734034	0.02631526	0.14106118
MC1R	117.118705	1.22310704	0.02632497	0.14106402
ELOVL7	115.169871	-1.8009454	0.02634619	0.1411285
ARHGAP22	453.420211	1.50270231	0.02638785	0.14126187
FAM114A1	2026.94714	0.68843677	0.02638948	0.14126187
ZNF511	202.232809	-0.5508103	0.02641882	0.14136965
FCER1A	50.9246948	1.79704336	0.02647933	0.14164412
RBM39	5739.08905	-0.3967513	0.02657225	0.14209173
RTF1	1308.87524	-0.4602937	0.02659845	0.1421823
DDX56	1000.75019	0.54203149	0.02662475	0.14222392
ZNF662	560.689077	0.73673681	0.02661898	0.14222392
ZFYVE19	289.020374	-0.529427	0.02674511	0.14281721
GLT1D1	127.653823	-1.3936447	0.02675824	0.14283768
FAM53B	1805.97046	0.61995069	0.02680734	0.14285161
GLCE	3680.98538	-1.1612153	0.02679011	0.14285161
MAT2A	2669.60473	-0.4692981	0.02680471	0.14285161
NEIL1	238.690493	1.0080532	0.026783	0.14285161
PARVA	3665.42567	0.91412333	0.02678374	0.14285161
MCEE	99.044786	1.12439299	0.02682681	0.14290579
LINC00663	151.952876	0.71019804	0.02687666	0.14297302
LINC01001	15.8492917	-1.4325521	0.02685988	0.14297302
NR4A1	3384.80128	-1.7562049	0.02687595	0.14297302
SNRPC	551.335576	-0.6845339	0.02687573	0.14297302
DTX3L	1426.49876	-0.4456164	0.02690317	0.1430645
CLEC11A	423.450409	1.5115196	0.02695042	0.14316419
DNAH1	1874.31632	0.77518855	0.02695686	0.14316419
HIP1	1506.71176	1.02326728	0.02694778	0.14316419
PPM1B	1267.85638	0.48210653	0.02695919	0.14316419
CHORDC1	477.2183	-0.4801034	0.02704651	0.14357828
MIB1	3815.40341	-0.7135999	0.02708989	0.14362288
MMP24-AS1	551.860775	-0.6336878	0.02709164	0.14362288
SLIRP	283.639586	-0.6617757	0.02709231	0.14362288

TBC1D22A	676.028481	-0.3934981	0.02707324	0.14362288
IQGAP1	10129.1257	-0.5802671	0.02715435	0.14390211
PAK2	2472.92209	-0.4097508	0.02720361	0.14411348
TRPV1	292.989097	0.63164928	0.02723705	0.1442409
TSPYL2	3746.62122	-0.703477	0.02725095	0.14426476
ACTN4	5699.94912	-0.5813057	0.02727964	0.14436688
BCR	1827.40284	-1.1067623	0.02733728	0.14452821
GSTP1	4059.30923	-0.4363979	0.02733042	0.14452821
OSBPL7	362.822408	0.44376701	0.02733834	0.14452821
TMEM102	33.5136755	1.42538828	0.02735549	0.1445691
SLC16A1-AS1	91.9476878	0.83982228	0.02739869	0.14474762
SGK494	171.706433	0.84950059	0.02745125	0.14494863
SLC29A3	153.278382	1.18648064	0.02745561	0.14494863
CASZ1	131.488619	-1.3014275	0.02747904	0.14495046
FLJ27354	11.6726423	1.4775544	0.02748261	0.14495046
SHCBP1	63.6298373	1.21087751	0.02748426	0.14495046
EXTL2	1145.85548	0.86662326	0.02755932	0.14529643
ATP5SL	327.197142	-0.5893054	0.02760999	0.14546374
IDS	3860.11925	-0.7821205	0.02760726	0.14546374
APEX1	1426.67182	-0.8267287	0.02764727	0.14553122
IST1	2464.28215	0.51265423	0.02765316	0.14553122
SRL	63.0323175	1.63960403	0.02766069	0.14553122
SURF2	78.9717721	-0.9550634	0.02764133	0.14553122
SFXN4	224.195159	0.56750824	0.02771213	0.14575198
C16orf96	11.5186669	1.191441	0.02777597	0.14593783
PLS1	335.366284	1.65755908	0.02777056	0.14593783
TMEM86A	316.48622	0.74826623	0.02776149	0.14593783
SDCBP	5652.13052	-0.7030466	0.02781756	0.14610636
CMAS	560.037334	-0.8677175	0.02784944	0.14622386
NGEF	71.0529958	-0.9942624	0.02788881	0.14633055
THAP3	157.831466	0.6654799	0.02788109	0.14633055
ANKRD11	2542.36676	0.67487229	0.02793286	0.14636332
ANKRD44	1932.83318	0.98757896	0.02790866	0.14636332
KIAA1045	23.0139191	1.63131932	0.02794269	0.14636332
MBTPS2	664.534636	-0.7776852	0.02794202	0.14636332
SHISA4	304.332294	-0.8935187	0.02793964	0.14636332
TMEM109	1905.23299	0.37904257	0.02796081	0.1464083
AK1	629.663056	1.09485287	0.02798247	0.1464718
MIR4697HG	170.13201	-1.1390666	0.02799645	0.14649506
SPRYD3	1398.4397	-0.8030437	0.02804056	0.14667118
TBCA	869.938324	-0.8493696	0.0280492	0.14667118
FDFT1	942.465847	-0.6504186	0.02807131	0.14673684
TCAIM	667.72516	0.5313928	0.02808871	0.14677786
LOC1005075	11.2144095	1.42974325	0.02809984	0.14678608
NDUFA11	879.827818	-0.5792592	0.02812081	0.14683177
ORC4	787.849396	-0.5683095	0.0281277	0.14683177
JPH3	7.38489561	-1.8949926	0.02814286	0.14686102
GNA14	101.302749	1.13277087	0.02818098	0.14696007
SKIL	4304.45716	0.50098727	0.02818071	0.14696007
RCHY1	476.71014	-0.4214584	0.0282049	0.1470349

NBPF15	591.560426	0.62281428	0.02822799	0.14704269
NDUFV1	1552.3066	0.461405	0.02821728	0.14704269
ODF3B	40.82419	-1.4445416	0.02823511	0.14704269
ZC3H12D	153.194264	1.13558249	0.028245	0.14704434
FOXO3	4768.73675	0.88250927	0.02826683	0.14705837
LOC1005064	222.631561	1.73634506	0.02826331	0.14705837
PCNX	3966.41786	-0.514653	0.02829164	0.14713761
CNOT11	634.430964	-0.3414221	0.02830445	0.1471544
PLEKHF1	192.980432	-1.5814329	0.02835047	0.14724814
POLR2J4	296.747935	0.62081931	0.02835123	0.14724814
SLC22A4	74.4005878	0.96506964	0.02833709	0.14724814
PAIP2	1644.32646	-0.6345945	0.02837173	0.14730478
DUSP16	1118.44004	-0.765786	0.02838211	0.1473089
KCNMB1	62.2678567	1.28061158	0.02839661	0.1473344
ADAM33	1224.0868	-1.539748	0.0284395	0.1474573
PTPRA	2309.53668	-0.6148792	0.02843414	0.1474573
LEMD2	948.136256	0.43588837	0.02845772	0.14750203
COX10-AS1	142.205838	0.77253286	0.02846808	0.14750596
SEC16B	218.555823	0.91405423	0.02852062	0.14768611
TRMT1L	806.713093	-0.5852196	0.02852208	0.14768611
LOC1001291	8.4509222	-1.8845897	0.0285626	0.1478461
LOC339803	182.308129	0.54159549	0.02863497	0.14812087
SYPL2	63.1913027	1.70316196	0.02862889	0.14812087
CTR9	1415.2306	-0.5467562	0.02868251	0.14829534
HIST1H1E	1941.00358	0.51309089	0.028688	0.14829534
ARMC7	200.704176	-0.8402428	0.028741	0.14831979
CCDC88A	2751.58616	0.64076091	0.02871847	0.14831979
KHSRP	1713.80882	-0.3893185	0.02873511	0.14831979
PGD	961.104208	-0.7049455	0.02873168	0.14831979
SYN1	28.2023117	-1.1708581	0.02872088	0.14831979
LINC00852	57.7301915	0.8609684	0.02877602	0.14841283
XPNPEP2	18.2194448	1.67516724	0.02877835	0.14841283
C2orf81	60.0952009	0.94315944	0.0288834	0.14846503
CCDC115	438.947652	0.41544973	0.02887285	0.14846503
CLEC5A	479.860346	1.63468142	0.02882419	0.14846503
FSCN1	1761.43817	0.61602683	0.02887147	0.14846503
GPR98	11.0527119	-1.560419	0.02886748	0.14846503
IRF2	573.053367	-0.5937704	0.02880811	0.14846503
LAMP3	18.8101669	-1.1198695	0.02880926	0.14846503
MIR497HG	231.77805	1.38723521	0.02888551	0.14846503
RANBP1	475.819926	-0.6918149	0.0288593	0.14846503
ROR1	821.319069	1.61723291	0.02888585	0.14846503
SNRPD2	1065.38694	-0.6404091	0.02889478	0.14846503
DIAPH1	1655.82678	-1.0540676	0.02895087	0.1486254
PHLDB2	11557.6095	-0.7038945	0.02895501	0.1486254
TCF3	794.701496	-0.4062426	0.02894042	0.1486254
ZNF581	223.974705	-0.5695317	0.02897823	0.14869489
ARHGAP31	6470.93009	1.12759388	0.02900271	0.14877081
RRP7B	213.254868	0.49947505	0.02902838	0.14885282
PDXP	151.186816	-0.8805352	0.02904065	0.14886603

CARNS1	8.91061406	1.50155008	0.02912663	0.14912712
PEPD	836.616795	-0.5012744	0.0291167	0.14912712
PRKD3	2764.14672	0.5241652	0.02913749	0.14912712
SMG1P7	70.9191836	1.19142222	0.02910456	0.14912712
TEX21P	10.366418	1.61352	0.02914012	0.14912712
MTO1	493.709362	0.4682437	0.0291675	0.14916787
RCSD1	1875.26646	0.8988876	0.02916405	0.14916787
SLC17A7	18.9421718	1.73804104	0.02918177	0.1491912
ENKD1	255.58859	0.77148109	0.02919542	0.14921134
NCL	5384.24617	-0.6338622	0.02923433	0.14931082
RASSF1	388.208639	0.82945958	0.02922535	0.14931082
ZNF577	557.659425	0.58075513	0.02927682	0.14947815
RNF19B	436.402849	-0.9605685	0.02931496	0.14962317
HIST1H4H	591.960057	0.67557074	0.02934104	0.14968972
SPN	207.753686	1.37321476	0.02934749	0.14968972
PRR14	318.916188	-0.5945849	0.02940986	0.14995809
KIF3C	258.470171	0.9431338	0.02942499	0.14998542
HAUS2	588.826102	-0.5612181	0.02946215	0.15012506
LOC1001333	78.3409766	-1.039185	0.0295112	0.15032514
ADRB1	353.138484	-1.5978852	0.02954358	0.15044019
UBE3B	1266.58509	-0.4898471	0.02955413	0.15044405
MAGI1	842.195195	0.81243266	0.02957505	0.15050065
HCCAT3	32.9658959	1.14517351	0.02962184	0.15068887
KIAA1210	318.232463	1.73280723	0.02970271	0.15105022
LAMP2	3622.32402	-0.63392	0.02973926	0.15118607
CCDC25	846.8412	-0.5636708	0.02975994	0.15124115
CDKN1B	1773.39673	-0.7658984	0.02978872	0.15133733
PDLIM7	1497.99013	-1.0351886	0.02979946	0.15134185
NFATC3	3089.00347	0.54785619	0.02990264	0.15178536
PEMT	189.278315	0.79681941	0.02990655	0.15178536
LONP1	1245.47025	-0.5168732	0.02994854	0.15189815
PTRH2	146.686279	-0.7663625	0.02994182	0.15189815
SLC12A4	1777.97401	0.95835812	0.02997856	0.15195003
ZDHHC21	1409.99137	0.59457422	0.02997496	0.15195003
PRKD2	608.381749	-0.6621826	0.0301072	0.15255174
MRC2	25610.1756	0.85575544	0.03018367	0.15284538
MRPS2	311.64565	-0.5402504	0.03020338	0.15284538
PPP1R3G	18.0922132	1.22859367	0.03020495	0.15284538
TRAK2	1373.8468	0.42436795	0.03020483	0.15284538
ANK2	4453.66848	1.59286803	0.03022703	0.15290676
CCDC88C	624.519698	1.13809116	0.03025386	0.15294177
LTBP4	7869.15635	-0.5986702	0.03024931	0.15294177
COQ10B	476.895275	-0.7053773	0.03035103	0.15333684
HIST1H4J	16.2311714	1.15113088	0.03035197	0.15333684
TPPP3	95.5238237	-1.4160232	0.03045422	0.15380283
EIF5A	2443.93631	-0.5410644	0.03052045	0.15386527
HPS1	1079.50463	-0.380601	0.03052668	0.15386527
LINC00882	20.0871602	0.95031598	0.03050161	0.15386527
LOC1005062	29.3939753	1.35004121	0.03051174	0.15386527
RIN2	1470.2049	0.6798335	0.03052242	0.15386527

TIMP2	11022.57	0.62190918	0.03051507	0.15386527
SLAIN1	125.638691	-1.3272661	0.03063767	0.15437409
OSBP	1656.54154	-0.4495953	0.03066862	0.15447934
C9orf85	107.384039	0.51469914	0.03069726	0.15457294
ZSCAN22	139.292979	-0.6406518	0.03071644	0.15461884
PMM1	216.740173	-0.868133	0.03078128	0.15489447
EIF3D	1901.01742	-0.5775351	0.03082311	0.15505417
IL34	964.090738	0.95705379	0.03085927	0.1551345
SUZ12	957.214437	-0.579703	0.03085015	0.1551345
RPS15	4214.99381	-0.4818487	0.03087751	0.1551754
SLC35C1	470.942683	-0.4353078	0.03091574	0.15531673
EIF5A2	314.195431	0.64558323	0.0309801	0.1553613
GATA2	84.7266732	1.55388272	0.03101563	0.1553613
GPRASP1	2001.18036	0.84292272	0.03101403	0.1553613
HMGA1	304.356757	-1.0944121	0.03094962	0.1553613
LOC1001312	13.2281174	1.75945525	0.03099968	0.1553613
MAGOH2	9.41533156	1.55221724	0.03100314	0.1553613
RASAL2	2462.17332	0.73502488	0.03100512	0.1553613
SET	4789.87074	-0.5129984	0.03098685	0.1553613
UBXN4	3000.62337	-0.3424413	0.03096942	0.1553613
CDIP1	480.001518	0.87618444	0.03102952	0.15538022
SMARCB1	502.961376	-0.6892039	0.03111736	0.15576931
DNAJB5	498.077603	-1.0495387	0.03118648	0.15606446
NELFCD	972.215802	-0.6351633	0.03123962	0.15627948
HLA-DRA	1123.25279	1.77949338	0.03128307	0.1564459
NFIA	7900.71781	0.84558154	0.03131049	0.15653209
KCNIP3	10.7239212	1.78144984	0.03135187	0.15668793
CAND2	285.958457	0.93160667	0.03137117	0.15672253
HCN3	144.811341	1.04121021	0.03137919	0.15672253
MYL6	9091.89801	-0.6287297	0.03145299	0.15683616
NAA16	796.936641	-0.587339	0.03144568	0.15683616
NPIPB5	81.4701332	-0.9513061	0.03143482	0.15683616
PCED1B-AS1	65.5488417	1.26949422	0.03144661	0.15683616
RPUSD3	164.122945	0.49338566	0.03143121	0.15683616
DBNL	1794.10225	0.57101797	0.03149991	0.15696825
LUZP1	2026.5645	0.74761713	0.03149822	0.15696825
PGM2	497.443027	0.38194751	0.03151445	0.15698976
MAPK8IP1	257.852231	-1.1540818	0.03168747	0.1578005
BCAS4	90.1018572	-1.3691625	0.0317345	0.1579835
CCDC147	15.2937551	-1.194867	0.03174843	0.15800167
RPL34-AS1	13.136738	1.51881732	0.03176683	0.15804203
LDHD	98.0089679	1.61800111	0.03178613	0.15808686
RGS5	5401.56365	1.07387709	0.03180418	0.15812543
RTN4R	36.1711849	-1.4507659	0.03182962	0.158155
ZNF747	351.578939	0.62147388	0.03183071	0.158155
RAB11FIP1	1525.41871	-0.8323197	0.03190553	0.15847546
CDH23	7250.19103	1.67337451	0.03192712	0.15853146
FAM198B	1061.17732	0.93256688	0.03194413	0.15856467
GPR34	1345.90169	1.00144269	0.03201189	0.15884971
ACPP	64.5171983	1.33928972	0.03205188	0.1589968

STOML2	785.029068	-0.5876533	0.03207142	0.15904235
TMEM79	61.3733905	0.93368884	0.03212912	0.15927709
TAF10	467.98628	-0.7762534	0.03214114	0.15928525
C16orf13	318.460627	-0.446676	0.03222793	0.15962966
MYL12B	5343.81495	-0.6965439	0.03223645	0.15962966
PES1	708.047853	-0.8102523	0.0322418	0.15962966
SLC10A7	229.559911	0.44232499	0.03229028	0.15981818
ZNF101	123.545661	-0.5972879	0.03236248	0.16012394
ANKRD24	56.9491029	1.10429504	0.03242465	0.16037989
RBBP7	1952.63852	-0.6970849	0.03245589	0.16048271
SPATA5L1	162.758329	-0.5934579	0.03249375	0.16061821
FAM221A	164.211308	0.94421218	0.03255407	0.16081291
MAGIX	53.1969876	1.50529145	0.03254703	0.16081291
C1orf213	98.1476163	1.06525005	0.03256786	0.16082932
UBAC1	581.509036	0.53086928	0.03258208	0.16084787
ADAMTS17	554.187519	-1.1483254	0.03266546	0.16120768
ZBTB24	746.363278	0.6016008	0.03271184	0.16138474
COLQ	198.233002	0.69621734	0.03275868	0.16150102
FTL	22811.6316	-1.1130774	0.03277746	0.16150102
LAG3	11.1220837	-1.1649651	0.0327732	0.16150102
SLC6A6	2193.93917	1.3218689	0.03274885	0.16150102
GIMAP2	188.379521	0.98103468	0.03285498	0.16182562
PIK3R6	49.5071404	0.98950246	0.03286441	0.16182562
PHF10	564.287371	-0.7607133	0.03292109	0.16205278
CENPL	109.487104	0.64954077	0.03293744	0.1620813
CEBPA	281.333553	1.34179759	0.03295453	0.16211347
CTNNBL1	507.309502	-0.626254	0.03297953	0.16218454
MFSD2A	21.7262997	-1.2267915	0.03303229	0.16239202
FAM122B	572.375678	-0.7171939	0.03313262	0.16283316
LINC01114	21.139701	1.83519986	0.03315952	0.16291326
CCT2	1533.83577	-0.4668691	0.03318932	0.16295544
PDGFA	366.989126	-1.0459494	0.03318069	0.16295544
BTNL9	806.631853	-1.4217403	0.03333081	0.16359785
NME8	12.6214228	1.42504054	0.03335888	0.16368331
RRAGD	173.522775	-1.4669657	0.03339696	0.16381784
LOC1002880	222.641155	0.67072389	0.03341422	0.16385018
CD82	137.234709	-0.9016466	0.03343291	0.16388952
ALOX12P2	89.722965	-1.7841395	0.03350379	0.16412856
NBL1	106.475606	-1.1034798	0.03351372	0.16412856
TIMM8A	83.9304228	-0.6392196	0.03350769	0.16412856
TNFRSF10D	504.283371	-1.4690546	0.03353846	0.16419736
IGF2-AS	40.989406	1.53863927	0.03359045	0.16435861
SSBP3	844.882283	-0.7030188	0.03359279	0.16435861
ITGAE	79.4284417	0.86253839	0.03362691	0.16447318
CHN2	567.649541	1.47966464	0.0337625	0.16486956
FAM155A	70.3857346	1.45762182	0.03374854	0.16486956
KCNK15	94.9062023	-1.0483923	0.03377325	0.16486956
LINC00467	52.9038473	-0.6436723	0.03378307	0.16486956
LOC286367	58.1361466	0.74502767	0.03373193	0.16486956
PRR34	12.2144349	1.44545576	0.0337822	0.16486956

PTX3	19.0064259	-1.6703849	0.03376118	0.16486956
DIMT1	267.356846	-0.3258008	0.03380574	0.16487544
STK31	51.6174387	1.15911544	0.03379779	0.16487544
PUM2	3633.99569	-0.463855	0.0338368	0.16492218
TMEM127	1532.24077	-0.2945945	0.03383317	0.16492218
ATF2	2419.12471	-0.6213767	0.03389939	0.16504766
HRAS	278.577695	-0.4241201	0.03387725	0.16504766
SULT4A1	58.0502833	-1.4906939	0.03390551	0.16504766
THRA	1053.09835	0.57450227	0.03390146	0.16504766
HPN	31.7118241	-1.3423815	0.03392859	0.16505541
HSPA2	285.347072	-1.307716	0.03392524	0.16505541
SLC25A42	250.577245	0.71266114	0.03395168	0.16511543
B3GNT5	287.297835	-0.8635248	0.0339899	0.16515057
PPM1K	955.455242	0.72959282	0.03398824	0.16515057
TCEAL4	1595.01321	-1.0252578	0.03399115	0.16515057
BLNK	389.173316	0.99190446	0.03402151	0.1652458
NDUFAF2	160.573187	-0.7991336	0.03407688	0.1654624
LACE1	102.795954	0.70335296	0.03409183	0.16548271
RAC3	53.6341996	1.4444895	0.03410296	0.16548444
FN3KRP	487.620086	-0.4806963	0.03411719	0.16550122
TAS2R31	126.5386	0.56158282	0.03414361	0.16557711
GLS2	19.5930228	-1.4713381	0.03417311	0.16566789
MRPS24	21.8611947	-1.1113622	0.03423592	0.16581537
PIANP	11.7408666	-1.7914219	0.03421435	0.16581537
ZBTB2	313.69557	-0.5623797	0.03423456	0.16581537
ANKRD12	3649.53614	-0.5024105	0.03427092	0.16592622
IFT20	354.67076	0.58408191	0.0342804	0.16592622
CLSTN3	826.191149	-0.6870138	0.03430625	0.16599902
TM7SF2	237.755053	-0.9111917	0.03433531	0.16608734
PCLO	234.129536	-1.3304322	0.03436505	0.16617888
AFG3L1P	356.819927	0.69167478	0.03439394	0.16625501
NAPG	1496.2954	-0.9121722	0.03440244	0.16625501
LINC01232	19.2989868	1.40226324	0.03442691	0.16632096
SH3GL1	1135.76654	-0.5659755	0.03446021	0.1664295
LOC654841	27.3788438	1.04609403	0.03447386	0.1664431
FRMD4B	970.515732	0.75736358	0.03449329	0.1664846
ADC	128.187872	1.01887921	0.03451155	0.16651312
ALDH3B1	371.858095	0.87466943	0.03452088	0.16651312
TUBA4A	129.509144	-1.3648378	0.03454639	0.16653158
ZNF561	1076.23145	-0.5954767	0.03453993	0.16653158
CROCCP2	379.468531	0.97394278	0.03464076	0.16688179
CYP2U1	927.042413	0.90998492	0.03463773	0.16688179
BIN1	964.263755	0.79731667	0.03473687	0.16718752
OMA1	393.487735	0.57840057	0.0347261	0.16718752
ST6GALNAC4	346.543823	-0.7977081	0.03473341	0.16718752
GNB1	5082.46163	-0.670773	0.03474939	0.16719541
PET117	76.742868	-0.6272317	0.03476126	0.16720012
MAGT1	2709.09291	-0.5580095	0.0348096	0.16738025
AIF1L	136.279137	1.45804777	0.03484552	0.16744816
EEF1DP3	14.6296076	1.32182706	0.03483522	0.16744816

NARS	2288.85189	-0.4577523	0.03486499	0.1674893
OCIAD2	73.8768284	-1.478066	0.03488167	0.16751708
FAM21C	703.757949	-0.4419219	0.03494557	0.16761434
KRT18	2599.65566	-1.7836739	0.03493577	0.16761434
MYRIP	111.227116	1.55449416	0.03494279	0.16761434
TSEN2	277.99601	0.54369927	0.03493467	0.16761434
ZDHHC7	1343.07508	0.50251632	0.03497678	0.16771169
CCDC58	88.8041774	-0.7110377	0.03499177	0.16773123
EFCAB1	31.1474691	1.59449382	0.03500597	0.16774267
MRO	263.03472	-1.2330267	0.03501599	0.16774267
TCF25	1791.45573	0.52716061	0.0350961	0.16807399
KCTD10	1593.77176	-0.8846915	0.03515727	0.16831446
RAD54B	65.7187122	1.16354201	0.0351835	0.16838759
NR2F2-AS1	109.575638	1.35174226	0.03540135	0.16937744
GBE1	720.482404	-1.0059966	0.03544467	0.16947914
LINC00341	143.622781	0.92932622	0.03543564	0.16947914
CLIP1	3322.85027	-0.6942187	0.03548047	0.16959753
PIK3R2	1088.35165	-0.4956606	0.03550338	0.16965423
LOXL2	331.174015	-1.2233538	0.03555435	0.16984497
BRIX1	292.940116	-0.6610903	0.0355681	0.16985787
NR3C2	1631.52923	-1.3861587	0.03563368	0.17011818
DOK1	423.122181	0.66347893	0.0356782	0.1702778
ZNF675	254.662758	-0.4218515	0.03573886	0.17051433
C15orf52	622.776946	-1.0663514	0.03577891	0.17054651
COA4	383.176294	-0.4692429	0.03577271	0.17054651
MYLK	3507.54419	1.42402812	0.03576027	0.17054651
SLC24A5	32.6874641	1.60866721	0.03583416	0.17075687
PCYT1A	602.652915	-0.4118901	0.03590396	0.17103645
RBM5	3003.3701	0.53441419	0.0359408	0.17115887
MNT	536.322484	-0.6372177	0.03595598	0.17117811
S100A4	240.879425	1.12713663	0.03598041	0.17124134
AAAS	852.679113	-0.650725	0.03599403	0.17125313
AIMP2	297.528173	-0.6156282	0.03607213	0.17148861
HLA-DRB3	106.502529	1.77905763	0.03608591	0.17148861
SGTA	842.831139	-0.3709503	0.03605809	0.17148861
ZNF141	560.483546	-0.4883094	0.03608818	0.17148861
MTRNR2L10	837.197566	-0.9257399	0.03610982	0.17153839
TSTD1	175.696137	-0.7391817	0.03615019	0.17167708
FOXO6	63.8652083	-1.1372501	0.03619629	0.17180904
RRM2B	1349.02521	-0.6731175	0.03621153	0.17180904
UPF3B	182.215266	-0.5901448	0.03620071	0.17180904
PHEX	46.3243926	-1.6990764	0.03629987	0.17217502
LOC374443	545.361	0.81970129	0.0363382	0.17230365
PAN3	1719.68311	-0.4331174	0.03639302	0.17251031
PDIA3	6820.70217	-0.6103037	0.03642245	0.17259656
DNAH9	76.5988193	1.22009813	0.03644439	0.17264728
CAMK2N1	656.629349	-1.6782892	0.03647833	0.17275482
ST3GAL2	702.710402	0.71347607	0.03650526	0.17282905
GNAS	14674.9336	-0.4668964	0.03655159	0.17289142
IDI1	646.805227	-0.6086136	0.03654648	0.17289142

KLF7	1845.49142	0.66651265	0.03656345	0.17289142
SRM	575.024999	-0.830653	0.03655605	0.17289142
RAP2C	792.886509	-0.6769949	0.03659739	0.17296566
RND2	97.6156796	-1.0261898	0.03660539	0.17296566
SLC4A7	1706.81611	0.93915459	0.03661292	0.17296566
EIF4B	15270.4258	-0.6370996	0.03676537	0.17356282
FOSL2	3749.32243	-1.4252754	0.03677322	0.17356282
YTHDF3	2375.21976	-0.4696136	0.03676972	0.17356282
ARIH2	1619.94339	0.46246509	0.03685606	0.17390041
PAQR8	869.206916	1.38307814	0.03688684	0.17399219
DNAJC27-AS	106.918129	1.22438372	0.03690785	0.17403782
FKSG29	16.9983559	1.22437888	0.03694883	0.17417762
MARS	1388.95352	-0.4248273	0.03700008	0.17434098
PDXDC2P	663.50696	0.93551702	0.03700618	0.17434098
HEYL	219.986231	1.16305645	0.03703218	0.17435982
SLC37A3	821.473881	0.58505371	0.03703288	0.17435982
ATP11B	2280.72065	0.31043522	0.0370481	0.17437806
ZKSCAN3	313.11356	0.60180287	0.03706132	0.17438686
FRAS1	2773.81572	1.52552437	0.03710912	0.17455829
AFMID	233.642095	-0.6881244	0.03713247	0.17456125
FOXS1	29.9923836	1.4056619	0.03713176	0.17456125
PTOV1-AS1	23.6408687	1.23735176	0.03718112	0.17473648
CDC42EP1	337.471164	0.57449258	0.03719621	0.17475394
LOC1002894	36.0567382	0.91156493	0.03724232	0.17478065
MXI1	3371.0112	-1.187535	0.0372474	0.17478065
SLC6A17	82.6088228	1.60402783	0.03722072	0.17478065
THUMPD3-A	279.584365	0.53113056	0.03724072	0.17478065
DAZAP1	857.209496	-0.4927145	0.03733695	0.17504047
NUDT13	55.2038509	0.9159489	0.03731999	0.17504047
SLC5A4	17.9816407	1.66661467	0.037329	0.17504047
R3HDM4	573.672681	-0.5587715	0.03736126	0.175101
UBL5	1223.3182	-0.7915828	0.0373823	0.17514616
LOC1019293	22.6568802	1.62201424	0.03744879	0.17535072
PPP2CA	2105.01809	-0.6372768	0.03743826	0.17535072
MAGI2-AS3	2920.78448	0.62751498	0.03756398	0.17583651
DAO	8.15746631	1.79694348	0.03757872	0.17585193
LAMB3	266.83262	-1.5855091	0.03763693	0.1760707
IRF4	28.8301975	-1.0058714	0.03775878	0.17658696
C16orf70	339.173328	0.41648272	0.03781444	0.17672225
NUDT18	157.362258	0.80021585	0.03782222	0.17672225
PLAUR	227.908507	-1.3925245	0.03781108	0.17672225
PVR	692.425924	-0.8575691	0.03784605	0.17676477
RPGRIP1L	525.252611	0.83033344	0.03785433	0.17676477
NAT10	1061.84389	-0.5967903	0.03791409	0.17699001
MGP	6401.16285	1.19405522	0.03797049	0.17719947
RAN	1928.87624	-0.5317006	0.03816798	0.17806699
PDXK	9439.71069	0.74998478	0.03820199	0.17817157
SAMD3	14.8900231	1.44558212	0.03833459	0.17873573
USPL1	512.760838	-0.5002642	0.03839879	0.17898077
ME3	253.341288	0.65326182	0.03845066	0.17916817

C14orf28	100.831794	0.78549038	0.03846775	0.17918114
KATNA1	231.26458	0.49265889	0.03847677	0.17918114
SHE	467.533467	0.38540281	0.03861897	0.17973439
ZBED3	71.3562435	0.59497039	0.03861344	0.17973439
ZNF445	1841.41639	0.56367494	0.03864803	0.17981516
DOLK	359.351825	0.51354826	0.03870102	0.17999661
ZNF680	658.873739	0.80186789	0.03871046	0.17999661
MTURN	2011.07642	0.67783146	0.03873347	0.18004454
RCOR2	7.96769846	-1.2900096	0.03875593	0.18004454
WNK1	10835.0688	-0.5894537	0.0387462	0.18004454
INCA1	14.1881423	1.09177913	0.03878966	0.18011507
NUCKS1	14162.003	-0.5225921	0.03879456	0.18011507
NMNAT1	261.652034	0.89367263	0.03883861	0.18026514
MED25	439.623699	-0.4711238	0.03886346	0.18032599
WDR44	763.698515	-0.6118533	0.03890187	0.18044969
LFNG	194.799127	1.36112049	0.03893208	0.18053535
WDR91	589.917791	0.62997945	0.03894943	0.18056128
LOC1005066	72.0948992	-0.527209	0.03896776	0.1805723
RASL12	80.9835265	1.39336996	0.03897531	0.1805723
HNRNPA1	2146.05935	-0.6301332	0.03900698	0.18061008
RBPMS	597.658717	-0.9748185	0.03900461	0.18061008
ASAP1-IT2	32.5764035	1.27704009	0.03904472	0.18073036
SFRP2	67751.755	1.58226212	0.03906042	0.18074857
PITPNA	1929.77885	-0.5265907	0.03910664	0.18085348
ZBTB47	1629.77176	0.74891979	0.03910451	0.18085348
C12orf5	594.113987	-0.6741531	0.0391287	0.18090107
THOC6	179.98231	-0.5718876	0.03917946	0.18108123
FBXO22	790.715205	-0.4348993	0.03922931	0.18125711
C2orf91	9.29538989	1.61502104	0.0392718	0.18138178
KLK10	58.5416609	-1.6635193	0.03927991	0.18138178
CLPX	787.72677	-0.4749118	0.03931025	0.18146738
NEK10	19.168573	1.32783957	0.03932546	0.18148305
P4HTM	527.538869	0.63792734	0.0393513	0.18154774
VMAC	212.399209	0.66112998	0.03942817	0.18184777
CYB5RL	133.474132	0.68888066	0.03946906	0.18192714
ROBO4	439.682919	1.10838477	0.0394586	0.18192714
C1orf115	283.9116	-1.507121	0.03955261	0.18220294
NPHP3	350.956179	0.5495127	0.0395511	0.18220294
VMA21	1794.21244	-0.5670373	0.03958397	0.18229271
HSD17B10	582.877539	-0.5969803	0.03965495	0.18234631
6-Sep	1203.56264	-0.9904471	0.03962553	0.18234631
TGFB1I1	442.482213	-0.8709957	0.03961236	0.18234631
TMC3	33.4246905	1.78052958	0.03964714	0.18234631
UFM1	1582.44192	-0.5425639	0.03963156	0.18234631
CSTF2	235.663517	-0.5348823	0.03969996	0.18249863
NHP2	676.13625	-0.525633	0.03971509	0.18251361
ZFP2	84.1108356	0.97823784	0.03978718	0.18279021
DHODH	108.229029	0.57447469	0.03981825	0.18287826
ZNF350	358.096635	-0.5285605	0.03983515	0.18290118
SEPT7P2	213.846962	0.81470577	0.03987494	0.18302918

OCA2	149.927943	1.72605215	0.03992957	0.1832252
KLF5	8777.79522	-1.1612776	0.03997215	0.18336582
C16orf89	2147.61369	1.23785696	0.04001567	0.18345589
LGI3	83.0790265	-1.4107631	0.04001088	0.18345589
KAT5	834.620873	-0.4052703	0.04007245	0.1836614
GYG1	836.249559	-0.515735	0.04008953	0.1836849
CCNL1	1283.81954	-0.5401969	0.04010627	0.18370679
IQCG	206.018834	0.71830569	0.04012131	0.18372091
C12orf57	1094.88893	-0.5628332	0.04017297	0.18390264
PGBD5	41.5574307	-0.9639795	0.04026419	0.18426533
MAMSTR	82.874511	1.35063273	0.04028597	0.18431011
RHNO1	314.813968	-0.626767	0.04030561	0.18434505
FAM98C	171.032371	-0.5326787	0.04033968	0.18444596
GCSH	124.118978	-0.675388	0.04035648	0.18446789
PPM1D	567.766359	-0.5106257	0.04041121	0.18466314
HPGDS	76.206555	0.92927	0.0404408	0.18474338
SDC1	310.278408	0.9240647	0.04046387	0.18474897
SIRPB2	188.633046	1.23428549	0.04046607	0.18474897
PNPLA7	280.464684	0.88093146	0.04047959	0.18475576
APBB3	699.912022	-0.4431285	0.04051499	0.18486241
CASP8AP2	974.652688	0.43327636	0.04059544	0.18511955
TANK	625.769986	-0.6413447	0.04058995	0.18511955
JPH4	80.8124154	1.72575141	0.0406855	0.18547517
DIABLO	619.940281	-0.4677553	0.04083822	0.18611618
LOC1019271	25.4096836	1.19228853	0.04085269	0.18612691
KDELC1	223.179073	0.86137488	0.04094995	0.18633039
METTL5	266.972285	-0.5020155	0.04094229	0.18633039
SAAL1	153.258553	0.57011269	0.0409288	0.18633039
SPAG4	114.233872	-1.5210845	0.04092391	0.18633039
TFE3	1704.93979	-0.6365201	0.040958	0.18633039
CPS1	454.571616	1.01843886	0.04099305	0.18637947
TNPO2	2307.48723	-0.4409928	0.04098521	0.18637947
ABCA10	122.71863	-1.1927391	0.04103006	0.18643739
CROCC	548.567885	0.68401082	0.04102623	0.18643739
DHX33	499.834105	-0.5815338	0.04108658	0.18663736
KCNMB2	7.92214541	1.764213	0.04110165	0.18663736
11-Sep	4359.80066	0.92407531	0.04111051	0.18663736
FBXL3	2268.6555	-0.5174164	0.04114632	0.18674473
REEP2	325.401308	-1.3659449	0.04120586	0.18695974
JOSD1	1221.58578	-0.7991479	0.04128986	0.18728555
PTGS1	524.014216	0.94410053	0.04133341	0.18742775
SHKBP1	493.457537	-0.5704404	0.04143292	0.18782351
SYS1	1145.77842	-0.6265988	0.04149212	0.18798096
WDR81	1598.48427	0.6479701	0.04148619	0.18798096
ERI2	255.94301	0.45647721	0.04152251	0.18806319
EXT2	2539.70213	0.61299743	0.0416096	0.1883466
LAGE3	114.54063	-0.5720918	0.04159836	0.1883466
PARP6	1228.49522	-0.4064053	0.04165049	0.18847614
NT5DC2	665.444804	1.20383729	0.04170772	0.18867954
LYL1	118.108022	1.12588449	0.04175963	0.18885876

LOC1005075	170.568354	0.9058486	0.04179255	0.18890274
TRIM71	8.51127974	1.75738613	0.04179395	0.18890274
APBA2	353.937619	1.14252744	0.04188109	0.18924093
PRKCI	2208.1639	-0.7602948	0.041909	0.18931139
HK2	755.325587	-1.1948799	0.0420702	0.1899837
CASC4	5488.42822	-0.5906655	0.04211895	0.19001981
GXYLT1	1496.5006	-0.8202105	0.04212052	0.19001981
N6AMT2	148.29626	-0.6647066	0.04212768	0.19001981
SPG20OS	9.83702712	1.42175794	0.04210144	0.19001981
ADAMTS9-AS	165.265537	1.52970504	0.0421804	0.19014598
MBD6	1046.47838	-0.6284752	0.04217023	0.19014598
RASSF2	11983.9063	1.26063239	0.04222	0.19026867
TMEM221	10.4184485	1.33712523	0.04231897	0.19065877
HNRNPR	2359.58263	-0.4834422	0.04236166	0.19079514
RNF2	475.094046	-0.3587982	0.042414	0.19097488
BCL2	1496.27477	1.33845559	0.04248351	0.1912318
FARSB	553.130862	-0.5292486	0.04251491	0.1913171
PRKAG1	751.763767	-0.436374	0.0426539	0.19188635
ERLEC1	1473.41667	0.6490634	0.04275786	0.19229771
FCGRT	4589.85567	0.54758549	0.04278912	0.19238199
NDUFB5	825.01833	0.55373987	0.04282759	0.19249863
MKL1	843.633737	-0.7039984	0.04284762	0.1925323
FGF17	25.9454388	-1.0442245	0.04288821	0.19260202
PPP1R37	417.571038	-0.386154	0.04287944	0.19260202
CECR1	856.36453	0.90211734	0.04292974	0.19267589
GMNN	118.361363	-1.173501	0.04292891	0.19267589
LINC00032	7.69319968	1.62345411	0.04294927	0.19270727
SLC2A3	2447.75673	-1.644266	0.04296423	0.19271807
CCL2	121.239641	-1.1128269	0.04302759	0.19288968
L3MBTL1	444.769102	0.61144811	0.04301815	0.19288968
SCYL2	1259.23281	-0.6376317	0.04307656	0.19299654
TMEM205	1367.6984	-0.9707061	0.04307241	0.19299654
CBX6	1103.68373	-0.658424	0.0431173	0.19312276
CPA1	19.2362736	1.48096599	0.04315089	0.19321686
TIFA	152.292403	0.52774371	0.04317271	0.19325825
NOL4L	797.574561	-0.8182362	0.04319911	0.19332012
SLC18B1	341.560447	0.70381207	0.04324122	0.19345218
ANAPC7	880.232366	-0.3857773	0.04328293	0.1934698
ARPC1A	1406.6551	-0.6432467	0.04325811	0.1934698
PRPH2	198.427157	1.32534361	0.04327308	0.1934698
RPS6KA5	132.881163	0.94016387	0.04330863	0.19352837
ZBTB18	422.273059	-0.5741481	0.04335597	0.19368357
ALDOA	10429.7778	-0.8550982	0.04343435	0.19393275
MED15	914.142185	-0.6481001	0.04346224	0.19393275
SLC25A44	577.369513	-0.6007212	0.04345587	0.19393275
STX16	1402.12832	-0.7545511	0.04346186	0.19393275
MAD2L2	182.517531	-0.7706284	0.04349184	0.19398724
TMEM135	1263.47723	0.66326838	0.04349971	0.19398724
ATP8B5P	9.49010833	-1.5007067	0.04357862	0.19428277
LOC1005062	8.33178708	1.51362813	0.04362824	0.19438508

RRN3P1	133.857383	0.87137293	0.04361847	0.19438508
TMEM170A	118.46182	0.74882173	0.04363953	0.19438508
DOCK2	1404.00595	1.05442284	0.04367165	0.19447176
SEMA4D	595.754487	1.02373877	0.04369325	0.19451157
FAM199X	2813.05769	-0.5870897	0.04370691	0.19451599
ATG16L2	291.176169	0.90510148	0.04379442	0.19480398
HDHD2	1020.5887	-0.6389936	0.04379698	0.19480398
PCDHGB3	393.181254	-0.7714037	0.04386481	0.19504921
TIMP4	30.0067995	-1.421195	0.04389694	0.19513562
BRWD3	1309.495	-0.51405	0.04392087	0.19518554
RABEP2	289.458948	0.62458493	0.0439458	0.19523984
TIMM17B	277.192007	-0.6088438	0.04396202	0.19525544
LOC1019277	20.0901499	1.69148304	0.04397565	0.19525953
TET2	2204.29422	0.49388786	0.04400232	0.19532146
RBM28	535.273247	0.41545957	0.04408473	0.19563076
AK9	203.721743	0.9366001	0.04419996	0.19599423
C9orf129	8.16769448	-1.603152	0.04420491	0.19599423
NBPF11	27.566811	0.97134088	0.04419561	0.19599423
BRE-AS1	45.6777617	-1.3728161	0.04433619	0.19646291
DCC	782.467291	1.73046457	0.04432471	0.19646291
FAM124B	31.9189756	1.34560148	0.04438345	0.19655894
URAHP	11.5925216	1.66194892	0.04437854	0.19655894
CADPS	41.6683245	1.73766166	0.04442389	0.19668135
NDUFAF1	291.773131	-0.7289968	0.04456301	0.19724042
BTK	260.923328	1.14609228	0.04462844	0.19742014
CWC25	435.830178	-0.5498775	0.04463052	0.19742014
SNORA80B	13.1682768	1.21822514	0.04464216	0.19742014
MRPL42	870.617747	-0.5349578	0.04467278	0.19749866
CD151	2733.46182	-0.545807	0.04469562	0.19754279
PSMA4	983.153628	-0.5512856	0.04473539	0.19766171
RPL18	6077.25252	-0.4569376	0.04475058	0.19767197
NHP2L1	1059.19523	-0.7052079	0.0447897	0.19778791
GPR161	889.060853	0.85778651	0.04482756	0.19789824
SLC7A14	88.5162193	1.50581281	0.04484558	0.19792088
LAMTOR5	489.584802	-0.7850519	0.04489319	0.19807412
MRPL17	510.811017	-0.4657841	0.04492255	0.19814674
RAB1B	2194.7423	-0.4499224	0.04494911	0.19820701
ASGR2	9.0852703	1.48156438	0.04510855	0.19862502
C14orf37	311.149733	1.28092156	0.04509729	0.19862502
DLX5	14.6287446	1.66131825	0.04509687	0.19862502
DOCK3	80.5828776	-1.4740639	0.04509167	0.19862502
MOCS1	536.470769	0.71674933	0.04509721	0.19862502
CDV3	3795.37264	-0.5635047	0.04516224	0.19880443
BCL7B	564.528227	-0.7361353	0.04522942	0.19898615
CLHC1	268.311637	0.77952311	0.04521649	0.19898615
CENPO	235.812187	0.65013033	0.04528032	0.19915304
GDF7	13.4808576	1.41194712	0.04530049	0.19918392
REEP4	194.597466	0.58669064	0.04531327	0.19918392
F11R	1023.81092	-0.7667957	0.04533699	0.19923119
RARRES1	21.9167082	-1.6493982	0.04535861	0.19926919

EMP1	13609.5461	0.765115	0.04546373	0.1995598
PIM1	237.2633	-1.4011279	0.04546296	0.1995598
SHARPIN	554.972149	-0.6489048	0.04544768	0.1995598
KLHDC3	1636.82184	0.58869652	0.0455439	0.19979754
MYO5C	910.999174	-1.0032864	0.04553414	0.19979754
CNTN3	593.174456	1.57384342	0.04559609	0.19996939
AMPH	70.8927887	1.04900599	0.04567763	0.2002698
ARMCX2	1801.18755	0.75243925	0.04571812	0.20030686
CCDC171	357.312914	1.0606391	0.04572651	0.20030686
MEA1	438.71693	-0.5431163	0.04573823	0.20030686
PPAPDC1A	36.8782835	1.64443722	0.04571002	0.20030686
TXNRD1	2168.34907	-0.9352531	0.0457531	0.20031487
DHX30	1434.30683	0.46407194	0.04578437	0.20035286
NIF3L1	298.814167	-0.408496	0.0458009	0.20035286
VAMP4	626.201169	0.39104932	0.04579011	0.20035286
FNBP1L	647.43064	0.59451895	0.04587596	0.20062407
CNOT3	524.011937	-0.4945584	0.0459461	0.20075937
CRTC3	1189.43211	-0.6362198	0.04594181	0.20075937
REV3L	2588.07418	0.5136925	0.04593213	0.20075937
ZSCAN30	697.3514	-0.3679368	0.04596912	0.20080283
TREX2	18.7926436	1.32403801	0.04598492	0.20081476
RASSF8	4065.1315	-0.6438038	0.04617895	0.20160478
CLU	96995.8273	-0.7012204	0.04622904	0.20160814
EGLN1	2055.16381	-0.6005326	0.04622147	0.20160814
GDI1	2469.30926	-0.6335464	0.04625846	0.20160814
LZTS2	824.521872	-0.433109	0.0462389	0.20160814
PSPC1	1127.19967	-0.4585833	0.04623827	0.20160814
TREX1	157.783172	0.76516411	0.04624901	0.20160814
CYBRD1	16522.6678	0.92559481	0.04628544	0.20162575
STRN4	1251.7392	-0.3246683	0.04628875	0.20162575
MIER3	857.718095	-0.6621699	0.04635056	0.20183776
ATP6V1E1	1264.37556	-0.7853715	0.04638388	0.2019247
CD1D	37.3576019	1.26008224	0.04639681	0.2019247
SLC38A6	560.509547	-0.5165114	0.04646397	0.20215974
SUZ12P1	88.8602674	0.47869168	0.04649005	0.20221591
ALPK3	600.045945	1.17570931	0.04656542	0.20242913
TEN1-CDK3	38.8790215	1.07664009	0.0465607	0.20242913
CHD4	4873.14109	-0.5471762	0.04672597	0.20281099
DCP1B	326.426024	-0.4964123	0.04673247	0.20281099
DYNLT1	652.645332	-0.8077453	0.04669683	0.20281099
LOC155060	402.584272	0.79339255	0.04670002	0.20281099
RANBP17	137.919195	1.54040113	0.04671511	0.20281099
ZNF107	423.754998	0.38458877	0.04670725	0.20281099
TMEM57	1042.06971	-0.7210338	0.04675163	0.20283684
YPEL3	738.144857	0.51165616	0.0468029	0.20300196
ATP6V1D	816.95618	-0.6664008	0.04682366	0.2030347
PCDHGA6	589.91055	-0.7403212	0.04691214	0.20336093
ZBTB7B	902.0966	-0.5698357	0.04700656	0.20371275
TMEM240	24.3761768	1.26043384	0.04705875	0.20388143
BASP1	445.593815	-0.6893823	0.0471479	0.20415255

TRIT1	301.215761	0.66487713	0.04713498	0.20415255
RPA1	1247.23486	-0.5088651	0.04723747	0.20448273
ERRFI1	1122.78983	-0.7102755	0.04727829	0.20454597
RFPL1S	49.3228328	1.50657135	0.0472787	0.20454597
AFF3	920.476149	1.14680325	0.04732655	0.20469532
TIAF1	22.1651954	1.1179182	0.04738224	0.20487851
RPS2	9264.60874	-0.4183987	0.0473996	0.20489591
PGGT1B	683.988781	-0.5849497	0.0474167	0.20491218
PNMA1	1171.22441	-0.7881341	0.04746608	0.20506788
NUDT9	417.281402	-0.6772995	0.04750153	0.20516334
NME6	155.731474	0.57797071	0.04758697	0.20547459
COX6A1	1494.30993	-0.5891922	0.04762095	0.20552837
ISM1	236.104501	1.23887951	0.04762618	0.20552837
CCDC36	148.929277	1.61322898	0.04773082	0.2059221
USP6	311.14961	1.70186009	0.04774591	0.20592937
CTSW	22.4956723	1.45730843	0.04779026	0.20600499
ETV5	1708.01595	0.9081562	0.04777857	0.20600499
ATF7IP	4520.05191	-0.4165831	0.04795749	0.20628884
C10orf32	725.725066	0.37678043	0.04799363	0.20628884
COLCA1	320.494921	-1.3620264	0.04800359	0.20628884
MND1	11.1559432	-1.4191363	0.04800381	0.20628884
MYD88	509.818816	-0.790473	0.04789803	0.20628884
NAA25	963.107207	-0.3816468	0.0479247	0.20628884
RAB27A	292.631663	-0.7116502	0.04791752	0.20628884
SENP3	166.393676	0.44473871	0.04789727	0.20628884
SNORA32	19.3276661	-0.9928769	0.0479517	0.20628884
ZCRB1	621.822841	-0.5591351	0.04800218	0.20628884
ZNF703	53.6861876	-0.9932194	0.04800376	0.20628884
C19orf55	115.724748	-0.6408641	0.04817876	0.20675147
GPR63	26.386188	1.39761641	0.04817406	0.20675147
NES	324.405287	1.14768458	0.04815397	0.20675147
SLC25A13	564.565413	0.58252941	0.04816006	0.20675147
SNORA47	136.368339	-1.1477052	0.04814884	0.20675147
CDC73	1512.91719	-0.4187763	0.04823071	0.20685885
GLS	4112.99137	0.837032	0.04822797	0.20685885
LOC1027249	16.196106	1.36418148	0.04825375	0.20689992
PDRG1	188.485519	-0.6938472	0.0484091	0.20750808
MIEF2	228.663562	0.59481899	0.04860349	0.2082409
PML	1249.57451	-0.4407481	0.04860717	0.2082409
SERP1	3662.66309	-0.4911173	0.04862243	0.20824823
AP3S2	80.9053823	-0.6916069	0.04866008	0.2083514
DCHS2	194.835499	-1.5631706	0.04867855	0.20837242
PRAF2	262.211966	-0.5276335	0.04871446	0.20846805
BTD	458.16527	0.90363513	0.04879897	0.20859197
CCDC125	544.391904	-0.5500415	0.04877424	0.20859197
FRAT2	220.963698	0.70290708	0.04876884	0.20859197
PDZD11	443.343507	-0.5306073	0.04879711	0.20859197
SLC25A46	1029.79973	-0.6633297	0.04881131	0.20859197
KLC2	427.80139	-0.5948194	0.04885883	0.20873699
BANK1	28.2219574	-1.2156808	0.04889728	0.20884319

FBXO24	42.6248729	1.00200879	0.04896542	0.20907607
EWSR1	2805.86255	-0.4708869	0.04909918	0.20958897
TERF2	461.231625	-0.5671406	0.04915782	0.20978101
PPM1E	36.5062564	-1.3606945	0.04923482	0.21005124
C14orf159	866.486434	0.83908739	0.04927744	0.21005809
EFNB3	352.797423	1.29653798	0.04925923	0.21005809
ST8SIA5	13.6500976	-1.5417312	0.04926732	0.21005809
PGLS	537.223397	-0.3720133	0.04931327	0.2101525
DHX58	422.249938	0.56840607	0.0493476	0.21023834
HIC1	600.741892	-0.7959318	0.04936078	0.21023834
A1BG	13.3295573	1.19260028	0.04941553	0.21035488
UGCG	521.469695	-0.736306	0.04941428	0.21035488
PTPRVP	41.3666182	1.45220842	0.04943307	0.21037128
MFAP2	100.019122	1.40076454	0.04946156	0.21043423
RILP	99.8378283	1.04929388	0.0494934	0.21045313
ZNF442	211.869665	-1.1973556	0.04949028	0.21045313
OXR1	1533.71265	0.50107493	0.04956008	0.21059604
PACRGL	237.661543	0.48822184	0.04956814	0.21059604
TRIOBP	1589.71882	-0.6799364	0.0495649	0.21059604
ATP6V0A1	1356.30805	-0.5620612	0.04961353	0.21073062
C10orf131	14.4815076	1.31714878	0.04971068	0.21106311
MAGI3	648.916434	0.72706476	0.04971929	0.21106311
PPP1R13L	262.68528	1.02436633	0.04981104	0.2113942
RGS11	752.264403	0.97372821	0.0498569	0.2114673
SPG20	2949.86294	-0.4975469	0.04985313	0.2114673
THEM4	351.291078	-0.6748847	0.04986956	0.2114673
ZNF584	244.995883	-0.5099468	0.04991513	0.21160213
