# Supplementary material for: Transcriptome signatures associated with meningioma progression
Source: Acta Neuropathol Commun. 2019 Apr 30;7:67. doi: 10.1186/s40478-019-0690-x (PMC6489307; doi:10.1186/s40478-019-0690-x)
Supplement: Supplementary file 3 — Table S2. List of significantly differentially expressed genes between all grade I and all grade II meningiomas, as identified by RNA-seq. (PDF 426 kb) [file 40478_2019_690_MOESM3_ESM.pdf]

Supplementary Table 2: Differentially expressed genes between GR I and GR II meningiomas

Gene	baseMean	log2FoldChai	pvalue	padj
SNORA54	64.7422976	2.79051051	2.70E-12	4.69E-08
CYB5R4	163.351946	0.79342842	8.63E-12	5.15E-08
FOXC2	1389.1451	-2.0535001	8.89E-12	5.15E-08
KIF18B	49.1523813	-2.0889794	1.12E-09	4.87E-06
FAM46A	971.176794	1.48273008	9.27E-09	3.22E-05
CDT1	43.6735385	-1.8225603	1.26E-08	3.65E-05
TONSL	106.035819	-1.1999743	3.92E-08	9.75E-05
ADCY5	3873.82711	-1.3428415	4.55E-08	9.90E-05
GATA3	40.597388	-2.4292529	7.08E-08	0.00013671
FAM111B	88.9554194	-2.0708647	8.91E-08	0.00014088
MME	115.438402	2.45659495	8.29E-08	0.00014088
MKI67	524.154621	-1.5324041	1.06E-07	0.00015352
SLC12A1	49.9326174	2.48254528	1.41E-07	0.00018888
HJURP	40.3367613	-1.7629658	2.51E-07	0.0003115
RPL21	4.53268964	2.43670542	2.73E-07	0.00031681
DHRS9	5.09850812	2.35093662	3.75E-07	0.00040707
H2AFX	297.192015	-1.2393945	6.05E-07	0.00061858
SAMD5	452.302445	2.3591128	6.47E-07	0.00062465
SNORA68	64.7557195	1.61643717	8.55E-07	0.00078248
CIT	256.260186	-1.3798076	1.00E-06	0.00081324
SAPCD2	23.8000482	-1.5352375	1.08E-06	0.00081324
SFRP4	2954.03806	2.29567173	1.06E-06	0.00081324
SNORA8	43.4061861	1.07437583	9.82E-07	0.00081324
PRF1	21.8570483	1.73179515	1.34E-06	0.00097149
CXCR6	13.1844377	1.96045075	1.83E-06	0.00122138
FOXM1	113.116152	-1.7000599	1.80E-06	0.00122138
MEX3A	51.4076688	-1.9343662	2.38E-06	0.00153144
NNMT	234.298871	2.14376607	2.63E-06	0.00157758
XKR5	10.1792088	-1.9937042	2.56E-06	0.00157758
MYBL2	30.7118251	-1.6994684	3.60E-06	0.00208421
CDCA8	23.387494	-1.2392254	3.86E-06	0.00211984
EMP1	6904.94278	1.02601769	3.90E-06	0.00211984
RABGAP1L	2426.43287	1.0132424	4.30E-06	0.00226494
CRTAM	7.47832962	2.01753894	4.78E-06	0.00244478
PIK3R2	1082.76502	-0.7533264	4.98E-06	0.00247296
DHCR7	522.223932	-1.4315901	6.25E-06	0.00287182
FASN	2440.98023	-1.029502	6.28E-06	0.00287182
HES7	9.17848303	-1.7865296	6.02E-06	0.00287182
LMCD1	188.364241	1.99831876	6.74E-06	0.00300527
ATP6V0C	952.322412	-0.925104	7.76E-06	0.00336274
TM7SF2	326.641124	-1.2802461	7.93E-06	0.00336274
NCAPH	47.4022198	-1.3730055	8.73E-06	0.00361232
LRFN1	61.6358327	-1.1952381	9.28E-06	0.0037543
LRCH4	1205.26121	-0.6944232	1.05E-05	0.00414693
CD300LF	12.8331021	1.6821662	1.09E-05	0.0041607
SNORA48	444.826349	1.341037	1.10E-05	0.0041607

CKMT1B	6.25381488	-2.0868705	1.15E-05	0.00417597
CNIH2	10.0412955	-1.6871654	1.14E-05	0.00417597
CD48	42.0489364	1.7553302	1.23E-05	0.00428463
LINC00312	20.1184521	1.99094282	1.23E-05	0.00428463
IQGAP3	135.909972	-1.5349083	1.28E-05	0.00436842
CENPF	385.490294	-1.3417648	1.38E-05	0.0045403
MYEOV	20.5617856	1.95857389	1.37E-05	0.0045403
TACC3	203.199935	-1.0642017	1.47E-05	0.00465482
TK1	80.2210915	-1.3952776	1.45E-05	0.00465482
FGF7	269.29751	2.0367368	1.53E-05	0.0047389
GSG2	13.0983519	-1.5853267	1.58E-05	0.00480472
HNRNPUL2	569.176155	-0.6319396	1.68E-05	0.00490909
MYO7B	15.1969971	1.96994831	1.69E-05	0.00490909
TROAP	41.975803	-1.6778743	1.64E-05	0.00490909
RSAD2	134.219731	1.1428797	1.80E-05	0.00512497
TUBB4A	20.6916111	-2.0271591	1.93E-05	0.00541562
ESPNP	1.791792	-2.022447	2.05E-05	0.00565644
SNAP23	832.314878	0.433493	2.13E-05	0.00579345
INMT	3654.08907	1.99689177	2.45E-05	0.00654172
SPP1	2077.98996	-1.7181546	2.50E-05	0.00657823
IRF4	11.9281245	1.60744779	2.84E-05	0.00731724
PRAF2	261.387204	-0.7552604	2.86E-05	0.00731724
ARHGEF39	38.9795826	-1.5887614	3.00E-05	0.00745674
WDR62	43.7854862	-1.25529	2.97E-05	0.00745674
HMMR	44.4257183	-1.2693392	3.05E-05	0.00747781
GLDC	224.224138	-1.6168839	3.30E-05	0.00765989
RNF125	160.778848	1.20169958	3.23E-05	0.00765989
UPK3B	93.3631398	-1.7135747	3.28E-05	0.00765989
ZNF777	229.449886	-0.4996994	3.21E-05	0.00765989
FOXL2NB	7.88568688	-1.9095702	3.49E-05	0.00787987
MASP1	45.1146474	1.94386215	3.53E-05	0.00787987
ZDHHC23	66.5945428	-1.7555192	3.53E-05	0.00787987
ASF1B	55.8757288	-1.3510609	3.80E-05	0.00820692
GRB14	65.1340959	-1.9108577	3.75E-05	0.00820692
SAT1	2459.92264	0.83547748	3.82E-05	0.00820692
TNC	456.817626	1.93542344	3.97E-05	0.00841471
GVINP1	157.839687	1.06773523	4.18E-05	0.00865355
SNORA57	199.996023	1.16678941	4.14E-05	0.00865355
C9orf172	17.8450152	-1.8558674	4.27E-05	0.00873978
NR3C1	2045.44673	0.86228076	4.33E-05	0.008749
ATP2B2	33.2653921	-1.9371524	4.55E-05	0.00880981
HIST1H2AL	70.8788399	-1.1472042	4.51E-05	0.00880981
TIMELESS	327.122821	-0.7470356	4.56E-05	0.00880981
TOP2A	528.593361	-1.2755639	4.50E-05	0.00880981
RRN3P2	67.4032899	1.53490355	4.67E-05	0.00882622
SNORA20	7.52893899	1.80402304	4.65E-05	0.00882622
ALAS2	9.68681026	1.69878359	4.83E-05	0.00894796
CCDC106	211.42203	-0.6755561	4.89E-05	0.00894796
NTSR1	7.2397686	-1.8968845	4.88E-05	0.00894796
MYCL	88.867699	1.69658252	5.03E-05	0.0091038

TNNI2	5.44160085	1.87697497	5.16E-05	0.00924711
BAALC	95.1209984	-1.878775	5.82E-05	0.01032116
CHRM1	60.8437769	-1.8884128	6.42E-05	0.01123791
E2F8	11.394818	-1.630846	6.55E-05	0.01123791
EXO1	29.6515732	-1.486033	6.66E-05	0.01123791
SLC27A6	59.7498857	-1.897834	6.64E-05	0.01123791
SQRDL	541.905034	0.68324605	6.56E-05	0.01123791
SV2A	160.178665	-1.8344552	7.01E-05	0.01171817
KIF5C	108.598516	-1.7193137	7.26E-05	0.01191291
LIX1	24.704353	-1.8443825	7.23E-05	0.01191291
BIRC5	59.6859769	-1.4832718	7.42E-05	0.01205063
APOD	2645.65144	1.85713074	7.88E-05	0.0126782
MLC1	10.612227	-1.8126585	8.04E-05	0.01270728
PLK1	40.6286641	-0.9303432	7.97E-05	0.01270728
CKAP2L	53.3841787	-1.5277708	8.22E-05	0.01286985
UBAP2L	2300.81448	-0.5473994	8.35E-05	0.01296689
CKMT1A	8.10189129	-1.8686413	8.47E-05	0.01303567
FLG	6.0170056	-1.7177142	8.57E-05	0.01306831
STOML3	6.96968784	1.86106032	8.67E-05	0.01310403
GPR171	5.61620884	1.67579016	8.85E-05	0.01314433
RBP4	106.375847	1.8573325	8.81E-05	0.01314433
APBB2	3763.6239	0.95670132	9.48E-05	0.01326195
C9orf72	278.998949	0.69846978	9.53E-05	0.01326195
CD247	10.7359677	1.64873711	9.50E-05	0.01326195
CEACAM21	15.9932161	1.38799454	9.36E-05	0.01326195
FAM180B	44.8329746	1.8601509	9.01E-05	0.01326195
SLC31A2	301.467561	0.97050588	9.32E-05	0.01326195
SNORD15A	4.20904752	1.72899087	9.09E-05	0.01326195
STARD5	51.113757	1.68318704	9.16E-05	0.01326195
ITK	23.3001736	1.58781246	9.62E-05	0.01327671
KIAA1161	268.66546	-1.509933	0.00010257	0.01396808
TMEM71	34.6004469	1.460647	0.00010283	0.01396808
CCR2	22.305924	1.6771506	0.00010479	0.01403851
TBC1D17	551.588804	-0.4072952	0.00010496	0.01403851
HRCT1	69.3284308	-1.3460609	0.00010671	0.01416369
BST2	215.742455	1.12600963	0.00010925	0.01439049
EFCAB12	5.41830619	-1.6210696	0.00011226	0.01459019
GPR21	68.3343227	-0.9175629	0.00011245	0.01459019
SMAD6	466.211727	-1.3058669	0.00011905	0.015278
TIMP2	11693.2804	-0.854391	0.0001195	0.015278
BCAR3	291.733933	1.56945153	0.00012245	0.01535817
DTL	109.754687	-1.3360436	0.00012165	0.01535817
NUSAP1	208.147086	-1.3154905	0.00012278	0.01535817
RNF214	416.623726	-0.521379	0.00012686	0.01568357
TMEM151A	2.70571895	-1.8184341	0.00012719	0.01568357
CDH1	6964.26813	-1.0626426	0.0001296	0.01576438
DDX60	758.615443	0.78408633	0.00012965	0.01576438
ELOVL6	273.414928	-0.9541431	0.00013474	0.01621834
ZNF462	1315.21857	-0.5307467	0.00013525	0.01621834
GLI4	96.8118172	-0.8492944	0.00014179	0.01688529

FAM57B	1.84152747	-1.7896021	0.00014338	0.01695928
CNTN6	21.3682893	1.71536857	0.00014567	0.01708991
MAG	16.2178851	-1.8005717	0.00014726	0.01708991
MRPL12	249.288035	-1.0872394	0.0001494	0.01708991
PDE1C	131.709733	1.75325866	0.00014754	0.01708991
SGOL1	25.118392	-1.3597875	0.00014897	0.01708991
CLEC16A	965.642503	-0.5966922	0.00015048	0.01710096
HSD11B1	18.8336478	1.77845123	0.00015286	0.01715736
PLAC9	139.713449	1.76407172	0.00015295	0.01715736
AMICA1	87.6598187	1.45832366	0.00015495	0.01726955
E2F7	29.7945589	-1.3083616	0.00015647	0.01732875
CSPG5	22.9474439	-1.580894	0.00015877	0.01736143
RRM2	123.921238	-1.4704583	0.00015819	0.01736143
KIF13B	795.455984	0.64665399	0.00016443	0.01786793
RPL10	6274.98926	0.66197874	0.00016898	0.01824852
CNTD2	10.4343686	-1.6294239	0.00017244	0.01850707
HIST1H2BO	79.785941	-1.2210019	0.00017461	0.01851182
MTRNR2L9	44.9687745	1.28915051	0.00017438	0.01851182
MAFA	9.28379036	-1.7774093	0.00017769	0.01872467
PM20D2	309.816305	0.9406419	0.00018124	0.0189832
APLP2	18394.4455	-0.9773797	0.00018741	0.01951235
CABLES2	222.678344	-0.9708408	0.00019214	0.01977204
ELMOD3	193.82149	0.64481761	0.00019218	0.01977204
LOC1030918	39.642703	-1.0999908	0.00019552	0.01988017
SPC25	13.3479767	-1.5846745	0.0001955	0.01988017
AGBL1	44.0690941	-1.7659194	0.00020253	0.02047328
AGR2	190.346518	-1.7309541	0.00020613	0.02053107
SNORA46	4.74953345	1.69763564	0.00020665	0.02053107
VMO1	32.6277353	1.46854069	0.00020558	0.02053107
BST1	140.238402	1.48490116	0.00021265	0.02100752
FTH1	12139.9142	-0.8951283	0.00021508	0.0210092
NKD1	1058.92778	1.75300857	0.00021493	0.0210092
LOC1019269	2.16560281	1.743871	0.00022368	0.02160671
TMEM223	130.184901	-0.7194769	0.00022259	0.02160671
DAPL1	90.6012377	-1.7491294	0.00022495	0.02160934
MELK	53.827606	-1.263311	0.00023224	0.02216628
PID1	291.92102	1.65073619	0.00023585	0.02216628
RECQL4	68.1728441	-1.0414441	0.00023491	0.02216628
RIPK3	29.0237823	1.1979901	0.00023569	0.02216628
FCER1A	18.4550699	1.74075252	0.00024195	0.02261737
ARRDC1	301.255895	-0.5887503	0.00024668	0.02293585
DPP6	79.6289983	-1.7356044	0.00024813	0.02294767
HTR2C	3.80921066	1.71809611	0.00025332	0.02306011
NUDCD2	161.101517	0.5877164	0.00025186	0.02306011
THEMIS	15.1005149	1.39862486	0.00025211	0.02306011
WSCD2	20.0834717	1.72955311	0.00025479	0.02307278
CDC25A	53.9950623	-1.2069971	0.0002594	0.02336869
CD6	14.5510264	1.46125051	0.00026203	0.02348454
EPHA3	213.837595	-1.6505499	0.00026353	0.02349703
AKAP7	178.360219	0.97705692	0.00026728	0.02360284

PITX1	21.7746426	-1.724441	0.00026743	0.02360284
OGFRL1	1274.54116	0.74105156	0.00027682	0.0241867
OR7E2P	15.2545802	1.70373385	0.00027593	0.0241867
ANLN	224.004783	-1.3168124	0.00028031	0.0243688
KIF20A	75.0544624	-1.3643683	0.00028725	0.02484754
WNT6	855.977741	-1.4565186	0.00029771	0.02562542
SLC28A1	5.26275606	1.69933971	0.00029971	0.02566989
AIM1	142.33772	1.17055757	0.00031664	0.02618391
BUB1	71.9879182	-1.1734406	0.00030817	0.02618391
IKZF3	63.53037	1.39188211	0.00031926	0.02618391
KCNE1	19.8029955	1.60736699	0.00031359	0.02618391
OLIG1	9.33133211	-1.709968	0.00031902	0.02618391
RGS6	78.866059	1.7108689	0.00031784	0.02618391
SLC29A1	1385.46232	1.21253615	0.00031563	0.02618391
TESK1	383.369415	-0.6399641	0.00031189	0.02618391
ZNF812	106.826012	1.50674087	0.00031743	0.02618391
DNAJC30	262.306403	-0.6399887	0.00032206	0.02628932
NTN4	449.456681	1.4465496	0.00032391	0.02631714
FAM105A	1031.8286	1.08888095	0.0003321	0.02647044
FSIP2	128.67232	1.70549569	0.00033341	0.02647044
HIST1H2AI	84.522384	-0.9963856	0.00032887	0.02647044
2-Mar	125.791235	-1.0117828	0.00033057	0.02647044
SNRNP200	5316.06865	-0.570378	0.00033043	0.02647044
C11orf30	601.7236	-0.4399414	0.00033736	0.02654116
FAM95A	36.5818931	-1.6813035	0.0003369	0.02654116
P2RX1	7.2688877	1.53537318	0.00033941	0.02658237
SNORA81	296.870745	-0.8684158	0.00034107	0.02659308
FASLG	1.5326484	1.69649556	0.0003475	0.02686949
GREM2	14.4871489	1.67633949	0.00034836	0.02686949
LOC1005075	86.4116688	0.99595091	0.0003508	0.02686949
ZNF444	168.463001	-0.5419499	0.00035055	0.02686949
ARRDC4	567.55272	-1.1603636	0.00035294	0.02691492
ANKRD20A8I	62.0939695	-1.556732	0.0003596	0.02699578
SMOC2	1130.42935	1.54784275	0.00035798	0.02699578
TAGLN3	4.95845704	-1.6727621	0.00035975	0.02699578
XG	1.85115307	1.685851	0.00036021	0.02699578
C11orf84	214.61158	-0.535387	0.00036199	0.02701038
FBXO41	386.96081	-0.8209366	0.00036351	0.02701038
SPRED1	1540.45874	0.41647624	0.00036563	0.02705188
C10orf32	395.707134	0.77338939	0.0003757	0.02754983
CXCL11	14.714114	1.50218837	0.00037977	0.02754983
OTX1	6.06169637	-1.6918088	0.00037549	0.02754983
RNF208	22.8795617	-1.0174066	0.00037908	0.02754983
TGFB3	648.138033	1.22815434	0.00038028	0.02754983
ALCAM	10301.5201	0.92035656	0.00038349	0.02757769
BUB1B	83.0742314	-1.2701914	0.00038504	0.02757769
CCDC85B	88.8916804	-0.6451072	0.00038542	0.02757769
STAG1	1154.22536	0.36951585	0.00039861	0.02840454
KCNS2	3.74361182	-1.683917	0.00040087	0.02844891
MILR1	42.3398894	1.2595035	0.00040304	0.02848642

STK17B	500.471038	1.15344003	0.00040487	0.02850002
MYLK2	2.77132371	-1.6174819	0.00041009	0.028751
MARK1	676.342396	-1.257605	0.00041369	0.0288867
PRNCR1	63.6379107	-1.3307521	0.00041833	0.02909432
ASPM	203.142139	-1.3373551	0.00042536	0.02946522
CDH8	100.669311	-1.6426531	0.00044007	0.03005806
DAPP1	39.1659324	1.39710417	0.00043785	0.03005806
GPR123	2.77180718	-1.672036	0.00044084	0.03005806
PRRC2C	5684.74013	-0.3447565	0.00043729	0.03005806
SIGMAR1	593.673992	-0.5186597	0.00044784	0.03041671
KCNJ5	46.0448957	1.09471902	0.00045683	0.03064417
SNORD89	3.3554971	1.661578	0.00045365	0.03064417
TFG	1324.92941	-0.5763249	0.00045824	0.03064417
WDR66	29.7686489	1.32909951	0.00045757	0.03064417
MCOLN2	22.2842173	1.46254036	0.0004639	0.03090381
FCN1	9.58779747	1.53624245	0.00046957	0.03111324
GADL1	12.2254329	-1.6634357	0.00047063	0.03111324
CCDC126	206.915522	1.05496355	0.00047843	0.03150916
GJA3	105.822494	-1.65261	0.00048578	0.03187237
COG8	361.729413	-0.4679964	0.00048977	0.03201365
DCDC5	19.8728414	-1.3518532	0.00049778	0.03205493
EZH2	153.360065	-0.9664642	0.00049747	0.03205493
POLR2L	1021.25858	-0.7201249	0.00049283	0.03205493
TMEM140	143.045343	0.82019757	0.00049651	0.03205493
ST8SIA1	157.07857	1.65504971	0.00050321	0.0322847
TYRP1	4.29170551	-1.6295323	0.00050506	0.0322847
UST	806.468149	0.92203249	0.00050924	0.0324329
EHMT1	1043.19389	-0.420528	0.00051287	0.03254453
OAZ1	2893.69413	0.42755196	0.00053237	0.03353712
PIWIL2	14.4295837	1.48403773	0.00053198	0.03353712
LRRK2	964.687633	0.77476071	0.00054196	0.03401803
C4orf19	214.311013	-1.4065298	0.00054612	0.03415619
SERPINI1	62.2110083	1.2625427	0.00055271	0.03432122
TEX36	9.73891582	-1.4774842	0.0005519	0.03432122
MISP	80.2065308	-1.6170623	0.00057062	0.03527775
ZNF831	11.991846	1.5192112	0.00057217	0.03527775
ESRRG	27.4190578	-1.6286888	0.00057608	0.03536497
PAX2	9.91804074	1.55366877	0.00057969	0.03536497
SOX18	73.517234	-1.3077802	0.0005793	0.03536497
KCNJ3	12.795677	1.62940066	0.00058264	0.03537998
MACC1	52.1831125	1.27095458	0.000584	0.03537998
GSTM1	11.0895293	-1.6321575	0.00059207	0.03574433
DAAM2	461.864341	1.33448715	0.00060091	0.03604123
FAM83D	20.6180869	-1.4114271	0.00060296	0.03604123
IL12RB1	30.3169513	1.16579459	0.00060321	0.03604123
IL18R1	61.9000131	1.22943095	0.00060933	0.03608977
STAT4	13.9256443	1.30016166	0.00061232	0.03608977
ZBP1	6.13699663	1.44784316	0.00061152	0.03608977
ZNF620	58.3415371	-0.6239588	0.00061143	0.03608977
LINC00899	20.6782781	1.10552892	0.00061718	0.03613108

RABGGTB	372.295027	0.59536083	0.00061608	0.03613108
TMEM37	307.689208	-1.1812858	0.00061981	0.03616305
EHD1	4469.46966	-0.9512801	0.0006219	0.03616382
DRD2	26.6357451	1.61852212	0.0006283	0.03635173
HIST1H2AJ	58.857517	-1.0956936	0.00062945	0.03635173
STK31	24.2490155	1.20611604	0.0006314	0.03635173
ANGPTL1	38.4590596	-1.6071767	0.00063428	0.036397
NBPF9	57.3939878	0.94717243	0.00064061	0.03663913
CTDSPL	781.928522	0.98372183	0.00064709	0.03685678
LOC1019272	9.78402027	-1.6217263	0.00064866	0.03685678
FAM127B	336.144905	-0.7140623	0.00065298	0.03698179
TEKT3	11.4762194	1.30214049	0.00065961	0.03711519
THBS1	3879.64074	1.57919956	0.00065851	0.03711519
CCNB2	40.1810529	-1.3010459	0.00066673	0.03739519
DRD4	13.8962408	-1.2393353	0.00067198	0.03756824
C1QTNF2	32.2221223	1.43508498	0.00069967	0.03796438
CCR7	8.92309499	1.44767236	0.0007009	0.03796438
CNTN4	585.262809	1.60242811	0.00069377	0.03796438
COL8A2	1309.36471	1.44751474	0.0006968	0.03796438
HHIP-AS1	40.5567379	-1.5729606	0.00069895	0.03796438
MAP4K2	390.73645	-0.4928783	0.00068617	0.03796438
NCBP2-AS2	135.966265	-0.5672064	0.00069873	0.03796438
PAQR4	63.3348128	-0.9839475	0.00069241	0.03796438
PCDHGB6	459.839468	-1.1329565	0.00069214	0.03796438
TEC	120.521681	1.18170893	0.00069262	0.03796438
NBPF25P	26.6113457	0.93004574	0.00070388	0.03800749
GPM6A	34.8664574	-1.6025461	0.0007102	0.03822973
C19orf54	128.334239	-0.7245333	0.00071661	0.03828402
FLJ22184	13.367885	-1.4081695	0.00071849	0.03828402
FPGT	293.02551	0.8072588	0.00071548	0.03828402
LOC1005068	15.0375787	0.98412443	0.00072001	0.03828402
DAPK2	86.1383211	1.38100327	0.00072777	0.03855662
PDLIM5	3338.41928	0.64966617	0.00072958	0.03855662
LOC1019273	8.76884021	1.59511917	0.00073566	0.03876028
GPR64	498.340095	-1.5870444	0.00074777	0.0388106
LYST	1648.24958	0.72135079	0.000747	0.0388106
PHYHIP	22.7438981	-1.4834445	0.00074615	0.0388106
PLS1	143.229114	1.41661268	0.00074242	0.0388106
TMEM63C	4.68724508	-1.5850096	0.00074626	0.0388106
MIR3648	115.332821	-1.5888939	0.00075177	0.03890207
RTP4	32.6177503	1.28010557	0.00076225	0.03932713
CD300C	42.5485309	1.16743996	0.00077671	0.03994704
SCARNA2	3244.44112	0.89773993	0.00077886	0.03994704
EPHA1	4.54795795	1.57090151	0.00079072	0.04043595
AKT1S1	499.073891	-0.6182784	0.00079419	0.04043975
CORIN	22.0802553	1.56805267	0.0008001	0.04043975
EIF2AK1	1861.22239	-0.446919	0.00079625	0.04043975
GIT1	1092.95322	-0.5820877	0.00079841	0.04043975
ABLIM2	38.2692368	-1.2501081	0.00080555	0.04059754
NEK2	19.831797	-1.1907412	0.0008299	0.04170385

ALG3	241.211946	-0.5951646	0.00085764	0.04176984
CCDC146	109.833519	0.89223801	0.00084249	0.04176984
CD300LB	7.47382311	1.39437042	0.00085702	0.04176984
CEP55	41.0790013	-1.0984243	0.00084178	0.04176984
CRAT	752.581721	-0.6063502	0.00084417	0.04176984
KIF4A	46.7707193	-1.2723694	0.00084978	0.04176984
KIFAP3	832.683299	0.43584585	0.00084625	0.04176984
PCSK1N	266.49682	-1.3623285	0.0008569	0.04176984
RNF148	28.1598837	-1.3172246	0.0008561	0.04176984
RSPO3	330.284535	1.56933262	0.00085734	0.04176984
SNORA72	3.99947742	1.53708817	0.00085421	0.04176984
EBP	173.839071	-0.7110062	0.00086866	0.04212618
IFI44L	238.458502	1.12193095	0.00087465	0.04212618
PCDHB10	233.861643	-1.1647519	0.00087063	0.04212618
PCLO	377.98576	-1.3261758	0.00087457	0.04212618
ZNF136	315.559268	0.61527854	0.00088036	0.04228422
OAS1	278.489062	0.70737919	0.00088589	0.04241226
UHRF1	89.9798516	-1.1039898	0.00088791	0.04241226
CFP	14.3801087	1.16423172	0.00089278	0.04252812
COMMD10	237.557634	0.72679253	0.00092684	0.04402985
CLPTM1	1462.90645	-0.4765615	0.00093462	0.04415811
PPP1R14B	483.461703	-0.8518198	0.00093371	0.04415811
ATCAY	2.05592009	-1.5662547	0.00093773	0.04418529
TRAF4	547.558686	-0.6617523	0.00094497	0.044406
CDC20	22.3895803	-1.1427767	0.00094819	0.0444372
SCML4	6.37639474	1.44184643	0.00095516	0.0446435
ELP2	1003.58075	0.5588401	0.00096062	0.04477809
ATG9A	931.559122	-0.5709322	0.00096411	0.04482078
FBN3	53.6619682	-1.4136766	0.00096823	0.04489226
SMG7	1788.97433	-0.7230937	0.00097708	0.04518219
ARHGAP23	2148.24881	-0.6441682	0.00098023	0.04520777
LOC1006535	88.1373372	-0.836919	0.0009853	0.04532134
BVES-AS1	3.05804349	1.56122997	0.00098827	0.0453378
FABP7	4.09153181	-1.5472487	0.00100019	0.04565462
LOC729683	15.7319099	1.09857655	0.00100043	0.04565462
PRRX1	2251.38101	1.41309832	0.00100334	0.0456678
FFAR2	2.97140918	1.56111662	0.00101556	0.04577716
HIST1H3B	190.856075	-1.0900759	0.00100913	0.04577716
PMEL	32.0076202	-1.1428225	0.00101117	0.04577716
UBXN7	831.356524	-0.3029137	0.00101628	0.04577716
BCAN	4.68393993	-1.5548811	0.00102631	0.04599104
PCDHGA3	310.829476	-1.4838208	0.0010242	0.04599104
TGIF1	368.640799	0.68378685	0.00102943	0.04601196
DDX58	495.400716	0.5246241	0.00103607	0.0461901
CDCA5	43.8916124	-1.181419	0.00104489	0.04646418
FDPS	872.758372	-0.6426946	0.00104914	0.04647449
PROC	2.14426731	-1.5488683	0.00105047	0.04647449
INCENP	260.145488	-0.5362484	0.00106668	0.04683446
KL	16.4374657	1.12945836	0.00106553	0.04683446
VNN1	30.7929398	1.16492364	0.0010658	0.04683446

JADE3	297.051228	-0.6834265	0.00107211	0.04695398
AMER2	12.9198525	-1.5496231	0.00108596	0.04722388
ATG4C	162.724091	0.77325239	0.00108913	0.04722388
DPYSL5	5.34227726	-1.5449853	0.00108663	0.04722388
ISG15	174.603844	0.95056697	0.00108899	0.04722388
KBTBD12	12.0700598	1.530281	0.00110396	0.04761635
MEN1	363.925957	-0.578082	0.0011064	0.04761635
SEC14L5	7.56753197	-1.3481778	0.00110517	0.04761635
RABL6	1057.50984	-0.4650734	0.00111053	0.04767618
AATK	159.188281	-0.961993	0.00111562	0.04771311
ZAK	1010.20175	0.69052556	0.00111688	0.04771311
RAD51	24.9627904	-1.0934468	0.00112124	0.04778169
CDH7	32.3563319	-1.5407543	0.00113288	0.04816001
LINC00861	7.66901119	1.25354494	0.00114432	0.0485274
CNOT3	470.275534	-0.5358071	0.0011579	0.04874684
FLRT2	2645.62996	1.32790283	0.00115728	0.04874684
ZNF467	157.037014	-1.0537655	0.00115666	0.04874684
SOX9	86.2333095	-1.5414808	0.00117338	0.04927902
CALB1	257.196546	-1.506831	0.00118575	0.04938759
FBRSL1	560.503295	-0.7170961	0.00118732	0.04938759
SEMA6D	402.560072	1.30661259	0.00118684	0.04938759
SLC27A2	8.00170566	1.46447194	0.00118155	0.04938759
MYPOP	52.7350641	-0.5992111	0.00119392	0.04953524
RAB43	41.3249386	-0.6808013	0.00119657	0.04953524
CD300E	18.1270086	1.47970317	0.00120908	0.04981593
PKIB	59.3386544	1.26710108	0.00120785	0.04981593
IL6ST	11081.6218	0.62487189	0.00123363	0.05043512
PCAT1	11.7975226	-1.3621275	0.00123095	0.05043512
PCGF5	2006.6329	0.89496413	0.00123571	0.05043512
UBE2C	53.3635218	-1.2771861	0.00123052	0.05043512
SLC7A14	387.469028	-1.5358217	0.00124734	0.05079036
HCN2	51.5538773	-1.3973687	0.00126644	0.05144765
CENPU	71.0962352	-0.9539759	0.0012743	0.05164634
DCLK1	960.485365	-1.2628441	0.00128526	0.05196908
NOD2	44.0843502	1.2857291	0.00128824	0.05196908
CERS2	4129.22422	-0.6036296	0.00131165	0.05242243
LRFN3	102.943811	-0.7217898	0.00130994	0.05242243
RHOD	227.523735	-0.65476	0.00130702	0.05242243
RIMS1	33.0494728	-1.5261625	0.00131574	0.05242243
UGT8	14.003366	-1.5281533	0.00130563	0.05242243
ZNF367	168.679194	-1.0170026	0.00131757	0.05242243
KRT4	6.91901968	-1.5102722	0.00133251	0.05289573
AP1S2	287.786319	0.91890369	0.00134048	0.05309085
ZNF865	253.105494	-0.7664909	0.00135527	0.05355481
AICDA	1.61705965	1.45370486	0.00137026	0.05390188
IL33	51.1608722	1.41486499	0.00137004	0.05390188
FADS2	1239.73439	-1.1399395	0.00138257	0.05414594
NUF2	23.9022785	-1.0560375	0.0013858	0.05414594
TEX41	7.80273756	1.38246971	0.00138467	0.05414594
SLC35C1	515.084931	-0.8827823	0.00139205	0.05426814

CTNND2	18.1070977	-1.4693879	0.00140015	0.05434012
MCM2	243.276245	-0.622713	0.00139922	0.05434012
SESN1	1713.9264	0.91954935	0.00141242	0.0546945
CADPS2	1244.84375	-1.0922617	0.00142319	0.05486685
NFE2L1	15529.557	-0.5338257	0.00142304	0.05486685
CLEC7A	519.609595	1.23990595	0.00142981	0.05487875
ST3GAL5	1023.22008	-0.9070243	0.0014277	0.05487875
ABCC2	25.4278383	1.08967844	0.00145096	0.05544584
HDAC4	2335.17948	-0.7891909	0.00144896	0.05544584
DSE	2447.3402	0.72549317	0.00146095	0.05558327
KIF11	142.275288	-0.9698018	0.00145909	0.05558327
PDHA1	778.86514	-0.5217928	0.00148153	0.05624315
TEX21P	4.36166667	1.40399038	0.00150365	0.05683489
UFSP1	17.2339413	-1.1202075	0.00150054	0.05683489
KCNA6	91.1832728	1.50046299	0.00151034	0.05685841
MEF2D	1687.23935	-0.4834369	0.00151409	0.05685841
UBQLN4	406.237548	-0.5357323	0.00151263	0.05685841
ATP1A3	36.1258303	-1.4870047	0.00152523	0.05694433
DENND5B	517.074911	0.64294817	0.00152812	0.05694433
PRKAA2	649.440585	0.96192509	0.00152613	0.05694433
RPP30	250.311393	0.50338448	0.00152976	0.05694433
ZC3H12D	76.2732659	1.02514355	0.00153275	0.05694433
BRD3	1039.00833	-0.5026635	0.00153648	0.05696133
VAMP5	142.29248	0.86945896	0.00154437	0.05713198
LPHN1	1217.21103	-0.6241518	0.00155319	0.05733621
PIGR	21.1502786	1.50508626	0.0015603	0.05735508
ZNF672	303.222428	-0.6235997	0.00155765	0.05735508
MYRF	32.0929361	-1.453913	0.00157441	0.05775178
FAM114A2	380.5984	0.47470769	0.00158029	0.0578454
PKMYT1	18.5580691	-1.0804598	0.00158371	0.05784883
CD300A	179.312747	1.05578818	0.00159777	0.05823977
GET4	291.176169	-0.4362736	0.00161893	0.05854776
LOC1019271	11.6306092	1.22828343	0.00161969	0.05854776
PTPN22	49.6126799	1.24593279	0.00161364	0.05854776
TRPV6	3.33123132	-1.3826763	0.00161002	0.05854776
CPQ	1538.29294	0.6891295	0.00162652	0.05867274
FAM19A2	29.0226631	0.96077763	0.0016336	0.05868473
SNORA27	8.78269463	0.99740329	0.00163083	0.05868473
CALML3-AS1	34.2981976	-1.4380574	0.00164723	0.05882848
ELOVL2	204.172901	-1.3541436	0.00164775	0.05882848
TBC1D32	304.78348	0.70113663	0.00164696	0.05882848
CHRDL2	2.35827471	-1.4943543	0.0016685	0.05932563
OPA3	799.682831	-0.4968464	0.00166735	0.05932563
PMS2P5	105.193726	-0.5136152	0.00168959	0.0599527
ZNF562	1036.3687	0.34109906	0.00170438	0.06035461
TOMM7	1174.45746	0.56460933	0.00170993	0.06042802
SLC25A22	273.218778	-0.5375116	0.00171687	0.06055009
STON1	134.988846	0.98111675	0.00172244	0.06062372
AFAP1-AS1	10.8211427	-1.4899628	0.00173463	0.06079203
ECH1	746.708395	-0.6118864	0.00174275	0.06079203

FOXD2-AS1	490.291701	-0.6295266	0.00173514	0.06079203
PPP5C	761.149406	-0.4297325	0.0017482	0.06079203
SETD1A	635.289942	-0.519508	0.0017402	0.06079203
ZNF853	258.763868	-0.6514348	0.00174499	0.06079203
GAL3ST3	8.75365684	-1.4540576	0.00175574	0.06093211
PLSCR1	305.033368	0.53386675	0.00176819	0.06124214
KIF14	56.9840475	-1.0590854	0.00177789	0.06137598
LOC1019296	12.7237898	1.48655668	0.00177912	0.06137598
PLEKHG2	1315.73299	-0.6547956	0.00178774	0.06155131
ITGA8	289.883369	1.46569327	0.0018054	0.06179217
LOC1019270	139.401177	0.73832598	0.00180233	0.06179217
ZMYND19	135.822448	-0.5136854	0.00180138	0.06179217
BRINP2	1.62332854	-1.4798459	0.00182516	0.06218981
MMP28	192.213943	1.30493501	0.00182192	0.06218981
PDPN	607.873804	-0.7832818	0.00182774	0.06218981
CLEC4A	45.2094336	0.91303814	0.00185032	0.06259037
CPB2-AS1	11.8309476	1.16847998	0.00184617	0.06259037
GIN1	131.375841	0.62159596	0.00184927	0.06259037
ITGAL	138.738009	1.15539727	0.0018598	0.06276458
NCAN	30.324849	-1.4436373	0.00186269	0.06276458
CREBL2	1212.64574	0.47630466	0.00187034	0.06276822
CYS1	198.06972	1.43146513	0.00188084	0.06276822
DLAT	632.209486	-0.5809461	0.00187957	0.06276822
LY75	33.1145869	1.13643867	0.00186996	0.06276822
MAZ	1061.83571	-0.3785528	0.00187584	0.06276822
RUNX3	57.4784787	1.24502568	0.00189444	0.06310079
LZIC	221.116095	0.58796543	0.00190933	0.06347506
CCDC62	9.06321255	1.02029051	0.00196838	0.06378783
CEND1	22.9678115	-1.4227675	0.00193663	0.06378783
CKS2	55.2696058	-0.9204408	0.00193491	0.06378783
CLASRP	498.758081	-0.4124842	0.00193269	0.06378783
ELAC2	895.959836	-0.3934123	0.00194661	0.06378783
EP400	2174.99998	-0.4984105	0.00195615	0.06378783
GZMA	13.5231778	1.38285634	0.00196124	0.06378783
LRRC56	67.1363228	-1.0339847	0.00195656	0.06378783
LY6H	14.7437169	-1.4734456	0.00195664	0.06378783
MAP3K5	699.737954	0.98622333	0.00194319	0.06378783
POLI	450.190195	0.64174524	0.0019701	0.06378783
RAB31	1324.5195	0.92751953	0.00196453	0.06378783
RNF44	725.340282	-0.4658581	0.00196317	0.06378783
ZC3H3	279.197518	-0.5231628	0.00194244	0.06378783
HEPACAM	10.5372701	-1.4632577	0.0019806	0.06400861
SAMHD1	1023.92067	0.79567058	0.0019887	0.06415128
MAP1LC3B	1424.6711	-0.546608	0.00200371	0.06451571
EPN1	1108.02551	-0.4428056	0.00201862	0.06463685
OASL	40.8264602	1.31210198	0.00201617	0.06463685
RELN	770.32184	-1.4501384	0.0020115	0.06463685
HIST1H3C	105.420162	-1.0338894	0.00202371	0.06468054
CDCA3	21.1465718	-0.9510816	0.00204782	0.06497338
CGN	27.0956535	-1.3197377	0.00204239	0.06497338

HIST1H4I	27.7752329	1.12177974	0.00204386	0.06497338
NAV1	4411.67642	-1.097536	0.00204775	0.06497338
FST	37.0397657	1.46362663	0.00208532	0.0657987
OGDH	2504.39468	-0.6721333	0.00208897	0.0657987
RPPH1	89119.3981	0.49366733	0.00207933	0.0657987
SENCR	3.57665264	1.24059707	0.0020852	0.0657987
GS1-259H13	19.4537603	1.06771356	0.00212206	0.06672004
HLA-DRB5	605.376679	1.44863273	0.00212977	0.06684169
KIF27	162.950138	0.66834507	0.0021527	0.06743971
ARHGAP39	169.059728	-0.8145926	0.0021581	0.06748727
DTWD1	291.969803	0.4970582	0.00218067	0.06807046
LINC00649	7.3261222	1.42019341	0.002189	0.0682081
ZDHHC12	127.23311	-0.7271638	0.00222361	0.0691625
LOXL3	201.583648	1.24792937	0.00224143	0.0694682
NLRC4	45.7561377	1.04764907	0.0022403	0.0694682
KCNJ10	18.6732819	-1.4380122	0.00224653	0.06950251
RASD1	70.1378513	1.42828754	0.00225859	0.06975142
COMMD8	106.676609	0.69619217	0.00229397	0.07046879
STXBP5L	6.70791027	-1.4438401	0.00229314	0.07046879
ZNF775	65.9169199	-0.467364	0.00228826	0.07046879
GRIK2	19.2947482	1.30657513	0.00230261	0.07060937
ABI1	703.155716	0.37152985	0.00232675	0.07122389
NCAM2	15.4647447	-1.2968272	0.00233647	0.07139593
BANF1	420.300617	-0.732991	0.00236402	0.07198454
TRIM9	61.4999241	1.36991203	0.00236075	0.07198454
CCDC79	1.9125435	1.39819574	0.00238462	0.07235842
OAS2	267.937696	0.83741969	0.00238093	0.07235842
GRIN1	8.78227356	-1.3973291	0.00244694	0.07412004
LINC00565	5.13099167	1.3698408	0.00245694	0.07416452
NLRP12	12.6805793	1.26908317	0.00245666	0.07416452
TLR2	828.95426	0.99898925	0.00246177	0.07418174
CHL1	11.4677714	-1.4384912	0.00247134	0.07428925
HSPA12A	418.869675	1.30183106	0.00247389	0.07428925
ATP2B4	9242.95109	-0.4966506	0.00248272	0.07436904
INTU	332.597555	0.78702899	0.00250221	0.07436904
SLC6A6	1033.31681	1.14006649	0.00249282	0.07436904
TBCD	1261.79407	-0.4230105	0.00249314	0.07436904
TMEM242	187.644603	0.54026166	0.0024997	0.07436904
TRIP13	33.6756831	-1.0436208	0.00250157	0.07436904
CPSF7	1443.75445	-0.3626112	0.00250969	0.07446413
PDZK1IP1	7.70385047	1.42760105	0.00251423	0.07447161
BNIP1	70.3813559	0.50656567	0.00254771	0.07475426
BOC	5077.69244	1.20726093	0.00255188	0.07475426
CSMD2	40.8196538	-1.3726518	0.00254918	0.07475426
LOC442028	42.4067647	-1.4030321	0.00255128	0.07475426
PCDHB5	589.296158	-1.1604251	0.00255459	0.07475426
PCYT1A	497.957724	-0.3591695	0.00252965	0.07475426
PHF12	805.026372	-0.3582017	0.00255809	0.07475426
SPATA17	13.8910718	-1.0229323	0.00255816	0.07475426
CDR2L	170.909109	-0.7891797	0.00257054	0.07498978

COLGALT2	21.8237669	-1.3523222	0.00258768	0.07536341
IGSF9B	328.787612	-1.064559	0.0026004	0.07560728
RWDD3	57.1841108	0.74392954	0.00262513	0.07595669
TAF4	342.047653	-0.7807454	0.00262552	0.07595669
TBC1D13	614.882199	-0.2867799	0.00262332	0.07595669
TMPO-AS1	42.5529552	-0.6247427	0.00263258	0.07603448
ECHDC3	14.3366894	1.3424348	0.00266381	0.07621264
NLRP3	278.130152	1.18045544	0.00266098	0.07621264
SCAF4	752.70992	-0.59436	0.00264391	0.07621264
SH3BP4	791.993944	1.08293279	0.00265425	0.07621264
TXK	46.2060951	1.22013929	0.00266505	0.07621264
UBALD2	222.693235	-0.5271109	0.00266093	0.07621264
ZNF423	1683.53135	-0.702123	0.00269017	0.07680466
EDC3	520.134917	-0.3978702	0.00269827	0.07690962
PLEKHB1	61.2241121	-1.2972927	0.00271819	0.07735052
CCNYL1	485.257454	-0.6056742	0.00275323	0.07749221
COL24A1	36.0074805	1.09878254	0.00275443	0.07749221
DKK2	2101.90859	-1.392236	0.00274433	0.07749221
LCK	11.4517425	1.22355793	0.0027561	0.07749221
MCM10	23.8513822	-1.0556516	0.00275882	0.07749221
PIK3R1	4264.52316	1.18217041	0.00273384	0.07749221
PPP2R3C	174.816873	0.69335584	0.00274967	0.07749221
TTK	39.0981922	-1.1188768	0.00274397	0.07749221
TXNDC15	1015.35325	0.47309699	0.0027635	0.07749827
JPH3	32.1483446	-1.4209861	0.00276808	0.07750166
SPATA9	4.17467606	1.40698605	0.00279738	0.07819613
CABP1	3.69424024	-1.4119929	0.00280333	0.07823677
CPLX2	25.4642236	-1.3830655	0.00281201	0.07825479
CPSF4	235.021466	0.48758856	0.00281298	0.07825479
ACY1	19.0695241	-0.9898145	0.00284122	0.07878836
ATXN2	1328.08016	-0.4494518	0.00283961	0.07878836
CD180	59.6859285	1.20019994	0.00287199	0.07946547
LOC1005073	107.406546	-1.3054834	0.00288297	0.07946547
PDCD1	6.88419005	1.41303141	0.00288392	0.07946547
SLC22A18	222.926518	-0.6797516	0.00288008	0.07946547
SLC25A5	1475.8538	-0.5612657	0.00290268	0.07985579
HMP19	3.17481616	-1.3934677	0.0029133	0.08002152
CNTN2	30.97919	-1.4065623	0.00295699	0.08071109
IFT43	79.6509272	0.71891132	0.00296626	0.08071109
IPCEF1	92.9260025	1.12469851	0.00295546	0.08071109
LPIN2	1026.12689	0.74780832	0.00296614	0.08071109
PBK	32.9299384	-1.2986264	0.00294321	0.08071109
PEX5L	61.5979785	-1.3934005	0.00296226	0.08071109
DPF2	676.042073	-0.3176147	0.00298401	0.08094081
NEFM	14.4416212	-1.3864106	0.00298136	0.08094081
ELN	2550.34436	1.37885694	0.00299492	0.08102658
MIR4697HG	219.262736	-1.0734321	0.00299971	0.08102658
RAB11B	950.774311	-0.6214044	0.00300116	0.08102658
HIP1R	359.295327	-0.7872982	0.00301074	0.08115939
CDH23	3054.61716	1.33447104	0.00303081	0.08138545

SNORA76C	9.02129668	1.34859667	0.00303317	0.08138545
TRIM17	124.13236	-0.8865744	0.00303178	0.08138545
HCAR1	75.1732506	-1.0916929	0.00304389	0.08151866
KRTAP5-10	3.25571167	-1.353105	0.00304751	0.08151866
ARRDC3	2976.74667	1.09277638	0.0030594	0.08158567
LILRA1	53.1545123	1.16918182	0.00305907	0.08158567
ADPRM	71.2110662	0.68676373	0.00306492	0.08160748
HAPLN4	40.6838625	-1.3419671	0.00307466	0.08174184
ZBTB24	440.538747	0.53680156	0.00308852	0.08198477
KATNA1	138.310687	0.49605385	0.00309365	0.08199575
LOC1001315	624.574918	0.67259025	0.00310117	0.08207002
MRPL14	156.285927	0.46572597	0.00311306	0.08225957
FBLL1	21.6624985	-1.3280561	0.00313543	0.08263355
ITGB4	8808.19914	-1.1464662	0.00313672	0.08263355
APOL1	612.969323	0.86034052	0.00316233	0.08293501
RGP1	408.689085	-0.4451677	0.00316247	0.08293501
SPAG5	144.038065	-0.8613314	0.0031588	0.08293501
GPKOW	252.064259	0.36711946	0.003184	0.08324835
KIAA0101	31.6370357	-1.0807114	0.00318002	0.08324835
FAM13A-AS1	101.553891	0.72296104	0.00321952	0.08392478
HIVEP3	334.903314	1.16739266	0.00321617	0.08392478
CNTNAP2	11.7921351	-1.2607248	0.00325019	0.08443111
FAM131A	215.878807	0.86563474	0.00325351	0.08443111
RASSF7	352.287673	-0.9285615	0.00324537	0.08443111
ITGB1BP2	12.7707951	1.14155843	0.00327422	0.08484173
HECA	1457.14026	0.42531012	0.00327924	0.08484535
CALCOCO1	3899.10098	0.54900779	0.0032992	0.08514155
HNRNPUL1	2907.41128	-0.2625461	0.00330048	0.08514155
FAM171A2	207.695354	-0.6127328	0.00331773	0.08545982
CLEC10A	32.7795779	1.38809144	0.0033321	0.08570309
AAED1	98.9985597	0.56937638	0.00334801	0.08594263
UBE2Z	1682.28508	-0.3603113	0.0033513	0.08594263
CCNE2	39.760047	-1.0609124	0.00337062	0.08599824
CD3G	5.46230276	1.30502635	0.00336827	0.08599824
RUNX2	265.948576	1.27665222	0.00335895	0.08599824
SASH1	805.735521	1.07803443	0.00337326	0.08599824
TNXB	12.0008569	-1.1965942	0.00338592	0.08619481
SLC24A2	11.2619786	-1.3863819	0.00340455	0.08654233
CMPK1	1211.37932	0.54167863	0.00343607	0.08666451
IDH3G	515.944883	-0.3059609	0.00343463	0.08666451
IFIT3	530.199371	0.62529397	0.00343811	0.08666451
IL20RA	42.8323099	1.34818294	0.00343103	0.08666451
POLQ	59.7926793	-1.0443231	0.00343996	0.08666451
SLC24A5	13.0960738	1.37054102	0.00344007	0.08666451
TRPC2	3.53317512	1.33216754	0.00344425	0.08666451
GLP2R	21.5659436	1.38258272	0.00345349	0.08668449
HIST1H2AM	119.560927	-0.8223041	0.00345715	0.08668449
RPL36A	405.479259	0.56324071	0.00346	0.08668449
PLCD3	2177.10844	-0.5883187	0.00347108	0.08683695
SAMD1	109.680849	-0.5717205	0.00350317	0.08751376

FUS	2215.03512	-0.8439204	0.00352898	0.088032
PSKH1	672.598954	-0.5169536	0.00356381	0.08877358
ID4	1605.00303	-1.1767496	0.00357751	0.08898728
VGF	2.0483029	-1.3279221	0.00358401	0.08902169
GALM	189.510216	1.0131004	0.00359825	0.08924795
CST7	13.0451121	1.31510239	0.00360508	0.08928994
GPR174	4.90680508	1.25854859	0.00361492	0.08940616
SUCNR1	10.02809	1.36598692	0.00362791	0.08959999
ZNF503	908.66972	-1.0154582	0.0036488	0.08998809
PODXL2	142.016895	1.33340091	0.00366331	0.09021818
PLA2G3	33.5467988	-1.3823094	0.00367033	0.09026305
HSD17B12	1341.13429	-0.4499659	0.00368035	0.09038169
ATP6V0E2	503.754585	-0.7985195	0.00369075	0.09044757
EPSTI1	142.496041	0.79493116	0.00370143	0.09044757
PCDHGB8P	274.578245	-0.8640492	0.00370009	0.09044757
PRR14	280.130115	-0.4909005	0.00370384	0.09044757
COX5A	453.453775	-0.8178721	0.00372413	0.09077033
CREBRF	1557.29524	0.35863574	0.0037358	0.09077033
FAM198A	1450.86288	1.12882921	0.00373794	0.09077033
INMT-FAM1[illegible]	6.67703785	1.37255922	0.00373676	0.09077033
C9orf129	15.0691183	-1.3049409	0.00375136	0.09096928
CCKBR	16.4530412	-1.3501218	0.00376659	0.09108453
SYTL2	267.170501	1.07998979	0.00376488	0.09108453
PRR18	2.65289822	-1.3670338	0.00378194	0.09125903
USP42	373.157657	-0.445877	0.00378431	0.09125903
LOC653602	11.5032088	-1.2870678	0.0038232	0.09187803
SIDT1	79.5899168	-1.2443386	0.00382583	0.09187803
ZFP2	42.5589296	0.95644595	0.00382516	0.09187803
TAF1A-AS1	12.2930768	-0.8422873	0.00383898	0.0920668
GRIA2	5.66403225	-1.3504063	0.00385361	0.09229017
C4orf48	50.46995	-1.0394095	0.00386071	0.09230963
GTF2E1	184.989679	-0.3986459	0.00386504	0.09230963
ATP6AP1	1798.06851	-0.3601205	0.00388101	0.09256394
NOB1	440.656963	-0.5674025	0.00390421	0.09286253
PYHIN1	5.88669689	1.27225648	0.00390263	0.09286253
ITPKA	8.21757623	-1.3086315	0.00392464	0.09309379
SHROOM3	273.709548	1.27792761	0.00391957	0.09309379
CYTIP	101.521928	1.01908808	0.00393614	0.09316253
PCNXL3	1260.3133	-0.5845613	0.00394328	0.09316253
TF	730.73347	-1.3558665	0.00394361	0.09316253
NAALAD2	44.8591939	1.32687954	0.00397397	0.09375218
PSMG2	406.714032	0.53570898	0.00399063	0.09401773
IDUA	295.528376	-0.7385885	0.00400304	0.0941824
OLFM2	157.844652	1.12608149	0.00402028	0.0944603
LHCGR	142.98527	1.2487511	0.00403972	0.09478899
EIF4A2	4320.54712	0.54849423	0.0040527	0.09486458
OTOA	5.26343811	1.26644498	0.00405854	0.09486458
RBM4B	247.045248	-0.3756326	0.00405931	0.09486458
SEZ6L2	448.267581	-1.2531142	0.00407745	0.09516053
NCL	4740.46615	-0.4899548	0.0040945	0.0953128

ZNF57	112.741913	-1.0868014	0.00409494	0.0953128
FREM2	3030.62725	-1.3306465	0.00411055	0.09542136
TPI1P2	12.8044472	-1.0255647	0.00411058	0.09542136
CSNK1D	1993.55491	-0.6084116	0.00412326	0.09558818
C10orf10	243.232188	1.11453191	0.00413001	0.09561722
GZMH	6.04943231	1.32958897	0.00415188	0.09599559
PLEC	20519.2094	-0.6021258	0.00416452	0.09615995
HPCAL4	42.9840433	1.33591051	0.0041898	0.09635979
RBPMS2	241.637265	-1.0675055	0.00418866	0.09635979
TRAF3IP3	50.9482677	1.13753488	0.00418062	0.09635979
CENPA	6.51095311	-1.2550075	0.00429596	0.09765759
EBF3	281.523312	1.28726677	0.00429588	0.09765759
FANCA	96.1499422	-0.7824018	0.00429168	0.09765759
LOC1002890	129.03819	-0.8358688	0.00430069	0.09765759
NRK	8.84799623	1.24863715	0.00426267	0.09765759
PAK4	705.48698	-0.6534323	0.00430239	0.09765759
PSD	25.7075168	-1.1333507	0.00428361	0.09765759
RMI1	90.5334681	-0.5231139	0.00428493	0.09765759
RPL23AP32	121.91716	0.95907969	0.00429187	0.09765759
STC2	101.460474	-1.3168167	0.00425788	0.09765759
KHNYN	1185.50239	0.55421798	0.00431651	0.09785026
NUP214	1494.87086	-0.3247347	0.00434466	0.09836006
LOC728730	48.4954003	0.76577097	0.00435822	0.09853874
THAP2	80.3221008	0.59497368	0.00437587	0.09880935
SOX12	219.660189	-0.7791785	0.00440045	0.09923549
ASPRV1	43.711272	1.06956728	0.00441704	0.09935197
PPAPDC1A	14.4831348	1.34866952	0.0044152	0.09935197
ZCCHC14	1034.69885	-0.6036831	0.00444504	0.0998527
MFGE8	758.063176	1.0550793	0.00445997	0.10005867
GNG12	1124.33516	0.78388498	0.00448778	0.10020966
GPATCH8	2215.65479	-0.4048219	0.00448975	0.10020966
KIAA1217	3649.44134	-0.5588894	0.00448832	0.10020966
TMEM19	577.677939	0.60979725	0.00448277	0.10020966
PTGES2	450.224357	-0.6208874	0.00451738	0.10069706
LCP1	895.643789	1.04196259	0.00455058	0.10113551
9-Mar	155.784313	-0.4828311	0.0045545	0.10113551
SLAMF1	3.38253024	1.34069148	0.00455233	0.10113551
APLP1	318.548823	-1.102055	0.00458322	0.10164351
COQ9	612.114644	-0.7822085	0.00461679	0.10225748
BCL2	701.365488	1.11852604	0.00462466	0.10226056
CADM3	26.860274	-1.3343959	0.004637	0.10226056
GPD1L	636.862154	0.84842188	0.00464045	0.10226056
PRCC	538.9565	-0.4131572	0.00462965	0.10226056
HRH2	28.7239976	1.0449794	0.00468217	0.10292879
LAMTOR4	408.633587	0.42179319	0.00468262	0.10292879
AGPS	784.295824	0.70086883	0.00469324	0.10303212
TMEM134	137.386849	-0.4683123	0.0047117	0.10330684
PCDHGA11	449.109429	-0.897999	0.00472013	0.10336128
KMT2B	1216.85744	-0.4128647	0.00474138	0.10369602
CHCHD10	76.4417125	-0.9663219	0.00476346	0.10395058

IGIP	472.350334	0.47838994	0.00476497	0.10395058
BCORL1	347.68527	-0.7134923	0.00477747	0.1039942
MMD2	2.73898484	-1.2920363	0.00478442	0.1039942
USP22	5176.53062	-0.2812405	0.00478492	0.1039942
SPTBN2	314.857273	-1.0758415	0.00479935	0.10417761
NXPH2	346.296907	1.3082155	0.00481346	0.10435368
CD244	6.75949211	1.24172253	0.00482672	0.10451077
XAF1	630.808799	0.69243886	0.00483993	0.10453649
ZNF768	372.819997	-0.3619769	0.00483573	0.10453649
CHERP	615.26542	-0.5354948	0.00487586	0.10492151
FAM180A	111.783528	1.33502251	0.00487467	0.10492151
8-Sep	1246.53147	0.56214788	0.00487136	0.10492151
CDKN2A	39.6918372	-1.2549045	0.00488681	0.1050271
LINC00486	1.87087957	1.33780617	0.00489709	0.10511824
DDIT4	2895.69408	1.05000609	0.00490877	0.10523898
C16orf54	21.8968587	1.0181682	0.0049451	0.10587327
RAB23	873.865324	0.67711701	0.00495054	0.10587327
P2RX7	238.095556	0.8629675	0.00496341	0.1060181
ESPL1	55.2829507	-0.9208443	0.00506671	0.10753061
FOXK2	777.601811	-0.4602529	0.00507132	0.10753061
IFIT2	350.308076	0.69711553	0.00506403	0.10753061
IL2RG	29.1428043	1.15862859	0.00505584	0.10753061
PRX	426.820265	1.06301298	0.00506996	0.10753061
RAPGEF6	1223.6652	0.41621812	0.0050516	0.10753061
ASIC3	41.1661982	-0.9157001	0.00508512	0.10756072
ZNF18	216.949839	0.47292197	0.00508182	0.10756072
PCDHB16	640.45772	-1.1235345	0.00510399	0.10782874
IMMT	1121.14307	-0.3892911	0.00511295	0.10788697
FAM120A	3865.91049	-0.335353	0.00512623	0.10800046
SYN1	31.5551233	-0.9108988	0.00513075	0.10800046
SLC14A2	9.8875767	1.26640764	0.00514288	0.10812489
ZNF570	138.862007	0.53727532	0.00514921	0.10812715
EPAS1	8965.43384	-1.038519	0.00516737	0.10837758
ABCG4	20.9313267	-1.185688	0.00522017	0.10915151
DKFZp779M(	1.88158929	1.31441103	0.00523566	0.10915151
NDUFA8	294.843364	-0.5730603	0.00523501	0.10915151
RSPO2	96.9009835	-1.2903398	0.00522162	0.10915151
SERPINE2	289.130712	-1.0999768	0.00522731	0.10915151
COLCA2	57.5554105	-1.1969528	0.00524314	0.10917662
USP45	276.8965	0.52214992	0.00525615	0.10931657
CCNA2	62.2279533	-0.8279537	0.00527287	0.10953327
CNNM3	400.623147	-0.4606211	0.00528463	0.10954615
CYSLTR2	11.4243506	1.16622611	0.00529239	0.10954615
MEA1	377.58335	-0.4474876	0.00529057	0.10954615
LMNB1	162.734565	-0.7608415	0.00530945	0.10976857
AIM2	8.35258149	1.29162715	0.00533892	0.11011596
POLN	67.1782299	0.65392212	0.00533458	0.11011596
DPH6-AS1	19.7011596	0.86084924	0.0053617	0.11040633
HILPDA	211.790283	1.17077693	0.00538093	0.11040633
LOC440300	118.954683	0.86951248	0.00540045	0.11040633

MTERFD2	480.148397	-0.4098536	0.0053928	0.11040633
P2RY2	8.90017722	1.20914232	0.00541015	0.11040633
PCDHB9	296.550381	-1.1419611	0.00539153	0.11040633
SAMD10	51.4236982	-0.9513371	0.00536872	0.11040633
SAP30BP	922.511839	-0.660785	0.00540956	0.11040633
TM4SF1	689.67252	0.9264365	0.005409	0.11040633
RPS6KA5	71.2122553	0.80317097	0.00542696	0.11061964
JPH2	70.2374291	-1.0424023	0.00543947	0.1106659
TSPAN2	25.4463665	-1.290619	0.00544196	0.1106659
TEAD2	260.734017	-0.6942381	0.00545571	0.11081596
MGAM	114.952898	1.05954637	0.00546909	0.11095808
RPRM	64.6575887	-1.3172638	0.0055002	0.11145916
PTGES	171.819563	-1.1193807	0.0055105	0.11153787
ZBED8	204.412699	0.59044184	0.00554697	0.11214553
IGSF9	60.8109669	-1.1343686	0.00555822	0.11221927
TMC8	132.129391	0.86910131	0.00556352	0.11221927
CSNK2A2	389.725445	-0.4194468	0.00559269	0.11267682
CD164	4157.21805	0.45226273	0.0056432	0.11330052
DCHS2	387.54053	-1.2765581	0.00563371	0.11330052
VSIG1	10.0069903	-1.2096089	0.00564283	0.11330052
CDH26	16.533001	0.99882668	0.00565654	0.11330666
SLC24A1	436.639499	-0.52246	0.00565353	0.11330666
ZFPM1	116.774994	-0.697804	0.00566801	0.11340588
LINC00158	10.3037477	1.21986217	0.00567892	0.11349355
ABCA3	1903.17601	-0.7440868	0.00570596	0.11351206
ARHGAP15	135.947087	0.9127107	0.00570278	0.11351206
KIAA0825	254.420614	0.76807855	0.00569738	0.11351206
TNR	2.36313147	-1.3058055	0.00570409	0.11351206
CLEC9A	69.2180326	1.28671649	0.00573142	0.11388814
DGKD	809.634719	-0.6830989	0.0057419	0.11396631
SCG2	249.680883	1.29659144	0.0057674	0.11421154
TBPL1	122.579651	0.51979301	0.00576343	0.11421154
ADAMTS16	6.5761487	-1.2413322	0.00581761	0.11451342
GAB2	903.092845	0.74202023	0.00582656	0.11451342
LINC00612	7.86485129	-1.0685544	0.00580084	0.11451342
MCM4	736.629338	-0.824	0.00579772	0.11451342
MSI2	1566.21724	-0.5799445	0.00582874	0.11451342
PTH2R	16.2332361	-1.297308	0.00582501	0.11451342
TMEM127	1275.38136	-0.3693338	0.00582716	0.11451342
ANGPTL5	34.9815915	1.30136447	0.00584291	0.1146622
NCKAP5	140.11651	0.90796442	0.00585413	0.11475285
ERI1	227.88476	0.4088752	0.00586857	0.11490627
ZC3H18	537.640961	-0.3594875	0.00587648	0.11493174
C16orf59	10.8639897	-1.0564613	0.00595265	0.1161602
OR56B1	13.0455618	1.12193681	0.00594977	0.1161602
AURKB	15.489296	-0.9380534	0.00598536	0.11666751
SSPN	529.500125	1.20436378	0.00601171	0.11693842
STXBP5	607.609627	0.7734594	0.00601271	0.11693842
ADAM28	330.969652	0.98504708	0.00605142	0.11755988
TNFAIP3	1303.11517	0.91943125	0.00606212	0.11763621

NCK2	1182.03887	-0.6160038	0.00607746	0.11773568
ZCCHC10	167.848614	0.50440645	0.00608079	0.11773568
C10orf85	2.35309528	1.25227533	0.00609889	0.11781092
RTN3	2106.90373	-0.445048	0.00610426	0.11781092
VIT	472.815338	1.16716167	0.006105	0.11781092
LOC80154	15.275874	-0.9209917	0.00612539	0.11794144
LRRC38	84.8316681	-1.2096145	0.00613211	0.11794144
NUDT9P1	12.182018	1.11902339	0.00612512	0.11794144
ABTB2	178.356892	-0.9874243	0.00619667	0.11803671
CD2	20.5167263	1.19579119	0.00620495	0.11803671
FAM110A	53.7685845	-0.602084	0.00618416	0.11803671
FHOD3	26.0532854	1.25822977	0.00617617	0.11803671
MLST8	348.33947	-0.5208741	0.00615805	0.11803671
MMP16	407.674189	1.27261281	0.00619083	0.11803671
OTUB1	600.267761	-0.4201807	0.00619491	0.11803671
RNF152	145.137445	1.15362223	0.00619869	0.11803671
SLA	591.956149	0.95660755	0.00618925	0.11803671
SNX10	161.459496	0.71043009	0.00615488	0.11803671
PTTG2	4.36363997	1.23679387	0.0062355	0.11836114
RFX1	218.417854	-0.4747616	0.00623562	0.11836114
DNAJC4	307.652101	-0.5738854	0.00628934	0.11847679
GATAD2B	1292.67104	-0.3466513	0.00628095	0.11847679
IGF1	723.459871	1.22660554	0.00629623	0.11847679
LPHN3	353.719364	-1.2940989	0.0062911	0.11847679
LYZ	501.312717	1.09626237	0.00626862	0.11847679
OLFM1	100.559586	-1.1345331	0.00626494	0.11847679
TOP3B	145.461937	0.50033069	0.00626544	0.11847679
TP53	788.070542	-0.5509043	0.00628751	0.11847679
FUBP3	980.00338	-0.2868961	0.00632462	0.11862582
H19	11697.126	-1.2805468	0.00632349	0.11862582
LINC00673	6.43395397	-1.2989312	0.00632235	0.11862582
NCAPG	87.0945539	-1.0358286	0.00634593	0.11889737
R3HDM1	525.237213	-0.3387705	0.00635953	0.11902384
PLEKHG4B	1996.59081	-0.9760627	0.00637224	0.11913353
KIF18A	24.6699473	-1.0471954	0.00638741	0.11928889
PRSS23	656.027551	1.05080657	0.00641003	0.11958286
PCDHGB3	462.604847	-0.9448892	0.00645268	0.12024954
GPX3	827.669826	1.12876419	0.00648729	0.12045754
HACE1	295.693129	0.56288507	0.00648971	0.12045754
LIMS2	441.766677	-0.7886258	0.0065039	0.12045754
LOC1005066	72.5561853	-1.2811276	0.00650541	0.12045754
MGAT4A	1196.00013	0.71537453	0.00650147	0.12045754
ZNF142	735.902888	-0.369913	0.00647722	0.12045754
ATP8B4	380.115258	0.96354073	0.00652032	0.12047703
DCTPP1	172.045757	-0.5033527	0.00651618	0.12047703
PMP2	19.5590563	-1.2436976	0.00652807	0.12049211
LOC642423	2.41758694	-1.1841047	0.00655838	0.12075431
POLR1B	600.777877	-0.5906282	0.00655334	0.12075431
ZNF438	202.171733	0.5777919	0.00656311	0.12075431
TEX30	36.9589824	0.63802349	0.00660547	0.12140526

ATE1-AS1	13.2990628	1.26282719	0.00661645	0.12147851
BLOC1S2	265.531384	0.49885858	0.00664131	0.12167808
CASC5	150.495053	-0.9325687	0.00664018	0.12167808
CDK16	1051.10514	-0.4033456	0.00665053	0.12171872
NPIPB3	254.212693	-0.7601431	0.0066629	0.12181684
CD3D	6.53565323	1.25101186	0.00667101	0.12183699
LINC00607	170.570491	1.09949617	0.00670085	0.12218723
MMP15	203.928607	-0.8983571	0.00670424	0.12218723
CD8A	23.3407866	1.18436888	0.00673787	0.12267164
MALT1	580.725957	0.55946842	0.00676187	0.12272297
NAGPA	113.366373	0.33631021	0.00675975	0.12272297
PCDHGA6	598.709092	-0.7448074	0.00675397	0.12272297
HORMAD1	2.75954033	1.28482994	0.00678803	0.12306927
CNDP1	5.00845455	-1.2665686	0.00679728	0.12310863
RNF26	418.759799	-0.5994232	0.00680892	0.12319123
CDC6	64.5634376	-0.8318732	0.00683123	0.12326245
DCC	266.139958	1.19555014	0.00684122	0.12326245
FAM189B	323.410998	-0.6890702	0.00683319	0.12326245
SNORA74A	170.512487	-0.9074401	0.00683504	0.12326245
WDR5	489.82047	-0.2830949	0.00686174	0.12350426
HIATL2	58.5729647	-0.5809169	0.00687752	0.12366024
LRFN4	141.689137	-0.8728522	0.00690232	0.12397798
C11orf35	45.501862	-0.7281417	0.0069227	0.12421568
LOC1019270	36.6055844	1.19545066	0.00694226	0.12443814
A2ML1	2.63978252	1.24157641	0.00702184	0.12530216
MTNR1A	10.2845264	-1.2173543	0.00701866	0.12530216
PIWIL4	45.2329739	0.65993807	0.00702649	0.12530216
RCN3	359.859744	1.02935622	0.00702551	0.12530216
TMX1	506.620155	0.42558387	0.00700728	0.12530216
ARMC7	203.379469	-0.7439774	0.00705267	0.12551149
MORC3	1074.32393	0.42540844	0.00704716	0.12551149
MATN2	805.827523	1.23561188	0.00707959	0.12573325
RCBTB2	929.11056	0.48202489	0.00707236	0.12573325
SLC5A12	5.64099751	1.27475693	0.00713017	0.12624474
TMEM8A	558.729079	-0.6107606	0.00712958	0.12624474
WNT10A	10.0492106	-1.2590329	0.00712748	0.12624474
FAR2	127.656723	0.9177934	0.0071533	0.12652527
ELFN1	212.645431	-1.2085464	0.00719331	0.12671743
LOC1005061	10.001516	1.08131146	0.00719059	0.12671743
RASL10A	4.40295805	-1.2075424	0.00717934	0.12671743
TERF2	406.564654	-0.4940296	0.00717192	0.12671743
GFRA1	1393.35114	1.23393058	0.00720843	0.12683542
NAV3	200.451222	1.26969451	0.0072146	0.12683542
ASCL4	2.40084663	1.26057602	0.0072332	0.12690587
LINC01277	1.94798293	1.22732923	0.00722757	0.12690587
ADAMTS8	4.76093005	-1.2685834	0.00725162	0.12710071
IFT20	214.647391	0.46168129	0.00726776	0.12725527
CD226	19.9320982	0.95534324	0.00729432	0.12733576
NAIP	365.861178	0.55951946	0.00729065	0.12733576
TNFRSF11B	648.656867	-1.2363194	0.00728641	0.12733576

ACACB	1032.8495	-0.5171263	0.00733899	0.12795958
SRCAP	2795.47514	-0.432962	0.00734478	0.12795958
CSF2RB	131.202943	0.86669293	0.00736768	0.12810181
PDK4	1431.76572	1.06665065	0.00736271	0.12810181
CCRL2	15.194304	0.94395075	0.00737744	0.12814347
FAM69B	253.193655	-0.5439148	0.00741349	0.12864115
AACS	483.406284	-0.5965074	0.00745658	0.12913102
OTOG	600.368587	-1.2638118	0.00745616	0.12913102
AGRN	4540.16447	-0.7400086	0.00749003	0.129151
C5orf56	44.8754375	0.70131306	0.0075023	0.129151
FIZ1	139.027269	-0.6284904	0.00747651	0.129151
SPIN2B	70.731814	-0.5707647	0.0074887	0.129151
SSRP1	1349.71949	-0.4118005	0.00747784	0.129151
ZBTB14	251.090396	0.42361669	0.00749715	0.129151
CALN1	4.82881805	-1.2276631	0.00751965	0.12923712
LINC00663	90.9470634	0.49825546	0.00752217	0.12923712
ZNF487	52.5235142	0.80039227	0.00755204	0.12962227
DSCAML1	15.8013186	-1.2428101	0.00758309	0.13001289
OSGEPL1-AS	10.0894108	1.0120173	0.00758976	0.13001289
SF3B4	474.43826	-0.4951451	0.00762277	0.13044984
C19orf84	1.90016798	-1.2543399	0.00763224	0.13048351
CCNF	61.6376615	-0.6631208	0.00764277	0.13053513
ZEB1	1789.54006	0.63422753	0.00772498	0.13180983
CTXN1	57.2517326	-1.0887509	0.00773316	0.1318201
ADCY10P1	86.0881013	1.03187122	0.00776065	0.13215903
LINC00528	2.89570624	1.25138309	0.0077701	0.13219047
OSBP	1392.33471	-0.3905737	0.00780236	0.13260955
CMTM2	4.17466554	1.22634993	0.00788256	0.13332605
IMPACT	603.019098	0.61377123	0.0078796	0.13332605
NME9	4.58331902	1.2634046	0.00791305	0.13332605
PHYHIPL	9.97701071	-1.2387324	0.00790897	0.13332605
PPP1R16A	290.950376	-0.5325679	0.0078895	0.13332605
TMEM255A	28.2194144	-1.2463335	0.00787067	0.13332605
TOX	94.3178186	-1.2565958	0.00789384	0.13332605
XRCC1	404.862327	-0.3053093	0.00786446	0.13332605
ZNF629	897.76587	-0.3294286	0.00791353	0.13332605
CCR10	15.5168054	-1.1725094	0.00792871	0.13345256
SOX8	16.6782149	-1.0738841	0.00795956	0.13384226
AMIGO2	45.251571	0.90295222	0.00801482	0.13451123
TAC3	52.5196817	-1.2420754	0.00801405	0.13451123
CERS5	623.365968	-0.4720995	0.00805085	0.13498571
ACACA	1985.95449	-0.333319	0.00810385	0.13533125
LCA5	110.271634	0.78454272	0.00810826	0.13533125
LOC283038	5.15195719	-1.2385322	0.00808096	0.13533125
STAT2	3379.68105	0.48046068	0.00811038	0.13533125
SYT1	14.2163311	-1.2320904	0.00810926	0.13533125
ABHD12B	1.78657509	-1.2584342	0.00813844	0.13550442
COMT	579.639681	0.46346936	0.00814414	0.13550442
ZWINT	73.834579	-0.8265865	0.0081383	0.13550442
MYO5C	992.074197	-0.8441486	0.00817133	0.13569651

NRTN	3.07197564	-1.2189326	0.0081709	0.13569651
RHPN1	108.200868	-0.9780445	0.00818555	0.13569651
TCF3	695.671983	-0.4767267	0.00819471	0.13569651
TMEM246	1191.17467	-0.5941036	0.00819442	0.13569651
CC2D2B	8.979964	1.02297914	0.00820294	0.13570363
MYO1E	701.839052	0.88139205	0.00821526	0.13577818
KLHL6	154.938294	0.91341376	0.00822763	0.13585352
LTK	11.7758143	-1.2525205	0.00824904	0.1360778
MED8	239.105288	0.60455151	0.00826121	0.13614942
SERPINB9	691.380238	0.76983432	0.00828809	0.13633406
SLC25A39	920.928433	-0.4207606	0.00828125	0.13633406
ANKRD52	1604.22751	-0.6040626	0.00832676	0.13634446
ARHGDIA	2807.66185	-0.4793724	0.00830878	0.13634446
LIG1	391.633325	-0.6101171	0.00830048	0.13634446
PKDREJ	5.86225089	1.236513	0.00831613	0.13634446
SNORA34	41.5526932	0.91718881	0.00832794	0.13634446
AKAP1	920.791321	-0.605876	0.00836439	0.1366703
CXorf22	12.3686689	-1.252552	0.00837537	0.1366703
KIRREL3	3.02992025	-1.2051763	0.00837001	0.1366703
KLK10	98.6965964	-1.1846132	0.00837928	0.1366703
BBS9	726.939438	0.8690632	0.00840578	0.13671781
MRO	344.37948	-1.0377046	0.00839911	0.13671781
ZNF513	239.474864	-0.4504987	0.00839638	0.13671781
TRPC1	648.915532	0.61637573	0.00844126	0.13716645
HSPA6	99.5555297	0.97225371	0.00849793	0.13782983
WHSC1	1173.76822	-0.4007102	0.00849416	0.13782983
TLL1	84.0375218	-1.2228575	0.00851511	0.13797976
MRPS24	23.0313649	-0.8284015	0.00853739	0.13821187
RHOBTB3	933.686218	1.20188183	0.00859549	0.13902313
GANAB	6021.59322	-0.397464	0.0086241	0.13935615
RPS29	1214.40755	0.65134405	0.00864763	0.13947716
ZIC2	2732.40827	-0.5978106	0.00863966	0.13947716
COL9A2	71.3719431	1.15699009	0.00866411	0.13961349
IRF5	217.029081	0.88760338	0.00868552	0.13969945
PTPRC	1067.6525	0.89063111	0.00867985	0.13969945
SLC26A4	14.1547574	1.09411195	0.00871211	0.13999768
CBFA2T2	1468.91879	-0.5168188	0.00872681	0.14010438
COX7B	787.053174	-0.6433588	0.00875203	0.14037971
NEAT1	27049.2006	-0.7499036	0.00879134	0.14075052
SYVN1	756.847702	-0.5138263	0.00878753	0.14075052
CGNL1	2733.14815	1.08445893	0.00880172	0.140787
GUCA1B	24.1658682	0.80902271	0.00881775	0.14091382
PDIK1L	139.561963	0.50374927	0.00883795	0.1411069
C1orf123	273.464227	0.54807778	0.00887196	0.1413902
ITGB5	1197.03985	0.84883004	0.00886882	0.1413902
KIAA0195	1108.76507	-0.3479974	0.00888683	0.14145614
SETD1B	942.427482	-0.4413795	0.00889237	0.14145614
DPY19L1P1	49.5023723	0.7604294	0.00890123	0.14146776
CD96	23.0915027	1.02569978	0.00895374	0.14217227
TSPO	192.828854	0.84838253	0.00896365	0.14219972

MUC1	209.220032	-0.8061194	0.00899491	0.1425656
SAYSD1	143.171344	0.53104721	0.00900585	0.14260909
MED14OS	3.24563843	1.03746409	0.00910591	0.14406232
FAM222A	14.1037936	-0.8737198	0.00912369	0.14414267
LOC1019287	6.25394951	1.23804872	0.00912757	0.14414267
PSTPIP2	158.05915	0.64807259	0.00914732	0.14432345
NME8	5.61397612	1.16151762	0.00915912	0.14437862
HSPA1B	31.9372676	0.93259314	0.00919586	0.14454216
PSAP	15814.4941	0.49509876	0.00918885	0.14454216
TNFSF8	119.722889	0.95757694	0.00920275	0.14454216
ZNF780A	570.767845	0.43642562	0.00919377	0.14454216
SYNPO2	2277.60722	1.17000059	0.00922969	0.1447068
TCF7L1	509.093607	-0.6008722	0.00922988	0.1447068
ADRBK2	376.385221	0.7245664	0.00929095	0.14529075
CCDC23	103.85524	0.66036132	0.00929219	0.14529075
MAEL	7.39286542	1.23772284	0.00928513	0.14529075
MX2	110.948321	0.89934805	0.00930755	0.14540008
COX10	195.490373	-0.5343056	0.00933449	0.14569004
KIAA1467	363.547678	0.63035908	0.00935126	0.14576845
MED25	368.79228	-0.3885898	0.00936228	0.14576845
SPAG8	43.704786	0.7443603	0.00936466	0.14576845
REPIN1	846.007974	-0.551974	0.00938423	0.14594247
EPM2A	115.151749	0.53708555	0.00939609	0.14599627
NR2F1-AS1	360.606317	0.99270862	0.00942355	0.14603148
POM121	954.135389	-0.4639764	0.00941752	0.14603148
SNORA80A	21.4710247	1.15526451	0.00941588	0.14603148
NAP1L4	1395.83371	-0.2649872	0.00945631	0.14640863
PPFIBP2	363.370161	0.84928573	0.00951349	0.1471628
RNFT2	106.631705	-1.1211275	0.00952701	0.14724093
COG5	1033.59123	0.33118998	0.00956893	0.14738377
EIF1B-AS1	18.8354416	0.9307276	0.00956172	0.14738377
HGF	98.1869171	1.04708174	0.00958325	0.14738377
IL10	8.51333969	1.22315111	0.00958711	0.14738377
RNU6ATAC	11.5811961	1.14521201	0.00955055	0.14738377
SPN	96.904883	1.08100549	0.00958071	0.14738377
APOL6	900.663947	0.54092724	0.00963193	0.14794197
PPAN	29.5318206	0.68464434	0.00970686	0.14896126
ST6GALNAC4	355.12604	-0.7534811	0.00971969	0.14902669
TIGD5	97.5833098	-0.5079671	0.00974744	0.1493205
SPC24	18.2866519	-0.910083	0.00976998	0.149534
LLGL2	262.352419	-0.9187091	0.00977867	0.14953535
AQP1	2128.3035	1.21868593	0.00981966	0.14983623
C1orf116	3.54996246	-1.1500501	0.00983281	0.14983623
RPL9	5156.65453	0.4408049	0.00982102	0.14983623
ZDHHC24	126.346543	-0.5556511	0.00982512	0.14983623
FGF12	46.6268966	1.02724557	0.00989242	0.15061258
MYOC	2.10422338	1.21435788	0.00992219	0.15093367
CCDC86	189.26437	-0.5911012	0.00994949	0.15111762
CSNK1A1L	2.30904193	-1.1787599	0.00995448	0.15111762
MPP6	1249.39495	0.93218699	0.00997735	0.15111762

SETD5	2641.38139	-0.3617846	0.00996862	0.15111762
TRAP1	773.178921	-0.5460188	0.00997774	0.15111762
ZNF688	114.765813	0.48348667	0.00998882	0.15115378
COL23A1	47.1400289	1.22272646	0.01001025	0.15121477
SNHG17	99.7567226	-0.6535967	0.0100051	0.15121477
CCDC78	22.7071517	-0.9608463	0.01008316	0.15128527
HCFC1	2466.20267	-0.3690178	0.01011933	0.15128527
HECTD4	5405.77221	-0.4466255	0.01007055	0.15128527
LARP1	4796.76754	-0.4488413	0.01010172	0.15128527
LOC151475	10.885035	1.20900978	0.01003731	0.15128527
MEF2BNB	103.826471	-0.4205329	0.01006077	0.15128527
NXT2	120.969605	0.58872075	0.01005334	0.15128527
P2RY14	67.5285175	1.20230807	0.01011682	0.15128527
SCD	1827.3229	-1.0532735	0.01010302	0.15128527
TERC	611.114088	-0.7976235	0.01004399	0.15128527
TJP3	43.9346563	-1.1170133	0.01007351	0.15128527
VENTX	137.081066	1.0894644	0.01008677	0.15128527
ANGPTL7	122.80334	1.08941026	0.01015902	0.15174822
GALR1	4.29222708	-1.151156	0.01017377	0.15183802
ERCC6L	12.7929458	-0.9415923	0.01019209	0.15198103
LAMC2	62.4795192	-1.100929	0.01022459	0.15207437
SMIM14	684.613848	0.59700162	0.01022454	0.15207437
UBE2Q2P2	4.73681831	-1.072153	0.01021387	0.15207437
ZMYND15	91.602034	0.85348539	0.01027697	0.15272276
FBXO48	13.4513328	0.83773032	0.01031617	0.15317441
WDR11-AS1	6.33752247	1.11688337	0.01033548	0.15319941
ZMYM6NB	161.763544	0.5694422	0.01033251	0.15319941
RASGRP1	27.7195293	1.03072096	0.01035532	0.15336288
SLITRK5	5.0040401	-1.2021901	0.01036498	0.15337519
BIRC2	1165.11445	0.30522632	0.01039417	0.15351971
BLM	42.6677178	-0.920692	0.01038958	0.15351971
EPS8L2	657.689184	-0.7194229	0.01041737	0.15351971
PLCB1	864.266921	1.17265443	0.01042298	0.15351971
PMVK	229.200788	-0.585291	0.01040994	0.15351971
RANBP10	491.958005	-0.474661	0.01042772	0.15351971
RAP2C-AS1	75.4980512	0.65080201	0.01048605	0.15424779
CHEK1	74.5853897	-0.7613636	0.01059425	0.15457468
CYP4Z1	99.7157804	0.92687073	0.01055841	0.15457468
MOB3B	217.786822	1.0763301	0.01057669	0.15457468
NPLOC4	1446.5964	-0.3745012	0.01061409	0.15457468
PARP14	2544.5395	0.26444493	0.01057179	0.15457468
RAB7A	2604.94738	-0.4128879	0.01061495	0.15457468
RBFADN	7.79072187	1.04768702	0.01058145	0.15457468
RGL1	1499.66731	0.70651893	0.01060265	0.15457468
SIVA1	215.232726	0.44166397	0.01057727	0.15457468
TCP10L	21.628993	0.9209895	0.01055665	0.15457468
XRRA1	436.472486	-0.6900179	0.01059282	0.15457468
ZNF394	328.698202	0.3329492	0.01058426	0.15457468
CD3E	13.8523911	1.03852142	0.01064487	0.15475849
SF1	2377.66236	-0.4302568	0.01064538	0.15475849

FAM87A	1.60410609	1.20068698	0.01067288	0.15489936
PIK3IP1	636.93926	0.77997366	0.01066887	0.15489936
RELL2	70.0476919	-0.924645	0.01068204	0.1549029
CEACAM6	8.00245756	-1.1625295	0.01071192	0.15509559
TRG-AS1	9.62726025	1.05662363	0.01071316	0.15509559
SPATA41	15.7096963	-0.8116362	0.01073622	0.15517093
ZSCAN12	385.420625	-0.3913698	0.01073413	0.15517093
ODF2	740.308082	-0.2989179	0.01077655	0.1554954
ZNF414	101.52742	-0.6629451	0.01077524	0.1554954
BTLA	4.12232135	1.0475676	0.01079275	0.15559997
ANKRA2	276.335794	0.45423869	0.0108377	0.15611857
CH25H	52.2681297	1.11314868	0.01085896	0.15627996
HMBS	116.782842	-0.7950866	0.01086895	0.15627996
SUGP1	347.722632	-0.2980373	0.01087587	0.15627996
C7orf13	47.5905302	-1.0246094	0.01088705	0.1563114
FXYD1	374.310114	0.76412908	0.01095708	0.15633688
LSMEM1	17.7610936	0.80736155	0.01093845	0.15633688
MYOZ1	44.0574581	1.20250401	0.0109686	0.15633688
OSGIN1	21.2605267	1.07297384	0.01094004	0.15633688
SPNS3	20.0993753	1.16988814	0.01093483	0.15633688
SRGN	1619.40024	0.77603455	0.01096975	0.15633688
SUPT6H	2507.18533	-0.2408909	0.01094455	0.15633688
USP18	36.5368904	0.78679219	0.01093318	0.15633688
ZER1	990.879811	-0.2867667	0.01096447	0.15633688
COQ4	359.362809	-0.3926853	0.01099731	0.1566013
HSCB	41.9657516	0.54340226	0.01106356	0.15741582
NDUFA10	818.551784	-0.335348	0.01108989	0.15766144
ESCO2	24.3651731	-0.8856309	0.01112075	0.15784196
SLAMF6	13.7847419	1.09645205	0.01111612	0.15784196
METTL3	392.065755	0.51639387	0.01114658	0.15807963
TP63	932.19699	-1.2017513	0.01117306	0.15832606
IL11RA	421.881555	0.96711979	0.01122369	0.15891399
ACSM5	46.7471534	1.01379097	0.01127412	0.15916827
ADAMTS17	703.936395	-0.9937085	0.01127827	0.15916827
RAB3A	41.6775145	-0.833868	0.01126823	0.15916827
USP53	4851.49661	0.67096267	0.01127581	0.15916827
RICTOR	1326.46633	0.39507509	0.01129243	0.1592388
EIF2B3	189.10403	0.593571	0.01132283	0.15953819
GTF2H5	335.690733	0.44967994	0.01133943	0.15963514
SLFN13	330.636284	-0.8800623	0.01134808	0.15963514
SH3PXD2A	10222.0726	-0.6107023	0.01138662	0.1600478
PHRF1	1270.1884	-0.3435798	0.01142409	0.16044476
TMEM220-A!	2.92202369	1.12152177	0.01143932	0.16052895
SUMO4	33.3619673	0.57298223	0.01145749	0.1606543
CCDC144A	209.782176	-1.1674793	0.01148895	0.16083609
TBC1D8	2765.79114	-0.8473267	0.01148601	0.16083609
CDADC1	144.777075	0.48252786	0.01150337	0.16090838
DOCK7	1010.84801	0.51407094	0.01155963	0.16093371
PDE1B	67.8933641	0.70583663	0.01154858	0.16093371
POM121C	794.854968	-0.4606332	0.01153932	0.16093371

PRRG3	9.26512699	-1.1937274	0.01156072	0.16093371
SRRT	1005.42359	-0.356287	0.01155004	0.16093371
TBX1	14.2111524	-1.1842009	0.01153555	0.16093371
FN3K	132.749316	-0.7115957	0.01158076	0.16095502
TRAPPC2	234.527915	0.49605228	0.01157801	0.16095502
FAM73A	560.649247	0.46539849	0.01163368	0.16117513
GNAI3	817.259588	0.50466268	0.01161791	0.16117513
PAICS	1134.75443	-0.6693982	0.01162983	0.16117513
PRC1	346.44825	-0.8093184	0.01162216	0.16117513
LOC1005065	33.648286	1.02894222	0.01165464	0.16133696
PMPCA	466.159141	-0.3596346	0.01167783	0.16152943
ANP32B	1059.09231	-0.762596	0.0116993	0.16156934
MEX3B	27.7571941	-0.8295444	0.01169045	0.16156934
PAPPA	968.776248	1.17687927	0.01174047	0.16200911
BSDC1	885.18238	0.41551077	0.01177687	0.16238265
CD19	2.36897488	1.1589703	0.01181134	0.16272531
CREG1	1281.58627	0.39779949	0.01183948	0.16272531
HHATL	2.45751574	-1.1399832	0.01183478	0.16272531
SLC7A1	582.253997	-1.1244927	0.01184852	0.16272531
TMSB4X	6304.6274	0.49651132	0.01183071	0.16272531
BVES	138.304628	1.0756916	0.01195284	0.16298159
DEDD	367.232065	-0.2495573	0.01194173	0.16298159
KCNA3	28.7325465	0.98121897	0.01195288	0.16298159
MGRN1	1031.32358	-0.3483598	0.01195301	0.16298159
PCDH10	9.26158839	-1.1896242	0.01194462	0.16298159
PCDHGA7	436.531653	-0.7361225	0.01196092	0.16298159
PGP	290.854353	-0.4747362	0.01191667	0.16298159
RFXAP	156.130525	-0.4746134	0.01194867	0.16298159
SERINC1	5438.76693	0.40410873	0.01192962	0.16298159
TMEM200A	7.68620266	1.10010312	0.01188615	0.16298159
CNPY4	300.649113	0.58384985	0.01198604	0.16300346
TARBP1	1214.5507	0.6109109	0.01197853	0.16300346
UBASH3A	3.37158858	1.19531337	0.01199065	0.16300346
CCDC147	7.2248508	1.04171358	0.01203603	0.16321
CETN4P	1.86146031	1.19388706	0.01205278	0.16321
FRY	722.262343	1.04396862	0.01201559	0.16321
LOC1019291	7.748513	0.79114692	0.01205049	0.16321
PDIA2	16.3686519	1.13365187	0.01203913	0.16321
ANKRD22	125.129426	1.16024775	0.0121089	0.16335125
FAM134A	1384.80764	-0.3972345	0.01209131	0.16335125
PCGF2	463.201876	-0.4234541	0.01208158	0.16335125
STRADA	516.153922	-0.4006634	0.01211134	0.16335125
TPGS1	57.2631202	-0.5869432	0.01211958	0.16335125
ZC2HC1C	151.855844	1.04583252	0.01211307	0.16335125
DVL3	1348.68604	-0.3257433	0.01219926	0.16384216
EMR3	5.56927621	1.1848634	0.01218018	0.16384216
RRP7A	387.24887	0.37716469	0.0121693	0.16384216
SRSF10	82.7831157	0.54623178	0.01220312	0.16384216
TRIM38	879.146358	0.24409589	0.01219279	0.16384216
LOC1027237	2.04884415	1.18821209	0.01221803	0.16391583

AMN1	87.0127806	0.43255944	0.01229942	0.16475785
PIEZO1	4003.02899	-0.5275182	0.01229975	0.16475785
ASGR2	4.16391869	1.10205947	0.01233528	0.16510666
TATDN2	1110.31089	-0.446087	0.01236351	0.16535725
PCDHGC5	193.8909	-0.7208168	0.01241619	0.16593412
SLFN12L	8.12008473	1.02582644	0.01245059	0.16626608
LINC01085	2.11243231	1.18145405	0.01248655	0.16651208
NME2	264.517116	-0.6793119	0.01248817	0.16651208
UNC80	126.894466	-1.1329483	0.01251117	0.16669096
RAB32	131.309679	0.51841132	0.01252566	0.16675619
AFAP1	3280.65422	-0.6747229	0.01258312	0.16688636
CALD1	5665.91243	0.53606676	0.01260275	0.16688636
HIST2H3D	32.2290831	-0.7439196	0.01261222	0.16688636
MKRN7P	20.9857972	0.95096741	0.01258002	0.16688636
SLC6A4	157.633745	1.18515365	0.01260274	0.16688636
STK32A	208.326164	1.17003551	0.01255765	0.16688636
TMSB15A	3.87857309	-1.0908803	0.01257445	0.16688636
ZNF383	122.914451	0.42470869	0.01259339	0.16688636
ELL2	951.448304	0.86084364	0.01264641	0.16694396
ICOS	2.51360007	1.17222437	0.01265746	0.16694396
PPFIBP1	10306.5223	-0.8263218	0.01265085	0.16694396
SCARNA16	118.581361	-0.7471296	0.01265369	0.16694396
SLFNL1	5.7795162	1.0739411	0.01266458	0.16694396
CCDC81	19.4586089	1.04514057	0.01269407	0.16695716
NOTUM	2.68912242	-1.133995	0.01269439	0.16695716
PNPLA2	693.748431	-0.5871226	0.01267938	0.16695716
ACAP2	1959.97349	0.19089727	0.01278611	0.16702634
CASS4	105.668904	1.01611028	0.01276415	0.16702634
GOT1	463.656203	-0.8053855	0.01276444	0.16702634
IRF2BP1	335.756416	-0.3726382	0.01272563	0.16702634
PDE4DIP	1047.9932	0.76007764	0.01274817	0.16702634
PSMC5	848.868551	-0.3936374	0.01277966	0.16702634
PTOV1	1089.25517	-0.2784454	0.01273959	0.16702634
SFRP2	31319.6044	1.07349819	0.01277335	0.16702634
TBKBP1	297.469658	-0.365871	0.01273927	0.16702634
SAMD3	6.43837634	1.14979047	0.01281447	0.16727116
FAM157B	1.93367816	1.18270245	0.01289289	0.16741502
FBLN1	2502.60624	1.18300938	0.01288746	0.16741502
GABRA5	2.5907835	-1.183573	0.01285771	0.16741502
GPAA1	945.770273	-0.4578624	0.01287303	0.16741502
NNT	2073.09638	-0.5039758	0.01288499	0.16741502
TTBK1	7.05324183	-1.1240889	0.01284416	0.16741502
ZDHHC13	163.277339	-0.6608369	0.01288107	0.16741502
PCDHGB4	398.068348	-0.8173902	0.01291803	0.16761627
HIST1H2AD	19.8265023	0.61149708	0.01294306	0.1676907
PMS2	354.769219	-0.3101954	0.01294183	0.1676907
AP2B1	5016.72262	-0.4547853	0.01297701	0.16770265
CSNK2A3	11.6462684	-1.0661971	0.01296282	0.16770265
GLIPR1	718.27556	0.52990127	0.01300884	0.16770265
KCNG2	5.26004572	-1.1805233	0.01300088	0.16770265

POLR3GL	284.987901	0.38339126	0.0130115	0.16770265
SLC16A13	33.7583934	-0.8509023	0.01297273	0.16770265
ZBTB46	532.400721	-0.7449609	0.01298313	0.16770265
RNF133	20.5821498	-0.9660345	0.01305673	0.168161
PAQR6	97.8407192	-1.0232668	0.01307045	0.16821309
DCAF7	2479.18854	-0.4493869	0.01309432	0.16839165
TRAPPC9	739.277489	-0.4148361	0.01310369	0.16839165
ZDHHC5	1656.70847	-0.4558907	0.01312702	0.16856689
C8orf82	175.61262	-0.6325059	0.01314533	0.16867739
HAPLN3	74.358028	1.05226951	0.01320616	0.16896399
HLA-DQA1	403.843569	1.15140411	0.01320654	0.16896399
KMO	19.0951192	0.99114378	0.01319562	0.16896399
POLR1A	1194.32826	-0.3986532	0.01317972	0.16896399
USP43	49.8484437	-0.9964045	0.01322236	0.16904203
C19orf24	171.106667	-0.4273619	0.01325392	0.16907547
CD5	8.26073233	1.09998643	0.01325415	0.16907547
MALAT1	517211.071	-0.5053933	0.01324893	0.16907547
TBC1D8B	187.339731	0.58665457	0.01327207	0.16918001
ENOX2	146.092369	0.43444876	0.01329706	0.16937436
PFKM	2603.579	-0.5030221	0.01331003	0.16941545
CCDC117	289.691987	0.47175704	0.01332376	0.1694662
SCRT1	1.50918684	-1.0721162	0.01335311	0.16960053
TAS2R20	90.4845316	0.41263919	0.01335383	0.16960053
LOC613037	27.773497	0.82547046	0.01342169	0.17033785
GPA33	2.25003689	1.09086631	0.01347449	0.17088328
ACO2	1307.32586	-0.6046198	0.01358058	0.17122957
ASAP3	999.610095	0.72034743	0.01353677	0.17122957
CTBS	342.146294	0.53814741	0.01355321	0.17122957
DUT	588.856905	0.33203134	0.01355511	0.17122957
FEN1	221.938506	-0.5247579	0.0135445	0.17122957
HPR	31.0786999	1.02442641	0.01357201	0.17122957
SKA3	32.5153125	-0.9458187	0.01357537	0.17122957
TBX21	4.46263379	1.12332689	0.01354409	0.17122957
CRHR1	2.72163918	1.17025111	0.01363111	0.17165528
SYT10	1.6567405	-1.1412244	0.01363409	0.17165528
ASS1	3387.66637	-0.7985742	0.01364749	0.17169963
RGS9BP	3.42207909	-1.1318092	0.01367602	0.17193412
CAPN6	186.362762	-1.1635225	0.01371472	0.1722598
SRP68	974.617948	-0.5330505	0.01372174	0.1722598
NAA35	360.248431	-0.2891684	0.01373995	0.17236403
PRR12	903.593117	-0.4544463	0.01377523	0.1725839
UPK3BL	47.3857284	-0.7278565	0.01377733	0.1725839
GPR98	21.2176588	-1.1563273	0.01379351	0.17266218
MFSD6L	1.71545815	1.17073481	0.01380755	0.17271356
AUNIP	4.28476668	-1.077138	0.01384666	0.17295384
LRIT3	9.7193576	0.95670218	0.01384875	0.17295384
MRPS2	264.739895	-0.4184522	0.0138566	0.17295384
CHRNB2	13.4658606	-1.0715257	0.0138974	0.17303404
LRRC8A	915.693983	-0.4613285	0.01388042	0.17303404
MYBPC2	12.7973607	-1.1235327	0.01388654	0.17303404

ST8SIA4	349.045669	0.63597826	0.01390283	0.17303404
B9D2	29.4041227	0.75227922	0.01397449	0.1735467
DPM3	116.665494	-0.5296622	0.01398314	0.1735467
HMGA2	40.956061	-1.1692166	0.01398395	0.1735467
LINC00667	475.666239	0.51126568	0.0139641	0.1735467
ADORA1	8.52211296	-0.9560396	0.01399454	0.17355421
RPS14	5711.49364	0.45457329	0.0140236	0.17379064
KIAA0226L	95.3057597	0.90843464	0.0140678	0.17380721
NOXRED1	7.44322383	0.89341831	0.0140845	0.17380721
TBC1D2B	1709.25511	0.42837484	0.01405037	0.17380721
TGM4	5.92681025	-1.164403	0.01408081	0.17380721
ZNF559	453.204436	0.47286801	0.01408491	0.17380721
ZNRF2P1	37.9996369	-0.7149621	0.01406134	0.17380721
PTCH1	405.595585	-0.8477653	0.014104	0.1739193
C10orf35	20.3861996	-0.9802382	0.01420703	0.17441642
CDCA2	70.5595493	-1.0786497	0.01423072	0.17441642
CENPN	122.251268	-0.8414737	0.01420597	0.17441642
FTCD	4.67842612	-1.1629763	0.01422146	0.17441642
MAP3K8	314.891156	0.7269678	0.01418468	0.17441642
NABP1	295.171308	0.88623189	0.01422404	0.17441642
NRIP3	22.3134265	1.13808655	0.01423459	0.17441642
PJA1	469.152276	0.43418358	0.01421682	0.17441642
RNF13	1338.1564	0.27432799	0.01416857	0.17441642
UBALD1	156.210386	-0.5898085	0.01426407	0.17465448
SMC4	983.112079	-0.6959869	0.01430055	0.17494947
XCL1	2.33644077	1.155053	0.01430828	0.17494947
MNDA	203.889638	0.87225944	0.01433437	0.1751453
COPS6	724.536523	-0.4328385	0.01439502	0.17556241
HSD17B13	3.33237242	1.06519678	0.01438471	0.17556241
PRSS16	11.2831081	-1.0959091	0.0143988	0.17556241
SLC25A23	1525.64488	-0.5927992	0.01444911	0.17605239
SAFB2	837.652444	-0.4298334	0.01449138	0.17625227
TMEM219	677.802363	0.2977458	0.01449593	0.17625227
ZBTB45	130.124654	-0.451981	0.01447832	0.17625227
POLR2D	387.657036	-0.2811384	0.01454765	0.17675755
TAF15	779.280803	-0.486454	0.0145967	0.17722965
SLC22A20	9.20505104	-1.0474038	0.01461642	0.17734517
RPAP2	271.778985	0.43673027	0.01464604	0.1775807
FAHD2A	130.301695	0.51822359	0.01479805	0.17860773
GRIN2C	9.06999783	-0.9387617	0.01479438	0.17860773
LOC283683	32.5897722	1.11181688	0.01478237	0.17860773
MFSD3	116.259356	-0.5944656	0.01481293	0.17860773
NAT10	917.560944	-0.4476666	0.01480754	0.17860773
OMA1	236.501145	0.47918563	0.01476361	0.17860773
SNORA23	227.184007	-0.5204034	0.01479511	0.17860773
SQLE	374.188337	-0.7941936	0.01475605	0.17860773
PTPRZ1	12.7713193	-1.1473378	0.01482408	0.17861834
ABCF3	637.530067	-0.258424	0.01484187	0.17870882
ABCC6	16.4896135	1.05451538	0.01492162	0.17892562
DDX39A	347.405299	-0.4302479	0.01490045	0.17892562

IGF2BP2	416.80574	-1.1510982	0.01491235	0.17892562
KCNIP1	29.9068144	-1.1529329	0.0148739	0.17892562
METTL10	276.94677	0.50214526	0.01491966	0.17892562
TAF6	477.408677	-0.3761622	0.01490342	0.17892562
CS	2063.50982	-0.4097896	0.01496326	0.17930133
DAO	2.70350094	1.02702124	0.01500773	0.17959447
EPN3	22.3068063	-1.1533094	0.01502904	0.17959447
LOC1001336	3.67237557	1.11897096	0.0150152	0.17959447
SDHC	785.760084	-0.4706286	0.01501917	0.17959447
EI24	959.016557	-0.295224	0.0150807	0.18008803
COMTD1	31.6493117	-0.7250692	0.01510134	0.18021074
NEB	704.185113	0.95883238	0.01513872	0.18053283
TNK1	83.5893021	-0.7633907	0.01515122	0.18055811
DOCK10	685.79726	0.81011484	0.01523434	0.18142433
FLJ31104	8.34937624	0.88364977	0.01524722	0.18145337
LOC1009965	7.41638425	-1.0895712	0.01527475	0.18165665
ZNF25	419.466547	0.58230736	0.01529585	0.1817833
SLC6A3	154.287986	-1.1496523	0.01533059	0.18207175
MYL12A	2126.1642	0.48449133	0.01534828	0.18215737
PI4KB	1417.54572	-0.2401294	0.01538344	0.18245012
BTNL9	982.117681	-0.9410668	0.01539917	0.18251215
ROBO3	179.496297	1.04147704	0.01541846	0.18261633
C2orf15	15.9208483	-0.8671911	0.01543209	0.18265331
SMPD4	685.919526	-0.2753414	0.01545541	0.18280491
BLNK	205.852261	0.80467795	0.01549999	0.18283465
C5orf49	3.65264342	1.13069192	0.0154999	0.18283465
KCNH1	11.5915859	-1.0807278	0.01549854	0.18283465
SLC37A4	225.217709	-0.4050251	0.01547849	0.18283465
KCTD2	821.354827	-0.4099064	0.01553825	0.18303758
LEAP2	3.49837654	1.03275487	0.01553732	0.18303758
DNMT3A	701.854445	-0.4725667	0.01556513	0.18310614
LOC1019282	3.44305196	-1.135925	0.01555469	0.18310614
C19orf48	294.411674	-0.4243307	0.01559075	0.18328361
PRRT3	91.6150895	-0.7452494	0.01562261	0.18353404
TSTD3	63.2364623	0.73284722	0.01563822	0.18359335
CES3	22.1203481	1.02427351	0.01566588	0.18379401
COX6B2	4.89335354	-1.1468595	0.0157073	0.1841556
CAP1	2371.90794	0.42432981	0.01573693	0.18425453
THAP4	435.310782	-0.2919158	0.0157315	0.18425453
MSS51	98.1073984	0.67483052	0.01580466	0.18492303
RNF182	6.85710365	-1.1469258	0.01588382	0.18572427
CHAF1A	169.218385	-0.666102	0.01592351	0.1860632
GRM3	11.4956089	-1.1374406	0.01596554	0.18642908
LACC1	228.301674	0.54055691	0.01599089	0.18659977
RBM4	728.764555	-0.3701869	0.01603722	0.18701481
SSTR5-AS1	2.14356812	-1.1166227	0.01605984	0.18715306
FITM2	109.434761	-0.5307921	0.01607424	0.18719541
ITGAX	455.045449	0.92606638	0.01609079	0.1872628
ACSL1	1056.03906	0.72512212	0.01623819	0.1873922
CCR5	65.0582348	0.99330811	0.01612224	0.1873922

COMMD2	641.403255	0.4450997	0.01627436	0.1873922
FAM86C1	51.3468536	-0.5634412	0.01617634	0.1873922
GLTSCR1	210.451893	-0.7377625	0.01624873	0.1873922
HPS5	881.543494	0.58881881	0.01625165	0.1873922
KLK5	7.32857935	-1.0499592	0.01623049	0.1873922
NCF4	123.715603	0.77018404	0.01614381	0.1873922
NRCAM	40.3345165	-1.1018349	0.01626107	0.1873922
PCDHGB7	892.987703	-0.8027306	0.01612816	0.1873922
PRKAG2-AS1	12.2358975	1.00299524	0.01626655	0.1873922
RASSF2	6024.07386	0.91075672	0.0161871	0.1873922
RLF	589.143563	0.5387284	0.01618821	0.1873922
SENP7	894.668701	0.35986602	0.01625784	0.1873922
TLCD1	13.2609508	-1.0041464	0.016217	0.1873922
WDR11	1458.77934	0.48475314	0.01615811	0.1873922
CABIN1	1078.29729	0.33600993	0.01628886	0.18743504
MINOS1P1	196.476316	0.47437658	0.01632873	0.18764549
SYMPK	1140.62695	-0.3045909	0.01632427	0.18764549
ABCB6	437.081678	-0.4326825	0.01641295	0.18790826
AKR1B10	24.9980425	1.14062085	0.01641592	0.18790826
ATP5D	485.750065	-0.5848239	0.01643806	0.18790826
CLIC3	195.948641	-1.0202733	0.01638594	0.18790826
DFNB31	192.773908	-0.747445	0.01638825	0.18790826
LOC1005073	2.50191083	-1.132414	0.01642745	0.18790826
NT5C1A	15.6317329	1.1384546	0.01641328	0.18790826
RPGRIP1	9.92410529	1.04235924	0.0164359	0.18790826
DNM1	64.5215266	-0.9113088	0.01645401	0.18796704
CALCR	8.57697787	1.07072298	0.01651972	0.18809984
EPPK1	1089.65394	-1.1078982	0.01649693	0.18809984
LOC399715	60.3839193	0.88655092	0.01651797	0.18809984
LPPR2	1176.77495	-0.4982927	0.01650818	0.18809984
PDIA5	163.556368	0.50264791	0.01649132	0.18809984
PIK3R6	27.2678802	0.72385608	0.01653801	0.18818483
ATP8A1	992.608097	-0.8345749	0.01657716	0.18826064
RHPN1-AS1	2.42737368	-1.0516708	0.01657102	0.18826064
TRAT1	3.94143439	1.12162441	0.01657221	0.18826064
ISY1	130.741211	0.46304547	0.01659599	0.18832428
LOC374443	314.827597	0.58641042	0.01660442	0.18832428
DNAJB1	1920.9973	0.63929147	0.01662268	0.1884084
FBXO8	290.793072	0.29716836	0.01663743	0.18845281
SNX22	26.3391682	0.94099405	0.01669728	0.1890076
NEK10	9.42755327	0.95467198	0.01674512	0.18917955
PIH1D2	21.6655313	-0.685592	0.01672817	0.18917955
RAMP1	27.6680943	-1.1105047	0.01673842	0.18917955
SRRD	116.69331	0.45368991	0.01677789	0.18942678
C6orf226	31.4031886	-0.6247535	0.0168422	0.19002942
HID1	72.8834107	-0.9561944	0.01689458	0.19049682
ATIC	675.562003	-0.7285448	0.01691187	0.19056817
HERC2	3890.14121	-0.279105	0.0169236	0.1905768
CACFD1	238.992506	-0.4640635	0.0169464	0.19071002
E2F1	183.77865	-0.8395974	0.01696551	0.19080159

ARHGAP20	3220.22704	-0.8564474	0.01706208	0.1911038
ATP5B	5600.99872	-0.5481065	0.01702757	0.1911038
CDKAL1	380.907436	0.35826593	0.01704586	0.1911038
FAM102B	438.959412	0.60964523	0.0170865	0.1911038
OIP5	8.72995543	-0.9433605	0.0170184	0.1911038
PIP4K2A	486.775243	0.37634484	0.01704271	0.1911038
PROX1	27.2684032	0.90818651	0.0170952	0.1911038
SCARA3	4456.38511	-0.8038482	0.01710229	0.1911038
TFEC	268.844865	0.86526918	0.01706702	0.1911038
TPCN2	317.356818	-0.3373143	0.01707049	0.1911038
CTGF	13665.3728	1.03075049	0.0171511	0.19133894
LRRC42	311.022336	0.61775557	0.01716735	0.19133894
MAPK1	1988.85738	0.30028091	0.01714064	0.19133894
MPHOSPH6	114.52078	0.54305119	0.01715703	0.19133894
ACKR2	30.1111343	1.0025344	0.01732517	0.19145563
ACLY	2181.29011	-0.3040585	0.01730602	0.19145563
ACP2	584.593295	-0.3901762	0.01733198	0.19145563
ATG13	1348.80266	-0.2963339	0.01727515	0.19145563
CCND1	19967.1935	-0.7993785	0.01723183	0.19145563
GTPBP10	436.713416	0.27787701	0.01721605	0.19145563
INTS10	495.307468	0.44230977	0.01732125	0.19145563
MT3	44.8849859	-1.0798902	0.01729119	0.19145563
OVGP1	45.0434046	0.74480726	0.01723431	0.19145563
RAI1	868.637253	-0.5200545	0.01725364	0.19145563
SCAMP5	285.292929	-0.8820961	0.01728973	0.19145563
SEC22A	261.621064	0.27671166	0.01730831	0.19145563
SF3B5	409.133445	0.39578967	0.01721411	0.19145563
TSEN54	119.368107	-0.4967998	0.01726864	0.19145563
TLK1	1121.6758	-0.3345975	0.01737764	0.19183817
BLOC1S3	157.194829	-0.4940881	0.0173945	0.1919024
ZNF598	388.837672	-0.4643994	0.01740775	0.19192681
TRIM23	506.352714	0.33762106	0.01742658	0.1919626
ZNF711	232.514651	-0.8552239	0.01743308	0.1919626
FGFR1OP2	577.526444	0.29571449	0.0174699	0.19223439
GORAB	177.209262	0.45684595	0.01747987	0.19223439
PIGO	588.022591	-0.3664763	0.0174925	0.1922516
SLA2	10.3487425	0.87600969	0.01755268	0.19279116
POLK	953.118054	0.30203528	0.01762566	0.19347051
CSTB	898.35981	-0.7513695	0.01763943	0.19349949
CASP2	550.064222	-0.3915954	0.01772541	0.19424918
ILDR2	236.496739	1.12592011	0.01774128	0.19424918
SIRT1	503.372634	0.44055795	0.01773293	0.19424918
BZW2	195.374091	-0.6059189	0.01781281	0.19458405
CDKN3	19.7869369	-0.905422	0.01783719	0.19458405
GBA2	1186.03805	-0.3780648	0.01785402	0.19458405
PATL2	9.73952006	0.96329975	0.0178405	0.19458405
SDHAP1	518.487196	-0.5507409	0.01784142	0.19458405
SLC26A6	736.579593	-0.8780507	0.0178614	0.19458405
SRPX2	15.7824909	1.11794584	0.01783797	0.19458405
TRIM7	26.7614931	0.98158775	0.01782526	0.19458405

INTS6-AS1	30.2287167	0.49178985	0.01788161	0.19468228
C1orf61	5.27018462	-1.0440995	0.01789715	0.19472955
ABCA1	2710.2128	0.68773355	0.01793348	0.19498074
DOCK2	756.903144	0.75595777	0.01795994	0.19498074
KIF5A	226.378624	-1.1259126	0.01796335	0.19498074
SKAP2	488.297166	0.71707496	0.0179651	0.19498074
MDH2	1288.62729	-0.4885631	0.01797771	0.19499592
ACTR6	190.377064	0.40522564	0.0180569	0.19514269
CLEC12A	16.7587941	1.11433031	0.01801492	0.19514269
EPG5	1827.44992	0.49014433	0.01804255	0.19514269
HIST1H4B	250.373391	0.60581777	0.01807306	0.19514269
KIF21B	187.803962	0.75105179	0.01802902	0.19514269
MRPL24	382.777471	-0.4520213	0.01803205	0.19514269
NPRL3	430.839468	-0.4471044	0.01808103	0.19514269
TUBG1	383.461482	-0.5488228	0.01806437	0.19514269
BMP2	47.4909301	0.98039928	0.01812239	0.19546769
LAMA5	5585.01242	-0.8831955	0.01819024	0.19584568
MRPL54	169.432363	0.40706951	0.0181879	0.19584568
SNORA38B	12.4094927	0.98184428	0.01819122	0.19584568
ANAPC16	853.97322	0.4979903	0.0182297	0.19613848
FES	319.943194	0.42481216	0.0182567	0.19630754
GPC3	110.988335	-1.1218105	0.01827008	0.19632993
HSD17B11	408.435192	0.51812922	0.01828443	0.1963628
GLMN	83.116912	0.68582362	0.01832031	0.19662671
UBAP2	797.449702	-0.4950695	0.01835232	0.19684869
AEBP1	4884.14427	1.04992806	0.01839345	0.19716823
SCARNA10	4433.99956	0.39589014	0.01841787	0.19729815
SPOPL	912.912664	0.60242475	0.01842826	0.19729815
CLDN23	4.68164198	0.96297832	0.01851118	0.19761212
RGS18	86.3581588	0.95492831	0.01848401	0.19761212
SEC31B	444.70062	0.48402269	0.01848723	0.19761212
TAS2R10	24.4450799	0.72881669	0.018501	0.19761212
TMEM11	148.371573	-0.3892681	0.01851442	0.19761212
NDUFB2-AS1	6.13048241	-0.9207575	0.01857261	0.19762662
PCDHGB5	579.909784	-0.9098306	0.01854479	0.19762662
TNFRSF10A	22.0537324	0.89392939	0.01856724	0.19762662
ZFAND5	3289.28953	0.5996366	0.01856215	0.19762662
ZFP41	383.341293	-0.4980865	0.01855514	0.19762662
DSEL	611.097607	0.70461021	0.01858405	0.19762749
SHPK	390.217852	-0.3649571	0.01865158	0.19822431
FBXL18	410.955757	-0.3512331	0.01868855	0.19849588
INPP5J	188.971831	-0.8246993	0.0187151	0.19865654
STK16	224.077854	-0.4140773	0.0187615	0.19902754
IFI27L1	48.0921958	0.70406199	0.01880199	0.19921402
TMEM175	387.283106	-0.4384063	0.01880165	0.19921402
SLC12A8	16.6637185	-0.9705674	0.0188782	0.1998997
CLN6	342.624497	-0.3616015	0.01890946	0.19998713
DNAL4	103.025928	0.56034046	0.01889931	0.19998713
CDK18	213.429492	-0.7744051	0.01893745	0.20016135
LMNB2	678.346203	-0.5228833	0.01897287	0.20029227

UBE2Q1	836.140839	-0.2347195	0.01896869	0.20029227
PQLC3	356.8924	0.55022388	0.019024	0.20071017
ATP8B3	92.8611495	-0.8697718	0.01910489	0.2007449
FBXO31	713.013858	-0.364787	0.01908701	0.2007449
HTR7	20.5064833	1.02563025	0.01911652	0.2007449
MCOLN3	14.6077605	1.02163996	0.01909479	0.2007449
NFKBIA	2585.72042	0.54790364	0.01908437	0.2007449
OSBPL8	1946.56494	0.38894605	0.01905298	0.2007449
TNNI3	4.7184326	-1.115221	0.01903985	0.2007449
VSIG10	272.259493	-0.5190829	0.01911966	0.2007449
DHX33	432.06263	-0.4497081	0.01916575	0.20110735
CECR1	447.045644	0.82263168	0.01917751	0.20110934
FBXO46	207.039032	-0.4658394	0.01923608	0.20111713
FMOD	4418.7983	0.54991032	0.01922066	0.20111713
NFAM1	144.207476	0.86565821	0.01919451	0.20111713
SPCS2	542.791516	-0.4168308	0.01923186	0.20111713
ZFAND3	1391.49006	-0.3689104	0.01922359	0.20111713
STAC2	11.7699095	-1.1093399	0.01925194	0.20116199
AOAH	256.168053	0.90072539	0.01929212	0.20121896
LOC1019276	6.29801763	1.07087018	0.01928409	0.20121896
SCAMP3	691.406861	-0.4462246	0.01927212	0.20121896
SET	3991.69927	-0.369849	0.01935741	0.2016825
SYCP2	112.655948	1.05120675	0.01935976	0.2016825
IFI6	667.401381	0.64846233	0.01938086	0.20178141
MAP3K9	183.480868	-0.7079798	0.01942373	0.20210677
LINC01140	4.55691799	0.90864555	0.01943666	0.20212032
CWF19L1	305.818563	0.41032781	0.01946097	0.20225222
CNN2	914.695212	0.82081381	0.01949787	0.20239376
RNF175	6.23939798	1.10950429	0.01949385	0.20239376
BBOX1	1.68468181	1.07306883	0.01952841	0.20246898
CAMLG	495.951153	0.43599267	0.01952707	0.20246898
SCAF1	888.378566	-0.3368728	0.01954768	0.20254795
C21orf88	4.72866787	-1.107982	0.01957095	0.2026683
UQCRC1	1286.21517	-0.6211502	0.01959065	0.2027516
ROR1	384.425255	1.01696193	0.01964346	0.20305641
SCUBE3	728.573639	-0.8218843	0.01963711	0.20305641
FAF1	562.543406	0.29782297	0.0196801	0.20331423
GLYR1	1419.53814	-0.2955588	0.01970676	0.203348
SIGLEC11	60.4539464	1.02248131	0.0196975	0.203348
ARPP21	2.22810699	-1.0908135	0.01983157	0.20336723
ATP13A1	885.805704	-0.3465892	0.0198353	0.20336723
EMC6	123.248514	-0.5765723	0.01984863	0.20336723
FIGNL2	9.34717859	1.06954148	0.01977984	0.20336723
GAPT	79.9413979	0.98527206	0.01972199	0.20336723
HIST1H3G	73.0565818	-0.9043276	0.01986067	0.20336723
HUNK	35.8323698	-0.8701762	0.01984215	0.20336723
LMCD1-AS1	13.948951	1.05220182	0.01978264	0.20336723
LRRC4B	130.244496	-1.0885588	0.01985729	0.20336723
PLEK	244.813059	0.78794883	0.01980845	0.20336723
RSF1	1486.43638	-0.3299063	0.01982598	0.20336723

TLX1	12.689766	0.99405157	0.01984443	0.20336723
TMEM65	372.74902	0.62119155	0.01973322	0.20336723
NCKIPSD	599.9651	-0.5109912	0.01988872	0.2035345
BID	120.978359	0.41620386	0.01990463	0.20357755
DLGAP5	24.0083964	-0.9530211	0.01991693	0.20358357
SLC24A4	24.7506912	0.95831546	0.02003246	0.2046442
CCL2	61.4177212	0.86899819	0.02009888	0.20520211
CKS1B	81.9887304	-0.5498094	0.02019444	0.20605677
C7orf55	47.9986345	0.56512067	0.02028478	0.20685713
CBR3-AS1	13.50144	0.7090397	0.02029762	0.20686681
ATAD2	505.639468	-0.6312285	0.02032167	0.20699054
ASPG	7.99686321	1.01819742	0.02035402	0.20707745
MCM6	382.547095	-0.4646323	0.02034815	0.20707745
GPR126	308.139693	0.90038941	0.02038433	0.20726451
AGPAT9	67.5119367	0.87640276	0.02040135	0.20731636
PTPMT1	357.471986	-0.2819249	0.02042769	0.2074628
ERMARD	251.673881	0.44907719	0.02045544	0.20746534
ITGB3	133.230491	0.85949379	0.02045048	0.20746534
PCDHGA8	453.042476	-0.7087617	0.02046648	0.20746534
SNTG1	11.1011777	-1.0247754	0.02047567	0.20746534
DDX50	531.331121	0.44471711	0.02053826	0.20768997
FAM168B	2427.861	-0.2742553	0.02054562	0.20768997
MTR	2202.69586	-0.3247952	0.02052724	0.20768997
PIK3CD	231.695171	0.79294009	0.02051486	0.20768997
RHOT2	731.295603	-0.3839604	0.02057668	0.20776238
SCAND1	260.729242	-0.4929399	0.02057503	0.20776238
BAZ2A	2621.33734	-0.4376202	0.02067766	0.20795268
CLCN4	348.277016	-0.6677132	0.02061223	0.20795268
COL5A3	360.6863	0.84947071	0.02064515	0.20795268
DPYD	729.645364	0.72850242	0.02067925	0.20795268
OMG	3.30517885	-1.0887	0.02063774	0.20795268
SHMT2	971.350834	-0.5594682	0.02063303	0.20795268
SYF2	387.446518	0.5030314	0.0206562	0.20795268
FGD3	122.305496	0.84202053	0.02076778	0.20872218
DPY19L2P1	3.07955221	-1.0919747	0.02081742	0.20910026
PELI2	142.112994	0.74127422	0.02083484	0.20915438
NDUFS8	734.348193	-0.5035675	0.02085035	0.20918933
C19orf68	48.6472269	-0.5621976	0.02095091	0.20999151
CAPN1	1566.37691	-0.4027477	0.02096654	0.20999151
LINC00260	67.0448266	-0.5808949	0.02096484	0.20999151
LOC283335	125.136252	-0.5119278	0.0210373	0.21055132
THAP8	38.6487974	-0.6020883	0.02104666	0.21055132
SLC25A25	483.81566	0.75424476	0.02107039	0.21066754
SCAP	1467.98571	-0.3708484	0.02109398	0.21078217
SAP130	469.096323	-0.3758627	0.02111395	0.21086052
SAMD9L	1618.07619	0.40169307	0.02112641	0.21086387
LOC1019272	40.3230912	0.58866208	0.02114418	0.21092018
ZFPL1	261.263955	-0.3345085	0.02116482	0.21100504
CYB5RL	80.1570353	0.48662857	0.02120471	0.21102958
HCG11	396.977876	0.85444441	0.02121583	0.21102958

MRPS5	441.433288	-0.3650312	0.02119338	0.21102958
SKINTL	9.63701817	0.96943952	0.02119264	0.21102958
C12orf61	10.607419	-0.7686292	0.02131109	0.21185587
ASPHD1	2.79159796	-1.0921215	0.02136907	0.21231083
GNB4	1378.75206	0.74032026	0.02139059	0.21240335
SCP2	1172.02763	0.43404301	0.02141268	0.21250129
ABCC4	969.669624	-1.0051327	0.02142658	0.21251798
PRLR	46.0667755	0.95407071	0.02144086	0.21253831
NAA60	679.787761	-0.4079099	0.02146001	0.21260699
KLHL8	341.765666	0.34943375	0.02150445	0.21292586
DCT	9.20397316	0.88646962	0.0215407	0.21292612
NSUN5P1	329.433093	0.66335717	0.02152891	0.21292612
TMCC3	614.216183	0.64258343	0.02154121	0.21292612
LACTB	239.709333	0.57604961	0.021613	0.21351436
CXCR2	17.8931913	0.81784564	0.02162606	0.21352201
ARL16	247.621612	-0.3969941	0.02166547	0.21378977
S1PR4	5.21458868	1.03209087	0.02171602	0.21416698
NCAPD2	655.945655	-0.2937795	0.02173395	0.21422229
RAB6C	8.29464806	-0.9161483	0.02176147	0.21437203
ELF4	571.687794	-0.4650494	0.02177497	0.2143836
C12orf29	148.240804	0.35647449	0.02186062	0.21452397
GM2A	1117.26506	0.35651929	0.02182236	0.21452397
HMGCS1	911.652454	-0.7171924	0.02186289	0.21452397
IDH3A	801.884116	-0.6547476	0.02189548	0.21452397
MYO3A	106.623365	-1.077086	0.02180205	0.21452397
PSMD10	365.616454	0.30677418	0.02186603	0.21452397
SECTM1	49.067733	0.85253711	0.02189186	0.21452397
STMN2	2.39704459	-1.0742643	0.02188136	0.21452397
ZNF22	249.891944	0.56915901	0.02190027	0.21452397
MB21D2	73.0048467	0.80856601	0.02193474	0.2147406
ABCB4	37.1981269	0.79368286	0.02196255	0.21489184
ACAP1	28.5664862	0.86034876	0.02199375	0.2149552
FOXD2	942.465971	-0.6287259	0.02199211	0.2149552
AKAP3	11.2480988	0.91690054	0.02203102	0.21496607
CAPN15	493.434178	-0.5240137	0.02203372	0.21496607
CNIH3	23.5528585	0.86501915	0.02205138	0.21496607
PLEKHF2	193.468751	0.35078398	0.02205316	0.21496607
SEL1L3	485.227304	-0.7609562	0.02205668	0.21496607
FAM127C	206.909285	-0.5002763	0.02211144	0.2150177
IPO7	3149.28198	-0.3251077	0.02209604	0.2150177
8-Mar	681.023024	0.39575728	0.02210053	0.2150177
SNORA52	23.0559988	0.80566754	0.02208905	0.2150177
ANKRD13C	565.789963	0.44004773	0.02219015	0.21557485
ERBB2IP	3374.26577	0.27756064	0.02219353	0.21557485
TNNT2	1475.20501	-0.9325079	0.02221806	0.2156026
ZNHIT6	311.704594	0.37093872	0.02222119	0.2156026
NSUN7	76.2870332	-0.7089598	0.0222589	0.21575755
TCTN3	829.238342	0.37830277	0.02226198	0.21575755
CD52	13.6399702	0.87895603	0.02234388	0.21627593
HERC4	963.61392	0.29143824	0.02238071	0.21627593

HHIPL1	88.0496148	0.7851386	0.0223901	0.21627593
JUP	2473.55204	-0.5995679	0.02235301	0.21627593
SLC30A3	22.6380149	1.082923	0.02234443	0.21627593
TRABD2A	14.7184264	-0.9514251	0.02238316	0.21627593
CEP41	178.313391	0.46650335	0.02242751	0.21639681
TANGO6	355.619864	0.45995293	0.02241776	0.21639681
C16orf93	27.1076684	-0.5959893	0.02248243	0.2164463
KIAA0895L	428.970034	-0.5863698	0.02247273	0.2164463
POTEJ	28.3274686	-0.7833509	0.02246992	0.2164463
ULK1	895.465734	-0.5465802	0.02247607	0.2164463
FGD1	315.451466	-0.3506129	0.02250534	0.21654697
LOC220729	420.605955	-0.4642984	0.02253884	0.21662953
POLDIP2	1095.68949	-0.3822202	0.02253702	0.21662953
HSBP1L1	91.9221083	-0.5251779	0.02259062	0.21688741
SEMA3E	4.38552869	0.99599892	0.02258093	0.21688741
PDF	16.460665	-0.7497043	0.02266379	0.2174698
LRRC40	199.181019	0.42229174	0.02268386	0.21754237
BTN3A1	391.206293	0.56953387	0.02277199	0.21778688
CPO	10.4482068	0.84403212	0.02274252	0.21778688
HTRA1	1536.66315	0.74335913	0.02275144	0.21778688
1-Mar	379.859474	0.79825416	0.02276107	0.21778688
RPS23	6613.63454	0.45185596	0.02272692	0.21778688
RSRP1	765.066818	0.50532918	0.02280135	0.21782805
WHAMMP3	138.607852	0.52783199	0.02278934	0.21782805
CEP135	226.552622	0.45224283	0.0229153	0.21859047
KMT2D	4100.80275	-0.4158093	0.02291887	0.21859047
SLC25A13	347.854663	0.40886223	0.02291568	0.21859047
MX1	1088.20851	0.51438313	0.02294095	0.21867294
TOB1	2268.61505	-0.6877087	0.02295267	0.21867294
LOC1019272	2.87444917	1.06242824	0.02297759	0.21879044
OGDHL	549.683424	-1.0153491	0.02300299	0.2189124
SNX9	1092.41067	0.40987145	0.02304448	0.2191873
TSTA3	307.565367	-0.6122919	0.02307823	0.21938831
ITGAM	683.923182	0.86078714	0.02311663	0.21963325
ANKRD39	70.7748302	-0.4513276	0.02316986	0.21978369
FAAH	146.221423	-0.8561108	0.02318073	0.21978369
NRG4	38.7973849	-1.0654855	0.02319255	0.21978369
RPUSD3	137.418033	-0.365119	0.02317888	0.21978369
SNHG10	69.0186401	0.54481739	0.02319567	0.21978369
ACTN4	4774.61394	-0.3803664	0.02322562	0.21980358
CHST11	663.672446	0.67747885	0.02322473	0.21980358
HNRNPLL	576.215159	0.38243633	0.02329873	0.21980358
NME4	800.256034	-0.5490517	0.0232989	0.21980358
RPS19	3971.51888	0.36284608	0.02323645	0.21980358
SNX29	1299.29766	0.5117252	0.02329825	0.21980358
TMEM260	430.044493	0.48357613	0.02328556	0.21980358
TTYH1	7.1437205	-1.0795841	0.02327117	0.21980358
ITGA3	768.657328	-0.7316395	0.02332039	0.21988702
GFI1	7.84912419	0.90554414	0.02335019	0.22004862
CLK2	655.148327	-0.3422149	0.0234194	0.2205813

LOC728819	4.00477243	-1.0729496	0.02346078	0.22085138
C7orf76	3.06143702	1.04768068	0.02350998	0.22119481
ZFHX4	1144.16192	-0.7384786	0.02352682	0.22123352
LINC00632	2.24320119	-1.0421596	0.02354306	0.22126656
KLC2	379.75976	-0.4972352	0.02360938	0.22145809
LOC1005070	19.7006025	0.79203955	0.02358671	0.22145809
MAP4K1	46.6511979	0.71486945	0.02362091	0.22145809
TPX2	243.274106	-0.8622154	0.02359646	0.22145809
ZNF699	99.6650107	0.52957391	0.02362712	0.22145809
GUSBP3	31.1473823	0.6918888	0.02365509	0.22160081
ADAMTS4	121.0676	1.00606538	0.02366811	0.22160331
NCAPH2	281.76428	0.33632678	0.02368737	0.22166435
CUX1	3187.06854	-0.4641897	0.0237041	0.22170154
MIR155HG	24.0683038	0.96302953	0.02375797	0.22184742
NBAS	1832.27167	0.27532798	0.02375185	0.22184742
SPRY1	725.704707	0.81108963	0.02373873	0.22184742
FGD2	347.152224	0.81760202	0.02380393	0.22211994
MICU3	166.352668	0.50395795	0.02381271	0.22211994
GORASP2	1000.53062	-0.3448157	0.02396279	0.22340002
NHLRC4	22.9725902	0.8507309	0.0239999	0.22362605
CCL19	2.73843641	1.07384808	0.02401449	0.22364215
CCDC178	6.10404208	-1.0734467	0.02406278	0.22376212
GOT2	958.047822	-0.4282375	0.02406067	0.22376212
TMEM151B	10.8733918	-1.0411875	0.02406598	0.22376212
CHCHD6	145.135902	0.8418858	0.02412437	0.22418514
SLC47A1	18087.3545	-0.765393	0.02414973	0.22430094
EAF2	52.3195924	0.61812557	0.02421297	0.22476828
CXCL16	618.1594	0.59633366	0.02426519	0.22479214
FZD2	922.284736	-0.5313459	0.02426961	0.22479214
ICK	698.567284	0.54131913	0.02428019	0.22479214
TNFSF13B	90.3036759	0.67587961	0.02423749	0.22479214
ZFP36L2	3711.77489	-0.5278764	0.02424291	0.22479214
GPR133	727.241265	1.00178976	0.0242962	0.22482069
CEP120	1316.42276	0.43608802	0.02438171	0.22525231
GAS2L2	3.97672747	1.04930584	0.02437693	0.22525231
TSPAN7	1093.2896	1.00298209	0.02436466	0.22525231
COMMD3	144.607933	0.47082503	0.02440063	0.22530737
C15orf56	5.02940631	-0.8947364	0.02441758	0.22534419
CLEC17A	13.3043742	1.04030977	0.0245615	0.22602899
ECT2L	5.15152394	0.91239442	0.02456782	0.22602899
KLK14	1.48870345	-1.054773	0.02456978	0.22602899
LRRC8C	1350.67903	0.56654901	0.02453134	0.22602899
5-Sep	7.00289732	-0.8140521	0.02455168	0.22602899
SH2B2	12.0060691	-0.8624739	0.02456924	0.22602899
CHAMP1	302.351162	-0.501813	0.02458733	0.22607079
HNRNPD	1198.63101	-0.5032638	0.02460929	0.22615317
C21orf128	2.04934458	-0.987804	0.02467428	0.22651097
ZNF683	2.64533359	1.05764801	0.02466782	0.22651097
TM6SF2	27.8290442	-0.8036723	0.02472036	0.22681417
AOAH-IT1	3.97782033	1.06798343	0.02478354	0.22708669

ARPC5L	169.938089	-0.3817064	0.02480281	0.22708669
FAM209B	1.83019968	1.02331912	0.0247986	0.22708669
INHBA	35.4329491	0.95308813	0.02477483	0.22708669
LOC1005060	13.2443838	0.92419905	0.02481536	0.22708669
AMH	71.3763542	-1.0425391	0.02489788	0.227722
FZD8	192.692848	0.72706782	0.02492896	0.22788633
MTMR4	1504.43764	-0.3746614	0.02505049	0.22839687
SLC25A44	488.020317	-0.4017687	0.02502591	0.22839687
STAT5A	513.060626	0.42143253	0.02502689	0.22839687
TTLL13	2.77602982	0.95485214	0.02499833	0.22839687
ZNF574	267.234425	-0.3298897	0.02504835	0.22839687
CMAHP	245.634786	0.8110777	0.02513503	0.22880776
HEXB	1339.26651	0.45052303	0.02512886	0.22880776
ZNF587	1604.24219	-0.3908286	0.02512937	0.22880776
MAPK7	340.761987	-0.396828	0.02514971	0.22882158
SNORA47	139.93796	-0.7345851	0.02519316	0.229097
GALNT6	33.8115769	0.72726315	0.02523491	0.22923689
STS	544.439592	0.86039644	0.02522246	0.22923689
FRA10AC1	203.864359	0.54622705	0.02525419	0.22929063
SLC6A17	35.9318781	1.05185537	0.0252672	0.22929063
GFAP	463.348055	-0.9769606	0.0253298	0.22961114
IL7R	50.5738433	0.91289791	0.02532611	0.22961114
KIAA1328	314.680197	0.50019435	0.02534214	0.22961114
CDK10	330.42023	-0.3596547	0.02545491	0.23051277
SNTB2	1670.47162	0.59458364	0.02550029	0.2308035
SLC29A4	40.2400635	-1.017314	0.02553219	0.230972
ASH1L-AS1	55.4314656	-0.5060008	0.02554889	0.23100291
APOL3	185.002227	0.69841869	0.02559005	0.23125478
LOC1019271	2.4332027	1.06108091	0.0256382	0.23132922
MIA3	1947.25171	-0.2723124	0.02563525	0.23132922
PYGL	706.36427	0.5826898	0.02561255	0.23132922
FCGR2A	1159.28735	0.64252388	0.02566012	0.23140692
GZMM	2.211031	1.05998369	0.02568412	0.23150323
LOC1019297	12.4007671	1.06113374	0.02569971	0.23152377
LPCAT2	528.828608	0.83619309	0.02572553	0.23163635
GALNT4	14.4813983	0.75017334	0.02575486	0.2317804
LRRC66	15.1803837	0.88528067	0.02583438	0.23237578
CA8	110.526211	-1.0294945	0.02585702	0.23239019
NOL12	161.06156	0.43037076	0.02586271	0.23239019
INSRR	15.9576085	-0.9918663	0.02587969	0.23242258
POLR3K	81.0842043	-0.505911	0.02595817	0.23300704
PDHX	639.379876	-0.3605973	0.02600117	0.23315233
TRIM47	144.958676	-0.6898732	0.02598972	0.23315233
C5orf27	1.89735155	1.03890282	0.02603021	0.23320499
GZMK	15.5997253	0.99388617	0.02603875	0.23320499
SNAP25	17.255428	-1.0576736	0.02604728	0.23320499
TNFRSF1B	755.703004	0.70192278	0.0260612	0.23320947
GMFG	86.4196396	0.71543611	0.0260753	0.23321569
CCR1	197.787665	0.83359577	0.02610804	0.23338842
ADAT2	111.203347	0.60640682	0.02616298	0.23339956

MTRNR2L2	4241.1046	-0.7260674	0.02615022	0.23339956
PLEKHO1	363.881833	0.56444004	0.02613101	0.23339956
UBQLN1	2000.31087	-0.375093	0.02615229	0.23339956
CCR4	4.01110993	1.0524117	0.02618238	0.23345282
CELF5	3.75096798	-1.0083411	0.02623164	0.23354527
FARSB	458.348173	-0.3600004	0.02620796	0.23354527
SOX11	265.871433	-1.034849	0.02623304	0.23354527
IMMP2L	95.8151445	0.5007294	0.0262615	0.23367899
LOC1019272	12.3568726	0.98038527	0.02628788	0.23379406
C11orf21	13.7672304	1.00355534	0.02642508	0.23477404
DOCK11	482.877773	0.53957063	0.02641911	0.23477404
LOC1019271	2.93275656	1.05307382	0.02648339	0.23517197
ZIC1	3537.61055	-0.9088952	0.02656076	0.23573859
CXorf21	37.1817928	0.94684863	0.02658733	0.23579899
SMU1	1318.37444	0.16131158	0.02659469	0.23579899
CALY	7.3030528	-1.0479285	0.0267759	0.23621475
CDKL4	5.55580025	0.91374789	0.02681819	0.23621475
CRYM	12.7679414	-1.0535627	0.0267356	0.23621475
CTSS	1017.31921	0.65938736	0.02670216	0.23621475
HIF3A	1349.91939	-0.8805369	0.026766	0.23621475
NDNF	1552.03356	-1.0169922	0.026797	0.23621475
NFKB2	285.747939	0.44182201	0.02679863	0.23621475
PCDHGA1	140.860376	-0.87027	0.02681438	0.23621475
PCSK9	25.9853045	-1.0478789	0.02679507	0.23621475
PLA1A	6.88930622	0.94286523	0.026692	0.23621475
SIN3A	1649.3924	-0.3703822	0.02668016	0.23621475
SMAD5	2423.18462	0.38990122	0.02674339	0.23621475
ZNF8	357.246858	-0.3292774	0.0267363	0.23621475
TLX1NB	1.6017985	0.91137272	0.02689014	0.23672852
SLC22A2	15.4594627	1.05248051	0.02690795	0.23676548
DLGAP1-AS2	13.9576287	0.64018166	0.02692939	0.23683424
FUT7	4.42490503	0.98288143	0.0269534	0.23692561
ARMC2	120.714659	0.6113575	0.0269904	0.23696541
NUMBL	783.050564	-0.4167651	0.02699288	0.23696541
SCYL1	1084.51047	-0.2939607	0.02699882	0.23696541
HOGA1	133.47213	0.82053366	0.027054	0.23713153
LDLRAD2	12.57815	1.04200095	0.02705866	0.23713153
WEE2-AS1	15.8343188	0.73394923	0.02705513	0.23713153
MCM7	724.184681	-0.4355571	0.02707725	0.23713982
OR2D2	4.07557367	-1.0354666	0.02708689	0.23713982
ARHGEF26-A	1.58492064	1.05127697	0.02713192	0.23728674
LOC283194	9.6005265	0.96197045	0.02712955	0.23728674
ZCCHC24	2831.57915	0.33407174	0.02714461	0.23728674
PPTC7	360.357962	-0.4885756	0.02718576	0.23752702
EIF4H	2666.67296	0.2586488	0.02721001	0.23755311
PSENEN	217.50706	0.3905194	0.02722973	0.23755311
TTC22	1.47608749	-1.050152	0.02722827	0.23755311
MROH9	2.82990702	1.03815968	0.02724998	0.23761053
C10orf128	29.0221794	0.73816619	0.02728245	0.23762702
LOC1027248	302.46769	0.63350933	0.02729287	0.23762702

PCDHB17	44.2809041	-0.9241422	0.02728794	0.23762702
RHBDL3	88.1858549	-0.8601094	0.02731369	0.23768928
ZNF799	94.9182206	0.50034934	0.02732853	0.2376994
YIPF1	209.253192	0.45781954	0.02735027	0.2377696
C3orf70	257.453749	0.72783225	0.02737808	0.2378924
CYP2W1	10.4778669	-1.0079018	0.02743358	0.23799378
TTYH3	995.015923	-0.5938502	0.02740562	0.23799378
ZCCHC4	144.524958	0.4210944	0.0274445	0.23799378
ZNF384	812.383789	-0.3792345	0.0274283	0.23799378
CTAGE8	11.8415379	-0.8454339	0.02750684	0.23829669
SYNJ1	1063.77201	-0.4568981	0.02749751	0.23829669
MNT	460.607299	-0.4311802	0.02755498	0.2385948
MSR1	1122.472	0.76517209	0.02764574	0.23908202
NFIA	4695.35083	0.50357291	0.02765045	0.23908202
ZNF263	458.631253	0.24289028	0.0276525	0.23908202
TRMT2A	269.082734	0.32450134	0.02768218	0.23921969
LOC440311	1.58760966	-1.0302663	0.02771039	0.23927697
PIANP	20.1547217	-1.0366883	0.02771633	0.23927697
CADM2	8.84553976	-1.0244218	0.02776167	0.23943063
FBXO4	139.410242	0.41349648	0.02775451	0.23943063
KLRB1	4.40917614	0.97902083	0.0277866	0.23952686
COL26A1	168.995378	1.03137249	0.0278021	0.23954167
TMEM17	41.0920674	0.56000186	0.02783389	0.23969684
CIZ1	1006.58931	-0.2951435	0.02785692	0.23977634
PRPF3	893.608182	-0.4212349	0.02789542	0.23998893
HNRNPL	2039.33172	-0.1807739	0.027964	0.24045994
PPP1R7	467.31146	-0.4189074	0.02801042	0.24074008
ACTR3	2073.06319	0.22894459	0.02827137	0.24115809
ALG1L9P	28.2355557	0.6820289	0.02813935	0.24115809
COX6A1	1301.77256	-0.4599284	0.02822812	0.24115809
DLL1	99.9794692	0.97791376	0.02819264	0.24115809
ESR2	27.4881677	-0.9707338	0.02824681	0.24115809
FOXL2	1.91061421	-0.9983792	0.02827316	0.24115809
GABRB1	2.21531293	-0.9908772	0.02827155	0.24115809
LOC1005056	6.60667641	-0.8149844	0.02827936	0.24115809
ROCK1	1936.47062	0.41264077	0.02828098	0.24115809
SEPP1	3837.36251	0.76631073	0.02814344	0.24115809
SPECC1L	347.750616	0.41516206	0.02808477	0.24115809
TMC4	110.611116	0.93666905	0.02827263	0.24115809
TUBB1	10.0103888	0.99654438	0.02820181	0.24115809
WDPCP	145.120243	0.43077885	0.02820646	0.24115809
WFDC10B	2.7102209	1.02575326	0.0281136	0.24115809
WFIKKN1	3.49430515	-1.0317745	0.02809227	0.24115809
DAXX	2.18132962	1.03312409	0.02831041	0.24117254
FAM188A	425.822679	0.38147236	0.02829749	0.24117254
SMC2	458.197226	-0.3790248	0.02837149	0.24157449
LRRC25	154.145267	0.74158593	0.02838801	0.2415968
ABI2	1588.09546	-0.2244299	0.02842888	0.24182633
ATP5I	596.748727	-0.3759869	0.02844572	0.24185123
BSCL2	98.9082478	-0.4568161	0.02846294	0.24187934

KLHDC1	165.277197	0.61448016	0.02851091	0.24196269
PNKD	509.222999	-0.5047797	0.02851449	0.24196269
ZNF316	610.254601	-0.4104447	0.0285145	0.24196269
CTSE	1.74359092	-0.9131511	0.02855699	0.24207301
DHRS2	61.6674676	-1.0416588	0.02856927	0.24207301
TMIE	3.59024775	1.00916909	0.02855631	0.24207301
COL8A1	4766.84407	1.03381115	0.02859737	0.24215972
RECK	1388.12718	-0.5304479	0.02863521	0.24215972
RPS16	3764.08004	-0.651808	0.02862735	0.24215972
SON	12987.1743	-0.3197481	0.02861298	0.24215972
DBI	477.384109	-0.4450336	0.0287581	0.24233968
DUSP27	39.0080029	0.89970107	0.02874457	0.24233968
FAM72B	11.7650599	0.78445663	0.02876799	0.24233968
KATNBL1	330.67351	0.34263206	0.02874713	0.24233968
LY9	3.34822	1.0116067	0.02870403	0.24233968
PPIF	501.850804	-0.6684394	0.02870631	0.24233968
UBE2S	175.049783	-0.6541533	0.02869478	0.24233968
UVRAG	510.237376	0.35761816	0.02873016	0.24233968
ANXA7	1250.97796	0.36454752	0.02878225	0.24234239
SF3A2	488.462279	-0.4132267	0.02881175	0.24245974
TRIT1	176.837889	0.53209546	0.02882408	0.24245974
N4BP3	164.795274	-0.7782043	0.02887775	0.24279373
RNF38	1406.56878	-0.2226329	0.02891506	0.24298989
BTBD8	25.8296113	0.80026617	0.02893139	0.24300969
PABPC1	6572.32782	-0.4367733	0.02903672	0.24360824
RAB38	19.4078108	-0.9525181	0.02904468	0.24360824
UBXN4	2386.46795	-0.2646732	0.02902293	0.24360824
UBA7	751.064416	0.60678756	0.02911635	0.24409156
C1GALT1	491.396361	0.55677444	0.02916964	0.24430275
CHMP4C	73.2725944	-1.0358508	0.02916002	0.24430275
TAF10	409.21639	-0.4687711	0.02918503	0.24431399
MANEA-AS1	37.6129926	0.62146432	0.02920709	0.24438095
YBX3P1	5.60707308	-0.8753374	0.02923912	0.24453132
LURAP1L	158.239325	-0.9759252	0.02937835	0.24556629
LZTR1	823.072208	0.37694541	0.02939112	0.24556629
C20orf197	18.5921855	0.86821356	0.02944396	0.24576946
CLUH	788.136164	-0.4430084	0.02945784	0.24576946
TNIK	243.45394	0.91971437	0.02944546	0.24576946
CARF	450.817438	0.37394428	0.02953852	0.24588562
CRLF3	233.985765	0.47084252	0.02956407	0.24588562
FADS1	1134.11158	-0.8429896	0.02950928	0.24588562
MAPKAPK5-A	142.698748	0.36364934	0.02957076	0.24588562
PGF	993.816492	-0.9426984	0.02956328	0.24588562
2-Sep	6866.83478	-0.2768692	0.02956863	0.24588562
WNT9A	103.000363	-0.5986895	0.02951711	0.24588562
BHMT	23.6309174	0.98048819	0.02964521	0.24610089
CCL22	4.28369254	1.00005721	0.02965984	0.24610089
FOPNL	391.817337	0.34416315	0.02965182	0.24610089
LINC00417	21.8407874	0.91120929	0.02966742	0.24610089
MFNG	128.447871	0.67411958	0.02966305	0.24610089

SLC22A4	41.4563244	0.66172526	0.02970503	0.24629535
PCSK2	12.6119309	-1.0252625	0.02972395	0.24633477
EPHA6	7.43172728	-1.0312239	0.02973935	0.24634499
CYP2D7P	21.1510444	0.60225551	0.02977938	0.24655908
NAALADL2	150.442286	0.63472871	0.0298674	0.24717013
LOC729080	1.82990467	-1.0051258	0.02988915	0.24723243
MZF1	388.809985	-0.4162759	0.02990872	0.24727673
OCIAD2	83.8014111	-0.8405512	0.02993647	0.2473402
UNC5A	3.49804663	-1.0286366	0.02994485	0.2473402
ROGDI	291.872456	-0.4610736	0.02997633	0.24748264
FAM106CP	23.2047452	-1.0324095	0.02999199	0.24749439
GRM7	50.5610799	-0.9586207	0.03002835	0.24767691
SMPDL3B	15.6079415	-1.0218329	0.03005422	0.24777277
IGHMBP2	278.573861	-0.3009374	0.03008683	0.24792401
SNORA53	762.388792	-0.6696936	0.03010378	0.24794622
HSPA5	7384.48575	-0.7000995	0.03013111	0.24805378
PM20D1	8.05558329	1.02991409	0.03019773	0.24824957
RPL39	1464.92433	0.43363649	0.03019353	0.24824957
YWHAE	5386.98731	-0.3747037	0.0301836	0.24824957
LPCAT4	118.612118	-0.4651358	0.03023203	0.24829676
PPP1R1C	4.25093995	-1.0295253	0.03022303	0.24829676
NT5C2	2071.54906	0.50139497	0.03026479	0.24844848
CILP2	57.5657309	1.02498144	0.03032018	0.24878577
FLJ37201	27.3326022	0.77564309	0.03034607	0.24888073
ANO7	33.189716	-0.765824	0.03040081	0.24908734
NPW	49.094654	-1.0267467	0.03042958	0.24908734
PPP1R12B	2418.77574	0.44332694	0.03041518	0.24908734
RAD1	360.789756	0.35699653	0.030431	0.24908734
TMED1	260.162408	0.36802719	0.03044289	0.24908734
LGALS4	3.8645593	-0.9395184	0.03048867	0.24934453
GABRA2	2.58386773	-0.9898431	0.03054013	0.24964798
CNOT1	4162.53804	-0.3343013	0.03056389	0.24968022
GLS	2331.75842	0.62070117	0.0305728	0.24968022
PFKFB3	1196.27315	0.62419674	0.03065101	0.25018168
POTEF	9.77577132	-0.8079386	0.03066298	0.25018168
GOLGA8N	119.706053	0.54571469	0.03075154	0.25067957
KRTAP5-AS1	1.76275552	-1.0163574	0.03076725	0.25067957
TAS2R13	74.4867692	0.6507873	0.03075984	0.25067957
SEC16A	2977.57665	-0.3194636	0.03080685	0.25076721
SIRT7	117.562439	-0.4154089	0.03080644	0.25076721
FAM45B	12.1676615	0.64797468	0.03083902	0.25091157
SUPT3H	107.072298	0.69345907	0.03086394	0.25091307
ZNF410	441.712418	0.40915591	0.03086807	0.25091307
ACTR3C	25.830835	-0.6619959	0.03089857	0.25104365
NUP210	631.301645	-0.5449269	0.0309296	0.25117843
DEAF1	438.200457	-0.3292755	0.03101467	0.25147112
GALNT13	14.8042493	-1.0252868	0.03103875	0.25147112
GOLM1	1684.28937	-0.4968753	0.03106689	0.25147112
KIAA0100	5238.0351	-0.2815523	0.03101884	0.25147112
PLEKHA1	1156.35909	0.58104557	0.03104246	0.25147112

RAVER1	586.469172	-0.4096233	0.03098096	0.25147112
SFTPD	5.55003428	1.02331747	0.03106311	0.25147112
RAPGEF3	967.416952	-0.5602348	0.031104	0.25165435
TPRXL	6.72877117	-0.9423401	0.03112081	0.25167325
CD177	8686.63157	-1.001403	0.03117176	0.25196807
EIF4EBP3	42.8032088	-0.7631246	0.03120873	0.25214974
B4GALNT3	22.0902395	0.80238931	0.03124021	0.25227048
LAMTOR3	577.955848	0.29306656	0.0312527	0.25227048
HNRNPH3	1162.12577	-0.3536107	0.03132253	0.25259968
IBA57	466.399993	-0.383062	0.03132222	0.25259968
MSTN	20.87648	0.7404169	0.03141902	0.25326035
PDE2A	97.9535327	0.90196606	0.03152195	0.25397224
GRIA4	2.94025015	-1.0208396	0.03153904	0.25399225
KCNJ13	59.8457904	0.95936417	0.03158809	0.25403429
LOC1005066	5.27257744	1.01263623	0.03156907	0.25403429
TICRR	32.5133081	-0.9092545	0.03157459	0.25403429
FAM229B	283.705755	0.60403567	0.03168117	0.25442977
FAM26E	362.506306	0.69174444	0.03167818	0.25442977
TNFRSF10C	16.041332	0.87283315	0.03167245	0.25442977
BRIP1	97.4356108	-0.7754927	0.03176517	0.25469118
CD93	1841.3488	0.64245529	0.0317663	0.25469118
HADH	363.424091	-0.3303971	0.03177231	0.25469118
PIGK	523.976558	0.41876465	0.03175254	0.25469118
CDK1	83.1077465	-0.7440842	0.03181009	0.25487653
STK17A	516.192281	0.5131071	0.03182794	0.25490207
KRT14	35.2896569	-0.9290192	0.03186176	0.2549973
RPF2	279.903542	0.42586135	0.03186916	0.2549973
BET1	226.599881	0.26542838	0.03194943	0.25515481
CEP85L	438.217602	0.56305543	0.03199157	0.25515481
COL6A4P2	23.2813084	0.97394546	0.03198451	0.25515481
ESRRA	338.095416	-0.5579757	0.03198623	0.25515481
RAB8B	1442.46223	0.57380756	0.03192629	0.25515481
SGMS1	775.989973	0.42794088	0.03195488	0.25515481
TCTEX1D1	3.64934807	0.95780663	0.03191531	0.25515481
RIMS2	5.81810488	-1.0014582	0.03200849	0.25517266
ABO	40.4452288	1.00444209	0.0320307	0.25523268
HIST2H2BE	754.436763	-0.6410071	0.03205181	0.2552839
HIP1	862.214974	0.59700508	0.0321757	0.25615338
ABCC13	9.3123992	-0.9622002	0.03222055	0.25639298
CD302	35.5567428	-0.7578501	0.03232882	0.25659273
MAP1S	402.749446	-0.3685481	0.03228056	0.25659273
MFAP5	1780.12705	1.00243595	0.03233133	0.25659273
MORN1	58.8724376	0.66584871	0.03229395	0.25659273
MTERF	192.283013	0.34151933	0.03233842	0.25659273
NBEA	1068.86355	-0.5195477	0.03234895	0.25659273
ZNF579	106.219169	-0.5102617	0.03230863	0.25659273
PBDC1	204.548602	-0.4608272	0.03243798	0.25694719
SNHG21	45.569645	0.39907104	0.03243372	0.25694719
ZRANB1	1095.67798	0.36661588	0.03241275	0.25694719
SHB	737.855232	-0.6726187	0.03247336	0.25711036

DDX12P	104.241495	-0.645682	0.03249043	0.25712839
C9orf16	317.794992	-0.4008341	0.0325708	0.25717916
MYH14	712.063452	-0.8502224	0.03254292	0.25717916
NTRK1	2.89889109	-0.957311	0.0325594	0.25717916
QRFPR	28.312584	1.01017245	0.03252975	0.25717916
RDX	3597.57349	-0.5245057	0.0325464	0.25717916
C11orf80	88.5485413	-0.5232623	0.03261565	0.25734123
SV2B	17.1061879	-0.9925743	0.03263175	0.25734123
ULBP1	4.52073693	0.99365837	0.03263573	0.25734123
POF1B	63.2780644	-0.8952659	0.03269798	0.25759845
TNFRSF10D	240.268672	0.93405353	0.03269121	0.25759845
CAPS2	102.19301	0.54202835	0.0328256	0.25813603
LEPR	18695.1597	0.95409096	0.0328087	0.25813603
RPA2	399.540277	0.43748664	0.03281131	0.25813603
STARD4-AS1	23.4696881	-0.8249105	0.03278525	0.25813603
UBE2D1	185.24464	0.37388731	0.03289566	0.25856999
CACTIN-AS1	3.97012487	0.99207421	0.03291339	0.25859098
MIR181A1HG	53.466918	-0.6629919	0.03292807	0.25859098
SNORA28	4.21996007	0.86458776	0.03296239	0.2587436
ZFYVE9	1147.37377	0.50447793	0.03299893	0.25891351
CLSTN3	714.88059	-0.4444353	0.03301878	0.25895242
LINC00599	1.65128869	-0.9796687	0.0330574	0.25913842
ALDOA	9587.04594	-0.5566282	0.03313438	0.25944429
GPI	4045.66818	-0.5345555	0.03313871	0.25944429
TGM5	2.5881356	0.82786529	0.03315611	0.25944429
ZNF687	699.257902	-0.301315	0.03314226	0.25944429
ITM2C	13461.8973	-0.8203987	0.03318615	0.25954011
PRICKLE3	78.034731	-0.4920662	0.03319821	0.25954011
GPR132	92.2024032	0.80625709	0.03326639	0.25957517
GTF2B	197.608775	0.45775631	0.03323382	0.25957517
NCOA6	1422.81506	-0.4236846	0.03332193	0.25957517
PLEKHH3	615.502014	-0.5807605	0.03335855	0.25957517
PTCHD2	5.90659327	-1.011665	0.03325297	0.25957517
RUNX1-IT1	26.6330095	0.81742803	0.03334639	0.25957517
SCARNA12	714.151407	0.49946907	0.03336691	0.25957517
SEC14L1	2037.92264	-0.4408411	0.03336308	0.25957517
SLC35A3	424.410031	0.41423025	0.03331399	0.25957517
UBR2	1798.37249	0.31756913	0.03327663	0.25957517
YTHDF1	902.74001	-0.4198816	0.03334828	0.25957517
DOC2A	32.5693249	-0.8965832	0.03339072	0.25964422
FLT3	6.51196141	1.00689467	0.03345	0.25966781
MIR612	2.62414733	-0.937963	0.0334134	0.25966781
PDLIM2	440.868916	0.68969736	0.03345635	0.25966781
TIMMDC1	451.925978	0.27875804	0.03346843	0.25966781
WDR92	110.455294	0.40440628	0.03346487	0.25966781
EIF5AL1	8.87422587	-0.8061487	0.03349143	0.25973034
NPPA-AS1	6.18480862	0.89286365	0.03353727	0.25996995
DTHD1	4.10677117	0.9973754	0.03357389	0.26002195
ZNF285	88.7450096	0.47082396	0.03357035	0.26002195
FAM151B	43.7755245	0.54721059	0.03360574	0.26003691

FRG1	191.350772	0.22386993	0.0335967	0.26003691
NUDT12	365.079779	0.60855795	0.0336459	0.26016499
RBX1	228.861031	0.4026856	0.03365221	0.26016499
ALDH3A1	4.44993743	-0.9917011	0.03374344	0.26024523
ATP7A	772.695741	0.26486307	0.03375043	0.26024523
DKFZP586I14	268.440952	0.55211716	0.03378234	0.26024523
DYRK4	203.449138	0.36925473	0.03377017	0.26024523
HERC2P7	46.343404	-0.5237765	0.03369772	0.26024523
LOC1001303	1.51491822	1.00229542	0.03375058	0.26024523
LRRC37A11P	13.7574548	-0.9362379	0.03374727	0.26024523
TRMU	122.978278	0.44229808	0.03377568	0.26024523
TP73	9.03126914	-0.9353175	0.03381513	0.26038246
SCN4B	682.149213	0.91624896	0.03383874	0.26044894
DEPDC1	29.3538541	-0.7522466	0.03388682	0.26058828
PRKCB	168.730706	0.70645899	0.03387915	0.26058828
PRR24	106.408013	-0.3411223	0.03395932	0.26103038
KPNA2	404.120769	-0.5136959	0.03399266	0.26117122
YY1AP1	928.926095	-0.2672628	0.03406707	0.26162727
CCDC175	14.9213095	0.997589	0.03409939	0.26173542
SNX30	654.993546	0.46289364	0.03411126	0.26173542
STXBP1	544.753885	-0.4085159	0.03417583	0.26211517
EPB41L1	1761.53547	-0.4547256	0.03419405	0.26213934
MBD6	899.441666	-0.4306399	0.0342595	0.26252529
AQP11	15.694978	-0.6946091	0.03429455	0.2625797
CELF3	2.345369	-1.0066138	0.03431993	0.2625797
ENDOG	90.0832287	-0.6635248	0.03430434	0.2625797
PRPS2	291.605368	-0.3044003	0.03432782	0.2625797
SLC6A16	21.836444	-0.9207346	0.03434211	0.2625797
CENPP	153.120335	-0.5652788	0.03436163	0.2626135
EZR	3787.78277	-0.6956582	0.03438529	0.26267888
GCLM	224.552565	0.65868474	0.03448515	0.26332602
ENO1	5423.13343	0.47077332	0.03456116	0.2634656
GPR37	12.7618819	-1.0053798	0.03451992	0.2634656
PFDN4	105.280458	0.43888168	0.03455707	0.2634656
RBPMS	570.196153	-0.6158569	0.03456404	0.2634656
GATAD2A	1007.69208	-0.4070371	0.03459536	0.26358873
KIN	283.250612	0.31670163	0.03462356	0.26368804
ARHGEF2	1720.35952	-0.371447	0.03468187	0.26401649
APC2	24.285472	-0.8965719	0.03480383	0.26412823
FIRRE	46.7012616	-0.9746658	0.03479986	0.26412823
FOXO4	180.862643	0.5715275	0.03477741	0.26412823
LOC1002889	34.8548662	0.84667121	0.03471777	0.26412823
NEK11	137.053172	0.62740364	0.03481808	0.26412823
NOC3L	410.349646	0.44337025	0.03476272	0.26412823
SLC34A2	5.37077976	-0.979904	0.03480952	0.26412823
STIP1	1026.56816	-0.4568712	0.03473865	0.26412823
ADC	72.3678863	0.63101383	0.03501644	0.26459398
ARID5B	1513.06533	0.84319878	0.03499948	0.26459398
FSCN2	27.2874152	-0.8529285	0.03501473	0.26459398
JAKMIP1	6.04890863	0.99025493	0.03496603	0.26459398

PPP1R14A	9.86507382	-0.9199968	0.03493289	0.26459398
SNORA14B	5.26042776	0.87637688	0.03492122	0.26459398
SRPX	138.008018	0.97601978	0.03491352	0.26459398
TRPM5	1.53016188	0.99546159	0.03497962	0.26459398
TTC39C	58.6416238	0.76307779	0.03499878	0.26459398
NFIB	2777.02954	-0.6188451	0.03507957	0.26491273
RNF11	1805.62384	0.43453384	0.03508909	0.26491273
HAUS1	158.827873	0.51960255	0.03515599	0.26530261
UBTF	2544.03829	-0.3406819	0.03519528	0.26548384
AGPAT3	1635.03922	-0.4321913	0.03524045	0.2655292
FCGRT	2874.28949	0.3598767	0.03523853	0.2655292
LOC1001295	17.7800253	0.69886227	0.0352471	0.2655292
SYNGR3	7.46182862	-1.000781	0.03531112	0.26589629
VTI1A	552.972445	0.40125071	0.03536642	0.26619737
SCARB2	3176.64916	0.32583301	0.03546977	0.26662903
TTLL7	27.760018	-0.982668	0.03546054	0.26662903
WLS	1798.1894	0.59845173	0.03545774	0.26662903
SYNE3	151.007644	0.73825873	0.03550049	0.2667446
RPL13A	1810.6061	0.45987168	0.03555754	0.26705783
CYTH2	633.32164	-0.363564	0.03563276	0.26739181
NPHP3-ACAD	119.16256	0.55941334	0.0356206	0.26739181
MYOZ3	169.77601	0.94427203	0.0356881	0.26769152
GUCY1A2	116.427027	0.85916668	0.03579798	0.26778011
KIAA2018	2143.72539	-0.3580921	0.03575328	0.26778011
KLF14	3.63582504	0.97978409	0.03577909	0.26778011
LSS	1731.01942	-0.5449273	0.03578425	0.26778011
NEK7	1144.26013	0.2764103	0.03582312	0.26778011
NXPE3	364.574639	0.51878102	0.03580795	0.26778011
SLC4A4	24287.7123	-0.7122558	0.03571637	0.26778011
SNAI3	22.5751323	0.94733467	0.03574754	0.26778011
RASA4B	28.1553011	0.58545035	0.03593888	0.26852974
ZNF695	9.3408885	-0.9495857	0.03595429	0.26852974
GGN	4.97282166	-0.8823928	0.03599394	0.26860974
NDUFAF3	578.807609	-0.4734766	0.03599826	0.26860974
UBP1	774.241121	-0.3224984	0.03601135	0.26860974
GRM5	1.94054753	-0.9651215	0.03605907	0.26873514
LINC00475	13.2997491	0.91066201	0.03605321	0.26873514
THUMPD1	1243.52995	0.31078916	0.03607633	0.26874857
CHST7	83.5367891	0.82950705	0.03609226	0.26875212
BOP1	172.01269	-0.411079	0.03616518	0.26902302
GPR83	97.6404571	-0.9538398	0.03616162	0.26902302
PDZD4	1529.21641	-0.502252	0.03620218	0.26902302
PRKAG2	2585.51782	-0.8612053	0.03622148	0.26902302
RAB13	624.048858	-0.4492747	0.03621693	0.26902302
ZNF818P	222.047814	0.53413216	0.0361985	0.26902302
C6orf147	14.0166211	-0.9825537	0.03623915	0.26903934
C16orf86	35.1161305	0.86524828	0.03625656	0.26905369
NLRP1	391.065987	0.7488479	0.03628582	0.26905662
PIK3C2B	2399.41825	-0.3985507	0.0362879	0.26905662
NAGLU	460.658084	0.53557116	0.03642864	0.26975503

PRDM6	1031.12253	0.65345811	0.03641457	0.26975503
PRPF18	260.376656	0.38539382	0.03642342	0.26975503
AQP6	1.86246211	-0.9932409	0.036498	0.2700393
HLA-DQB1	650.52169	0.98826468	0.03649824	0.2700393
PTDSS2	506.299136	-0.3850659	0.03651363	0.2700393
RGS7	4.41832602	-0.9262263	0.03659497	0.2705258
NBEAL1	1337.42368	0.22721845	0.03662948	0.27066586
GNG12-AS1	1.72735871	0.99228241	0.03667998	0.27080881
PRIMPOL	210.852062	0.37767819	0.03666731	0.27080881
CHST1	61.5982223	0.77175536	0.03676206	0.27129962
CCDC40	156.857752	0.66117743	0.03681906	0.27133205
FBXO2	43.8409303	-0.89311	0.03682887	0.27133205
NDUFS1	1488.33693	-0.383188	0.0368121	0.27133205
ZNF552	100.004044	-0.3929919	0.03680738	0.27133205
ACAT2	258.737226	-0.5988931	0.03684623	0.27134491
ADAMTS9-AS	78.5517199	0.92595895	0.0368785	0.27146758
C11orf53	4.24123763	-0.953657	0.03690322	0.27153458
C5orf45	170.999118	0.43836375	0.03692222	0.27155949
SNORA61	5.62742004	0.85225086	0.0369475	0.27163055
PLXDC2	3376.97911	0.70736671	0.03701331	0.27193911
VAT1	1964.90691	0.4269666	0.03702076	0.27193911
GATAD1	1029.08666	0.36183963	0.03704203	0.2719805
WHAMMP1	142.186605	0.41490622	0.03706871	0.27206151
ATP6V0E2-A	85.7262469	-0.6959897	0.03710506	0.27216435
BACE1	1317.29991	-0.4454027	0.03731357	0.27216435
BEX1	39.8136419	-0.9719247	0.03726458	0.27216435
CCDC88B	272.950148	0.79728827	0.03718194	0.27216435
CELSR2	474.279088	-0.5960277	0.03729918	0.27216435
DDX42	2339.46004	-0.2426831	0.03738784	0.27216435
FAM154A	3.89490558	-0.9360304	0.03732257	0.27216435
FER1L4	439.225114	-0.8545348	0.03712461	0.27216435
HSPD1	2913.14853	-0.5110863	0.037182	0.27216435
LINC00276	4.40037068	0.96148825	0.03726922	0.27216435
LINC01105	5.44053071	-0.9694252	0.03735107	0.27216435
MPPE1	397.052622	0.41599255	0.03726518	0.27216435
MVD	235.281159	-0.4819	0.03720862	0.27216435
NAT8L	117.880009	-0.7379688	0.03717656	0.27216435
NRGN	29.4861336	-0.9634807	0.03716261	0.27216435
OSMR-AS1	9.00876576	0.92956971	0.03740528	0.27216435
OVCA2	19.3162338	-0.6679967	0.03728548	0.27216435
PPP6R1	976.752379	-0.3332594	0.03740089	0.27216435
SLC2A14	12.6375939	-0.9183478	0.03738874	0.27216435
SRCIN1	30.5937671	-0.8692926	0.03726183	0.27216435
WNK2	232.285351	-0.9785639	0.03741145	0.27216435
CSMD1	9.1170282	-0.9754298	0.03745814	0.27238172
EPN2-IT1	11.3672404	-0.645082	0.03748832	0.27238172
PI15	12.5939787	0.9589599	0.03747694	0.27238172
FAM135A	582.849453	0.34353757	0.03755554	0.27264223
WDR83OS	634.424847	0.34802627	0.03755534	0.27264223
AGAP1	939.829378	-0.5406648	0.03761507	0.27294446

ANK3	615.808876	0.88334961	0.03765996	0.27294446
EZH1	1096.08695	0.43575812	0.03765595	0.27294446
ZNF664	2470.60067	-0.4979881	0.03765391	0.27294446
PGAP2	188.713815	-0.3836286	0.03768181	0.27298904
ERCC3	718.457412	-0.2422016	0.03769852	0.27299632
QRICH2	216.573469	0.39113794	0.03774364	0.27320925
FN3KRP	398.442907	-0.3284363	0.03777543	0.27323028
SYT15	406.383181	0.91497595	0.03777797	0.27323028
TGFB1	580.030978	-0.5346668	0.03789271	0.27394617
DNAH12	58.1714389	0.81071247	0.03798345	0.27437399
TPRG1	17.463735	0.78444605	0.03798056	0.27437399
CKLF	58.5061326	0.63148063	0.03805237	0.27475773
CACNA1B	12.0039185	-0.8751986	0.03807842	0.27483166
MYOZ2	5.18289622	0.83093099	0.03810704	0.27492412
PHLPP2	1183.73254	-0.562468	0.03812337	0.27492783
SIL1	686.971136	0.33283409	0.0381761	0.27519397
FAM64A	10.391731	-0.8525901	0.03830648	0.27601935
CCDC144CP	66.9508236	-0.905738	0.03832646	0.27604897
TIAF1	11.8817602	0.75044325	0.03841384	0.27656372
HERC2P4	32.1324526	0.82055504	0.03851397	0.27679492
KYNU	51.9773702	0.75095315	0.03852555	0.27679492
MSANTD4	432.318757	-0.3550136	0.03849801	0.27679492
NMRAL1	259.075588	-0.3941105	0.03848415	0.27679492
TMEM135	771.840731	0.42894507	0.03850375	0.27679492
NPHP3	217.800124	0.38648862	0.03855988	0.27686616
SGK1	3291.86491	0.72493874	0.03856731	0.27686616
DDX60L	700.60564	0.60851751	0.03869754	0.27758989
DIP2B	3919.77241	-0.4690283	0.03879772	0.27758989
FASTKD1	276.731299	-0.4789059	0.03880436	0.27758989
ID1	4110.57193	-0.7231956	0.03882778	0.27758989
LRRTM3	9.40772064	0.98000564	0.0387396	0.27758989
PNRC1	3128.78958	0.43819271	0.03875454	0.27758989
RAB14	1924.84922	-0.2612497	0.03874292	0.27758989
RORB	1.62774479	-0.9797824	0.03881327	0.27758989
SERPINB10	8.33163944	-0.9144987	0.03877234	0.27758989
SLC25A45	147.61706	0.62118175	0.03873417	0.27758989
ATXN3	581.57186	0.44100273	0.03892935	0.27764708
GINS2	42.0889261	-0.8352946	0.03892591	0.27764708
GPATCH2L	1484.39881	0.36816066	0.0389414	0.27764708
KIAA1107	109.141259	0.63050183	0.03885714	0.27764708
TLR5	148.833926	0.74492331	0.03894756	0.27764708
TOB2P1	10.9223073	-0.8764541	0.03889478	0.27764708
ZNF420	203.638408	0.33751207	0.03886877	0.27764708
ITIH2	1752.42222	0.93301819	0.03904268	0.27821113
B4GALT6	216.64172	0.76572121	0.03908714	0.27829976
FCGR1C	34.4507193	0.84471474	0.0390761	0.27829976
LCMT2	267.380127	-0.3066983	0.03911401	0.27837709
FKBP9P1	24.7579199	-0.5828345	0.03916063	0.27848095
RNF215	144.334746	0.45272736	0.03915016	0.27848095
LNX2	655.694719	-0.4972547	0.0391951	0.27849827

TECR	981.190268	-0.5920261	0.0391848	0.27849827
ATP2A2	3946.51702	-0.3700324	0.03923312	0.2785407
ZNF546	176.07313	0.36932821	0.03922869	0.2785407
HNMT	808.408315	0.5708467	0.03928812	0.27858609
NELFB	549.339786	-0.2324227	0.03926873	0.27858609
PRDM11	492.612181	-0.5537795	0.03928216	0.27858609
SHC1	2585.34972	-0.3836242	0.0393036	0.27858609
PKD1L1	29.6010199	0.59083965	0.03933829	0.27871835
CAMKV	1.95861817	-0.9558379	0.0394467	0.27894917
CXorf56	286.172069	0.31128574	0.03944325	0.27894917
EXD2	418.322305	0.46965836	0.03947098	0.27894917
FOXD3	25.5408077	-0.8422214	0.03948317	0.27894917
MRPL38	379.883053	-0.3578318	0.03941678	0.27894917
OTUD3	342.720203	0.54628104	0.03941529	0.27894917
PHC2	939.108051	0.56937968	0.03947721	0.27894917
SKIV2L2	1139.41776	0.22666279	0.03952093	0.27910249
CACNB1	106.946898	-0.4259483	0.03968374	0.27968375
GALC	475.483135	0.49014461	0.03971583	0.27968375
GTSE1	22.783406	-0.7654897	0.03968434	0.27968375
KIAA1671	4178.60918	-0.4680113	0.03971123	0.27968375
LINC01265	1.61050231	0.95983242	0.03968306	0.27968375
USP24	2514.80313	0.39673101	0.03969653	0.27968375
ZNF674	102.182652	0.41269988	0.03971143	0.27968375
OSBPL7	301.615877	-0.3514729	0.0397483	0.27979907
COQ3	34.8927639	0.47306689	0.03988284	0.27998788
GBP4	262.006052	0.66867836	0.0398918	0.27998788
ID2	898.420445	-0.4995397	0.03986119	0.27998788
KIAA1045	11.2153267	0.90206335	0.03990395	0.27998788
LOC1005070(	10.033383	0.9612159	0.03981877	0.27998788
LSM12	247.51395	-0.4094184	0.03989536	0.27998788
SNORA22	82.3157269	-0.7239715	0.03990095	0.27998788
TMC6	650.804923	-0.5914718	0.03986002	0.27998788
CDKL3	50.8509108	0.5320423	0.03994706	0.28002269
CLPB	432.194066	-0.3884781	0.04000713	0.28002269
DECR1	543.588583	-0.6387604	0.04006019	0.28002269
EIF4G1	5224.34861	-0.296015	0.04006996	0.28002269
EMBP1	14.9684546	0.77437996	0.03992694	0.28002269
HPGD	212.75152	0.92319221	0.04004075	0.28002269
METTL12	19.3847977	0.68775744	0.0400079	0.28002269
RPS19BP1	273.786213	0.4133929	0.03999735	0.28002269
USP4	1133.65029	-0.3290514	0.04004966	0.28002269
UTP6	497.334396	0.27866161	0.04006333	0.28002269
CBWD6	17.4164562	0.63629035	0.04013679	0.28037703
C16orf45	174.210899	0.8525631	0.04017666	0.28043018
PPIE	306.342668	0.31910308	0.04017501	0.28043018
TACR2	2.0019773	0.97449705	0.0401972	0.28046096
LOC286189	1213.88053	-0.9699566	0.04022334	0.28053076
TBC1D10C	21.3504227	0.84287821	0.04025345	0.28062821
MON1B	625.888486	-0.2900557	0.0402717	0.28064289
GUSBP1	289.508559	0.38775115	0.04034543	0.28104405

NOTCH2NL	1812.35424	0.40534241	0.04036501	0.28106786
ADAMTS5	1435.55644	-0.9749045	0.04040562	0.28112546
RPS6KA2	1477.71851	-0.6975398	0.04039187	0.28112546
DLEU2L	30.1131164	0.64951506	0.04044899	0.28131466
KIAA0247	3463.6778	0.58145639	0.04047187	0.28136121
CDPF1	67.310882	0.46731311	0.04056973	0.28192882
CLEC4E	71.3540307	0.93012997	0.04075964	0.28246006
HCG22	5.93490261	-0.9715639	0.04071886	0.28246006
LINC00323	2.7576419	0.96089158	0.04071497	0.28246006
PTGER4	309.770798	0.78655975	0.04077254	0.28246006
RP2	362.398583	0.35725184	0.04070312	0.28246006
SLC22A18AS	5.34057598	-0.9057135	0.04068452	0.28246006
TRNAU1AP	132.166352	0.50026665	0.04075716	0.28246006
WASH2P	241.322685	0.30790242	0.04077614	0.28246006
ZMIZ2	1118.4873	-0.412152	0.04084787	0.28284425
EVC2	263.517941	0.47527855	0.04096566	0.28308504
HCST	62.2666162	0.84821338	0.04097885	0.28308504
LILRB3	29.3745786	0.84531263	0.04096135	0.28308504
MT1M	13.5097112	0.97096011	0.04099434	0.28308504
MXD1	166.900415	-0.5213509	0.04099661	0.28308504
PTK2	2184.08681	-0.313606	0.04099124	0.28308504
SNHG3	255.161288	0.44215988	0.0409767	0.28308504
SLC25A36	1329.68039	-0.1728363	0.04116008	0.28410098
BAIAP2	431.548201	-0.5619958	0.04118086	0.28413159
ETV7	20.7666495	0.76900607	0.04122356	0.28420065
UBA52	3056.02804	0.24701963	0.04122226	0.28420065
SNAPC4	268.847905	-0.3488743	0.04124692	0.28424897
CXCR3	3.04927541	0.92420772	0.04133557	0.28447916
LOC1005061	1.79249043	-0.9706678	0.04129708	0.28447916
NFYA	846.842545	-0.3650932	0.04133834	0.28447916
SETDB1	816.874861	-0.2409091	0.04134577	0.28447916
ANKRD23	96.223187	-0.4318454	0.04150213	0.28487859
ARHGEF5	274.458753	-0.6868734	0.04150168	0.28487859
COL20A1	1.5024451	-0.9296306	0.04148906	0.28487859
LRRC69	11.0172308	0.71104639	0.04146291	0.28487859
PPP2R2C	67.9105492	-0.925748	0.0414429	0.28487859
TM2D1	267.65153	0.47012112	0.04147922	0.28487859
LOC1005056	1.976428	0.96813859	0.04163031	0.2855445
LSR	605.967045	-0.6954349	0.04163198	0.2855445
C11orf44	3.34314401	0.95579203	0.04171523	0.28559111
EME1	22.828361	-0.7525498	0.04174326	0.28559111
ENOSF1	516.889486	0.48468703	0.04176191	0.28559111
SC5D	790.465922	-0.4000192	0.04168044	0.28559111
SLC25A33	50.5915034	0.52880828	0.04168847	0.28559111
SSR4P1	22.3712592	0.80388528	0.04177018	0.28559111
STRIP2	393.494214	0.73135434	0.04172355	0.28559111
TST	128.347208	0.61884557	0.04172484	0.28559111
BCAM	568.944732	-0.6035557	0.04182968	0.28564772
C15orf26	3.15347247	0.95206342	0.04182089	0.28564772
NALCN-AS1	3.36293138	0.90632322	0.04186061	0.28564772

RPL34	3177.36785	0.39208666	0.04181979	0.28564772
UNK	789.817844	-0.3916629	0.04184508	0.28564772
PTBP1	2385.01573	-0.2631175	0.04190619	0.28584658
ADAMTS19	5.3261175	-0.9626264	0.04195864	0.28597993
RTKN	268.719071	-0.4798178	0.04195235	0.28597993
GRAP2	9.70566292	0.87476423	0.04203423	0.28615864
RTCB	599.536946	0.41546102	0.04202577	0.28615864
SLED1	25.0878902	0.86358713	0.04200634	0.28615864
TTC37	2394.99473	0.26702345	0.04211414	0.28647828
ZNF79	186.869571	0.23541471	0.04210415	0.28647828
NFX1	1195.24434	-0.1951308	0.04214364	0.28656689
IFI44	285.241207	0.59863159	0.0422413	0.28711865
DLGAP1-AS1	88.9253748	0.50366055	0.04230148	0.287187
GABRB3	109.156666	-0.949475	0.04231394	0.287187
LINC00467	48.1299689	-0.5490448	0.04231743	0.287187
TTC38	187.246504	0.29736853	0.04229642	0.287187
ADAMTSL4-A	102.696984	0.61427842	0.04238578	0.28753865
ALKBH6	64.143404	-0.371156	0.04244112	0.28757743
CABP4	9.76031284	0.85237259	0.04241314	0.28757743
CCDC22	161.935695	0.26618439	0.04243777	0.28757743
ITPKB	5621.1047	-0.6236363	0.0424676	0.2876448
CKAP2	501.713985	-0.5279695	0.04252181	0.2876468
FANCI	241.138936	-0.5835618	0.04253138	0.2876468
MGP	3466.97637	0.70719482	0.0425319	0.2876468
POTEE	35.4809727	-0.7103817	0.04253407	0.2876468
UBE2Q2L	6.5495613	0.90795395	0.04257327	0.28779993
AHCTF1P1	9.57267473	-0.584194	0.0426179	0.28796384
OSTF1	258.101705	0.35996855	0.0426472	0.28796384
VAV1	198.851952	0.70774269	0.04263493	0.28796384
C16orf13	273.156004	-0.4253561	0.04268495	0.28799505
FAM214A	1066.45972	-0.2976284	0.04267105	0.28799505
GSE1	961.637756	-0.500252	0.0427313	0.28819594
LOC730668	1.78567214	0.96328442	0.04275556	0.28824774
CDK15	8.91276692	0.81768196	0.0428419	0.28838986
CNTLN	892.186498	0.39205643	0.04285587	0.28838986
GYS1	1074.6438	-0.4524089	0.04284256	0.28838986
LOC1002870	28.870373	0.49861823	0.04285957	0.28838986
TRIB1	354.230735	0.76633852	0.04285382	0.28838986
TRAPPC8	1177.13316	0.34341445	0.04295208	0.28890051
USH1G	3.39075486	-0.9622049	0.04297944	0.28897273
CROCCP2	217.771915	0.58548456	0.04304754	0.28931872
METTL4	173.834149	0.5205981	0.04312085	0.28969946
SHC4	331.458492	0.9511609	0.0431733	0.28993981
FIBIN	2512.43172	0.95311768	0.0433223	0.29004424
LMO3	257.411734	-0.9039116	0.04326791	0.29004424
MAOB	23.0810582	-0.9566028	0.0432092	0.29004424
NBPF1	1058.6085	0.59269936	0.04331653	0.29004424
PIP5KL1	8.72167291	-0.8441203	0.04331675	0.29004424
SCNN1A	265.462006	-0.904087	0.04323406	0.29004424
SELP	10.6456268	0.94193629	0.04328746	0.29004424

TP53INP2	947.562047	-0.6328973	0.04326257	0.29004424
EMC10	1546.48423	-0.3530551	0.04335669	0.2901627
MIR497HG	109.841497	0.91303159	0.0433977	0.29032543
TNS3	2943.11639	0.80248531	0.04343138	0.29043903
ANKRD12	2187.73232	0.46872549	0.043476	0.29062559
FBXL19	330.298453	-0.3100256	0.0435186	0.29068684
ZBED3	60.9603196	-0.3935176	0.04351235	0.29068684
LOC1019290	1.77766921	-0.9503286	0.04355859	0.29074106
SIRPG	4.37519364	0.95645146	0.04356016	0.29074106
HUWE1	9730.49811	-0.2625652	0.04358138	0.29077108
DYNLL2	616.044834	-0.4173644	0.04361754	0.2907892
WASF1	461.888544	-0.5305168	0.04360794	0.2907892
HCRTR2	4.31407248	-0.8998055	0.04365774	0.29083846
MYBPC1	3.65131557	0.95932228	0.04365839	0.29083846
SLC2A13	213.268905	0.60376678	0.0436965	0.29098089
ZMYND10	12.264362	-0.8560196	0.04371423	0.29098751
FGF13	8.18410415	-0.8882587	0.0439075	0.29193871
KIF24	85.522181	-0.562611	0.04390097	0.29193871
MTF2	351.699367	0.42817369	0.04390619	0.29193871
PSMD1	1420.84667	-0.2526515	0.04396231	0.29206771
9-Sep	1748.75227	-0.4937572	0.04394986	0.29206771
SLC38A5	38.4038245	-0.9583982	0.0439773	0.29206771
SWSAP1	34.4959614	0.53393653	0.0440626	0.2925225
BRSK2	19.7095977	-0.8588042	0.04418768	0.29254192
CD27	3.71353994	0.90592269	0.04410482	0.29254192
KCNC3	539.258124	-0.8343294	0.04410171	0.29254192
LINC01301	7.06696249	0.78560787	0.04415588	0.29254192
LRMP	96.1043249	0.67402319	0.04418981	0.29254192
MED7	155.382569	0.36791183	0.04421261	0.29254192
SEC61G	219.102531	0.51558555	0.04414365	0.29254192
TMEM41B	787.160006	-0.404528	0.04419822	0.29254192
WIPF1	925.040568	0.38878534	0.04421695	0.29254192
COL7A1	348.596038	-0.7535239	0.04431774	0.29291774
GNB1L	27.6817308	0.57914862	0.0443243	0.29291774
LMBR1	999.988112	-0.3653125	0.04430995	0.29291774
AKNA	719.385753	0.46354284	0.04437634	0.29315024
INTS5	349.558358	-0.3500775	0.04440249	0.29321155
GATSL2	337.483828	-0.3708043	0.04444761	0.29328675
ULK2	730.680421	0.38070753	0.04443386	0.29328675
FBN2	2872.10129	-0.7653572	0.0444648	0.29328888
LTBP1	2896.08338	0.85791888	0.04449028	0.29334569
EVI5	978.685723	0.45161334	0.04453615	0.29341793
MRAP2	153.979706	0.91878261	0.04455187	0.29341793
SCNN1D	89.680802	0.71211548	0.04455012	0.29341793
DR1	582.102461	0.37801972	0.04465429	0.29397323
RNGTT	446.381462	0.39747349	0.04467	0.29397323
ZNF837	16.6585097	-0.5427916	0.04469781	0.29404493
NAT6	117.547097	-0.3856552	0.04476432	0.294265
PARK2	103.664163	0.44447917	0.04476511	0.294265
SFRP1	1520.13925	0.95455218	0.04479514	0.29435113

DUSP5	359.280666	0.91539134	0.04489438	0.29444681
GOPC	851.428014	0.35783465	0.04488667	0.29444681
PRKG1	436.752065	0.8351847	0.04486087	0.29444681
PTPRO	94.9805703	0.72649477	0.04486459	0.29444681
WBP11P1	1.73634311	-0.9348516	0.04482732	0.29444681
GPNMB	1342.43008	0.85043772	0.04492371	0.29452813
DMGDH	199.327808	0.74540831	0.04496428	0.29468298
PLXNB1	2311.72592	-0.4901985	0.04504786	0.2951195
LOC283731	3.17339451	-0.9497984	0.04506819	0.29514148
WDSUB1	175.170855	-0.380831	0.04513163	0.29544568
NDUFA3	506.233513	-0.405626	0.04518398	0.29561382
SYCE1	8.86085681	-0.9517531	0.04519132	0.29561382
BTAF1	1767.50489	0.17800532	0.04520866	0.295616
GNPDA1	359.808631	0.3257884	0.04524951	0.29577188
C1orf127	1.6519177	0.95166105	0.04526675	0.29577339
POLD2	674.226927	-0.3794372	0.04536549	0.29630719
ISM1	121.316402	0.80609013	0.04541284	0.29650507
SBNO1	2025.96316	-0.2600983	0.04548049	0.29683533
PIPOX	10.7821296	0.80079089	0.04554955	0.29717451
CKB	543.700268	-0.818191	0.04559414	0.29735384
ZYG11B	1407.04465	0.40738391	0.0456588	0.29766388
C1QL1	95.9093743	-0.7844572	0.04574449	0.29793183
NLRC3	32.2614304	0.65548339	0.04573992	0.29793183
ZNF75A	298.591548	-0.3565064	0.04575131	0.29793183
DOK2	25.7160803	0.8144571	0.0458229	0.29823291
WWC2-AS2	19.7000961	0.72035755	0.04583185	0.29823291
ANO5	281.493821	-0.6538425	0.04585117	0.29824702
FAM182A	5.33468726	0.94791167	0.0458933	0.29840944
LOC284837	11.4445821	0.91360546	0.04598457	0.29880105
RDH16	4.14568512	0.88000874	0.0459879	0.29880105
ANKAR	238.662382	0.43771399	0.04616632	0.29950803
CDCP1	73.9957181	0.82021306	0.04618284	0.29950803
FBXW5	828.395571	-0.2794455	0.04618045	0.29950803
SCARB1	825.771898	-0.5199057	0.04615642	0.29950803
SNAI1	19.4417949	0.91833427	0.04616909	0.29950803
MIR22HG	653.439819	-0.4608408	0.04631308	0.30012881
USP32P1	358.63198	-0.9432735	0.04631262	0.30012881
AKAP12	19704.2877	-0.7720792	0.04633769	0.30017635
CLYBL	85.1579792	-0.469573	0.04637196	0.3002865
CD53	485.928798	0.66395396	0.0464503	0.30068179
C15orf52	608.184476	-0.6403437	0.0465194	0.3008368
CDH5	1483.66981	0.6456579	0.04652615	0.3008368
RAB24	751.407439	-0.5427453	0.0465099	0.3008368
CCDC183-AS1	89.6165992	-0.4844973	0.04654886	0.30084915
SGTB	283.356978	0.49289144	0.0465803	0.30084915
TMED5	816.950538	0.48449716	0.04659727	0.30084915
TOP1	1784.25218	-0.3280322	0.04659456	0.30084915
AURKA	48.2167656	-0.7498923	0.04662376	0.30090843
PIP5K1A	1201.38879	-0.3162666	0.04666682	0.30107456
CD8B	4.98520529	0.9243526	0.0467169	0.30128587

ABCA2	1113.26001	-0.6230317	0.04693734	0.30237109
CRIP2	881.359008	-0.5014141	0.04693264	0.30237109
NFATC1	141.036928	0.53192557	0.04691361	0.30237109
GMFB	926.104862	0.39830045	0.04698399	0.30244283
RUNX1	794.618285	0.61779157	0.04700066	0.30244283
ZNRF1	137.344578	-0.4064314	0.04698042	0.30244283
TRAF3IP2-AS	116.932994	0.68729841	0.04702317	0.30247573
NCAPD3	474.440757	-0.3325282	0.04709418	0.30248488
NOMO2	245.96431	-0.4276906	0.04708711	0.30248488
SUN2	1042.3393	0.37276993	0.04706847	0.30248488
ZNF322	280.01766	0.34456843	0.04708386	0.30248488
ASTE1	130.865478	0.35352731	0.04721051	0.30258722
CCDC28B	30.9187655	-0.5734882	0.04720531	0.30258722
FAM107B	374.274453	0.61565622	0.04721072	0.30258722
FANCC	205.108414	-0.4764945	0.04724934	0.30258722
HUS1B	5.50991741	0.73628231	0.04723877	0.30258722
LOC1001283	55.9449318	-0.6826616	0.0472349	0.30258722
PAK6	61.678803	-0.7186164	0.04722083	0.30258722
PSMB10	245.533605	0.46123952	0.04724287	0.30258722
RPH3A	3.86726321	-0.9281386	0.04728725	0.3026071
TSC22D3	5893.87706	0.69254925	0.04727999	0.3026071
ECM2	2114.20237	0.58065472	0.04734936	0.30276786
LINC00630	107.746742	0.30774267	0.04741686	0.30276786
NUDT8	29.988286	-0.5856036	0.04740602	0.30276786
STK10	550.878994	0.49129415	0.0473968	0.30276786
WDR70	505.760183	0.30506455	0.04735512	0.30276786
ZHX3	2889.82775	0.44074633	0.04740822	0.30276786
KCNJ1	1.9227854	0.91286626	0.04750044	0.30307892
MAPK8IP1	255.612486	-0.6632929	0.04749287	0.30307892
TRIM61	28.8714327	0.51759913	0.04752268	0.30310961
SURF2	70.7155327	-0.5171059	0.04755943	0.3032328
C16orf52	369.614216	0.32595714	0.04770311	0.30331054
CTNNBIP1	314.583598	0.51387594	0.04767756	0.30331054
KCTD15	433.055575	-0.5405209	0.04763388	0.30331054
PRKDC	8244.70853	-0.3268692	0.04770123	0.30331054
SCN2B	214.053607	0.93393556	0.04771118	0.30331054
TLR8	59.0842709	0.81838735	0.04761667	0.30331054
TNPO1	4825.76039	0.1972061	0.04770198	0.30331054
ZIM2	5.68833354	0.93308547	0.04764045	0.30331054
MRPL2	147.338194	-0.3713688	0.04774244	0.30334026
YRDC	71.4831759	0.48162034	0.04775075	0.30334026
SNORA37	16.933543	0.69139394	0.04778284	0.30343326
CD1D	19.2970154	0.7927279	0.04785619	0.30378811
GIMAP2	106.844767	0.61054064	0.04795187	0.30417333
IL6R	408.794325	0.67673822	0.04793608	0.30417333
ADPGK	686.831391	0.20413597	0.04797935	0.30419582
NSUN6	204.629373	0.3824202	0.0479904	0.30419582
SNRNP40	315.690534	0.38329332	0.04800869	0.30420084
ARG2	84.9546397	-0.8776643	0.04803216	0.30423867
FAM171B	1040.68248	-0.5770648	0.04807213	0.3043692

SEPHS2	427.28759	-0.5243923	0.04808778	0.3043692
ARMC4	17.2263311	0.90788017	0.04818573	0.30487822
ADRA1B	47.8037431	-0.7610955	0.04827205	0.30500707
KIF26B	39.7559684	0.83665612	0.0483013	0.30500707
LINC01355	183.701257	0.50311172	0.04829469	0.30500707
RBM15B	1090.19787	-0.3544174	0.04831135	0.30500707
TFIP11	298.42584	0.39494605	0.04824768	0.30500707
TXNL4B	135.781901	0.63822061	0.04823438	0.30500707
LOC286437	175.031258	0.36908347	0.04833319	0.30503419
CDH11	6758.83287	0.54681225	0.04840823	0.30528615
STXBP3	519.581978	0.42072713	0.04840435	0.30528615
NSFP1	3.32927051	-0.7865296	0.04846023	0.30539254
SORL1	3635.39636	0.80154822	0.0484478	0.30539254
STAG3L4	221.070652	-0.3820309	0.048486	0.30544421
TMEM186	89.1699913	-0.3715173	0.04851564	0.30552028
SP9	3.15825321	0.82645837	0.04853951	0.30555991
ZBTB5	462.398136	-0.3487669	0.04858045	0.30570695
TATDN3	199.236772	0.37381639	0.04868334	0.30624356
CTLA4	5.47144335	0.93514186	0.04880602	0.30644691
EHD2	2028.46351	-0.5644265	0.04878837	0.30644691
GSKIP	97.0750007	0.49543181	0.04882141	0.30644691
TIRAP	193.758405	-0.3128189	0.04874435	0.30644691
TMEM236	14.8728875	0.88269081	0.04878378	0.30644691
TNNT3	45.6100178	0.93354857	0.04879467	0.30644691
RCAN1	529.094012	0.4666429	0.04887168	0.30665174
OSCAR	32.6224729	0.7267398	0.04893682	0.3069497
GPC5	13.9935055	-0.9187894	0.04896355	0.3070066
CD2BP2	853.420835	0.25752319	0.0490294	0.30728173
HDAC5	819.52194	0.30800647	0.04905454	0.30728173
KRTCAP2	623.068545	-0.370615	0.04907238	0.30728173
SAP25	86.1624175	-0.5272342	0.04907813	0.30728173
TMED7	1839.22977	0.32979033	0.04911824	0.3074222
C9orf85	68.9065386	0.30403354	0.04916561	0.30760794
PKN3	123.782766	-0.6064484	0.04927569	0.30818574
HIST1H3D	191.583063	-0.4359402	0.04935623	0.30850867
LINC00174	192.661821	-0.4723448	0.04936281	0.30850867
AIG1	499.254886	0.38083614	0.04948433	0.30874822
ARHGAP11B	25.1563681	-0.7393604	0.04948992	0.30874822
ATXN2L	1440.30119	-0.309118	0.0494512	0.30874822
PCDHGA4	470.603934	-0.7420188	0.04947812	0.30874822
RAD51AP1	44.2764752	-0.5877783	0.04948085	0.30874822
EEFSEC	173.901142	-0.3964343	0.04957259	0.30915299
CLN3	283.514403	-0.2457046	0.0496046	0.30924172
MAGI2-AS3	1820.77999	0.37209514	0.04964695	0.3093948
ABCC11	3.62557008	-0.8466062	0.04978182	0.30946668
BRWD1-IT2	17.1921556	-0.6475554	0.04978799	0.30946668
GUSBP4	51.3890277	0.50292844	0.04978694	0.30946668
KLK11	3.90791312	-0.786536	0.04980087	0.30946668
MCM3AP-AS	86.5816529	-0.73842	0.04979771	0.30946668
PLK4	65.7785236	-0.6385111	0.04969875	0.30946668

TM4SF18	93.3771418	-0.7736156	0.04976876	0.30946668
ZNF670	57.0265316	0.32358979	0.04978974	0.30946668
C1orf198	1128.8129	0.59745659	0.04989361	0.30982148
VPS53	1086.03002	-0.292913	0.04988285	0.30982148
APOB	167.620887	0.78448258	0.04993701	0.30986966
CCDC96	10.0120555	-0.6674957	0.04992503	0.30986966
