# Supplementary material for: Transcriptome signatures associated with meningioma progression
Source: Acta Neuropathol Commun. 2019 Apr 30;7:67. doi: 10.1186/s40478-019-0690-x (PMC6489307; doi:10.1186/s40478-019-0690-x)
Supplement: Supplementary file 4 — Table S3. List of significantly differentially expressed genes between all grade I and all grade III meningiomas, as identified by RNA-seq. (PDF 659 kb) [file 40478_2019_690_MOESM4_ESM.pdf]

Supplementary Table 3: Differentially expressed genes between GR I and GR III meningiomas

Gene	baseMean	log2FoldChar	pvalue	padj
CERS4	189.750633	3.82520492	2.10E-27	3.61E-23
AGR2	1047.80481	-7.1030778	1.22E-23	1.05E-19
NTRK2	6092.66084	4.34632286	2.09E-22	1.19E-18
RWDD1	525.268835	1.03491578	5.21E-18	2.23E-14
CENPF	714.663326	-2.6977362	2.61E-16	8.94E-13
MKI67	1127.40949	-3.0623596	3.76E-16	1.08E-12
CCDC137	256.66806	-0.9360497	2.57E-15	4.91E-12
MYBL2	102.435386	-3.8388712	2.43E-15	4.91E-12
NUF2	41.543764	-2.3086201	2.10E-15	4.91E-12
LINC01234	75.7412903	-5.4370058	1.21E-14	2.08E-11
IRAK1BP1	171.019234	2.05899909	3.18E-14	4.96E-11
TUBB4A	23.823265	-5.0081004	3.62E-14	5.18E-11
SENP6	2275.60455	1.15983587	3.99E-14	5.27E-11
KIF18B	103.757594	-3.5519634	5.06E-14	5.79E-11
MED23	1021.44067	0.94660556	4.95E-14	5.79E-11
CACNG4	147.11187	-3.8040175	9.38E-14	1.01E-10
CCNC	479.556791	1.20162036	1.15E-13	1.10E-10
HJURP	72.1588247	-3.0512907	1.10E-13	1.10E-10
E2F7	53.7619306	-2.6450329	2.16E-13	1.95E-10
STX7	2033.10931	0.99686031	3.03E-13	2.60E-10
HECA	1515.01179	1.60687925	4.97E-13	4.06E-10
LMBRD1	963.1915	1.65860921	5.50E-13	4.29E-10
CDR2L	272.55473	-1.8286924	9.66E-13	6.91E-10
TMEFF2	23.3588381	-5.046366	9.39E-13	6.91E-10
RWDD2A	98.0920786	1.64381823	1.57E-12	1.08E-09
SAPCD2	41.6178141	-2.7394281	2.27E-12	1.50E-09
PPIL4	624.554496	0.92645531	3.32E-12	2.11E-09
IQSEC3	229.627434	3.67262457	4.64E-12	2.84E-09
PDCD2	490.05953	1.00149268	1.13E-11	6.66E-09
CIT	487.288493	-2.6807335	1.49E-11	8.54E-09
BUB1	131.950515	-2.4822577	1.60E-11	8.88E-09
PEG10	353.671951	-4.3886949	1.71E-11	9.19E-09
CENPA	19.7599718	-3.4358046	1.82E-11	9.48E-09
ATP2B2	114.786611	-4.5692897	2.10E-11	1.06E-08
IMMT	1503.02252	-0.9532513	2.86E-11	1.40E-08
TACC3	283.611634	-1.834947	3.04E-11	1.45E-08
BIRC5	119.561845	-2.9622592	3.18E-11	1.47E-08
C10orf32	474.112716	1.38677755	3.74E-11	1.69E-08
SNORA54	99.9043822	4.11548288	5.93E-11	2.61E-08
FAM229B	309.977049	1.81881691	6.41E-11	2.75E-08
KIF14	114.791741	-2.5220978	6.63E-11	2.78E-08
ASPM	449.665452	-2.9672818	7.13E-11	2.92E-08
ARMC2	129.952766	1.96362087	7.34E-11	2.93E-08
FAM111B	88.9714414	-2.6535305	9.01E-11	3.48E-08
HIST1H2AL	120.292698	-2.2821191	9.12E-11	3.48E-08
CKMT1B	15.6458903	-4.5303962	1.03E-10	3.76E-08
OSTM1	824.513337	1.16875634	1.02E-10	3.76E-08

HMMR	91.307773	-2.6855834	1.53E-10	5.49E-08
TMEM242	219.656883	1.01473736	2.05E-10	7.17E-08
ESPL1	114.103215	-2.420652	2.42E-10	8.32E-08
CEP162	321.979837	1.42379165	2.47E-10	8.32E-08
EEF1A1	89363.1313	1.17821103	2.61E-10	8.45E-08
MINOS1P1	207.166029	1.61242327	2.61E-10	8.45E-08
LRFN1	81.2631211	-1.8778725	3.22E-10	1.02E-07
SMAP1	573.905874	0.78812666	3.35E-10	1.04E-07
GABRQ	111.976089	-4.4980713	3.80E-10	1.16E-07
IQGAP3	250.352714	-2.8248868	4.60E-10	1.36E-07
PRR11	184.488683	-2.0964993	4.53E-10	1.36E-07
TRMT11	320.12743	1.18201884	5.07E-10	1.47E-07
TMEM50A	1042.81388	1.20056878	6.24E-10	1.79E-07
RNF146	951.039511	1.38992901	6.89E-10	1.94E-07
F5	248.549368	-4.4118258	7.42E-10	2.05E-07
NDUFB9	608.994862	-1.2034916	1.29E-09	3.50E-07
TONSL	158.342024	-2.0612642	1.34E-09	3.60E-07
GSG2	22.0888944	-2.7635739	1.52E-09	4.01E-07
SNX14	1049.71873	1.03751087	1.60E-09	4.17E-07
UFL1	1083.60999	0.97030313	1.72E-09	4.42E-07
DLEC1	129.905746	2.79816381	1.87E-09	4.72E-07
MAP3K7	1195.30523	0.86079742	2.14E-09	5.32E-07
HOXB3	68.4336951	-4.1131355	2.34E-09	5.74E-07
FOXM1	202.228809	-2.9108887	2.39E-09	5.78E-07
CHCHD10	166.117878	-2.5285321	2.92E-09	6.96E-07
TMEM30A	3358.50437	1.30016151	3.26E-09	7.66E-07
TROAP	71.8199488	-2.9113128	3.33E-09	7.72E-07
CEP55	90.1769735	-2.6270636	3.49E-09	7.98E-07
TSPYL1	2564.20771	1.30788003	3.85E-09	8.69E-07
KCNH1	86.6983315	-3.8808765	4.33E-09	9.66E-07
CDCA8	34.1756386	-2.0902646	5.12E-09	1.13E-06
HBS1L	873.13494	0.94413537	5.24E-09	1.14E-06
RARS2	509.418643	0.78608016	5.50E-09	1.18E-06
TOP2A	902.638104	-2.4083093	5.92E-09	1.25E-06
CASC1	35.0090539	2.43905227	6.21E-09	1.30E-06
HOXC10	23.4512162	-4.3368958	6.95E-09	1.44E-06
PRKAA2	811.966344	1.62076614	7.28E-09	1.47E-06
TK1	172.999065	-2.8183614	7.20E-09	1.47E-06
FBXL4	625.909964	1.123498	8.75E-09	1.75E-06
SYNGR3	42.4740037	-3.8579564	9.61E-09	1.90E-06
MYLK	1972.61456	3.19820971	1.10E-08	2.12E-06
PPP1R14B	635.51348	-1.4777182	1.09E-08	2.12E-06
PNISR	2988.31296	0.9834288	1.16E-08	2.20E-06
CDC20	44.573848	-2.5394847	1.22E-08	2.30E-06
LINC01359	28.0996429	2.11126651	1.39E-08	2.59E-06
IBTK	1723.7439	0.81588295	1.49E-08	2.75E-06
MRPS5	604.264857	-0.9785329	1.72E-08	3.10E-06
SHOX2	136.01592	-4.1206183	1.71E-08	3.10E-06
POLI	529.02087	1.21476702	2.10E-08	3.72E-06
SYNJ2	2025.17572	1.70160238	2.09E-08	3.72E-06

DPP6	133.610762	-3.980776	2.39E-08	4.19E-06
EXO1	51.4603561	-2.6820335	2.47E-08	4.28E-06
THUMPD3	793.292772	-0.8773761	2.57E-08	4.41E-06
ATP5I	803.66628	-0.949383	2.70E-08	4.53E-06
CDT1	65.1661409	-2.6788366	2.68E-08	4.53E-06
KIF11	260.424961	-2.2202487	2.72E-08	4.53E-06
XKR5	17.8512704	-3.246982	2.96E-08	4.88E-06
COQ9	827.883568	-1.4706152	3.06E-08	4.95E-06
FAM57B	3.39488512	-4.0498853	3.06E-08	4.95E-06
GOT1	655.837205	-1.6304576	3.09E-08	4.95E-06
PAQR4	90.5834405	-1.8004099	3.12E-08	4.96E-06
STXBP5	715.880661	1.59964303	3.16E-08	4.97E-06
PNRC1	3340.87371	1.43951585	3.26E-08	5.09E-06
ZNF124	397.81158	1.35897298	3.31E-08	5.13E-06
CDCA5	74.7821707	-2.3608108	3.57E-08	5.46E-06
WDR62	72.2141462	-2.3029174	3.59E-08	5.46E-06
UBQLN4	505.627989	-0.9301327	3.64E-08	5.48E-06
FSCB	37.2343189	-3.790303	3.82E-08	5.70E-06
GLIPR1	849.674851	0.93181839	4.03E-08	5.96E-06
PEX3	241.821839	1.00227692	4.14E-08	6.07E-06
COL25A1	40.3888042	2.38123502	4.20E-08	6.11E-06
NDUFA8	367.059232	-0.9911868	4.46E-08	6.43E-06
KIF4A	82.976849	-2.534331	4.81E-08	6.83E-06
ZIC3	16.3489514	-3.8731113	4.80E-08	6.83E-06
GABRA3	120.466834	-3.8168392	5.52E-08	7.76E-06
UBL4B	116.475158	3.52115403	5.59E-08	7.80E-06
SERINC1	5949.9413	1.14944866	5.64E-08	7.80E-06
SYT10	5.84080507	-3.7867546	5.85E-08	8.01E-06
TRIM47	241.965111	-1.8068925	5.88E-08	8.01E-06
DLGAP5	44.6291107	-2.3533645	6.66E-08	9.00E-06
PKMYT1	40.3872776	-2.5672189	7.31E-08	9.80E-06
PCMT1	600.531562	1.02816571	7.74E-08	1.02E-05
TECTA	85.1945482	1.12246093	7.69E-08	1.02E-05
HIST1H2BO	126.20248	-2.2304391	8.61E-08	1.13E-05
KIF20A	109.216911	-2.3313709	9.47E-08	1.23E-05
SV2A	200.504474	-3.081204	9.83E-08	1.27E-05
CLK1	2836.67005	0.97765787	1.01E-07	1.29E-05
SRSF12	101.959565	1.95115424	1.09E-07	1.38E-05
DLAT	761.106901	-0.9043226	1.11E-07	1.40E-05
KPNA5	310.236329	1.38907312	1.30E-07	1.62E-05
MAP3K4	864.768898	1.31041174	1.37E-07	1.71E-05
NCAM2	42.7464225	-3.1020292	1.43E-07	1.74E-05
NOMO2	353.113083	-1.1799335	1.41E-07	1.74E-05
SYF2	451.260599	0.97816243	1.43E-07	1.74E-05
AURKB	32.5612541	-2.3851695	1.50E-07	1.81E-05
MEX3A	51.0328421	-2.5068488	1.69E-07	2.02E-05
FBN3	258.521007	-3.5379921	1.72E-07	2.05E-05
MRPL41	396.434536	-1.0064159	1.84E-07	2.17E-05
CKAP2L	74.2150284	-2.4456053	1.88E-07	2.21E-05
NEK2	35.5527468	-2.404616	2.00E-07	2.34E-05

DSE	2862.39982	1.45822681	2.09E-07	2.41E-05
RRM2	217.035993	-2.6770957	2.09E-07	2.41E-05
ZMIZ1	4635.11992	-1.207117	2.10E-07	2.41E-05
ARID5B	1789.43922	2.16231502	2.22E-07	2.51E-05
SPAG5	224.581724	-1.8284126	2.23E-07	2.51E-05
SHC1	3203.09103	-0.7305704	2.27E-07	2.55E-05
FAM83D	36.2923202	-2.6808958	2.31E-07	2.58E-05
DIAPH3	125.033919	-2.0553154	2.48E-07	2.75E-05
CMKLR1	1379.81861	-2.6365796	2.64E-07	2.90E-05
LINC00087	59.3621938	1.6989002	2.80E-07	3.06E-05
C7orf13	72.6146521	-2.1022016	2.87E-07	3.11E-05
FAM120B	1242.42581	0.79923394	2.88E-07	3.11E-05
LRFN4	293.638075	-2.3071119	2.90E-07	3.11E-05
SYT1	35.6706259	-3.1380237	2.99E-07	3.19E-05
SMOC1	3404.74899	3.21154772	3.02E-07	3.19E-05
MAN1C1	2645.23056	1.39963289	3.12E-07	3.29E-05
TBX20	7.18106339	-3.7659902	3.21E-07	3.36E-05
KCNK5	143.353602	2.55543763	3.58E-07	3.72E-05
DENND5B	601.00255	1.29475829	3.75E-07	3.88E-05
SPC25	23.8396586	-2.8714312	3.82E-07	3.93E-05
N4BP2L2-IT2	853.833093	0.9176217	3.95E-07	4.04E-05
UPK3B	189.546719	-3.0463521	4.00E-07	4.06E-05
KPNA2	656.648351	-1.5422775	4.17E-07	4.21E-05
EME1	44.7365651	-2.1426622	4.22E-07	4.24E-05
CASC5	247.03552	-2.0254371	4.47E-07	4.46E-05
CXADRP2	17.5506404	-3.7138487	4.52E-07	4.49E-05
ALG3	321.798351	-1.1720291	4.69E-07	4.63E-05
NUSAP1	298.705166	-2.1782406	4.85E-07	4.76E-05
SGOL1	40.9064776	-2.4095751	4.93E-07	4.80E-05
E2F8	20.9454261	-2.8663528	5.12E-07	4.96E-05
HOXD13	12.8682832	-3.7751459	5.22E-07	5.03E-05
BUB1B	140.20289	-2.3828323	5.29E-07	5.07E-05
MELK	112.466703	-2.6113249	5.61E-07	5.35E-05
DKFZp434J02	15.0694913	-3.5314399	5.75E-07	5.45E-05
HIST2H3D	59.2919232	-1.9794402	6.12E-07	5.77E-05
CCDC28A	242.893215	1.35011479	6.46E-07	6.06E-05
UST	968.089564	1.78410291	6.68E-07	6.23E-05
TRIM37	1092.7114	-0.9253673	6.82E-07	6.32E-05
ARHGEF39	54.7582187	-2.4624286	6.97E-07	6.37E-05
OGFRL1	1498.99694	1.42678533	7.01E-07	6.37E-05
SF3B5	466.577844	0.86716837	7.01E-07	6.37E-05
WHSC1	1694.57389	-1.1476005	6.93E-07	6.37E-05
PCGF2	626.940149	-1.0135808	7.35E-07	6.60E-05
TULP4	1560.53747	0.64693533	7.34E-07	6.60E-05
KCNMA1	15014.0204	3.09717854	7.50E-07	6.71E-05
SRCIN1	61.5705554	-2.3679635	7.62E-07	6.78E-05
GUSBP4	55.9270719	1.45150422	7.74E-07	6.81E-05
RHBG	13.006298	-3.7072445	7.73E-07	6.81E-05
SAYSD1	160.179653	1.30782265	7.82E-07	6.85E-05
C19orf54	166.11146	-1.2555705	7.91E-07	6.89E-05

DDX60	940.119993	1.1440712	9.19E-07	7.93E-05
DOPEY1	1232.26857	1.34239806	9.17E-07	7.93E-05
PEX7	85.5184372	1.4101819	9.33E-07	8.01E-05
COG8	430.895931	-0.7068139	9.41E-07	8.04E-05
SIDT1	139.704396	-2.5636287	9.53E-07	8.10E-05
MRPL12	337.812754	-1.7881733	9.77E-07	8.26E-05
IGF2BP1	231.64847	-3.6818096	1.07E-06	8.99E-05
LATS1	1542.64653	0.8732865	1.08E-06	9.04E-05
RECQL4	108.950376	-2.0126349	1.09E-06	9.07E-05
SLC31A2	368.157749	1.70963175	1.13E-06	9.39E-05
NCAPH	67.7986317	-2.1640402	1.22E-06	0.00010053
ELF3	218.822004	-3.3871162	1.26E-06	0.00010312
CYB5R4	201.466521	1.12284394	1.30E-06	0.00010659
FASN	3312.88578	-1.6861714	1.36E-06	0.00011067
MUC15	40.9082842	-3.6405306	1.40E-06	0.00011256
RPS13	2999.5097	0.64873646	1.40E-06	0.00011256
ZNF292	2247.5179	0.74403458	1.40E-06	0.00011256
WTAP	1186.35796	0.90318237	1.43E-06	0.0001139
GPX3	1033.51045	2.62874958	1.46E-06	0.00011483
GTF2H5	373.740459	1.13843488	1.45E-06	0.00011483
IFIT3	650.176732	0.89833254	1.47E-06	0.00011483
RPS18P9	68.5748586	1.57875738	1.46E-06	0.00011483
HRCT1	92.0000258	-2.0806229	1.54E-06	0.00011924
HSF2	304.913977	1.16485131	1.54E-06	0.00011924
ARMCX1	514.528882	0.65976058	1.55E-06	0.00011982
BMPR1B	3902.22607	2.69579407	1.59E-06	0.00012128
UBE2T	69.1767508	-1.923823	1.59E-06	0.00012128
USP45	321.415377	1.0217161	1.59E-06	0.00012128
ATP8B3	177.059151	-2.2059594	1.62E-06	0.00012336
SESN1	2046.58208	1.87055264	1.70E-06	0.00012824
CPO	11.4286543	2.80120058	1.74E-06	0.00013089
HACE1	337.322223	1.23345601	1.77E-06	0.00013225
LOC1005066	45.340747	1.62257354	1.77E-06	0.00013225
PRAC2	4.75975228	-3.5927256	1.81E-06	0.00013451
TRAP1	1033.62389	-1.1326422	1.82E-06	0.00013452
CKS2	92.6602683	-1.9800654	1.85E-06	0.00013623
CDKN3	34.8028122	-2.1318236	1.93E-06	0.00014161
GINM1	649.942712	0.75587965	1.94E-06	0.00014202
LOC1002890	208.493174	-1.8477811	2.03E-06	0.00014764
CD164	4757.87276	0.95740065	2.06E-06	0.00014895
EZH1	1237.76466	1.02049272	2.11E-06	0.00015132
RHOD	292.059907	-1.1563768	2.10E-06	0.00015132
MMP15	659.516792	-2.7765751	2.16E-06	0.00015439
OTX1	6.95966886	-3.1296096	2.19E-06	0.00015588
FIG4	320.469056	1.11772505	2.21E-06	0.00015678
DPH6-AS1	22.5921435	2.14756982	2.33E-06	0.00016362
TYRP1	6.32465598	-3.5205894	2.32E-06	0.00016362
HAND2	13.147496	-3.5602485	2.37E-06	0.00016619
MORN2	103.625866	1.30844082	2.49E-06	0.00017361
GIT1	1478.98271	-1.1883845	2.50E-06	0.00017385

NUP210	1016.78538	-1.549609	2.55E-06	0.00017385
PDE1C	193.370745	3.12860655	2.54E-06	0.00017385
PDLIM5	3899.07628	1.26019789	2.55E-06	0.00017385
PHLDB1	3073.50344	-0.8109664	2.53E-06	0.00017385
RNF11	1993.70043	1.1625444	2.55E-06	0.00017385
BZW2	246.811942	-1.0824528	2.57E-06	0.00017457
GPC3	284.049285	-3.0820848	2.59E-06	0.00017515
CPQ	1836.40044	1.21075513	2.66E-06	0.00017912
CACNB1	176.123656	-1.469067	2.68E-06	0.0001794
PSAT1	303.119766	-2.0785142	2.69E-06	0.00017964
RSRP1	841.707998	1.36554089	2.76E-06	0.00018367
LRRC20	212.866114	-1.4888188	2.78E-06	0.00018423
ZNF676	157.230263	3.33125122	2.81E-06	0.00018539
ATP5B	6873.97422	-0.9266386	2.94E-06	0.00019344
ADCY5	3813.43826	-1.4091768	2.97E-06	0.00019426
MTNR1A	9.6365119	-3.4038739	3.04E-06	0.0001983
LOC729603	194.967892	1.68542351	3.12E-06	0.00020262
CPE	13020.586	3.14882534	3.18E-06	0.00020627
CCDC62	10.8197653	2.26256578	3.41E-06	0.00021979
DTL	169.202332	-2.2644001	3.50E-06	0.00022482
HOXB4	24.3954249	-3.1470726	3.58E-06	0.00022956
MAG	23.1706354	-3.4365661	3.62E-06	0.00023087
WDR96	35.8050312	2.52918824	3.67E-06	0.00023321
MTRNR2L9	60.2387764	1.84483019	3.80E-06	0.00024038
HIST1H2AI	142.773791	-2.0307412	3.86E-06	0.00024381
CCNA2	103.991996	-1.8858171	3.91E-06	0.0002456
DCPS	166.54925	-0.7676433	3.92E-06	0.0002456
SLC25A5	1890.76588	-1.0363742	4.03E-06	0.00025173
E2F2	33.2583075	-1.9578989	4.31E-06	0.0002681
HCFC1R1	785.10632	-1.1530419	4.38E-06	0.00027116
LETM1	1216.06092	-0.7262154	4.40E-06	0.00027198
CTGF	16772.0814	2.63587531	4.44E-06	0.00027337
MSANTD4	553.30069	-0.779774	4.55E-06	0.00027918
AIG1	542.882633	1.13713222	4.60E-06	0.00028103
LOC1001305	22.3392687	-3.2959837	4.65E-06	0.00028297
RGS9BP	8.0100499	-2.7879327	4.67E-06	0.00028315
CREBL2	1380.98212	1.02451338	4.72E-06	0.00028516
POLR3K	108.155596	-1.0835682	4.86E-06	0.00029257
CDCA7	86.2404134	-2.8756172	4.91E-06	0.00029277
NCBP2-AS2	158.504754	-0.7892614	4.90E-06	0.00029277
OLIG1	9.35801703	-3.1913325	4.90E-06	0.00029277
ZUFSP	147.308907	0.88104873	4.95E-06	0.00029376
CHCHD3	674.561202	-0.796445	4.98E-06	0.00029378
TEX14	49.3032691	-2.9735908	4.97E-06	0.00029378
HIST1H3B	311.842893	-2.1076488	5.12E-06	0.00030115
KIAA0101	71.2455429	-2.5211944	5.28E-06	0.00030955
H2AFX	355.751863	-1.6668248	5.43E-06	0.00031712
REV3L	1794.60545	0.96376713	5.53E-06	0.00032151
PDHA1	974.865193	-0.9221397	5.65E-06	0.00032653
SLC6A17	45.7597071	3.06501388	5.65E-06	0.00032653

AR	1106.58485	3.10747518	5.80E-06	0.00033417
DHRS2	187.740547	-3.090836	5.88E-06	0.0003378
COL20A1	6.13872003	-3.3230554	5.95E-06	0.00033816
GVINP1	201.483536	1.54424025	5.95E-06	0.00033816
PDPK1	1476.20875	-0.5173099	5.95E-06	0.00033816
CCNB3	12.4387654	-1.8574961	5.98E-06	0.0003389
EPG5	2043.04764	1.20502699	6.15E-06	0.0003471
LMNB1	242.135009	-1.6055719	6.18E-06	0.00034807
GTF2E1	224.362145	-0.6738294	6.29E-06	0.00035263
PGP	351.04566	-0.7723226	6.55E-06	0.00036642
BCAR3	401.105924	2.60460343	6.97E-06	0.00038711
ESRRG	13.6583075	-2.8620839	6.97E-06	0.00038711
PDSS2	325.406244	0.95029141	7.03E-06	0.00038927
CDK15	10.0351473	2.51851191	7.30E-06	0.00040287
CGN	33.3921441	-2.1660079	7.41E-06	0.00040754
CRAT	861.166467	-0.7910012	7.50E-06	0.00041141
MANEA	506.14749	1.48727277	7.64E-06	0.00041741
ASIP	11.4341827	3.26366927	7.76E-06	0.00042148
CHRM1	113.222887	-3.2345914	7.76E-06	0.00042148
DLL1	121.633819	2.68210564	7.85E-06	0.0004249
PRRC2C	7025.77414	-0.6559472	7.98E-06	0.00043061
FAM189B	484.130739	-1.5377963	8.20E-06	0.00043904
HIST1H2AJ	101.506483	-2.1617782	8.21E-06	0.00043904
IL33	70.203025	2.62250106	8.19E-06	0.00043904
MATK	63.5777062	-2.4538325	8.38E-06	0.00044653
ALAS2	13.5767688	2.7860484	8.47E-06	0.00045003
UBE2C	104.118917	-2.5357729	8.63E-06	0.00045719
C16orf59	20.2374665	-2.2973256	8.71E-06	0.00046009
APOL1	751.789721	1.48706428	8.82E-06	0.00046462
LRPPRC	2895.99261	-0.7494965	8.95E-06	0.00046995
SNX9	1245.64034	0.89452774	8.98E-06	0.00046995
ADAMTS8	19.9188237	-3.203176	9.24E-06	0.00048192
CHEK1	110.153241	-1.6029651	9.45E-06	0.00049148
SDCCAG3	406.040437	-0.7409432	9.62E-06	0.00049901
CYP2G1P	7.16891677	2.59112549	9.71E-06	0.00050178
AHCYL2	7694.72543	1.30660841	9.82E-06	0.00050618
POLQ	99.7733178	-2.1154062	9.97E-06	0.00051254
PQLC3	423.913135	0.95548875	1.02E-05	0.00052038
CDCA3	34.7083687	-1.9643426	1.11E-05	0.00056783
GDA	23.9999149	-3.0768595	1.11E-05	0.00056783
SRL	35.2209087	2.9227017	1.12E-05	0.0005706
EIF4EBP1	224.138678	-1.3301138	1.13E-05	0.0005714
LY6H	16.6810797	-2.8482722	1.17E-05	0.00059105
NPLOC4	1816.43533	-0.745685	1.17E-05	0.00059105
OAZ1	3442.91957	0.66645915	1.19E-05	0.00059891
SCAF8	1929.21957	0.5795074	1.21E-05	0.00060398
CRMP1	447.584463	-2.3791852	1.22E-05	0.00060806
SYNPO2	2996.14692	2.50015839	1.23E-05	0.00061126
INCENP	304.487873	-0.7607108	1.26E-05	0.00062455
CYR61	1431.06778	1.96888846	1.27E-05	0.00063004

LOC1019276	13.5648244	-3.0476393	1.32E-05	0.00064939
FOXK2	979.870481	-0.8644701	1.33E-05	0.00065212
LOC90784	341.606191	-0.9712168	1.33E-05	0.00065212
COX8A	1239.57547	-0.6852824	1.37E-05	0.00066762
HIST1H1B	457.697536	-1.777744	1.37E-05	0.00066762
TICRR	84.9926078	-2.5934655	1.37E-05	0.00066762
LOC1005073	214.578961	-2.6619119	1.39E-05	0.00067407
MAP7	200.469916	1.88400214	1.40E-05	0.00067548
GOT2	1220.95143	-0.8612187	1.41E-05	0.00068064
RGL1	1700.02139	1.69521101	1.43E-05	0.000688
DARS2	437.030174	-1.0971507	1.45E-05	0.00069594
ADCY10P1	104.099812	2.38791264	1.46E-05	0.00069637
FAM69B	325.351957	-1.0249865	1.50E-05	0.00071135
LCA5	130.012521	1.61286941	1.50E-05	0.00071135
TAF1A-AS1	18.301868	-1.6645139	1.50E-05	0.00071135
ZNF99	78.8006945	2.92572968	1.50E-05	0.00071135
ADAM20	118.677473	1.09904336	1.57E-05	0.00074006
FILIP1	160.414314	2.35938347	1.58E-05	0.0007451
ZWINT	119.326931	-1.8242616	1.59E-05	0.00074545
HIST1H2BL	100.253881	-1.6521021	1.60E-05	0.00074683
NCAPG	144.15022	-2.115952	1.60E-05	0.00074683
DVL3	1694.75406	-0.6859586	1.63E-05	0.00075971
LINC00858	7.05622068	-3.2433372	1.73E-05	0.00079901
MYLK4	533.863279	2.75172964	1.73E-05	0.00079901
LPCAT4	194.575983	-1.4893779	1.79E-05	0.00082499
PHGDH	477.027866	-2.5225292	1.81E-05	0.00083306
FOXO3	3020.39724	1.60161576	1.82E-05	0.00083587
EPM2A	133.929413	1.04187552	1.87E-05	0.00085166
FOXC2	1002.97953	-1.7341882	1.86E-05	0.00085166
PARK2	108.596854	1.53861881	1.87E-05	0.00085267
RNGTT	496.71505	1.02728337	1.88E-05	0.00085408
TIGD5	124.480378	-0.9612661	1.89E-05	0.00085408
DNAH12	64.806084	2.49831565	1.93E-05	0.00087197
ZFYVE9	1317.75229	1.07302254	1.94E-05	0.00087384
ACACB	1254.78765	-0.8444689	2.00E-05	0.00089651
OAS2	348.1129	0.99955702	2.00E-05	0.00089651
RHOBTB3	1237.32268	2.62526322	2.01E-05	0.00089651
PSKH1	824.397151	-0.8603921	2.04E-05	0.00090874
ANLN	300.15081	-2.0464932	2.08E-05	0.00092476
ZNF214	73.5851041	1.88845473	2.08E-05	0.00092476
MCM10	47.5591747	-2.3142874	2.10E-05	0.00092692
FANCC	308.14393	-1.3199883	2.13E-05	0.00093787
TMEM181	864.523989	0.88757839	2.14E-05	0.00094295
CDC6	106.441338	-1.8494201	2.16E-05	0.00094551
PM20D1	10.5748545	2.8592376	2.16E-05	0.00094551
ZNF136	375.574507	1.04820284	2.15E-05	0.00094551
CCNF	106.408207	-1.7517829	2.20E-05	0.00095787
BLM	74.4257353	-2.0574241	2.22E-05	0.00095904
LINC00271	6.46810286	2.98145265	2.22E-05	0.00095904
R3HDM1	659.467861	-0.6949552	2.21E-05	0.00095904

CPXM2	2020.51216	2.73491811	2.25E-05	0.0009688
LRCH1	724.257118	1.84201167	2.26E-05	0.00097044
LOC1002881	35.2557841	-2.771954	2.27E-05	0.00097209
PPIL6	31.2187078	2.14942118	2.27E-05	0.00097341
PM20D2	380.474503	1.57587537	2.29E-05	0.00097647
ARRDC1	339.99334	-0.7200804	2.31E-05	0.00098405
TIAM2	37.9355733	2.04656026	2.32E-05	0.00098424
INTU	401.456976	1.37403505	2.33E-05	0.00098556
HMBS	157.98461	-1.4808698	2.33E-05	0.00098633
RPS16P5	172.144782	1.47395843	2.36E-05	0.00099619
ARHGEF3	1065.43332	1.88962003	2.39E-05	0.00099998
FANCA	162.750654	-1.8305793	2.39E-05	0.00099998
MRPS34	423.988861	-0.7786843	2.39E-05	0.00099998
ORC3	539.902136	0.63566001	2.39E-05	0.00099998
CENPQ	91.2372306	0.90606443	2.42E-05	0.00100935
FABP7	4.34177352	-3.1147125	2.43E-05	0.00101181
MYPOP	66.6268479	-1.0326046	2.45E-05	0.0010174
PLD3	6535.51423	1.35159055	2.47E-05	0.00102215
DCTPP1	217.247848	-0.9253946	2.48E-05	0.00102305
RAB3A	51.7026733	-1.3824253	2.49E-05	0.00102305
RGS2	527.56758	1.97215115	2.49E-05	0.00102397
ASF1B	63.4340203	-1.7656742	2.51E-05	0.00102733
ADPRM	84.797888	1.19694101	2.52E-05	0.00102915
LOC1009965	12.0025991	-3.1344714	2.54E-05	0.00103427
LLGL2	432.374843	-1.9690518	2.66E-05	0.0010806
GPSM3	4.85810158	-3.1119083	2.67E-05	0.00108172
TPCN2	422.544664	-0.8625859	2.71E-05	0.00109791
ESCO2	37.6594801	-1.8303369	2.73E-05	0.00110418
COX7B	981.520009	-1.0857837	2.80E-05	0.00112044
MN1	1614.00488	1.79551486	2.80E-05	0.00112044
PRRX1	3056.59019	2.50827007	2.79E-05	0.00112044
UNC13A	578.676722	-2.6590537	2.80E-05	0.00112044
ESRRA	431.007054	-1.0450882	2.87E-05	0.00114259
TRIM46	85.602036	-2.4178418	2.87E-05	0.00114259
FRAT1	147.508673	1.70734265	2.89E-05	0.00114899
CXorf22	33.0657129	-2.9705374	2.94E-05	0.00116111
SNAP23	1007.97928	0.57496786	2.93E-05	0.00116111
FAM135A	639.913018	0.96814144	2.96E-05	0.00116687
NCAN	20.7571318	-3.0671839	2.98E-05	0.00117389
TBC1D32	363.429979	1.2383956	3.00E-05	0.00117646
TCEAL2	90.4612237	2.85929252	3.03E-05	0.00118908
SMAD4	2526.59397	0.8137513	3.07E-05	0.00119948
SEPT5	12.3980255	-1.9701256	3.10E-05	0.00120817
RMI1	118.182424	-1.0254222	3.23E-05	0.00125805
OLFM1	186.833758	-2.3742273	3.27E-05	0.00127094
DCX	47.5716476	-2.8981894	3.30E-05	0.00127816
CAMSAP1	1095.01468	-0.616789	3.31E-05	0.00128049
LOC1005056	10.6383038	-1.8162965	3.33E-05	0.00128468
RSPH3	229.275561	0.7484524	3.41E-05	0.00131116
TEKT3	14.8264595	2.38865585	3.41E-05	0.00131116

MTO1	352.282619	0.79764995	3.45E-05	0.00132094
TMC6	918.146787	-1.3272139	3.46E-05	0.00132094
CLK4	530.763903	1.0127948	3.48E-05	0.00132931
EBP	224.792986	-1.2237917	3.50E-05	0.00133273
CDK1	153.144048	-1.9620813	3.56E-05	0.00135042
KIAA1919	442.62422	0.95328511	3.58E-05	0.00135343
NDUFS1	1828.65311	-0.7006914	3.58E-05	0.00135343
HUNK	65.8482424	-2.0799651	3.62E-05	0.0013659
STL	48.9626979	1.57368863	3.74E-05	0.00140595
LHX2	10.782016	-3.008705	3.76E-05	0.0014141
DDX26B	423.4942	1.42629718	3.81E-05	0.00142955
MAP3K5	869.149961	1.7070595	3.83E-05	0.00143102
ISL2	9.01970494	-3.0926222	3.90E-05	0.00145241
LRRC58	1841.52787	-0.5371554	3.90E-05	0.00145241
SQLE	611.483362	-1.8119246	3.93E-05	0.00145981
ACSL5	295.415922	1.55494832	3.94E-05	0.00146053
FST	54.5418543	2.86839402	4.01E-05	0.00148194
KIF13B	938.948019	1.15184502	4.05E-05	0.00149556
ANO6	6760.96571	0.96587104	4.12E-05	0.00151633
TERC	797.383563	-1.4043536	4.13E-05	0.00151861
NPIPB3	356.448919	-1.4835018	4.17E-05	0.0015278
ASF1A	313.925085	0.89800273	4.20E-05	0.00153527
NOB1	535.664427	-0.904292	4.20E-05	0.00153527
CNOT6L	1224.10583	0.67219862	4.28E-05	0.0015512
LYST	2011.07914	1.11055987	4.27E-05	0.0015512
NELL2	65.1681531	-2.1766015	4.26E-05	0.0015512
TSIX	25.197287	2.7055309	4.28E-05	0.0015512
ALDH3A2	4080.80388	1.6245864	4.31E-05	0.00155512
CENPU	102.956962	-1.7323383	4.32E-05	0.00155512
PSMG2	493.222189	0.78093027	4.31E-05	0.00155512
IFIT2	430.17998	1.05287935	4.37E-05	0.00157035
CAMTA1	368.046412	1.08748127	4.45E-05	0.00159451
LOC145474	137.476354	1.64852921	4.46E-05	0.00159571
L3MBTL1	304.41966	1.04517634	4.53E-05	0.00161688
CCL26	115.087392	1.94555587	4.58E-05	0.00163046
LBX1	2.96910094	-3.0755482	4.59E-05	0.00163046
CTSC	1993.99921	-1.8738567	4.68E-05	0.00165932
TEAD2	358.937121	-1.3577703	4.84E-05	0.00171302
EZH2	195.63929	-1.504868	4.86E-05	0.00171478
MORN1	69.5911079	1.34785303	4.89E-05	0.00172325
ZNF777	273.773685	-0.7430855	4.90E-05	0.00172485
LINC00630	123.174527	0.65815035	4.94E-05	0.00173558
MRPL18	341.708565	0.75114873	4.96E-05	0.00173692
BOLA3	103.51029	-1.0505564	5.01E-05	0.0017516
C9orf172	16.0726124	-2.4576165	5.05E-05	0.00175463
COX5A	643.766982	-1.5607778	5.04E-05	0.00175463
LOC1005075	104.422068	1.86075064	5.06E-05	0.00175463
XAB2	654.597657	0.58242336	5.04E-05	0.00175463
DNM1	84.5012565	-1.6228364	5.11E-05	0.00176785
ACLY	2798.0069	-0.7162406	5.14E-05	0.00177566

CYC1	742.702446	-0.9145154	5.21E-05	0.00179287
THAP8	52.8262099	-1.2521619	5.21E-05	0.00179287
SOX9	150.447586	-2.8958472	5.32E-05	0.00182535
CTDSP1	2597.39607	-0.3669105	5.44E-05	0.00186401
CDH6	343.727496	2.32776878	5.47E-05	0.00186744
PRMT9	380.541758	1.07699715	5.46E-05	0.00186744
REPS1	686.247849	0.90285413	5.49E-05	0.00186923
PPP2R3C	217.44895	0.94103282	5.57E-05	0.00189104
WNT9A	143.088226	-1.2963317	5.57E-05	0.00189104
AUNIP	6.12551054	-2.0723158	5.72E-05	0.00193244
RAB23	1004.48589	1.45922249	5.72E-05	0.00193244
SLC31A1	813.319715	-0.8267849	5.75E-05	0.00194033
OPA1	1763.08803	-0.3522978	5.78E-05	0.00194097
RAP1GAP2	511.234059	-1.6677629	5.77E-05	0.00194097
KATNA1	165.076917	0.79991619	5.85E-05	0.001961
B9D2	34.9184025	1.43919362	5.98E-05	0.00199465
GATA3	16.1290326	-2.2381928	5.96E-05	0.00199465
LOC1019277(	28.7519384	-3.0271534	5.99E-05	0.00199465
MRPL38	468.268488	-0.672859	6.01E-05	0.00199465
THAP4	584.01637	-0.8231227	6.00E-05	0.00199465
ERMARD	293.83184	0.84033897	6.07E-05	0.00201168
ITIH2	2115.32064	2.62648354	6.08E-05	0.00201236
TNFSF4	63.6216652	1.80300737	6.23E-05	0.00205551
KLHL41	25.3285332	1.78578388	6.25E-05	0.00205818
APOL6	1071.24896	0.91316967	6.54E-05	0.00215189
DHCR7	536.223012	-1.6560317	6.62E-05	0.00217143
FRRS1L	51.1771502	2.71728022	6.65E-05	0.0021774
RNF217	731.17544	0.92137958	6.66E-05	0.00217804
ZNF285	99.8641501	1.11782438	6.69E-05	0.0021837
CCDC106	248.70774	-0.939799	6.73E-05	0.00218845
RRP7B	146.409736	1.04286337	6.73E-05	0.00218845
MDH2	1634.51204	-0.9231303	6.75E-05	0.00218921
NUP43	829.364562	0.70612278	6.80E-05	0.00220324
GAS7	1129.53846	-1.6802774	6.83E-05	0.00220946
GALR1	10.8997174	-2.9954237	6.86E-05	0.00221083
HCN2	55.0892803	-2.0124318	6.88E-05	0.00221083
LOC1005061	12.1621182	2.41892826	6.89E-05	0.00221083
NKX6-1	21.9903555	-2.7142192	6.90E-05	0.00221083
TMEM223	142.583002	-0.8190842	6.90E-05	0.00221083
MPPE1	463.505729	0.7720622	6.96E-05	0.00222379
CKS1B	116.256286	-1.2646658	6.99E-05	0.00222895
PCSK1N	351.45395	-2.1413328	7.00E-05	0.00222928
ENO4	17.4809736	2.00727376	7.05E-05	0.0022413
HIST1H2BK	805.92466	-0.7523827	7.09E-05	0.00224826
ZMYND19	170.795132	-0.9151931	7.10E-05	0.00224826
URB2	413.649764	-0.794511	7.17E-05	0.00226498
TDRKH	152.098109	-1.2795479	7.37E-05	0.00232477
KLHL31	35.9349537	1.88090853	7.41E-05	0.00232971
POTEC	4.65710768	-2.9819395	7.40E-05	0.00232971
AFMID	288.580292	-1.1651723	7.46E-05	0.00234165

SERAC1	237.759241	1.34781828	7.50E-05	0.00234932
CENPE	185.969002	-1.7114243	7.60E-05	0.00237544
BRCA2	299.655358	-1.5531872	7.62E-05	0.00237749
SMIM8	158.99756	1.16118354	7.64E-05	0.00238084
FAM222A	21.9603379	-1.8034848	7.66E-05	0.00238217
ISPD	81.2811233	1.28513183	7.71E-05	0.00239374
LINC00667	576.972372	0.74147016	7.76E-05	0.00240019
SYTL2	335.476005	1.99144379	7.76E-05	0.00240019
DNAJB4	626.61137	1.44724042	7.88E-05	0.00243239
QKI	5965.32111	0.71687965	7.91E-05	0.00243775
DNMT3A	927.805446	-1.0083287	7.94E-05	0.00244106
ABTB2	232.164895	-1.6585692	7.96E-05	0.00244412
IL17D	73.5367361	1.93663018	8.01E-05	0.00245568
PIGM	399.055253	-0.7937959	8.06E-05	0.00246637
LMO4	3536.85782	1.55861018	8.16E-05	0.00249338
PDF	23.9345297	-1.5398567	8.18E-05	0.00249397
CEP120	1437.61695	1.22402124	8.22E-05	0.00250041
CCNI2	9.66356134	2.88536249	8.31E-05	0.00252479
GMPR	465.381328	1.65184384	8.33E-05	0.00252692
CABLES2	249.552588	-1.2358958	8.36E-05	0.0025296
CS	2566.38966	-0.7649442	8.48E-05	0.00252967
ETNK1	1832.74558	0.82162691	8.44E-05	0.00252967
FBXO8	333.779414	0.61519609	8.49E-05	0.00252967
HIST1H3C	157.737725	-1.8763874	8.41E-05	0.00252967
L3MBTL4	289.252115	1.74017481	8.41E-05	0.00252967
MCM2	339.926666	-1.2918126	8.45E-05	0.00252967
PHF3	3460.51166	0.81567075	8.49E-05	0.00252967
SCUBE2	67.0789182	2.26465289	8.49E-05	0.00252967
ZNF672	357.587882	-0.8941488	8.41E-05	0.00252967
DHRS12	147.70652	0.84435259	8.56E-05	0.00254162
DNAH9	45.2005898	2.17102557	8.57E-05	0.00254162
LOC1005066	101.449776	-2.4628654	8.57E-05	0.00254162
MEN1	407.190815	-0.6828016	8.67E-05	0.00256658
CKMT1A	9.41546909	-2.9424299	8.81E-05	0.00259295
HINT3	654.723417	1.03460684	8.79E-05	0.00259295
SPC24	26.2604581	-1.7224864	8.80E-05	0.00259295
CRYL1	521.057548	1.22211735	8.84E-05	0.00259703
IER3IP1	393.418654	0.89144267	8.87E-05	0.00260309
TP73	14.1024329	-2.0233717	8.94E-05	0.00262011
BCAM	1809.13492	-2.420144	9.12E-05	0.00264228
C9orf72	327.127198	1.3037916	9.09E-05	0.00264228
COX5B	1251.09146	-0.7763162	9.07E-05	0.00264228
CTD-2201I18	20.0337165	2.50661287	9.10E-05	0.00264228
PMS2P5	126.289064	-0.7909445	9.13E-05	0.00264228
RPL21	7.56596272	2.77170684	9.10E-05	0.00264228
ZFP2	52.6519586	1.65359319	9.09E-05	0.00264228
MCM6	507.510335	-1.0045768	9.21E-05	0.00266034
APC2	46.4191172	-2.2048045	9.45E-05	0.00272668
NDUFS8	890.748786	-0.8282238	9.50E-05	0.00273196
NR2F2	4448.64928	-2.0899679	9.50E-05	0.00273196

BVES	173.550331	2.33798944	9.56E-05	0.00274528
ANKRD13B	833.193348	-1.8138214	9.75E-05	0.00278928
OR7E2P	22.6363261	2.72066272	9.74E-05	0.00278928
CCDC146	134.14664	1.50101021	9.78E-05	0.00279082
SLC27A6	30.5007492	-2.6890319	9.80E-05	0.00279082
TSC22D3	6894.45486	1.53531031	9.80E-05	0.00279082
EHMT1	1260.95715	-0.6881422	9.86E-05	0.00280325
PTGER2	101.362545	2.23272796	0.00010217	0.00289871
ZDHHC12	155.950556	-1.1282735	0.00010271	0.0029093
KIAA1161	341.757211	-2.1544188	0.00010313	0.0029142
MYRF	68.3507771	-2.7189276	0.00010322	0.0029142
PSMB1	1032.56325	0.69186316	0.00010409	0.00293395
IL18R1	80.7247321	1.95756547	0.00010482	0.00293996
NUDT8	44.5598621	-1.4081245	0.00010474	0.00293996
TACO1	222.855513	-0.8107289	0.00010465	0.00293996
HSD17B4	1834.95704	0.70083812	0.00010531	0.00294889
ECM2	2480.8377	1.1549121	0.00010609	0.0029658
GRID1	201.862546	-2.6496958	0.0001073	0.00299484
LIMK1	555.058691	-0.9101639	0.0001077	0.00300099
C19orf66	402.274747	0.7229594	0.00010801	0.00300493
KIF2C	42.8328453	-1.6233373	0.000111	0.00308301
MRPS11	275.781889	-0.8835221	0.00011151	0.00308716
TPI1P2	15.9895174	-1.5956463	0.00011139	0.00308716
POGK	1096.09634	-0.5828263	0.00011191	0.00309316
ZNF117	1499.85531	1.18010687	0.0001123	0.00309907
ALDH1B1	306.219828	-1.3351782	0.00011313	0.00311705
TRUB2	336.876257	-0.7230646	0.00011363	0.00312565
HSPB7	78.4810681	1.92622992	0.00011422	0.00313677
SPAG8	51.2605804	1.47120029	0.0001144	0.00313677
COMTD1	43.638699	-1.3962221	0.00011549	0.00316181
ANXA2P2	87.8964958	-1.3250098	0.00011571	0.00316277
WDR12	311.855879	-0.6765557	0.00011916	0.00325176
TMX1	601.166302	0.67991768	0.00012145	0.00330907
GPR88	38.8237346	2.80414094	0.00012179	0.00331312
ATP5H	1054.35198	-0.8692094	0.00012209	0.00331591
CCSER1	98.7646479	-1.8843108	0.00012296	0.00333432
MARC2	154.133524	-1.4746532	0.00012361	0.00334131
SLC50A1	340.897958	-0.8090785	0.00012348	0.00334131
MTMR9LP	217.432809	2.18661547	0.00012423	0.00335274
PRNP	2412.42909	0.98983822	0.000125	0.00336821
PRC1	531.738753	-1.69796	0.00012548	0.00337591
SLC4A1	4.63005093	2.42326777	0.00012828	0.00344593
RPLP2	3787.55906	0.55248754	0.00012984	0.00348248
CCDC85B	104.358385	-0.9078729	0.00013159	0.00351835
RIPK3	39.7618223	1.3403813	0.00013149	0.00351835
TIMELESS	401.631126	-1.127422	0.00013299	0.00355029
DEPDC1	53.8861135	-1.9310441	0.0001337	0.00356361
UQCRC2	2133.71286	-0.7663379	0.00013475	0.00358611
CIAPIN1	471.391814	-0.775412	0.00013539	0.00358831
ERCC6L	21.3022483	-1.9648254	0.00013546	0.00358831

TSTD3	75.2298507	1.40353966	0.00013541	0.00358831
SMAD7	660.240059	1.08154594	0.00013571	0.00358942
POLN	77.5734597	1.32802293	0.0001372	0.00362309
CREBRF	1789.91482	0.72846358	0.00013998	0.00368839
LOC1005068	18.6346242	1.58540214	0.0001401	0.00368839
PDPN	852.52662	-1.4749944	0.00014577	0.00383168
IARS	3244.03228	-0.8212254	0.00014612	0.00383498
LMBR1	1345.92297	-0.923191	0.00014732	0.00385746
SFT2D1	212.002906	0.86606002	0.00014742	0.00385746
CCDC126	260.441051	1.6901095	0.00014802	0.00386125
CYP27A1	913.354954	0.8026243	0.00014788	0.00386125
PABPC3	32.6427085	-1.7372337	0.00014883	0.00387646
AKAP1	1228.87028	-1.1822414	0.00014953	0.00388892
CD82	238.571309	-1.784179	0.00015233	0.00395569
COMMD10	293.364288	1.04569473	0.00015261	0.00395715
SMC4	1277.72898	-1.2532868	0.0001531	0.00396385
EIF4A2	5280.19509	0.75897535	0.0001542	0.00398148
LZTFL1	439.450046	1.009042	0.00015425	0.00398148
LYRM2	687.908591	1.2532591	0.00015628	0.00402789
MDH1B	47.1730417	1.85376645	0.00015655	0.00402877
TRAF3IP2-AS	133.859045	1.6550858	0.00015717	0.00403876
TMEM220	40.7422903	1.6260988	0.00015931	0.00408754
SLC7A14	459.550986	-2.6869834	0.0001598	0.00409399
MPP6	1508.55775	1.92258463	0.00016022	0.00409865
LAMC1	9140.33591	-1.0575858	0.00016072	0.00410522
CTBS	404.668931	0.93383506	0.00016194	0.00413038
CLEC4E	88.6025144	2.26029415	0.00016302	0.0041424
FGF14-AS2	37.7236256	1.60355708	0.00016304	0.0041424
SLC1A1	176.119505	2.22655926	0.00016314	0.0041424
SERPINB9	800.75722	1.68232157	0.0001636	0.00414789
MAPT	53.5800608	-1.7508615	0.00016478	0.00416561
MRPS23	325.306119	-0.4756168	0.00016454	0.00416561
THEM6	322.956187	-1.1712765	0.00016531	0.00417289
HIST1H2BF	135.90679	-1.5682674	0.00016597	0.00417732
TIMP2	15337.4576	-1.4029452	0.00016577	0.00417732
BEND5	103.954372	1.95835568	0.00016721	0.0041901
C10orf2	210.79507	-1.3427407	0.00016717	0.0041901
IDUA	403.377281	-1.3818771	0.00016703	0.0041901
NPHP1	108.689468	1.43120331	0.00016814	0.00420312
NT5DC1	436.772834	1.13172376	0.00016822	0.00420312
ERV3-1	844.687903	1.03330187	0.00016916	0.00422032
FBXL5	3235.58885	0.73470678	0.00017094	0.00425249
PCNXL3	1460.06875	-0.8006624	0.00017081	0.00425249
LOC1005074	8.13585309	2.15266794	0.00017308	0.00429952
SAMD10	77.3553454	-1.8050234	0.00017345	0.00430239
LBX1-AS1	6.72389605	-2.7623232	0.00017674	0.00437778
MRPS2	338.229981	-0.8415276	0.00017718	0.00438232
C9orf16	448.900992	-1.0814935	0.000178	0.00438992
HES7	7.06358606	-1.8078519	0.00017777	0.00438992
FAM199X	1879.21858	1.18135795	0.00017858	0.00439263

LINC00222	7.00281924	2.44689419	0.00017862	0.00439263
KCNK10	22.3266702	2.62817117	0.00017931	0.00440333
C17orf89	191.105613	-0.9962898	0.00018239	0.00446967
ZNF345	163.831504	0.88053482	0.00018254	0.00446967
CENPN	154.729313	-1.4068752	0.00018315	0.00447841
ZNF775	82.7527897	-0.8315043	0.00018388	0.0044898
CDC25A	63.8150687	-1.6605987	0.00018518	0.0045105
DDAH1	883.720379	1.56776742	0.00018525	0.0045105
MAP1LC3C	28.8368735	-2.5717236	0.00018571	0.00451522
HLTF	1815.90079	-0.6487671	0.00018731	0.00452973
MKRN7P	25.706245	2.00038041	0.00018736	0.00452973
SMYD5	336.704422	-0.818111	0.00018691	0.00452973
SNORA46	6.94855576	2.57228365	0.00018732	0.00452973
C19orf48	388.061252	-0.9302439	0.00018775	0.00453264
SHPRH	1146.98563	0.70114834	0.00018847	0.00454371
TSEN54	195.462697	-1.4852239	0.00018961	0.00456479
CLEC16A	1203.50919	-0.9899233	0.00019203	0.00461642
TTYH1	12.8939837	-2.5504251	0.00019273	0.00462693
EPN3	39.0606446	-2.5126434	0.00019365	0.00464252
CTXN3	1148.98939	2.56934706	0.00019435	0.00465283
RAD51	41.4549046	-2.028272	0.00019513	0.00466496
LOC1006535	118.069318	-1.4415257	0.00019652	0.00469157
KIF23	504.074339	-1.8319492	0.00019685	0.00469298
TUBE1	172.559197	0.69283897	0.00019801	0.00471414
TMEM220-A	3.66909545	2.59235512	0.00019864	0.00472259
ITGB5	1486.03279	1.38972292	0.00020065	0.00475976
KIF22	318.336416	-0.8458277	0.00020076	0.00475976
CLEC9A	94.168264	2.56631347	0.00020151	0.00476182
KLHL8	379.232307	0.90601315	0.00020161	0.00476182
RGS7BP	131.521175	2.45966387	0.00020168	0.00476182
IGFL2	9.80452004	-2.7067203	0.00020623	0.00486262
BRIP1	145.270013	-1.6470914	0.00020655	0.00486345
WTAPP1	2.23001789	-2.7925802	0.00020787	0.00488772
MEDAG	360.800744	2.32246218	0.00021048	0.00494248
RNFT2	172.232107	-2.1771785	0.00021284	0.00499103
CD200	81.4677834	1.78789969	0.00021609	0.00505838
CDC14A	575.34166	1.26469451	0.0002166	0.00505838
MGAM	144.19361	1.97981563	0.0002164	0.00505838
TMEM219	792.831119	0.52105167	0.00021755	0.0050736
KIF5C	103.809846	-2.1571787	0.00021979	0.00511595
ZBTB24	507.794376	1.07530935	0.00021996	0.00511595
PITPNM3	157.055628	2.06480758	0.0002205	0.00512174
CLPB	490.44286	-0.465431	0.00022229	0.00514604
HAPLN4	64.0068092	-2.3859077	0.00022245	0.00514604
SETD5	3290.06041	-0.7038004	0.00022239	0.00514604
HIST2H2BE	1047.9319	-1.3371906	0.00022307	0.00515351
ELL2	1144.52413	1.70244815	0.00022603	0.0052147
FAM198A	1888.23166	1.85143871	0.00022733	0.0052377
CLUH	966.350207	-0.7759485	0.00022768	0.00523798
FREM2	4270.30067	-2.3613957	0.00022795	0.00523798

SHCBP1	101.887209	-1.6350906	0.0002283	0.005239
LAMC2	105.797996	-2.1184559	0.00022915	0.00525151
OLFM4	156.839495	-2.2658996	0.00023002	0.00526441
ATAD5	200.605286	-1.120538	0.00023572	0.00538774
ECHDC1	615.319592	0.7457743	0.00023685	0.0054005
RAD51AP1	64.5557327	-1.3771117	0.00023691	0.0054005
ARFGAP2	1043.05913	0.61684891	0.00023864	0.00543261
ABCB6	558.991916	-0.8673554	0.00024024	0.00546192
FAM180A	164.527557	2.70583731	0.00024115	0.00547536
ECT2	364.725971	-1.3469425	0.0002432	0.00551457
ALYREF	411.112032	-0.6654804	0.00024408	0.00552715
RPS12	4924.09275	1.02797183	0.0002451	0.00554305
ATHL1	630.743052	1.69968435	0.00024592	0.00555433
TATDN2	1331.63807	-0.7097336	0.00024757	0.0055842
BAALC	71.4713469	-2.4013083	0.00024814	0.0055896
SERPINB1	1265.14137	1.06020756	0.00025026	0.00563014
GPR19	4.51231855	-2.3405214	0.00025067	0.00563186
CNIH3	29.2256758	1.58847743	0.00025177	0.00564923
PECAM1	500.989592	1.30065593	0.00025252	0.00565114
STK31	30.9713347	2.01317594	0.00025247	0.00565114
SULT1E1	305.737938	-2.7503437	0.00025334	0.00566217
MYO19	754.798381	-0.9774903	0.00025391	0.00566753
COX6A1	1643.23631	-0.8745413	0.00025482	0.00568043
ISG20L2	465.305487	-0.5923765	0.0002558	0.00569485
VGF	1.92302427	-2.7568652	0.00025636	0.00570001
NTNG1	51.024001	-2.7255356	0.0002574	0.00571533
UFSP1	18.8172231	-1.4538489	0.00025771	0.00571533
ARL14EP	347.435451	0.84196092	0.00025885	0.00571833
NELFB	685.353606	-0.5391594	0.00025874	0.00571833
ZNF367	187.280192	-1.314402	0.00025851	0.00571833
GNMT	11.330862	1.90535331	0.00025973	0.0057304
PABPC1	8493.45743	-0.9059963	0.00026082	0.00574705
SLC15A2	317.112991	1.80387542	0.00026206	0.00576704
TOMM7	1399.7614	0.93116056	0.00026371	0.00579586
IZUMO4	41.4960666	1.18277478	0.00026539	0.00582526
PRSS23	864.365955	1.57010068	0.00026623	0.00583642
SGK494	231.068438	-1.3238711	0.00026804	0.00586861
ROGDI	407.93226	-1.1226007	0.00027051	0.00591495
WDR78	118.769446	1.90270867	0.00027165	0.0059325
CDH1	7468.2671	-1.2556474	0.00027227	0.0059384
DRICH1	7.22245582	2.21278142	0.00027426	0.00597417
CCDC13	9.51709794	2.00881552	0.00027527	0.00598696
YPEL3	529.709671	0.76011965	0.00027556	0.00598696
ZDHHC23	49.3409826	-1.8335836	0.00027589	0.00598696
STAG3L2	473.697172	-0.7593672	0.00027732	0.00601024
BCL11A	17.5351864	-2.1736591	0.00027897	0.00603838
CIRBP	5289.07344	0.88801836	0.00028152	0.00608602
FGFR1OP2	662.61098	0.62006022	0.00028336	0.00611805
PPP6R3	2851.30307	-0.4086823	0.00028716	0.00618193
RPL10	7952.20635	0.75038017	0.0002874	0.00618193

SYNCRIP	2852.04262	0.47212743	0.00028683	0.00618193
UHRF1	102.61957	-1.483989	0.00029104	0.00625252
NEDD9	655.022411	1.52155047	0.00029197	0.00626461
ATP6V0E2	549.051721	-0.9615738	0.00029274	0.0062655
EIF3G	1276.96924	0.66037225	0.00029242	0.0062655
CASD1	552.330167	0.56382499	0.00029335	0.00627076
CCDC11	12.7544481	1.70852875	0.0002944	0.00627399
GNAI1	309.201718	-1.2610589	0.0002946	0.00627399
TRIM22	4155.54578	0.82745872	0.00029429	0.00627399
RABGAP1L	3297.7452	0.96628967	0.00029734	0.00632444
HRASLS5	64.0179346	2.61804389	0.00030002	0.00636779
NEK11	154.088402	1.53529042	0.00030012	0.00636779
CHRNA5	25.3652833	-2.1777738	0.00030154	0.00639002
ATCAY	3.94507772	-2.7236418	0.00030655	0.00648815
RPL22	2195.97502	0.66388227	0.00030725	0.00649501
ASCC3	2055.64086	0.81505546	0.00031222	0.00659186
TCF7L1	682.76036	-1.1847665	0.00031293	0.00659878
VWA3A	12.2849723	1.92270628	0.00031464	0.00662665
FAM43A	818.239836	-1.7119285	0.00031828	0.00669518
ANAPC11	425.779214	-0.6814031	0.00032019	0.00672712
KNOP1	203.472487	-0.822768	0.00032101	0.00673518
MYCL	129.329519	2.15951465	0.00032136	0.00673518
SLC44A1	2765.41702	1.49972265	0.00032243	0.0067412
ZNF324	229.567101	0.85585224	0.00032205	0.0067412
AP1S2	366.618719	1.29005768	0.00032316	0.00674826
UTRN	10341.317	0.94465981	0.00032599	0.00679908
ACACA	2473.18793	-0.66383	0.00032761	0.0068245
SNORA48	619.0239	1.48169208	0.00032819	0.00682842
AACS	601.710288	-1.002899	0.00033088	0.00686769
STK33	237.241703	1.72459358	0.00033066	0.00686769
AFF1	6208.74632	0.88285531	0.00033315	0.00690642
TPX2	390.788123	-1.8436142	0.0003338	0.00691154
KIAA1467	424.627052	1.22224089	0.00033507	0.00692957
CLMN	923.807581	-1.44638	0.00033623	0.0069452
BVES-AS1	4.53477223	2.60633349	0.00033695	0.00695154
TBX3	33.676083	-2.1322251	0.00033835	0.00697212
SMU1	1469.66871	0.51103278	0.00033946	0.00698656
ANKRD31	45.0577375	-1.8227088	0.0003408	0.00700163
NDE1	350.182163	-0.7326301	0.00034101	0.00700163
DAAM2	628.752793	1.89413437	0.00034402	0.00702463
EHD3	240.767178	-1.6482593	0.00034425	0.00702463
MAZ	1359.05598	-0.7936333	0.00034372	0.00702463
PRR12	1120.22057	-0.8146115	0.00034499	0.00702463
SLC19A1	114.915368	-1.4145532	0.00034421	0.00702463
WBP4	299.217104	0.70031478	0.00034295	0.00702463
ZNF280C	200.003222	-0.5553735	0.00034499	0.00702463
DUXAP10	24.6485984	-2.1929618	0.00034606	0.00703807
GKAP1	91.8152254	0.8986497	0.00034747	0.00705836
MRPL24	487.134941	-0.875862	0.00034908	0.00707775
PLEKHA5	732.073681	1.41394243	0.00034925	0.00707775

AMN1	101.61935	0.7697038	0.00034974	0.00707932
CLUAP1	430.119637	0.76276047	0.00035217	0.00710834
EFNA2	15.0232553	-1.6989717	0.0003518	0.00710834
ZNF629	1094.04721	-0.5937022	0.00035241	0.00710834
ADORA1	15.2153744	-2.0683476	0.00035569	0.00712571
HIST1H2BB	80.4871698	-1.6313733	0.00035577	0.00712571
ICA1L	331.577227	1.39448677	0.00035497	0.00712571
MT3	88.2658974	-2.3415072	0.00035381	0.00712571
MYO6	1747.78964	1.43897397	0.00035423	0.00712571
PBK	37.2225468	-1.9334803	0.00035467	0.00712571
PURA	1612.97774	0.89935678	0.00035704	0.0071428
ADAMTSL1	1852.45158	2.4035538	0.00035756	0.00714468
APLP2	19794.6459	-1.1439583	0.00035838	0.00714468
FEZF1-AS1	12.441937	-2.5732274	0.0003582	0.00714468
CYTH2	834.183125	-0.8593085	0.00035913	0.00715138
KLHDC3	1157.79518	0.84009354	0.00036115	0.00718329
SLC2A13	246.007447	1.30967873	0.00036206	0.00719308
SGK1	3798.39874	1.71293932	0.00036262	0.00719573
PVRL1	486.440156	-1.3384071	0.00036314	0.00719773
DPY19L2	220.61414	1.63114929	0.00036391	0.00720009
ZBTB48	205.379868	0.74483056	0.00036409	0.00720009
KRT4	453.065284	-2.6299706	0.00036869	0.00728264
TGFBR3	2424.63084	1.08442075	0.00037029	0.00730572
FBXO22-AS1	1.95442496	-2.6353469	0.00037084	0.00730819
COL5A1	7409.96485	-1.6046327	0.00037137	0.00731034
C1QTNF7	44.6914944	2.50126262	0.00037236	0.00732136
SLC47A1	10194.2277	2.03960855	0.00037902	0.00744373
COL15A1	2588.66501	-2.3350516	0.00038695	0.00759076
AURKA	88.5183427	-1.9142647	0.00039007	0.00760958
LRRC71	48.8388597	-2.6009	0.00038917	0.00760958
MBD1	912.025414	0.60797465	0.00039015	0.00760958
MX1	1313.02479	0.78456816	0.00038841	0.00760958
PIGR	31.4368485	2.62055144	0.00039056	0.00760958
ROBO4	278.578547	1.5555926	0.00038932	0.00760958
HIVEP2	1274.95902	0.88478189	0.0003921	0.00763074
LRRC43	29.8395875	1.94044149	0.00039298	0.00763937
C1orf61	5.65114718	-2.6721476	0.00039502	0.00767023
LIX1	23.2924236	-2.4326274	0.00039574	0.00767559
GFRA2	8.90015368	-2.4539263	0.00039779	0.00770655
AKAP7	222.618187	1.51414192	0.00039979	0.00771918
C1QTNF6	210.90213	-1.3156552	0.00039905	0.00771918
RPP25	126.694315	-2.0517664	0.00039947	0.00771918
SNHG5	398.424507	1.56288703	0.0004014	0.00774163
IGSF9B	478.316946	-1.8389657	0.00040291	0.00776201
POLR1A	1424.26359	-0.6260271	0.00040667	0.00782561
SIPA1L1	6930.61488	1.56020076	0.00041167	0.00791303
FGF19	3.42398509	-2.6547609	0.00041317	0.00793304
IGSF5	16.9181552	-2.3804865	0.00041544	0.00796761
HOXC13	4.01527941	-2.6468542	0.00041699	0.00798848
C6orf165	11.4857626	1.90845204	0.00041777	0.00799438

HIST1H3G	125.291072	-1.957324	0.00041858	0.00800094
LRCH4	1380.28941	-0.8869965	0.00042027	0.00801721
TCP10L	25.4245876	2.00348677	0.00042036	0.00801721
ALPK3	372.864107	1.69378759	0.00042171	0.00802785
MDN1	2624.69475	0.57704081	0.00042185	0.00802785
GRIN1	3.56929355	-2.6211876	0.00042274	0.00803588
AHCTF1P1	13.9984223	-1.3207502	0.00042327	0.00803691
SPRY1	883.873878	1.51811925	0.00042475	0.0080561
LIG1	478.567492	-0.9787635	0.0004255	0.0080615
LY75	42.8714803	1.77188116	0.00042645	0.00806309
PCNXL4	953.39378	0.71159023	0.00042652	0.00806309
NQO1	1214.71305	-1.4251166	0.00042933	0.00810723
AGAP3	1057.12357	-0.5556818	0.00043016	0.00811389
C11orf49	425.11031	1.02951865	0.00043121	0.00812366
CHN2	333.07502	2.14774824	0.00043162	0.00812366
TMEM143	105.593209	-0.6951397	0.00043524	0.00818283
PHKB	1920.71511	-0.4251158	0.00043808	0.00822717
POC1A	42.6338936	-1.2196332	0.00043986	0.00825158
C1orf228	12.7235425	1.74457565	0.0004428	0.00829763
PSTPIP2	183.86777	1.25988232	0.00044346	0.00830094
CYP2B7P	6.96294209	2.31578199	0.00045037	0.00842121
FAM227A	60.8260312	1.49322171	0.00045161	0.00843512
FAM53C	741.649547	0.81840915	0.00045365	0.00845511
IGF2R	5656.93894	1.24874133	0.00045366	0.00845511
TRIM38	976.435706	0.68409611	0.00045482	0.0084674
CENPP	191.080109	-0.989087	0.00045681	0.00849024
FOXD2-AS1	580.797422	-0.9114837	0.00045753	0.00849024
KIAA1324L	2748.79652	1.48542067	0.00045704	0.00849024
ZNF280A	3.61939963	-2.5968003	0.00046161	0.0085567
ATG5	447.358585	0.59771462	0.00046382	0.00857342
BCLAF1	4208.30812	0.45696269	0.00046308	0.00857342
TMEM132A	352.47559	-1.6526519	0.00046401	0.00857342
MIR4697HG	307.803117	-1.8052062	0.0004653	0.00858801
FLJ22184	16.6427191	-2.0472247	0.00046583	0.00858864
TTC9C	252.943509	-0.5177111	0.00046751	0.00861028
AHI1	723.757944	1.51247342	0.00047058	0.00864826
CHDC2	19.0498656	-2.5021497	0.00047037	0.00864826
TXNDC16	766.549345	0.94596834	0.00047172	0.00866006
HIST1H2AH	146.020914	-1.3465764	0.00047492	0.00870946
BRI3BP	156.965994	-1.0533122	0.00047725	0.00874288
KCNG3	19.0886033	-2.3131418	0.00048022	0.00878784
TFG	1539.74386	-0.7803391	0.00048192	0.00880945
NCAPD3	588.039419	-0.6544449	0.00048346	0.00882835
TCTN1	494.930716	0.86702725	0.00048767	0.00889566
PTGES2	541.733352	-0.9450819	0.000491	0.00894692
ARHGEF18	637.881684	0.48352742	0.00049331	0.00897958
KDM5A	3425.55025	0.62310116	0.0004948	0.00899705
LOC1019278	13.6415573	1.99346691	0.00049571	0.00900416
ENPP1	182.734759	1.99112161	0.00049705	0.0090189
ZC2HC1C	190.43529	2.06617756	0.00049976	0.00905856

LOC645752	37.683026	-2.4711644	0.00050217	0.00909249
TRAF3IP2	292.447989	0.90113694	0.00050501	0.00913428
GANAB	7036.31685	-0.5588857	0.00050808	0.0091802
GLYCTK	256.651058	1.00326871	0.00051652	0.00932288
DENND6B	75.1952559	0.87867641	0.00051878	0.00932427
SFXN1	729.138422	-0.972464	0.00051926	0.00932427
SORD	261.05509	-1.6133691	0.00051931	0.00932427
ZNF709	202.976884	1.2710995	0.00051773	0.00932427
ZNF786	169.182907	-0.8853399	0.00051872	0.00932427
C6orf120	1069.79294	0.63687579	0.00052222	0.00935846
CLSPN	120.890194	-1.4157742	0.00052231	0.00935846
AKR1B10	34.7815551	2.37070458	0.00052405	0.00937009
CD276	1494.62004	-1.0966522	0.00052381	0.00937009
MSH4	37.2783773	2.23438271	0.00052592	0.00939374
SERGEF	241.94896	0.82451026	0.00053022	0.00946065
NR4A2	177.499009	1.77336766	0.00053102	0.00946509
CCDC23	125.917647	1.03644508	0.00053355	0.00950046
LINC00607	219.169028	1.93491428	0.00053908	0.00958886
TBPL1	142.037179	1.01580226	0.0005422	0.0096344
CENPM	19.4258523	-1.7363687	0.00054343	0.00964157
ZSWIM7	129.849363	0.96215895	0.00054373	0.00964157
IFNGR1	4121.99499	1.22723329	0.00054738	0.00969643
LINC00701	8.25806476	-2.6017299	0.00054798	0.00969697
SNAP25	45.1372069	-2.5578433	0.00055392	0.00979198
ATP2B4	11224.2585	-0.8012469	0.00055539	0.0098035
PHB	1417.64125	-0.6264266	0.00055571	0.0098035
CMTM2	5.48949655	2.32230766	0.00056154	0.00989621
TVP23C	356.452231	0.97451054	0.00056295	0.00991084
PAFAH1B3	121.081069	-1.1181021	0.0005641	0.00992086
ZNF620	72.4764744	-0.9863669	0.0005677	0.00997396
FADS2	1395.51986	-1.5303249	0.00056936	0.00999286
CDADC1	165.734835	0.99768939	0.00057421	0.01006782
LINC00158	13.6793854	2.16790398	0.00057906	0.01014234
METTL7A	6182.46124	0.65708942	0.0005824	0.01019046
ARID1B	2042.43249	0.65631411	0.00058487	0.01021283
GPATCH3	129.474668	0.87759701	0.00058444	0.01021283
ZDHHC9	543.702004	-0.9479827	0.0005888	0.0102711
RBFOX1	21.3835807	-2.5506911	0.00059633	0.0103919
PTPRZ1	18.5795642	-2.5049354	0.00059696	0.01039231
FAR2P1	7.22182673	-2.5839337	0.00059993	0.01043341
C5orf56	52.6780376	1.29945712	0.00060508	0.01051232
ABCA3	2128.90513	-0.9475983	0.00061301	0.01063927
ATAD2	628.266586	-1.0590913	0.00061454	0.01064586
DIP2B	3110.53466	0.92607468	0.00061463	0.01064586
FEN1	274.105763	-0.8894933	0.00061654	0.01066823
SP4	335.726343	0.8365278	0.00061806	0.01068375
KRT8	291.577062	-2.4465368	0.00062066	0.01071798
CHD3	9304.14622	0.8638745	0.00062238	0.01073155
KLHL29	1517.56186	-1.971033	0.0006227	0.01073155
CDKAL1	426.643791	0.87128862	0.00062661	0.01075478

NFE2L2	3043.2062	-0.8009025	0.00062524	0.01075478
PKD1L1	34.1632979	1.1771564	0.00062718	0.01075478
SPIRE2	169.521862	-1.7734803	0.00062701	0.01075478
TNNC2	10.4054188	2.09127642	0.00062552	0.01075478
HSP90AB1	11876.0734	0.54912065	0.00063429	0.01086585
CLEC4A	56.5060472	1.3518071	0.00063696	0.01090043
NAV1	4883.5019	-1.4490387	0.00063758	0.01090043
EP400	2598.34814	-0.7586582	0.0006391	0.01091556
PLEKHA6	7656.49845	1.47892494	0.00064083	0.01092596
PRCC	644.901729	-0.6471207	0.00064098	0.01092596
PMPCA	568.093282	-0.6324413	0.00064322	0.01095332
KNTC1	754.776904	-0.9587772	0.00064476	0.01096861
CELF4	15.2574098	-2.0726593	0.00064817	0.01101568
VAMP5	175.638105	1.36506193	0.00064889	0.01101702
THSD7B	75.8133064	-2.3736436	0.00065513	0.01111201
KIAA0753	471.568142	0.75042213	0.00066082	0.0111974
GTSE1	34.7143867	-1.652473	0.00066236	0.01121093
TSPYL4	856.969345	1.00785359	0.00066292	0.01121093
GSK3B	1761.52723	-0.4823907	0.00066538	0.01124147
MTERF	219.676622	0.7231728	0.00066741	0.01126453
CITED2	1808.55421	1.1659712	0.00066923	0.01127316
SLC6A6	1359.72084	1.69638836	0.00066863	0.01127316
GALK1	200.079925	-1.0292721	0.00067101	0.011292
FBXO41	466.565362	-1.186262	0.00067439	0.01133781
ERG	263.338361	1.53195083	0.00068209	0.01145604
TGIF1	447.221141	1.06593959	0.0006838	0.01147351
HEBP2	285.393065	1.09503245	0.0006848	0.01147917
CDH26	20.8486687	1.63060332	0.00068803	0.011522
ATP5G1	406.348868	-0.616238	0.00068934	0.01153267
ARSA	504.567451	0.96638373	0.00069163	0.01155968
AGBL1	71.4873392	-2.5555105	0.00069544	0.01161215
KIF13A	1826.96573	0.85809151	0.00069706	0.0116279
ARL16	303.93674	-0.7080554	0.00070145	0.01168971
KCNA3	36.8001507	1.56119386	0.00070223	0.01169141
LOC1002894	23.1401822	1.44852706	0.00070416	0.01170077
PGM3	1062.47679	0.92753263	0.00070359	0.01170077
LAMA3	120.226581	1.67962731	0.00071015	0.01177744
LINC00312	31.537788	2.22219066	0.00070956	0.01177744
PRDM5	648.327222	0.58186707	0.00071301	0.01181353
HPS5	1020.79856	1.18090256	0.00071676	0.01183003
MANEA-AS1	44.0807263	1.21652785	0.0007162	0.01183003
PSME3	1219.87948	-0.5683357	0.00071676	0.01183003
RAB11B-AS1	43.0759939	0.98143442	0.00071641	0.01183003
LOC652276	102.971761	-0.7861904	0.0007198	0.01186871
SMPD4	878.211737	-0.6659528	0.00072127	0.01188154
RAD54L	32.7019723	-1.4802353	0.00072537	0.0119262
RTN4R	82.9478643	-2.0542796	0.0007249	0.0119262
CXCL12	1501.52191	-1.7522579	0.00072781	0.01195494
ETV4	72.2173791	-2.1214601	0.00073403	0.01204558
TRAF2	152.357627	-0.672361	0.00073713	0.01208493

CXCR2	22.0034779	1.36684933	0.00074017	0.01212318
CXXC1	472.669965	0.74988511	0.0007418	0.01213747
DEDD	443.796016	-0.4512689	0.0007428	0.01213747
EEFSEC	207.238608	-0.6317904	0.00074328	0.01213747
HELLS	272.229174	-1.2759679	0.00074388	0.01213747
DEPDC5	342.36894	-0.71447	0.00074775	0.01218918
GEM	159.050382	1.61330438	0.0007488	0.01219463
KIAA1109	13290.0036	0.84224612	0.00075396	0.01226585
LINC00526	24.2214655	1.15954494	0.00075486	0.01226585
SKA3	45.1535573	-1.693946	0.00075532	0.01226585
ZNF768	452.850313	-0.6212267	0.00076331	0.01238402
GAS2L3	98.3848839	-1.1997331	0.00076684	0.01242941
RSG1	72.8437744	1.14635632	0.00077373	0.01252934
STRIP2	464.582219	1.55751546	0.00077577	0.01255048
VGLL4	3631.38672	-1.5983697	0.00077676	0.0125547
FAM13B	1561.13317	0.7829212	0.0007776	0.0125564
GRAMD4	352.805878	0.77383987	0.00078946	0.01272397
RGS6	120.899621	2.45445058	0.00078941	0.01272397
OR51B5	21.7851859	1.51782695	0.00079386	0.01278258
ZNF781	103.333209	1.72735381	0.00079458	0.01278258
HIST2H2BC	4.62248636	-1.947198	0.0007967	0.01280467
APOL3	222.152169	1.26080589	0.00079951	0.01283774
MRRF	361.857272	-0.6480316	0.000801	0.01284968
SOX8	19.3811529	-1.599816	0.00080493	0.01290072
SLC7A1	908.847362	-2.1094332	0.00080579	0.0129024
TEX41	10.7699936	2.10446287	0.00080726	0.01291398
FANCI	376.9013	-1.4713748	0.00080921	0.01292099
PIK3CA	1256.98526	0.6452013	0.00080873	0.01292099
POLDIP2	1321.63677	-0.6357983	0.00081207	0.01295468
URM1	535.743765	-0.3947559	0.0008134	0.01296386
IMPG2	132.547955	1.87564409	0.00081873	0.01303661
MMRN2	280.507869	1.52336773	0.00081971	0.01304014
HIST1H2AM	160.33861	-1.4159917	0.00082071	0.01304397
BPTF	5759.07026	-0.5260146	0.00082162	0.01304639
CCNYL1	570.015206	-0.8554106	0.00082421	0.01307536
ZNF846	495.024866	1.05474189	0.00082588	0.01308977
ASNS	414.753388	-1.0760397	0.00083107	0.01315105
HIST2H2AC	705.021089	-0.7854599	0.00083128	0.01315105
LINC-PINT	854.531119	0.9392736	0.00083346	0.01317347
CRIP3	6.57396564	2.25831173	0.00084375	0.01332387
FBXO30	397.274999	0.68010512	0.00084661	0.01335667
API5	1232.93009	0.6437148	0.0008552	0.01338616
MTMR3	1406.05999	0.53713502	0.00085254	0.01338616
NCAPG2	371.805271	-1.1149418	0.00085184	0.01338616
NOL10	544.498879	-0.4513555	0.00085102	0.01338616
PAX9	5.73947602	-2.5155941	0.00085132	0.01338616
QPCT	44.2580963	1.91300845	0.00085582	0.01338616
SLC12A1	82.9767408	2.3688517	0.00085628	0.01338616
SNORA81	326.624824	-1.0360242	0.00085561	0.01338616
SORL1	4292.59305	1.85074367	0.00085604	0.01338616

SUGT1P1	22.5275922	1.45310488	0.00085514	0.01338616
HEPACAM	8.42451798	-2.4331217	0.00085922	0.01342
FARSB	579.250145	-0.7385949	0.00086331	0.01347152
LOC1001315	769.435829	0.95873989	0.00086551	0.01349363
TESK2	81.8882445	1.5007278	0.00086708	0.01350581
TPTE2	7.92049855	2.21414128	0.00087311	0.01358739
SH3PXD2A	12566.7607	-0.99326	0.00087616	0.01362249
CAB39L	2718.98204	1.78895093	0.00087959	0.01366348
ZNF211	289.057166	0.73026132	0.00088072	0.01366869
PCDH10	10.2754896	-2.3483987	0.0008825	0.01367152
RAB32	152.945861	0.97009227	0.00088245	0.01367152
DCT	11.0323714	1.81197063	0.00088572	0.01370909
TMEM97	409.811063	-1.0603448	0.00089002	0.01376328
POTEF	19.9034784	-1.9900269	0.00089095	0.01376516
TNRC6C-AS1	234.433402	-1.0205555	0.00090117	0.01391066
WASH7P	177.923923	0.71644305	0.00090301	0.01392651
GTF3C2	971.728105	-0.5848217	0.00090947	0.01401354
ARHGDIA	3365.20458	-0.7468638	0.0009134	0.01406147
PODN	824.051439	1.35421549	0.00091532	0.01407832
MZT2A	226.557619	-0.841242	0.00091877	0.01411878
RAB6C-AS1	52.750054	-2.4791947	0.00092657	0.01422593
CRYBB3	19.752094	-1.873805	0.00093471	0.01433799
SLC26A4	18.4937026	1.84715152	0.00093949	0.01439848
XAF1	760.702177	1.12881896	0.00094074	0.01440488
AGPAT4-IT1	40.3628248	1.4021336	0.00094228	0.01441555
FSIP1	13.0745615	1.96424024	0.00094943	0.01449903
ZHX3	3305.23335	0.94441283	0.00094942	0.01449903
RBM20	40.6283621	1.8180211	0.000951	0.01451014
MKNK1	801.661173	0.72744228	0.00095586	0.01456397
VANGL2	516.451115	-0.9251137	0.00095622	0.01456397
SHISA9	113.475738	-2.4697819	0.00096233	0.01464401
BLNK	254.60909	1.30838655	0.00096752	0.01470998
KIAA0195	1376.32117	-0.6742268	0.00096954	0.01472761
ADAM20P1	95.4001076	1.07912814	0.00097107	0.01473778
LDHD	57.4363316	2.0810704	0.00097869	0.01484029
OIP5	13.6749362	-1.8529186	0.00098239	0.01488327
NUPL2	282.443895	0.66448509	0.00098696	0.01493935
ORC6	47.0312714	-1.6539333	0.00099024	0.01497567
ADM2	17.0887549	-2.4833428	0.00099381	0.01500576
DBF4	112.437692	-0.7421557	0.00099397	0.01500576
FAM136A	324.771358	-0.7351868	0.000998	0.0150533
HOXA3	9.57066427	-2.4390696	0.00100047	0.01507736
MTSS1L	1950.35909	-1.3330283	0.00100323	0.01510563
DDX60L	841.930693	1.03687265	0.00101109	0.01514432
FAM200A	140.450393	0.80604145	0.00100946	0.01514432
IGFBP5	71990.9742	1.79861798	0.00100916	0.01514432
MAPK7	387.18569	-0.4740265	0.00101079	0.01514432
SLC6A9	88.8878923	-1.6424541	0.00100728	0.01514432
TMEM164	598.739427	-1.5243541	0.00100831	0.01514432
ATXN2L	1903.58571	-0.8029012	0.00101208	0.01514588

GTF3C6	288.640715	0.71571628	0.00102437	0.01530628
HIST1H3D	243.503527	-0.8571971	0.00102458	0.01530628
BANF1	512.565118	-1.1107869	0.00102885	0.01535677
ANGPTL7	159.347297	1.89685618	0.00103533	0.01543589
C11orf84	255.113969	-0.7763858	0.00103685	0.01543589
TEX36	11.7532417	-2.0582752	0.00103629	0.01543589
FLJ44511	23.2600253	1.48068918	0.00103848	0.01544668
LRP1B	4730.88506	2.11828722	0.00104043	0.01546225
CDC5L	1168.58877	0.71811968	0.00104228	0.01547636
NDUFS2	1026.07198	-0.6910532	0.00104694	0.01553219
CDON	2026.44798	1.7283299	0.00105006	0.015565
WDR6	3278.65058	0.53714803	0.00105129	0.0155698
LMCD1	293.560468	2.1149796	0.00105839	0.01564789
RAD23B	3638.12527	-0.5851232	0.00105823	0.01564789
CDK16	1259.79543	-0.6407904	0.00106956	0.01577217
FZD10	34.6450296	-1.8785482	0.00106784	0.01577217
LMNB2	830.929838	-0.8714802	0.00106999	0.01577217
SLC8A3	11.3764376	2.17544036	0.00107047	0.01577217
POLR1B	680.886028	-0.7473993	0.00107212	0.01578301
SLC12A2	6227.24271	1.50490712	0.001075	0.01581187
BTBD8	31.8693083	1.37381761	0.0010768	0.01582466
GNG10	82.1661998	0.59854729	0.00108078	0.01585791
POLD1	352.813441	-0.8032767	0.00108091	0.01585791
FIRRE	88.4445081	-2.1848263	0.00108281	0.01587229
TUBG1	442.60965	-0.7501704	0.00108842	0.01594083
C11orf30	664.406054	-0.4286919	0.00109067	0.01596029
THUMPD1	1454.86319	0.54914854	0.00109364	0.0159827
TTK	46.2068963	-1.5909549	0.00109407	0.0159827
KIF27	198.723246	0.98743832	0.00109549	0.01598993
ANKRD20A5I	164.038219	2.35921871	0.0010974	0.0160008
ARHGAP6	343.355205	1.72580126	0.00109904	0.0160008
MFAP2	271.579729	-2.1492945	0.00109825	0.0160008
NOL11	672.256752	-0.5515633	0.00110058	0.01600975
GJB2	2323.56971	-1.2216983	0.00111424	0.01619475
ITGA1	2831.42337	1.18643779	0.00111572	0.01620241
METTL13	463.571822	-0.6591453	0.0011171	0.01620887
NUMBL	1017.90667	-0.8866368	0.00112197	0.01623835
PIP5K1A	1517.39512	-0.6848042	0.00112184	0.01623835
TSGA10	114.688363	1.15096485	0.00112088	0.01623835
HSPA1B	38.0865077	1.88201411	0.00113418	0.01640121
PPM1G	1029.99977	-0.5481927	0.00113535	0.01640424
DHRS9	8.59919656	2.07229699	0.00114666	0.01651616
HS6ST1	981.297563	-1.1076362	0.00114594	0.01651616
KIAA1107	127.202916	1.26933344	0.00114523	0.01651616
MALT1	703.414512	0.83104522	0.00114694	0.01651616
CLN5	871.077128	0.81041164	0.00114873	0.01652807
PGAM5	441.661146	-0.7254994	0.00115234	0.01656615
TBL1X	1058.13907	-0.9528603	0.00115926	0.01665158
RTN3	2431.61011	-0.5887026	0.00116699	0.0167487
RPS2	10162.5887	-0.8593592	0.00116901	0.01675658

TOPBP1	1027.30037	-0.6243485	0.0011695	0.01675658
N4BP2L1	221.310491	1.30641388	0.00117252	0.01678591
CEND1	25.4753316	-2.0891728	0.00118086	0.01689122
LDB1	1356.77231	-0.7355417	0.00118411	0.01692365
DTYMK	158.971844	-0.9208962	0.00118594	0.01693562
CALCOCO1	4603.2491	0.9447423	0.00119208	0.01696681
DUSP2	56.0722365	2.03554546	0.00119137	0.01696681
LOC399715	74.3065689	1.58225127	0.00119032	0.01696681
RNF44	822.404203	-0.5585062	0.00119046	0.01696681
C2orf43	656.493042	-0.5790887	0.0011989	0.01704984
SLC24A1	559.956949	-0.9731202	0.00120182	0.01706303
UBN1	1063.09455	-0.4098324	0.0012012	0.01706303
DUSP1	9526.6895	1.69431309	0.00120415	0.01708128
GNAZ	163.723893	1.54890516	0.00120509	0.01708128
SRSF5	3314.42385	0.84733288	0.00120766	0.01710351
TAF4	371.591571	-0.9113338	0.00121476	0.01718989
RBM6	2258.99966	0.69247026	0.00121965	0.01723098
RPGR	117.521233	1.20073122	0.00121967	0.01723098
MARCH8	807.358721	0.63060868	0.00122345	0.0172694
NEB	861.749288	1.8857499	0.0012248	0.0172694
TCF3	805.010059	-0.6407309	0.00122541	0.0172694
KL	20.7511832	1.81634859	0.00123353	0.01736964
TTC37	2802.69012	0.46802523	0.00123586	0.0173882
LZIC	271.241402	0.7981519	0.0012412	0.01742106
NNMT	383.609941	2.01117405	0.00124135	0.01742106
PRX	553.153283	1.63794568	0.00123963	0.01742106
SNCG	14.0055747	1.86687891	0.00124226	0.01742106
IFT57	509.323907	0.80768151	0.00124425	0.01743474
PPAP2B	2313.54713	1.48151375	0.00124621	0.01744189
PXDC1	484.426921	1.04927616	0.00124679	0.01744189
AK8	16.9643409	1.15585605	0.00124812	0.01744617
ZNF461	147.47955	0.89032578	0.00125457	0.01752218
CD48	64.2758482	1.69509866	0.00125692	0.01752637
TRIT1	205.846052	1.02344734	0.00125668	0.01752637
MRPS12	207.43249	-0.7391524	0.0012651	0.01762618
SMAD5-AS1	2.97624756	2.29281971	0.00126954	0.01767367
USP53	5881.4522	1.07871471	0.00127635	0.01775405
BATF3	14.6626612	1.85005375	0.00127975	0.01778692
LOC151475	14.7566026	2.28365735	0.00128734	0.017878
STARD5	76.9358054	1.87622851	0.00128936	0.01789152
SYT13	6.53080471	-2.1395836	0.00129998	0.01802433
AMH	104.36811	-2.042852	0.00130113	0.01802577
OMA1	277.211105	0.84855611	0.00130675	0.01808895
CUX1	3693.01544	-0.6373571	0.00131141	0.01813885
SSPN	723.469241	1.85922185	0.0013169	0.01820011
AMMECR1	176.336145	-0.7329185	0.00131935	0.01821938
N4BP2L2	2986.44771	0.61252323	0.00132867	0.01832619
SPATA9	5.88097492	2.36164314	0.00132963	0.01832619
TMTC2	82.9161844	-1.5272101	0.00133029	0.01832619
CCDC144A	342.857556	-2.1836111	0.0013353	0.01837411

CYP4X1	1206.0532	1.4790451	0.00133698	0.01837411
PRR14	332.526327	-0.726835	0.00133653	0.01837411
HIST1H3I	102.375829	-1.5673931	0.00133852	0.01837995
SLC29A1	1862.06641	1.53272458	0.00133955	0.01837995
FAM117A	252.76083	1.06305071	0.00134132	0.01838963
DHRS3	3187.08943	1.27108662	0.00134326	0.01840154
KCNK2	102.874146	-2.4079047	0.00135331	0.01852437
CD22	48.855432	-2.0150464	0.00135752	0.01856718
SLC22A4	47.9447114	1.37133686	0.00135937	0.01857765
FBXO46	283.811015	-1.0621151	0.00136261	0.01860719
KIAA1210	182.747411	2.18429064	0.00137275	0.01871363
RNF141	755.390069	0.94219926	0.00137368	0.01871363
TPT1-AS1	141.408557	0.84978208	0.00137298	0.01871363
SEZ6L2	569.938811	-1.9488075	0.00137576	0.0187272
TMEM140	180.20944	1.0890493	0.00137753	0.01873637
DPEP1	11.5999979	-2.3881286	0.00137956	0.01874917
PHACTR2	15671.5525	0.97059167	0.00138543	0.01879731
RBFADN	9.75974641	1.89671179	0.00138587	0.01879731
SCARNA16	139.899167	-1.0892116	0.00138639	0.01879731
POPDC2	30.1829606	1.24874313	0.00139862	0.0189342
RASGRF2	2030.80111	1.30832037	0.00139869	0.0189342
GOLGA8M	42.8456256	1.95363469	0.00141164	0.01909441
STARD13	1040.65172	-1.5179136	0.00141283	0.01909544
CC2D2B	11.3915146	1.60419915	0.00141791	0.01913401
PTPRM	3437.50754	0.94727867	0.00141713	0.01913401
CBX7	503.636443	0.90043663	0.00142249	0.01915064
CCNH	535.254754	0.67580699	0.00142105	0.01915064
SRGAP3	908.060117	-1.3475568	0.00142158	0.01915064
NLRP3	377.255365	1.60810064	0.00143	0.0192216
ZNF383	149.23505	0.56142509	0.00142899	0.0192216
ARHGAP5-AS	40.8410053	-1.2126579	0.00144512	0.01939449
PAICS	1391.57257	-1.0628082	0.00144459	0.01939449
ANO4	82.8656861	-2.3307418	0.00144694	0.01940199
TLR2	1064.01813	1.43712542	0.00144794	0.01940199
MTHFD2	570.18688	-1.555792	0.00145185	0.01943918
IMP4	302.271544	-0.5173006	0.00145486	0.01945188
MSH6	1167.95043	-0.7531202	0.00145507	0.01945188
ALB	12.7707844	2.13716151	0.00146465	0.01953441
CALY	10.4902844	-2.2873523	0.00146348	0.01953441
SLC6A8	1079.59827	1.39457493	0.00146364	0.01953441
GOLGA8N	135.817126	1.21111348	0.00146625	0.01954046
ZWILCH	280.078151	-1.0245607	0.00146833	0.01955307
ADRA1A	17.3851513	-2.2229752	0.00147056	0.01955876
PCBP3	277.883941	-1.9958919	0.00147202	0.01955876
XYLT1	1042.28911	-1.4154242	0.00147218	0.01955876
LOC441455	8.00888039	2.23792754	0.00147451	0.01955946
SUSD5	127.884015	1.94695076	0.00147368	0.01955946
UBQLN3	51.0947575	1.08203849	0.0014809	0.0196291
RPUSD3	173.666469	-0.7551619	0.00148517	0.01967052
ZNF626	659.279532	0.8496819	0.00149131	0.01973656

MYO7B	24.0516677	2.11674593	0.00152023	0.02010378
NIPSNAP1	680.404026	-1.1118037	0.00152652	0.02017144
PCNT	1513.83379	-0.8046168	0.00153459	0.02026243
RAB38	31.2899302	-1.9385335	0.00154489	0.02038276
GCC2	1725.90577	0.27822446	0.00154885	0.02039703
KIF18A	29.8203684	-1.5613537	0.00154953	0.02039703
VPS13D	5773.80395	0.73994148	0.00154864	0.02039703
ZC3H6	1001.56393	0.84668188	0.00155184	0.0204118
HOXC9	18.2191577	-2.3082431	0.00155821	0.0204799
BICD2	1517.42892	-0.4650648	0.00156343	0.02053268
TMEM246	1454.52638	-0.9449598	0.00157116	0.02061844
MLST8	419.885853	-0.8119297	0.00157265	0.02062228
PMVK	265.110597	-0.7989237	0.00157442	0.02062974
HIST1H3H	165.466329	-1.2465041	0.001577	0.02064781
RASGRP2	246.020796	-1.5377505	0.00158031	0.02067537
DGKD	1005.66767	-1.0953568	0.00158282	0.02067666
GINS4	45.9884129	-1.1116429	0.0015828	0.02067666
MDGA1	452.818387	1.44577218	0.00158409	0.02067745
ATP7A	878.68307	0.60677085	0.00159007	0.02073399
HTRA3	115.611529	-1.9645544	0.00159083	0.02073399
C20orf203	23.8945126	1.67202131	0.00159227	0.02073693
C1orf123	339.098564	0.67929291	0.00159466	0.02075231
METTL1	110.521558	-0.8840594	0.0015987	0.02078916
FRMD1	40.6662829	-2.2931675	0.00160686	0.02087857
IFT43	102.6274	0.78871818	0.00160833	0.02087857
RPF2	330.092652	0.73343398	0.00160923	0.02087857
CIDECP	60.6673384	-0.8729961	0.00161216	0.02090086
PKDREJ	8.07039585	2.19793322	0.00161726	0.02095111
TBC1D14	2002.44375	-0.5134443	0.00162086	0.02098191
KCNA6	134.354028	2.25921186	0.00163131	0.02108543
LOC553103	66.6447826	1.57572177	0.00163029	0.02108543
ASB9	19.9470507	-1.2824627	0.00163274	0.02108798
C4orf19	260.21719	-1.9235691	0.00163426	0.02109176
ANKRA2	331.00809	0.68854292	0.00164811	0.02125459
CYB561D2	130.909645	0.84178676	0.00166573	0.02142863
ENOSF1	606.87895	0.888267	0.00166698	0.02142863
FAM46D	9.67180719	-2.3634494	0.00166532	0.02142863
RNU12	74.7621188	0.98104957	0.00166785	0.02142863
ZNF598	504.48218	-0.9352137	0.00166365	0.02142863
LOC284454	166.860445	1.5410739	0.00167103	0.02145337
FBXW5	1028.12965	-0.5847952	0.00167952	0.02154626
PCLO	379.418928	-1.6093939	0.00169019	0.021667
KIAA0895L	506.729482	-0.8688515	0.00169386	0.02168168
PDLIM4	455.807233	1.14305266	0.00169346	0.02168168
UBE2Z	1953.94047	-0.481603	0.00169603	0.0216933
PLK4	88.5812264	-1.2616449	0.0016994	0.02171596
SEMA3A	90.9769536	-2.2390008	0.00170033	0.02171596
HIST1H2BM	74.7731647	-1.630031	0.00171408	0.02187392
NRBP1	1392.6956	-0.5950193	0.00171603	0.02187392
PPAN	36.8174755	0.93601344	0.00171653	0.02187392

TGFB3	877.302045	1.5296332	0.00171947	0.02189517
MMP24	97.9497532	-1.7218602	0.00174567	0.02221226
SLC30A3	31.3840526	2.33144292	0.00174723	0.02221567
ABCC11	5.14627126	-1.663518	0.00175106	0.02224796
MIR497HG	140.53445	1.8097588	0.00175469	0.02227758
COLCA2	57.9434186	-1.5781837	0.00176062	0.0223033
ISG15	229.526947	1.12564002	0.00175841	0.0223033
MRPL15	355.684002	-0.6988205	0.00175971	0.0223033
ALX3	129.268985	1.89454435	0.00176783	0.02234746
C18orf25	705.410681	0.78464656	0.00177089	0.02234746
PIK3R2	1126.92232	-0.7046522	0.00177271	0.02234746
SLC7A5P2	387.411109	-1.1620926	0.00177151	0.02234746
TMEM145	22.6635677	-1.9476691	0.00177322	0.02234746
ZNF483	1141.64274	1.2003137	0.00177125	0.02234746
ZSCAN18	1087.84174	1.08709938	0.00176793	0.02234746
MTRNR2L3	6.38629406	1.92188361	0.00177616	0.02236161
WDR5	612.734883	-0.6049902	0.00177694	0.02236161
DMXL1	2812.67913	0.78179718	0.00178006	0.02238445
MIR3648	141.038687	-2.2819623	0.00178294	0.02240427
TRNT1	341.594119	-0.6775007	0.00178878	0.02244627
YDJC	81.7557394	-1.0961784	0.0017889	0.02244627
CCDC86	235.156243	-0.9846193	0.00179326	0.02248454
ATP5G3	1738.5021	-0.7470066	0.00180191	0.02251152
CASC2	45.2944467	0.96569208	0.00180086	0.02251152
CYP26A1	11.7710599	-2.268612	0.00180197	0.02251152
KLHDC7A	249.154821	-2.3001673	0.00180188	0.02251152
NOL12	192.768799	0.65715334	0.00180068	0.02251152
ADM5	23.7002989	-1.5809722	0.0018098	0.02259287
TMEM8A	624.680858	-0.7398685	0.0018128	0.02261384
HIST2H2BF	408.625992	-1.1284759	0.00181485	0.022623
CAPN15	595.673267	-0.8337828	0.00181781	0.02264347
MANEAL	174.625186	-1.7606648	0.00182364	0.02266079
PHIP	4273.47166	0.70014487	0.00182111	0.02266079
PPP5D1	11.9107552	1.65724052	0.00182448	0.02266079
WBP1L	6237.28752	1.14858199	0.00182306	0.02266079
SH3GL2	33.4481035	-2.2401612	0.00182834	0.0226923
HSPD1	3471.52048	-0.7911154	0.00183062	0.02270397
RAMP3	72.5314518	1.65853324	0.00183193	0.02270397
IGIP	548.507441	0.89061304	0.0018418	0.02280994
H1F0	1087.20752	-1.0180609	0.00185702	0.02298181
ZNF467	175.011364	-1.3437564	0.00185988	0.02300064
AKT1S1	577.730136	-0.8209276	0.00186135	0.02300216
CCND3	565.787627	0.82966504	0.00186348	0.02301198
OXCT1-AS1	10.7933478	1.93867419	0.00186677	0.02303604
CNKSR3	481.436455	1.18824583	0.00187067	0.02306759
LRFN3	106.25239	-0.6709613	0.00187811	0.02313979
SS18L1	377.735792	-0.7317658	0.00187922	0.02313979
LDLRAP1	735.039113	1.25392629	0.00188272	0.02315087
LOC1019269	3.52232998	2.33419946	0.00188282	0.02315087
ANKRD52	1940.0204	-0.9352525	0.00188514	0.02316277

MYOZ3	216.823416	1.86596615	0.00188908	0.02319462
ARHGAP11A	250.559868	-1.4059606	0.00189135	0.02320583
GPR98	21.0285112	-1.8334219	0.00189588	0.02324482
FAM168B	2917.64668	-0.4738943	0.00189961	0.02326561
GIN1	165.686935	0.71009026	0.00190028	0.02326561
SNX3	1470.6351	0.63466901	0.00191116	0.02338205
EGFR-AS1	3.53965923	-1.8959241	0.00191468	0.02340844
CCDC69	131.54201	1.40398792	0.0019269	0.02354115
C6orf132	12.6005264	-2.3114981	0.00193955	0.02367879
NEIL3	21.9375522	-1.4096827	0.00194428	0.0237196
ADAM28	425.738729	1.45637306	0.00194703	0.0237363
ANKRD39	82.5393955	-0.633842	0.00194988	0.02373775
C11orf80	112.893948	-0.9771489	0.00194993	0.02373775
SNORA71C	10.1039381	-1.8841957	0.00195242	0.02373775
TNIK	301.680645	1.81349997	0.00195268	0.02373775
SAMD5	753.352076	2.21781763	0.00195476	0.02374621
TMPRSS3	3.16872455	-2.3367347	0.00195755	0.02376333
SH3BGRL	2278.6696	0.72133478	0.00196362	0.02380337
UBA7	869.551753	1.23154543	0.00196345	0.02380337
MAPK10	479.180439	1.28570429	0.00196654	0.02382193
ZNF852	107.091429	0.84069095	0.00196798	0.02382257
MGC16142	19.1928651	1.32236974	0.00197084	0.02382362
UBALD2	257.897039	-0.7006199	0.0019703	0.02382362
SLC28A1	8.10139347	2.1616262	0.0019733	0.02383647
CASKIN2	926.435967	-1.3650059	0.00198529	0.0239589
CDC37L1	253.619267	0.97923231	0.00198622	0.0239589
BST2	292.811953	1.21128721	0.00199052	0.02399385
GLDC	179.335586	-1.555167	0.00199728	0.0240585
SETDB2	428.468732	0.59536124	0.00200112	0.02408784
CHST6	7.5452823	-1.7644237	0.00200887	0.02416413
KIAA1671	5491.90052	-0.978085	0.00201254	0.02419132
RUNX2	368.127697	1.83845344	0.00202923	0.02437493
CEP135	268.464728	0.73812753	0.00203335	0.02440735
EPN1	1333.33974	-0.7001034	0.0020478	0.02456362
CNOT1	4960.34791	-0.5330523	0.00206265	0.02472438
LINC00924	87.6838434	-2.1783988	0.00207451	0.02484926
C19orf44	196.918869	1.06943921	0.0020776	0.02485152
INMT	6039.0062	2.29047259	0.00207739	0.02485152
CACNA1D	402.722824	2.1126815	0.00208416	0.02491267
ATR	1338.0322	-0.4561585	0.00208634	0.0249129
SWSAP1	40.8051496	0.94865981	0.00208708	0.0249129
AARS	2841.97073	-0.9119378	0.00208913	0.02491565
INTS6-AS1	35.3352901	0.89585559	0.00209167	0.02491565
LRRC48	50.7255488	1.57746465	0.00209082	0.02491565
BBS7	300.710476	0.65525523	0.0021019	0.02495098
EBF1	946.67931	1.93238051	0.00210169	0.02495098
GLS	2802.80877	1.04353209	0.00209776	0.02495098
PVT1	110.385797	-1.276991	0.00209769	0.02495098
UBE3C	1945.50272	-0.3856154	0.00209936	0.02495098
COL6A4P2	28.3936823	2.17892167	0.0021036	0.02495394

ST6GALNAC6	1098.5731	0.87724115	0.00210952	0.02500679
PRDM8	11.6510162	-1.939293	0.00212252	0.0251299
SNHG17	129.264675	-1.1479919	0.00212429	0.0251299
ZNF563	102.954463	1.38196685	0.00212343	0.0251299
KCTD15	552.634963	-1.0083246	0.00213128	0.02519525
TPBG	305.037937	1.09215723	0.00213381	0.02520775
XRCC2	77.5902277	-1.4217765	0.00213565	0.02521208
DBI	556.306062	-0.6273194	0.00213752	0.02521691
C4orf48	55.6614555	-1.3618402	0.00214283	0.02524486
RPTOR	1338.83402	-0.6139392	0.00214266	0.02524486
TFAP4	104.857285	-1.1782069	0.00214523	0.0252557
NBPF25P	34.2175364	1.18453755	0.00215296	0.02532934
ERMAP	285.49061	0.98228088	0.00215708	0.02534338
SLITRK6	123.111736	-2.2010578	0.0021571	0.02534338
HVCN1	181.578435	1.17118226	0.00215989	0.02535877
LINC00482	28.6124186	-1.9599555	0.00216486	0.02539598
MGAT4A	1446.76123	1.16156178	0.0021675	0.02539598
PDE3A	827.964064	1.63464726	0.00216624	0.02539598
LPIN2	1288.03502	0.99598819	0.00217349	0.02544885
MSH2	482.392743	-0.6713693	0.00218145	0.02552463
FAM200B	581.44751	0.51611362	0.0021879	0.02558259
FLJ46906	15.5370701	1.77057748	0.00219892	0.02569398
DYM	860.664124	0.64457483	0.00220421	0.0257383
AIFM2	351.726048	-0.7121389	0.00221972	0.02586662
USP33	1390.7408	0.70003407	0.00221868	0.02586662
VSIG10	348.898276	-0.9725678	0.00221857	0.02586662
SVEP1	2438.44533	2.15137922	0.00222757	0.0259405
KRT77	4.085993	-2.3068073	0.00223381	0.02597789
RAB6C	10.7675914	-1.5524791	0.00223375	0.02597789
PEX6	461.457982	1.04115247	0.00223897	0.02601573
SYT12	14.1424868	-2.0374528	0.00224137	0.02601573
TSPAN17	435.818983	-0.9193898	0.00224161	0.02601573
HDDC3	137.6595	-0.7656883	0.00224364	0.02602172
AGBL3	29.3883431	1.13879267	0.00224958	0.02603103
CNTNAP3	621.028277	-1.6557585	0.00225051	0.02603103
FAM64A	15.2264474	-1.6789353	0.00224954	0.02603103
IPO5P1	435.821779	1.02487849	0.00225031	0.02603103
CPNE5	95.6677313	1.99367537	0.00225728	0.02609171
CCDC138	73.9127432	-0.5774792	0.00226303	0.02614056
ATP6V1B1	27.2981235	-2.0595787	0.00226827	0.0261659
ZBTB37	497.227017	-0.5592586	0.00226811	0.0261659
KIAA1683	193.335389	1.31557697	0.00227975	0.02628066
GRPEL1	320.813641	-0.6581938	0.00228474	0.02632051
KANK1	885.014032	1.27561841	0.00229197	0.02637684
PTGER3	6.90526532	1.91933055	0.0022927	0.02637684
SLITRK2	11.3109762	-2.2757585	0.00229508	0.02638648
KIRREL3	4.82672072	-2.1735542	0.00229878	0.02641139
C15orf27	7.50547587	-1.8465818	0.00230478	0.02645727
FHOD1	776.051157	-1.089511	0.00230586	0.02645727
IFT81	299.923009	0.60856935	0.00231709	0.0265331

PDGFD	3156.2016	1.65724122	0.00231696	0.0265331
PHACTR1	201.472804	1.70122327	0.00231711	0.0265331
FH	639.949501	-0.6753713	0.00232258	0.02657733
POLR2D	468.423871	-0.4943714	0.00232406	0.02657733
CEACAM4	10.403475	1.65952401	0.00235067	0.02686369
ELP2	1234.85893	0.72694637	0.00235363	0.02687957
SLC25A39	1067.18085	-0.5633387	0.00236066	0.02694197
ZNF317	683.117942	0.59954327	0.00236632	0.02698859
LOC1005073[illegible]	3.18088161	-2.2356352	0.00237075	0.02702124
HIC1	713.997397	-1.0488258	0.00237396	0.02703987
UBE2S	221.664508	-1.1287797	0.00237853	0.02707387
C11orf21	18.2819229	1.723905	0.00240997	0.02739543
GAS2L1	226.95251	-0.6765103	0.00240907	0.02739543
LGI4	129.714652	1.28664855	0.00241567	0.02744209
RTKN	340.108024	-0.9052746	0.00241833	0.02745417
ZNF780A	676.609415	0.70440351	0.00242146	0.02747152
ASIC4	5.73547159	-2.0473093	0.00244386	0.0276643
PPP1R12B	2820.14057	0.82207233	0.00244402	0.0276643
SIAE	782.419322	-1.1490476	0.00244082	0.0276643
XPOT	2103.59205	-0.8276514	0.0024449	0.0276643
INPP4B	174.572546	1.1141902	0.00245153	0.02772106
STRADA	607.466999	-0.5826338	0.00245501	0.02774208
LOC1019270[illegible]	5.4688666	2.11789512	0.00246	0.02777048
SLC7A5	522.395957	-1.8423006	0.00246198	0.02777048
ZNF699	117.556287	0.92135152	0.00246238	0.02777048
LOC1002889[illegible]	44.2998711	1.41331866	0.00246623	0.02779572
CYP4F35P	93.1341738	2.27658624	0.0024679	0.02779626
LOC80154	18.2344592	-1.3162345	0.00247186	0.02782263
ABCG4	27.4083914	-1.8459429	0.00247445	0.02782814
FLYWCH1	970.298389	-0.5388438	0.00247559	0.02782814
VPS4A	1178.91948	-0.5227625	0.00248368	0.02790078
PI16	162.197352	2.25894177	0.00249929	0.02805776
B9D1	101.488171	0.90952674	0.00251138	0.02817504
PRUNE2	139.367841	-1.6484438	0.00251416	0.02818784
RPL7L1	630.540234	0.62640303	0.00251929	0.02822694
SOCS1	27.7106868	-1.8197845	0.0025299	0.02832732
LOC1019282[illegible]	8.37696555	-2.2437666	0.0025316	0.02832787
PIGW	161.076146	-0.8311094	0.00253546	0.02835261
LYRM5	272.291057	0.7780649	0.00253716	0.02835312
CLEC17A	17.0755281	2.10527402	0.0025513	0.02849256
TRIM59	104.285691	-1.4915594	0.00255907	0.02856079
EFHC1	621.460441	0.84114958	0.00256659	0.02862611
ZNF865	297.538663	-1.054857	0.00257296	0.02867849
ECHDC3	20.682522	1.75190217	0.00260729	0.02904227
BMP1	1444.4165	-0.9636938	0.0026341	0.02912831
C14orf80	51.5381201	-0.9517562	0.00263229	0.02912831
C7orf26	256.251098	0.52554711	0.00262359	0.02912831
CELF6	188.39072	1.28035862	0.00263063	0.02912831
DKK2	971.753515	-2.2385058	0.00262703	0.02912831
FILIP1L	401.501959	1.28244023	0.00263538	0.02912831

IQCB1	332.698005	0.62879103	0.00262556	0.02912831
LOC1001314	8.10775353	1.88770947	0.00262319	0.02912831
LOC284023	69.7033385	0.9981956	0.00263256	0.02912831
NUP62	956.34253	-0.3875187	0.00261888	0.02912831
PTRH2	164.272518	-0.9115543	0.00262318	0.02912831
TLE2	1579.92989	1.22272486	0.00262285	0.02912831
HSD3B7	207.873995	-0.754137	0.00263927	0.02915254
CEP95	635.981727	-0.6787631	0.00267079	0.02948177
C16orf80	268.822049	-0.605006	0.00267865	0.02954948
SLITRK5	6.45844416	-2.0323308	0.0026888	0.02964241
PGBD4	70.8775149	-0.764577	0.00269194	0.02965799
CXorf30	16.2048557	-2.2448123	0.00269396	0.02966124
KLHL6	194.884969	1.42870633	0.0026978	0.02967356
LINC01279	507.128826	1.56427215	0.00270021	0.02967356
TBX1	15.6004767	-1.8923872	0.00270027	0.02967356
ZYG11B	1650.3326	0.7238526	0.00270408	0.02969641
FOXP3	4.36980564	-1.7370081	0.00271164	0.02976042
IL18	634.034858	-0.9102616	0.00271391	0.02976178
KANSL1L	1056.9378	0.92679548	0.00271575	0.02976178
YAF2	754.190019	0.76165758	0.00271697	0.02976178
SPRED1	1874.73876	0.52033233	0.00272041	0.02978055
GTF2IRD1	714.480897	-0.9446843	0.00272245	0.0297839
GUCY1B2	9.22127151	-2.2306617	0.00273555	0.02990807
BRE-AS1	27.3683528	1.8095816	0.00274658	0.03000961
OXLD1	103.244284	-0.5406022	0.00275274	0.03005771
UBFD1	1725.84616	-0.5059559	0.00275764	0.03009208
PSAPL1	35.1864233	-2.1773792	0.00276415	0.03014397
FLYWCH2	416.410859	-0.8182537	0.00276809	0.0301678
LINC00649	17.1048066	2.09563293	0.00277686	0.03022611
SPOP	1115.7336	0.57903286	0.00277696	0.03022611
GOPC	981.931201	0.7071263	0.00279119	0.03034251
KIAA1217	4459.2736	-0.8931673	0.00278978	0.03034251
FLJ44087	20.9248549	1.85475741	0.00279781	0.03039521
TOMM40L	233.406411	-0.8850135	0.00281627	0.03055703
TRAPPC2P1	60.9524381	0.87142056	0.00281577	0.03055703
SERPINE2	319.120369	-1.4838467	0.00282104	0.03058943
GTF3C4	626.887436	-0.5212839	0.00283938	0.03076892
LOC728819	5.38740955	-1.9859064	0.00284615	0.03078711
SGK2	44.7542483	1.58068277	0.00284644	0.03078711
SPOPL	1083.20439	1.05369952	0.00284422	0.03078711
ZNF92	265.127591	-0.4800261	0.00285171	0.0308247
TMEM127	1434.95239	-0.3940036	0.00287918	0.031102
CEP57L1	202.982479	1.16681374	0.0028818	0.03111074
ARHGAP11B	37.5565646	-1.5538388	0.00288698	0.03114707
NPIPB5	97.6179968	-1.0755123	0.00289101	0.0311588
PLCD3	2516.06217	-0.7896383	0.0028917	0.0311588
PTPN9	1561.63929	-0.8772841	0.00290426	0.03127451
BCORL1	431.188534	-1.1173065	0.00291956	0.0314037
PLD4	244.614243	1.71729877	0.0029206	0.0314037
PLEKHG2	1521.60421	-0.8719122	0.00292174	0.0314037

HECW2	510.993587	1.67470575	0.00293644	0.03152219
MAL	5.58643138	-2.2415046	0.0029357	0.03152219
SKINTL	12.5098149	1.58898557	0.00293839	0.0315234
CNN3	1946.36516	0.64357193	0.00294091	0.03153071
CABYR	17.2545992	-1.4840561	0.00294555	0.03156073
MCM9	406.378184	0.83597208	0.00295022	0.0315911
ZIC4	353.318379	-1.4340981	0.00295705	0.03164451
ELAVL2	7.00070138	1.79317064	0.00296863	0.0317486
ARHGAP23	2334.15158	-0.6891403	0.00297048	0.03174865
CXCR3	3.73398302	1.97557066	0.00297243	0.03174972
C10orf10	326.467562	1.52702163	0.00297756	0.03178466
SULF2	12251.6404	1.12218386	0.00297958	0.03178647
BHMT	29.6301781	2.02894476	0.00298506	0.03181551
DPF1	4.66312355	-1.9908934	0.00298786	0.03181551
SAMD4A	5484.72354	1.42868595	0.00298647	0.03181551
AGAP2-AS1	34.7799133	-1.5218028	0.00299218	0.03184177
TSSC4	261.846306	0.62365705	0.00299969	0.03190195
CDK5	89.6437799	-0.8598243	0.00300432	0.03193132
CDC40	564.143543	0.73144953	0.00300675	0.03193744
HNRNPUL2	602.587041	-0.5771393	0.00301328	0.031987
ALKBH3	240.849193	0.55163155	0.00301919	0.03202986
PIK3IP1	771.311773	1.3391692	0.00303552	0.03217628
VAV2	608.345885	-0.961902	0.00303674	0.03217628
ECM1	77.4429685	-1.4668723	0.00304113	0.03218313
PSMD3	1309.90554	-0.5565736	0.00303952	0.03218313
UBXN7	968.380507	-0.4073598	0.00304868	0.03224312
IRF4	18.3652543	1.39533644	0.00305133	0.03225131
HTRA1	1853.75891	1.31171851	0.00307628	0.03247501
ZMYND15	112.62855	1.40587389	0.00307579	0.03247501
PPP1R1C	7.68977215	-2.1400877	0.00307887	0.03248234
JAM2	11206.6255	1.44161607	0.00308586	0.0325117
LOC1005064	9.45037946	2.2017099	0.00308923	0.0325117
NR4A3	297.675063	1.9593387	0.00308734	0.0325117
SQRDL	669.381428	0.91723628	0.00308884	0.0325117
DNASE1L1	856.695277	-0.5893268	0.00310852	0.0326835
ITPKB-IT1	45.551863	1.05962862	0.00310936	0.0326835
ATXN1	3059.14247	0.75874907	0.00311838	0.03275831
FBXO43	9.97592291	-1.6382124	0.00312881	0.03284771
FBXL17	770.537304	0.76777469	0.00313605	0.03290364
BRD8	1065.22849	0.6275343	0.00314287	0.03291777
CAPN11	8.89213929	1.66030271	0.00314315	0.03291777
CTTNBP2	202.042694	1.81996252	0.00313973	0.03291777
FAM3B	11.7808148	-1.5665217	0.00314832	0.03293761
NDUFA10	988.718609	-0.5720463	0.00314889	0.03293761
SGCE	325.000852	-1.7066447	0.00315103	0.03293995
AGAP7	21.8353381	1.91922691	0.00315836	0.03297642
PLCB1	1204.2541	1.7986061	0.00315809	0.03297642
GATSL2	433.089985	-0.7931639	0.00316029	0.03297648
CCNB1	169.743903	-1.332297	0.00316632	0.0330194
CCDC173	7.16817592	1.51809253	0.0031686	0.03302312

LOC1019277	11.2567284	2.15955449	0.00317062	0.03302414
ACOT7	131.250765	-0.8565235	0.00317911	0.03307665
CCDC28B	34.83675	-0.7602251	0.00317952	0.03307665
ZNF333	436.357303	0.65533731	0.00318821	0.03314699
P4HB	8102.18497	-0.656894	0.00319318	0.03317848
COX10	223.447136	-0.6940084	0.00319768	0.03320517
ICT1	167.615082	-0.5547789	0.00320898	0.03330235
NAA35	431.049041	-0.4752047	0.00321725	0.03336808
IGSF9	72.9243955	-1.6547553	0.00322132	0.0333882
ZBTB40	1197.91602	0.60060505	0.00322308	0.0333882
ADRA1D	47.0123034	-2.2222005	0.00322835	0.03341237
TMSB10	9563.38218	-0.6862328	0.00322931	0.03341237
HOXA10	13.466388	-2.1785338	0.00323644	0.03346598
ARHGAP39	179.145835	-0.8979501	0.00324336	0.03351733
CLN6	471.400778	-0.9462971	0.00325089	0.03353456
OMG	3.62716979	-2.1362132	0.00324897	0.03353456
SH2D5	2.6379253	-1.9990579	0.00324741	0.03353456
CWF19L2	536.221555	0.49689868	0.00326332	0.03364259
TTYH3	1302.16912	-1.1148581	0.00326996	0.03369078
ZNF790	179.599959	0.91393891	0.00327495	0.03372198
ZNF570	167.595273	0.79038363	0.00328024	0.03375621
TRIM34	9.88774366	1.26376214	0.00328495	0.0337844
ANKRD12	2654.2596	0.67000308	0.00329048	0.03379695
LANCL2	335.874484	0.91996958	0.00329154	0.03379695
SETD1A	764.348102	-0.7953112	0.00329208	0.03379695
PCOLCE-AS1	53.4071483	-1.5260806	0.00331221	0.03398329
EIF3L	2962.5386	0.73848238	0.00332319	0.03407563
FAM193A	1086.89607	-0.5423206	0.00332862	0.0341109
CRIPT	685.351879	0.61474065	0.00333901	0.03417658
POLE2	28.3302203	-1.1597844	0.00333731	0.03417658
ARRDC3	3943.74734	1.4993014	0.00334339	0.03420097
GRAP	43.2620164	1.20165407	0.00335355	0.03426888
ZNF683	3.49856481	2.08451443	0.00335402	0.03426888
DNAH1	1314.94222	0.87274393	0.00335644	0.0342732
AMMECR1L	624.136662	-0.5269141	0.0033623	0.03429248
ZDHHC5	1861.59247	-0.5246145	0.00336232	0.03429248
PLEKHB1	60.9126636	-1.6646	0.00336558	0.03430533
TATDN1	144.003179	-0.5157176	0.00337364	0.03436708
BACH2	171.15765	1.26558268	0.00337587	0.03436935
ATG4B	780.806814	-0.6534321	0.00338649	0.03443668
ZNF98	19.8905506	1.96203235	0.0033862	0.03443668
DDX3X	6830.4636	0.50700198	0.0033942	0.03447422
FKBP11	170.719731	-0.8750328	0.00339322	0.03447422
HLF	4432.03454	1.16894727	0.00339848	0.03447694
STRA13	157.436413	-0.7513179	0.00339836	0.03447694
NS3BP	93.6033922	1.01946589	0.00340821	0.03455516
RGS4	79.459636	-1.9995343	0.00341898	0.03464388
DLGAP3	14.0346719	-1.1511147	0.0034269	0.03469705
HDC	13.7689203	2.02540466	0.00343099	0.03469705
LAMA1	1020.64707	-1.9198952	0.00343433	0.03469705

SH2B2	16.078944	-1.5034074	0.00343324	0.03469705
TXK	62.4823468	1.67300261	0.00343206	0.03469705
SSBP2	1204.60617	1.16182746	0.00343711	0.03470473
C20orf194	1481.06887	0.71335669	0.00344003	0.03471379
NPR1	40.018206	1.4444875	0.00344906	0.03477488
TCP11	1.90789901	2.19517296	0.00345014	0.03477488
CELF5	7.07496766	-2.0492878	0.00345463	0.03477928
PID1	442.443753	1.85208177	0.00345295	0.03477928
CLK2	837.271851	-0.7422949	0.00345735	0.03478631
B4GALT3	367.580251	-0.686504	0.00346156	0.03480574
NRTN	5.87377629	-2.1383976	0.00346334	0.03480574
FOSB	1731.02116	1.951142	0.00347191	0.03487147
EPHA3	144.490435	-1.7017574	0.00347838	0.03491597
LPAR1	1126.66865	-0.851735	0.00348578	0.03495502
NOP14	724.742903	-0.526919	0.00348634	0.03495502
DUOX1	265.837448	2.17163537	0.00349513	0.03502271
SLC12A8	17.4713755	-1.2700377	0.00350678	0.035119
ANGPT1	66.5151633	1.88728614	0.00351928	0.03522357
PALB2	324.168775	-0.3810841	0.00352526	0.0352629
KCNS2	3.10866281	-2.1957787	0.00352766	0.03526631
CDC45	35.4211497	-1.9012009	0.00355011	0.03546772
ENKD1	174.809903	1.0066853	0.00355381	0.03546772
PBX2	12.5627942	1.41616003	0.003554	0.03546772
CALML3-AS1	36.1948565	-1.9452597	0.00355769	0.03548384
MST1P2	35.0872945	-1.6806911	0.0035753	0.03563878
ELOVL6	297.159586	-1.0998733	0.00358597	0.03572441
ACSM5	61.6258427	1.43799747	0.00360542	0.03589733
COQ7	404.555662	0.41333173	0.00361051	0.03590733
GINS1	118.288493	-1.2995917	0.00361061	0.03590733
GPN1	371.757219	-0.4579104	0.00361549	0.03593503
CCNE2	44.0237407	-1.3728496	0.00362062	0.03596321
SCP2	1382.84572	0.73142934	0.00362251	0.03596321
IGF2BP2	583.631992	-2.0478642	0.00362465	0.03596366
UBALD1	208.924472	-1.1329715	0.00362765	0.03597259
BOP1	215.742917	-0.7810554	0.0036439	0.03611294
NME4	1020.00106	-0.9943617	0.00364799	0.03613261
TACC1	9448.3341	1.17917313	0.00365358	0.03616709
OR56B1	17.1368906	1.66773431	0.00366255	0.03621429
RSAD2	178.534989	1.32504547	0.00366257	0.03621429
TRPC6	49.7332448	1.64736699	0.00366987	0.03626554
ABHD6	250.415815	1.11308182	0.00367623	0.03630757
PSMC5	1025.04058	-0.6486327	0.00368101	0.03633382
LPPR4	74.0959753	1.85971043	0.00371019	0.03660082
ART5	17.7399535	1.90279493	0.00371501	0.03662733
EVPL	7.14828727	-1.8291975	0.00372303	0.0366853
AGL	2440.20877	1.63153338	0.00373257	0.03671604
METTL15	286.149225	0.71473696	0.00373097	0.03671604
SNORA37	20.5534387	1.1437247	0.00373187	0.03671604
IGLL5	20.9359594	-2.0731731	0.00374679	0.03682272
MCOLN3	18.5983689	1.86987994	0.00374826	0.03682272

RBMS3-AS3	2.9400584	2.08062711	0.00374985	0.03682272
IRF6	8.35319556	1.62721311	0.00376209	0.03690913
RASGRF2-AS	3.97044565	2.1343417	0.00376295	0.03690913
C21orf58	104.943208	-1.0369664	0.00378057	0.03706077
KIAA0100	6103.15338	-0.3842984	0.00378535	0.03706533
LOC1005055	83.9040428	1.05469506	0.00378527	0.03706533
MGRN1	1253.76389	-0.6093896	0.00380182	0.03720541
CASC9	9.98535833	-2.1725536	0.00382614	0.03738863
MTMR4	1829.45308	-0.6470389	0.00382708	0.03738863
PDPR	2258.14585	-0.8636432	0.00382512	0.03738863
CENPO	260.930604	-0.8667698	0.00383787	0.03747272
CDK19	547.338553	0.72011962	0.00385963	0.0375996
DNAJC4	357.027522	-0.779287	0.00385634	0.0375996
PRKCH	872.907512	1.0852631	0.00385383	0.0375996
TERF2	467.844087	-0.6453162	0.00385789	0.0375996
NPHP3	256.505647	0.65594043	0.00386491	0.03761242
PFKM	2900.09114	-0.5706896	0.00386533	0.03761242
LRRC37A3	293.36485	1.16067411	0.00386843	0.03762135
UQCRHL	31.312598	0.93341598	0.00387263	0.03764082
STAT5B	1507.1416	0.62965462	0.00387937	0.03768503
TNRC6C	2277.39854	-0.7345731	0.00388565	0.03772468
CECR7	100.123495	-2.0533805	0.00389395	0.03776253
COLGALT1	1683.86294	-0.6740655	0.00389256	0.03776253
ZXDA	289.718498	0.60923545	0.00390525	0.03785069
C6orf89	2705.28231	0.62622378	0.00391368	0.03791102
FLJ33360	12.1843327	1.38012287	0.0039273	0.03794066
HDHD2	714.656605	0.87179563	0.00392518	0.03794066
HIPK3	4633.85838	0.62797719	0.00392491	0.03794066
LINC00925	10.3893705	1.75810244	0.00392453	0.03794066
NKD1	1645.70691	2.09785382	0.00392919	0.03794066
SH3BGRL2	512.741829	1.51867319	0.00393	0.03794066
MED14OS	4.30088293	1.78334835	0.00393792	0.03799575
UBE2J1	989.985124	0.6062113	0.00394418	0.03803471
WEE2-AS1	18.8206728	1.41381625	0.00395604	0.03810634
ZNF607	218.794196	0.75201209	0.0039558	0.03810634
ECI1	471.759283	-0.8269462	0.0039605	0.03812786
ECT2L	6.58555597	1.50231516	0.00396564	0.03815595
VSTM2L	55.1833869	-1.9370156	0.00398604	0.03833071
CCDC148	55.3027131	1.6008758	0.0039967	0.03841173
ABCA13	7.44385764	-1.9954982	0.00400794	0.03847666
COLGALT2	21.896129	-1.7832415	0.00400656	0.03847666
ZNF615	361.185038	0.75031859	0.00401471	0.03852011
BRPF1	449.585829	-0.5977531	0.00402058	0.03853339
WRNIP1	837.918466	0.73117838	0.00401841	0.03853339
GPC1	1561.01058	-1.2921923	0.00404159	0.03869032
MORC2	710.491776	-0.8507482	0.00404372	0.03869032
PCYT1A	576.968493	-0.4709115	0.0040395	0.03869032
SNX13	1690.38096	0.64016107	0.0040467	0.03869723
RCAN2	209.481177	1.45848421	0.00405973	0.03878737
SV2B	32.0561246	-2.0422568	0.00406064	0.03878737

TLK1	1355.49967	-0.5722372	0.0040697	0.0388523
RAP2C-AS1	91.3740034	1.02636113	0.00407762	0.03888461
RPL13A	2172.16089	0.70574368	0.0040757	0.03888461
CDAN1	374.469454	0.47460411	0.00409777	0.03899551
MORC3	1328.03354	0.46842243	0.004099	0.03899551
MYCT1	43.3789149	1.03686494	0.00410012	0.03899551
PREX2	658.030261	1.57637392	0.00410061	0.03899551
ZNF737	1048.47503	1.00549817	0.00409798	0.03899551
SMAGP	186.550631	-1.7197585	0.00410558	0.03902121
FRMD3	75.6829353	1.32414739	0.00410939	0.03903579
DFFA	361.994441	0.45171826	0.00412868	0.03919738
BBOX1	2.10920626	2.09723632	0.00413514	0.03923695
ATG9A	1049.64397	-0.6824348	0.00416881	0.03953458
SLC6A10P	4.94057111	-2.132689	0.00417413	0.03956316
RELN	594.213147	-2.1615808	0.00417747	0.03957301
CUBN	611.039141	0.93458816	0.00419891	0.03975418
SNORA71A	58.9572636	-1.4807612	0.00420955	0.03979703
SUCLG1	748.629125	-0.5446801	0.00420728	0.03979703
USP18	78.5411983	-0.9414419	0.00421039	0.03979703
BCAS4	162.424893	-1.693129	0.00423295	0.03998825
FDPS	945.379083	-0.6756711	0.00423972	0.04000815
STRN4	1187.11573	-0.4347182	0.00423873	0.04000815
CAHM	15.9756763	1.42148834	0.0042434	0.04002082
HIST1H4A	125.369009	-1.0317319	0.00424812	0.04002138
RSPH4A	19.5302361	1.16460069	0.00424661	0.04002138
HOXC11	2.15767463	-2.1320153	0.00427672	0.04024035
IGJ	14.83067	-1.8041293	0.00427637	0.04024035
SNAPC4	319.604224	-0.5466595	0.00427839	0.04024035
RPIA	165.056633	-0.7002634	0.00428098	0.04024258
FAM221A	110.209197	1.1224071	0.00429104	0.04029304
MLXIPL	79.8380697	1.63143891	0.00428976	0.04029304
FRMPD4	2.02325919	-2.149212	0.00430433	0.04039575
BEND4	3.70234263	-2.0497439	0.00430913	0.04041872
TBP	209.990861	0.43980862	0.00431883	0.04048753
CHL1	13.8471059	-2.1224438	0.00432362	0.04051034
ICK	809.35967	1.05515498	0.00432707	0.04051543
SLC6A16	23.3523035	-1.3300787	0.00432888	0.04051543
APOB	204.832343	1.477353	0.00435365	0.04060846
C16orf70	323.925678	-0.4466553	0.00434606	0.04060846
CENPK	63.7100189	-1.5854161	0.0043514	0.04060846
ENTPD8	19.7925913	1.91218362	0.00435724	0.04060846
PUS7	287.93443	-0.6990053	0.00435775	0.04060846
SLC45A4	483.63168	0.86818563	0.00434454	0.04060846
TMEM63C	3.23220188	-1.8864711	0.00434792	0.04060846
ZC3H12D	100.344828	1.27233658	0.00435624	0.04060846
KIN	341.113273	0.41113963	0.00437313	0.04072967
BNIP1	86.5581503	0.6446495	0.00437918	0.04073611
FAM129B	2799.62534	-1.0638562	0.00438061	0.04073611
FNIP2	1264.21727	1.08895696	0.00438094	0.04073611
ANKRD20A9I	85.407314	2.08254715	0.0043843	0.04074526

FAM86C1	58.5468052	-0.7397923	0.00439076	0.04078319
ENPP4	913.268383	1.03003786	0.00440367	0.04088097
SAMD11	358.070147	1.68650104	0.00442381	0.04104429
SERPINH1	3217.18584	-1.105236	0.00442604	0.04104429
FTH1	12689.5344	-0.9829089	0.00443274	0.04108418
C9orf89	111.506118	-0.7926037	0.00443978	0.04112728
ZNF426	475.322463	0.69120621	0.00445587	0.04125408
CYP2D7P	24.7024148	1.1748323	0.0044693	0.04135609
GUCA1B	29.8848257	1.17862145	0.00447998	0.04143255
CSNK1G3	1683.21948	0.84106155	0.00448403	0.04144771
CERCAM	289.160169	-1.208453	0.00449704	0.04150036
E2F1	236.084812	-1.3763223	0.0045016	0.04150036
ME3	173.078291	1.01936469	0.00449671	0.04150036
MSTN	24.588975	1.45755577	0.00450182	0.04150036
VPS41	2476.06863	0.79989541	0.00449746	0.04150036
FAM155B	17.310087	-1.4555864	0.00451472	0.04153009
FLAD1	357.716954	-0.5496479	0.00451015	0.04153009
IFT88	342.866835	0.8950582	0.00451157	0.04153009
RPS8	8080.21016	0.60498982	0.00451429	0.04153009
WDR76	136.760893	-1.2040939	0.00452347	0.04158828
SCN4A	24.9909335	1.78858786	0.00452633	0.04159232
RPS6	10647.1058	0.52620584	0.00452909	0.04159541
FOXS1	64.4122473	-1.838797	0.00453658	0.04164194
EPB41L4A-AS	185.743573	0.76536956	0.00455108	0.04173988
PER1	3393.91828	0.984313	0.00455212	0.04173988
AC093375.1	18.4861432	-2.0520019	0.0045606	0.04179533
HIST1H2BH	187.680421	-1.625691	0.00457785	0.04193103
CAMK4	14.920853	-1.5812273	0.00460341	0.04214272
ITGB3	166.616482	1.39977952	0.00460714	0.04215433
FLJ31104	10.2769687	1.46895518	0.00461709	0.04222293
BLOC1S6	1222.66218	0.5466414	0.00463958	0.04240592
DUSP23	144.940047	-0.8191707	0.00465008	0.04245669
VCAM1	1212.85921	1.7664618	0.00464766	0.04245669
COL8A2	1890.26693	1.71034058	0.00466326	0.0425469
ZNF812	150.898431	1.86288774	0.00466491	0.0425469
CCR6	10.318635	1.35506242	0.00467849	0.04264808
TTC23L	10.9074329	1.33158774	0.00468661	0.04269939
NUFIP2	5453.88287	-0.4568484	0.00469029	0.04271021
ZEB1	2215.55912	0.85414371	0.00469463	0.04272714
RSPO3	532.871308	2.08622413	0.00471361	0.0428771
BCAT1	3307.18267	-1.6926873	0.00474159	0.04308594
LRMP	114.542622	1.23207668	0.0047392	0.04308594
MATR3	6676.38399	0.39128681	0.00476173	0.0432461
TGFBR3L	2.87226701	-2.0873565	0.00477096	0.04330696
C11orf58	1883.93867	0.57027242	0.00478249	0.04336795
MRAS	2829.41971	-0.877302	0.00478273	0.04336795
AQP5	36.6768939	-2.1002321	0.00480616	0.04355738
FOS	7655.23027	1.38186786	0.00481881	0.04360297
LRRC49	288.12321	0.91692206	0.004817	0.04360297
MACC1	73.132157	1.41332896	0.00481683	0.04360297

TAOK1	5276.08436	-0.2744031	0.00483014	0.04365944
ZNF862	844.418341	-0.6913582	0.0048285	0.04365944
PJA2	4814.54098	0.82097103	0.00484125	0.04373691
ULK4P3	5.31658967	-1.7727836	0.00486706	0.04394696
BAI3	66.2352117	1.81026726	0.0048732	0.04395432
IFI44L	325.568142	1.24501269	0.00487556	0.04395432
ORC1	22.346957	-1.250644	0.00487165	0.04395432
LOC1001295	21.5243481	1.2030983	0.00488979	0.04405947
CYSLTR2	15.0410136	1.70165159	0.00492493	0.04435283
LRRC37A4P	438.766255	-0.9877271	0.00493193	0.04439256
CNOT11	609.067359	-0.4746053	0.00493934	0.04443591
GCSH	138.233839	-0.8936798	0.00495165	0.04452334
LOC401320	437.574065	0.8501202	0.0049679	0.04464605
MXD3	119.22131	-0.7404107	0.00497094	0.04465005
TLR5	181.192904	1.30033317	0.00499744	0.04486453
GID4	327.311861	0.50628466	0.00500147	0.04487731
VTA1	567.277195	0.53845999	0.0050071	0.04490435
DRD2	42.128529	2.10371561	0.0050149	0.04495078
HMGB3	150.75317	-0.9045823	0.00502649	0.04503115
POMGNT2	430.532539	0.84048439	0.00503006	0.0450396
C16orf89	1345.76007	1.53179658	0.00503496	0.04506002
HIST1H2BD	671.454582	-0.9210374	0.00505025	0.04507929
PCYOX1L	205.853665	-1.0853117	0.0050479	0.04507929
PSIP1	1630.12949	-1.0916292	0.00503976	0.04507929
SOX11	309.266008	-2.0842854	0.00504455	0.04507929
ZNF490	521.398521	0.74099267	0.00504759	0.04507929
H6PD	2086.79148	0.87753187	0.00505694	0.04511558
CMPK2	129.806789	0.86814583	0.00507007	0.04518575
MYO1E	919.81966	1.07886443	0.00506802	0.04518575
RPL38	3369.66431	-0.5300683	0.00509086	0.0453475
PCMTD1	1926.58418	0.71596902	0.00509902	0.04539662
SEPT9	2163.81629	-0.8605254	0.00510397	0.04541717
EPT1	808.773575	-0.6586355	0.00511315	0.04547527
TRAF4	678.940193	-1.0316107	0.00511789	0.04549384
RHBDD2	1476.50995	1.03667019	0.00513069	0.04558404
GINS2	59.9129096	-1.5745325	0.00514111	0.04565294
ENDOG	116.372946	-1.1786238	0.0051456	0.04566915
ZNF552	115.542186	-0.5135102	0.00515026	0.04568696
BCL9	660.7233	-0.7949513	0.00517057	0.04584342
EFTUD1P1	37.5016689	1.80569751	0.00518125	0.04591435
EML1	353.349714	1.08220993	0.00520536	0.04600522
MYO10	2264.79294	-1.2539026	0.00519971	0.04600522
N4BP3	223.272259	-1.4051061	0.00520539	0.04600522
RAPSN	4.91728753	1.89729199	0.00520758	0.04600522
SLC29A2	19.1215194	-1.8227101	0.0051977	0.04600522
SURF4	3919.96777	-0.5827079	0.00520152	0.04600522
HAS2	2.37117907	-1.9164875	0.00521391	0.04603747
CHMP6	312.042991	-0.4657958	0.00521827	0.04605222
GALNT1	2228.38408	0.79213105	0.00522359	0.04607548
PFDN2	211.856458	-0.7771034	0.0052277	0.04608785

PYCRL	134.519624	-0.7628226	0.00523036	0.04608785
LBH	1043.06307	1.273497	0.00524827	0.04622189
ANKRD62	129.430409	2.09780434	0.00526598	0.04633661
PDSS1	48.3351773	-0.7610152	0.00526669	0.04633661
TMEM133	86.6444093	-1.4479397	0.00526947	0.04633729
ELP4	292.373877	0.59109536	0.00527287	0.04634344
C2orf61	7.53932057	1.84545007	0.00528028	0.04636722
EPSTI1	192.519906	0.68815383	0.00528368	0.04636722
SUPT3H	135.066408	0.94837299	0.00528123	0.04636722
DAP3	1032.35425	-0.5276989	0.00528811	0.04638241
RICTOR	1578.958	0.60937959	0.00529188	0.04639178
LRRC36	74.3459133	-1.0423695	0.0053014	0.0464515
SELK	282.028073	0.74465811	0.00531778	0.04657128
BEND6	87.2142187	1.49227828	0.00532377	0.04657619
SCUBE1	860.866124	1.95142737	0.00532288	0.04657619
DENND6A	967.72952	0.67419284	0.00533479	0.04658241
HOXA5	6.24651894	-2.1006427	0.00532941	0.04658241
SLC38A6	398.291944	0.79545165	0.00533534	0.04658241
SUMO4	39.4745625	0.96770439	0.00533173	0.04658241
ALKBH2	142.930538	-0.7589414	0.00534164	0.04661378
RNF26	467.982122	-0.7156217	0.00535722	0.04672596
PHF12	961.424239	-0.5654783	0.00536476	0.04676793
BCHE	26.861545	-1.9783887	0.00537053	0.04679451
LEPREL4	566.214942	-1.1581335	0.00539138	0.04692856
SPAG9	8288.69792	0.52138617	0.00538994	0.04692856
HOXB13	29.4208686	-2.0962844	0.00539563	0.04694174
COL12A1	2319.94561	1.6923512	0.00541758	0.04710882
CA4	12.7355678	2.0291102	0.00544077	0.04728653
THAP3	113.409582	0.74446481	0.0054518	0.04735838
GRIA2	2.95661982	-2.0721533	0.00545626	0.04737315
PLP2	800.076482	-1.0672174	0.00546088	0.04738928
PLSCR1	376.45402	0.66309521	0.00548139	0.04751921
ZFAND5	3985.99769	0.92369751	0.00547906	0.04751921
FBXO44	335.926385	0.59333738	0.0054875	0.0475349
NRG2	13.4443537	1.5547485	0.00548874	0.0475349
PSMD1	1717.8568	-0.4642026	0.00549913	0.04760086
PPFIA3	195.13519	-1.3815763	0.00550809	0.04763041
SMAD2	3599.95751	0.69848926	0.00550649	0.04763041
LINC00189	90.7759037	-1.5568705	0.00551746	0.04768743
GALNT13	17.1924751	-1.8628041	0.00552157	0.04769889
CACFD1	281.266678	-0.6714101	0.00553	0.04770613
KIF15	90.7960562	-1.1036811	0.00552744	0.04770613
QRSL1	282.647728	0.77659324	0.00553074	0.04770613
MARK4	613.767053	-0.7373108	0.00554323	0.0477679
SNORA76C	12.986939	1.80543186	0.00554347	0.0477679
PLEC	23148.1292	-0.7425971	0.00556453	0.04792529
C16orf13	313.003794	-0.5486982	0.00557109	0.04795778
HDAC1	535.633469	0.71024275	0.00559596	0.04814772
JHDM1D-AS1	57.0462787	-0.9013648	0.00560158	0.04817193
AGK	417.629068	-0.4447448	0.00560539	0.04818056

LRRC24	45.3327738	-0.9365957	0.00561302	0.04822196
SAC3D1	84.9003854	-0.8751327	0.00562201	0.04827501
LOC1009966	54.9376297	2.0871057	0.00564171	0.04841995
IQCK	100.631641	1.19213262	0.00565177	0.04848207
CATSPER2P1	29.6099488	-1.1285643	0.00565476	0.04848353
CD40	132.429915	1.50705246	0.00566711	0.04854091
CENPC	990.89715	0.5413656	0.00566467	0.04854091
SMOX	410.583919	1.07159129	0.00567093	0.04854938
CRNKL1	995.415879	0.53687097	0.00569991	0.04873585
MMS19	900.284847	-0.5212587	0.00570123	0.04873585
SLC9A7P1	84.9143077	1.35943989	0.00569997	0.04873585
PAK4	906.636898	-1.108287	0.00570897	0.04877776
FRMD4A	2801.42325	-0.8943016	0.00571376	0.04879439
GLI4	98.9593927	-0.8030996	0.00573036	0.0488875
PRKXP1	83.9186646	1.37718525	0.00572844	0.0488875
LOC1019277	7.69634555	1.45346884	0.00574471	0.04898556
CLASRP	603.994333	-0.6697542	0.00574761	0.04898592
WNT6	692.078208	-1.3981773	0.0057587	0.04905613
FAM167B	18.8618785	1.66622235	0.00576629	0.04909644
PDE3B	295.522469	1.56413906	0.00579111	0.0492833
SNORA74A	189.169508	-1.1650116	0.0057965	0.04930474
SMPD2	111.787966	0.89377109	0.00580119	0.04932016
TBXA2R	24.900744	1.65637055	0.00580695	0.04934472
ADAM10	2492.31148	-0.5445537	0.00581322	0.04934915
ADAMTSL3	7013.84282	1.60196311	0.0058131	0.04934915
HN1L	1169.39027	-0.7222917	0.00582011	0.04938316
GRB7	18.0914051	-1.6074095	0.00584558	0.0495503
THBS1	5970.71565	1.78383123	0.0058431	0.0495503
ZNF296	12.9736503	-1.2364993	0.0058486	0.04955143
FBXO32	1301.52474	1.61371529	0.00585436	0.04957575
ZFP14	495.784189	0.68551826	0.00585839	0.04958544
ATP9A	4744.41726	1.01002891	0.00586488	0.04959142
N6AMT2	104.478324	0.81356085	0.00586405	0.04959142
VPS26B	1265.54879	-0.3512648	0.00587651	0.04966532
EPB41L5	3138.29951	-1.0893832	0.00588096	0.04967851
FRA10AC1	251.420897	0.71621142	0.00588673	0.04968449
HIST1H2BI	163.2453	-1.1200238	0.00588746	0.04968449
PPIF	602.021444	-1.0240081	0.00589497	0.0497234
SGCA	15.8199528	1.92876541	0.00590285	0.04976541
NMRAL1	324.94477	-0.7551004	0.00591343	0.04980574
PGS1	594.852916	-0.5880934	0.00591138	0.04980574
MGARP	23.7645214	2.01328216	0.0059218	0.04985173
STMN2	3.05622375	-2.0618309	0.00593165	0.04991018
DPY19L2P4	9.74041036	1.66161533	0.00595217	0.05005829
DNAJC30	280.736746	-0.6236367	0.00595694	0.05007387
LOC1019281(	240.952443	-1.7291015	0.0059679	0.05014149
AUP1	1215.10258	-0.6967535	0.00598735	0.05025567
FAM179A	132.628517	-1.8768433	0.00598552	0.05025567
SLC27A4	421.067562	-0.5913264	0.00599164	0.05026714
UBR2	2029.23013	0.7423653	0.00599829	0.05029828

FOXD2	1083.57445	-0.8573815	0.00600826	0.05035728
FAM127B	371.802428	-0.8254654	0.00601168	0.0503614
FER	831.547208	0.665951	0.00602547	0.05043446
GIMAP1	135.852427	1.29633312	0.00603216	0.05043446
PCP4	13.1051356	-2.0597087	0.0060305	0.05043446
TPM1	4622.34456	0.95825708	0.00603075	0.05043446
C17orf80	696.792802	-0.4300273	0.00604239	0.0504954
SLC8A1	923.52921	1.27633524	0.00604626	0.05050318
SASH1	1085.14875	1.32352004	0.00605306	0.05053541
WNT11	9.44407768	-1.7279219	0.00606471	0.05060802
PI3	150.589396	-2.0247213	0.00608418	0.05074589
STAT5A	607.294332	0.68685743	0.00609619	0.05082134
FIBIN	3374.77603	1.78371757	0.00612031	0.05099764
OLAH	80.5255718	-1.3014862	0.00613235	0.05107312
ERAL1	407.021374	-0.4824235	0.00614171	0.05110157
LRRK2	1229.07082	0.92722706	0.00614088	0.05110157
TPTE2P5	24.824826	1.26260943	0.00617238	0.05133185
ABRACL	72.6295796	0.94979572	0.00617893	0.05134894
HOXB7	8.7779432	-1.9254573	0.00618042	0.05134894
SNX19	3394.00828	-0.4567259	0.00619574	0.05145131
ADRBK2	466.059681	1.04034086	0.00622357	0.05165747
FPGT	385.818874	0.805867	0.00623449	0.05172309
EPHX3	24.5961	1.48786394	0.0062582	0.05189469
TBC1D9	1738.14209	-0.894682	0.00627743	0.05202899
CSNK1E	813.632945	0.71051258	0.0062903	0.05211055
CLEC2A	50.8388214	-1.9467254	0.00630462	0.05215365
LOC1001323	62.1936604	1.03275763	0.00630289	0.05215365
PAPPA	1345.39712	1.86039654	0.00630207	0.05215365
GLI2	921.17144	-0.8436666	0.00630784	0.05215512
GRIP2	160.416159	-1.6569197	0.00631738	0.05220886
RQCD1	897.226779	-0.473756	0.00633697	0.05232973
SNORA10	10.5108689	-1.5558008	0.0063381	0.05232973
FAM227B	54.2748493	0.81179254	0.00636399	0.05251824
LOC283731	3.62668434	-1.8884388	0.00637088	0.05254985
RMI2	55.1186368	-1.2336133	0.00642066	0.052935
YIPF5	1178.99041	0.8545267	0.00642622	0.05295536
PTTG1	87.0891452	-1.4591522	0.00644009	0.05301929
TMED3	1043.11691	-0.8543441	0.00644015	0.05301929
RAB31	1726.76241	1.12636358	0.00645016	0.05305837
USP35	259.711928	-0.6921081	0.00645108	0.05305837
SGOL2	163.883471	-0.9750399	0.00646028	0.05310857
PPRC1	870.235954	-0.885045	0.00648858	0.05331571
SYTL3	70.6946832	1.07463886	0.00649509	0.05334365
ALG14	88.8597293	0.83069437	0.00651133	0.05342593
FOXRED2	381.061644	-0.9351043	0.00650839	0.05342593
ARHGEF28	1335.97084	1.36200722	0.00652257	0.05344147
FZD2	1088.36644	-0.7793314	0.0065206	0.05344147
TM6SF1	224.370492	1.28360331	0.00652115	0.05344147
CCDC134	61.6837973	-0.5985774	0.00654893	0.05363183
ADAT2	133.258312	0.99200497	0.00657013	0.05375616

UBE2Q1	975.049209	-0.3176319	0.00657037	0.05375616
ITGB4	9234.21776	-1.406245	0.0065775	0.05378886
IBA57	549.246701	-0.5634545	0.00659729	0.053925
PREP	476.607025	0.51376093	0.0066225	0.05401075
PXN-AS1	17.2033294	-1.2429264	0.0066197	0.05401075
SCNN1G	1.99952281	-2.0237135	0.00661716	0.05401075
WDR31	54.3316975	0.97532769	0.00662016	0.05401075
ZNF713	49.2735757	0.89608046	0.00662351	0.05401075
AGPAT4	372.29096	0.85585855	0.00663161	0.05402545
LOC644936	3.87323012	1.78670077	0.00662911	0.05402545
ISLR	15430.1438	-1.5628182	0.00664053	0.05405198
ITGB8	1726.83721	1.26574356	0.00664117	0.05405198
SMC2	573.2246	-0.7292619	0.00667108	0.05426969
RUFY1	768.756107	0.4613224	0.00667577	0.05428215
LMO7	1783.72467	0.82901637	0.00670777	0.05449251
TBC1D19	433.377246	0.73709315	0.00670799	0.05449251
EXOSC5	114.189437	-0.4574989	0.00672604	0.05458481
FAM106CP	39.9898218	-2.0172126	0.00672675	0.05458481
TMEM37	298.133335	-1.2300772	0.00672889	0.05458481
LARP1	5708.34192	-0.6777393	0.00675655	0.05475744
RWDD3	74.2248274	0.792337	0.00675463	0.05475744
HIF3A	1822.16596	-1.5204172	0.00676002	0.0547597
NFKBIA	3094.88372	0.87927373	0.00677365	0.0548442
BRD3	1232.74403	-0.7363914	0.00680391	0.05501133
CPB2-AS1	15.668177	1.46731078	0.00680288	0.05501133
POLR3H	539.509861	0.58328283	0.00679849	0.05501133
FLVCR2	447.784896	-1.611659	0.00683206	0.05519441
HIST1H2BG	292.862191	-1.2146257	0.00683298	0.05519441
MOCS2	690.586911	0.60147634	0.00687055	0.05546268
TCEB2	2133.25657	-0.788403	0.00687265	0.05546268
CHD9	6638.62703	-0.3580639	0.0068834	0.05552326
CA5B	257.726779	0.69624983	0.00689554	0.05559506
CLEC7A	731.791066	1.37168634	0.00690874	0.05566641
CYP4F12	86.748621	1.83041104	0.00691411	0.05566641
HBB	1773.80747	1.59954789	0.00691279	0.05566641
FXN	291.59151	-0.6608763	0.0069337	0.0556935
LINC00260	86.2465464	-1.0422265	0.00693075	0.0556935
SBF2	2136.21966	0.61022128	0.00692177	0.0556935
TMEM106B	1750.94077	0.65460385	0.00693012	0.0556935
WWP2	1226.2534	0.8085752	0.00693036	0.0556935
DDX17	12337.6172	0.55480893	0.00693786	0.05570081
TMEM44	157.247669	-0.7934601	0.00694221	0.05570968
CXCL16	748.082	0.92385938	0.00695266	0.05571775
RHBDF1	1071.3008	-0.6433893	0.00695295	0.05571775
ZNF674	119.205164	0.73583427	0.00694984	0.05571775
TMEM42	134.750902	0.94225551	0.00696747	0.05580801
COX6C	782.48972	-0.6326695	0.0070013	0.0560093
GFER	179.505868	-0.5093602	0.00700239	0.0560093
TESK1	437.091936	-0.7975699	0.00700224	0.0560093
RFX2	201.368225	1.48390599	0.00701024	0.05603841

TMED1	308.514465	0.57665292	0.00701255	0.05603841
CENPI	53.9935784	-1.3389148	0.00702521	0.05611341
AMZ2	740.845818	-0.518292	0.00704698	0.05618269
NRG4	58.1780388	-1.9139156	0.00704675	0.05618269
SEZ6	4.19834112	-1.9210312	0.00704083	0.05618269
SLC25A25	594.888015	1.21436111	0.00704289	0.05618269
CNR1	25.868729	-1.7373877	0.00706241	0.05621765
KIRREL2	9.18516179	-2.0287947	0.00705793	0.05621765
PACRG	7.25587069	1.69172972	0.00706774	0.05621765
SEPP1	4885.35171	1.0682398	0.00705942	0.05621765
TNFAIP3	1682.74444	1.24479596	0.00706675	0.05621765
PTCH2	44.538669	1.42257676	0.00708164	0.05630216
BTBD9	648.661963	0.63504249	0.00708774	0.0563246
CDK11A	53.6343103	1.32628932	0.00712211	0.05656328
SLC24A5	18.7241982	1.94293532	0.00712437	0.05656328
GUK1	1374.16767	-0.5713268	0.00713972	0.05665892
PLA1A	8.61523127	1.70443251	0.00714881	0.05670483
LOC1019271	4.07264096	2.00341797	0.00716718	0.05680331
SIN3A	2100.20812	-0.764134	0.00716784	0.05680331
ARHGDIG	34.2241546	1.60049338	0.00718256	0.05689371
ASB2	6.44536415	-1.703015	0.00719125	0.05690998
KIAA1143	927.679556	0.72080088	0.00719123	0.05690998
PIK3R1	5849.45022	1.45560837	0.00719582	0.05691997
GEN1	328.539507	-0.8569772	0.00720098	0.05693452
RPL10A	5206.8919	0.52874394	0.00720707	0.05695645
ESD	933.213434	0.55577521	0.00721469	0.05699044
FAM160A2	673.876984	0.51606428	0.00721879	0.05699658
DLGAP1-AS1	103.564078	0.98726102	0.00725518	0.05725755
HIST1H2BN	168.813367	-0.9025258	0.00731482	0.05764956
MRPL54	197.970672	0.71626861	0.00731493	0.05764956
RAD18	353.624039	-0.6906736	0.00730883	0.05764956
LPCAT2	668.187659	1.28640969	0.00733313	0.05776649
CYP27B1	9.97164553	-1.2353965	0.00734551	0.05782898
DCAF7	2756.73759	-0.479215	0.0073478	0.05782898
E2F6	221.627496	-0.5893622	0.00735551	0.05785841
LIMA1	2457.84072	0.77479478	0.00736165	0.05785841
SNORA80A	30.172161	1.54601444	0.00735898	0.05785841
LINC00294	266.579251	0.71814872	0.00737688	0.05795159
SURF2	89.0467018	-0.9288996	0.00738258	0.05796984
DAPP1	56.93932	1.42280097	0.00739844	0.05804125
IL13RA1	2099.93995	0.63901967	0.00739716	0.05804125
NAALADL2	191.538964	0.74539246	0.00741289	0.05810145
ST8SIA3	75.6270177	-2.0039047	0.00741228	0.05810145
FEZ2	856.948969	0.50516515	0.00742277	0.05815233
LINC01184	299.012291	0.60319259	0.00743574	0.0582274
REEP3	1883.63965	0.73979336	0.00744275	0.05825571
KMT2D	4663.73407	-0.5055928	0.00744774	0.05826061
SLC19A3	3.32449847	-1.7128771	0.00745016	0.05826061
FAM192A	1147.07011	-0.4477445	0.00746678	0.05836397
CCDC132	596.116904	0.5295562	0.00748415	0.05844654

FAM71F1	4.59118151	1.78877525	0.00748279	0.05844654
NAA40	533.677982	-0.3992447	0.00749549	0.05848189
VAX2	14.9424057	-1.8739394	0.00749489	0.05848189
DKFZp451B0	2.39850685	2.01714035	0.00750332	0.05851248
FGL2	22752.0141	1.85022429	0.00750623	0.05851248
MAN1A1	1245.55747	0.72536039	0.0075115	0.05852694
COL6A4P1	3.80681575	-1.8375506	0.0075236	0.05859468
GBA2	1459.72247	-0.6822743	0.0075297	0.05861559
CPA2	2.72092164	-1.9433244	0.00754354	0.05869092
HIST2H2AB	157.056031	-0.9068056	0.00754622	0.05869092
PCCB	539.489388	-0.7572983	0.00755076	0.05869961
CACTIN-AS1	5.18873734	1.87964533	0.00756609	0.05871246
MRVI1	177.478778	0.97554987	0.00756089	0.05871246
SNORA68	96.3066557	1.38722567	0.00756427	0.05871246
ZNF445	1359.4412	0.5963025	0.00756233	0.05871246
HYI	257.233144	0.76386361	0.00757619	0.05876423
CCDC175	20.7857755	2.00418629	0.0075823	0.05878222
H1FX-AS1	70.4250349	0.95309253	0.00758873	0.05878222
STEAP4	250.119007	1.77765935	0.00758878	0.05878222
C21orf33	1062.59497	-0.3218612	0.00762091	0.05898804
LAMTOR2	252.916438	-0.5519996	0.00762222	0.05898804
RELL2	72.0384442	-1.0701105	0.00764506	0.05913812
KCNH8	2.71411029	-1.9948275	0.00765589	0.05919519
SHC4	434.380846	1.83937792	0.00766789	0.05926126
KCNH4	3.08702219	-1.8450014	0.00769664	0.05942996
LINC01366	6.08688679	-1.8509896	0.00769391	0.05942996
C16orf54	28.9974274	1.31282753	0.0077207	0.05957675
C6orf62	2450.60961	0.56766298	0.00772259	0.05957675
NBPF9	77.039643	0.96306461	0.00773608	0.059654
GSTM5	244.45456	2.00899323	0.0077464	0.05970679
SEC14L5	8.55750648	-1.7130203	0.00776348	0.05981152
PPP1R14A	10.7435077	-1.3799238	0.00777797	0.05989629
RNF8	273.060287	0.52533554	0.00778724	0.05994076
SACM1L	1009.34853	0.56816228	0.00780464	0.06004781
ARHGAP44	188.937952	1.63151895	0.00781359	0.06007268
DTWD1	363.398332	0.5568022	0.00781487	0.06007268
KLHL33	3.33573774	1.9181053	0.00783178	0.06017567
PTGES	189.773296	-1.4821399	0.00783881	0.06020273
CLYBL	99.6385714	-0.6901352	0.00784938	0.0602031
DCAF4	159.989308	-0.7022731	0.00784457	0.0602031
IL11RA	540.038322	1.45562496	0.00784725	0.0602031
HMOX2	552.998437	-0.4665219	0.0078547	0.06021704
SCD	2187.35226	-1.5334769	0.00786661	0.06027096
TMEM132E	6.60662739	-1.3897985	0.00786876	0.06027096
NEXN	160.54599	1.49499659	0.00789829	0.06047016
KANK4	27.4115955	-1.6820445	0.00790549	0.06049831
XKR9	27.5097518	-1.5542951	0.00791557	0.06052148
ZFX	2272.60444	0.57538581	0.00791429	0.06052148
PTMA	5336.07218	-0.6511711	0.00794159	0.06068158
SYTL5	316.706429	-1.8829876	0.00794358	0.06068158

ACO2	1482.79945	-0.7707903	0.00796127	0.0607897
ZNF322	319.8622	0.73022003	0.007967	0.06080639
BHMT2	711.460475	1.62983607	0.00798635	0.06088451
UBAP2L	2522.10571	-0.5604717	0.00798788	0.06088451
ZNF564	435.116941	0.73161021	0.00798756	0.06088451
ECH1	830.377243	-0.7049156	0.0080244	0.06110863
ZNF487	64.3926573	1.23315709	0.00802233	0.06110863
CCDC144CP	358.330654	-1.8117598	0.00803832	0.06117216
EFCAB6	62.5885848	0.98286252	0.008047	0.06117216
FOXA1	5.71477992	-1.9970284	0.00804306	0.06117216
PKDCC	1812.60139	-1.3972093	0.00804641	0.06117216
TTC18	177.090553	0.90951237	0.0080572	0.06122261
COX19	236.033435	0.71590897	0.00807145	0.06128194
KIFAP3	996.341189	0.63940974	0.00807215	0.06128194
THBS2	1103.66408	-1.5779264	0.00808119	0.06132345
DRAM2	393.464312	0.64966738	0.00811159	0.06139128
GORASP1	631.109409	0.56476526	0.00810906	0.06139128
ITGB1BP1	463.507262	-0.5704942	0.00810312	0.06139128
RER1	1096.10671	0.49672211	0.00811121	0.06139128
SLC25A44	589.378796	-0.6585727	0.00810333	0.06139128
TMEM231	149.646936	1.09870775	0.0081061	0.06139128
SWAP70	1957.38026	0.69504653	0.00811898	0.06142011
SET	4675.92742	-0.5261989	0.0081231	0.06142421
LPAR6	1089.98902	0.73221266	0.00813793	0.06150927
B3GALT5	6.1425304	-1.8215477	0.00814305	0.06152086
CHRNE	24.8956806	1.65187091	0.00815639	0.06156748
PELO	681.755208	0.68614572	0.00815473	0.06156748
CEACAM21	23.1959393	1.29695355	0.00817229	0.06160625
HDLBP	11992.7354	-0.6041916	0.00817111	0.06160625
SEPT8	1523.56038	0.7700163	0.00816957	0.06160625
NT5C2	2487.63752	0.77254072	0.00817754	0.06161872
PSMD11	1162.0521	-0.4816604	0.00818177	0.06162358
PIBF1	652.116626	0.47861276	0.00818596	0.06162807
OGDHL	646.9937	-1.5865305	0.0081921	0.06164729
SLED1	31.9584795	1.43202361	0.00820163	0.06169193
A2M	7620.76385	0.94680295	0.00820743	0.06170853
ANKRD20A4	13.5972	1.9509734	0.0082132	0.06172485
AMD1	1392.88563	0.56009598	0.00822056	0.06172611
OTOA	7.20024615	1.64007428	0.00821821	0.06172611
CYP4F24P	28.60532	1.87912045	0.00822532	0.06173485
CACNA1C	699.787033	1.0429302	0.00825427	0.06192504
SORT1	3537.17238	0.63465856	0.00826198	0.06195585
ADRM1	869.427273	-0.7283198	0.00827579	0.06203231
ABCC9	3847.83412	1.88224533	0.0082928	0.06213268
KIT	324.157563	1.40915667	0.00829888	0.06215109
ATP2A2	4778.08038	-0.6303822	0.00830359	0.06215926
ANAPC16	1075.9279	0.51426812	0.0083217	0.06225118
WDR4	233.744129	-0.6801156	0.00832313	0.06225118
KLRC3	6.12798566	-1.4826812	0.00832957	0.06227224
EIF3D	1399.55452	0.6057608	0.00834354	0.06232692

ZNF618	1440.42419	-0.79426	0.00834415	0.06232692
DYNC2H1	1628.56783	0.62915413	0.00835338	0.06234316
ZNF197	696.45656	0.53923817	0.00835358	0.06234316
MRPL45	419.505754	-0.3949992	0.00837081	0.06244454
GTF2H1	720.715871	0.68900254	0.00839286	0.06255468
OGG1	317.305232	-0.5755739	0.00839182	0.06255468
TIAM1	975.464799	-1.3209952	0.00840416	0.06261173
LOC1019272	18.9888751	1.98700789	0.00841394	0.06265067
SETD7	4504.19054	0.5733295	0.00841669	0.06265067
CMPK1	1487.28476	0.70385519	0.00843355	0.06272176
LOC1001309	31.0562142	1.08965847	0.00843204	0.06272176
B4GALT6	267.382125	1.28433177	0.00844345	0.0627644
IGSF1	56.5474237	-1.8499845	0.00845391	0.0627644
SH3RF1	1056.77569	0.68913212	0.0084539	0.0627644
USHBP1	25.3738418	1.52150252	0.00845389	0.0627644
CLEC10A	48.7716075	1.85245904	0.00847482	0.0628653
GABRR2	11.4760626	1.51479784	0.0084717	0.0628653
MANBA	677.703101	0.45539222	0.00848661	0.06292557
ZFPM2	124.529917	-1.4660205	0.00853618	0.0632658
LINC01061	111.037849	-1.0298206	0.00854859	0.06329216
NAA60	822.045479	-0.6695557	0.00854819	0.06329216
TMEM30B	3949.63526	1.55649718	0.0085508	0.06329216
DAAM1	538.615969	1.11003203	0.00856778	0.06336319
LEPREL1	300.521522	1.67705072	0.00856501	0.06336319
LTB4R	305.657401	-1.0187078	0.00858204	0.06343102
ZNF134	499.662729	0.41107264	0.00858434	0.06343102
RACGAP1	359.939631	-1.0929003	0.00859805	0.06350493
SMC6	688.994634	-0.4209072	0.00860215	0.06350791
STXBP5-AS1	11.4319823	1.44836394	0.00860694	0.06351594
DLD	1392.39456	-0.6163039	0.00861421	0.06351637
DSCR9	16.8803889	-1.0510479	0.00861525	0.06351637
FLT1	988.804813	1.46690867	0.0086181	0.06351637
NR1H3	278.96387	-0.5694198	0.00863148	0.06358768
GPC6	8439.21989	0.90427539	0.00866418	0.06380123
CAMKK2	801.376173	-0.5166421	0.00867435	0.06384869
GIMAP8	384.072947	1.13666541	0.00868461	0.06389682
ARRB1	3169.66576	-0.8065003	0.00870079	0.06395057
GPATCH4	212.993316	-0.7765638	0.00869583	0.06395057
RRN3P3	125.48331	-0.6481057	0.0087061	0.06395057
TBC1D20	1058.11656	0.66029423	0.00870682	0.06395057
MPHOSPH8	1433.87623	0.50933976	0.00871917	0.06399604
RAC3	84.8001599	-1.4843184	0.00872046	0.06399604
GSTM3	794.279728	1.52646042	0.00874622	0.06413026
SKA1	17.9698748	-1.4235268	0.00874444	0.06413026
DPF3	15.1549725	1.44129155	0.00875883	0.06419527
ZNF554	123.033765	0.70787368	0.00878547	0.06436306
CDC14B	601.457715	1.1372044	0.0087968	0.06437144
CXCL11	21.5384146	1.6355419	0.00879787	0.06437144
TNFSF13	241.606578	0.75129188	0.00879545	0.06437144
EMR3	7.98024507	1.83435283	0.00882136	0.06451584

SERPINB10	9.93247409	-1.9712693	0.00883182	0.0645648
ZC3H8	114.360403	-0.6364538	0.00884768	0.06465321
AP5Z1	457.772266	0.5411064	0.00885164	0.06465466
DCST2	10.220257	-1.1615115	0.00886228	0.06470485
PHC2	1131.04021	0.91020721	0.00888781	0.06486365
TBC1D26	2.4782848	1.84045253	0.00889362	0.06487842
ZNF503-AS1	7.06931014	1.94454342	0.00891701	0.06502141
AGAP1	1103.95155	-0.7888124	0.00892902	0.06508134
TMEM11	176.861992	-0.6035973	0.00894219	0.0651497
GPNMB	1705.94082	1.43408468	0.00896539	0.06529105
MBLAC1	17.0003695	1.14674417	0.00897453	0.06532988
LOC1005060	126.018112	1.04158651	0.00900822	0.06553899
USP47	2030.02167	0.53920113	0.0090109	0.06553899
ZNF407	1065.76714	0.44151026	0.00902918	0.06564413
SSNA1	232.591184	-0.4659128	0.00905171	0.06578011
FZD7	2596.90415	1.46118605	0.00905684	0.06578953
TMEM9	987.303798	-0.5278781	0.00907493	0.06589308
RPS15AP10	36.9763756	0.88204228	0.00908474	0.06593638
MIER1	1013.98754	0.62517316	0.00911513	0.06612898
AEBP1	6549.80346	1.60066701	0.00915701	0.06640473
ACTR6	231.411342	0.52837567	0.00917223	0.06648707
LINC00987	28.9957754	1.54267195	0.00918759	0.06657025
C9orf106	28.0935146	1.30463845	0.00919551	0.06659953
CHST7	102.63651	1.4758169	0.009208	0.06666191
RFX7	1006.17471	-0.6443104	0.00921785	0.06670505
NKAIN4	70.4389217	-1.8371148	0.00922997	0.06676458
SYNGR2	873.258163	-0.9930452	0.0092386	0.06679886
TRAIP	21.7506853	-0.8798329	0.00924406	0.06681025
EPCAM	4.62925693	-1.7422811	0.00926162	0.06690894
SNORA72	6.05630542	1.74315655	0.00927043	0.06694443
CNOT3	530.458919	-0.6329934	0.00929141	0.06706769
TRAPPC2	286.767673	0.64504509	0.00929727	0.06708184
ANGPTL1	8.65055371	-1.9029205	0.00931439	0.06716517
TCP1	1519.98515	0.55769191	0.00931665	0.06716517
SLC37A4	260.377074	-0.5342745	0.00934495	0.06734095
FHL1	2542.63634	1.47817462	0.00936145	0.06743154
SPON1	120.054772	1.69313443	0.00936562	0.06743323
COLEC10	4.70988509	1.8613549	0.00937291	0.06745743
RHOT2	865.500935	-0.5775498	0.00941196	0.06768172
SMAD5	2876.99763	0.6159544	0.00940924	0.06768172
KLHL7	538.365977	0.69277263	0.00942716	0.06774004
RANBP10	528.79362	-0.4077282	0.00942796	0.06774004
CHTF18	208.862337	-0.8278267	0.00947319	0.06802478
ZNF366	48.0946597	1.31592111	0.00947552	0.06802478
ESYT3	201.685167	1.37491394	0.00948556	0.06803993
NDFIP1	2707.00616	0.73064131	0.00948339	0.06803993
C10orf35	22.3661661	-1.3131854	0.00949989	0.06811427
NME9	6.53143497	1.93831064	0.0095309	0.06830808
ZRANB2	1574.52616	0.55649795	0.00954601	0.06838781
AGAP11	357.588613	1.44733319	0.00956638	0.06845436

PHF11	207.018906	0.70748474	0.00956726	0.06845436
RAPGEF6	1455.5127	0.64089181	0.00955982	0.06845436
POGLUT1	408.579816	-0.4597409	0.00959434	0.06861955
ALDH1A2	2575.78023	-1.9232867	0.00961847	0.06872162
SETDB1	1021.87937	-0.5509722	0.00962063	0.06872162
TYMS	356.787532	-1.161168	0.00961878	0.06872162
SFRP1	2081.21187	1.84053477	0.00962561	0.06872861
SFXN5	345.395042	-0.8284039	0.00964643	0.06884864
ARHGAP42	373.979664	-1.3112134	0.00965177	0.06885811
SLC25A10	78.0576193	-1.1260475	0.00965851	0.06887757
DHX15	2182.00349	-0.3622549	0.00966943	0.06887965
KLHDC1	202.320483	0.91241879	0.0096663	0.06887965
PIGL	142.203524	0.68062242	0.00967084	0.06887965
PCSK7	1028.24398	-0.5283827	0.00967713	0.068888
SNCAIP	126.944387	1.22894314	0.00968004	0.068888
DUOX2	46.2789374	1.8782949	0.0097121	0.06908754
FLI1	517.243581	0.93457395	0.00972105	0.06912255
PRAME	217.908461	-1.8117397	0.00973508	0.06919368
ZNF529	764.597477	0.49621854	0.00973986	0.06919895
EFCAB7	145.178044	0.92612003	0.009745	0.06920686
MYLK2	3.03257897	-1.8884672	0.00975178	0.06922635
LRRC3	63.7861426	-1.3102918	0.00975594	0.06922727
ATP5C1	1450.97507	-0.4867029	0.00976619	0.06927133
CFL1	4378.57729	-0.5408725	0.0097724	0.06928679
TPRXL	8.88426379	-1.5861188	0.009788	0.06936873
FGD5P1	70.5173286	-1.6094175	0.00980188	0.0694384
THSD1	87.1930108	0.93925721	0.00980784	0.06945195
TOMM5	457.254019	-0.7991904	0.00983965	0.0696485
TPM3	3694.35836	-0.4263558	0.00984716	0.06967291
C6orf57	45.7137926	0.91234021	0.0098741	0.06980951
WDR35	851.74731	0.48020339	0.0098746	0.06980951
ASIC1	54.3097494	-1.2956843	0.00990969	0.06991635
COL9A2	96.6759369	1.66258583	0.00990642	0.06991635
PARD6G-AS1	23.9237749	1.11115862	0.00990146	0.06991635
S100A10	4647.2852	-0.914473	0.00991008	0.06991635
UGGT1	3477.84003	-0.3824076	0.00989884	0.06991635
MTR	2692.02438	-0.590797	0.00994165	0.07011029
DPY19L1P1	60.5956366	1.17957793	0.00995682	0.07018842
RAI1	957.281853	-0.5656857	0.00999405	0.07042198
TMED7	2158.13855	0.57008424	0.01000743	0.07048727
H19	10737.2027	-1.9427607	0.01004423	0.07071749
BST1	202.87752	1.56963457	0.01004913	0.07072294
RANBP2	6021.04218	0.36495517	0.01006754	0.07081622
TRIM27	2.30187108	-1.8864537	0.01007353	0.07081622
ZNF396	41.2500396	0.92614487	0.01007476	0.07081622
ZSCAN26	367.595188	0.79923449	0.01008377	0.07085053
MUC1	258.34957	-1.2103563	0.01009177	0.07087772
GEMIN6	87.6024504	-0.6340254	0.01012287	0.07106709
GUSB	702.093138	-0.7238316	0.0101511	0.07117794
ST3GAL4	752.818507	-0.8784393	0.01014563	0.07117794

WDR45B	1228.11298	-0.4098596	0.01014752	0.07117794
USP32P1	507.176851	-1.7979277	0.01018349	0.0713759
LOC115110	15.8581663	1.43168957	0.01019595	0.07143407
FBXL19	407.310068	-0.5963665	0.01021212	0.07151818
CCDC144NL	3.2129158	1.93750918	0.01023496	0.07164886
ADSS	780.806595	-0.5526489	0.01024062	0.07165926
COL7A1	381.033903	-0.9959902	0.0102528	0.07168608
PKP2	4151.34433	1.28079264	0.01025269	0.07168608
GLTSCR1	247.699465	-1.0532436	0.01028824	0.07187934
IL20RB	34.0647643	-1.1417293	0.01028882	0.07187934
SHMT2	1257.24954	-1.0219832	0.0102975	0.07191073
RGS18	113.493151	1.33999893	0.01035463	0.07228028
IL18RAP	6.80238924	1.71926193	0.01037361	0.07238331
MARS	1341.65454	-0.4929535	0.01040192	0.07252195
NID1	5266.82491	-1.1470546	0.01039907	0.07252195
KIAA0355	1340.79466	0.47700495	0.01045225	0.0728366
UVRAG	612.487824	0.49458023	0.01045554	0.0728366
UNC80	139.539175	-1.6271767	0.01048169	0.07298916
SNORA27	11.8550608	1.1138567	0.01048717	0.07299767
CNTD2	7.60593482	-1.5424562	0.01051991	0.07317496
SNORA52	28.5645995	1.27063869	0.01052117	0.07317496
LINC00884	3.65533838	1.74483531	0.01054406	0.07330451
TMEM232	44.770693	1.21573305	0.01056014	0.07338657
ANKRD1	50.6319352	-1.9287816	0.01057804	0.0734812
KCNMB1	39.8533712	1.35273541	0.0105899	0.07353382
FN3K	161.35135	-1.0677735	0.01064342	0.07387558
GPR176	163.801685	1.28529806	0.01064859	0.0738816
CD300LF	19.4060419	1.45353636	0.01068116	0.07407763
NARS	1732.9917	0.46518522	0.01070386	0.07420509
RBPMS2	263.497129	-1.3334241	0.01071808	0.07427368
PRAF2	276.93547	-0.7510403	0.01072435	0.07428714
CHODL	48.562793	1.8026375	0.01075111	0.0744424
SLC41A2	782.898058	1.25036666	0.01076469	0.07450644
SHF	244.323112	1.4864288	0.01077105	0.07452036
ASB14	2.24576783	1.87074888	0.01079759	0.07467388
AAED1	124.701804	0.63002244	0.0108275	0.07485058
CSMD3	3.54582853	-1.8791224	0.01085027	0.07497783
ABCC13	6.83482329	-1.9045583	0.01086759	0.07504797
PHPT1	1045.0231	-0.5581308	0.01086917	0.07504797
PPM1D	557.868208	-0.5470715	0.0108863	0.07511835
STIP1	1163.21822	-0.5587853	0.01088811	0.07511835
COL27A1	342.462255	-0.9934954	0.01089866	0.07516092
CACHD1	865.706583	1.2930526	0.01092025	0.07527956
DDX39A	429.357322	-0.7515026	0.0109317	0.07532824
SEMA4G	125.199491	-1.1165108	0.01093829	0.07534342
ZNF571	113.78242	0.810556	0.01095791	0.07544825
SLC14A2	13.7611618	1.72934564	0.01096265	0.07545066
ENTPD3-AS1	27.1851327	1.25229991	0.01099003	0.07559907
IGF2-AS	69.9562034	-1.5549011	0.01099303	0.07559907
SERPINF2	47.3232129	1.37248327	0.01102287	0.07577395

ZNF205-AS1	7.23676928	1.30952912	0.01104749	0.07591283
GOLGA1	728.829179	-0.4589393	0.01110866	0.07630258
APBB2	5009.6499	0.98219611	0.01117525	0.076653
FAM182A	6.86994956	1.89212847	0.01117617	0.076653
RSF1	1662.76032	-0.3178454	0.01117494	0.076653
UQCRFS1	692.805088	-0.5388565	0.01117754	0.076653
LRP4	1214.71602	1.19216826	0.01118377	0.0766651
LASP1	4362.17402	-0.3816403	0.01122262	0.07680873
RIN1	64.8563962	-1.2570147	0.01121678	0.07680873
SLC1A5	625.641044	-1.3448954	0.01121933	0.07680873
ZNF561	787.50549	0.63140063	0.01122164	0.07680873
LACE1	74.4107401	0.7112509	0.01125015	0.07696644
T	5.19080408	-1.8837022	0.01125926	0.0769981
ZNF462	1438.73821	-0.5310749	0.01126435	0.07700224
DRG2	387.578944	0.61242872	0.01127899	0.07701026
PGLS	511.893974	-0.4497267	0.01127536	0.07701026
PRKCA	3499.67935	-1.0453103	0.01127651	0.07701026
RABL3	338.687964	0.48778888	0.01131794	0.0772273
SNAI3	29.2719704	1.61602879	0.01132877	0.0772273
TMEM185B	756.089023	-0.5783878	0.0113238	0.0772273
ZNF319	256.877792	-0.5730873	0.01132682	0.0772273
ZNF331	769.922842	1.05396587	0.01134525	0.07730895
ZC3HAV1L	283.139117	-0.7387501	0.01135876	0.07737031
SCMH1	594.902631	0.61345442	0.01137913	0.07747829
YTHDF1	1086.97496	-0.6767239	0.01139023	0.07752312
NOL4L	904.854345	-0.9229433	0.01140251	0.07757594
CLDN11	7608.14268	-1.593297	0.01141738	0.07764633
FLJ45513	39.3716118	1.1808917	0.01142191	0.07764635
TRG-AS1	12.7302089	1.4614937	0.01146424	0.07790331
RAD51C	175.131977	-0.67591	0.01148417	0.07800782
NUP214	1738.80548	-0.4340968	0.01149587	0.07804174
PGAP1	1778.3961	-0.8390569	0.01149826	0.07804174
RIC3	555.509609	0.66824551	0.01152105	0.07816552
FAR1	1954.82331	0.58245108	0.01153034	0.07817273
RPL34	3780.51608	0.61298328	0.01153122	0.07817273
SLFNL1	7.63134039	1.52134694	0.01159091	0.0785464
CLDN9	4.74766426	-1.6250438	0.01160852	0.07863467
SLC39A4	61.1813325	-1.3278805	0.01162657	0.07872586
BHLHE41	748.534257	1.20521239	0.01164543	0.07876784
CNTNAP2	11.8453614	-1.5446666	0.01165571	0.07876784
GPS1	1303.07698	-0.4238308	0.01165256	0.07876784
KCNE4	1246.77641	1.23806884	0.01164787	0.07876784
NADK2	827.686718	0.79491015	0.01164037	0.07876784
EEF2	39579.9684	0.54131326	0.01167677	0.07881042
GIMAP6	395.545371	1.06241146	0.01167271	0.07881042
GLIS2	1403.47331	-0.968532	0.01168038	0.07881042
ITGAV	5031.1878	0.579264	0.01167316	0.07881042
KCNG2	8.66385743	-1.8964535	0.01170889	0.07897175
FAM184A	44.414164	1.33519257	0.01171932	0.07901104
HNMT	960.256651	0.98252234	0.01174929	0.07918201

FCER1A	30.1137743	1.74032923	0.01175703	0.07920308
ABHD3	255.579426	1.21777098	0.01178484	0.07935633
C10orf82	13.630791	1.85273255	0.01179827	0.07935633
SLC44A3	58.6761052	1.0495101	0.01179716	0.07935633
TRAF7	1236.47001	-0.6294168	0.01179181	0.07935633
ANKHD1	162.386007	0.66892888	0.01180461	0.07936786
C10orf131	8.94172676	1.55711146	0.01183944	0.07950861
MAATS1	64.9935251	1.32631102	0.01183186	0.07950861
MCM4	791.977083	-0.9484687	0.01183621	0.07950861
TPCN1	1607.39401	-0.5577558	0.0118578	0.07960078
FMNL3	2198.69732	0.93875153	0.01188821	0.07976381
NME1	362.486719	-0.7397702	0.01189138	0.07976381
LHPP	138.111968	0.88537864	0.01191493	0.07989056
GREM2	24.491314	1.89619654	0.01192293	0.07991295
LINC00167	4.3895501	1.39315178	0.01195428	0.08006053
RAB11FIP1	1758.67228	-0.9572497	0.01195117	0.08006053
FAM8A1	1761.46559	0.7765869	0.01196132	0.08007646
SLC52A3	47.2230301	-1.8675503	0.01199887	0.08029655
TWIST2	98.4163057	-1.4246735	0.01200447	0.08030271
CRYM	14.7010581	-1.7768592	0.01202461	0.08037477
MTG1	357.01368	-0.5744737	0.01202231	0.08037477
DDX58	592.472701	0.79219975	0.0120475	0.08049642
KCMF1	951.447544	-0.3473681	0.01206958	0.08055704
LOC440300	154.509066	1.05705863	0.01207065	0.08055704
PCGF6	114.33862	0.71291486	0.01206642	0.08055704
COX4I1	2368.79132	-0.6136874	0.01209322	0.08067633
ANKRD36BP2	14.6589471	-1.5361188	0.01212993	0.08088973
ATXN2	1514.77047	-0.5491427	0.01214384	0.08095106
DYNLRB2	2.99097255	1.76303444	0.01216109	0.08102512
EMC1	1282.9721	0.5110346	0.01216439	0.08102512
FGD1	387.285793	-0.6393395	0.01217055	0.08103474
JPH2	86.3300855	-1.4675645	0.01218577	0.08109186
SLC16A1-AS1	64.3766486	0.87528746	0.01218858	0.08109186
CPD	6816.6299	-0.7159762	0.01222397	0.0812958
TMEM107	156.482856	0.88529949	0.01223169	0.08131563
LINC00857	45.2717159	-1.4696813	0.01226229	0.08148747
PGR	2141.91369	1.71816178	0.01232812	0.08189327
GPR171	8.60630535	1.53711003	0.01235945	0.08206964
ALDOA	11566.136	-0.8688393	0.01238333	0.0821964
SEC14L6	32.8471655	1.804709	0.01239102	0.08221564
LOC1005071	17.3440072	1.42318761	0.01240419	0.08223343
ST8SIA1	247.658761	1.86611088	0.01241286	0.08223343
THAP9-AS1	482.809192	-0.515233	0.01240675	0.08223343
WDR83OS	749.142473	0.567327	0.01240951	0.08223343
BMI1	72.8999874	0.70478697	0.01243633	0.08226187
CASP8AP2	730.30833	0.52585843	0.01242392	0.08226187
PAQR6	108.501565	-1.3921062	0.01242755	0.08226187
SDHC	890.87713	-0.5691411	0.01243618	0.08226187
EXOC3L1	19.4539286	-1.0394509	0.01244954	0.08227557
PXK	321.013855	0.84270266	0.01245278	0.08227557

ST14	58.5631808	-1.2958331	0.01244464	0.08227557
F3	5066.03716	1.34093721	0.01247067	0.08236068
FAM19A2	39.6150477	0.89542935	0.01247696	0.08236068
TMEM132D	110.632298	-1.8773816	0.01248005	0.08236068
MTF2	420.847297	0.64801322	0.01248658	0.08236725
ST8SIA4	436.42303	0.80496977	0.01249344	0.08236725
VAMP1	249.192593	0.63750839	0.01249545	0.08236725
GPR64	300.403038	-1.6662181	0.01251795	0.08242063
NCL	5382.81214	-0.5956052	0.01251457	0.08242063
TOP1	2152.89966	-0.559939	0.01251069	0.08242063
GPRASP1	1386.23289	0.92902417	0.0125461	0.08254833
PPM1K	682.515791	0.76089093	0.01254696	0.08254833
IDH3A	940.977248	-0.9327844	0.01257206	0.08268177
CAMLG	593.232125	0.6612785	0.01258269	0.08268832
GS1-259H13	26.6951239	1.17552002	0.01257841	0.08268832
SAMHD1	1315.57983	0.92377645	0.01261746	0.08288511
CHML	532.465149	-0.6092914	0.012624	0.0828963
RSPO2	34.4255861	-1.8521695	0.01264159	0.08298008
FASTKD1	315.012849	-0.6117206	0.01265448	0.08300122
ID3	1554.29154	0.65527343	0.01265392	0.08300122
COX6B2	4.29163777	-1.6185642	0.01266622	0.08301477
LOC145783	172.815446	-0.6591797	0.01266512	0.08301477
DLGAP4	1417.68046	-0.8121452	0.012676	0.08304716
C18orf61	37.2606043	1.88062526	0.0127044	0.08313804
MLC1	5.89523734	-1.6750666	0.01269591	0.08313804
TACC2	985.179082	-0.8045493	0.01270073	0.08313804
PON2	1135.02943	-0.5581722	0.01270966	0.08314077
CIZ1	1210.00236	-0.5008195	0.01272862	0.08323307
HTR1D	15.1636745	-1.6440094	0.01274963	0.08333871
LOC442497	3.11541959	1.81753967	0.01275649	0.08334294
PIWIL2	21.7797708	1.4664652	0.01275999	0.08334294
LOC728752	18.44171	1.04573858	0.01278909	0.08350123
GUSBP1	347.611459	0.559385	0.012815	0.08362564
LOC1026064	21.6365625	1.07994635	0.01281824	0.08362564
MAOB	26.9234308	-1.6558942	0.01282276	0.08362564
LOC1001304	12.4894744	1.26875046	0.01284117	0.08371392
AGO4	871.285923	0.65223632	0.01286398	0.08383073
TSPYL2	2608.06923	0.89334534	0.01290171	0.08404473
AS3MT	8.81247888	1.33278774	0.01291143	0.08406886
CHTOP	754.481952	-0.3875759	0.01291521	0.08406886
TCEAL1	349.024275	0.82694603	0.01292257	0.08408488
FAM76A	201.51956	0.83401419	0.01294004	0.08416667
C1GALT1	583.112785	0.9466836	0.01294786	0.08417958
SUSD2	244.231955	-1.7694356	0.01295184	0.08417958
TMPO-AS1	49.067487	-0.8015635	0.01298898	0.08438906
CCND1	22751.1851	-1.0766407	0.01300661	0.08447158
RNF32	9.95793103	-1.0920307	0.01301205	0.08447499
DENND4B	1189.4186	-0.5793959	0.01301783	0.08448053
ZNF7	376.897777	-0.5558643	0.01303891	0.08458534
GPSM2	224.745362	-1.3567214	0.01306458	0.08470861

P2RY14	95.9404077	1.69464866	0.01306778	0.08470861
RBM38	118.99023	-0.8461386	0.01308196	0.0847476
RPL7	8443.94547	0.58789959	0.01308438	0.0847476
SYN1	34.7525129	-1.1098594	0.0130886	0.0847476
HIST1H2AG	255.162468	-1.2935568	0.01309393	0.08475014
FAM180B	73.8928919	1.84749517	0.01310149	0.0847671
KCNN3	186.686389	1.30348369	0.01311757	0.08483911
PCDHGA12	725.168666	1.29932648	0.01312618	0.08486288
AFTPH	869.80851	0.36275326	0.01313877	0.08488381
GABRE	2630.99348	-0.9932298	0.01313931	0.08488381
LIMS2	501.98017	-1.0163114	0.01314912	0.08491524
MIR143HG	48.861001	1.46979458	0.01317273	0.0850357
TIE1	143.775835	1.28223109	0.01319152	0.085125
AHCY	1627.72533	-0.7470747	0.01321299	0.08523146
ZNF442	135.013638	1.35221226	0.01322114	0.085252
RDM1	3.29128647	-1.7035083	0.01323453	0.08530633
TCEB3	925.735769	0.46303397	0.01324524	0.08534329
SCN1B	262.830952	0.9375866	0.01325823	0.08539493
TNNT2	1734.30876	-1.3706612	0.01327462	0.08546847
CDK12	2185.33667	-0.3161308	0.01329596	0.08557372
C15orf61	54.4096137	-0.8301987	0.01330422	0.08558676
OR9A4	12.9612677	1.60411835	0.01330796	0.08558676
RNASEH1-AS	63.7902795	-0.9874018	0.01332897	0.0856898
ZAK	1284.79105	0.77730678	0.01335091	0.0857987
LYRM4	228.984028	0.73385609	0.01335642	0.085802
LRIG1	1347.16246	-0.860089	0.01339108	0.08599247
NPY6R	29.5863237	1.8628773	0.01340447	0.08604625
CYP1B1-AS1	42.2315578	1.58639085	0.01341911	0.08610802
FKBP5	8976.31718	1.09265575	0.01344086	0.08617029
FXYD1	454.511342	1.23734823	0.01344387	0.08617029
PPP5C	851.366584	-0.4560603	0.01343479	0.08617029
TMEM255A	31.5448855	-1.7625907	0.01346684	0.0862531
ZNF423	1810.10195	-0.7490569	0.01346361	0.0862531
FLJ34503	14.9021221	1.77120477	0.01348512	0.08627359
HIST1H2AB	66.8129292	-1.0124375	0.01348386	0.08627359
TIMM8B	348.89786	-0.5607825	0.01347603	0.08627359
MFN1	967.719379	-0.2231113	0.01350525	0.08637019
PYCR1	707.567385	-1.2785933	0.01353243	0.08651179
IFFO2	150.257781	1.00750754	0.01353808	0.08651571
ELTD1	180.9101	1.38700056	0.01356752	0.08663929
FAM120A	4433.02412	-0.4044342	0.01356668	0.08663929
CDHR2	3.52956483	-1.3891138	0.01358348	0.08667676
SLC16A1	2479.31882	1.52761823	0.01357949	0.08667676
CASP2	661.049895	-0.6260626	0.01363691	0.08695106
HDDC2	521.32563	0.40323487	0.01364639	0.08695106
TLR8	76.3917225	1.21127351	0.01363528	0.08695106
ZMYM6NB	199.446305	0.7400392	0.01364673	0.08695106
RAMP1	31.4835777	-1.6069701	0.01368789	0.08718093
S100A11	3067.14225	-0.7513039	0.01369583	0.08719916
NFASC	191.22798	-1.5087686	0.01373147	0.08738931

TSNAXIP1	46.9759526	1.02222062	0.01373588	0.08738931
TIPRL	768.863194	-0.3105456	0.0137457	0.08741942
KDM1B	1047.83793	1.2697235	0.01377529	0.0875751
CHI3L1	34.6361934	-1.842029	0.01378792	0.08762297
HNRNPUL1	3416.70867	-0.3804514	0.01380349	0.08768944
CHCHD7	180.164068	0.61577859	0.0138379	0.08771606
DCLK3	2.11080874	1.82560219	0.01382438	0.08771606
KDELC1	250.270813	-0.8863153	0.013813	0.08771606
LOC1005066	143.852102	0.69835476	0.0138339	0.08771606
MFSD4	40.3042932	-1.0534003	0.01383938	0.08771606
RIMS2	5.31752978	-1.8043747	0.01384345	0.08771606
TBC1D17	617.160091	-0.4194385	0.01383601	0.08771606
HGF	131.326586	1.39843147	0.01385744	0.08773147
MARCKS	4785.81304	0.81343835	0.01385985	0.08773147
SNORA53	918.351007	-1.0189993	0.01386121	0.08773147
ABHD17B	315.721704	-0.430179	0.01390854	0.08774486
AGBL4	7.66666987	1.369489	0.01390961	0.08774486
BRINP3	74.6869152	-1.6957511	0.0138951	0.08774486
C16orf86	43.9054727	1.45615127	0.01390958	0.08774486
DLG1	1683.60919	-0.2840911	0.013897	0.08774486
HMGA1	394.12784	-1.1804006	0.01387514	0.08774486
ITPKA	9.2495015	-1.7119335	0.0139186	0.08774486
PATL1	790.938978	-0.6522433	0.01391956	0.08774486
SCARF2	596.127502	-1.0930297	0.01391075	0.08774486
TUBBP5	3.67441524	-1.8555333	0.01389508	0.08774486
ZNF69	107.465685	1.46673685	0.01390828	0.08774486
RNF214	450.761992	-0.4781178	0.01399388	0.08818095
GPM6A	16.8665676	-1.580705	0.01400966	0.08824801
CHKB	33.9014179	0.78768241	0.01401679	0.08826053
ZMYND12	35.6565802	1.11423165	0.0140474	0.08842085
ETAA1	441.34506	-0.4525188	0.01407829	0.08858281
GORASP2	1195.13236	-0.5479981	0.01410974	0.08873333
JMJD1C	4906.87414	0.43926109	0.01411255	0.08873333
RAB40AL	25.6260794	0.97240762	0.0141356	0.08884567
CAPN3	257.084962	0.72891758	0.01414818	0.08886146
CTSS	1272.47703	0.87903652	0.01415364	0.08886146
PCM1	3905.87314	0.56992293	0.01414876	0.08886146
SLC25A33	58.3369267	1.04087023	0.01417362	0.08895437
TTC36	3.51108271	-1.4367273	0.01418213	0.08897525
GNA11	2279.18019	0.49611844	0.01419441	0.08898722
TBC1D13	704.382142	-0.3267121	0.01419009	0.08898722
MBOAT1	745.919654	1.22456469	0.01420109	0.08899662
EDC3	646.440415	-0.7164176	0.01420641	0.08899749
MASTL	273.370145	-0.4473171	0.01422065	0.08905414
C8orf82	189.427644	-0.6735217	0.01423586	0.08909487
CREB3L4	107.307002	-1.0187208	0.01425269	0.08909487
SNRPG	304.414104	-0.6461942	0.01424601	0.08909487
TAF15	920.172138	-0.7043941	0.0142531	0.08909487
ZNF391	70.9586254	0.86018496	0.01425161	0.08909487
ATP2A3	105.146767	0.97126217	0.01427206	0.08916053

ELAC1	129.819679	0.63508315	0.014274	0.08916053
CMTR1	991.426007	0.4848056	0.01427936	0.08916158
FAM132A	28.8393157	1.66502908	0.01433205	0.08945805
WFIKKN1	3.55572494	-1.5436628	0.01434961	0.08953509
SLC7A5P1	21.1507242	-1.5004333	0.01441036	0.08988146
PLXND1	3437.92348	-0.7791945	0.01444959	0.09009233
PSMD2	2506.4311	-0.381374	0.01445466	0.09009233
ZNRF1	173.690619	-0.7895664	0.01448572	0.09025316
BCL2	970.551194	1.2800116	0.01450975	0.09035478
CERS5	726.569488	-0.6432023	0.01451256	0.09035478
AFF3	1306.80488	-1.3167907	0.01454481	0.09037799
BAIAP2-AS1	317.947531	0.62392944	0.01459487	0.09037799
CDC123	608.343908	-0.4889287	0.01458939	0.09037799
CKAP2	628.39096	-0.9213349	0.01455231	0.09037799
DLGAP1-AS2	16.6752907	1.06452804	0.01460677	0.09037799
DZANK1	41.4978801	1.03159815	0.01453758	0.09037799
EID2B	80.7535469	0.85878509	0.01461162	0.09037799
FGGY	232.227891	0.68892082	0.01461079	0.09037799
GIMAP4	472.694256	0.95030619	0.01461633	0.09037799
HBG2	4.18430609	1.7336502	0.01460712	0.09037799
KCNMB3	168.452276	-0.8692673	0.01456441	0.09037799
LTB4R2	64.8857768	-0.9659983	0.01461129	0.09037799
PRPF3	1035.09059	-0.5660786	0.01458392	0.09037799
RILPL1	374.891029	-0.5548606	0.01460932	0.09037799
RNF2	452.221528	-0.4473669	0.01454904	0.09037799
SNRNP200	5761.75576	-0.5610828	0.01453151	0.09037799
TMEM150A	193.818767	0.54758799	0.01460689	0.09037799
ULK1	1038.83325	-0.7484053	0.01461064	0.09037799
ZNF451	2106.35879	0.61426592	0.01458925	0.09037799
ZRANB1	1365.79421	0.33926783	0.01462383	0.09039178
MED21	720.408602	0.62733549	0.01463035	0.09039956
BCS1L	276.225609	-0.5050172	0.01465264	0.09050472
ZNF649	299.53749	0.39754496	0.01466308	0.09053659
CAPN12	23.2064626	1.67001727	0.01468102	0.09058389
LOC1005063	59.2578169	1.40634623	0.01468129	0.09058389
ZNF787	267.473499	-0.5638705	0.01468807	0.09059316
ZNF438	253.589021	0.64997351	0.01471136	0.09070421
DUOXA1	64.4771733	1.80339939	0.01476598	0.09094294
FAM89B	359.022064	-0.6380621	0.01476375	0.09094294
TRIM23	607.054229	0.47225707	0.01475985	0.09094294
CA12	229.427588	1.76329063	0.01478755	0.09104317
IFNLR1	114.922804	1.21273274	0.01482154	0.09118845
PARP11	231.375661	0.60807475	0.01482178	0.09118845
SMAD9	2971.29214	1.52589193	0.01484925	0.09132473
GFM1	993.101008	-0.4260019	0.01485488	0.09132663
GAPT	108.963986	1.24483813	0.01487929	0.09141126
RHCE	2.31583359	1.74985561	0.01487644	0.09141126
EMC10	1971.62991	-0.7459591	0.01489458	0.09146609
SPNS3	27.3312957	1.72331903	0.01489888	0.09146609
HHIP	241.866653	-1.8204509	0.01495509	0.09174468

MAP1S	476.590411	-0.5584416	0.01496769	0.09174468
RTP4	46.842017	1.25874066	0.01496125	0.09174468
TNFSF8	158.271972	1.18881264	0.01495442	0.09174468
UCKL1	476.149234	-0.6268887	0.01497098	0.09174468
FAM172A	1072.95612	0.65915269	0.01501314	0.09191614
NLRP12	17.4973676	1.56017135	0.01501502	0.09191614
USP21	325.020705	-0.5163378	0.01501359	0.09191614
TMEM57	751.43204	0.70443406	0.01504059	0.09203986
HES6	62.0686461	-0.9975653	0.01510288	0.09238808
DGKB	42.8492869	1.44988981	0.01511645	0.09243815
TBC1D10C	27.7028089	1.20193184	0.01513017	0.0924891
FAM86FP	12.6986736	1.04311634	0.01516006	0.09260586
RYR1	736.440903	1.2602584	0.01515618	0.09260586
CHST11	828.94034	0.92012013	0.01517044	0.0926347
LINGO1	117.901517	-1.200868	0.01517558	0.0926347
LOC150776	528.859305	-0.8909921	0.01518497	0.09265911
PAX2	16.6807826	1.83135754	0.01520204	0.09273026
PRKAR1B	310.336625	0.88021317	0.01525774	0.09303698
LOC283194	12.4451992	1.48485215	0.01530121	0.09326889
TMEM9B	711.513338	0.61514537	0.0153133	0.0933095
GLIPR1L2	45.0167404	1.36282752	0.01536989	0.09358785
MTMR11	198.360617	-0.7925133	0.01536898	0.09358785
CHPT1	526.069641	0.97196103	0.01538277	0.09363306
SLC9A3R1	1286.44852	-0.8022339	0.01541204	0.09377799
AGO2	849.251734	-0.5984071	0.01544033	0.09391681
ACSL6	28.3910778	1.48724504	0.01545989	0.09396919
SH3RF2	16.8866182	-1.3527966	0.01545927	0.09396919
RPS6KA4	561.861331	-0.6195229	0.0154875	0.09410371
GTF2F1	1160.04298	0.43375381	0.01550007	0.09414676
MRPL35	531.168522	-0.3677419	0.01551745	0.09421897
RRS1-AS1	386.314159	-1.1502825	0.01552797	0.09424951
TAF6	558.766089	-0.526487	0.01553896	0.09428289
ARID3B	105.811254	0.78497463	0.01556451	0.0944045
DIRAS1	83.9316757	-1.4002953	0.01559159	0.09453539
THBS4	150.66402	-1.3891816	0.01560196	0.09456487
GPC4	636.826007	1.12040075	0.01561108	0.09457112
LTBP2	17929.4314	1.54336758	0.01561401	0.09457112
DDX41	800.502875	-0.4703319	0.0156279	0.09462182
TBXAS1	447.421025	1.05731653	0.01564083	0.09466673
EFS	467.57484	1.48985546	0.01565082	0.09468828
TRPV3	96.8397973	-1.4359948	0.01565542	0.09468828
CSTB	961.470134	-0.8279481	0.01568263	0.09481945
LINC01105	5.10580199	-1.7832553	0.01569117	0.09483764
ATP5J2	738.924309	-0.4764902	0.01570668	0.09488596
CCDC181	53.4214493	1.21551282	0.01571022	0.09488596
METRNL	1084.56494	-0.9794075	0.01571603	0.09488766
USP24	3028.0501	0.55550485	0.01574298	0.09501695
SSRP1	1529.44914	-0.4765459	0.01576211	0.09509898
NUTF2	774.949351	-0.6075887	0.01577144	0.09512183
SLC16A4	500.419042	0.67118063	0.01578315	0.09512565

ZBTB16	2292.48418	1.02342203	0.01577788	0.09512565
PEX26	701.071792	-0.7622027	0.01579099	0.0951395
SLC5A12	8.46304761	1.64408669	0.01582174	0.09525785
SSUH2	4.23494279	1.73015728	0.01581825	0.09525785
IL6ST	14310.509	0.58758251	0.01584086	0.09531924
SIGMAR1	656.596095	-0.5506117	0.01584304	0.09531924
AP2M1	4020.13191	-0.3293875	0.01586768	0.09543401
RASD1	105.561955	1.66450251	0.01589086	0.09553996
SCO2	104.955183	-0.7494045	0.01589808	0.09554992
KLHDC8B	342.528116	0.77825562	0.01591739	0.09563248
PIF1	27.7163585	-1.3904276	0.01592691	0.09565622
LINC00176	80.2438181	-1.0753347	0.01595373	0.09578375
OSBPL8	2361.05078	0.50127159	0.01601853	0.09613918
CALU	5344.75733	-0.7606673	0.01604882	0.0962873
DKK3	2451.19709	1.20036317	0.01607602	0.09639437
HTR7P1	175.081175	0.71054788	0.0160779	0.09639437
GPR116	536.269837	1.30957232	0.01610401	0.09648352
LOC1019271(	3.37566585	1.7290978	0.01609898	0.09648352
FADD	248.135971	-0.560172	0.01616344	0.0967046
NCALD	793.969901	1.6716877	0.01615833	0.0967046
PRDM2	1993.24153	0.63533728	0.01616168	0.0967046
RTP1	2.07831859	1.78486409	0.0161505	0.0967046
YY1AP1	1096.06738	-0.4011333	0.01618441	0.09679631
SLC17A5	384.199171	0.98345907	0.01620653	0.09689482
VWDE	2.45823095	-1.8035091	0.01621303	0.09689997
PEBP4	4.41478903	1.6692792	0.01622571	0.09694195
LTK	2.84221803	1.70015967	0.01625667	0.09709314
KCNE1	30.8538702	1.46976835	0.01627082	0.09714387
MRPL30	742.718926	-0.2778447	0.016303	0.09730217
ZBTB45	151.429211	-0.6132237	0.01631909	0.09736433
VWA2	36.6086103	1.76553274	0.0163545	0.09754167
ARHGAP15	177.03699	1.11816606	0.01638906	0.09764729
LINC01021	4.84231688	-1.8051299	0.01638927	0.09764729
SMG1P3	772.925438	-0.6187758	0.01637984	0.09764729
MVD	291.31567	-0.8292518	0.01640182	0.09768814
DPYD	924.230486	0.9489106	0.01643927	0.09785023
POLR3D	337.057399	-0.4975077	0.01644043	0.09785023
TNIP2	278.31101	-0.545324	0.0164618	0.09794343
VKORC1L1	817.690944	-0.4551299	0.0164735	0.09797908
TAF8	576.603881	0.55586616	0.01648486	0.09801269
ME1	897.406413	1.00341062	0.0165244	0.09821376
ATL1	536.005444	0.97195638	0.01653881	0.09826537
LOC1019290.	9.77399465	1.23439614	0.01658929	0.09853119
ULK2	879.864259	0.52919443	0.01665856	0.09890838
FBRSL1	612.933889	-0.7983456	0.01667312	0.09896063
CKAP4	2377.48722	-0.8470144	0.0166978	0.09905753
YBX2	3.06590676	-1.5884556	0.01670099	0.09905753
ARSB	975.471404	-0.6481673	0.01672146	0.09914471
FUT7	5.80296749	1.47188903	0.01676988	0.09939345
TEX21P	6.41018392	1.54473346	0.016775	0.09939345

FOLR1	61.5994305	1.69581926	0.01679417	0.09947271
GPI	4754.27336	-0.771151	0.01681548	0.09949925
KNSTRN	152.38402	-0.6626434	0.01680791	0.09949925
KRCC1	409.219135	0.55216243	0.01681992	0.09949925
PPP6R2	970.002027	0.41341564	0.01682184	0.09949925
EPB41L2	5096.80963	0.95955621	0.01684333	0.09952353
LOC1019276	9.45018979	1.80354677	0.01684333	0.09952353
MPZL1	4650.19166	-0.6368375	0.01683744	0.09952353
TMEM156	46.0368078	1.59715191	0.01685124	0.09953595
FAM105A	1403.87643	1.11409068	0.01687231	0.09962617
OASL	60.0060013	1.33564667	0.01688644	0.09967528
SPATA7	177.665882	0.72362393	0.01690223	0.09973418
DNAH8	4.39895924	1.48965523	0.01693027	0.09974517
HERC2	4478.61533	-0.3394645	0.01692003	0.09974517
PLD1	2134.05242	0.94351642	0.01693314	0.09974517
PWWP2A	577.036699	0.60188495	0.01691522	0.09974517
SLA	788.702097	1.1105791	0.01692973	0.09974517
C1orf131	135.00592	-0.52108	0.01695234	0.09982397
GTF2IRD2	108.516774	0.6827914	0.01695849	0.09982596
DBP	275.761349	-0.8669699	0.0169681	0.0998483
C19orf60	251.085508	-0.6104872	0.0169984	0.09992382
CXCR6	21.1482044	1.46253933	0.01699583	0.09992382
LCN10	5.35600293	1.5977052	0.01699677	0.09992382
HINT2	211.507295	-0.6508788	0.0170086	0.09994954
MIR181A1HC	63.9506814	-0.9879983	0.01702352	0.10000297
ADAR	6966.18155	-0.4240248	0.01703994	0.1000652
FOXO4	218.285989	0.88301531	0.01704615	0.10006742
PRKAR2B	519.599221	1.20661205	0.01705748	0.10009972
DNAJB1	2382.21651	0.86216694	0.01709266	0.1002719
TPMT	510.62597	1.07757892	0.01710127	0.10028809
MBD2	854.985155	0.79989134	0.01710922	0.10030048
METTL25	156.640239	0.57245165	0.01712408	0.10033419
TMEM134	158.503434	-0.6012905	0.01712666	0.10033419
NPIPA5	74.0461572	-0.8831313	0.01713863	0.10033946
RASAL3	121.74344	1.1310848	0.01713925	0.10033946
SPATA41	17.5348674	-0.9887038	0.01716484	0.10045499
CHD7	995.687042	-0.7206942	0.01722871	0.10071013
MROH8	80.5365789	0.92576878	0.01722003	0.10071013
TASP1	237.027806	0.65218352	0.01722374	0.10071013
UROD	683.339778	0.54486347	0.0172319	0.10071013
ALK	10.245064	-1.4220913	0.01725459	0.10073983
C14orf79	82.6879533	0.88520893	0.01724516	0.10073983
PRKG2	459.944723	-1.7902839	0.01725211	0.10073983
AZI2	788.567475	0.65507005	0.01726318	0.10075567
C9orf156	201.372596	0.45295959	0.01728953	0.10077952
HIST1H3F	139.330821	-1.3409709	0.01728661	0.10077952
MAPK6	1190.67181	-0.8506348	0.01729075	0.10077952
XPR1	1086.71249	-0.3851007	0.01729034	0.10077952
HIST1H2AE	345.924389	-0.9812011	0.01732573	0.10092715
LRRC59	1468.09664	-0.668105	0.01733767	0.10092715

RNU6ATAC	16.4678441	1.38187965	0.0173396	0.10092715
USP36	1096.01582	-0.7009168	0.0173324	0.10092715
LINC00869	62.6609195	0.82905395	0.01737137	0.10104356
NCOR2	4842.27601	-0.5562436	0.01736661	0.10104356
PRIM2	230.75792	0.48481612	0.0173952	0.10114793
TRIM9	89.9664283	1.56554336	0.01745346	0.10145228
MAP2K3	420.681472	-0.8370345	0.01746028	0.10145756
ZNF536	352.581328	-1.3211415	0.01747395	0.10150268
WFDC10B	4.00801565	1.78649046	0.01748338	0.1015231
EIF5B	1019.3991	-0.3198341	0.01750553	0.1016173
HOXA4	7.71694105	-1.7487241	0.01751405	0.1016324
ADAMTSL4-A	121.829142	1.08654054	0.01754	0.10174857
RPS14P3	31.5273082	0.92215318	0.01755178	0.10178255
CRABP1	6264.83547	-1.5423573	0.01756337	0.10181537
PHOSPHO2	56.9950352	0.8043453	0.01757016	0.10182033
UBE2W	771.419387	0.4835192	0.01760438	0.10198422
UBXN4	2743.26263	-0.3110759	0.01761156	0.10199139
DDX51	388.961833	-0.4578221	0.01762973	0.10201172
HEATR5A	1788.28791	0.64962842	0.0176329	0.10201172
MOK	180.683895	0.9663067	0.01762646	0.10201172
MID1IP1	669.79548	-0.9444643	0.01764947	0.10203878
PPM1N	30.0895096	-1.3142506	0.01764466	0.10203878
CDC25C	13.7858299	-1.4602168	0.01767709	0.10211063
ETFA	940.163595	-0.5563869	0.01769167	0.10211063
FAM216A	89.2877286	-0.6147113	0.01766829	0.10211063
KIAA1958	267.963244	-0.8479314	0.01769759	0.10211063
MAPKAP1	1298.14841	-0.2748896	0.01768381	0.10211063
TIMM17A	725.780229	-0.5926668	0.01769013	0.10211063
C4orf46	129.430198	-0.7593182	0.01770481	0.10211794
C14orf1	346.338629	-0.5866746	0.01771592	0.10214773
CRNDE	92.2200669	1.15200216	0.01772909	0.10218933
ACCS	279.27533	1.01341873	0.0177418	0.10222824
LOC1019297	4.00244019	1.48995556	0.01775082	0.10224496
PRR18	2.13280901	-1.7872123	0.01775661	0.10224496
CLU	67442.9224	0.89222021	0.01776707	0.10227089
DNAJA3	710.43536	-0.4676758	0.01779558	0.10240063
FBXO4	159.92971	0.83197122	0.01782558	0.1025389
PRKAG2-AS1	15.9455915	1.4692483	0.01783193	0.10254109
CNN2	1188.55978	1.02530441	0.0178631	0.10258283
GRHPR	994.676894	-0.4792573	0.01786298	0.10258283
PPAT	222.565394	-0.6767171	0.01786159	0.10258283
SOX7	21.9790243	1.41672662	0.01785756	0.10258283
SPTA1	5.15820969	1.520888	0.01787789	0.10263342
LINC00276	5.91128777	1.51783812	0.01788965	0.1026666
KCNJ8	398.520384	1.68475297	0.01790235	0.10270515
NME2	298.018347	-0.8505252	0.01791303	0.10273208
ZNF695	10.2762556	-1.4203042	0.01794387	0.10287459
CD93	2297.11598	0.86842946	0.01795717	0.10291645
HMGN3-AS1	59.9048056	1.10156725	0.01797036	0.1029362
HYMAI	152.356897	1.32073592	0.01797261	0.1029362

C19orf25	285.978897	0.58601377	0.01797944	0.10294097
ANP32E	943.616824	-0.5998683	0.01803078	0.10320053
SHC2	673.508095	0.98022763	0.01810579	0.10359532
CHRNA6	14.0883672	1.4174843	0.01812328	0.10366079
C11orf70	64.7913217	1.32880831	0.01820515	0.10402624
LINC01119	5.71495144	1.40078617	0.01820316	0.10402624
SOGA3	23.1598953	1.42361964	0.01820535	0.10402624
CDH8	58.8669958	-1.5888913	0.01821915	0.10406572
LOC643733	37.7282601	1.33379152	0.01822438	0.10406572
SRD5A1	123.987278	-0.8794075	0.0182409	0.10412538
ATF3	284.007951	1.57536806	0.01825434	0.1041675
RPS29	1556.60887	0.69797653	0.01826235	0.10417852
LOC653712	9.54158566	-1.5288839	0.01827	0.10418754
NUTM2D	32.5486423	1.08265515	0.01828031	0.10421176
CDCA2	75.8952973	-1.3880591	0.01828854	0.10422403
CASP7	247.975456	0.71227609	0.01831541	0.10434255
SEC16A	3600.1054	-0.5525003	0.01832349	0.1043539
S1PR1	219.317483	1.26700182	0.01833048	0.10435909
CLEC14A	156.9852	1.23301483	0.01835414	0.10439035
CXorf36	137.803221	1.27270704	0.01835229	0.10439035
HERC6	240.451455	0.7921372	0.0183603	0.10439035
MGAT3	2398.74645	-1.2265903	0.01835881	0.10439035
TRAK2	1281.80865	-0.3757653	0.01839977	0.10458016
CNIH2	6.56985539	-1.1894254	0.01840879	0.10459679
SLC35G1	419.859939	1.16069414	0.01841672	0.10460723
VENTX	187.974408	1.36756407	0.0184241	0.10461454
RAB40C	317.959242	-0.5035523	0.01850983	0.10506653
ASIC3	43.5797018	-1.0341777	0.01852115	0.10508689
ITIH3	21.0675805	1.65245044	0.01852566	0.10508689
RASEF	90.2514861	1.72076958	0.0185806	0.10536373
ZNF107	398.261054	-0.3937657	0.01859711	0.10542252
IL6R	507.998775	0.99632209	0.0186392	0.10562623
LINC00460	10.7580327	-1.7734447	0.01865314	0.10563551
TTL	1033.11273	-0.4840586	0.01865006	0.10563551
IKBIP	408.039834	-0.8021457	0.01866273	0.10565493
FBN2	3602.31898	-1.2450474	0.01872659	0.10592388
RABL2B	153.494792	1.00615968	0.01872875	0.10592388
SMUG1	361.815648	-0.5084065	0.01872701	0.10592388
CD28	100.340207	-1.3518192	0.01875269	0.10602433
WDR66	43.9050092	1.17927852	0.01879864	0.10624916
LOC286437	206.427126	0.60674979	0.01880822	0.10626827
PHLPP2	1392.29705	-0.8160303	0.01881623	0.10627858
GCSHP3	162.282557	-0.5319524	0.01883905	0.10633749
PRKRIR	859.399332	-0.408863	0.01883567	0.10633749
TPBGL	3.80726737	-1.5227741	0.01885041	0.10636661
GADD45B	1851.75385	1.22091043	0.01887401	0.10639487
ITGA8	439.048041	1.65981642	0.01887363	0.10639487
UBAC2-AS1	15.7325874	1.01702639	0.01886513	0.10639487
ZNF512	1265.13988	-0.3963204	0.01888461	0.1064197
LAPTM4B	1614.85534	-0.8098255	0.01892707	0.10662395

ITIH4	517.329704	1.32710166	0.01895756	0.10676067
CISD1	316.282478	-0.5758579	0.0189782	0.10677184
FBXO38	954.940999	0.47967513	0.01897327	0.10677184
LOC1002892	154.447735	0.71255847	0.01896956	0.10677184
LOC283788	684.527991	1.20996975	0.01900496	0.10685896
RERGL	13.2011755	1.45525522	0.01900614	0.10685896
GNE	824.05466	-0.5890328	0.01902308	0.10688419
GRIK2	27.0409332	1.53461786	0.01902033	0.10688419
WDR81	1162.14716	0.66989301	0.01902996	0.10688784
BMX	12.4159133	1.53513351	0.01904354	0.10692912
SLC27A2	12.950246	1.43210499	0.01905883	0.10694503
UTP20	1144.27985	-0.3842276	0.01905705	0.10694503
IQCC	35.7448966	0.96209588	0.0190952	0.10707908
STAM2	1225.16519	0.3720252	0.01909234	0.10707908
FAM114A2	481.09737	0.43944139	0.01911768	0.10717013
RRN3P2	101.510297	1.27789711	0.01915954	0.10736221
TMEM91	84.259149	0.69924591	0.01916445	0.10736221
GNG12	1458.44564	0.87004955	0.01918542	0.10740959
SEC22C	962.68368	0.59103468	0.01918404	0.10740959
NSUN5P2	208.334343	0.82254159	0.01923807	0.10766919
SNX22	33.4198641	1.41013858	0.01926529	0.1077513
SPNS1	454.028095	-0.5514697	0.01926273	0.1077513
SRPRB	947.379963	-0.4467302	0.01927853	0.10779022
PLSCR4	1083.88082	1.00914738	0.01928872	0.10781207
KBTBD12	18.6695835	1.62972336	0.01930677	0.10787784
MTHFD1	832.662548	-0.5599714	0.01931718	0.10790087
ANP32B	1247.62665	-1.0577193	0.01936116	0.10804063
C17orf70	640.460367	-0.4775011	0.01938491	0.10804063
C3orf62	124.759786	0.80871652	0.01936173	0.10804063
DAK	389.470046	-0.6289456	0.01937962	0.10804063
OR2A7	8.34378238	1.40316723	0.01937281	0.10804063
PTPRS	2326.9269	0.59757968	0.01937509	0.10804063
VILL	222.081085	0.68070367	0.01938626	0.10804063
PGPEP1	1135.94535	0.35485628	0.01939482	0.10805328
SLC52A2	283.095502	-0.5695698	0.01944896	0.10831973
ZNF502	242.030996	0.59691569	0.01949842	0.10855996
WDR3	623.125899	-0.5502156	0.01951202	0.10860048
NAA25	912.785326	-0.4332788	0.01952519	0.10863852
AQP6	2.86727586	-1.7348396	0.01953323	0.10864804
AKIP1	223.183921	0.66183044	0.01955207	0.10871761
PIK3C3	958.765643	0.6588203	0.01957079	0.10878645
ESCO1	626.336207	0.56798292	0.01958703	0.10884149
EIF4E3	553.58955	0.83042672	0.01961778	0.10897709
ADCY4	81.1936314	1.25483788	0.01963838	0.10902095
ZNF271	714.947805	0.55260583	0.01963363	0.10902095
XRCC6	2220.29944	0.5835786	0.01965324	0.10906818
SCIN	202.229855	1.29474663	0.0196774	0.10916694
SURF1	356.437675	-0.3876341	0.0196863	0.10918107
ASPHD1	2.84059966	-1.7269924	0.01970263	0.10920107
NPPA-AS1	8.06957441	1.34314394	0.01969966	0.10920107

PRKRA	444.868932	-0.2828259	0.01971556	0.10923744
APOBEC3B	11.7956594	-1.2859418	0.01973722	0.10932215
C17orf107	58.5117103	1.05035288	0.0197524	0.10933647
ZZZ3	890.405336	0.54739459	0.01975254	0.10933647
DMGDH	242.768888	1.2775386	0.01979344	0.10952755
GSTM2	307.228774	1.05720671	0.01982608	0.10967276
DLG2	226.674019	1.36955937	0.01987245	0.109887
RPL39L	72.6218578	-0.9985328	0.01987761	0.109887
C12orf4	489.975137	0.48142085	0.01989834	0.10993082
NETO1	38.8873156	-1.7262867	0.0198952	0.10993082
POLG	1027.70939	-0.5507344	0.01991542	0.10995436
ZNF512B	1504.70915	-0.6883647	0.01991183	0.10995436
C1orf198	1358.36919	0.97456837	0.01995066	0.11009442
GOLM1	2028.5157	-0.7773127	0.01995361	0.11009442
ISM1-AS1	3.70243711	1.65051083	0.01996265	0.1101089
LOC283038	4.44830574	-1.5391476	0.01997297	0.11013042
HSPA1A	14.9504345	-1.7189129	0.01998227	0.11014634
PAM	5680.66381	1.08353983	0.01999975	0.11018589
SNX2	2116.04213	0.77342364	0.02000229	0.11018589
TELO2	446.365992	-0.5984625	0.02002548	0.11027825
BBS2	1032.70092	0.65995635	0.02004243	0.11031953
RPL26L1	182.673323	-0.5101035	0.02004583	0.11031953
C9orf170	15.4189691	1.17238433	0.02008113	0.1104594
PKN2	1586.39645	0.49633655	0.02008411	0.1104594
HPN	20.4907578	1.26237248	0.02009759	0.11049814
COL2A1	2511.06533	-1.6068087	0.02013644	0.11057003
GEMIN8	290.257195	0.56165361	0.02012337	0.11057003
SNF8	464.925242	-0.4169734	0.02013138	0.11057003
TRIB3	123.407595	-1.3009365	0.02012568	0.11057003
DHRS1	195.686427	0.76872757	0.02017507	0.11074674
PDZRN3	1825.27759	1.15160905	0.02019919	0.11084369
MRPL21	247.396782	-0.454697	0.02022385	0.11090811
THRB	67.0258217	1.53066903	0.02022016	0.11090811
GTPBP3	142.309699	-0.548129	0.02023153	0.11091477
COMMD8	141.326622	0.59995986	0.02026303	0.11105149
DHTKD1	1183.82895	-0.6820005	0.02027866	0.11105149
PTPLB	307.820395	0.84626153	0.02028234	0.11105149
RTCB	727.011549	0.56486784	0.02027204	0.11105149
TMEM189	515.110623	-0.8761575	0.02029139	0.11106559
LRRC27	167.50679	0.70310572	0.0203016	0.11108607
TOE1	129.891419	0.81855045	0.02031348	0.11111563
CHRNA4	417.093931	-1.7450167	0.02032307	0.11113268
C10orf11	79.7484539	0.78227464	0.02041768	0.11129772
CD86	318.373859	0.96628204	0.02041598	0.11129772
CPOX	220.793169	-0.5516575	0.02042099	0.11129772
LINC00839	10.8564168	-1.7416852	0.02041513	0.11129772
MCM7	862.656905	-0.6620375	0.0203784	0.11129772
NINL	1274.41555	-0.9798697	0.02037037	0.11129772
NME5	75.4577754	1.37831307	0.02041721	0.11129772
PANK1	416.339057	-1.1097651	0.02039716	0.11129772

PCGF5	2705.88435	0.844488	0.02042614	0.11129772
PINLYP	92.0573632	-0.6291399	0.02038489	0.11129772
RRP8	223.258719	0.53114248	0.02043106	0.11129772
TLE1	261.321828	0.96576038	0.02042892	0.11129772
MTX2	266.75279	-0.3763987	0.02050196	0.11164852
DOCK9-AS2	19.4667428	1.29125623	0.02051991	0.11171084
DLL4	123.080349	1.366389	0.02052817	0.11172037
RFPL1S	30.6003401	1.46295911	0.02055471	0.11182935
ZBTB46	572.550252	-0.843011	0.02058269	0.11194607
EMC9	70.1757727	-0.7760699	0.02062749	0.11215419
ESYT1	2464.20758	0.48631722	0.02065603	0.11227385
VAT1	2280.95861	0.79602782	0.02069745	0.11246336
LRRC38	90.4002961	-1.5316222	0.02071033	0.11249773
LOC727896	58.6877227	1.02448607	0.02074092	0.11262824
CAT	1773.41932	-0.720784	0.02075525	0.11263481
FARS2	224.858314	0.6555715	0.02075151	0.11263481
SH2D4B	11.5757425	-1.6089969	0.02076531	0.11265377
ABCF3	723.35902	-0.259881	0.02081579	0.11269294
EGFL6	3351.67877	1.7440432	0.02081849	0.11269294
GTF2B	240.811257	0.60472	0.02080601	0.11269294
HN1	406.539619	-0.6623968	0.02079163	0.11269294
LINC00467	57.5224084	-0.8129509	0.0208048	0.11269294
PAQR5	3776.11758	-1.4469024	0.02078033	0.11269294
POLK	1155.7845	0.35447787	0.02081706	0.11269294
DPY19L4	1032.89447	0.61946431	0.02085671	0.11275763
MPG	494.336217	-0.5131134	0.02085309	0.11275763
SDK1	86.9905638	-0.822688	0.02084164	0.11275763
SEC61G	262.831707	0.81948741	0.02085233	0.11275763
ITPKC	599.213534	0.49159614	0.02089534	0.11293087
TMEM104	538.411892	-0.5590652	0.02095898	0.11322453
TMEM201	270.750953	-0.7847897	0.02096684	0.11322453
TXNRD2	172.762009	-0.5597603	0.02096946	0.11322453
TNFSF13B	111.850048	0.94590833	0.02098512	0.11327348
PIEZO2	3612.49629	-1.3938375	0.02099399	0.11328571
TRAPPC8	1422.68175	0.44526634	0.02101812	0.11338026
ATE1-AS1	20.3149198	1.36284628	0.02104504	0.11338901
CSRNP1	596.340544	1.21938614	0.02103977	0.11338901
PTTG2	6.1697044	1.50112658	0.02104616	0.11338901
TRABD2A	14.8753174	-1.1728748	0.02102636	0.11338901
CD300LB	11.2344462	1.27975301	0.02107729	0.11352107
DLEU2L	36.3651922	1.03248648	0.0211129	0.11360593
FFAR2	4.71347766	1.65541603	0.02110953	0.11360593
WNT10A	9.29393723	-1.5870373	0.02110451	0.11360593
GFAP	537.481296	-1.6499769	0.02112052	0.11361131
BTG2	6984.34617	1.0033847	0.02114374	0.11366618
SLC35E3	281.935814	-0.5258201	0.02114396	0.11366618
PTCHD4	170.980958	1.2015769	0.02115893	0.11371102
SRSF2	2589.75345	-0.3690251	0.02119284	0.11385763
RGN	17.9322119	1.71139563	0.02120954	0.11390255
TSPO	255.460096	0.91893984	0.02121447	0.11390255

PHF13	202.885754	0.57612974	0.02123694	0.11398753
PAXBP1	1246.85915	-0.8170796	0.02124572	0.11399897
GMPPA	325.181104	-0.5764772	0.02126155	0.11404829
C19orf68	54.0471283	-0.6553951	0.0212792	0.11407165
RBM4	804.055284	-0.3338691	0.02127778	0.11407165
WDR34	432.928856	-0.4903039	0.02130018	0.11414848
GFRA1	2186.48388	1.73734541	0.02131812	0.11420895
BMP7	419.901145	-1.5098441	0.02133556	0.11426671
C5orf49	5.17829301	1.620571	0.02137659	0.11435894
GALNT8	3.43699559	1.61272478	0.02137258	0.11435894
LINC00673	3.5807248	-1.4796914	0.02137942	0.11435894
ZNF674-AS1	92.0867957	0.63893405	0.02136388	0.11435894
COPS6	836.921838	-0.5642288	0.02139039	0.11436479
FGF13	8.90206187	-1.3155369	0.02139729	0.11436479
TEKT2	2.43109057	1.67031668	0.02140051	0.11436479
PHF1	54.998836	0.72110356	0.02140743	0.11436619
AOAH	338.834967	1.125257	0.0214148	0.11436994
FAM161A	98.3010741	-0.6768981	0.02142941	0.11441241
LOC1001304	11.0348227	1.63564974	0.02146863	0.11458613
RNF216	1586.51278	0.35832809	0.02149035	0.1146664
MICAL2	1504.49289	-0.7902231	0.0215173	0.11477452
FBXO10	312.623268	-0.7734786	0.02156692	0.11500347
C6orf211	338.554542	0.5254302	0.02160185	0.11504678
LINC00174	243.767055	-0.8644777	0.02160086	0.11504678
LMAN2L	359.692531	0.65494138	0.02159452	0.11504678
TMEM170B	491.422144	0.69869127	0.02159819	0.11504678
SLC37A3	801.079978	-0.514099	0.02161119	0.11506081
MRPL46	231.011845	-0.7152375	0.02167662	0.11537341
AARS2	606.625563	0.53281866	0.02168866	0.11537653
SCRIB	1058.17123	-0.6169613	0.02169065	0.11537653
DISP2	496.785995	1.27617558	0.0217086	0.11543622
MYBPC2	16.9520664	-1.6406782	0.02173651	0.11544676
NLRC3	39.5340022	0.97658421	0.02173748	0.11544676
PARM1	81.3725911	1.27370496	0.02173666	0.11544676
ZNF671	295.425037	0.93334031	0.02171911	0.11544676
CMIP	576.545958	-0.8618803	0.02175921	0.11552639
NFYC	472.828088	-0.493231	0.02179138	0.11565834
TSPAN12	154.022834	1.22205175	0.02179754	0.11565834
ARAP3	231.297077	1.14582322	0.02181001	0.11568875
S1PR5	4.27670052	-1.3665024	0.02182606	0.11573811
FLJ16779	96.7014024	-1.5953991	0.02184355	0.11579511
C4orf3	1973.31924	0.62723271	0.02187466	0.11583565
CLASP1	2277.71977	-0.2436208	0.02186076	0.11583565
EXT2	1849.88958	0.65884076	0.02188248	0.11583565
RAB36	148.985626	1.083651	0.02187045	0.11583565
ZNF791	600.316909	0.57453332	0.02188494	0.11583565
TMEM119	364.811736	1.47671038	0.02189271	0.11584103
NPEPPS	2015.77707	-0.49753	0.02193003	0.1159902
SCAF1	1036.56141	-0.4655122	0.02193441	0.1159902
C11orf24	442.820261	-0.5676807	0.02197437	0.11616569

ZNF286A	341.115594	0.59206854	0.02198322	0.11617673
EML5	168.916919	1.22404137	0.02203237	0.11640063
ASB6	439.717181	-0.4249328	0.02207089	0.11653241
PRICKLE2	588.390033	1.08131489	0.02206753	0.11653241
TCERG1L	13.6385159	-1.7191858	0.02210258	0.11666384
KIF26A	95.972735	-1.3787468	0.02215004	0.11685046
NARF	366.387205	-0.5455336	0.02215836	0.11685046
VAPA	2247.48085	0.59375646	0.02215183	0.11685046
TEAD4	156.466631	-0.9137532	0.02217681	0.11691185
EFCAB14	3555.60162	0.49818094	0.02218773	0.11693351
IFIT1	718.545608	0.50925159	0.02219612	0.11694179
CRYGS	17.3284475	1.13726434	0.02221437	0.11700202
RAD21-AS1	3.08948919	1.58823625	0.02224297	0.11711675
C20orf196	27.4447588	0.98849024	0.02228696	0.11713277
GSE1	1158.55812	-0.7907294	0.02228537	0.11713277
HBD	6.68293534	1.44825878	0.02225511	0.11713277
PCAT1	9.61682346	-1.3149296	0.02227408	0.11713277
RAB3IL1	2074.56965	-0.6018492	0.02228355	0.11713277
SMG7	1873.68615	-0.6936084	0.02227158	0.11713277
HCG4B	10.2270767	1.67792257	0.02232127	0.11727718
DENND4C	2357.9721	0.43811369	0.02233537	0.11731536
SLC7A8	566.379547	1.0729248	0.02236106	0.11741437
ZNF562	1238.3956	0.48167975	0.02237387	0.11744571
BIRC2	1419.71455	0.33447856	0.02239636	0.11749189
MKX	26.3316314	-1.4881019	0.02239194	0.11749189
MSI1	54.4423414	0.84690287	0.02242692	0.11757433
NOMO1	882.716877	-0.559645	0.02243262	0.11757433
UBQLN1	2357.85255	-0.5497912	0.02241954	0.11757433
CHMP1B	2670.73151	1.15771545	0.02246008	0.11768234
CIDEB	104.643706	0.81705087	0.02248877	0.11775513
RPL27A	5356.81169	0.4001114	0.02249456	0.11775513
TRIM26	38.2113068	-1.6804735	0.02249081	0.11775513
UBE2I	1049.62405	-0.4881219	0.02251158	0.11780834
RARA	722.729577	-0.5206474	0.02252938	0.11786552
LEFTY2	3.27560521	1.59456812	0.022544	0.11790611
DAGLB	370.712165	0.59421618	0.02256803	0.11799583
CYBA	713.383201	0.72962552	0.02257727	0.11800816
GABARAP	2163.76226	0.56229326	0.02258716	0.11801886
RRAS2	565.465325	0.74801687	0.02259994	0.11801886
SNCB	3.75753458	-1.7088047	0.02259772	0.11801886
HEATR2	335.561361	-0.5050866	0.02260817	0.11802594
CCR2	35.5987882	1.34491737	0.0226438	0.11810418
NDUFB10	660.972147	-0.453013	0.02264013	0.11810418
SOX12	251.452184	-0.9954528	0.02264058	0.11810418
KIAA1524	215.430916	-0.6729588	0.02265398	0.11812138
RBM5	2264.19146	0.48987746	0.02266382	0.11813679
NDUFB6	299.123359	0.44548073	0.02267172	0.1181421
BOC	7068.19074	1.34917109	0.02270241	0.11826611
DRD4	11.8043634	-1.0667985	0.02272664	0.11835644
CXorf56	339.831118	0.47251769	0.02276004	0.11849442

ERBB3	60.0510822	-1.1955688	0.02277952	0.11855989
IGBP1	731.444842	0.49165558	0.02280338	0.11861211
MARCH9	173.967908	-0.5343338	0.02280327	0.11861211
C19orf38	24.9141443	1.15627083	0.0228158	0.11864079
PLXNA2	3080.63321	1.16983936	0.0228715	0.11889066
SRCAP	3189.55851	-0.5292993	0.02287771	0.11889066
CPVL	453.196492	0.81359351	0.02289426	0.11894068
GALC	597.221497	0.52646822	0.02291538	0.11901436
LTC4S	53.0292913	1.15062044	0.02294409	0.11912742
GDF7	20.5420762	-1.3545808	0.02295634	0.119155
NHSL1	492.866799	0.92180786	0.02297115	0.11919582
ZNF827	462.0327	-0.9358806	0.02304123	0.11952333
KRTCAP3	9.09576851	-0.9938747	0.02305128	0.1195299
PKN3	162.094701	-1.0926161	0.02307035	0.1195299
SELL	90.9446262	0.95212798	0.02306509	0.1195299
TNFRSF1B	974.791404	0.81023393	0.0230668	0.1195299
SETD4	318.994173	0.42281737	0.02310077	0.1196514
C15orf39	533.409495	-1.1055413	0.02315585	0.11981467
CTNNBIP1	380.015739	0.76758513	0.0231542	0.11981467
PDIA4	2921.44059	-0.7874469	0.02316021	0.11981467
WASH1	194.183184	0.66344484	0.02314703	0.11981467
AKAP8	754.992043	0.44040734	0.02317369	0.11983321
SEC31B	531.068873	0.74286598	0.02317776	0.11983321
AIM1	205.408669	0.94879816	0.02322704	0.1199074
ASIC2	2.25979159	-1.649114	0.02322494	0.1199074
GRTP1	89.5097649	-0.4761225	0.02320738	0.1199074
HTR3A	3.0351101	-1.7034964	0.02320852	0.1199074
TRPC3	565.022136	1.43286358	0.0232174	0.1199074
SF3B4	549.544051	-0.6528081	0.02323903	0.11993323
CHAF1A	188.598216	-0.8101147	0.02326222	0.12001686
AK9	142.425831	0.87223058	0.02330763	0.12009346
GPIHBP1	39.4294886	1.3286232	0.02332243	0.12009346
IP6K1	951.520278	0.48895713	0.0233258	0.12009346
ISCU	1110.07282	0.43489669	0.02332605	0.12009346
MAPK14	1048.11394	0.5203739	0.02330027	0.12009346
TP53INP2	1059.98649	-0.8051366	0.02329017	0.12009346
UBAC1	574.692795	-0.5560231	0.02329442	0.12009346
DNAH6	83.3163078	1.21119271	0.02333929	0.12012559
LOC339803	178.535549	-0.5262572	0.02336017	0.12016101
RCAN1	644.897121	0.63356566	0.0233584	0.12016101
PCDHB5	544.862374	-1.1643041	0.02338383	0.12022706
PDK4	1903.97842	1.38384145	0.02338702	0.12022706
IL23R	3.93080257	-1.5216647	0.02341486	0.12033415
GRIK3	5.73216054	-1.6178889	0.02342927	0.12037216
PRR29	21.0895705	1.44210318	0.02346067	0.12049743
AIM1L	2.33786897	-1.6399346	0.02350622	0.12067343
THOC7	258.089601	0.49298154	0.023509	0.12067343
GALNT9	39.5830833	-1.6752944	0.02355407	0.12071447
NRIP3	31.5442833	1.5387342	0.02352454	0.12071447
PAN3-AS1	44.0575589	0.66715652	0.02354013	0.12071447

RBMX2	228.686603	0.46574713	0.02355166	0.12071447
SAP25	102.059782	-0.7827351	0.0235567	0.12071447
WDR65	19.3455474	-1.5841323	0.02355919	0.12071447
CBWD1	210.499743	0.56859236	0.023586	0.12081579
THSD7A	125.531228	0.97267418	0.02359854	0.12084394
ITFG3	1327.4748	-0.597401	0.02360755	0.12085405
SMYD3	125.714848	-0.5377024	0.02363466	0.12095677
SLC25A51	301.791015	-0.6849255	0.02364362	0.12096656
PIK3C2A	3959.44502	0.57039223	0.02366326	0.12103095
GLO1	1354.29578	0.54686339	0.0236901	0.12113211
ACTG1	32942.7805	-0.5572906	0.02373316	0.12113579
C14orf132	482.024408	1.36556276	0.02371128	0.12113579
CXXC4	247.726625	1.54549481	0.02371855	0.12113579
SAMD9L	1943.27067	0.57203897	0.02369798	0.12113579
SLC39A10	1630.60638	-0.5749343	0.0237251	0.12113579
TNFRSF25	75.0488027	1.04456724	0.02373282	0.12113579
ARHGEF2	2095.69655	-0.6404723	0.02376439	0.12124621
IFT22	305.015859	0.60148785	0.02376892	0.12124621
ZNF559	558.81125	0.56605373	0.02378576	0.12129608
AKR1A1	593.404365	0.49594283	0.02380777	0.12131395
GPR132	118.761548	1.0818909	0.02380593	0.12131395
IQCH	46.0782348	0.86786843	0.02381754	0.12131395
OXNAD1	271.157326	-0.7326556	0.02381408	0.12131395
SLC23A2	4256.7663	0.89323167	0.02385196	0.12145323
GLMN	105.586331	0.82521149	0.02386782	0.12149797
ZNF568	398.338529	0.56713421	0.02390917	0.12167239
SLC2A9	106.615996	1.12818591	0.0239309	0.12174688
CDC7	90.2240093	-1.1286044	0.02394726	0.12179398
TRIM61	35.4307591	0.72632855	0.02397851	0.12191682
DOCK8	1528.64982	0.84044261	0.02399269	0.12195276
KLHL21	387.588477	0.82980323	0.024018	0.12204527
ZDHHC1	137.298785	0.66199367	0.02411026	0.12247783
ITGA9	491.021642	0.89596245	0.02411909	0.12248643
CLEC2D	426.33392	0.68846615	0.02418862	0.1228032
HDAC9	669.188001	1.36942747	0.02421357	0.1228775
MYCBP2	4961.21257	0.52255886	0.02421757	0.1228775
LOC1005073	60.6698513	0.5871773	0.02425624	0.12300606
OVGP1	54.8508969	1.12317859	0.02425724	0.12300606
NAGPA	137.122259	0.3750841	0.02430532	0.12321349
MIPEPP3	43.4958824	0.96568341	0.02433253	0.12331499
C6orf226	39.5742938	-1.0059035	0.02438344	0.12353651
EMP3	860.262783	-0.9306679	0.02441276	0.12364857
ANKRD18A	48.267087	-1.668472	0.02445847	0.12373417
CRLF3	289.19789	0.55242541	0.02444143	0.12373417
IFI44	359.146368	0.72808702	0.02444522	0.12373417
MGST1	30.1602919	-1.478711	0.02445849	0.12373417
KDM8	151.611721	0.74226659	0.02447353	0.12377375
GTF3C1	2840.26657	-0.3233081	0.0244952	0.12377569
HSD11B1	32.3507287	1.67025075	0.02449554	0.12377569
SNORA80B	8.64007681	1.22763989	0.02449451	0.12377569

ZC3H18	645.940103	-0.5787598	0.02451665	0.12384587
GMPS	957.94038	-0.4257725	0.02452995	0.12387662
CYS1	291.213277	1.60608066	0.02454576	0.12392
CCDC30	51.2893733	0.88039421	0.02456108	0.12394201
NRIP2	12.4944244	1.46931014	0.02456456	0.12394201
CYTIP	138.430539	1.07554155	0.02457777	0.12397223
ESAM	177.806821	1.19970977	0.02461648	0.1240217
LAMA5	7079.05707	-1.3265587	0.02461472	0.1240217
OSBPL9	1243.47037	0.46937817	0.0246134	0.1240217
TGIF2	431.934659	-0.6716672	0.02460211	0.1240217
LOC90834	53.5089183	0.63705584	0.0246269	0.12403778
CECR5	232.732008	-0.766879	0.02465033	0.12411232
LOC1001313	6.45910027	-1.121521	0.02465616	0.12411232
TANC1	1622.42134	-0.7258826	0.02470578	0.12432562
APBB3	517.580532	0.57933845	0.02471825	0.12435193
CTAGE5	526.116269	0.51554062	0.02479448	0.12469887
CREB5	458.670201	1.23622077	0.02485073	0.12490858
PRPS2	339.327308	-0.4021696	0.02484459	0.12490858
FAM134C	1584.62294	0.40534106	0.02486992	0.1249421
NAT8L	138.67939	-1.0702856	0.02487196	0.1249421
CRTAM	12.7348116	1.34322426	0.02490282	0.12502394
ZNF610	112.086187	0.86801758	0.02489889	0.12502394
GPKOW	315.332078	0.31181602	0.02492279	0.12502533
STK32A	293.083535	1.60082946	0.02492005	0.12502533
TRPV2	140.99804	0.90287285	0.02492495	0.12502533
PDZD11	427.803798	-0.4925725	0.02493799	0.1250542
PLEKHG4B	1957.65528	-0.9981201	0.02494767	0.12506623
PSMD4	1121.19094	-0.3774602	0.02498721	0.12522788
XPO6	1988.62672	-0.3912595	0.02499876	0.1252492
ARPP21	1.95169792	-1.6873966	0.02500987	0.12526828
PARP8	1111.09827	0.55643567	0.02503271	0.12530958
RCC2	1186.14503	-0.5397347	0.02502607	0.12530958
TBCC	212.061983	0.55872595	0.02507826	0.12550098
ABCC8	2.04990705	-1.6770053	0.02509469	0.12552016
SCG2	399.41769	1.66802841	0.02509672	0.12552016
OXR1	1163.70182	0.45863168	0.0251311	0.12565552
BBS9	936.956266	1.09904837	0.02515309	0.12571805
RANBP3	783.061655	0.38852463	0.02515826	0.12571805
LRRC69	13.6372815	1.10531897	0.0252418	0.12598879
SLC4A10	17.442999	1.59972781	0.02523401	0.12598879
USP51	181.432899	0.73650957	0.02523085	0.12598879
ZC3H7A	1956.31995	0.6338003	0.02524144	0.12598879
RASL10A	4.20208097	-1.4779756	0.02529171	0.12620123
DFFB	96.3980689	0.51313945	0.02531566	0.12620272
GADD45G	126.929347	1.48335622	0.02531955	0.12620272
JUN	4416.65788	0.83898506	0.02532142	0.12620272
ZBED3-AS1	26.5751922	-1.198725	0.02531306	0.12620272
LSM3	401.82646	-0.3524726	0.02532921	0.12620488
FGF20	5.43691713	1.56462587	0.02534497	0.12624677
ARPC1B	2009.93437	-0.6283106	0.02535636	0.1262669

PPP1R18	2.60965665	-1.674726	0.02538344	0.12636507
CREB3L1	317.835576	-1.4801669	0.02543866	0.12641999
FAR2P2	13.6430701	1.50825028	0.02542585	0.12641999
KIAA0825	328.783856	0.87128179	0.02543426	0.12641999
LMBRD2	544.620971	0.73767504	0.02543852	0.12641999
MTUS2	72.1951313	-1.6060919	0.02542705	0.12641999
RGP1	486.318403	-0.6549979	0.02541397	0.12641999
PLS1	209.2909	1.4175343	0.02545145	0.12642853
SLC45A2	2.00544564	1.6614697	0.02545511	0.12642853
SUPT6H	2924.84932	-0.3251265	0.02547755	0.12650338
AKR1C1	250.700384	-1.3083964	0.02553141	0.12673416
NEXN-AS1	14.4048247	1.34144111	0.02555881	0.12683348
ANAPC1	1151.85734	-0.3898159	0.02558314	0.12690866
SEMA3F	649.554657	1.12330329	0.02558875	0.12690866
MCTP2	236.214807	-1.2418453	0.02560158	0.12693564
LPCAT3	594.317772	0.73879031	0.02561332	0.12695714
RBM33	2333.41263	-0.4134326	0.02562912	0.12699882
SATB1	275.247201	0.68767578	0.02565097	0.12703375
USP39	568.904615	-0.3243568	0.02564412	0.12703375
TIMM8A	83.3041612	-0.5879519	0.02571927	0.1273189
ZBTB5	569.849834	-0.6424625	0.02572339	0.1273189
NFRKB	730.610114	-0.4690039	0.02573894	0.12732243
PTCH1	990.803022	-1.315386	0.02573168	0.12732243
TMEM43	2616.43893	-0.5088109	0.02581199	0.12764702
OR6W1P	2.25852763	-1.5033544	0.02582606	0.12767133
RBP7	37.0191234	1.26518191	0.02583178	0.12767133
CASP6	136.951308	0.5694236	0.02586577	0.12769223
FKBP7	383.905451	-1.030166	0.02584768	0.12769223
NCAPD2	815.706685	-0.5994845	0.02586562	0.12769223
SYMPK	1344.98737	-0.4490296	0.02585235	0.12769223
FKBP10	3438.96075	-0.7813434	0.02587763	0.12771405
JUNB	3138.96912	1.25069394	0.0259438	0.12796704
LOC1027248	66.6326519	0.76429939	0.02593778	0.12796704
PPM1F	723.665303	0.43089688	0.02596946	0.1280568
LAD1	8.68742698	-1.4852445	0.02601047	0.12815582
MAGOH2	5.89019086	1.40339094	0.02601194	0.12815582
RNF180	355.07037	1.28835177	0.02599966	0.12815582
DNAL1	565.682899	0.66516687	0.02602482	0.12818252
ALMS1P	6.16122474	1.374413	0.02605627	0.12826397
SNORA22	103.059249	-1.1490792	0.0260563	0.12826397
ATG9B	4.67027535	-1.5924296	0.0260965	0.12831465
CANT1	725.896712	-0.4223321	0.02607921	0.12831465
SCAMP3	795.280732	-0.5737237	0.02609183	0.12831465
SFXN4	220.447004	-0.5353343	0.02608244	0.12831465
CERS2	4215.18779	-0.4345053	0.02611412	0.1283645
ADA	63.2518246	-1.0087589	0.0261407	0.12844468
RPL29	3887.85456	0.46672564	0.0261454	0.12844468
NLN	394.500293	-0.8078809	0.02616026	0.12848093
SFRP2	41707.8196	1.47465168	0.026186	0.12857056
ARRDC4	467.391468	-0.9155791	0.02620015	0.12860235

ZDHHC16	401.75251	-0.5469359	0.02620746	0.12860235
ZDHHC24	135.919197	-0.5357825	0.02622157	0.12863482
FUZ	174.190677	0.42017571	0.02630277	0.12895948
LACC1	275.261748	0.79940869	0.02629867	0.12895948
DCUN1D3	390.311162	0.82484351	0.02634261	0.12908268
MAP7D2	76.4642795	-1.5906929	0.02634294	0.12908268
REXO4	308.222388	-0.3082616	0.02638149	0.12923468
PPARGC1A	1894.66524	-1.3000908	0.02640196	0.12926116
TMC3	8.41433983	1.66914704	0.02639777	0.12926116
CSNK2A3	9.60357036	-0.9957657	0.02642237	0.12927083
DNALI1	102.169497	1.44579352	0.02642653	0.12927083
ZNF440	248.225268	0.81519304	0.02641983	0.12927083
BBS10	518.495246	0.53117501	0.02644015	0.12930064
ISLR2	30.7779381	-1.367892	0.02646567	0.12938856
AP3S2	81.5564471	-0.6235705	0.02647601	0.12940225
RNF212	44.1883726	-1.6223122	0.02650577	0.12951083
LINC00152	50.8115421	-1.0324013	0.02651363	0.12951235
ATG4C	217.898581	0.64656743	0.02653973	0.12956056
EDN1	56.965399	1.2997794	0.02654374	0.12956056
MYO5B	1328.13786	1.18353227	0.02655369	0.12956056
STK16	251.32778	-0.4490402	0.0265524	0.12956056
SLMAP	1525.43957	0.66903236	0.02657346	0.12962021
SUV39H2	132.940142	-0.5622442	0.02659176	0.12967262
SNX32	21.8722173	-0.9711555	0.02663838	0.12986306
DHODH	104.570369	-0.4709834	0.02665585	0.12991134
CCR7	13.5279392	1.31040322	0.0266673	0.12991759
LILRB5	52.9301036	-1.5255227	0.02667984	0.12991759
SCARB2	3846.51033	0.40179017	0.02667685	0.12991759
ENPP5	220.48256	1.10542441	0.0267351	0.13014975
TVP23A	17.6534017	1.40199166	0.02678507	0.13035601
LINC00896	13.5704273	-1.2769872	0.02682747	0.13041994
PUF60	1089.98139	-0.4332241	0.02682859	0.13041994
SOCS2	219.797799	1.44103771	0.02682429	0.13041994
TTC33	514.276452	0.64700227	0.02681242	0.13041994
LINC01277	2.90527799	1.44328987	0.02683697	0.1304237
TMEM234	151.02528	0.71607529	0.02685889	0.13049331
TMEM132B	68.2330179	1.315333	0.02686957	0.13050827
SENP7	1085.10825	0.4474892	0.02688877	0.13056456
C1QTNF3	60.8695067	0.97593585	0.0269152	0.13063138
FAM212A	48.031838	1.29132624	0.02691775	0.13063138
CAPS2	124.628869	0.74847731	0.02693345	0.13067062
LRP8	285.24082	1.1706754	0.02698131	0.13082888
QRFPR	39.2306598	1.66926607	0.02697481	0.13082888
ZFP91	1530.58333	-0.3298531	0.02699992	0.13088212
IRF5	285.578808	1.0224387	0.02701096	0.13089867
AGER	3.16438148	-1.6141216	0.02703827	0.13099405
ACSL1	1313.77681	1.01195718	0.02710801	0.1310128
AFAP1L1	4169.02289	0.86163724	0.02711083	0.1310128
C3	9254.67645	1.19511277	0.02710756	0.1310128
MZT2B	502.762536	-0.5270393	0.02708555	0.1310128

PRDM1	225.177299	0.9282395	0.02708356	0.1310128
RFX3	493.634022	0.80406698	0.02705874	0.1310128
SLC35A1	643.355725	0.55752043	0.02707403	0.1310128
SMAD6	360.050339	-0.8987057	0.02709013	0.1310128
UNG	535.857989	-0.6438963	0.02707006	0.1310128
BLOC1S3	174.09414	-0.5392464	0.02713983	0.13106548
DNPEP	474.141319	-0.4698825	0.02716632	0.13106548
DTX4	852.738061	-1.0341675	0.02713854	0.13106548
GPC5-AS1	249.442443	1.59342807	0.02716367	0.13106548
KRTCAP2	750.664643	-0.6076181	0.02714743	0.13106548
PLCD4	800.455278	0.7668688	0.02716755	0.13106548
CHCHD4	172.180228	-0.6123519	0.02718518	0.1311137
SRP68	1098.02446	-0.636997	0.02720408	0.13116801
CD300C	58.9807403	1.16835366	0.02723576	0.13128385
FGFR1OP	307.470044	0.67955951	0.02724723	0.13130227
LINC00342	759.532764	-0.6420252	0.02726457	0.13134894
RPL23AP82	62.6364037	1.0764939	0.02729769	0.13147161
CISD3	225.357964	-0.509055	0.02734705	0.13156168
CORIN	34.8153773	1.51714991	0.02734232	0.13156168
LOC1019270(	51.4485325	1.43626763	0.02734171	0.13156168
RFC4	157.228085	-0.6930188	0.0273371	0.13156168
C1orf116	3.39676163	-1.3928775	0.02735667	0.13157111
LRRC66	20.2623066	1.08957928	0.0273754	0.13162431
LSM10	142.962134	-0.7907901	0.02743494	0.13187363
C14orf39	95.5006064	1.35746582	0.02745445	0.13189356
MED30	107.568864	0.70095221	0.02744688	0.13189356
NME8	7.75790777	1.41420249	0.027487	0.13201298
TAB2	2432.58114	0.43665428	0.02752005	0.13213472
TMED4	765.373246	0.51285566	0.02753829	0.13218535
GET4	320.717127	-0.4177681	0.02761662	0.1324132
MAP3K11	741.832891	-0.5812926	0.02761243	0.1324132
RPL5	6154.23266	0.48025197	0.02761514	0.1324132
SCYL1	1259.36662	-0.3881733	0.02760114	0.1324132
BTBD1	1867.4037	-0.5804158	0.02763972	0.13248695
PKM	15583.0573	-0.9714631	0.02766943	0.13259234
RCBTB2	1125.61826	0.6596535	0.02769542	0.13267985
NEFL	1401.28629	-1.6608735	0.02773898	0.13285143
CBR1	639.048291	-0.5545544	0.02781971	0.13320092
MNAT1	362.267605	0.47803373	0.02786954	0.13340228
KIAA1586	334.583691	0.56271904	0.02789848	0.1335036
ANKDD1A	183.163574	-1.1799002	0.02792978	0.13361613
CDK2	525.918871	-0.7326746	0.02796	0.1336482
CIRH1A	429.301974	-0.7099105	0.02796763	0.1336482
PPAPDC2	237.863896	0.57454335	0.02794818	0.1336482
PRKG1	555.71171	1.28666253	0.02795916	0.1336482
TAF9B	528.672341	0.52025293	0.02798084	0.1336741
DACT2	89.1474824	1.42153642	0.02802725	0.13378412
INMT-FAM18	10.4732269	1.64401517	0.02801988	0.13378412
VIPR2	158.59896	-1.6433324	0.02802128	0.13378412
CASKIN1	104.46023	-0.7537211	0.02806546	0.133817

CCDC136	223.471054	1.26977933	0.02806607	0.133817
LOC1027241	3.21777412	-1.5428824	0.02805773	0.133817
MYLIP	546.139625	0.68728661	0.02807311	0.133817
PRADC1	201.696351	-0.6954742	0.02804637	0.133817
C1orf145	31.1917885	-1.131913	0.02812339	0.13401944
CRADD	130.732189	0.41135568	0.02815076	0.13409187
FADS1	1189.48804	-1.0202866	0.02815421	0.13409187
ANKRD24	65.7244286	-0.9230789	0.02818399	0.13412204
OR52N4	27.7441441	1.29932704	0.0281807	0.13412204
PLIN1	8.68560759	1.16312263	0.02817369	0.13412204
TBC1D10B	724.446631	-0.4377486	0.02819301	0.13412775
TMEM71	53.0735749	1.09794379	0.02823322	0.13428185
PDE2A	127.35219	1.32342593	0.02826323	0.13438734
GIMAP2	131.443311	0.87479607	0.02832917	0.13462632
UBE2O	864.288907	-0.3691834	0.02832301	0.13462632
NFIL3	355.367879	1.31830098	0.02836658	0.13476676
VPS4B	985.355189	0.52913073	0.02842384	0.13500144
ARHGEF10	1909.08057	-0.5098635	0.02844555	0.13506721
SLC35E2	556.362969	1.06552901	0.02847378	0.13516383
LEMD3	849.021618	0.38909292	0.02851856	0.13533899
CCDC15	69.708332	-0.9672936	0.02855687	0.13546605
LAMTOR4	511.833589	0.39908139	0.02856112	0.13546605
XKRX	10.6500589	1.30137603	0.02857107	0.1354758
DESI2	867.904316	-0.4899349	0.02858549	0.13550674
HSPB2	225.184373	0.72719037	0.02860982	0.13558466
NPNT	11960.2101	1.27556737	0.0286597	0.13578354
CREM	560.990565	1.01392091	0.02871761	0.13598284
ZNF493	1224.76476	0.86554112	0.02871684	0.13598284
TARS2	356.87216	-0.4595845	0.02874499	0.13607493
ATP5SL	314.964929	-0.4831649	0.02878006	0.13609613
SNORA18	25.5494916	-0.855191	0.02877618	0.13609613
ST7L	287.136146	0.47252547	0.02876221	0.13609613
TLCD1	14.4225947	-1.2810642	0.02878118	0.13609613
GLTSCR1L	888.843743	0.52405472	0.02883105	0.13629442
FAM87B	3.57163246	1.55573071	0.02886168	0.13631086
KIAA1024	105.269617	-1.0363404	0.02884421	0.13631086
LRRC45	144.190883	-0.7603254	0.02887416	0.13631086
TTC39A	5.70290568	1.64882954	0.02887424	0.13631086
ZIC1	4255.64441	-1.3202826	0.02885702	0.13631086
DPM3	133.977371	-0.6792899	0.0289153	0.13643431
LINC01237	9.10961057	1.34948229	0.02893218	0.13643431
MBNL3	1226.50627	1.02023306	0.02892968	0.13643431
ST5	902.081063	0.58403547	0.02893192	0.13643431
ABI2	1870.94608	-0.3357621	0.02894297	0.1364477
ZBP1	9.30704164	1.2666733	0.02896144	0.13649729
GBP4	325.935581	0.94243693	0.02897535	0.13652535
PHYHD1	104.591747	1.11681945	0.02899226	0.13656753
CYB5A	780.933287	0.93603594	0.02901309	0.13662817
HOGA1	169.504494	1.15479519	0.02904634	0.13674725
ACBD7	67.3037957	-1.347143	0.02905847	0.13675575

HDX	159.709148	0.43809115	0.02906408	0.13675575
DKC1	588.021589	-0.4858267	0.02908213	0.13676569
KLHL9	1313.93123	1.06148808	0.02907877	0.13676569
SEPT6	1474.79325	-1.0533407	0.02916452	0.13711556
UBXN8	138.735251	0.48534547	0.02920659	0.13727575
POM121	1079.35349	-0.5461447	0.02932237	0.13778221
RALGDS	1209.64435	-0.4151705	0.02933668	0.13781176
MAT2A	2492.74617	-0.3821451	0.02935323	0.13785176
WLS	2233.63999	0.78497711	0.02937384	0.1379108
SRPK3	3.32314113	1.64154756	0.02939411	0.13796823
RASSF5	390.038739	0.82024616	0.02944939	0.13818993
CHN1	779.871265	0.93226165	0.02949043	0.13828636
GPRASP2	31.3131175	0.94045916	0.02950039	0.13828636
LINC00909	244.442706	0.61643761	0.02948723	0.13828636
SDC1	355.6093	-0.9195655	0.02950216	0.13828636
CLGN	10.9253742	-1.4063885	0.0296497	0.13860568
GPR108	496.57534	0.60879787	0.02959653	0.13860568
IFI6	890.499067	0.51583101	0.02961305	0.13860568
ITCH	2144.30184	0.2721853	0.02962509	0.13860568
KDM1A	931.967973	0.43768739	0.0296453	0.13860568
LOC1019274	4.5231065	-1.6410688	0.02964569	0.13860568
MIAT	721.42023	-1.1537405	0.02959422	0.13860568
PEF1	689.382234	0.51640217	0.02959693	0.13860568
ZNF579	126.261602	-0.7495035	0.02965104	0.13860568
ZNF771	53.5574076	-0.6989764	0.02963628	0.13860568
NLRP1	495.597859	1.0250919	0.02967439	0.1386771
LRRTM4	2.8213555	-1.5418185	0.02969362	0.13872918
ZNF257	55.9035073	1.30597889	0.02971276	0.13878081
PPP2R2B	33.831583	1.50914771	0.02972884	0.13881814
NSUN7	87.6415093	-0.9367011	0.02975914	0.13892186
COX6B1	1377.03411	-0.576102	0.02983018	0.13909406
LINC00907	5.53591265	1.50575205	0.02983655	0.13909406
LOC1019273	14.4619229	1.44690235	0.02983122	0.13909406
NPRL3	509.796798	-0.6545911	0.02983144	0.13909406
SLC39A1	1570.04082	-0.4744396	0.0298194	0.13909406
LOC1019285	15.4658728	1.18323772	0.02993591	0.13948152
NRP2	5162.38172	1.1563006	0.02993015	0.13948152
ARL6	105.844866	0.77480496	0.02995652	0.13953966
GATAD2B	1500.56118	-0.4570724	0.02998419	0.1395928
PSMB4	1583.25293	-0.3192791	0.02997722	0.1395928
EIF2B4	293.842058	-0.39263	0.03000819	0.13966662
FAM189A2	40.2252289	1.26173822	0.03005428	0.13984323
ZFP64	451.008339	-0.5135408	0.03006943	0.13987582
SLCO4A1	76.0493673	-1.4866546	0.03009536	0.13995852
MALL	12.7561198	1.35574886	0.03010481	0.13996452
CSGALNACT1	273.654587	0.85349016	0.0301489	0.14000521
DLL3	11.880609	-1.3346865	0.03013809	0.14000521
P4HTM	381.926314	0.68311662	0.03013093	0.14000521
PEX5L	38.6329417	-1.4097299	0.03015434	0.14000521
TMED8	106.753209	0.56770162	0.0301449	0.14000521

HRSP12	110.533537	-0.5449295	0.03018599	0.14011427
GIPC2	11.8158659	1.20777786	0.03022354	0.14025062
RPS6KA5	94.2354685	0.79745007	0.03024296	0.14030282
PCOLCE2	189.30952	1.35100419	0.03026567	0.14037021
EOGT	528.415293	0.77688831	0.03029811	0.14044481
KPNB1	4314.68839	-0.3644147	0.03029481	0.14044481
FEM1C	956.58026	0.60204713	0.03032832	0.14051646
WHAMMP3	174.056069	0.58296735	0.03032994	0.14051646
NPHP3-ACAD	149.883974	0.6521908	0.03036817	0.14065561
EGFLAM-AS2	2.43277288	1.59904153	0.03040482	0.14070391
OR7E14P	2.62546388	1.61320882	0.03039694	0.14070391
SLC35C1	445.537675	-0.4273831	0.03039074	0.14070391
ZNF35	233.690519	0.55433268	0.03041139	0.14070391
IAH1	363.032966	-0.3565164	0.03043558	0.14073995
KIF26B	49.6545202	1.3736424	0.03043479	0.14073995
LINC00472	677.322576	1.32664104	0.03045669	0.14076173
ZNF540	171.702431	1.01266104	0.03045132	0.14076173
NID2	8731.96137	-0.7621195	0.03047953	0.14082937
EVC2	312.135822	0.78032594	0.03055327	0.14109414
SFTPD	7.56664619	1.60213006	0.03055181	0.14109414
SNORA40	8.92475831	-0.8862205	0.0305895	0.14122342
MSL3	379.366943	0.49233552	0.03061591	0.14130735
C5orf45	208.13965	0.56829201	0.03064027	0.14138177
CCDC89	41.6921485	-1.0306528	0.03070151	0.14149704
CITED4	33.9332195	1.17713287	0.03071666	0.14149704
FAM20B	2462.84927	-0.5586982	0.03071841	0.14149704
KDM3B	2891.46009	0.68695637	0.03068532	0.14149704
NRN1	172.087525	1.54409456	0.03071148	0.14149704
SUV39H1	109.842635	-0.6174387	0.03070318	0.14149704
TMEM115	655.152544	0.48074732	0.03072295	0.14149704
TBC1D22A	526.545499	0.32826417	0.03073188	0.14150017
LRRC8E	77.7130781	-1.132514	0.03079495	0.14175257
CCM2L	19.9832095	1.19571677	0.03083617	0.14179512
DOK1	313.7758	0.56812069	0.03084364	0.14179512
LINC00663	111.973064	0.61165693	0.03082971	0.14179512
METTL2A	295.776982	-0.4357574	0.03082975	0.14179512
ZIC2	3173.58356	-0.797895	0.0308455	0.14179512
DRP2	6.57269055	-1.0380962	0.03087526	0.14189394
SOCS2-AS1	4.31004326	1.52187355	0.03090276	0.1419823
CCIN	5.93246132	-1.3696221	0.03093266	0.14205687
EMC7	681.226789	-0.5966593	0.03093554	0.14205687
HOXD4	4.36194519	1.55466401	0.03095463	0.14210651
DLX6-AS1	22.0923722	-1.6197361	0.03098167	0.1421926
LOC1019272	51.8287675	0.56715779	0.0310336	0.14239285
DMXL2	3729.19449	-0.5227305	0.03106209	0.14248549
CCDC71L	1057.51534	0.85578093	0.03110101	0.14262594
SNAI1	26.0403498	1.39827047	0.03112019	0.14267061
STIL	103.796619	-0.9345942	0.03112738	0.14267061
MCCC2	998.787483	-0.4164844	0.03118503	0.14285857
SLC25A36	1583.09899	-0.2975911	0.03118395	0.14285857

MIEF1	1020.4694	-0.6994528	0.03121674	0.14296568
HERC1	6216.5247	0.45261224	0.03128732	0.1432507
GOLGA8A	1120.25242	0.95142858	0.03133911	0.14344957
LST1	3.21287749	1.61090663	0.03135257	0.14347291
SPATS2	442.909043	-0.6061945	0.03140837	0.14368993
PRF1	36.0560624	0.95650616	0.03144846	0.14383499
SCARNA21	347.495923	0.64920532	0.03145745	0.14383782
SPSB2	70.4395433	0.83910558	0.03148812	0.1439397
PRTFDC1	163.73813	0.91660201	0.0315005	0.14395794
ARHGEF9-IT1	1.97134937	1.55449977	0.03154245	0.14395803
FAM26E	443.138542	1.06016998	0.03153776	0.14395803
HP1BP3	2962.77127	0.4794635	0.03152428	0.14395803
TTC17	1992.56439	0.36272271	0.03153013	0.14395803
TTC38	224.882014	0.39656017	0.03151084	0.14395803
RAB9A	241.086101	0.49288436	0.03155824	0.14399182
GUF1	682.090775	-0.3240397	0.03159602	0.14412587
KANK3	36.9955377	1.10433603	0.03161022	0.14415233
TTC25	22.2360119	0.8541265	0.03167214	0.14439636
CD34	425.53472	1.11382606	0.03169314	0.14445374
HIST3H2A	82.3058981	-1.103165	0.0317947	0.14487143
KRT10	280.960712	-0.8322886	0.03180166	0.14487143
CLPTM1	1604.74821	-0.4605852	0.03182793	0.14487573
KIAA1456	15.5585976	-1.4845707	0.03182229	0.14487573
NDUFA13	1425.86559	-0.4488608	0.03182257	0.14487573
PASK	126.143523	-0.6203485	0.03185685	0.14496896
CHST3	1111.13288	-0.8434657	0.03187131	0.14497117
TNFRSF10B	1686.5381	-0.7260185	0.03187423	0.14497117
FAM71F2	119.966731	0.77598545	0.03188431	0.14497857
ZNF329	431.882612	0.42157448	0.0319133	0.14507196
TOB2P1	12.2935122	-1.2416201	0.03200012	0.14542814
FN3KRP	481.974211	-0.556313	0.03204984	0.14561553
BMPER	769.114428	1.50682551	0.03205931	0.14562003
PDE1B	86.3139016	0.82366368	0.03207451	0.1456505
RHOF	88.4466314	-1.0815976	0.03209262	0.14569421
IREB2	2433.06495	-0.6087718	0.03212292	0.14579321
RNASEH1	326.172255	-0.442349	0.03215103	0.1458822
LOC1019283	2.59491129	1.58228533	0.03221937	0.14615369
SMG5	1714.60773	-0.3994434	0.03223383	0.14618062
TUBGCP4	423.308164	-0.5183976	0.03226196	0.14626956
C20orf24	130.310474	-0.9422296	0.03229605	0.14638547
SYCP2	152.456795	1.46299579	0.03235322	0.14660585
C22orf34	30.1287166	1.10278085	0.03237531	0.14666726
ABCB9	60.2257553	-0.5839908	0.03245785	0.14684086
ARHGEF33	23.0430408	1.10479763	0.03246717	0.14684086
BBC3	148.998538	-0.7235781	0.03248728	0.14684086
BNC1	35.7011631	1.58916696	0.03249063	0.14684086
MRPS25	1212.18874	-0.6140353	0.03246868	0.14684086
NEK7	1349.82792	0.44046666	0.0324369	0.14684086
SRSF3	2570.08605	0.42102937	0.03243614	0.14684086
THOC6	176.208267	-0.5329395	0.03248905	0.14684086

UNC13D	295.162726	-0.7737058	0.032481	0.14684086
FXR1	2177.47981	-0.3740751	0.03249924	0.14684114
ZKSCAN4	118.533143	0.41910568	0.03250783	0.14684129
MIR600HG	381.840405	0.74822419	0.03252498	0.1468801
HSPG2	6103.47653	1.00027914	0.03254854	0.14690998
MTCH1	2073.56377	0.46382199	0.03254871	0.14690998
CXCR4	801.992694	0.9324363	0.03258235	0.14702314
CD74	19282.6427	0.91948307	0.03260308	0.14707801
COL6A3	9453.42959	-1.1237947	0.03261785	0.14709923
EHD2	2331.49733	-0.7617259	0.03265125	0.14709923
IL17RE	63.8147293	1.44020244	0.03264486	0.14709923
PTPRC	1434.42913	0.92440972	0.0326592	0.14709923
TFAP2E	22.9034062	1.14087902	0.03265878	0.14709923
UNKL	497.427152	-0.6070652	0.0326415	0.14709923
TTC21A	54.2625262	0.9414253	0.03268908	0.14719517
PKD1	7742.51241	-0.4616228	0.0327147	0.14727194
SPNS2	97.8322836	1.02150943	0.0327545	0.14741242
PAIP1	867.78344	0.52341451	0.03278129	0.14746847
ZNF818P	266.256066	0.83278591	0.03278413	0.14746847
FGFBP3	16.420415	-1.144492	0.03281986	0.14758202
GPAA1	1056.64554	-0.5045596	0.03283517	0.14758202
PNMA6A	14.7186516	0.87573849	0.03283269	0.14758202
FAM19A5	200.364988	-1.3430834	0.03289707	0.14768491
LAT2	299.79145	0.9060496	0.03289185	0.14768491
PRNCR1	47.8168946	-0.9703361	0.03289243	0.14768491
SNHG1	463.528231	-0.654102	0.03287074	0.14768491
WFDC6	3.60440681	1.60995372	0.03290108	0.14768491
SCAF4	813.447764	-0.5933272	0.0330092	0.14813148
MFSD1	1277.06084	0.47794819	0.03308312	0.1484244
ETV7	25.8789344	1.15033166	0.03316342	0.14866808
KIAA1147	1470.55939	0.45873735	0.0331468	0.14866808
MPC2	1590.22471	-0.7207949	0.0331549	0.14866808
MT2A	381.330153	-1.2093549	0.03317659	0.14868829
SERPINE1	443.08164	1.49305787	0.03320369	0.1487709
EIF4G3	3143.08798	0.59925792	0.03324205	0.14890389
TEC	176.38516	0.91868999	0.03326893	0.14895306
TMC4	144.369794	1.34012252	0.03327038	0.14895306
OIT3	4.17200389	1.58003957	0.03328639	0.1489859
RMST	4.95808523	-1.3572693	0.03334895	0.149227
BCL9L	1620.34382	-0.4122733	0.03339583	0.1493978
PARP6	1213.07241	-0.5531421	0.03340469	0.14939851
ACADL	598.018547	1.35739331	0.03342999	0.14947273
CLSTN2	26.851297	-1.1518979	0.03347404	0.14963071
GABRD	11.5719541	1.45494857	0.0334908	0.14966664
AQP1	3147.23794	1.47664118	0.03357957	0.14996347
POU3F1	3.04718803	-1.5176644	0.03357466	0.14996347
RAB28	284.00304	0.33134857	0.03358343	0.14996347
COASY	645.339214	-0.5426327	0.03360561	0.15002349
SLC22A8	151.985805	-1.5832274	0.03361735	0.15003688
TTC29	16.6494292	-1.5276708	0.03364804	0.15013481

FAM73A	697.978456	0.5010335	0.03366454	0.15016938
GRIA4	2.25178589	-1.4703878	0.03369066	0.15020784
RPH3AL	56.0786422	0.84028368	0.03368672	0.15020784
LINC00852	59.3870412	-0.6451408	0.03375757	0.15046709
SCAMP5	299.959289	-1.0331073	0.0338048	0.15063847
EPC1	874.560943	0.37414011	0.03383895	0.15075149
ACAD11	123.103087	0.50479282	0.03386992	0.15085033
SAP30	223.742935	0.63055746	0.03390174	0.15092911
SLC22A1	14.1757997	1.29286259	0.03390519	0.15092911
MBOAT2	1415.92131	0.79945709	0.03392776	0.15099043
PPP1R3D	201.703555	0.66070239	0.03394235	0.15101617
POM121C	898.899575	-0.5405116	0.03395406	0.15102916
CBL	1710.28067	-0.4691717	0.0339788	0.15110003
HELQ	304.96581	0.28345521	0.03398784	0.15110109
SIX4	1029.50498	0.76106666	0.03401451	0.1511805
LIPG	16.690069	1.42560357	0.03408	0.1513702
MRAP2	231.171244	1.57820347	0.03408406	0.1513702
RPH3A	2.86191446	-1.5034379	0.03410128	0.1513702
SNORA32	26.1782758	-1.2027714	0.03407928	0.1513702
ZBTB34	485.210174	-0.4026881	0.03409606	0.1513702
PLEKHA8P1	61.6806004	0.86319297	0.03411989	0.15141363
LINC00933	8.04861727	1.2374644	0.0342006	0.15173255
KIF3A	655.12718	0.82537897	0.03423205	0.1517544
POLR1C	179.291141	0.56378356	0.03423151	0.1517544
TCP11L2	340.278628	0.66423328	0.03422467	0.1517544
CEP72	73.1222805	-0.6007049	0.03426222	0.15184894
FNDC3A	4555.83769	0.48926968	0.03431124	0.15195319
NRL	8.95131651	0.9821351	0.03429574	0.15195319
WDR11-AS1	8.84831457	1.32112582	0.0343123	0.15195319
SPG20	2226.95635	0.47520722	0.0343436	0.1520526
RSRC1	440.400821	-0.4101683	0.03436107	0.15209072
CDKN2C	425.458655	-0.910188	0.03438379	0.15215204
DKFZP586I14	325.320507	0.80392922	0.03444213	0.15229272
RPS16	4490.52304	-0.9468282	0.0344422	0.15229272
ZNF728	104.714612	1.48335431	0.03443032	0.15229272
PTPRN	4.83111092	-1.5898393	0.03445571	0.15231324
SEC14L1P1	96.1857928	0.60289266	0.03446607	0.15231978
AKR1B1	959.93454	-0.3495849	0.03449581	0.15241199
C1orf35	138.398817	-0.6015741	0.03450504	0.15241353
WDTC1	992.775087	0.51914936	0.03454334	0.15254348
ENTPD1	1033.41078	0.95359987	0.03455656	0.15256258
AOAH-IT1	5.71424726	1.54063386	0.0345949	0.1526926
C9orf43	10.4146908	-0.8679429	0.03465578	0.15271527
EMCN	92.9854338	0.71967969	0.03467088	0.15271527
FSIP2	209.835877	1.54725544	0.03465761	0.15271527
INPP5E	321.782852	-0.41129	0.03467122	0.15271527
P2RX7	308.958053	0.99797321	0.03466953	0.15271527
SSR4P1	28.5356434	1.12784251	0.03464935	0.15271527
TPRN	109.244167	-0.5069473	0.03463935	0.15271527
XG	3.1348359	1.47492251	0.03464181	0.15271527

LINC00961	26.7491316	-1.2524645	0.0346822	0.15271911
SGPP1	471.400942	0.66839481	0.03468988	0.15271911
CATSPER3	10.5364286	1.05648542	0.03471843	0.15277634
COQ3	41.7352018	0.68829536	0.03473848	0.15277634
FBXO9	799.899467	0.56642611	0.03472916	0.15277634
RPUSD1	221.016651	-0.5605265	0.0347343	0.15277634
MPHOSPH6	139.548452	0.75888126	0.03478672	0.15294928
ARFGAP1	1600.32514	-0.7068717	0.03482334	0.15303189
SALL1	216.958488	-1.483261	0.03482016	0.15303189
ATP5L	1235.58151	-0.4292391	0.03484108	0.15307069
ATP6V0E1	1847.63379	0.58631508	0.03490042	0.15327266
TUBB3	157.807752	-1.4374909	0.03490491	0.15327266
ATP5O	1363.95493	-0.4167176	0.03492294	0.1533126
C9orf169	6.06582511	-1.269607	0.03493996	0.15334808
GOLGA2P7	109.549437	-0.7580056	0.03495122	0.15335831
C18orf21	144.864465	0.54622166	0.03502366	0.1536369
PWARSN	463.34341	0.48792199	0.03507084	0.15378679
TIMM50	445.620976	-0.459918	0.03507575	0.15378679
CASS4	146.230168	1.1161217	0.03510821	0.15385051
PTPN13	11648.2345	0.51152227	0.0351072	0.15385051
SIRT1	642.212488	0.3490022	0.03515963	0.1540365
RBM11	37.423797	1.41333357	0.03519209	0.15413937
PHYHIPL	10.0209174	-1.5265389	0.03522123	0.15422765
HNRNPH3	1341.5983	-0.4554313	0.03524571	0.15425614
SRPR	2631.4663	-0.3364215	0.03523837	0.15425614
SAMD1	115.174029	-0.4881065	0.03529588	0.15443636
LAMTOR3	681.801425	0.47030608	0.03538711	0.15473645
P4HA2	1359.70555	0.87400914	0.0353893	0.15473645
PRKCE	623.528996	-0.5526981	0.03539151	0.15473645
CCDC58	87.9993339	-0.5748788	0.03541201	0.15475639
RMND1	210.21202	0.47702917	0.0354141	0.15475639
MICU3	195.645758	0.85148687	0.03544841	0.15486688
SOBP	776.440183	0.57568824	0.03546235	0.15488836
NXPH3	593.391696	1.29389251	0.03547666	0.15491143
FAM228A	19.4521436	1.1866272	0.03551294	0.15499099
LINC00478	1325.64013	-1.256096	0.03550652	0.15499099
TLX1	20.4413679	1.58037128	0.03554479	0.15509059
CUEDC1	569.134839	-0.5741978	0.0355837	0.15522089
PRAM1	71.1289618	1.19232243	0.03564636	0.15545472
COL14A1	167.087832	0.96752863	0.03574713	0.15577546
RNF208	21.5956799	-0.854781	0.03573862	0.15577546
SNAP47	420.901426	-0.3048994	0.03573428	0.15577546
SNORD10	508.552181	0.65516469	0.0357946	0.15590315
TPTE2P3	1.94793797	-1.5810236	0.0357932	0.15590315
S1PR4	7.39846383	1.23867033	0.03582811	0.1560095
NEURL1	11.4539596	-1.3127489	0.03584503	0.15604361
LINC00412	13.224605	0.98556536	0.03587683	0.15614246
KLF7	1380.77502	0.52712671	0.03589091	0.15616412
KIF6	4.76493292	1.32715644	0.03591958	0.15624927
GTF2H2C_2	14.1992006	0.83192154	0.03592897	0.1562505

NDUFB3	396.835805	-0.3766351	0.03594115	0.15626389
COA4	358.341194	-0.3892484	0.03598685	0.15638336
MIR3907	2.37999851	-1.3995485	0.03598397	0.15638336
MORC4	587.628997	0.66627092	0.03606254	0.15663299
SLC44A2	4648.60202	0.64191065	0.0360559	0.15663299
PDCD1LG2	19.1991143	1.03476554	0.03615722	0.15697218
ROR1	525.419175	1.27884318	0.03615893	0.15697218
HCG11	522.288776	1.02875712	0.03617526	0.15700337
LOC1019293	83.4092115	1.56203633	0.03619839	0.15706403
EPS8L2	744.869325	-0.9011829	0.03622698	0.15713091
HAUS1	194.899187	0.68909541	0.03624126	0.15713091
YIPF6	1299.04284	0.43646951	0.03623215	0.15713091
IL20RA	63.59745	1.45127056	0.03630824	0.15733892
MROH7	31.6436137	1.4272628	0.03631658	0.15733892
ZNF799	111.815578	0.82831895	0.03631674	0.15733892
KCNQ5	10.2351465	-1.4690359	0.03636034	0.15746473
ZNF429	250.823306	0.55013004	0.03636412	0.15746473
ERP44	1161.62205	-0.3155264	0.03640591	0.15760592
YPEL2	648.689765	0.90099845	0.03643293	0.15768313
ZNF534	18.9296958	1.41201414	0.03646944	0.15780137
RIMS4	312.902817	1.32415634	0.0365018	0.15790155
MOB3B	302.789553	1.231246	0.03654816	0.15806227
DOCK7	1239.3312	0.65767269	0.03660495	0.158268
ANKRD22	177.633035	1.44472907	0.03662585	0.15831849
FAM110B	706.120658	0.66826761	0.0366897	0.15853198
NDC80	70.3675028	-1.245522	0.03669371	0.15853198
DDX31	407.968414	-0.557379	0.03670925	0.15855921
LOC1002894	5.67994531	1.28272411	0.03672855	0.15860265
GSC	3.86504639	-1.565111	0.03675943	0.15869607
CPA4	4665.73347	-1.4820379	0.03678742	0.15874221
GIMAP7	119.651303	0.82340872	0.03678861	0.15874221
RAP1A	872.7472	0.62042737	0.03688171	0.15910392
ELF4	665.590411	-0.6328495	0.03696061	0.15940424
LIMCH1	1662.6335	0.84807244	0.03701352	0.1595354
RBBP9	616.067243	0.85239703	0.03700325	0.1595354
WDR59	704.653043	-0.6231121	0.0370189	0.1595354
M6PR	1205.27439	0.5021104	0.03707155	0.15972216
FHL5	46.722178	1.36241947	0.03712117	0.15989583
CASQ2	37.5453976	1.49173275	0.03715783	0.16001357
PARL	382.973354	-0.3637879	0.03716774	0.16001611
LOC440461	4.43513812	1.48662559	0.03718124	0.16003412
TMBIM4	1128.42719	0.3923107	0.03722102	0.16016514
RPRM	42.5060364	-1.559614	0.03723463	0.16018357
MC1R	148.126311	-1.0849308	0.03724727	0.16019779
C8orf88	338.39121	1.17807772	0.03733202	0.16044754
PCBP1	2123.78036	-0.6917871	0.03733338	0.16044754
SERPINA1	415.639813	0.99961983	0.0373317	0.16044754
XYLT2	870.440145	-0.5775439	0.03734896	0.1604743
NALCN	2534.81893	0.87001844	0.03735908	0.16047764
ARMC10	538.041975	0.50726982	0.03746809	0.16082523

DYNC1I1	235.725	-1.3318962	0.03745034	0.16082523
HAP1	12.0404201	-1.2892276	0.03748593	0.16082523
SLC16A5	1002.28317	-0.9872474	0.03746856	0.16082523
SPACA6P-AS	13.3306428	0.99450851	0.03748685	0.16082523
PSMD10	436.994104	0.42430895	0.03750419	0.16085943
CEBPG	565.937503	-0.6047147	0.03758398	0.1610809
G3BP2	2621.72827	-0.5125171	0.03756603	0.1610809
SHISA2	473.494903	-1.3024522	0.03757543	0.1610809
EFNA4	91.3781261	-0.5014193	0.03761325	0.16115562
MXD1	185.325466	-0.5835473	0.03762963	0.16115562
RBKS	45.042154	0.82130925	0.03763897	0.16115562
VIPR1	82.1612199	1.05570649	0.03762583	0.16115562
ADIRF	2463.17894	1.16600293	0.03765537	0.16118563
AIM2	12.4243933	1.38288531	0.03769291	0.1612659
LOC283856	4.27689379	1.4975081	0.03769118	0.1612659
TMEM178A	41.7652347	0.98349204	0.03772266	0.16135297
ABTB1	619.761557	-1.0231316	0.03777898	0.16147317
P2RY13	430.498032	1.15865962	0.03777677	0.16147317
TMEM59	2780.3716	0.5833456	0.03777558	0.16147317
ALPL	567.504478	1.08282598	0.0378257	0.16163259
ACKR1	10.626267	1.49845636	0.03784651	0.16164106
HTRA4	9.30924466	1.19731909	0.03784494	0.16164106
PLVAP	695.311976	1.16971402	0.03786319	0.16166578
PNMA3	111.82416	1.13006081	0.03787114	0.16166578
CENPT	563.390545	-0.3836354	0.03793296	0.16188943
MED1	1858.91414	-0.3678358	0.03796726	0.16199551
ARMCX5	286.11859	0.30965357	0.03800795	0.16212885
C16orf91	94.8814198	-0.5607289	0.03806153	0.16231708
ZNF692	407.822119	-0.5225834	0.03807932	0.16235259
LOC374443	396.955485	0.66003046	0.03809934	0.16239762
PPM1M	515.227008	0.61092594	0.03812258	0.16245634
NGF	28.7600657	1.32688528	0.03813515	0.16246957
SHPK	465.347689	-0.5680679	0.03827836	0.16303922
PABPC1L	342.411489	-0.8711774	0.03830938	0.16309456
TUFM	1742.09385	-0.4118919	0.03831036	0.16309456
ENGASE	640.597545	-0.6439339	0.03836623	0.16325143
MSRB2	229.192016	0.47342667	0.0383662	0.16325143
NUGGC	5.81850368	1.46394586	0.03838416	0.16328727
EGR1	2613.89312	1.29224616	0.03842337	0.16341357
RARRES3	255.306307	0.90035571	0.03847282	0.16358333
CYP17A1	5.38629159	1.51465977	0.03850817	0.16365257
HLA-DOA	46.6862641	1.14984133	0.03849986	0.16365257
WDSUB1	201.13731	-0.4783337	0.03854212	0.16375631
KLHDC4	223.777652	-0.6510814	0.03858921	0.16378931
LEPR	24807.8775	1.36177844	0.0385976	0.16378931
SCN4B	917.928194	1.19183289	0.03856154	0.16378931
SEC63	1751.97974	0.43779711	0.03859047	0.16378931
SKIV2L2	1350.41861	0.33510538	0.03858894	0.16378931
TPH1	12.2174543	0.88231602	0.03862456	0.16386321
CTSV	155.227398	-1.4523987	0.03864724	0.16391891

METTL23	191.520565	-0.5795315	0.0386625	0.16394312
EPN2	726.562976	-0.3713671	0.03872739	0.16417772
RPSAP9	6.71453024	1.17326781	0.03875345	0.16424763
LOC1001295	7.68049453	1.11461312	0.03876713	0.16426505
CNNM3	445.146027	-0.4824065	0.03881024	0.16436657
DLX6	13.7351087	-1.5539613	0.03880929	0.16436657
CENPH	76.9021963	-0.9067781	0.03883774	0.16444249
ZFP41	428.351766	-0.5665555	0.03885287	0.16446596
PRRT3	103.947037	-0.9560866	0.03886985	0.1644973
FGR	166.184988	0.96409554	0.03897303	0.16489328
ANKRD35	32.4641397	1.31589488	0.0390109	0.16501283
POLR2J	322.339786	-0.4615039	0.03902362	0.16502597
MFSD3	128.641652	-0.6780844	0.03904327	0.16506842
KDM5B	1716.79965	-0.4103611	0.03913557	0.1654179
KDM2A	2669.94715	-0.2780708	0.03917565	0.16554652
BNIP3L	3691.17528	0.58378457	0.03919575	0.1655907
MAP4K2	423.188213	-0.4496874	0.03923277	0.16566555
RAB8B	1795.33869	0.72779465	0.03922369	0.16566555
GPATCH8	2483.12801	-0.4277951	0.03924481	0.16567563
CARF	543.39684	0.49975754	0.03928362	0.16570043
DNAJC9	342.18941	-0.4418019	0.03929895	0.16570043
RIOK1	436.795803	0.55607997	0.0392978	0.16570043
SORCS2	1332.12695	-1.3693937	0.03929221	0.16570043
SRRM5	10.2606234	-1.0561187	0.03926701	0.16570043
AES	5276.26631	0.51881109	0.0393552	0.16586929
CD163L1	85.9318021	-0.9150233	0.03935832	0.16586929
ZNF277	361.622878	0.45581914	0.03937829	0.1659127
SNTB2	2100.79693	0.70045831	0.03951871	0.16646348
DNA2	107.030363	-0.6876712	0.03953959	0.16651056
BARD1	280.954893	-0.7016319	0.03954958	0.1665118
SAE1	964.988413	-0.4108527	0.0395695	0.16655481
SIX1	2645.73737	0.77986526	0.03964145	0.16681676
RPS9	4708.7241	0.49496342	0.03967321	0.16690945
GABBR1	7.53120248	1.54445098	0.0397079	0.1670145
ITGA6	2194.24368	-0.6852634	0.03974781	0.16714139
MBNL1-AS1	74.6330146	0.68160804	0.03977815	0.16722802
IPW	444.251067	0.63718833	0.03979544	0.16725971
RPN2	5098.3232	-0.5246556	0.03984811	0.16739912
ZNF557	521.969977	0.49888944	0.03984072	0.16739912
EFCAB1	19.033604	1.41966361	0.03987894	0.16747346
MYOM1	61.812216	0.95535311	0.0398922	0.16747346
NR4A1	2110.15436	1.35544971	0.03989984	0.16747346
PI4KB	1645.63428	-0.3058753	0.03990483	0.16747346
PFKFB3	1505.75213	0.7633741	0.0399179	0.16748734
FBXL3	1704.41715	0.49830752	0.03993963	0.16749665
KCTD5	330.503198	-0.4914347	0.03993762	0.16749665
COMMD3	183.159754	0.44158239	0.03996153	0.16750368
PRG1	4.29696062	1.54941336	0.03995922	0.16750368
SCAMP1	1286.83292	0.52699844	0.03997058	0.16750368
LOXL3	293.54708	1.16771032	0.04001099	0.16759117

RBM28	501.333455	-0.3821497	0.04000884	0.16759117
GALNT18	299.236283	-0.8355348	0.0400352	0.16765166
LYRM7	554.508142	0.65433938	0.04009731	0.16787081
LINC00887	132.778752	-1.4595004	0.04014601	0.16791088
NSG1	261.037855	-1.3904511	0.04013419	0.16791088
PYROXD1	362.684506	0.78547136	0.04013891	0.16791088
TMEM68	238.818587	0.44606061	0.04013375	0.16791088
KCTD2	917.841807	-0.4320312	0.04017761	0.16800212
CAMK2B	22.4851656	1.30233172	0.04022042	0.16814014
EMILIN1	1272.14646	1.04882344	0.04023195	0.16814741
PTEN	3042.38628	0.35554351	0.04024911	0.1681782
MAP2K6	102.044251	-1.0386826	0.04026077	0.16818597
ZNF551	417.911509	-0.4004567	0.04036038	0.16856104
CAPN10	264.288625	-0.5073286	0.04037052	0.1685624
ANAPC4	506.573495	0.28527509	0.04039244	0.1686129
NEGR1	11.6924607	1.49195337	0.0404335	0.16874325
C20orf197	23.6448047	1.20755414	0.04049262	0.16881865
CACNA2D2	395.028132	1.20283169	0.04051057	0.16881865
KIAA1462	6078.12074	0.84395393	0.04048408	0.16881865
LRRC8C	1658.71835	0.76198808	0.04046376	0.16881865
MPHOSPH9	678.328814	-0.3573624	0.04048032	0.16881865
SERPIND1	333.087784	1.42210229	0.04050727	0.16881865
IPP	267.699529	0.64735296	0.04054275	0.1689117
RAD54L2	573.301682	0.34713934	0.0405552	0.16892258
COPB1	2502.22725	0.34932343	0.04062026	0.16899276
HEATR6	1008.00742	-0.4196981	0.04059632	0.16899276
LIN9	87.1558606	-0.6580482	0.04062127	0.16899276
SCGN	2.85797627	1.52628067	0.04061125	0.16899276
ZNF75D	513.586793	0.39135419	0.04061375	0.16899276
AKAP11	4954.8661	0.47401766	0.04064762	0.16906138
FAM65A	954.952129	-0.4731353	0.04068938	0.16918762
STPG1	99.024897	0.78867151	0.04069768	0.16918762
APP	25906.9923	-0.9022798	0.0407629	0.16941771
DOC2A	32.7498229	-1.0611799	0.04078973	0.16944717
OPA3	866.001683	-0.4520011	0.04078847	0.16944717
ERBB2IP	4069.9461	0.33753074	0.04083697	0.16960238
AFAP1-AS1	590.381018	-1.518759	0.04086664	0.16964063
CPSF7	1586.47466	-0.2986291	0.04087583	0.16964063
TMUB1	343.008927	-0.6109222	0.04086823	0.16964063
LINC01114	12.2523568	1.53049503	0.04088578	0.16964091
RAPGEF1	1912.05948	0.39191394	0.04095115	0.16987106
UNC5A	3.02626144	-1.3723207	0.04096337	0.1698807
RPS4X	12337.7808	0.63855466	0.04099036	0.16995158
KIAA0247	4312.53002	0.7416564	0.04101202	0.17000033
MMP10	2.2712243	-1.53514	0.04103951	0.17007319
APH1B	710.955273	0.75796484	0.04105047	0.17007757
LIME1	123.393493	-0.6392351	0.04111534	0.17026455
LOC1019295	5.12140002	1.3366799	0.04111544	0.17026455
ZNF558	348.021042	0.46822119	0.04113651	0.1703107
SLC35F6	1147.50176	-0.4563458	0.04114921	0.17032219

LY86	127.65483	0.96982751	0.04117382	0.17038298
LILRA1	75.6352283	1.12695363	0.04118547	0.17039013
NR1D1	222.263978	-1.1494683	0.04123969	0.17057333
RNPS1	1274.21858	-0.3840592	0.04129076	0.17070227
SELPLG	489.514112	1.02815924	0.04128577	0.17070227
UBOX5	333.535126	0.46688297	0.04131282	0.17075237
EPHB3	155.967735	1.18182884	0.04136078	0.1708683
ZNF114	55.5185539	-1.1416571	0.04135297	0.1708683
KCNG1	5.98132477	-1.538171	0.04142219	0.17099854
LOC10050750	25.418436	-0.7274538	0.04141997	0.17099854
SLC22A11	1.955803	1.52327374	0.04140353	0.17099854
ZNF573	228.119316	0.53302389	0.04143494	0.17101005
OTUB1	665.515843	-0.4158878	0.04149457	0.17121496
UBE2B	744.991136	0.7774133	0.04151156	0.1712439
BIRC6	7667.55871	-0.3166979	0.04157367	0.17145892
EXPH5	187.0097	-1.1447648	0.04159831	0.17151935
CADM1	723.782529	0.69016318	0.04162388	0.17154237
RRP15	495.911358	-0.3199627	0.04161573	0.17154237
HLA-DRB5	920.637497	1.49729815	0.04163601	0.17155115
APPL1	1760.21167	0.52247831	0.04165861	0.17160306
ZNF623	612.16227	-0.4267892	0.04167755	0.17163992
ENPP6	10946.3657	1.23415306	0.041769	0.17197529
EAF2	65.4473724	0.76295567	0.04184156	0.17219139
FAM83H	546.143964	-1.2634266	0.04183339	0.17219139
LOC10254629	28.7990236	1.15285796	0.04187451	0.17228571
FOXP1	5993.78581	0.54165724	0.04190279	0.17236073
ACAT1	765.589409	-0.5586381	0.041962	0.17238557
GPC2	32.2743508	-0.896616	0.04194728	0.17238557
KBTBD4	305.4465	0.54142861	0.04196803	0.17238557
PALD1	1306.86787	0.87091083	0.04196908	0.17238557
RHOBTB1	834.748312	0.97811316	0.04195039	0.17238557
TMA16	221.685245	-0.4277766	0.0419636	0.17238557
LOC10012839	62.2680769	-0.8676218	0.04198673	0.17241678
RNPEPL1	643.962762	-0.5585723	0.04200314	0.17244293
TRIM68	276.011518	0.66074968	0.04202465	0.17248999
GSTA4	404.545198	0.65806924	0.04203656	0.17249762
PDCD4-AS1	44.6128679	-0.9800492	0.04205156	0.17251792
THUMPD3-AS	278.207697	-0.5648122	0.04206918	0.17254898
LOC10050660	34.532796	0.9093181	0.04209779	0.17256544
NAA30	501.424422	0.42344263	0.04210335	0.17256544
NPFF	31.9434915	0.84439591	0.04208929	0.17256544
BCKDHA	514.090957	-0.5157055	0.0421429	0.17263775
CSNK2A2	439.549014	-0.4658864	0.04214457	0.17263775
FASTK	701.786603	-0.4194599	0.04215117	0.17263775
AARD	12.7613555	-1.4264821	0.04219451	0.17277402
CREG1	1597.29348	0.38086866	0.04224291	0.17293096
MDC1	5.60883635	1.5247718	0.04228374	0.17305683
KCTD3	1177.64365	-0.3562583	0.04231517	0.1731442
PRMT2	2667.39086	0.4754315	0.04232571	0.17314604
HMCN1	1057.6856	1.41032288	0.04240667	0.17327075

PIK3C2B	3060.74544	-0.7830566	0.04238279	0.17327075
PPM1L	107.649262	0.82459264	0.04238662	0.17327075
SMAP2	1232.70844	0.88270967	0.04239497	0.17327075
WDR19	735.063706	0.67732205	0.04240509	0.17327075
HMHA1	808.029036	0.9215165	0.04245427	0.17342397
ANPEP	1062.57855	-0.8637699	0.04247787	0.17344894
MPP7	76.0425742	0.9998992	0.04248059	0.17344894
YIPF2	540.629973	0.36435344	0.04251378	0.17354319
CCNG1	4310.29495	0.55517678	0.04257101	0.17369419
LOC1019272	9.98329843	1.18347465	0.04256382	0.17369419
EIF1AD	336.316946	-0.3905252	0.0426028	0.1737413
STARD8	579.873647	0.82700567	0.04259894	0.1737413
KIAA1033	2303.6069	0.37096474	0.04263297	0.17378176
SOX6	66.8760069	-1.2392871	0.04262829	0.17378176
TMEM110	1280.18314	0.94222523	0.04269754	0.17400362
AGXT	5.33087884	-1.5290749	0.04272918	0.17405961
KBTBD11	579.10082	-0.9690425	0.0427417	0.17405961
MCUR1	300.274599	-0.5157947	0.04273669	0.17405961
LRIT3	13.2064488	1.06365194	0.04277886	0.174134
SAP130	537.8456	-0.4616971	0.04278025	0.174134
LTV1	328.511772	0.44648272	0.0428252	0.17427564
FAM195A	128.313032	-0.7511763	0.04287002	0.17439715
PPP1R37	389.043674	-0.3738727	0.04287539	0.17439715
ATP2A1	26.3764114	-0.8808947	0.04295285	0.17456023
CDKN2A	41.3267864	-1.4411512	0.04295616	0.17456023
ROMO1	341.92189	0.50408556	0.04292604	0.17456023
STAT2	4214.37454	0.51381139	0.04294744	0.17456023
LRRN2	184.161091	-1.4136204	0.04296894	0.17457084
CD7	12.6382089	1.12965998	0.04301897	0.17473276
CCDC102A	266.13445	-0.689926	0.04304889	0.17481293
GPR126	413.050086	1.0312631	0.04307384	0.17487288
RND2	63.5727814	1.16027893	0.04310966	0.1749769
BBS4	525.368856	-0.3928448	0.04315122	0.17510414
C2orf48	2.28443318	-1.4210973	0.04316658	0.17510414
KLHDC2	810.645567	0.61460318	0.04318074	0.17510414
YTHDC2	1378.94146	0.66730129	0.04318181	0.17510414
TOPORS	739.94946	0.45414064	0.04325326	0.17535242
ATXN3	730.371528	0.43337213	0.04327382	0.17539436
FIGNL2	13.1999833	1.33640715	0.04330697	0.17540446
PDP1	1229.68121	0.5562724	0.04329659	0.17540446
RNASE1	1953.60753	-1.2056814	0.04330243	0.17540446
ENDOD1	2492.40223	-0.5264429	0.04342907	0.17579458
GZMA	20.628336	1.27192603	0.04343401	0.17579458
PARK7	1048.0417	0.32044021	0.04342794	0.17579458
LOC1001909	29.9906551	-0.646073	0.04346136	0.17583639
NAA16	592.820596	0.54267004	0.04346483	0.17583639
STXBP5L	2.46052767	-1.4844181	0.04348013	0.17585684
PTAFR	320.500061	0.91036399	0.04351372	0.17595125
GUCY1A2	152.787075	1.14904079	0.04355365	0.17607123
SEC16B	154.573806	0.80498611	0.04362288	0.17630958

GALNT11	1227.96572	0.54473862	0.04366103	0.17638487
NCKAP5L	1114.42887	-0.5044742	0.04366206	0.17638487
CCDC24	54.6123503	0.71952527	0.04367953	0.17640544
SCAF11	3960.79203	0.42374421	0.04368771	0.17640544
C12orf60	9.85311761	0.88022966	0.0437688	0.17645354
CHST2	307.091	-1.085275	0.04377158	0.17645354
DAPK2	129.117883	1.18284359	0.04375897	0.17645354
DOK5	209.706011	1.32497402	0.04373382	0.17645354
TFB1M	115.634474	0.42199472	0.04372239	0.17645354
TNXB	11.2837391	-1.2202685	0.04374592	0.17645354
UQCRC1	1339.97628	-0.5643637	0.04373753	0.17645354
RLF	736.533822	0.61546119	0.04381446	0.17658494
ASH1L-AS1	64.5609255	-0.6660855	0.04384184	0.1766221
CENPL	112.087397	-0.6392787	0.04385586	0.1766221
GARS	1080.10636	-0.5305102	0.04384467	0.1766221
KDM3A	1109.5824	-0.6070527	0.04387716	0.1766221
SLC22A5	560.283808	0.8485466	0.04388542	0.1766221
SPIN1	3111.01308	-0.2832177	0.04387265	0.1766221
KMT2B	1363.70388	-0.4388735	0.04391298	0.17663985
POLM	337.92371	0.4871434	0.04392318	0.17663985
RANGAP1	647.098572	-0.5768373	0.043931	0.17663985
TIGD1	217.935263	-0.5770797	0.04392319	0.17663985
RAB7A	2961.5909	-0.4870824	0.04394295	0.17664656
FECH	519.411749	0.48438921	0.04398104	0.17671688
IDH2	348.728304	-0.6147639	0.04397111	0.17671688
SNX18	1512.89005	0.3839576	0.04406088	0.17699624
CD6	22.4349473	1.11105189	0.04410269	0.17712274
SNTA1	660.029565	0.67094788	0.04422517	0.17753157
ZSCAN16-AS	100.340074	0.71726732	0.044216	0.17753157
ATAD1	1251.65299	-0.4499272	0.04444015	0.17822783
FAM208A	2721.96725	0.38358126	0.04442042	0.17822783
SLC38A4	48.2293512	1.4382721	0.04443263	0.17822783
ZNF230	135.138499	0.63582994	0.04443254	0.17822783
AASDHPPT	758.215053	-0.2822748	0.04447564	0.17826368
FBN1	12879.3895	-0.7804791	0.04449063	0.17826368
GLI3	733.212528	0.90014248	0.04448829	0.17826368
ZHX2	698.12487	-0.5401193	0.04446969	0.17826368
MTERFD2	536.356555	-0.4238523	0.04452246	0.1783496
CSTA	42.8692518	-1.2669615	0.04453935	0.17837562
CETN4P	2.80180518	1.48365897	0.0445815	0.1785028
CAD	1548.03767	-0.5499761	0.04459384	0.17851055
MID2	674.405016	0.73934216	0.04461247	0.17854351
CD226	27.8047755	0.85575542	0.04466524	0.17862974
LINC00899	30.4571817	0.7557026	0.04465702	0.17862974
OPLAH	431.827803	-1.1089785	0.04466179	0.17862974
RTN2	87.6909492	-0.8114061	0.04468044	0.17864891
ACE	196.662473	1.00098375	0.04470154	0.17865833
F13A1	1931.41061	-0.9440558	0.04470361	0.17865833
N4BP2	1092.28885	-0.4745013	0.04476584	0.17879969
OXCT1	995.009991	0.70826371	0.04477023	0.17879969

VPS9D1	320.963545	-0.6020199	0.04476992	0.17879969
CASP8	413.629765	-0.5398356	0.04481611	0.17889966
FAM173A	88.9396509	-0.4742336	0.04481585	0.17889966
SNORA63	1398.52223	-0.6309827	0.04485342	0.17900697
CNTRL	907.537561	0.7893359	0.04487628	0.17905657
CIITA	820.819158	1.01271375	0.04500571	0.17940619
OR7D2	11.6492796	1.45584614	0.0449958	0.17940619
PCDH19	638.412316	-1.3890396	0.04499792	0.17940619
ZBTB38	2196.59695	0.42579654	0.04500259	0.17940619
PTGS1	366.477538	0.84694074	0.0450887	0.17969529
LOC1001334	8.28256578	1.21850782	0.04517204	0.17995097
SCN11A	22.4614504	1.17212271	0.04517383	0.17995097
ADCY2	1368.01986	1.01675372	0.04521489	0.18004626
ZNF418	378.983107	0.42214035	0.04521872	0.18004626
CHPF	3090.50259	-1.0410436	0.04529076	0.18020766
SLC25A12	781.038842	-0.5427474	0.04527003	0.18020766
ZNF496	411.578057	-0.5520774	0.04528705	0.18020766
ANGPTL5	54.7136993	1.50727084	0.04533624	0.18030503
TMX2	1005.52654	-0.4271737	0.04532959	0.18030503
MRPL13	193.512373	-0.3986293	0.04535401	0.18033393
CD96	32.1195667	0.99402129	0.04541472	0.18053349
SAP18	1601.7633	0.57879654	0.04544256	0.18060235
ATXN7	939.445923	0.45948079	0.04548256	0.18066341
C19orf57	15.2044265	-1.0160437	0.04550726	0.18066341
GOLGB1	7406.01579	0.18487261	0.04551055	0.18066341
PANK2	496.373721	0.39237754	0.04549176	0.18066341
TSFM	297.787427	-0.231584	0.04550464	0.18066341
DDX25	36.3302253	-1.2481667	0.04553629	0.18068201
LOC1005067	15.1085995	-1.106649	0.04553537	0.18068201
HK1	1553.64705	-0.7424404	0.04556201	0.18074231
RGPD1	4.90611412	-1.4906522	0.04559123	0.18081643
LINC00304	4.38955483	-1.3647801	0.04561667	0.18087552
DIRC3	119.22569	0.98795573	0.0456435	0.18091859
NEK6	940.837541	-0.6727382	0.04564861	0.18091859
B3GNT1	836.991407	0.50543598	0.04568409	0.18101741
CSNK1D	2207.14805	-0.6770555	0.04572398	0.18109185
RBP1	1019.23264	-1.3163937	0.04572042	0.18109185
FAM95C	7.98956277	-1.48087	0.04576538	0.18121401
MAP7D3	389.149742	1.0632898	0.04578277	0.18124108
RAB6A	2626.78302	-0.2975405	0.04580089	0.18127097
CACNA1A	57.0054151	-1.2095762	0.04584523	0.18132106
TMEM216	125.896322	-0.4618234	0.04582825	0.18132106
ZDHHC2	425.899773	0.59302105	0.04583478	0.18132106
CDH10	34.9389669	-1.3714773	0.0458776	0.18140362
FKBP8	2428.66031	0.31099354	0.04588724	0.18140362
PINK1-AS	186.253016	0.54697937	0.04589849	0.1814063
BRCA1	244.424204	-0.7524546	0.04600421	0.18175823
OSGEP	290.343451	0.47825	0.04600871	0.18175823
HAUS7	399.267545	0.71240156	0.04601956	0.18175924
MATN3	304.914825	-1.3350708	0.04605954	0.18183348

PRSS12	38.0720705	-1.4120865	0.04605605	0.18183348
FAM182B	7.75917229	1.25526317	0.04613005	0.18203377
REC8	150.679269	0.96795191	0.04614117	0.18203377
UNC5CL	24.2877313	0.84202854	0.04614209	0.18203377
SKP1	3938.92779	0.69650353	0.04619814	0.18221304
KDM4A	1020.63291	0.46861426	0.04626884	0.18241138
RNH1	1864.58299	0.5427323	0.04626969	0.18241138
ANO3	29.1479099	1.43821299	0.04632377	0.18241513
PTGIR	9.82565696	1.22300393	0.04632083	0.18241513
RPL17	1494.71379	0.56579625	0.04628286	0.18241513
ZKSCAN3	228.122212	0.65066777	0.04629217	0.18241513
ZNF76	463.447919	0.60124917	0.04631054	0.18241513
LOC1005062	19.2735949	1.12175354	0.04634778	0.1824678
C1orf112	150.261313	-0.4772337	0.04639296	0.18249732
CCRL2	20.4032135	0.98950657	0.04638401	0.18249732
DDN	4.83223474	-1.3518866	0.0463978	0.18249732
DSTN	3365.0772	0.67692233	0.04639676	0.18249732
ADAMTS17	701.281483	-1.0817774	0.04641297	0.18251516
LINC01018	323.190905	1.15482715	0.04646292	0.18266974
LOC220729	459.10466	-0.4415841	0.04650309	0.18274395
MIOS	461.397041	0.38628866	0.04649439	0.18274395
MYO3A	117.387108	-1.4478184	0.04653724	0.18282487
PCSK5	648.401682	-1.051992	0.04654498	0.18282487
NUDCD1	269.343471	-0.4030068	0.04656355	0.18285593
CEP131	204.950222	-0.5939486	0.04657946	0.18287657
C1orf53	8.18111669	-1.3036232	0.04667544	0.18307434
DCBLD2	1327.73823	-0.7165876	0.04666479	0.18307434
LIMD1-AS1	9.12434147	1.28145084	0.04664506	0.18307434
NFIX	2503.99486	0.92628742	0.04668316	0.18307434
NPAP1	6.25157672	1.49780807	0.04666714	0.18307434
MAT2B	908.051937	0.56435022	0.04670431	0.18311546
NEFH	36.9559593	1.39506342	0.04674019	0.18321369
TMEM179B	450.407776	-0.4170192	0.04675071	0.18321369
FRS2	1523.09774	0.29216842	0.04677929	0.18326589
TAPT1	432.319408	0.33819303	0.04678539	0.18326589
MARK1	521.20461	-0.8474311	0.04680281	0.18329232
AMICA1	137.412824	0.98785856	0.0468412	0.18335898
GPBP1L1	1359.99014	0.47211191	0.04683672	0.18335898
FREM1	1669.00369	-0.8399928	0.04687494	0.18340743
NLGN4X	56.4084435	-1.4649264	0.04687462	0.18340743
CD300LG	12.5140252	1.41992296	0.04690723	0.18340835
STK11	828.17159	0.56270659	0.04689485	0.18340835
TACR2	2.86128814	1.44336314	0.04690212	0.18340835
LOC1019269	4.16136354	1.29356275	0.04695673	0.18354851
SLC24A4	32.8253851	1.20411219	0.04696447	0.18354851
DDX56	757.493519	0.46803483	0.0469874	0.18359636
CACNA1F	12.2673716	-1.1950785	0.04701883	0.18362987
ELOVL4	67.1141601	1.15233673	0.04703686	0.18362987
MRPL9	348.991868	-0.369121	0.04703889	0.18362987
PPP1R36	10.8147805	1.21535211	0.04703122	0.18362987

ZNF24	1897.79754	0.46320387	0.04704947	0.18362987
LINC00173	35.6218727	-1.1672232	0.04713189	0.18386794
SEPHS2	496.158848	-0.7194523	0.04712148	0.18386794
GAS5-AS1	15.4741918	-0.674577	0.04717129	0.18397981
HAUS2	447.655134	0.43566839	0.04718704	0.18399945
MID1IP1-AS1	4.12528723	-1.298808	0.04723037	0.18408222
NDUFV1	1449.50136	-0.3757577	0.04724044	0.18408222
STAT4	20.6214955	1.05275484	0.04722905	0.18408222
MRO	340.626717	-1.115901	0.04725669	0.18410376
LOC1019272	4.20326142	1.49313876	0.04732746	0.18433761
SIRT7	138.730609	-0.6035766	0.04734116	0.18434914
TTC26	180.355434	0.62731576	0.04736465	0.18439877
MOAP1	461.837313	0.55881077	0.0473763	0.18440231
PDZD7	50.0164085	0.90474718	0.04741326	0.18450434
PDGFB	426.123167	0.72601414	0.04742625	0.18451304
ELN	3907.216	1.35083898	0.04748045	0.18468206
ZNF492	15.0434003	1.02864442	0.04756414	0.18496566
MEIS1	25.4646188	-1.2108833	0.04760039	0.18498091
TMCC1-AS1	77.6975453	-1.0339453	0.04759543	0.18498091
ZNF280B	58.0035755	-1.1342711	0.04759073	0.18498091
LOC1019271	3.61998486	1.31699388	0.04762435	0.18503215
EEPD1	415.251271	-1.0327365	0.04766248	0.18509648
SOS1	1968.66417	-0.2573852	0.04765275	0.18509648
LOC1009964	15.3718993	1.31742184	0.04768266	0.18513296
DMBX1	11.4493873	-1.4761965	0.04772247	0.18520375
ZNF663P	21.3658478	1.0783639	0.04771477	0.18520375
FGF7	455.323648	1.43744093	0.04773348	0.1852046
IRF7	138.668738	0.56277722	0.04778902	0.18529445
PRELID2	109.934402	0.85921887	0.04777724	0.18529445
STK17B	730.470562	0.84962988	0.04778725	0.18529445
CLIC5	1224.09727	1.39836969	0.04781313	0.18534606
CCDC124	352.233065	-0.6759398	0.04786768	0.18551562
KCNMB4	51.95995	1.07811757	0.04794322	0.18576645
PERM1	2.86806994	1.45852737	0.04797842	0.1858609
FAM102A	1448.59094	-0.6942295	0.04800456	0.1859202
RHBDL1	25.63259	-0.7940517	0.04805763	0.18608376
NACA	6606.87491	0.38748758	0.04809608	0.1861906
TBX21	6.34053246	1.30832632	0.04820263	0.18651896
ZNF614	338.579305	0.37619003	0.0481959	0.18651896
MTX1	211.315139	-0.4339559	0.04833236	0.18697881
TUBB1	13.8998921	1.22990984	0.04834563	0.18698801
BTBD19	117.172888	1.06758735	0.04841785	0.18722514
ALG1L	11.7183206	1.23433823	0.04844572	0.18727041
CLNS1A	1052.71232	-0.2483101	0.04845138	0.18727041
ACTR1B	990.398666	-0.4199489	0.04849661	0.18740303
UNK	894.243569	-0.4496675	0.0485101	0.18741297
C12orf29	181.983759	0.38961214	0.04857887	0.18755883
SLC27A1	839.157907	-0.4590038	0.04858063	0.18755883
SNX24	468.761182	0.68117154	0.04856966	0.18755883
FNTA	585.165467	0.41370825	0.04860868	0.18758273

GABPB1-AS1	655.446376	-0.5869917	0.04860279	0.18758273
LDLRAD4-AS	5.30396785	1.27144963	0.04866904	0.18777345
LOC1005065	772.017487	0.63836857	0.04868544	0.18779373
NRXN3	397.889538	1.06596305	0.04869618	0.18779373
HERC2P7	52.0610545	-0.6108343	0.04872379	0.187858
NAIP	465.607938	0.54969751	0.04875068	0.18791947
COQ10A	111.51998	-0.5204936	0.04880128	0.18799372
GAK	1326.58165	-0.3832021	0.04880847	0.18799372
LOC644762	2.09050049	-1.3782965	0.04880488	0.18799372
SF3A2	561.212167	-0.5229997	0.04881375	0.18799372
CARNS1	13.6236894	-1.2949635	0.04885303	0.18806062
PPFIBP2	474.090458	0.9673118	0.04884749	0.18806062
C3orf35	29.0771023	0.88251267	0.04889013	0.18812341
FAM228B	154.261459	0.72979002	0.04889126	0.18812341
TIPIN	86.93965	-0.4911964	0.04894977	0.18830633
RPL11	5533.53936	0.35527993	0.04898533	0.18840091
BMP8A	20.6159621	1.26853455	0.04907145	0.18863607
HEMK1	565.947826	0.50229489	0.04907945	0.18863607
NR6A1	142.903049	-0.8361443	0.04906115	0.18863607
TRNAU1AP	168.089339	0.4736298	0.04919829	0.18905052
MME	206.817806	1.32163701	0.04921661	0.1890786
IFI35	258.840986	0.60273506	0.04936233	0.18955357
PPARA	551.776334	0.32929869	0.04935806	0.18955357
LOC1019295	9.92928919	1.41153706	0.0494174	0.1897226
BET1L	846.935595	0.36975851	0.04942981	0.18972777
ACY3	11.0699588	1.38196004	0.04944455	0.18974193
ZNF853	256.012989	-0.4117335	0.04945902	0.18975504
C19orf52	160.250706	0.46468728	0.04948018	0.18979382
BCKDHB	524.657289	0.65096339	0.0495139	0.18988071
APLP1	285.368886	-1.01717	0.04955277	0.18998734
ADAMTS13	229.564135	-0.793791	0.04959559	0.19010904
ARFIP2	473.142953	0.42004512	0.04964758	0.19020139
FCHSD1	309.227541	0.65696109	0.04965292	0.19020139
MT1X	207.295402	-1.1593723	0.04963861	0.19020139
GPT2	128.024481	-0.7802697	0.04970057	0.19034143
LOC1006528	13.0307773	1.46568801	0.04977182	0.19057178
DIXDC1	2146.16405	-0.8137645	0.0498474	0.19081861
RNF187	1472.48569	-0.4112573	0.04994653	0.19115544
