# Supplementary material for: Transcriptome signatures associated with meningioma progression
Source: Acta Neuropathol Commun. 2019 Apr 30;7:67. doi: 10.1186/s40478-019-0690-x (PMC6489307; doi:10.1186/s40478-019-0690-x)
Supplement: Supplementary file 5 — Table S4. List of significantly differentially expressed genes between all grade I and grade II-III meningiomas, as identified by RNA-seq. (PDF 581 kb) [file 40478_2019_690_MOESM5_ESM.pdf]

**Supplementary Table 4: Differentially expressed genes between GR I and GR II/III meningiomas**

| Gene | baseMean | log2FoldChar | pvalue | padj |
|---|---|---|---|---|
| SNORA54 | 50.7952454 | 3.19328883 | 2.79E-14 | 4.92E-10 |
| FOXC2 | 1389.28921 | -2.0191834 | 3.32E-13 | 2.92E-09 |
| KIF18B | 81.0640303 | -2.6077351 | 3.33E-12 | 1.95E-08 |
| ADCY5 | 4074.23155 | -1.387015 | 1.30E-10 | 5.49E-07 |
| HJURP | 59.5483004 | -2.2349815 | 1.56E-10 | 5.49E-07 |
| FAM111B | 102.093607 | -2.2998221 | 3.01E-10 | 8.82E-07 |
| CDT1 | 58.4799344 | -2.1300611 | 4.79E-10 | 1.06E-06 |
| SAPCD2 | 34.0228708 | -1.9902187 | 4.83E-10 | 1.06E-06 |
| MKI67 | 845.240137 | -2.0930668 | 6.10E-10 | 1.19E-06 |
| CYB5R4 | 145.174574 | 0.88664356 | 1.54E-09 | 2.70E-06 |
| RPL21 | 3.5798149 | 2.97407395 | 2.58E-09 | 4.13E-06 |
| MYBL2 | 67.1024003 | -2.4627485 | 4.51E-09 | 6.61E-06 |
| H2AFX | 338.080292 | -1.4034867 | 5.12E-09 | 6.93E-06 |
| FOXM1 | 166.438157 | -2.1536478 | 5.82E-09 | 6.95E-06 |
| TONSL | 134.679437 | -1.5091557 | 5.93E-09 | 6.95E-06 |
| SLC12A1 | 40.2181579 | 2.8337392 | 8.12E-09 | 8.93E-06 |
| CIT | 380.131202 | -1.8608533 | 1.32E-08 | 1.37E-05 |
| GSG2 | 18.6302025 | -2.040642 | 1.89E-08 | 1.81E-05 |
| INMT | 2897.36131 | 2.85688335 | 1.95E-08 | 1.81E-05 |
| IQGAP3 | 201.292766 | -2.0213912 | 2.32E-08 | 2.04E-05 |
| CENPF | 562.612635 | -1.8298353 | 2.49E-08 | 2.08E-05 |
| LRFN1 | 73.5216663 | -1.4388648 | 3.10E-08 | 2.48E-05 |
| CDCA8 | 29.5819194 | -1.5510073 | 3.89E-08 | 2.98E-05 |
| SAMD5 | 366.169468 | 2.71600725 | 4.24E-08 | 3.01E-05 |
| TROAP | 60.5998905 | -2.1361303 | 4.28E-08 | 3.01E-05 |
| GATA3 | 37.2947269 | -2.4900474 | 5.40E-08 | 3.61E-05 |
| MEX3A | 58.6719381 | -2.1564204 | 5.54E-08 | 3.61E-05 |
| XKR5 | 15.3259131 | -2.3931121 | 6.41E-08 | 4.03E-05 |
| NCAPH | 60.1493133 | -1.6773279 | 8.08E-08 | 4.58E-05 |
| TACC3 | 246.1329 | -1.3366706 | 7.92E-08 | 4.58E-05 |
| TUBB4A | 26.9385879 | -2.7239338 | 8.00E-08 | 4.58E-05 |
| DHRS9 | 4.24834164 | 2.49258227 | 8.80E-08 | 4.84E-05 |
| TOP2A | 732.282545 | -1.7132954 | 9.15E-08 | 4.88E-05 |
| BIRC5 | 92.8476479 | -2.0314323 | 1.08E-07 | 5.55E-05 |
| TK1 | 128.862046 | -1.9470042 | 1.11E-07 | 5.55E-05 |
| LINC00312 | 16.5307872 | 2.35238172 | 1.29E-07 | 6.27E-05 |
| SNORA48 | 389.539786 | 1.43209678 | 1.32E-07 | 6.27E-05 |
| HMMR | 68.5502586 | -1.8065463 | 1.55E-07 | 7.17E-05 |
| FAM57B | 2.99769318 | -2.6544397 | 1.76E-07 | 7.75E-05 |
| RABGAP1L | 2205.84068 | 1.02370006 | 1.73E-07 | 7.75E-05 |
| DHCR7 | 568.394165 | -1.5367659 | 2.15E-07 | 9.22E-05 |
| EXO1 | 42.4647914 | -1.9403281 | 2.31E-07 | 9.68E-05 |
| HES7 | 9.36868329 | -1.8864776 | 2.68E-07 | 0.00010975 |
| CKAP2L | 68.3373256 | -1.8879325 | 3.16E-07 | 0.00011346 |
| CKMT1B | 11.953546 | -2.5984347 | 3.00E-07 | 0.00011346 |
| FASN | 2898.41727 | -1.2648917 | 2.96E-07 | 0.00011346 |
| SLC27A2 | 15.8595456 | 2.453663 | 3.11E-07 | 0.00011346 |

| | | | | |
|---|---|---|---|---|
| SNAP23 | 772.171453 | 0.47721192 | 3.08E-07 | 0.00011346 |
| WDR62 | 59.2474009 | -1.6448774 | 2.94E-07 | 0.00011346 |
| ASF1B | 63.2437152 | -1.5293387 | 3.27E-07 | 0.00011499 |
| RRM2 | 178.652762 | -1.9549914 | 3.75E-07 | 0.00012918 |
| E2F8 | 17.1744304 | -2.1115437 | 3.87E-07 | 0.00013081 |
| HIST1H2BO | 105.534224 | -1.6126726 | 4.41E-07 | 0.00014641 |
| LMCD1 | 155.889899 | 2.22831354 | 4.57E-07 | 0.00014901 |
| HIST1H2AL | 96.4948165 | -1.5557106 | 4.84E-07 | 0.00015493 |
| ATP2B2 | 114.190322 | -2.5454491 | 5.60E-07 | 0.00017116 |
| ATP6V0C | 983.993406 | -0.9302583 | 5.48E-07 | 0.00017116 |
| SGOL1 | 34.2664882 | -1.7740314 | 5.64E-07 | 0.00017116 |
| C9orf172 | 19.9189371 | -2.2023237 | 5.76E-07 | 0.00017187 |
| NUSAP1 | 264.027404 | -1.64722 | 6.50E-07 | 0.00019054 |
| E2F7 | 42.8751019 | -1.7744777 | 6.95E-07 | 0.00020051 |
| ARHGEF39 | 50.3718679 | -1.8967444 | 7.87E-07 | 0.0002122 |
| ASPM | 332.709337 | -1.9462479 | 7.91E-07 | 0.0002122 |
| DTL | 145.009427 | -1.706446 | 8.08E-07 | 0.0002122 |
| NNMT | 196.758136 | 2.26694158 | 8.07E-07 | 0.0002122 |
| SPC25 | 19.8197849 | -2.0919087 | 7.75E-07 | 0.0002122 |
| SPP1 | 3548.40948 | -2.1503879 | 7.70E-07 | 0.0002122 |
| SV2A | 205.711431 | -2.2793344 | 8.41E-07 | 0.00021744 |
| MYO7B | 12.5469678 | 2.25994347 | 9.07E-07 | 0.00023134 |
| ZNF777 | 239.322581 | -0.5801578 | 1.12E-06 | 0.00028178 |
| HNRNPUL2 | 568.168082 | -0.6195489 | 1.15E-06 | 0.00028376 |
| PIK3R2 | 1089.03598 | -0.743256 | 1.17E-06 | 0.00028502 |
| CD48 | 36.3051201 | 1.83646959 | 1.24E-06 | 0.00029494 |
| MRPL12 | 298.694681 | -1.3480755 | 1.23E-06 | 0.00029494 |
| BUB1 | 103.226377 | -1.6509057 | 1.39E-06 | 0.000321 |
| BUB1B | 114.891189 | -1.7124025 | 1.37E-06 | 0.000321 |
| CCDC106 | 223.61957 | -0.7673853 | 1.49E-06 | 0.00034149 |
| GVINP1 | 136.640853 | 1.21231104 | 1.56E-06 | 0.00034667 |
| MTRNR2L9 | 38.3214333 | 1.49372369 | 1.55E-06 | 0.00034667 |
| MYCL | 73.6429696 | 1.95419987 | 1.84E-06 | 0.00040427 |
| DDX60 | 674.970675 | 0.89307928 | 1.87E-06 | 0.00040629 |
| PDE1C | 104.418305 | 2.25029012 | 2.03E-06 | 0.00043498 |
| SNORA68 | 57.1790241 | 1.5811485 | 2.05E-06 | 0.00043498 |
| UPK3B | 149.517152 | -2.1201051 | 2.36E-06 | 0.00049384 |
| MELK | 84.1834083 | -1.7930083 | 2.43E-06 | 0.00050356 |
| CHRM1 | 96.566855 | -2.3320687 | 2.86E-06 | 0.00058036 |
| KIF20A | 96.8379339 | -1.7149335 | 2.87E-06 | 0.00058036 |
| LINC00649 | 8.94301587 | 2.20981188 | 3.10E-06 | 0.00061887 |
| CCNB2 | 60.7162711 | -1.8210265 | 3.31E-06 | 0.0006539 |
| HRCT1 | 84.7859688 | -1.6046925 | 3.37E-06 | 0.00065854 |
| CEP55 | 65.3702194 | -1.6929429 | 3.47E-06 | 0.00066417 |
| RECQL4 | 88.9841249 | -1.4096165 | 3.45E-06 | 0.00066417 |
| ANLN | 275.450442 | -1.6103234 | 3.61E-06 | 0.00068309 |
| LRCH4 | 1261.32814 | -0.7584794 | 3.77E-06 | 0.00070465 |
| BCAR3 | 236.926807 | 1.8897876 | 4.21E-06 | 0.0007804 |
| FLG | 16.1383328 | -2.230276 | 4.39E-06 | 0.00079535 |
| TIMELESS | 356.9589 | -0.8792598 | 4.37E-06 | 0.00079535 |

| | | | | |
|---|---|---|---|---|
| DPP6 | 121.779828 | -2.3300298 | 4.94E-06 | 0.00086082 |
| ESPNP | 1.71492126 | -2.3367279 | 4.99E-06 | 0.00086082 |
| KIF4A | 66.8081293 | -1.7436639 | 4.94E-06 | 0.00086082 |
| OR7E2P | 12.1334293 | 2.20803651 | 4.97E-06 | 0.00086082 |
| PLK1 | 63.9920114 | -1.5336953 | 4.89E-06 | 0.00086082 |
| APLP2 | 19398.266 | -1.0494163 | 5.20E-06 | 0.00088094 |
| PAX2 | 7.79189112 | 2.31099709 | 5.24E-06 | 0.00088094 |
| TMEM223 | 133.900336 | -0.7571325 | 5.26E-06 | 0.00088094 |
| FAM83D | 29.7931544 | -1.8746654 | 5.54E-06 | 0.00091926 |
| NUF2 | 32.885971 | -1.503286 | 5.74E-06 | 0.0009432 |
| CDCA5 | 60.6547559 | -1.6246869 | 5.88E-06 | 0.00095535 |
| UBAP2L | 2312.57921 | -0.5555536 | 5.92E-06 | 0.00095535 |
| OLIG1 | 11.3046264 | -2.2795658 | 6.01E-06 | 0.00095962 |
| PAQR4 | 77.5044507 | -1.281895 | 6.06E-06 | 0.00095962 |
| CDC20 | 33.7788291 | -1.6728236 | 6.36E-06 | 0.00098891 |
| IRF4 | 10.4847206 | 1.62332017 | 6.40E-06 | 0.00098891 |
| NEK2 | 28.2347138 | -1.6526384 | 6.41E-06 | 0.00098891 |
| LOC1019269 | 1.68356295 | 2.31201506 | 6.86E-06 | 0.00104857 |
| SNORA46 | 3.7947037 | 2.11791035 | 6.97E-06 | 0.00105762 |
| HIST1H2AI | 113.200208 | -1.3957637 | 7.10E-06 | 0.00106722 |
| CENPA | 13.1785921 | -2.0271622 | 7.72E-06 | 0.00113524 |
| COG8 | 375.945812 | -0.5479158 | 7.74E-06 | 0.00113524 |
| NCBP2-AS2 | 141.447313 | -0.6433662 | 7.66E-06 | 0.00113524 |
| GLI4 | 97.8714642 | -0.8516818 | 8.00E-06 | 0.00116266 |
| SLC31A2 | 257.075902 | 1.16184994 | 8.11E-06 | 0.00116985 |
| STARD5 | 43.2399513 | 1.86674065 | 8.26E-06 | 0.00118169 |
| BST2 | 192.297066 | 1.18187489 | 8.39E-06 | 0.00118814 |
| CDC25A | 61.5241314 | -1.3906176 | 8.53E-06 | 0.00118814 |
| KIAA1161 | 329.040779 | -1.7524767 | 8.58E-06 | 0.00118814 |
| LIX1 | 28.392782 | -2.122695 | 8.51E-06 | 0.00118814 |
| CCDC85B | 93.9505045 | -0.7352903 | 9.11E-06 | 0.00125177 |
| UBE2C | 80.7483119 | -1.7938763 | 9.25E-06 | 0.00126182 |
| KIF14 | 85.8147612 | -1.6000775 | 9.59E-06 | 0.00129825 |
| HIST1H2AJ | 80.8144191 | -1.5149477 | 9.81E-06 | 0.00131777 |
| RRN3P2 | 59.9623176 | 1.54163806 | 1.04E-05 | 0.00138695 |
| SLC29A1 | 1198.71716 | 1.36193672 | 1.05E-05 | 0.00138695 |
| ZDHHC23 | 67.7081576 | -1.836697 | 1.06E-05 | 0.00138695 |
| C19orf54 | 143.740222 | -0.910079 | 1.10E-05 | 0.00141286 |
| CRTAM | 6.68232808 | 1.89041712 | 1.10E-05 | 0.00141286 |
| KIF11 | 199.972869 | -1.4469048 | 1.11E-05 | 0.00141286 |
| MYPOP | 57.3529436 | -0.7552698 | 1.10E-05 | 0.00141286 |
| C10orf32 | 344.754718 | 0.93756411 | 1.15E-05 | 0.0014257 |
| EZH2 | 175.620944 | -1.1671562 | 1.14E-05 | 0.0014257 |
| HIST1H3B | 254.45393 | -1.4854918 | 1.14E-05 | 0.0014257 |
| PPP1R14B | 555.529416 | -1.0711047 | 1.13E-05 | 0.0014257 |
| DRD2 | 21.0921337 | 2.23908006 | 1.22E-05 | 0.00150179 |
| RPL10 | 5775.59675 | 0.69326202 | 1.29E-05 | 0.00156322 |
| SPRED1 | 1436.66948 | 0.45045419 | 1.29E-05 | 0.00156322 |
| EHMT1 | 1085.72985 | -0.5100029 | 1.37E-05 | 0.0016343 |
| TYRP1 | 6.20261738 | -2.2314795 | 1.36E-05 | 0.0016343 |

| | | | | |
|---|---|---|---|---|
| CDR2L | 217.311967 | -1.1704891 | 1.41E-05 | 0.001654 |
| CEACAM21 | 14.2185924 | 1.42545736 | 1.40E-05 | 0.001654 |
| RIPK3 | 25.6285416 | 1.27704678 | 1.40E-05 | 0.001654 |
| GLDC | 228.420038 | -1.6471388 | 1.44E-05 | 0.00167206 |
| ALAS2 | 7.81130824 | 1.95004045 | 1.47E-05 | 0.00169571 |
| RGS6 | 62.5657692 | 2.18770353 | 1.48E-05 | 0.00169665 |
| OGFRL1 | 1104.58312 | 0.92140738 | 1.49E-05 | 0.00170447 |
| ARRDC1 | 309.173779 | -0.6320918 | 1.53E-05 | 0.00173698 |
| OTX1 | 7.63324393 | -2.1559024 | 1.54E-05 | 0.00173774 |
| BAALC | 101.365098 | -2.0829846 | 1.64E-05 | 0.00183854 |
| DAAM2 | 389.514326 | 1.57110657 | 1.72E-05 | 0.00185482 |
| FTH1 | 12588.9789 | -0.9131303 | 1.71E-05 | 0.00185482 |
| PKMYT1 | 29.4323405 | -1.6506085 | 1.70E-05 | 0.00185482 |
| SNORA81 | 311.790334 | -0.9368512 | 1.69E-05 | 0.00185482 |
| ST8SIA1 | 126.725692 | 2.16872629 | 1.70E-05 | 0.00185482 |
| UST | 684.19328 | 1.14460366 | 1.69E-05 | 0.00185482 |
| GREM2 | 11.4756329 | 2.20562936 | 1.75E-05 | 0.00186797 |
| PRRC2C | 5930.24806 | -0.4488682 | 1.75E-05 | 0.00186797 |
| CABLES2 | 239.12275 | -1.0638635 | 1.78E-05 | 0.00188511 |
| NKD1 | 855.781993 | 2.13780194 | 1.79E-05 | 0.00188615 |
| TEKT3 | 9.345895 | 1.63047831 | 1.82E-05 | 0.00190158 |
| NCAM2 | 29.5336792 | -1.953319 | 1.95E-05 | 0.00202662 |
| FAM46A | 921.050535 | 1.21512577 | 1.96E-05 | 0.00203106 |
| PCSK1N | 329.989338 | -1.681595 | 2.01E-05 | 0.00205759 |
| TRIP13 | 50.1865887 | -1.568866 | 2.02E-05 | 0.00205759 |
| VIT | 369.803382 | 2.12292305 | 2.02E-05 | 0.00205759 |
| KIF13B | 702.11344 | 0.78673211 | 2.11E-05 | 0.00213413 |
| GPR123 | 2.86475031 | -2.1168612 | 2.14E-05 | 0.00215496 |
| CKMT1A | 10.4537221 | -2.1677327 | 2.25E-05 | 0.00219097 |
| CKS2 | 73.269701 | -1.3273956 | 2.25E-05 | 0.00219097 |
| IL18R1 | 51.8857673 | 1.46740379 | 2.24E-05 | 0.00219097 |
| JPH1 | 61.1368504 | -1.9588885 | 2.23E-05 | 0.00219097 |
| NBPF25P | 23.6781919 | 1.03998409 | 2.19E-05 | 0.00219097 |
| RNF214 | 414.411754 | -0.5110835 | 2.23E-05 | 0.00219097 |
| PDLIM5 | 2922.47757 | 0.81620545 | 2.32E-05 | 0.0022304 |
| PM20D2 | 266.454432 | 1.11244589 | 2.31E-05 | 0.0022304 |
| CDH1 | 7416.46071 | -1.1337317 | 2.34E-05 | 0.00223276 |
| PRF1 | 20.4740786 | 1.46142177 | 2.35E-05 | 0.00223538 |
| MAG | 22.9844483 | -2.1658099 | 2.38E-05 | 0.00225146 |
| RSPO3 | 265.405679 | 2.16357322 | 2.43E-05 | 0.00228698 |
| AKT1S1 | 520.582512 | -0.6929408 | 2.46E-05 | 0.00229923 |
| FAM180B | 36.3400992 | 2.16366104 | 2.47E-05 | 0.00229923 |
| EBP | 194.377924 | -0.8935254 | 2.52E-05 | 0.0023205 |
| MME | 102.358415 | 2.02086911 | 2.51E-05 | 0.0023205 |
| CD300LF | 11.1613717 | 1.64242723 | 2.55E-05 | 0.00233344 |
| AGR2 | 339.096519 | -2.1616941 | 2.60E-05 | 0.00237295 |
| IGSF5 | 17.4725539 | -1.9063363 | 2.63E-05 | 0.00237689 |
| NEFM | 58.9800755 | -2.1186521 | 2.63E-05 | 0.00237689 |
| TFG | 1377.68901 | -0.6463344 | 2.67E-05 | 0.00239888 |
| CCDC146 | 94.7600199 | 1.07388294 | 2.69E-05 | 0.00240177 |

| | | | | |
|---|---|---|---|---|
| CNIH2 | 9.57425482 | -1.6034805 | 2.70E-05 | 0.00240281 |
| OAZ1 | 2656.88785 | 0.49834615 | 2.73E-05 | 0.00240968 |
| C11orf30 | 598.755316 | -0.4375495 | 2.77E-05 | 0.0024404 |
| AKAP7 | 153.797385 | 1.13857575 | 2.83E-05 | 0.00246469 |
| FAM127B | 346.929004 | -0.7635246 | 2.86E-05 | 0.00246469 |
| GPR171 | 4.82824115 | 1.7508721 | 2.85E-05 | 0.00246469 |
| PID1 | 245.669167 | 1.86673188 | 2.85E-05 | 0.00246469 |
| CRAT | 780.023758 | -0.6689415 | 2.89E-05 | 0.00248053 |
| KIF5C | 121.516293 | -1.856606 | 2.91E-05 | 0.00248626 |
| DNAJC30 | 263.666376 | -0.6416231 | 2.95E-05 | 0.00250739 |
| ESPL1 | 83.4718255 | -1.4882705 | 2.96E-05 | 0.00250739 |
| MARC2 | 142.103579 | -1.1762083 | 3.02E-05 | 0.00254377 |
| COMMD10 | 212.520962 | 0.82849081 | 3.12E-05 | 0.0025888 |
| DSE | 2115.92783 | 0.91974067 | 3.11E-05 | 0.0025888 |
| HIST1H3C | 133.160862 | -1.3711253 | 3.12E-05 | 0.0025888 |
| LYST | 1465.70295 | 0.83722814 | 3.20E-05 | 0.00264438 |
| CXCR6 | 11.4929546 | 1.79993447 | 3.23E-05 | 0.0026586 |
| AURKB | 23.7697381 | -1.5154242 | 3.31E-05 | 0.00268179 |
| GIT1 | 1233.59455 | -0.7963723 | 3.29E-05 | 0.00268179 |
| SNRNP200 | 5330.60279 | -0.5725524 | 3.28E-05 | 0.00268179 |
| ALG3 | 270.503864 | -0.7953828 | 3.33E-05 | 0.00268937 |
| MIR3648 | 145.903221 | -2.0079559 | 3.36E-05 | 0.00270218 |
| KIAA0101 | 51.3030554 | -1.6630666 | 3.47E-05 | 0.0027759 |
| MEX3B | 56.1812173 | -1.6379148 | 3.52E-05 | 0.00279989 |
| CCDC126 | 176.472019 | 1.24320283 | 3.64E-05 | 0.00287329 |
| MCM10 | 35.7492435 | -1.5727973 | 3.64E-05 | 0.00287329 |
| PVALB | 93.3821155 | -2.0700755 | 3.66E-05 | 0.00287329 |
| TGFB3 | 560.995495 | 1.36145479 | 3.68E-05 | 0.00287786 |
| COL8A2 | 1112.8174 | 1.66944671 | 3.73E-05 | 0.00290669 |
| UBXN7 | 835.164915 | -0.3377697 | 3.96E-05 | 0.00306887 |
| FCER1A | 15.5023216 | 2.00904294 | 4.02E-05 | 0.00309823 |
| LOC1005073 | 167.337807 | -1.8338699 | 4.15E-05 | 0.00317529 |
| RHOD | 251.7704 | -0.8276442 | 4.19E-05 | 0.00317529 |
| SOX9 | 130.471956 | -2.0449262 | 4.17E-05 | 0.00317529 |
| SYT10 | 3.96003044 | -2.0930193 | 4.14E-05 | 0.00317529 |
| TBC1D32 | 267.697258 | 0.85650066 | 4.33E-05 | 0.0032719 |
| ZNF865 | 271.36676 | -0.880526 | 4.40E-05 | 0.00330995 |
| TEX41 | 6.43724474 | 1.7183386 | 4.43E-05 | 0.00331672 |
| SPAG5 | 182.366099 | -1.2178437 | 4.46E-05 | 0.00332383 |
| CDCA3 | 27.9288671 | -1.3410722 | 4.57E-05 | 0.003391 |
| POLQ | 80.5866274 | -1.4616995 | 4.64E-05 | 0.00342727 |
| ATG9A | 953.023008 | -0.6125039 | 4.70E-05 | 0.00345653 |
| KCNA6 | 73.2853661 | 1.98570697 | 4.78E-05 | 0.00350091 |
| PDHA1 | 836.588917 | -0.6586191 | 4.86E-05 | 0.00354864 |
| FST | 29.3481701 | 2.03006208 | 4.90E-05 | 0.003563 |
| PRKAA2 | 559.486348 | 1.14580665 | 5.01E-05 | 0.00362948 |
| UBQLN4 | 435.830843 | -0.6680334 | 5.05E-05 | 0.00364147 |
| BVES-AS1 | 2.39489742 | 2.0377413 | 5.13E-05 | 0.0036844 |
| CHCHD10 | 119.93423 | -1.5460665 | 5.17E-05 | 0.00369444 |
| ELOVL6 | 288.436023 | -1.0103559 | 5.23E-05 | 0.00372554 |

| | | | | |
|---|---|---|---|---|
| NBPF9 | 52.2523015 | 0.98087403 | 5.34E-05 | 0.00378535 |
| SLC28A1 | 4.29683427 | 1.99865023 | 5.41E-05 | 0.0038185 |
| SLC25A5 | 1613.91431 | -0.7272247 | 5.47E-05 | 0.00384905 |
| CPQ | 1355.16841 | 0.83559376 | 5.75E-05 | 0.0040318 |
| UHRF1 | 99.6174098 | -1.2521605 | 5.84E-05 | 0.00407947 |
| RAD51 | 33.5782143 | -1.4630289 | 5.93E-05 | 0.00410971 |
| TIMP2 | 13394.5311 | -1.0407824 | 5.93E-05 | 0.00410971 |
| TMEM242 | 167.470984 | 0.6722053 | 6.06E-05 | 0.0041782 |
| FBN3 | 95.2977436 | -1.8891479 | 6.09E-05 | 0.00418431 |
| CLEC16A | 1044.04424 | -0.7290124 | 6.12E-05 | 0.00418953 |
| PCLO | 410.065128 | -1.4863789 | 6.19E-05 | 0.00422027 |
| ZDHHC12 | 138.678433 | -0.8717801 | 6.28E-05 | 0.00426319 |
| CENPU | 87.1856044 | -1.2386323 | 6.41E-05 | 0.00429078 |
| LZIC | 201.633309 | 0.65786555 | 6.42E-05 | 0.00429078 |
| MACC1 | 45.4093662 | 1.39139746 | 6.35E-05 | 0.00429078 |
| TMEM37 | 319.807196 | -1.246242 | 6.40E-05 | 0.00429078 |
| CASC5 | 198.434647 | -1.3458046 | 6.57E-05 | 0.00431964 |
| ESRRG | 26.8381243 | -2.0361324 | 6.52E-05 | 0.00431964 |
| MEN1 | 371.550326 | -0.6144141 | 6.50E-05 | 0.00431964 |
| MYRF | 52.9594018 | -1.9296068 | 6.58E-05 | 0.00431964 |
| PRAF2 | 265.261773 | -0.7559087 | 6.57E-05 | 0.00431964 |
| WNT6 | 865.164438 | -1.5158792 | 6.74E-05 | 0.00440996 |
| TMEM140 | 127.707968 | 0.90823712 | 7.26E-05 | 0.00472848 |
| C16orf59 | 15.7295677 | -1.5462018 | 7.33E-05 | 0.00475944 |
| ZNF462 | 1318.09156 | -0.5324813 | 7.39E-05 | 0.00477946 |
| THBS1 | 3272.91554 | 1.83427492 | 7.46E-05 | 0.00480743 |
| INCENP | 270.370027 | -0.6101697 | 7.57E-05 | 0.00485875 |
| COX5A | 541.80078 | -1.0990473 | 7.67E-05 | 0.00489102 |
| WDR66 | 26.7580177 | 1.36008424 | 7.65E-05 | 0.00489102 |
| BCAN | 5.73249389 | -2.0137073 | 7.89E-05 | 0.00497323 |
| KATNA1 | 125.783625 | 0.58850767 | 7.84E-05 | 0.00497323 |
| ZNF367 | 182.280284 | -1.1346577 | 7.87E-05 | 0.00497323 |
| DAPP1 | 34.2941552 | 1.48317886 | 8.11E-05 | 0.00507165 |
| FBXO41 | 423.202272 | -0.9473254 | 8.12E-05 | 0.00507165 |
| FGF7 | 229.605366 | 1.96699608 | 8.13E-05 | 0.00507165 |
| CCR2 | 19.7584521 | 1.66502375 | 8.27E-05 | 0.00512761 |
| PIWIL2 | 12.5818086 | 1.6059227 | 8.31E-05 | 0.00512761 |
| SESN1 | 1445.00515 | 1.15608296 | 8.28E-05 | 0.00512761 |
| SLC27A6 | 59.0761017 | -2.0045168 | 8.40E-05 | 0.00516429 |
| FLJ22184 | 16.1699142 | -1.6502576 | 8.45E-05 | 0.00517647 |
| SLC52A3 | 23.9363324 | -1.9598946 | 8.48E-05 | 0.00517772 |
| ZNF136 | 281.106161 | 0.73730799 | 8.51E-05 | 0.00517802 |
| UFSP1 | 18.8595752 | -1.2600169 | 8.65E-05 | 0.00524924 |
| TMEM71 | 31.5320569 | 1.39575805 | 8.70E-05 | 0.00526104 |
| DLAT | 670.595031 | -0.688207 | 8.87E-05 | 0.00534384 |
| CCDC62 | 7.42325815 | 1.3298651 | 9.08E-05 | 0.00544817 |
| TGIF1 | 328.288533 | 0.7988463 | 9.23E-05 | 0.00552108 |
| LOC1002890 | 166.362292 | -1.2125769 | 9.32E-05 | 0.00555972 |
| COQ9 | 710.198822 | -1.0236323 | 9.49E-05 | 0.0056023 |
| FAM222A | 17.933353 | -1.2529535 | 9.52E-05 | 0.0056023 |

| | | | | |
|---|---|---|---|---|
| RTP4 | 28.9686581 | 1.35479415 | 9.49E-05 | 0.0056023 |
| SMG7 | 1802.67069 | -0.7283352 | 9.47E-05 | 0.0056023 |
| LRFN4 | 213.521887 | -1.4276789 | 9.57E-05 | 0.00560963 |
| C9orf72 | 243.181113 | 0.8559946 | 9.73E-05 | 0.00566854 |
| UBE2Z | 1700.62018 | -0.4014566 | 9.73E-05 | 0.00566854 |
| NCAPG | 117.069425 | -1.4571243 | 9.82E-05 | 0.00567946 |
| ZMYND19 | 146.160019 | -0.6512032 | 9.80E-05 | 0.00567946 |
| APBB2 | 3408.65417 | 0.9745954 | 9.93E-05 | 0.00568737 |
| FABP7 | 5.08655889 | -1.9976343 | 9.92E-05 | 0.00568737 |
| RSAD2 | 117.875292 | 1.18931741 | 9.91E-05 | 0.00568737 |
| TEX36 | 11.7558724 | -1.7025179 | 9.97E-05 | 0.00569189 |
| AGBL1 | 65.0639658 | -1.984122 | 0.00010048 | 0.00572 |
| LMNB1 | 198.243813 | -1.074809 | 0.00010111 | 0.00573511 |
| PSKH1 | 714.493095 | -0.6341934 | 0.0001014 | 0.00573511 |
| SYT1 | 25.6917807 | -1.874263 | 0.00010257 | 0.00578257 |
| FAM189B | 392.032837 | -1.0076961 | 0.00010426 | 0.00585924 |
| BANF1 | 457.605743 | -0.8724491 | 0.00010643 | 0.00590199 |
| HCN2 | 58.7038159 | -1.6339585 | 0.00010559 | 0.00590199 |
| LOC1005075 | 72.6661634 | 1.20703012 | 0.00010612 | 0.00590199 |
| MAP3K5 | 596.352752 | 1.19285589 | 0.00010651 | 0.00590199 |
| TAF1A-AS1 | 15.1303148 | -1.1459154 | 0.0001067 | 0.00590199 |
| LOC1006535 | 102.166809 | -1.0523943 | 0.00011022 | 0.00607754 |
| PIGR | 16.6089573 | 1.97642802 | 0.00011063 | 0.00608097 |
| PCYT1A | 502.460279 | -0.3982214 | 0.00011369 | 0.00622966 |
| PRR14 | 291.323827 | -0.5740449 | 0.00011438 | 0.0062482 |
| BST1 | 120.344152 | 1.57565974 | 0.00011586 | 0.00630101 |
| PROC | 2.28232483 | -1.9656599 | 0.00011673 | 0.00630101 |
| SERPINI1 | 55.6598327 | 1.30349696 | 0.00011608 | 0.00630101 |
| ZNF672 | 319.863488 | -0.7152926 | 0.00011678 | 0.00630101 |
| GIN1 | 121.185957 | 0.65654779 | 0.00011735 | 0.00631232 |
| SLC6A6 | 881.456994 | 1.35270165 | 0.00011822 | 0.00633966 |
| ZNF620 | 63.0210166 | -0.744258 | 0.00011885 | 0.00635428 |
| LOC1005068 | 12.8711807 | 1.15633382 | 0.00011922 | 0.00635498 |
| TESK1 | 397.669135 | -0.6949335 | 0.00012155 | 0.00645576 |
| ZNF467 | 170.797125 | -1.1739981 | 0.00012185 | 0.00645576 |
| FAM105A | 924.117731 | 1.12593947 | 0.0001227 | 0.00646222 |
| UBALD2 | 229.608995 | -0.588905 | 0.00012265 | 0.00646222 |
| CXorf22 | 23.6285936 | -1.915154 | 0.00012598 | 0.00654264 |
| HAPLN4 | 55.9645956 | -1.7602596 | 0.00012609 | 0.00654264 |
| IMMT | 1231.05069 | -0.5854557 | 0.00012464 | 0.00654264 |
| SQRDL | 489.363498 | 0.75143209 | 0.00012505 | 0.00654264 |
| TBC1D17 | 549.955146 | -0.4108706 | 0.00012552 | 0.00654264 |
| NDUFA8 | 318.248749 | -0.7156648 | 0.00012658 | 0.00654887 |
| CCNYL1 | 509.464347 | -0.6941949 | 0.00012966 | 0.00668807 |
| STK31 | 20.2289013 | 1.4242463 | 0.00013011 | 0.00669173 |
| POLI | 395.978981 | 0.79503536 | 0.00013067 | 0.0067009 |
| TPI1P2 | 14.7744256 | -1.2494453 | 0.0001318 | 0.00673935 |
| DENND5B | 451.472325 | 0.81267986 | 0.00013291 | 0.00675681 |
| HSD11B1 | 15.5189228 | 1.9623083 | 0.00013271 | 0.00675681 |
| NAV1 | 4831.43604 | -1.2462141 | 0.00013357 | 0.00677104 |

| | | | | |
|---|---|---|---|---|
| GTF2E1 | 192.393595 | -0.4906111 | 0.00013618 | 0.00686408 |
| KCNS2 | 4.20395567 | -1.942567 | 0.00013619 | 0.00686408 |
| C11orf84 | 224.346657 | -0.6133481 | 0.00013677 | 0.00687375 |
| AP1S2 | 253.222327 | 1.03878608 | 0.00013879 | 0.00693549 |
| FSIP2 | 107.839853 | 1.91431437 | 0.00013852 | 0.00693549 |
| USP45 | 246.585227 | 0.66309201 | 0.00014032 | 0.00699203 |
| TEAD2 | 301.693732 | -0.9371278 | 0.00014096 | 0.00700415 |
| PRRX1 | 1829.93048 | 1.69979064 | 0.00014231 | 0.00705153 |
| ECT2L | 6.37056898 | 1.67400408 | 0.00014552 | 0.0071677 |
| HPR | 24.2702459 | 1.86315386 | 0.00014588 | 0.0071677 |
| TMEM63C | 4.77884541 | -1.8168387 | 0.00014573 | 0.0071677 |
| PMS2P5 | 110.465706 | -0.6068234 | 0.00014722 | 0.0072133 |
| AIM1 | 130.762604 | 1.13108009 | 0.0001542 | 0.00721505 |
| ARHGAP23 | 2179.87123 | -0.6636683 | 0.00014962 | 0.00721505 |
| CGN | 32.566676 | -1.599882 | 0.0001528 | 0.00721505 |
| EPHA3 | 212.681755 | -1.7319762 | 0.00015293 | 0.00721505 |
| FBRSL1 | 576.01492 | -0.7556971 | 0.00015346 | 0.00721505 |
| FOXK2 | 832.222547 | -0.5984467 | 0.0001519 | 0.00721505 |
| INTU | 289.605265 | 0.95293486 | 0.00015423 | 0.00721505 |
| KCNE1 | 17.2076683 | 1.67783481 | 0.00014917 | 0.00721505 |
| LY75 | 28.050055 | 1.34732875 | 0.00015039 | 0.00721505 |
| PLSCR1 | 281.480473 | 0.5780889 | 0.00014938 | 0.00721505 |
| PPP5C | 761.089928 | -0.4413249 | 0.00015054 | 0.00721505 |
| PRCC | 557.396445 | -0.4922176 | 0.00015258 | 0.00721505 |
| PRR11 | 145.367974 | -1.3187197 | 0.00015357 | 0.00721505 |
| RMI1 | 99.4802141 | -0.6986817 | 0.00014902 | 0.00721505 |
| SMAD6 | 448.617935 | -1.2271272 | 0.00015243 | 0.00721505 |
| TNC | 402.402724 | 1.8493607 | 0.0001531 | 0.00721505 |
| ZNF812 | 88.9351678 | 1.66853239 | 0.00015282 | 0.00721505 |
| ECH1 | 763.27479 | -0.650323 | 0.00015695 | 0.00730375 |
| RAB31 | 1181.12518 | 1.01819327 | 0.00015684 | 0.00730375 |
| OLFM1 | 147.066869 | -1.6067274 | 0.00015836 | 0.00734991 |
| IL33 | 41.443055 | 1.72790304 | 0.00015951 | 0.00738361 |
| ATP1A3 | 49.6906851 | -1.8501377 | 0.0001602 | 0.00739597 |
| ZC3H12D | 67.2415498 | 1.13460863 | 0.0001616 | 0.00744135 |
| CLPTM1 | 1457.57449 | -0.4755516 | 0.00016435 | 0.00752828 |
| EP400 | 2272.55801 | -0.5860004 | 0.0001643 | 0.00752828 |
| TTK | 44.4089323 | -1.3183532 | 0.00016547 | 0.00756007 |
| CLEC4A | 39.4387689 | 1.05547806 | 0.00016598 | 0.00756378 |
| SIDT1 | 114.276164 | -1.668502 | 0.00016823 | 0.00764629 |
| C4orf19 | 255.926909 | -1.5905084 | 0.00017381 | 0.00787947 |
| SYNGR3 | 35.0597034 | -1.92422 | 0.0001759 | 0.00795387 |
| CD6 | 13.1443165 | 1.4179925 | 0.00017715 | 0.00798904 |
| NR3C1 | 1953.92573 | 0.75448223 | 0.00017758 | 0.00798904 |
| CDC6 | 84.1473931 | -1.2194748 | 0.00017942 | 0.00803485 |
| MMP15 | 417.196123 | -1.6528548 | 0.00017952 | 0.00803485 |
| MYLK2 | 3.27862054 | -1.7925107 | 0.00018029 | 0.00804883 |
| ATCAY | 3.36818337 | -1.9233803 | 0.00018099 | 0.00805993 |
| APOL1 | 530.621411 | 1.03705088 | 0.00018567 | 0.00820012 |
| PBK | 38.0584567 | -1.5696181 | 0.00018509 | 0.00820012 |

| | | | | |
|---|---|---|---|---|
| PLS1 | 123.938268 | 1.53431272 | 0.00018541 | 0.00820012 |
| SETD1A | 667.888907 | -0.6144524 | 0.00018601 | 0.00820012 |
| CCR7 | 7.85244231 | 1.51777018 | 0.00018808 | 0.00820907 |
| KBTBD12 | 10.1854184 | 1.78735729 | 0.0001876 | 0.00820907 |
| LRFN3 | 103.098441 | -0.7143417 | 0.00018675 | 0.00820907 |
| TRAP1 | 864.544188 | -0.7541584 | 0.00018786 | 0.00820907 |
| FANCA | 126.550092 | -1.1825617 | 0.00019036 | 0.00826779 |
| PPP2R3C | 157.863317 | 0.77367472 | 0.00018992 | 0.00826779 |
| EIF4A2 | 3952.07585 | 0.61597081 | 0.00019099 | 0.00827445 |
| SNORD15A | 3.9619909 | 1.56992838 | 0.00019311 | 0.00832553 |
| SYTL2 | 223.194879 | 1.34702697 | 0.0001931 | 0.00832553 |
| FOXD2-AS1 | 518.977041 | -0.7247344 | 0.00019811 | 0.00849951 |
| IFI44L | 211.654678 | 1.19171858 | 0.00019786 | 0.00849951 |
| CNTD2 | 10.4942447 | -1.646522 | 0.0002034 | 0.00864212 |
| IDUA | 342.406851 | -0.972191 | 0.00020297 | 0.00864212 |
| LOC1001315 | 562.580351 | 0.76526778 | 0.00020335 | 0.00864212 |
| NPIPB3 | 299.797242 | -1.0260914 | 0.00020197 | 0.00864212 |
| SNORA27 | 7.90069366 | 1.07227848 | 0.00020465 | 0.00867416 |
| MAP4K2 | 387.968439 | -0.4827269 | 0.00020712 | 0.00874479 |
| SPC24 | 22.4131091 | -1.2279812 | 0.00020731 | 0.00874479 |
| ATP2B4 | 9740.97413 | -0.6004066 | 0.00020945 | 0.00881383 |
| IGSF9B | 411.056669 | -1.35515 | 0.00021122 | 0.0088674 |
| EMP1 | 6568.40477 | 0.8830188 | 0.00021225 | 0.00886967 |
| STXBP5 | 520.934543 | 0.98797357 | 0.00021229 | 0.00886967 |
| ERCC6L | 17.1004845 | -1.3548097 | 0.00021332 | 0.00887329 |
| ZFP2 | 36.3249637 | 1.16591663 | 0.00021338 | 0.00887329 |
| FFAR2 | 2.45971494 | 1.8427776 | 0.000216 | 0.00896076 |
| AACS | 523.087218 | -0.7427629 | 0.00022063 | 0.00904527 |
| CLEC7A | 456.501222 | 1.34579062 | 0.00022112 | 0.00904527 |
| CREBL2 | 1076.54047 | 0.62366782 | 0.00021915 | 0.00904527 |
| DCTPP1 | 185.232775 | -0.6493446 | 0.00021997 | 0.00904527 |
| MCM2 | 281.115036 | -0.8609655 | 0.00021861 | 0.00904527 |
| VGF | 2.46037864 | -1.8986569 | 0.00022078 | 0.00904527 |
| CD164 | 3711.79589 | 0.59211108 | 0.00022212 | 0.00906516 |
| DPYSL5 | 6.23242565 | -1.8245267 | 0.00022295 | 0.00907809 |
| EPN1 | 1153.68787 | -0.5298968 | 0.00022494 | 0.00911914 |
| KIF27 | 146.108885 | 0.76753361 | 0.000225 | 0.00911914 |
| BLM | 58.3408023 | -1.3599573 | 0.00022804 | 0.00921739 |
| ZWINT | 95.1698552 | -1.2013669 | 0.00022847 | 0.00921739 |
| CEND1 | 27.1844942 | -1.7094941 | 0.00023127 | 0.00927035 |
| PIK3R1 | 3698.25152 | 1.34675958 | 0.00023136 | 0.00927035 |
| PTGES2 | 480.014016 | -0.7352544 | 0.00023041 | 0.00927035 |
| RUNX2 | 223.578535 | 1.54746456 | 0.00023212 | 0.00927934 |
| CXCL11 | 12.5161466 | 1.60222018 | 0.00023762 | 0.00945659 |
| ZNF775 | 70.3907826 | -0.5888633 | 0.00023755 | 0.00945659 |
| FDPS | 884.026021 | -0.6576732 | 0.00023952 | 0.00951052 |
| DRD4 | 13.8652944 | -1.2391792 | 0.00024215 | 0.00959322 |
| FREM2 | 3969.11009 | -1.7256748 | 0.00024513 | 0.0096894 |
| R3HDM1 | 552.299597 | -0.4591327 | 0.00024782 | 0.00977401 |
| BNIP1 | 65.032466 | 0.55289705 | 0.00025402 | 0.00994569 |

| | | | | |
|---|---|---|---|---|
| GPR64 | 486.942181 | -1.7637025 | 0.00025302 | 0.00994569 |
| IFIT3 | 479.849476 | 0.70832287 | 0.00025444 | 0.00994569 |
| NLRP3 | 239.351435 | 1.36141968 | 0.00025362 | 0.00994569 |
| TEX21P | 3.75423579 | 1.59688558 | 0.00025779 | 0.0100543 |
| TIGD5 | 105.906753 | -0.6666573 | 0.00025866 | 0.01006586 |
| PDPN | 717.616315 | -1.0281068 | 0.00026007 | 0.01009851 |
| C7orf13 | 61.4262235 | -1.4090647 | 0.00026148 | 0.01013088 |
| NCL | 4820.21214 | -0.5292549 | 0.00026692 | 0.01031906 |
| FAM180A | 87.8081712 | 1.86882252 | 0.00026908 | 0.01032376 |
| HMBS | 135.953336 | -1.0483013 | 0.00026855 | 0.01032376 |
| SCG2 | 204.338677 | 1.85597271 | 0.00026997 | 0.01032376 |
| SNORA72 | 3.39391047 | 1.73352806 | 0.00027008 | 0.01032376 |
| UPK3BL | 70.142603 | -1.2551297 | 0.00027057 | 0.01032376 |
| XG | 1.61446475 | 1.80302539 | 0.00026848 | 0.01032376 |
| SAMD10 | 64.6597453 | -1.2725594 | 0.00027148 | 0.01033627 |
| MLC1 | 10.2200631 | -1.7697808 | 0.00027256 | 0.01035476 |
| ATXN2 | 1345.63981 | -0.4851931 | 0.00027417 | 0.01036337 |
| FAM120A | 3869.92837 | -0.3595869 | 0.00027455 | 0.01036337 |
| NCAN | 32.5993958 | -1.8704163 | 0.00027422 | 0.01036337 |
| CACNG4 | 79.7878476 | -1.7877195 | 0.00027576 | 0.0103644 |
| TOMM7 | 1055.82931 | 0.6703041 | 0.00027569 | 0.0103644 |
| FPGT | 269.68476 | 0.81431677 | 0.00027922 | 0.01047212 |
| OAS2 | 242.499042 | 0.8966106 | 0.00028019 | 0.01048625 |
| CDK16 | 1086.80276 | -0.4844745 | 0.00028436 | 0.0105749 |
| DLGAP5 | 34.4550669 | -1.4623963 | 0.00028424 | 0.0105749 |
| PCNXL3 | 1311.68132 | -0.6576424 | 0.00028316 | 0.0105749 |
| SEZ6L2 | 545.105035 | -1.5567236 | 0.00028541 | 0.01059166 |
| C10orf10 | 210.097451 | 1.30267053 | 0.00028627 | 0.01060098 |
| PLEKHG2 | 1379.66757 | -0.7302272 | 0.00028723 | 0.01061441 |
| PRC1 | 433.139382 | -1.1555381 | 0.00028819 | 0.01062748 |
| SUSD2 | 94.5345744 | -1.6529285 | 0.0002899 | 0.01066818 |
| NOB1 | 468.982517 | -0.6807417 | 0.00029322 | 0.01076788 |
| OPA3 | 793.87245 | -0.4882549 | 0.0002948 | 0.01080327 |
| CD300C | 37.8247051 | 1.21344042 | 0.00029723 | 0.01083804 |
| ESCO2 | 30.8887832 | -1.2417175 | 0.00029819 | 0.01083804 |
| HIST1H2AM | 138.544927 | -1.0416952 | 0.00029823 | 0.01083804 |
| SNORA20 | 6.87675837 | 1.62999398 | 0.00029883 | 0.01083804 |
| SPATA9 | 3.30575699 | 1.8083566 | 0.00029795 | 0.01083804 |
| CD300LB | 6.64300026 | 1.44520454 | 0.00030362 | 0.01096637 |
| GFRA1 | 1109.79819 | 1.85520251 | 0.00030309 | 0.01096637 |
| ALCAM | 9746.0414 | 0.83791174 | 0.00030784 | 0.01109602 |
| GPX3 | 670.721187 | 1.48846131 | 0.00031094 | 0.01118481 |
| FOXL2NB | 6.68484407 | -1.758502 | 0.00031404 | 0.01127322 |
| MIR4697HG | 269.543671 | -1.3398562 | 0.00031598 | 0.01131999 |
| CORIN | 18.8552646 | 1.72762265 | 0.00031667 | 0.01132147 |
| ISG15 | 156.205295 | 1.01319209 | 0.0003178 | 0.0113391 |
| CDKN3 | 27.1741125 | -1.3621936 | 0.00032042 | 0.01140916 |
| ARHGDIA | 2933.35323 | -0.5722576 | 0.00032579 | 0.01141563 |
| CCNA2 | 81.7149116 | -1.2070347 | 0.00032366 | 0.01141563 |
| CCNF | 80.9304504 | -1.0843309 | 0.00032553 | 0.01141563 |

| | | | | |
|---|---|---|---|---|
| COX7B | 858.640947 | -0.7994163 | 0.00032409 | 0.01141563 |
| FADS2 | 1375.19473 | -1.2722738 | 0.00032198 | 0.01141563 |
| IFIT2 | 313.023953 | 0.80659595 | 0.00032416 | 0.01141563 |
| PHYHIP | 28.9783989 | -1.6837327 | 0.00032315 | 0.01141563 |
| TRIM9 | 52.1587527 | 1.59580221 | 0.00032504 | 0.01141563 |
| TNNI2 | 4.92155035 | 1.71627748 | 0.00033352 | 0.01166341 |
| C4orf48 | 54.8334369 | -1.1827969 | 0.00033701 | 0.01176185 |
| CDH8 | 97.7039636 | -1.7318958 | 0.00034003 | 0.01177398 |
| CNOT3 | 478.942488 | -0.568887 | 0.00033994 | 0.01177398 |
| HIST2H3D | 44.4354818 | -1.2053578 | 0.00033965 | 0.01177398 |
| TMEM151A | 3.45934977 | -1.8421416 | 0.00033817 | 0.01177398 |
| CHEK1 | 90.6303311 | -1.0679252 | 0.00034112 | 0.01178831 |
| ADPRM | 62.5806161 | 0.83175944 | 0.0003433 | 0.01179434 |
| CD247 | 9.89399053 | 1.48837034 | 0.00034281 | 0.01179434 |
| NUP214 | 1505.39717 | -0.3611044 | 0.00034207 | 0.01179434 |
| ITGA8 | 242.601177 | 1.73125049 | 0.00034472 | 0.01182007 |
| AMICA1 | 80.9566025 | 1.33464349 | 0.00034613 | 0.01184524 |
| TXK | 39.2854192 | 1.41999763 | 0.00034852 | 0.0119039 |
| FAM114A2 | 359.724447 | 0.46602164 | 0.00034972 | 0.01192152 |
| ARRDC3 | 2570.05246 | 1.25583187 | 0.000357 | 0.01212286 |
| RGS9BP | 5.83173819 | -1.6975078 | 0.0003566 | 0.01212286 |
| ZAK | 926.562975 | 0.72474548 | 0.00036509 | 0.01237357 |
| SIGMAR1 | 597.623214 | -0.5298744 | 0.00036999 | 0.01251546 |
| ADCY10P1 | 70.2877855 | 1.3692347 | 0.00037218 | 0.01255785 |
| TCF7L1 | 574.217216 | -0.813005 | 0.00037267 | 0.01255785 |
| TLR2 | 720.006014 | 1.14347851 | 0.00037563 | 0.01261745 |
| TRAF4 | 595.275102 | -0.7916523 | 0.00037587 | 0.01261745 |
| DGKD | 885.32875 | -0.8337344 | 0.00037714 | 0.01262068 |
| INMT-FAM18 | 5.44777256 | 1.81647289 | 0.0003776 | 0.01262068 |
| RBPMS2 | 262.679732 | -1.216272 | 0.00037812 | 0.01262068 |
| ATP6V0E2 | 524.280896 | -0.8610488 | 0.00037889 | 0.0126225 |
| AAED1 | 91.9558205 | 0.59526257 | 0.00038019 | 0.01264202 |
| BRD3 | 1082.62033 | -0.5816047 | 0.00038156 | 0.01266332 |
| CCDC86 | 204.514476 | -0.7366473 | 0.00038537 | 0.01276581 |
| LCA5 | 94.2640025 | 1.00640963 | 0.00038896 | 0.01286047 |
| TEC | 111.143581 | 1.13333713 | 0.00039364 | 0.012991 |
| LHCGR | 114.897837 | 1.75473045 | 0.00039468 | 0.01300063 |
| DTWD1 | 272.182944 | 0.51778623 | 0.00039586 | 0.01300295 |
| HHIP-AS1 | 43.8063011 | -1.6929007 | 0.00039696 | 0.01300295 |
| TICRR | 57.8854213 | -1.5856762 | 0.00039695 | 0.01300295 |
| PHF12 | 826.08144 | -0.4265477 | 0.00039787 | 0.0130083 |
| ITPKA | 9.58417332 | -1.5902849 | 0.00040071 | 0.01305273 |
| TERF2 | 416.465787 | -0.5494538 | 0.00040045 | 0.01305273 |
| ARHGAP39 | 173.782108 | -0.852134 | 0.00040701 | 0.0131289 |
| ECHDC3 | 12.1640194 | 1.56160482 | 0.00040902 | 0.0131289 |
| HACE1 | 258.6982 | 0.74000157 | 0.00040867 | 0.0131289 |
| IL6ST | 10372.169 | 0.61868154 | 0.00040697 | 0.0131289 |
| LY6H | 18.2078942 | -1.791041 | 0.00040382 | 0.0131289 |
| NPLOC4 | 1528.63467 | -0.5003301 | 0.00040756 | 0.0131289 |
| RNFT2 | 144.453353 | -1.5315322 | 0.00040791 | 0.0131289 |

| | | | | |
|---|---|---|---|---|
| RWDD3 | 52.6028447 | 0.77143383 | 0.00040813 | 0.0131289 |
| ANGPTL5 | 28.6184835 | 1.8009638 | 0.00041343 | 0.01323144 |
| BCORL1 | 381.167261 | -0.8628377 | 0.00041748 | 0.01323144 |
| GABRQ | 13.0521452 | -1.772165 | 0.00041721 | 0.01323144 |
| ITGB4 | 9527.50398 | -1.2847384 | 0.00041473 | 0.01323144 |
| PCGF5 | 1843.75785 | 0.8967184 | 0.00041731 | 0.01323144 |
| PSMG2 | 371.067929 | 0.60937018 | 0.00041424 | 0.01323144 |
| STK17B | 464.570012 | 1.08043741 | 0.00041594 | 0.01323144 |
| CHRDL2 | 3.31518216 | -1.7894875 | 0.00042102 | 0.01329657 |
| HUNK | 50.3183101 | -1.344042 | 0.00042105 | 0.01329657 |
| FAM69B | 276.998471 | -0.7091701 | 0.00042375 | 0.013358 |
| CCNE2 | 43.4123556 | -1.1979793 | 0.00042669 | 0.01342647 |
| CMPK1 | 1112.98894 | 0.59595519 | 0.00042804 | 0.0134272 |
| ZBP1 | 5.43126313 | 1.46383314 | 0.00042824 | 0.0134272 |
| SCAF4 | 756.605242 | -0.6030953 | 0.00043247 | 0.01353589 |
| LINC00607 | 142.49431 | 1.37875928 | 0.00043417 | 0.01356492 |
| VMO1 | 30.6308049 | 1.30995123 | 0.00043776 | 0.01365282 |
| TM7SF2 | 298.763556 | -1.0692821 | 0.00044035 | 0.0137094 |
| IGSF9 | 70.1731762 | -1.3728054 | 0.00044165 | 0.01372552 |
| PLAC9 | 122.913253 | 1.70201292 | 0.00044683 | 0.01386182 |
| AKAP1 | 1036.36415 | -0.8121052 | 0.00044973 | 0.01392751 |
| KCNH1 | 19.561271 | -1.6404016 | 0.00045665 | 0.01411684 |
| ABTB2 | 209.081426 | -1.2245323 | 0.00045872 | 0.01415587 |
| ELP2 | 921.045707 | 0.6098781 | 0.00045981 | 0.01416462 |
| KIAA0195 | 1160.87258 | -0.4583408 | 0.000462 | 0.01420718 |
| MLST8 | 366.789292 | -0.6219156 | 0.00046867 | 0.01438714 |
| SNORA57 | 187.926466 | 1.03061276 | 0.0004716 | 0.01445193 |
| FAM198A | 1224.83032 | 1.34775223 | 0.00047369 | 0.01446571 |
| TBC1D13 | 610.926846 | -0.3002822 | 0.00047345 | 0.01446571 |
| RNF44 | 734.490657 | -0.4960643 | 0.00047465 | 0.01446996 |
| LOC10192739 | 7.63735121 | 1.70532904 | 0.00048242 | 0.01468123 |
| PRX | 364.677496 | 1.26656489 | 0.00048643 | 0.01477781 |
| ACACB | 1092.73551 | -0.6269323 | 0.00049608 | 0.0150449 |
| LOC1005066 | 95.2437536 | -1.6844097 | 0.00049755 | 0.01506341 |
| IFT43 | 73.2569459 | 0.7491709 | 0.00050207 | 0.01512777 |
| KCNJ3 | 10.8182997 | 1.78493648 | 0.00050225 | 0.01512777 |
| ZNF423 | 1714.83136 | -0.7268706 | 0.0005013 | 0.01512777 |
| COMMD8 | 100.213998 | 0.67583097 | 0.00051027 | 0.01531695 |
| SMC4 | 1105.37663 | -0.9005417 | 0.00050982 | 0.01531695 |
| SAMHD1 | 928.120179 | 0.84742437 | 0.00051459 | 0.01542015 |
| GRIK2 | 16.4238168 | 1.48633808 | 0.000517 | 0.01544698 |
| IL12RB1 | 27.9559498 | 1.10793307 | 0.00051724 | 0.01544698 |
| ABCG4 | 25.4852897 | -1.4665661 | 0.00052017 | 0.01545571 |
| ANKRD52 | 1712.45412 | -0.7238782 | 0.00052016 | 0.01545571 |
| THAP8 | 44.1752848 | -0.8416921 | 0.00051844 | 0.01545571 |
| C5orf66 | 26.9533777 | -1.7017877 | 0.00052156 | 0.01547101 |
| LOC1005061 | 8.12480053 | 1.42405342 | 0.00052269 | 0.01547828 |
| RHOBTB3 | 753.136103 | 1.60545822 | 0.00052387 | 0.01548727 |
| DVL3 | 1417.25627 | -0.4471127 | 0.00053163 | 0.01569011 |
| BRINP2 | 1.7090732 | -1.7663031 | 0.00053391 | 0.01573105 |

| | | | | |
|---|---|---|---|---|
| LINC00899 | 19.5447284 | 1.01312958 | 0.00053713 | 0.01579959 |
| CALCOCO1 | 3493.32472 | 0.66365335 | 0.00053915 | 0.01583249 |
| SNORA76C | 7.58209681 | 1.59784007 | 0.00054166 | 0.01587962 |
| DEDD | 373.565474 | -0.3165357 | 0.00054391 | 0.01589325 |
| SASH1 | 707.905901 | 1.19709653 | 0.00054393 | 0.01589325 |
| SLC7A14 | 490.207095 | -1.7643402 | 0.0005461 | 0.01593026 |
| RAB3A | 46.6074591 | -1.0299987 | 0.00054865 | 0.01595296 |
| ZNF570 | 126.223985 | 0.61630701 | 0.00054869 | 0.01595296 |
| GOT1 | 554.609153 | -1.0871807 | 0.00055056 | 0.01598073 |
| CLEC10A | 27.0928777 | 1.70442122 | 0.0005544 | 0.01602736 |
| LPIN2 | 922.627809 | 0.828656 | 0.0005547 | 0.01602736 |
| SETD5 | 2774.98225 | -0.4775621 | 0.0005549 | 0.01602736 |
| MARK1 | 648.494192 | -1.1805344 | 0.00055602 | 0.01603333 |
| SQLE | 484.471623 | -1.1762146 | 0.00055761 | 0.01605297 |
| GET4 | 289.196534 | -0.4324585 | 0.00056245 | 0.01611326 |
| RTN3 | 2147.87717 | -0.4936435 | 0.00056154 | 0.01611326 |
| SRCIN1 | 45.8184274 | -1.4312402 | 0.00056237 | 0.01611326 |
| PRNCR1 | 61.289505 | -1.2688342 | 0.00056435 | 0.01611499 |
| TRIM47 | 187.888626 | -1.099577 | 0.00056389 | 0.01611499 |
| GPR21 | 64.6712267 | -0.7859985 | 0.00056898 | 0.01621487 |
| POLR1B | 619.043974 | -0.6476012 | 0.00056969 | 0.01621487 |
| ZNF780A | 521.780475 | 0.51870871 | 0.00057129 | 0.0162342 |
| ATP8B3 | 133.483461 | -1.3541126 | 0.00057329 | 0.01626488 |
| STAT4 | 12.6385604 | 1.26653699 | 0.00057593 | 0.01631338 |
| GPKOW | 240.546861 | 0.35121016 | 0.00057715 | 0.01632177 |
| TAF4 | 353.93982 | -0.8277686 | 0.00058251 | 0.0164467 |
| ABCA3 | 1991.39951 | -0.8190156 | 0.00058874 | 0.01651969 |
| CREBRF | 1415.21206 | 0.46400175 | 0.00058719 | 0.01651969 |
| F5 | 35.4527683 | -1.7391984 | 0.00058885 | 0.01651969 |
| GRIN1 | 8.33564623 | -1.7659356 | 0.00058697 | 0.01651969 |
| CNTN4 | 511.387956 | 1.67881399 | 0.00059323 | 0.01661127 |
| FBXO22-AS1 | 1.81562895 | -1.7602836 | 0.000594 | 0.01661127 |
| DAPK2 | 76.8680076 | 1.38536329 | 0.00059573 | 0.01663317 |
| ZBTB24 | 389.874381 | 0.6839447 | 0.0005979 | 0.01666741 |
| OAS1 | 262.874353 | 0.66661199 | 0.00059973 | 0.01669198 |
| ZNF629 | 928.118515 | -0.4169228 | 0.0006024 | 0.01673977 |
| ARRDC4 | 553.879342 | -1.0893213 | 0.00060428 | 0.01676533 |
| EPM2A | 102.270071 | 0.68002568 | 0.00061199 | 0.01689943 |
| GSTM1 | 15.6464808 | -1.7536691 | 0.00061101 | 0.01689943 |
| GTF2H5 | 294.876947 | 0.63082083 | 0.00061144 | 0.01689943 |
| MRPS5 | 489.172677 | -0.5808466 | 0.00061429 | 0.01693638 |
| C5orf56 | 39.0002888 | 0.87511901 | 0.0006188 | 0.01698886 |
| CCDC23 | 92.6751453 | 0.77944759 | 0.00061926 | 0.01698886 |
| DNAJC4 | 320.021597 | -0.6467883 | 0.00062006 | 0.01698886 |
| SLC24A1 | 475.659578 | -0.6798136 | 0.00061863 | 0.01698886 |
| SOX8 | 18.9243392 | -1.2910443 | 0.00062433 | 0.01707922 |
| LLGL2 | 346.843196 | -1.2897627 | 0.00062878 | 0.01717426 |
| LOC1030918 | 37.60704 | -0.977154 | 0.00063022 | 0.01718706 |
| MASP1 | 40.469208 | 1.69657645 | 0.00063156 | 0.01719683 |
| DEPDC1 | 40.6776099 | -1.2355744 | 0.00063524 | 0.01727025 |

| | | | | |
|---|---|---|---|---|
| CCDC144A | 292.273202 | -1.6061191 | 0.00063845 | 0.0173041 |
| SEC14L5 | 8.71510166 | -1.4847561 | 0.00063813 | 0.0173041 |
| XAF1 | 558.454296 | 0.82155873 | 0.00064411 | 0.01743056 |
| BOC | 4426.76207 | 1.32711879 | 0.00064767 | 0.01749995 |
| PRSS23 | 566.519414 | 1.23322343 | 0.00065538 | 0.01768131 |
| C16orf54 | 19.2289416 | 1.15332857 | 0.00065651 | 0.01768463 |
| CERS2 | 4042.99363 | -0.5583752 | 0.00066655 | 0.01781601 |
| CMTM2 | 3.34249336 | 1.62163149 | 0.00066243 | 0.01781601 |
| FAM19A2 | 26.3905342 | 0.97119367 | 0.00066684 | 0.01781601 |
| LAMC2 | 87.7307388 | -1.4525177 | 0.00066717 | 0.01781601 |
| MRPS2 | 283.818669 | -0.564537 | 0.00066747 | 0.01781601 |
| TATDN2 | 1156.10332 | -0.5355433 | 0.00066728 | 0.01781601 |
| ADORA1 | 11.9665477 | -1.4007846 | 0.0006704 | 0.01784209 |
| EPN3 | 32.7775118 | -1.6699652 | 0.00067047 | 0.01784209 |
| ANKRA2 | 253.376954 | 0.52825273 | 0.00068259 | 0.01800856 |
| GJA3 | 90.4196127 | -1.6870954 | 0.00068048 | 0.01800856 |
| PGP | 305.082739 | -0.5748915 | 0.00068354 | 0.01800856 |
| POLN | 58.2848447 | 0.8346727 | 0.00068278 | 0.01800856 |
| SYN1 | 33.4942971 | -1.0122078 | 0.0006839 | 0.01800856 |
| TERC | 698.249161 | -1.0093162 | 0.00068284 | 0.01800856 |
| ZNF768 | 386.135216 | -0.4474849 | 0.0006792 | 0.01800856 |
| BCL2 | 618.417534 | 1.24103621 | 0.00068626 | 0.0180437 |
| PCGF2 | 513.511009 | -0.6284921 | 0.00069247 | 0.0181798 |
| PCAT1 | 11.933166 | -1.41005 | 0.00069357 | 0.01818154 |
| SLC26A4 | 11.8801551 | 1.36429454 | 0.00069644 | 0.01820254 |
| VAMP5 | 123.79583 | 1.00210968 | 0.00069625 | 0.01820254 |
| LOC151475 | 8.73259242 | 1.6437356 | 0.00070035 | 0.01827756 |
| MEF2D | 1658.85447 | -0.4544054 | 0.00070725 | 0.01843042 |
| KLHL6 | 134.140627 | 1.09338099 | 0.00070936 | 0.0184307 |
| MGAM | 95.8588144 | 1.30969194 | 0.00070877 | 0.0184307 |
| AICDA | 1.3935683 | 1.73361337 | 0.00071465 | 0.01848743 |
| EFCAB12 | 4.80134365 | -1.4590905 | 0.00071549 | 0.01848743 |
| NLRP12 | 10.7581509 | 1.43509916 | 0.00071574 | 0.01848743 |
| SAYSD1 | 124.412289 | 0.72862066 | 0.00071544 | 0.01848743 |
| GPM6A | 32.4870844 | -1.6804744 | 0.0007173 | 0.0185004 |
| LARP1 | 4971.46735 | -0.5289115 | 0.00072122 | 0.01857432 |
| LOXL3 | 179.946148 | 1.30722965 | 0.00072321 | 0.01859831 |
| PLCD3 | 2264.05525 | -0.6563351 | 0.00072446 | 0.01860338 |
| DPH6-AS1 | 16.3454428 | 1.1334482 | 0.00072946 | 0.01867707 |
| ZC3H3 | 276.573113 | -0.5067905 | 0.00072871 | 0.01867707 |
| SRCAP | 2828.66378 | -0.4675429 | 0.00073103 | 0.0186902 |
| HMGA2 | 60.5557373 | -1.6953002 | 0.00073226 | 0.01869441 |
| FUS | 2232.18599 | -0.8533825 | 0.0007341 | 0.0187142 |
| TMX1 | 464.433402 | 0.50123569 | 0.00073534 | 0.01871876 |
| AUNIP | 5.44180801 | -1.442769 | 0.0007396 | 0.01875852 |
| CLASRP | 518.714182 | -0.4973292 | 0.00073863 | 0.01875852 |
| RNF208 | 22.8658994 | -0.9755788 | 0.0007401 | 0.01875852 |
| SSPN | 445.22142 | 1.46001697 | 0.00074546 | 0.01886723 |
| CDK1 | 115.213209 | -1.2225416 | 0.0007496 | 0.01887725 |
| ELMOD3 | 185.911549 | 0.56892542 | 0.00074748 | 0.01887725 |

| | | | | |
|---|---|---|---|---|
| OASL | 35.9697193 | 1.3953151 | 0.00074904 | 0.01887725 |
| TMEM8A | 573.487519 | -0.6592683 | 0.00075015 | 0.01887725 |
| ITGB5 | 1042.54733 | 1.01550564 | 0.00075536 | 0.01898108 |
| PDZK1IP1 | 6.41398065 | 1.7282132 | 0.00075696 | 0.01899426 |
| RASD1 | 58.9430648 | 1.63642629 | 0.0007587 | 0.01901075 |
| ATG4C | 152.249376 | 0.73982908 | 0.00076542 | 0.01915184 |
| CC2D2B | 7.66815468 | 1.24068274 | 0.00076687 | 0.01916083 |
| PKDREJ | 4.73309015 | 1.62941349 | 0.00077474 | 0.01933007 |
| TPX2 | 316.109967 | -1.2546908 | 0.00077781 | 0.01937917 |
| POLR1A | 1231.63425 | -0.474804 | 0.00078226 | 0.01942011 |
| SH3BP4 | 718.070325 | 1.1118275 | 0.00078277 | 0.01942011 |
| STRADA | 528.576915 | -0.4622899 | 0.00078207 | 0.01942011 |
| WHSC1 | 1346.05782 | -0.6679012 | 0.00078625 | 0.01947912 |
| NAA35 | 366.439475 | -0.3517087 | 0.000788 | 0.01949496 |
| HNRNPUL1 | 2919.68325 | -0.3005057 | 0.0007969 | 0.01960473 |
| LIG1 | 420.835765 | -0.7369048 | 0.0007963 | 0.01960473 |
| LRRK2 | 873.992373 | 0.8179721 | 0.00079552 | 0.01960473 |
| RABGGTB | 351.594057 | 0.56523747 | 0.00079622 | 0.01960473 |
| EME1 | 33.0575484 | -1.2786408 | 0.00080251 | 0.0197153 |
| ANGPTL7 | 101.955482 | 1.38795895 | 0.00080498 | 0.01974842 |
| RNF26 | 428.885173 | -0.6439039 | 0.00081307 | 0.01991915 |
| KIF18A | 28.1279117 | -1.2439904 | 0.00081933 | 0.02004452 |
| NOD2 | 39.8504384 | 1.28018057 | 0.00082084 | 0.02005354 |
| TMEM246 | 1276.54676 | -0.7173837 | 0.00082443 | 0.02011341 |
| ATP5I | 656.012373 | -0.5761399 | 0.0008261 | 0.02012608 |
| ZNF444 | 168.065277 | -0.5284299 | 0.00083489 | 0.02031222 |
| RNF125 | 157.74378 | 0.93996954 | 0.00083675 | 0.02032939 |
| MDH2 | 1390.38919 | -0.6392659 | 0.00083977 | 0.02037445 |
| TMEM134 | 140.205126 | -0.5171753 | 0.00084397 | 0.02044833 |
| SKA3 | 39.3125432 | -1.2364612 | 0.00086012 | 0.02081098 |
| MAZ | 1133.81199 | -0.5185187 | 0.00086683 | 0.02094449 |
| SNHG17 | 111.045606 | -0.834907 | 0.0008766 | 0.02115131 |
| POLR2D | 395.885823 | -0.3531347 | 0.00088003 | 0.02120499 |
| KPNA2 | 504.216966 | -0.8988128 | 0.00088308 | 0.02124945 |
| MCOLN2 | 20.1765127 | 1.38611838 | 0.00089231 | 0.02141312 |
| OR56B1 | 11.030116 | 1.33654706 | 0.00089185 | 0.02141312 |
| CNTN2 | 34.9796086 | -1.643711 | 0.00089432 | 0.02143202 |
| SCARNA2 | 3048.52922 | 0.83464158 | 0.00090115 | 0.02156634 |
| CSNK1D | 2033.1721 | -0.6409651 | 0.00090403 | 0.02159742 |
| RIMS1 | 30.3491435 | -1.6532323 | 0.00090491 | 0.02159742 |
| CALML3-AS1 | 39.5295346 | -1.5875127 | 0.0009078 | 0.02160775 |
| ELN | 2210.35411 | 1.54911259 | 0.00090662 | 0.02160775 |
| ABCB6 | 470.169809 | -0.5830816 | 0.00091039 | 0.02164012 |
| APOL6 | 810.490677 | 0.64891747 | 0.00092073 | 0.0217467 |
| CDH7 | 26.4467898 | -1.6987976 | 0.0009201 | 0.0217467 |
| GOT2 | 1028.52592 | -0.5777315 | 0.00092211 | 0.0217467 |
| IL18RAP | 10.3666287 | 1.60531864 | 0.00091987 | 0.0217467 |
| MCM4 | 765.122384 | -0.8864387 | 0.0009224 | 0.0217467 |
| PMVK | 238.6111 | -0.6620978 | 0.00091787 | 0.0217467 |
| SPAG8 | 37.6194156 | 0.94165172 | 0.00092352 | 0.0217467 |

| | | | | |
|---|---|---|---|---|
| MALT1 | 526.940268 | 0.64251047 | 0.0009269 | 0.02179703 |
| COLGALT2 | 24.3323051 | -1.5157432 | 0.00092987 | 0.02183768 |
| OIP5 | 11.3246478 | -1.3091058 | 0.00093487 | 0.0219257 |
| WNT9A | 118.75772 | -0.8552457 | 0.00094944 | 0.0222378 |
| CSPG5 | 19.9155185 | -1.4021514 | 0.0009544 | 0.02229469 |
| STAG1 | 1119.99183 | 0.31344564 | 0.000954 | 0.02229469 |
| B9D2 | 25.3105556 | 0.95196137 | 0.00096284 | 0.02244409 |
| COMTD1 | 36.9305474 | -0.9662192 | 0.00096335 | 0.02244409 |
| AMN1 | 78.929731 | 0.53319841 | 0.00096603 | 0.02247692 |
| SLC7A1 | 778.922323 | -1.5005092 | 0.00096884 | 0.02251246 |
| POLR3K | 90.0604691 | -0.707779 | 0.00098281 | 0.02279772 |
| SERPINB9 | 585.821468 | 0.99988304 | 0.00098371 | 0.02279772 |
| SLC25A39 | 937.682927 | -0.4686624 | 0.00098718 | 0.02284813 |
| SF3B5 | 367.622169 | 0.52705448 | 0.00099303 | 0.02295318 |
| RICTOR | 1223.15271 | 0.46184361 | 0.00099772 | 0.02303131 |
| MGAT4A | 1055.48437 | 0.84904213 | 0.00100004 | 0.0230547 |
| ARL16 | 259.412152 | -0.5035543 | 0.00100707 | 0.02315593 |
| MORC3 | 1008.96353 | 0.44155911 | 0.00100645 | 0.02315593 |
| BZW2 | 213.738735 | -0.7693154 | 0.00101706 | 0.02335518 |
| SOX18 | 68.2754286 | -1.2027017 | 0.00102098 | 0.02341473 |
| CD300E | 15.9858621 | 1.5191015 | 0.0010256 | 0.0234594 |
| GPC3 | 204.304664 | -1.6617582 | 0.00102462 | 0.0234594 |
| NTN4 | 413.956708 | 1.31985067 | 0.00103302 | 0.02356786 |
| SERINC1 | 4765.64937 | 0.59389746 | 0.00103213 | 0.02356786 |
| PLEC | 21119.1972 | -0.6504132 | 0.00103536 | 0.02359056 |
| CKS1B | 94.9805578 | -0.8094054 | 0.00104001 | 0.02362981 |
| PCDH10 | 11.2207642 | -1.6597985 | 0.0010409 | 0.02362981 |
| ZNF383 | 114.141583 | 0.47017274 | 0.00104111 | 0.02362981 |
| ADAM28 | 286.798688 | 1.14182644 | 0.00104478 | 0.02368259 |
| GZMA | 11.9375567 | 1.45579651 | 0.00104779 | 0.02368728 |
| RELN | 854.65471 | -1.6845648 | 0.00104903 | 0.02368728 |
| TMEM127 | 1269.82833 | -0.3782614 | 0.00104789 | 0.02368728 |
| FEN1 | 237.019663 | -0.6508164 | 0.00105543 | 0.02380126 |
| DNMT3A | 773.242333 | -0.6589495 | 0.0010579 | 0.02382653 |
| RPS6KA5 | 65.5958639 | 0.82672101 | 0.001061 | 0.02386578 |
| ANP32B | 1141.99694 | -0.8931546 | 0.00106415 | 0.02387554 |
| KIFAP3 | 766.375719 | 0.49595471 | 0.00106368 | 0.02387554 |
| PCDHB5 | 604.36811 | -1.2066907 | 0.00106711 | 0.02388091 |
| PSMC5 | 880.370864 | -0.4811021 | 0.00106692 | 0.02388091 |
| LOC1005056 | 8.57202714 | -1.190293 | 0.00107338 | 0.02393009 |
| MGRN1 | 1067.30745 | -0.4359741 | 0.00107262 | 0.02393009 |
| TMEM19 | 543.411898 | 0.5970375 | 0.00107317 | 0.02393009 |
| MYO1E | 629.230247 | 0.9714611 | 0.00107479 | 0.02393112 |
| IL11RA | 363.324887 | 1.16684634 | 0.00107985 | 0.02401338 |
| GNG12 | 1024.73374 | 0.82755839 | 0.00108231 | 0.02403077 |
| SNORA8 | 42.5785516 | 0.82955108 | 0.00108337 | 0.02403077 |
| HDAC4 | 2299.03017 | -0.7444871 | 0.00108788 | 0.02410058 |
| JPH3 | 39.4660634 | -1.6725893 | 0.00109539 | 0.02423632 |
| MT3 | 68.5162386 | -1.5444395 | 0.00109882 | 0.02426394 |
| RBFADN | 6.49763942 | 1.32857728 | 0.0010994 | 0.02426394 |

| | | | | |
|---|---|---|---|---|
| LINC00158 | 8.45878298 | 1.49559334 | 0.00110186 | 0.02428785 |
| IL20RA | 36.5973998 | 1.53347918 | 0.00110496 | 0.02432562 |
| ITGAL | 128.382463 | 1.1134683 | 0.00111017 | 0.02437106 |
| SEPT5 | 9.53015983 | -1.2365542 | 0.00111019 | 0.02437106 |
| TMEM219 | 628.20608 | 0.3647446 | 0.00111118 | 0.02437106 |
| FAM95A | 30.7282062 | -1.6017842 | 0.0011227 | 0.02459314 |
| DAO | 2.11890062 | 1.58648331 | 0.00112974 | 0.02471653 |
| CLEC9A | 55.3265936 | 1.6121298 | 0.00113214 | 0.02473821 |
| LOC1019296 | 11.1727499 | 1.63204752 | 0.00114082 | 0.02489701 |
| DCC | 219.514783 | 1.64737564 | 0.00114474 | 0.02494476 |
| ZC2HC1C | 125.300603 | 1.34904484 | 0.00114584 | 0.02494476 |
| SNX9 | 979.100046 | 0.54578172 | 0.0011476 | 0.02495212 |
| CENPN | 138.655407 | -1.0443479 | 0.00115206 | 0.02498528 |
| SLC14A2 | 8.23485044 | 1.50963729 | 0.00115339 | 0.02498528 |
| TAF15 | 808.588318 | -0.567172 | 0.00115298 | 0.02498528 |
| HIST1H3G | 99.1812688 | -1.308637 | 0.0011605 | 0.0251085 |
| CS | 2179.76067 | -0.529897 | 0.00116881 | 0.02525712 |
| THAP4 | 472.218264 | -0.4770179 | 0.00117358 | 0.02532923 |
| GLIPR1 | 645.402751 | 0.64347593 | 0.00118297 | 0.02550061 |
| RABL6 | 1034.16655 | -0.4263493 | 0.0011872 | 0.02556046 |
| ELL2 | 806.48783 | 1.09202164 | 0.00119 | 0.02556546 |
| NUP210 | 786.785757 | -0.9178991 | 0.00119034 | 0.02556546 |
| CERS5 | 640.738754 | -0.5324956 | 0.00120422 | 0.02582324 |
| ZMYND15 | 79.2494955 | 1.03211769 | 0.00120528 | 0.02582324 |
| LOC80154 | 16.8812641 | -1.0620171 | 0.00121347 | 0.0259671 |
| AURKA | 66.9420466 | -1.2333079 | 0.00122181 | 0.02611295 |
| LOC399715 | 51.5993357 | 1.11530182 | 0.00122565 | 0.02611295 |
| NDUFA10 | 842.745808 | -0.4138668 | 0.00122623 | 0.02611295 |
| SLC5A12 | 4.74215997 | 1.57816814 | 0.00122509 | 0.02611295 |
| CDH26 | 14.0610137 | 1.18447934 | 0.00122952 | 0.02611996 |
| TNFAIP3 | 1147.4414 | 1.03952777 | 0.00122827 | 0.02611996 |
| BBS9 | 646.563368 | 0.97924307 | 0.00123246 | 0.0261508 |
| SF3B4 | 487.560974 | -0.552363 | 0.00123553 | 0.02618434 |
| GANAB | 6139.28984 | -0.4498447 | 0.00124027 | 0.02622154 |
| JADE3 | 290.582377 | -0.6314249 | 0.00123987 | 0.02622154 |
| SSRP1 | 1356.39972 | -0.434937 | 0.00124329 | 0.0262538 |
| FBXO8 | 266.620987 | 0.3902239 | 0.0012531 | 0.02642925 |
| CSNK2A2 | 390.969314 | -0.4377229 | 0.00125924 | 0.02652705 |
| C1orf123 | 252.291551 | 0.59320941 | 0.00126362 | 0.02656196 |
| SLC24A5 | 10.6412288 | 1.61249705 | 0.00126392 | 0.02656196 |
| TMPO-AS1 | 44.4499 | -0.6820172 | 0.00126807 | 0.02661727 |
| ATP5B | 5987.12229 | -0.6757806 | 0.00127117 | 0.02665053 |
| TMEM11 | 152.66174 | -0.4659695 | 0.00127272 | 0.02665134 |
| NME9 | 3.64187204 | 1.64136848 | 0.00128059 | 0.02678436 |
| CYS1 | 165.531856 | 1.57451786 | 0.00128213 | 0.02678454 |
| HLA-DRB5 | 506.403834 | 1.61285999 | 0.00128624 | 0.02683869 |
| NDUFS8 | 775.46535 | -0.614962 | 0.00128971 | 0.02687904 |
| COL20A1 | 3.8768435 | -1.6535922 | 0.00129566 | 0.02693928 |
| GATAD2B | 1304.30886 | -0.3835576 | 0.00129457 | 0.02693928 |
| CTGF | 11035.6066 | 1.40065875 | 0.00129911 | 0.02697909 |

| | | | | |
|---|---|---|---|---|
| TCF3 | 713.086961 | -0.5311534 | 0.00130294 | 0.02702676 |
| LINC00663 | 84.3316825 | 0.54209834 | 0.00130886 | 0.02705387 |
| PLEKHB1 | 66.9747366 | -1.4176162 | 0.00130592 | 0.02705387 |
| POM121 | 965.083411 | -0.4953533 | 0.00130806 | 0.02705387 |
| GS1-259H13 | 17.3960998 | 1.11776046 | 0.0013139 | 0.0270627 |
| RGL1 | 1270.66502 | 0.94118081 | 0.00131238 | 0.0270627 |
| SNORA74A | 182.308408 | -1.0126991 | 0.00131179 | 0.0270627 |
| COLCA2 | 62.3665539 | -1.3469181 | 0.00131589 | 0.02707204 |
| DNM1 | 75.5388127 | -1.1611811 | 0.00132111 | 0.02713064 |
| RSPO2 | 58.7120293 | -1.6458034 | 0.00132183 | 0.02713064 |
| UBALD1 | 175.546366 | -0.7903937 | 0.00132964 | 0.02725907 |
| LOC440300 | 106.631912 | 0.94786482 | 0.00133702 | 0.02729914 |
| PTTG2 | 3.70810624 | 1.44606592 | 0.0013378 | 0.02729914 |
| RAB7A | 2623.53422 | -0.4418496 | 0.00133767 | 0.02729914 |
| RNF148 | 26.4814227 | -1.23633 | 0.00133576 | 0.02729914 |
| MKRN7P | 17.4713453 | 1.22635634 | 0.00134467 | 0.02739068 |
| MTERFD2 | 478.818441 | -0.4167512 | 0.0013454 | 0.02739068 |
| ABCC8 | 2.87409957 | -1.6221682 | 0.00135568 | 0.02744162 |
| PAICS | 1231.84201 | -0.8085988 | 0.001353 | 0.02744162 |
| RAPGEF6 | 1124.94941 | 0.4843541 | 0.00135564 | 0.02744162 |
| SNORD89 | 3.10219059 | 1.57898042 | 0.00135203 | 0.02744162 |
| TPCN2 | 344.960531 | -0.5198442 | 0.0013557 | 0.02744162 |
| MTNR1A | 12.2730164 | -1.6394793 | 0.00135737 | 0.02744388 |
| TLK1 | 1154.75141 | -0.4146357 | 0.00135983 | 0.02746203 |
| SAMD1 | 108.514442 | -0.5509032 | 0.00136594 | 0.02755377 |
| SYNPO2 | 1853.96087 | 1.48657655 | 0.00136802 | 0.02756412 |
| PRR12 | 958.689686 | -0.5760224 | 0.00138149 | 0.0278037 |
| PDF | 19.816098 | -1.0327413 | 0.00138316 | 0.02780537 |
| MCM6 | 421.781001 | -0.6534222 | 0.00138978 | 0.02787483 |
| SLITRK5 | 6.22754416 | -1.5388201 | 0.00138835 | 0.02787483 |
| CYSLTR2 | 9.59179348 | 1.36435445 | 0.00139225 | 0.0278926 |
| CCDC79 | 1.72630963 | 1.49650093 | 0.00139477 | 0.02789283 |
| UBE2T | 50.5326878 | -1.1073308 | 0.00139543 | 0.02789283 |
| CYTIP | 91.1643092 | 1.07216293 | 0.00140048 | 0.02793024 |
| EPSTI1 | 133.223497 | 0.77249949 | 0.00139975 | 0.02793024 |
| MRPL24 | 411.604492 | -0.5981415 | 0.00140499 | 0.02795682 |
| SPOPL | 810.603571 | 0.74133618 | 0.00140437 | 0.02795682 |
| AHCTF1P1 | 11.2114699 | -0.868518 | 0.00141134 | 0.028042 |
| LOC1019270 | 131.818656 | 0.69127198 | 0.00141246 | 0.028042 |
| ZNF831 | 11.1606371 | 1.37429114 | 0.00142365 | 0.02823232 |
| ITK | 21.9293646 | 1.32337114 | 0.00143263 | 0.02837841 |
| SNAP25 | 32.3155352 | -1.6252169 | 0.00143946 | 0.02848153 |
| ZEB1 | 1625.20311 | 0.70690341 | 0.00144473 | 0.02855361 |
| LINC00667 | 435.163276 | 0.58349405 | 0.00144652 | 0.02855706 |
| E2F1 | 210.102873 | -1.0563453 | 0.00145128 | 0.02861876 |
| BRIP1 | 119.926644 | -1.1053964 | 0.00145604 | 0.02868053 |
| MILR1 | 39.3232951 | 1.13344544 | 0.00145988 | 0.02872398 |
| RAB38 | 25.8871521 | -1.3711423 | 0.00146461 | 0.02878492 |
| NXPH2 | 284.108686 | 1.62944719 | 0.00147131 | 0.0288844 |
| AKR1B10 | 20.012381 | 1.5911946 | 0.00148494 | 0.02899318 |

| | | | | |
|---|---|---|---|---|
| COX6A1 | 1396.20748 | -0.6050737 | 0.00147899 | 0.02899318 |
| IGIP | 424.005829 | 0.59459427 | 0.00148353 | 0.02899318 |
| OTOA | 4.40539136 | 1.43931426 | 0.00148147 | 0.02899318 |
| PLCB1 | 727.423642 | 1.45111089 | 0.0014851 | 0.02899318 |
| CD300A | 165.709922 | 1.01227363 | 0.00149755 | 0.02920389 |
| CCDC137 | 204.245612 | -0.5126836 | 0.00150869 | 0.02926732 |
| FARSB | 485.074803 | -0.4908173 | 0.00151079 | 0.02926732 |
| KCNA3 | 24.6793099 | 1.18161341 | 0.00150585 | 0.02926732 |
| TCP10L | 17.8010776 | 1.19738959 | 0.00150927 | 0.02926732 |
| TRAPPC2 | 216.405084 | 0.54737081 | 0.00150804 | 0.02926732 |
| TSTD3 | 54.867222 | 0.91996324 | 0.00150247 | 0.02926732 |
| CHL1 | 14.4692145 | -1.6161702 | 0.00151359 | 0.02928937 |
| ARHGAP15 | 121.001076 | 1.00092051 | 0.00151934 | 0.02936841 |
| GABRA3 | 33.7975409 | -1.6116361 | 0.00152273 | 0.02937541 |
| PAPPA | 801.580257 | 1.50984894 | 0.00152472 | 0.02937541 |
| SCARNA16 | 127.648356 | -0.8714572 | 0.00152387 | 0.02937541 |
| IDH3G | 507.408287 | -0.2950119 | 0.0015305 | 0.0294025 |
| MPP6 | 1044.19467 | 1.17843046 | 0.00152846 | 0.0294025 |
| PLEKHG4B | 2041.61936 | -1.0243914 | 0.00153114 | 0.0294025 |
| RAB23 | 752.300689 | 0.86757535 | 0.0015345 | 0.0294228 |
| TRIM23 | 472.935211 | 0.38100748 | 0.00153554 | 0.0294228 |
| BVES | 112.697872 | 1.39057521 | 0.00154633 | 0.02959379 |
| PMPCA | 483.653955 | -0.4500768 | 0.00154783 | 0.02959379 |
| TRG-AS1 | 8.29341296 | 1.25769398 | 0.00155395 | 0.02967862 |
| RAB32 | 117.125978 | 0.64824332 | 0.00156584 | 0.02987326 |
| LAMTOR4 | 387.285617 | 0.41712254 | 0.00156854 | 0.02987679 |
| TNR | 2.712065 | -1.6208946 | 0.00156942 | 0.02987679 |
| CTBS | 307.039196 | 0.65243551 | 0.00158367 | 0.03010559 |
| RSRP1 | 660.875733 | 0.72026967 | 0.00158658 | 0.03010559 |
| SH3PXD2A | 11024.5558 | -0.7419111 | 0.00158572 | 0.03010559 |
| CHDC2 | 13.1726755 | -1.5756331 | 0.00159253 | 0.03012685 |
| COPS6 | 737.681825 | -0.481135 | 0.00159284 | 0.03012685 |
| PAK4 | 781.702373 | -0.8133537 | 0.00159172 | 0.03012685 |
| IGF2BP2 | 542.015584 | -1.5412335 | 0.00159724 | 0.03017768 |
| ACACA | 2078.23521 | -0.4435394 | 0.00160366 | 0.03026659 |
| STAT2 | 3164.70421 | 0.49696027 | 0.00161116 | 0.03037545 |
| APC2 | 35.4121764 | -1.3798412 | 0.00161421 | 0.03040029 |
| FLJ31104 | 7.2132584 | 1.09406216 | 0.00161654 | 0.03040179 |
| SYF2 | 346.226975 | 0.63485875 | 0.00161774 | 0.03040179 |
| LILRA1 | 47.5292939 | 1.20939823 | 0.0016228 | 0.03045895 |
| TBCD | 1236.20968 | -0.3926595 | 0.00162425 | 0.03045895 |
| KIAA0825 | 231.68004 | 0.8163375 | 0.00163249 | 0.030581 |
| GRIA2 | 5.5608161 | -1.6183002 | 0.00164344 | 0.03075329 |
| POLDIP2 | 1134.39862 | -0.4680379 | 0.00164964 | 0.0308365 |
| C9orf129 | 15.0271815 | -1.4023515 | 0.00165337 | 0.03087335 |
| GLTSCR1 | 226.203054 | -0.8675457 | 0.00166085 | 0.03095241 |
| LIMS2 | 468.944192 | -0.8762968 | 0.00166112 | 0.03095241 |
| STC2 | 130.502167 | -1.4926157 | 0.00166338 | 0.03096184 |
| SUPT6H | 2501.31431 | -0.2681252 | 0.00168141 | 0.03126419 |
| C10orf35 | 22.1245893 | -1.1472514 | 0.00168665 | 0.03131469 |

| | | | | |
|---|---|---|---|---|
| NME4 | 873.127687 | -0.7117093 | 0.00168768 | 0.03131469 |
| LINC01277 | 1.69621764 | 1.43017947 | 0.00170229 | 0.03155255 |
| SCML4 | 6.00842836 | 1.30498436 | 0.00170525 | 0.03157402 |
| RAB43 | 39.7794904 | -0.5998854 | 0.00171229 | 0.03167112 |
| AGPS | 739.299146 | 0.67050074 | 0.00171767 | 0.03170384 |
| WDR5 | 510.273618 | -0.3900488 | 0.00171726 | 0.03170384 |
| TLX1 | 10.0286466 | 1.54301906 | 0.00172198 | 0.03175009 |
| HIST2H2BE | 875.112893 | -0.8964036 | 0.00173067 | 0.03185284 |
| UBE2Q1 | 833.327736 | -0.2618728 | 0.00173117 | 0.03185284 |
| POM121C | 803.611042 | -0.491357 | 0.00174166 | 0.0320123 |
| SCD | 2092.2465 | -1.258293 | 0.00175423 | 0.03220974 |
| SLA | 528.934328 | 1.03464139 | 0.00175651 | 0.03221788 |
| ERMARD | 227.395089 | 0.56180886 | 0.00176498 | 0.03233951 |
| KIRREL3 | 4.21006064 | -1.5232235 | 0.00177394 | 0.03246991 |
| BIRC2 | 1104.7721 | 0.31639805 | 0.00179131 | 0.03275383 |
| ATAD2 | 550.299426 | -0.7826232 | 0.0017945 | 0.03277812 |
| HSPA1B | 26.5309834 | 1.18191487 | 0.00180336 | 0.03290576 |
| SEPT8 | 1137.69339 | 0.62694688 | 0.00181088 | 0.03300864 |
| CACNB1 | 133.149456 | -0.8207764 | 0.00181454 | 0.03304108 |
| HEPACAM | 11.6462748 | -1.6023091 | 0.00182669 | 0.03322796 |
| BLNK | 180.167666 | 0.96387367 | 0.00183362 | 0.03331959 |
| SH2B2 | 14.0801 | -1.1285235 | 0.00183908 | 0.03338433 |
| POTEF | 14.4147676 | -1.2812829 | 0.00185527 | 0.03364351 |
| ACLY | 2312.12286 | -0.4442432 | 0.00185858 | 0.0336689 |
| COX10 | 200.880261 | -0.5886137 | 0.00186888 | 0.03382056 |
| NAIP | 342.604339 | 0.56539673 | 0.0018762 | 0.0338834 |
| NME2 | 275.363198 | -0.7516044 | 0.00187507 | 0.0338834 |
| ZNF487 | 45.952682 | 0.93747786 | 0.00187884 | 0.03389626 |
| STXBP5L | 6.04142302 | -1.5921461 | 0.00189921 | 0.0342285 |
| HIVEP3 | 308.002256 | 1.14817907 | 0.00191595 | 0.03447709 |
| ZFAND5 | 2959.34573 | 0.70605194 | 0.00191692 | 0.03447709 |
| CDCA7 | 49.9616936 | -1.5080131 | 0.00191947 | 0.03448779 |
| LPCAT4 | 148.033614 | -0.8527451 | 0.00192152 | 0.03448929 |
| MSANTD4 | 460.869546 | -0.5000446 | 0.00192993 | 0.03460488 |
| SNORA80A | 18.5348982 | 1.35545634 | 0.00194518 | 0.03484288 |
| MARCH9 | 157.033649 | -0.5012808 | 0.00194991 | 0.03489204 |
| FN3K | 144.27091 | -0.8471207 | 0.00196042 | 0.03504455 |
| SET | 4061.29411 | -0.4232377 | 0.00196251 | 0.03504618 |
| DCT | 7.72263524 | 1.1631477 | 0.00196959 | 0.03513692 |
| ATE1-AS1 | 11.723148 | 1.42524186 | 0.00197748 | 0.03522447 |
| CPSF7 | 1418.5355 | -0.3430789 | 0.00198603 | 0.03522447 |
| GTSE1 | 28.374082 | -1.11227 | 0.00198651 | 0.03522447 |
| OTUB1 | 597.013392 | -0.4216225 | 0.00197944 | 0.03522447 |
| POLR2L | 971.293497 | -0.6097729 | 0.00198179 | 0.03522447 |
| ZNF562 | 966.410488 | 0.38347089 | 0.00198595 | 0.03522447 |
| TUBG1 | 396.984995 | -0.61993 | 0.00198895 | 0.0352322 |
| MED14OS | 2.76064576 | 1.27760341 | 0.00199292 | 0.03526714 |
| COL9A2 | 60.1715321 | 1.37902475 | 0.00199676 | 0.03529954 |
| CLUH | 830.662991 | -0.5569171 | 0.00201055 | 0.03533018 |
| E2F2 | 24.2538067 | -1.1316136 | 0.00200372 | 0.03533018 |

| | | | | |
|---|---|---|---|---|
| LINC00417 | 17.5959255 | 1.54183858 | 0.00200925 | 0.03533018 |
| NKX6-1 | 13.459662 | -1.5279959 | 0.00200585 | 0.03533018 |
| RBP4 | 95.5762866 | 1.56781645 | 0.00200818 | 0.03533018 |
| SRP68 | 993.51777 | -0.5754279 | 0.00200782 | 0.03533018 |
| EPHA1 | 4.18581159 | 1.47186554 | 0.0020137 | 0.03535032 |
| ZDHHC5 | 1668.79698 | -0.4807567 | 0.00201912 | 0.03541007 |
| RASL10A | 4.74617228 | -1.3825379 | 0.0020291 | 0.03554961 |
| DKK2 | 2029.09132 | -1.5824035 | 0.00204347 | 0.03576586 |
| VENTX | 119.306822 | 1.25417094 | 0.00204652 | 0.03578357 |
| GAB2 | 846.708939 | 0.72188002 | 0.00205329 | 0.03583079 |
| LOC729683 | 14.8042248 | 1.00317565 | 0.00205165 | 0.03583079 |
| PABPC1 | 7118.61875 | -0.6000677 | 0.00206292 | 0.03596307 |
| HGF | 85.1895756 | 1.20903111 | 0.00206715 | 0.03600122 |
| NRTN | 4.70017568 | -1.5330548 | 0.00207016 | 0.03601793 |
| ARMC2 | 100.804226 | 0.89771589 | 0.00207773 | 0.03611393 |
| BBOX1 | 1.32875708 | 1.47424627 | 0.0020895 | 0.03617553 |
| CPB2-AS1 | 10.1533558 | 1.24357769 | 0.00208891 | 0.03617553 |
| FBXO46 | 232.19465 | -0.6799195 | 0.00208585 | 0.03617553 |
| NUDCD2 | 157.229304 | 0.47976856 | 0.00208634 | 0.03617553 |
| KIAA1467 | 318.515922 | 0.78730169 | 0.00209564 | 0.0362462 |
| ADRA1A | 23.3400376 | -1.5725806 | 0.00211007 | 0.03642409 |
| KIAA1217 | 3891.30038 | -0.6688891 | 0.00210893 | 0.03642409 |
| SUMO4 | 29.853724 | 0.69258664 | 0.00211782 | 0.03652207 |
| CDADC1 | 128.536663 | 0.62484795 | 0.00212216 | 0.03656095 |
| FAM168B | 2472.40142 | -0.3397249 | 0.0021272 | 0.03661202 |
| IFT20 | 202.38328 | 0.46678695 | 0.00213491 | 0.03670884 |
| OMA1 | 213.304179 | 0.58725373 | 0.00213705 | 0.03670974 |
| HIATL2 | 58.2061199 | -0.5722018 | 0.00214016 | 0.03672719 |
| SENP7 | 839.16531 | 0.39039896 | 0.00214602 | 0.03679184 |
| GUCA1B | 21.2483771 | 0.92965781 | 0.00215135 | 0.03684741 |
| C19orf48 | 321.40122 | -0.5969687 | 0.00215954 | 0.03689266 |
| KMT2B | 1215.85003 | -0.4227335 | 0.00216238 | 0.03689266 |
| SLC25A25 | 424.906223 | 0.91456723 | 0.00215793 | 0.03689266 |
| STOML3 | 6.22218203 | 1.56654986 | 0.00216104 | 0.03689266 |
| ABLIM2 | 34.841937 | -1.0983886 | 0.00217661 | 0.03702762 |
| PAQR6 | 107.875763 | -1.2011282 | 0.00217488 | 0.03702762 |
| TMEM220-A | 2.33246163 | 1.45440761 | 0.00217543 | 0.03702762 |
| TBPL1 | 108.963802 | 0.65532732 | 0.00217924 | 0.0370365 |
| MCOLN3 | 11.9449639 | 1.34677638 | 0.00218388 | 0.03706046 |
| PHGDH | 288.313457 | -1.3693578 | 0.00218486 | 0.03706046 |
| IRF5 | 195.073432 | 0.96191193 | 0.00219238 | 0.03715216 |
| EMR3 | 4.58420212 | 1.50812427 | 0.00219807 | 0.03721277 |
| TSPO | 175.506638 | 0.89736821 | 0.00220179 | 0.03723997 |
| HPS5 | 773.559247 | 0.75221238 | 0.00220843 | 0.03731636 |
| ADRBK2 | 336.204353 | 0.82573534 | 0.00223142 | 0.03765758 |
| ASCL4 | 1.99346926 | 1.57136932 | 0.0022371 | 0.03765758 |
| PFKM | 2628.61986 | -0.5291315 | 0.00223753 | 0.03765758 |
| TNFRSF11B | 734.274616 | -1.4402414 | 0.00224324 | 0.03765758 |
| TP73 | 11.8422271 | -1.327318 | 0.00224254 | 0.03765758 |
| ZBTB45 | 133.211891 | -0.5105278 | 0.00223461 | 0.03765758 |

| | | | | |
|---|---|---|---|---|
| ZNF853 | 251.337883 | -0.5858667 | 0.00224361 | 0.03765758 |
| ZFPM1 | 119.47177 | -0.7265266 | 0.00225756 | 0.03785551 |
| KCNJ10 | 20.157932 | -1.5691187 | 0.00225999 | 0.03786018 |
| CCRL2 | 13.6468113 | 0.99694398 | 0.00226351 | 0.03788305 |
| RAB6C | 9.68137603 | -1.1668227 | 0.0022662 | 0.03789204 |
| MAFA | 7.74290844 | -1.5648252 | 0.00227217 | 0.03793089 |
| RELL2 | 72.8701386 | -1.0015033 | 0.00227283 | 0.03793089 |
| NDUFB9 | 473.667022 | -0.6598206 | 0.00228907 | 0.03814845 |
| SERPINE2 | 318.802024 | -1.2186833 | 0.00229021 | 0.03814845 |
| SDHC | 796.671851 | -0.5077297 | 0.0022958 | 0.03816937 |
| ZNF18 | 203.783687 | 0.48114025 | 0.00229365 | 0.03816937 |
| RAP2C-AS1 | 67.3901528 | 0.76444883 | 0.00229988 | 0.03820098 |
| TFPI2 | 20.1743434 | -1.5604717 | 0.00231085 | 0.03834703 |
| A2ML1 | 2.26219225 | 1.41522222 | 0.00231725 | 0.03841702 |
| SLFNL1 | 4.90327391 | 1.28875559 | 0.00232816 | 0.03856151 |
| UNC80 | 143.819261 | -1.3693423 | 0.00234929 | 0.03887496 |
| RPP30 | 241.904886 | 0.43526029 | 0.00235515 | 0.03893518 |
| CNTNAP2 | 12.8693127 | -1.3521869 | 0.00236992 | 0.03914254 |
| SLC35C1 | 487.809954 | -0.7704194 | 0.00237884 | 0.03925316 |
| PPFIBP2 | 327.098326 | 0.92320471 | 0.0023832 | 0.03928816 |
| LINC00673 | 6.0517173 | -1.5058857 | 0.00238858 | 0.03930317 |
| NOMO2 | 282.524282 | -0.6946329 | 0.00238675 | 0.03930317 |
| FANCI | 296.718394 | -0.9267192 | 0.00239454 | 0.03936444 |
| LOC1019271 | 10.7268381 | 1.15520612 | 0.00241663 | 0.0396836 |
| PQLC3 | 320.153179 | 0.66581295 | 0.00241847 | 0.0396836 |
| PTGES | 190.655792 | -1.2462995 | 0.00242278 | 0.03971741 |
| APLP1 | 320.713709 | -1.125309 | 0.00243001 | 0.03979869 |
| CELF5 | 5.5510553 | -1.4457848 | 0.00243735 | 0.03988192 |
| AFAP1-AS1 | 94.5466343 | -1.5414457 | 0.00245087 | 0.03991745 |
| NAA60 | 706.968921 | -0.4986945 | 0.00244671 | 0.03991745 |
| SHC1 | 2717.67389 | -0.4997136 | 0.00244788 | 0.03991745 |
| TNFSF8 | 106.030012 | 1.06223647 | 0.00244258 | 0.03991745 |
| UBQLN1 | 2043.47035 | -0.4371849 | 0.00245024 | 0.03991745 |
| VSIG10 | 296.606667 | -0.6783658 | 0.00245653 | 0.0399726 |
| CXCR2 | 15.2690353 | 1.01056637 | 0.0024615 | 0.039985 |
| KLHL8 | 304.870586 | 0.50129252 | 0.00246184 | 0.039985 |
| GPD1L | 584.581894 | 0.85992492 | 0.00246766 | 0.04004255 |
| FAM86C1 | 52.9203552 | -0.6274075 | 0.002476 | 0.04012838 |
| GRB14 | 52.2663526 | -1.5290012 | 0.00247979 | 0.04012838 |
| MANEA-AS1 | 32.9991073 | 0.79580314 | 0.00247841 | 0.04012838 |
| SLC25A44 | 506.748974 | -0.4910701 | 0.00248336 | 0.04014915 |
| FOXL2 | 3.1115344 | -1.4534435 | 0.0024929 | 0.04019257 |
| MUC1 | 232.246386 | -0.9537554 | 0.00249157 | 0.04019257 |
| SCRT1 | 1.46335665 | -1.532698 | 0.00249195 | 0.04019257 |
| KCNG2 | 7.50116547 | -1.5281966 | 0.00250127 | 0.04029064 |
| ACY1 | 18.50459 | -0.9279266 | 0.00250595 | 0.04029227 |
| C1orf116 | 3.76003512 | -1.3212519 | 0.00250517 | 0.04029227 |
| PTPRR | 4.58121532 | 1.45856998 | 0.00250915 | 0.0403068 |
| TSEN54 | 149.370835 | -0.8723183 | 0.00252675 | 0.04055243 |
| ACTR6 | 177.315103 | 0.4450235 | 0.00252921 | 0.04055493 |

| | | | | |
|---|---|---|---|---|
| PRR18 | 2.93358126 | -1.5519889 | 0.00253398 | 0.04059437 |
| HECA | 1236.70348 | 0.68125738 | 0.00254259 | 0.04065834 |
| NUDT8 | 35.9370935 | -0.889074 | 0.00254037 | 0.04065834 |
| RPRM | 65.4858054 | -1.5391229 | 0.00255341 | 0.04079421 |
| TBX3 | 25.5188493 | -1.343745 | 0.00255579 | 0.04079527 |
| HIST1H1B | 333.185146 | -1.0023404 | 0.00257739 | 0.04110216 |
| LINC00632 | 2.31269935 | -1.5418253 | 0.00258416 | 0.04110216 |
| LOC1019270 | 31.4362329 | 1.35249027 | 0.00258686 | 0.04110216 |
| SKINTL | 8.24894046 | 1.20498488 | 0.00258945 | 0.04110216 |
| TBX1 | 16.449054 | -1.4458652 | 0.00258347 | 0.04110216 |
| TBX21 | 3.86032949 | 1.33521002 | 0.00258544 | 0.04110216 |
| UBE2S | 193.173655 | -0.8299099 | 0.00259138 | 0.04110216 |
| NRIP3 | 18.8807186 | 1.40565546 | 0.00260763 | 0.04119969 |
| TAF6 | 485.852839 | -0.4266817 | 0.00260642 | 0.04119969 |
| TMC6 | 756.238212 | -0.8524716 | 0.00260271 | 0.04119969 |
| TTYH3 | 1108.718 | -0.785025 | 0.00260924 | 0.04119969 |
| ZNF142 | 732.91311 | -0.3777138 | 0.00260226 | 0.04119969 |
| FANCC | 243.432517 | -0.7837937 | 0.00261774 | 0.04129698 |
| FGFR1OP2 | 529.295339 | 0.38939645 | 0.00262594 | 0.04138922 |
| ZNF598 | 423.319377 | -0.6276844 | 0.00263096 | 0.04143108 |
| CNNM3 | 401.325258 | -0.4693776 | 0.00263917 | 0.04152328 |
| DDX58 | 449.713084 | 0.59916226 | 0.00264763 | 0.0416191 |
| AIM2 | 7.18798715 | 1.42934806 | 0.00265761 | 0.04170152 |
| BRCA2 | 230.878727 | -0.9256505 | 0.00265727 | 0.04170152 |
| SF1 | 2374.387 | -0.4394398 | 0.00267982 | 0.04197502 |
| SPNS3 | 16.6124083 | 1.43453784 | 0.00267759 | 0.04197502 |
| LMBR1 | 1097.7648 | -0.5616923 | 0.00268823 | 0.04206939 |
| ACSBG2 | 11.0410363 | 1.5357455 | 0.00269961 | 0.04217245 |
| CREG1 | 1214.91133 | 0.39598423 | 0.00269743 | 0.04217245 |
| ATP8B4 | 345.131251 | 0.99779798 | 0.00271142 | 0.04231931 |
| POLK | 901.368214 | 0.3202678 | 0.00271825 | 0.0423883 |
| MRPL38 | 396.703635 | -0.4635566 | 0.00272325 | 0.04242868 |
| PPAN | 26.6682761 | 0.76359407 | 0.0027335 | 0.04251306 |
| SMPD4 | 723.131715 | -0.4070775 | 0.00273146 | 0.04251306 |
| KRT14 | 33.2496729 | -1.5362673 | 0.00274135 | 0.04259745 |
| CNOT1 | 4255.0343 | -0.4009375 | 0.00274661 | 0.04264147 |
| CNIH3 | 20.1935479 | 1.07820898 | 0.00275224 | 0.04269131 |
| ZCCHC10 | 158.21385 | 0.49733118 | 0.00277864 | 0.04306278 |
| ASPRV1 | 40.3801062 | 1.05175014 | 0.00278482 | 0.0430827 |
| TLCD1 | 14.3662054 | -1.1793021 | 0.00278445 | 0.0430827 |
| C11orf35 | 45.0197732 | -0.7094954 | 0.0027958 | 0.04310096 |
| FAM229B | 238.940116 | 0.87006295 | 0.00279043 | 0.04310096 |
| MRPL41 | 326.905432 | -0.6257164 | 0.00279513 | 0.04310096 |
| SCAMP3 | 703.975153 | -0.4934911 | 0.00279389 | 0.04310096 |
| NAGPA | 106.707259 | 0.34875874 | 0.00280667 | 0.04323052 |
| SHCBP1 | 78.7332453 | -1.0013193 | 0.00281374 | 0.04330158 |
| SPATA41 | 16.6038716 | -0.8870349 | 0.00282935 | 0.04350367 |
| ESRRA | 369.902958 | -0.7247024 | 0.00283353 | 0.04352992 |
| RUNX3 | 53.4176828 | 1.15928662 | 0.00284014 | 0.04359338 |
| FAM106CP | 33.7272346 | -1.5032595 | 0.00286028 | 0.04379053 |

| | | | | |
|---|---|---|---|---|
| GPATCH8 | 2211.86536 | -0.4128738 | 0.00285688 | 0.04379053 |
| USP53 | 4313.66273 | 0.78816581 | 0.00286045 | 0.04379053 |
| CPO | 8.30705827 | 1.20774088 | 0.002874 | 0.04392154 |
| MINOS1P1 | 166.634016 | 0.72689329 | 0.00287269 | 0.04392154 |
| WEE2-AS1 | 13.6623406 | 0.94514495 | 0.00288495 | 0.0440506 |
| ASIC3 | 43.1203869 | -0.9633623 | 0.00290188 | 0.04427065 |
| PEX5L | 59.8901658 | -1.4467256 | 0.00291518 | 0.04443498 |
| ATP7A | 707.595756 | 0.3641526 | 0.00292711 | 0.04453973 |
| SPRY1 | 624.173493 | 1.01666815 | 0.00292631 | 0.04453973 |
| DLL1 | 80.4396858 | 1.35367564 | 0.0029332 | 0.0445552 |
| SOX12 | 233.512548 | -0.854358 | 0.00293279 | 0.0445552 |
| CETN4P | 1.54058385 | 1.5101748 | 0.00294511 | 0.04469763 |
| LOC728819 | 5.08743447 | -1.4433427 | 0.00296737 | 0.04499658 |
| DNAJB1 | 1740.88621 | 0.71985092 | 0.00298027 | 0.04515332 |
| ACSM5 | 40.5575885 | 1.16505162 | 0.00298991 | 0.04526034 |
| FZD2 | 963.904521 | -0.6206861 | 0.00299265 | 0.04526281 |
| C8orf82 | 177.730391 | -0.6554342 | 0.00299704 | 0.04529028 |
| TTYH1 | 10.6865586 | -1.5046439 | 0.00300111 | 0.04531293 |
| FRMPD4 | 2.15636193 | -1.5252861 | 0.00300602 | 0.04534814 |
| NRG4 | 51.5245271 | -1.4383975 | 0.0030115 | 0.0453919 |
| CALY | 9.79209512 | -1.5086657 | 0.00301787 | 0.04540999 |
| PTPN22 | 46.8368537 | 1.11899181 | 0.00301778 | 0.04540999 |
| CCND1 | 21355.0724 | -0.9160399 | 0.00302843 | 0.04552993 |
| LOC1019276 | 4.95133738 | 1.5054633 | 0.00303494 | 0.04558889 |
| CAPN15 | 521.236132 | -0.6302792 | 0.00303993 | 0.04562495 |
| TRIM46 | 55.9294482 | -1.3384695 | 0.00304394 | 0.0456461 |
| ANKRD39 | 72.6868642 | -0.5138767 | 0.00306625 | 0.04573696 |
| EPG5 | 1597.81399 | 0.67103874 | 0.00305493 | 0.04573696 |
| GPR98 | 23.424167 | -1.4100518 | 0.00306311 | 0.04573696 |
| HSPA12A | 379.714745 | 1.27385326 | 0.00306661 | 0.04573696 |
| LOC283038 | 5.43118841 | -1.4127541 | 0.0030682 | 0.04573696 |
| PTPRC | 971.915685 | 0.92983124 | 0.00305896 | 0.04573696 |
| TRPC1 | 603.414684 | 0.63147171 | 0.00306675 | 0.04573696 |
| PLA1A | 5.77362796 | 1.2331817 | 0.00307923 | 0.04586261 |
| ARHGAP11B | 30.9314246 | -1.070385 | 0.00309318 | 0.04603126 |
| LOC1019272 | 9.73653821 | 1.48128226 | 0.00310189 | 0.04608291 |
| UBA7 | 655.700195 | 0.78621443 | 0.00310161 | 0.04608291 |
| CST7 | 11.8065323 | 1.32276445 | 0.00312116 | 0.04633019 |
| AQP1 | 1810.00775 | 1.44452043 | 0.00314321 | 0.04648228 |
| DCAF7 | 2481.52292 | -0.4611992 | 0.00313894 | 0.04648228 |
| RNF11 | 1581.43529 | 0.62145455 | 0.00314462 | 0.04648228 |
| SLC30A3 | 17.8307891 | 1.51676004 | 0.00314445 | 0.04648228 |
| ZBED8 | 190.258235 | 0.60551393 | 0.0031362 | 0.04648228 |
| ANKRD20A8I | 52.1625303 | -1.3376395 | 0.00314779 | 0.04649004 |
| KIF23 | 362.283681 | -1.0644221 | 0.00316352 | 0.04668319 |
| ARID5B | 1255.1713 | 1.1487315 | 0.00317844 | 0.04675633 |
| HERC2 | 3874.27986 | -0.2988698 | 0.00317533 | 0.04675633 |
| IPCEF1 | 85.4745127 | 1.08484079 | 0.00317911 | 0.04675633 |
| SAP130 | 472.205882 | -0.4073437 | 0.00317682 | 0.04675633 |
| ITGB3 | 115.296176 | 1.04267228 | 0.00318391 | 0.04678518 |

| | | | | |
|---|---|---|---|---|
| NELFB | 568.186493 | -0.3349292 | 0.00318639 | 0.04678518 |
| NEB | 586.314523 | 1.20512144 | 0.00319691 | 0.04690041 |
| SHROOM3 | 244.47223 | 1.31128813 | 0.00320346 | 0.04695738 |
| IKZF3 | 61.0426669 | 1.14033402 | 0.00321014 | 0.04701606 |
| ACO2 | 1351.79574 | -0.6646326 | 0.00321501 | 0.04704831 |
| BLOC1S3 | 157.965236 | -0.5164942 | 0.00321926 | 0.0470713 |
| HIF3A | 1603.4172 | -1.1423435 | 0.00323248 | 0.04722536 |
| FAM13A-AS1 | 94.0139253 | 0.72120814 | 0.00323642 | 0.04724365 |
| THEMIS | 14.2077361 | 1.16494318 | 0.00324265 | 0.04729544 |
| AEBP1 | 4151.15313 | 1.29254512 | 0.00325749 | 0.04739388 |
| CYC1 | 609.978737 | -0.565131 | 0.00325513 | 0.04739388 |
| MRPL14 | 147.599837 | 0.45953914 | 0.00325582 | 0.04739388 |
| WDR11-AS1 | 5.53914016 | 1.26910206 | 0.00326337 | 0.04744027 |
| SOX11 | 328.616986 | -1.5071619 | 0.0032672 | 0.04745664 |
| AMH | 92.6189154 | -1.3987401 | 0.00327645 | 0.04755171 |
| CYP2D7P | 18.4793639 | 0.77439569 | 0.00328765 | 0.04765386 |
| ZNF438 | 187.129874 | 0.60317536 | 0.0032889 | 0.04765386 |
| CD244 | 5.91877599 | 1.32160104 | 0.00330233 | 0.04778337 |
| CUX1 | 3267.02176 | -0.5227282 | 0.00330786 | 0.04778337 |
| ORC6 | 37.339782 | -1.0721834 | 0.00330613 | 0.04778337 |
| SLC37A4 | 228.659393 | -0.4484349 | 0.00330871 | 0.04778337 |
| FIZ1 | 139.399511 | -0.6305609 | 0.00331223 | 0.04779502 |
| STON1 | 130.432274 | 0.83506459 | 0.00332444 | 0.04793187 |
| FUBP3 | 960.913236 | -0.2722478 | 0.0033305 | 0.04794063 |
| RGP1 | 422.969312 | -0.5132597 | 0.00332826 | 0.04794063 |
| HTRA1 | 1337.63966 | 0.91616869 | 0.00334278 | 0.04807809 |
| EHD1 | 4132.50246 | -0.7875156 | 0.00334992 | 0.04813384 |
| HMP19 | 2.75663015 | -1.5068792 | 0.00335487 | 0.04813384 |
| LSMEM1 | 16.3966629 | 0.82434466 | 0.00335358 | 0.04813384 |
| FAM135A | 517.102822 | 0.50986503 | 0.00337756 | 0.04822348 |
| LAMC1 | 7265.36256 | -0.6091379 | 0.00337371 | 0.04822348 |
| MX1 | 991.154475 | 0.59723847 | 0.00337286 | 0.04822348 |
| PSAT1 | 214.051277 | -1.1484501 | 0.00336393 | 0.04822348 |
| RPUSD3 | 145.896273 | -0.5005441 | 0.00337714 | 0.04822348 |
| SHMT2 | 1069.43287 | -0.7291454 | 0.00337009 | 0.04822348 |
| DPY19L1P1 | 43.5684963 | 0.88693367 | 0.00338499 | 0.04825306 |
| INTS6-AS1 | 27.2466626 | 0.60810292 | 0.00338512 | 0.04825306 |
| LOC728730 | 45.1218994 | 0.75325841 | 0.00339456 | 0.04834847 |
| TNXB | 12.5352774 | -1.2267921 | 0.00339961 | 0.04838113 |
| CD226 | 18.3975941 | 0.95740901 | 0.0034048 | 0.04841583 |
| ERBB2IP | 3192.35647 | 0.29779565 | 0.00340924 | 0.04843981 |
| TDRKH | 120.46616 | -0.7607495 | 0.00342438 | 0.04857655 |
| TXNDC15 | 957.192443 | 0.46936303 | 0.00342275 | 0.04857655 |
| SAT1 | 2458.71124 | 0.60806402 | 0.00343542 | 0.04869384 |
| PPP1R1C | 6.29176205 | -1.4651195 | 0.00344117 | 0.04873611 |
| RTN4R | 61.5644347 | -1.2904459 | 0.00345083 | 0.04883351 |
| CSMD2 | 37.3880403 | -1.3468282 | 0.00346178 | 0.04894907 |
| ARHGAP11A | 207.151778 | -0.9486742 | 0.00346886 | 0.04897054 |
| SLC6A17 | 28.3566681 | 1.45261771 | 0.00346769 | 0.04897054 |
| C1QL1 | 129.455126 | -1.192896 | 0.00347296 | 0.04898913 |

| | | | | |
|---|---|---|---|---|
| P2RY14 | 56.5488825 | 1.42817758 | 0.00347834 | 0.04902571 |
| RANBP10 | 485.878456 | -0.4580209 | 0.00348788 | 0.04908147 |
| SURF2 | 76.3025261 | -0.6746234 | 0.0034851 | 0.04908147 |
| LMNB2 | 721.672312 | -0.6396509 | 0.00351361 | 0.04919182 |
| MATN2 | 713.979172 | 1.33846953 | 0.00352369 | 0.04919182 |
| NDUFS1 | 1557.43859 | -0.4893952 | 0.00350017 | 0.04919182 |
| NUMBL | 847.965807 | -0.5791738 | 0.00350289 | 0.04919182 |
| PSMD10 | 342.652407 | 0.34503791 | 0.0035231 | 0.04919182 |
| RAP1GAP2 | 397.827078 | -1.0095698 | 0.00350591 | 0.04919182 |
| SNORA71C | 7.95084917 | -1.2633236 | 0.0035194 | 0.04919182 |
| SRSF10 | 77.4348237 | 0.5612431 | 0.00351677 | 0.04919182 |
| THUMPD3 | 637.728991 | -0.4953759 | 0.00352034 | 0.04919182 |
| TNIK | 203.637933 | 1.20465955 | 0.00351484 | 0.04919182 |
| RPL23AP32 | 115.135701 | 0.89084066 | 0.00353525 | 0.04931406 |
| TRAF3IP3 | 47.3773251 | 1.08864812 | 0.00354706 | 0.04943964 |
| LCK | 10.8696077 | 1.11586237 | 0.0035597 | 0.04957646 |
| ZMYM6NB | 148.180609 | 0.62462535 | 0.00356321 | 0.04958619 |
| EIF2AK1 | 1800.25327 | -0.3864185 | 0.00356821 | 0.04961639 |
| C9orf16 | 360.109414 | -0.6435515 | 0.00358119 | 0.04967906 |
| FBXW5 | 859.404595 | -0.3827278 | 0.00358098 | 0.04967906 |
| MTERF | 174.552108 | 0.45231153 | 0.00357683 | 0.04967906 |
| WNT10A | 10.885127 | -1.4135691 | 0.00358691 | 0.04971931 |
| SIN3A | 1755.37184 | -0.5080286 | 0.00359894 | 0.0498467 |
| LOC653602 | 11.1288635 | -1.2910275 | 0.00362378 | 0.05015126 |
| MATK | 48.3858419 | -1.3876817 | 0.00363388 | 0.05025154 |
| NMRAL1 | 274.484908 | -0.520026 | 0.00364094 | 0.05030955 |
| GAL3ST3 | 8.21105171 | -1.418312 | 0.00365876 | 0.05051619 |
| TNNT2 | 1650.11178 | -1.1280174 | 0.00366174 | 0.05051759 |
| SLC22A18 | 215.050917 | -0.6036876 | 0.00366811 | 0.05056584 |
| LOC374443 | 291.380026 | 0.62190905 | 0.00368166 | 0.05071293 |
| C19orf68 | 49.4224164 | -0.6043642 | 0.00370941 | 0.05103679 |
| PDCD1 | 6.33562299 | 1.40882393 | 0.00371098 | 0.05103679 |
| ABCC11 | 4.45788913 | -1.1799602 | 0.0037244 | 0.0511451 |
| GPAA1 | 950.589618 | -0.4759048 | 0.00372757 | 0.0511451 |
| THAP2 | 74.9523733 | 0.59111347 | 0.00372634 | 0.0511451 |
| STK32A | 173.689622 | 1.4197198 | 0.00373431 | 0.05119762 |
| P2RX7 | 213.792652 | 0.91472939 | 0.00373735 | 0.0511993 |
| FAM171A2 | 208.431893 | -0.6064887 | 0.00374405 | 0.05125117 |
| CASP2 | 568.817525 | -0.470954 | 0.00375085 | 0.05130444 |
| RNGTT | 394.952804 | 0.56603334 | 0.0037557 | 0.05133084 |
| C19orf84 | 2.15855958 | -1.4891186 | 0.00376768 | 0.05145269 |
| JPH2 | 80.6487556 | -1.1759404 | 0.00377632 | 0.05145269 |
| SMC2 | 483.678492 | -0.4999722 | 0.00377202 | 0.05145269 |
| TMSB4X | 5949.29007 | 0.49160454 | 0.0037761 | 0.05145269 |
| ATIC | 691.229388 | -0.7748901 | 0.00378178 | 0.0514873 |
| GLS | 2076.50844 | 0.75206033 | 0.00378542 | 0.05149197 |
| PIK3IP1 | 553.269914 | 0.93186551 | 0.00378798 | 0.05149197 |
| PSTPIP2 | 137.818774 | 0.80322356 | 0.00381008 | 0.05175231 |
| MORN1 | 51.3371211 | 0.84891093 | 0.00381838 | 0.0518096 |
| PRKAG2-AS1 | 10.4523365 | 1.20140471 | 0.00382018 | 0.0518096 |

| | | | | |
|---|---|---|---|---|
| BRI3BP | 128.277167 | -0.6591681 | 0.00382376 | 0.05181807 |
| COL24A1 | 32.4034502 | 1.10207995 | 0.00382904 | 0.05184968 |
| ROGDI | 331.252634 | -0.6941741 | 0.0038789 | 0.05248456 |
| RPS29 | 1123.54262 | 0.67267207 | 0.00388458 | 0.05252096 |
| STK16 | 224.112595 | -0.4298485 | 0.00388863 | 0.05253527 |
| ATXN2L | 1553.80302 | -0.4820157 | 0.00389738 | 0.05261312 |
| CHAF1A | 175.098804 | -0.7252597 | 0.00390663 | 0.0526169 |
| ZNF394 | 312.013309 | 0.33759042 | 0.00390434 | 0.0526169 |
| ZNF699 | 89.2600049 | 0.64630416 | 0.00390655 | 0.0526169 |
| EZH1 | 972.572201 | 0.59210823 | 0.00391505 | 0.05268992 |
| ZFYVE9 | 1015.76131 | 0.658123 | 0.00391816 | 0.05269142 |
| CDH23 | 2780.14431 | 1.30474793 | 0.00392325 | 0.05271959 |
| DBI | 489.686461 | -0.5077332 | 0.00392883 | 0.0527543 |
| SFRP2 | 26626.5836 | 1.26512037 | 0.00393184 | 0.05275438 |
| THUMPD1 | 1149.73997 | 0.38272192 | 0.00395106 | 0.05297185 |
| ENDOG | 100.884023 | -0.857078 | 0.00396906 | 0.05317268 |
| CRMP1 | 287.447831 | -1.2687385 | 0.00397445 | 0.05318157 |
| TTC37 | 2229.48872 | 0.32799035 | 0.00397577 | 0.05318157 |
| KIAA0895L | 452.122542 | -0.6828871 | 0.00399117 | 0.05324371 |
| LOC1019282 | 6.07772508 | -1.4650541 | 0.00399252 | 0.05324371 |
| SRRT | 1005.28008 | -0.3741494 | 0.00398759 | 0.05324371 |
| ZNF683 | 2.13867688 | 1.42196789 | 0.00399159 | 0.05324371 |
| ACSL1 | 942.53583 | 0.82954358 | 0.0039999 | 0.05330163 |
| KIF2C | 32.1922651 | -0.9428833 | 0.00400433 | 0.05332037 |
| NAV3 | 172.516836 | 1.4236052 | 0.00401639 | 0.0534405 |
| CCDC3 | 499.56544 | -1.3959137 | 0.00402359 | 0.05345538 |
| ZNF285 | 77.9659753 | 0.64521124 | 0.00402296 | 0.05345538 |
| PHB | 1189.45674 | -0.4170433 | 0.00404127 | 0.0536497 |
| ZDHHC24 | 126.254234 | -0.5541912 | 0.00404435 | 0.05365011 |
| RCBTB2 | 854.64281 | 0.53727152 | 0.00405581 | 0.05372111 |
| SFRP4 | 2848.9995 | 1.4552105 | 0.00405488 | 0.05372111 |
| PNRC1 | 2685.09502 | 0.67313357 | 0.00406938 | 0.05380039 |
| RAD51AP1 | 52.4767029 | -0.8736393 | 0.00406625 | 0.05380039 |
| VGLL4 | 2794.24916 | -0.9835649 | 0.00407097 | 0.05380039 |
| CAMLG | 455.358184 | 0.50583234 | 0.00408288 | 0.05391727 |
| LINC00630 | 98.2719017 | 0.41174733 | 0.0040914 | 0.05398932 |
| ZIC2 | 2853.9414 | -0.6692618 | 0.00409591 | 0.05400829 |
| TMED1 | 240.428736 | 0.4338828 | 0.00410192 | 0.05404697 |
| C11orf21 | 11.6607747 | 1.27624885 | 0.00411116 | 0.05408932 |
| KIAA0100 | 5252.63161 | -0.3150958 | 0.00411128 | 0.05408932 |
| SLC19A1 | 90.9028422 | -0.8676169 | 0.00412829 | 0.0542725 |
| BTBD8 | 22.4478222 | 0.98328613 | 0.00415344 | 0.05456235 |
| BOP1 | 182.634935 | -0.5392051 | 0.00416746 | 0.05469173 |
| STAT5A | 469.596612 | 0.50226888 | 0.00416951 | 0.05469173 |
| ASPHD1 | 3.2033583 | -1.4483584 | 0.004198 | 0.05502447 |
| FIG4 | 251.917675 | 0.62762689 | 0.00420308 | 0.05504998 |
| CLEC17A | 10.7647456 | 1.37964341 | 0.00421681 | 0.05518873 |
| ALDOA | 10177.4213 | -0.6731153 | 0.0042219 | 0.05521379 |
| GZMH | 5.45880893 | 1.34560473 | 0.00422646 | 0.05521379 |
| HIST1H2BM | 57.4956526 | -1.029034 | 0.00422814 | 0.05521379 |

| | | | | |
|---|---|---|---|---|
| CDKAL1 | 341.026633 | 0.49839512 | 0.00425792 | 0.0555202 |
| ZNF345 | 131.176292 | 0.49427418 | 0.00425787 | 0.0555202 |
| OSBPL8 | 1816.74921 | 0.42560287 | 0.00426997 | 0.05563615 |
| CH25H | 46.7467649 | 1.20413206 | 0.00427921 | 0.05571521 |
| ANKRD13B | 584.243642 | -0.9982559 | 0.0043042 | 0.05596396 |
| RPL13A | 1657.98562 | 0.53817452 | 0.00430468 | 0.05596396 |
| NME8 | 4.78623948 | 1.30355961 | 0.00432819 | 0.05622814 |
| ADAT2 | 99.2355043 | 0.72830565 | 0.00433678 | 0.05628406 |
| CSF2RB | 121.027558 | 0.86490144 | 0.0043389 | 0.05628406 |
| CYTH2 | 686.297052 | -0.5342585 | 0.00434713 | 0.05634925 |
| FAM110A | 53.9211951 | -0.6002888 | 0.00436192 | 0.05649944 |
| SLC26A7 | 279.090127 | -1.4341303 | 0.00438103 | 0.05670521 |
| CIZ1 | 1028.07374 | -0.3633949 | 0.00438915 | 0.05676855 |
| APOL3 | 162.018703 | 0.85877188 | 0.00442718 | 0.05691542 |
| CEP135 | 206.394112 | 0.53861112 | 0.00442705 | 0.05691542 |
| H19 | 13475.5423 | -1.4620329 | 0.00442983 | 0.05691542 |
| LRIT3 | 8.68846458 | 1.04197954 | 0.00442334 | 0.05691542 |
| MTSS1L | 1532.41335 | -0.806025 | 0.00440688 | 0.05691542 |
| PI4KB | 1410.24429 | -0.2615663 | 0.00441218 | 0.05691542 |
| PIP5K1A | 1264.55959 | -0.4417414 | 0.00442327 | 0.05691542 |
| RTKN | 289.876196 | -0.6289715 | 0.00441663 | 0.05691542 |
| SGK1 | 2778.67963 | 0.97949674 | 0.00441634 | 0.05691542 |
| SMAD5 | 2231.53611 | 0.45980737 | 0.00443287 | 0.05691542 |
| CENPE | 140.260413 | -1.0009327 | 0.00445076 | 0.05710347 |
| IDH3A | 850.762888 | -0.7592703 | 0.00445792 | 0.05712337 |
| LRRC38 | 94.9163077 | -1.3166308 | 0.00445881 | 0.05712337 |
| SNORA53 | 822.408514 | -0.806796 | 0.00446305 | 0.05713619 |
| PSMD1 | 1447.79871 | -0.323311 | 0.00447319 | 0.05722428 |
| C10orf2 | 169.570123 | -0.8307185 | 0.00447881 | 0.05725458 |
| GATSL2 | 360.967222 | -0.5180188 | 0.00449605 | 0.05743186 |
| SYCP2 | 95.6139762 | 1.28486746 | 0.00450228 | 0.05743186 |
| SYVN1 | 743.308005 | -0.4836508 | 0.00450248 | 0.05743186 |
| FAM64A | 12.884585 | -1.1623211 | 0.0045633 | 0.05816554 |
| EHD3 | 185.320994 | -1.0002367 | 0.00457101 | 0.05822161 |
| PKD1L1 | 25.6654873 | 0.75573502 | 0.00459866 | 0.05853148 |
| ANKRD22 | 106.482737 | 1.35215537 | 0.00461281 | 0.05854213 |
| EDC3 | 547.469837 | -0.5021533 | 0.0046122 | 0.05854213 |
| HIST1H2BL | 71.5843595 | -0.8752459 | 0.00460733 | 0.05854213 |
| OLFM4 | 107.744791 | -1.3098076 | 0.00460406 | 0.05854213 |
| USP22 | 5114.42123 | -0.2822917 | 0.00462872 | 0.0587017 |
| CACFD1 | 246.951225 | -0.5324579 | 0.00463265 | 0.05870916 |
| ATP5H | 864.491629 | -0.5317001 | 0.00463893 | 0.05874643 |
| MEF2BNB | 102.175816 | -0.404804 | 0.00464813 | 0.05882061 |
| OGDHL | 631.046674 | -1.2504116 | 0.00466178 | 0.05895085 |
| NFKBIA | 2331.13834 | 0.64827695 | 0.00467163 | 0.05903299 |
| REV3L | 1414.28752 | 0.56243808 | 0.0046803 | 0.0590489 |
| SLC6A4 | 129.222134 | 1.45222422 | 0.00468029 | 0.0590489 |
| UNC13C | 6.82379954 | -1.3676112 | 0.00468296 | 0.0590489 |
| ABCF3 | 628.150914 | -0.2592692 | 0.00469203 | 0.05912094 |
| SERPINB10 | 10.660512 | -1.450311 | 0.00470731 | 0.05922863 |

| | | | | |
|---|---|---|---|---|
| TNFRSF12A | 109.989699 | -1.0151776 | 0.00470439 | 0.05922863 |
| ENOSF1 | 464.894621 | 0.60488755 | 0.00472068 | 0.05935432 |
| CDCA2 | 77.939032 | -1.2074363 | 0.0047297 | 0.05942527 |
| CNN2 | 821.020199 | 0.91201053 | 0.0047454 | 0.05957933 |
| LYZ | 460.679776 | 1.08430769 | 0.00475889 | 0.05957933 |
| PAFAH1B3 | 97.7260217 | -0.6870667 | 0.00475758 | 0.05957933 |
| SUCNR1 | 9.32426093 | 1.33288117 | 0.00475854 | 0.05957933 |
| ZER1 | 975.79909 | -0.2811065 | 0.00475494 | 0.05957933 |
| TF | 891.361795 | -1.4407533 | 0.00477931 | 0.05979233 |
| NELL2 | 46.4406824 | -1.2348406 | 0.00479087 | 0.05989439 |
| DCPS | 134.600549 | -0.4296463 | 0.00479464 | 0.0598989 |
| GINS2 | 51.266067 | -1.126402 | 0.00480145 | 0.05994149 |
| NR2F1-AS1 | 328.316341 | 1.01990095 | 0.00481716 | 0.06009498 |
| NOL12 | 148.132498 | 0.49976185 | 0.00482147 | 0.06010606 |
| CCDC175 | 11.8188737 | 1.44838667 | 0.00483091 | 0.06013848 |
| DCHS2 | 378.109381 | -1.3264042 | 0.00482999 | 0.06013848 |
| TRIT1 | 157.069383 | 0.67146224 | 0.00483648 | 0.06016532 |
| CADM3 | 24.7724202 | -1.3772983 | 0.00485525 | 0.06022844 |
| DPM3 | 120.0774 | -0.5833015 | 0.00485226 | 0.06022844 |
| GBA2 | 1240.68969 | -0.4800638 | 0.00485189 | 0.06022844 |
| RAI1 | 874.337993 | -0.5385548 | 0.00484841 | 0.06022844 |
| APOBEC3B | 10.328715 | -1.0161492 | 0.00486676 | 0.06032865 |
| RSF1 | 1468.00614 | -0.3271816 | 0.00487094 | 0.06033796 |
| KMT2D | 4134.92374 | -0.4465098 | 0.00488179 | 0.06039952 |
| RPS16 | 4029.24921 | -0.7742386 | 0.00488278 | 0.06039952 |
| PPP1R12B | 2184.45996 | 0.55356658 | 0.00489262 | 0.06044266 |
| ULK1 | 928.997369 | -0.6191068 | 0.00489314 | 0.06044266 |
| RBM4 | 718.766728 | -0.3605022 | 0.00489716 | 0.06044983 |
| NPHP3 | 199.792151 | 0.46990021 | 0.00490064 | 0.06045042 |
| REPIN1 | 849.548658 | -0.5586223 | 0.00491104 | 0.06053627 |
| CLK2 | 696.250375 | -0.4781837 | 0.00492035 | 0.06060847 |
| LOC1019272 | 2.28517208 | 1.44162799 | 0.00492845 | 0.06066575 |
| HCG11 | 355.882328 | 0.95391719 | 0.00493426 | 0.06069484 |
| CXCL16 | 556.142166 | 0.6985937 | 0.00494218 | 0.0607074 |
| NSUN5P1 | 303.427761 | 0.70817596 | 0.00494189 | 0.0607074 |
| KHNYN | 1132.28657 | 0.50750961 | 0.00494837 | 0.06074095 |
| ATP2A2 | 4089.59735 | -0.4591633 | 0.00495559 | 0.06074487 |
| RGS18 | 75.3373293 | 1.10622125 | 0.00495502 | 0.06074487 |
| TRAF3IP2-AS | 99.1558169 | 0.93859921 | 0.00496288 | 0.06079183 |
| CCNH | 425.10051 | 0.42582614 | 0.00498456 | 0.06101495 |
| PDE1B | 61.8168712 | 0.75225524 | 0.00498939 | 0.0610316 |
| NSUN7 | 80.6799572 | -0.8050773 | 0.00499627 | 0.06107325 |
| GORASP2 | 1025.03431 | -0.4126538 | 0.00500297 | 0.06111265 |
| GALNT13 | 17.4973658 | -1.3780566 | 0.00501755 | 0.0612482 |
| GDA | 13.8464772 | -1.4407077 | 0.00502229 | 0.06126353 |
| CKAP2 | 541.196848 | -0.675572 | 0.00503397 | 0.06131414 |
| HNRNPH3 | 1170.69953 | -0.3901552 | 0.00503689 | 0.06131414 |
| RHOT2 | 749.839717 | -0.4477283 | 0.00503195 | 0.06131414 |
| CDC45 | 26.4369442 | -1.2483797 | 0.00504121 | 0.06132426 |
| SNORA52 | 20.1762829 | 0.96220423 | 0.00505174 | 0.06140987 |

| | | | | |
|---|---|---|---|---|
| UVRAG | 475.716176 | 0.40210903 | 0.00505676 | 0.0614285 |
| RWDD1 | 424.771097 | 0.52279861 | 0.00507325 | 0.06158619 |
| ROR1 | 335.621477 | 1.18433441 | 0.00511752 | 0.06208076 |
| FUT7 | 3.77103372 | 1.21966056 | 0.00512505 | 0.06212934 |
| SLC22A4 | 35.7180829 | 0.85651516 | 0.0051394 | 0.06226041 |
| VANGL2 | 428.511389 | -0.5936418 | 0.0051465 | 0.06230341 |
| AOAH | 228.152811 | 1.00970322 | 0.00515458 | 0.06231548 |
| PM20D1 | 6.3717607 | 1.41860078 | 0.00515133 | 0.06231548 |
| MFSD3 | 118.590727 | -0.629548 | 0.00516234 | 0.0623664 |
| AOAH-IT1 | 3.30808031 | 1.40935483 | 0.00516938 | 0.06240868 |
| KLHDC7A | 165.061482 | -1.4165095 | 0.00519698 | 0.06269883 |
| NRK | 7.74432513 | 1.39998411 | 0.00520416 | 0.06274246 |
| LINC00486 | 1.68177761 | 1.39139492 | 0.00522829 | 0.06275857 |
| MISP | 44.2150991 | -1.3161495 | 0.00522136 | 0.06275857 |
| MPPE1 | 360.549037 | 0.51814528 | 0.00521268 | 0.06275857 |
| RNF146 | 778.089527 | 0.6140877 | 0.00521008 | 0.06275857 |
| THEM6 | 256.081564 | -0.6843767 | 0.00522387 | 0.06275857 |
| UBXN4 | 2369.71476 | -0.2805858 | 0.00523048 | 0.06275857 |
| XCL1 | 2.02661592 | 1.35872006 | 0.00522746 | 0.06275857 |
| MAP1LC3B | 1357.04694 | -0.4506641 | 0.00524148 | 0.06278025 |
| PHYHIPL | 11.1751061 | -1.3710769 | 0.00524224 | 0.06278025 |
| SLC12A8 | 17.8157457 | -1.0967189 | 0.00524299 | 0.06278025 |
| SNX22 | 22.5688493 | 1.11254228 | 0.00525751 | 0.06291131 |
| SPTBN2 | 296.987874 | -1.0012533 | 0.00526981 | 0.06297393 |
| ZFP41 | 387.599033 | -0.5247829 | 0.00526991 | 0.06297393 |
| MTMR4 | 1563.20035 | -0.4649125 | 0.00527748 | 0.06302167 |
| RPF2 | 255.094655 | 0.51744196 | 0.00529015 | 0.06313013 |
| DDX60L | 624.713464 | 0.73952208 | 0.00530534 | 0.06314006 |
| EMC6 | 125.510461 | -0.618083 | 0.00530416 | 0.06314006 |
| IBA57 | 476.805809 | -0.4431109 | 0.00530261 | 0.06314006 |
| LIMK1 | 450.997546 | -0.5423185 | 0.00529542 | 0.06314006 |
| FAIM2 | 32.5764038 | -1.3142549 | 0.00533202 | 0.06341459 |
| CENPP | 165.767334 | -0.7093026 | 0.00534064 | 0.06347235 |
| FIGNL2 | 8.10312593 | 1.2754611 | 0.00535131 | 0.06347235 |
| NCAPG2 | 295.068998 | -0.6552961 | 0.00535047 | 0.06347235 |
| SKIV2L2 | 1074.88126 | 0.26100023 | 0.00534582 | 0.06347235 |
| C11orf80 | 96.2688336 | -0.6794979 | 0.0053812 | 0.06374096 |
| SPIRE2 | 125.040861 | -1.0564163 | 0.00537975 | 0.06374096 |
| CPSF4 | 229.085268 | 0.40422699 | 0.00539355 | 0.06379865 |
| ECM2 | 1864.13354 | 0.74269672 | 0.00539248 | 0.06379865 |
| XRCC1 | 393.791622 | -0.273606 | 0.00539695 | 0.06379865 |
| QRFPR | 22.275286 | 1.42974672 | 0.00540471 | 0.06380462 |
| TGM5 | 2.00783131 | 1.23589107 | 0.00540124 | 0.06380462 |
| PTPRZ1 | 17.6295957 | -1.4298618 | 0.00541089 | 0.06383466 |
| C12orf29 | 139.927624 | 0.36876324 | 0.0054412 | 0.06413304 |
| DOCK7 | 931.525089 | 0.56130915 | 0.00544347 | 0.06413304 |
| EEF1A1 | 72424.0787 | 0.56721496 | 0.00545002 | 0.06416728 |
| POC1A | 35.5166157 | -0.8009907 | 0.00545612 | 0.06419607 |
| PPP6R3 | 2402.74013 | -0.2865113 | 0.00547731 | 0.06440233 |
| EI24 | 951.135265 | -0.3041846 | 0.00549853 | 0.06456549 |

| | | | | |
|---|---|---|---|---|
| LOC1019271( | 1.98396388 | 1.39707562 | 0.00549725 | 0.06456549 |
| FBXL4 | 509.425143 | 0.54057298 | 0.00551808 | 0.06475191 |
| CD2 | 18.7311302 | 1.19028981 | 0.00553278 | 0.06479462 |
| SMIM8 | 128.035042 | 0.59084476 | 0.00553127 | 0.06479462 |
| ZCCHC14 | 1000.10242 | -0.5339689 | 0.00552996 | 0.06479462 |
| SV2B | 25.4268329 | -1.369538 | 0.00553723 | 0.06480369 |
| GALR1 | 8.20608744 | -1.3968436 | 0.00555028 | 0.06491314 |
| SCAMP5 | 298.258001 | -0.9640464 | 0.00556222 | 0.06500956 |
| ZNF503 | 863.818222 | -0.924981 | 0.00557032 | 0.06506103 |
| PPIF | 540.165842 | -0.7968083 | 0.00558013 | 0.06513233 |
| CHST7 | 71.4249869 | 1.05372209 | 0.00559086 | 0.06521428 |
| ZC3H18 | 553.588682 | -0.4308902 | 0.00560653 | 0.06535378 |
| COX6B2 | 5.2221668 | -1.3646098 | 0.00561461 | 0.06540462 |
| CDK15 | 7.17600588 | 1.16412342 | 0.00563702 | 0.06557882 |
| PSD | 24.7597973 | -1.0820648 | 0.00563601 | 0.06557882 |
| CHST11 | 599.081514 | 0.76369272 | 0.0056492 | 0.06563372 |
| ZEB2-AS1 | 1.32368021 | 1.36184111 | 0.00564915 | 0.06563372 |
| DDX39A | 366.462655 | -0.5387024 | 0.00569517 | 0.06612407 |
| CCNB3 | 9.62144859 | -1.0730091 | 0.00569923 | 0.06612759 |
| CFP | 13.6705981 | 0.96976251 | 0.00571028 | 0.06621211 |
| C5orf49 | 3.05683968 | 1.35757893 | 0.0057306 | 0.06634594 |
| GUSBP4 | 44.0534114 | 0.73322231 | 0.00573313 | 0.06634594 |
| PMEL | 29.7234524 | -0.9855012 | 0.00572862 | 0.06634594 |
| GAPT | 70.6416616 | 1.11714321 | 0.00573885 | 0.06636844 |
| LOC1001305. | 10.0375103 | -1.4198576 | 0.00574418 | 0.06638637 |
| ARMC7 | 204.768554 | -0.7404941 | 0.00576153 | 0.06643255 |
| HIVEP2 | 1004.77078 | 0.53452214 | 0.00576231 | 0.06643255 |
| LINC00260 | 73.8223309 | -0.7408331 | 0.00576328 | 0.06643255 |
| SEC31B | 405.172773 | 0.56560755 | 0.00575628 | 0.06643255 |
| IL2RG | 27.4951858 | 1.07213033 | 0.00577179 | 0.06648711 |
| LPCAT2 | 462.831246 | 0.99039592 | 0.00577732 | 0.06650728 |
| ITIH2 | 1403.05965 | 1.29701162 | 0.00580339 | 0.06672002 |
| SNTB2 | 1539.15493 | 0.64137246 | 0.0058015 | 0.06672002 |
| MAPK7 | 342.564725 | -0.4227899 | 0.00580793 | 0.06672154 |
| RPS14 | 5360.01173 | 0.46829599 | 0.00581111 | 0.06672154 |
| PKIB | 55.5752694 | 1.10964947 | 0.00582218 | 0.06680507 |
| C17orf89 | 158.521387 | -0.6377718 | 0.00584776 | 0.06686476 |
| DDIT4 | 2655.66891 | 1.02344923 | 0.00583503 | 0.06686476 |
| ELAC2 | 872.003181 | -0.3508181 | 0.00585779 | 0.06686476 |
| LOC1019272. | 7.77615769 | -1.4131086 | 0.0058492 | 0.06686476 |
| NPPA-AS1 | 5.37771404 | 1.09609348 | 0.00585468 | 0.06686476 |
| SAMD9L | 1498.68799 | 0.45590142 | 0.00583225 | 0.06686476 |
| ZNF559 | 421.651756 | 0.50447723 | 0.00585529 | 0.06686476 |
| ZNF57 | 111.988631 | -1.0557548 | 0.00585172 | 0.06686476 |
| HSPD1 | 3053.22197 | -0.6076484 | 0.00586335 | 0.06688475 |
| LOC1019287( | 5.42803061 | 1.3819573 | 0.00587154 | 0.06693478 |
| LOC1002889 | 30.2617573 | 1.0384787 | 0.00587575 | 0.06693946 |
| RAMP1 | 31.9463908 | -1.300571 | 0.005892 | 0.06708109 |
| ZNF674 | 92.8306411 | 0.51104139 | 0.0059041 | 0.06717538 |
| CHERP | 599.004873 | -0.4842045 | 0.00591301 | 0.06718987 |

| | | | | |
|---|---|---|---|---|
| ZBTB46 | 547.545263 | -0.7844902 | 0.00590949 | 0.06718987 |
| S1PR4 | 4.61156697 | 1.19628042 | 0.00591847 | 0.06720841 |
| RAB11B | 908.885037 | -0.5295726 | 0.00592844 | 0.06727826 |
| PLA2G3 | 28.4466703 | -1.395845 | 0.00596003 | 0.06759313 |
| UBN1 | 878.561784 | -0.246809 | 0.00598312 | 0.06781129 |
| COL6A4P2 | 18.3523729 | 1.31929381 | 0.00599467 | 0.0678202 |
| CSTB | 924.797617 | -0.7772236 | 0.00599826 | 0.0678202 |
| MARCH8 | 626.506098 | 0.46529177 | 0.00599206 | 0.0678202 |
| STRIP2 | 335.950393 | 0.95759266 | 0.00599933 | 0.0678202 |
| P2RX5 | 20.9619462 | -1.3735595 | 0.00601053 | 0.06786656 |
| UNC13A | 340.059048 | -1.3450177 | 0.00601115 | 0.06786656 |
| KCTD15 | 473.094064 | -0.705585 | 0.00601692 | 0.06788818 |
| ZNF331 | 580.053114 | 0.71552566 | 0.00602091 | 0.06788961 |
| NFE2L1 | 15083.6028 | -0.4731025 | 0.00602477 | 0.06788966 |
| PHLPP2 | 1241.89966 | -0.6598804 | 0.00602971 | 0.06790175 |
| SLA2 | 9.25717512 | 0.95470225 | 0.00603839 | 0.06795603 |
| MRPS24 | 22.5364258 | -0.7988738 | 0.00605747 | 0.06808361 |
| SLC25A22 | 268.850336 | -0.5022879 | 0.00605451 | 0.06808361 |
| CIAPIN1 | 404.805335 | -0.5505523 | 0.00607453 | 0.06823183 |
| ETV4 | 51.9704662 | -1.2703831 | 0.00610628 | 0.06853423 |
| FAM200A | 109.603044 | 0.52162858 | 0.00610925 | 0.06853423 |
| ICK | 618.203465 | 0.68480714 | 0.00611731 | 0.06858088 |
| NABP1 | 272.560044 | 0.89848207 | 0.00614878 | 0.06884601 |
| PLK4 | 75.1299168 | -0.8624847 | 0.00614604 | 0.06884601 |
| ARPC5L | 169.102961 | -0.3947112 | 0.00615944 | 0.0688587 |
| EAF2 | 47.8869908 | 0.67910202 | 0.00616557 | 0.0688587 |
| ELOVL2 | 191.454218 | -1.2377104 | 0.00615623 | 0.0688587 |
| NCK2 | 1153.49149 | -0.5642976 | 0.00616232 | 0.0688587 |
| SUPT3H | 96.6941348 | 0.78641569 | 0.00617012 | 0.06886578 |
| LOC1019271 | 2.3389472 | 1.40700437 | 0.00617811 | 0.06891122 |
| DHRS2 | 82.125077 | -1.3840876 | 0.00618497 | 0.06894394 |
| EPS8L2 | 690.567974 | -0.7854088 | 0.00618944 | 0.06895009 |
| KIAA1671 | 4588.91117 | -0.6454011 | 0.0062001 | 0.06899085 |
| MYOZ3 | 141.600014 | 1.23428279 | 0.00620353 | 0.06899085 |
| NAT8L | 127.393646 | -0.8838707 | 0.00620486 | 0.06899085 |
| MRO | 359.048622 | -1.0866831 | 0.00621306 | 0.06899473 |
| TMEM255A | 33.1432279 | -1.3686403 | 0.00621099 | 0.06899473 |
| EEFSEC | 179.324462 | -0.4754959 | 0.00623337 | 0.06917666 |
| SCP2 | 1068.13341 | 0.52020486 | 0.00624602 | 0.0692733 |
| HIST2H2BF | 326.124561 | -0.6810791 | 0.0062575 | 0.06935693 |
| CARF | 420.413737 | 0.41693356 | 0.00627469 | 0.06945986 |
| PDIK1L | 132.591691 | 0.47772894 | 0.00627388 | 0.06945986 |
| TRNT1 | 286.624074 | -0.4480701 | 0.0062795 | 0.06946949 |
| CD96 | 20.8541379 | 1.04770698 | 0.00628814 | 0.06947762 |
| SYNGR2 | 740.582145 | -0.6948021 | 0.00628431 | 0.06947762 |
| FITM2 | 108.07815 | -0.515004 | 0.0062988 | 0.06955168 |
| PDK4 | 1236.06121 | 1.16445164 | 0.00631684 | 0.06970715 |
| CTDSP1 | 2173.20378 | -0.2455021 | 0.00632615 | 0.06976614 |
| CAMKK2 | 678.591547 | -0.3665919 | 0.00633173 | 0.0697839 |
| AIG1 | 437.373804 | 0.57260121 | 0.00635671 | 0.07001533 |

| | | | | |
|---|---|---|---|---|
| CXorf56 | 266.651562 | 0.36137981 | 0.00636077 | 0.07001625 |
| CDKN2A | 44.3753833 | -1.30695 | 0.00637062 | 0.07008075 |
| MIR5572 | 1.53033586 | -1.3356566 | 0.00637795 | 0.07011754 |
| GLI2 | 776.124295 | -0.5731502 | 0.00639271 | 0.07023599 |
| LOC1005065 | 30.7798968 | 1.05395867 | 0.0063982 | 0.0702524 |
| SFXN1 | 579.156389 | -0.5499733 | 0.00641865 | 0.070433 |
| AFMID | 226.139881 | -0.6583809 | 0.00644911 | 0.0705111 |
| C1GALT1 | 439.416701 | 0.67623731 | 0.00644278 | 0.0705111 |
| CRYBB3 | 15.3762176 | -1.1724154 | 0.00643677 | 0.0705111 |
| FIRRE | 69.2577526 | -1.3410921 | 0.00644714 | 0.0705111 |
| QKI | 4881.01974 | 0.37435863 | 0.00644982 | 0.0705111 |
| WHAMMP3 | 129.087267 | 0.55234857 | 0.00643798 | 0.0705111 |
| CHCHD6 | 131.189074 | 0.92335625 | 0.0064684 | 0.0706008 |
| FBLN1 | 2178.81423 | 1.37307973 | 0.00647616 | 0.0706008 |
| GLMN | 75.7645119 | 0.74005732 | 0.00647167 | 0.0706008 |
| GPI | 4221.6021 | -0.6210638 | 0.00646253 | 0.0706008 |
| TSC22D3 | 5050.89579 | 0.91327237 | 0.0064781 | 0.0706008 |
| LAMC3 | 98.8062433 | -1.233243 | 0.00648256 | 0.07060567 |
| MRRF | 306.875979 | -0.4492405 | 0.00650342 | 0.07078531 |
| PRRT3 | 96.9781263 | -0.8283575 | 0.0065071 | 0.07078531 |
| TBC1D8 | 2814.24225 | -0.8683395 | 0.00653515 | 0.0710465 |
| DNAH12 | 46.9387259 | 1.13457881 | 0.00658559 | 0.07155063 |
| LOC1019272 | 37.7708471 | 0.59421365 | 0.00660707 | 0.07173975 |
| CASS4 | 94.4592572 | 1.08591875 | 0.00661186 | 0.07174748 |
| FXYD1 | 326.7294 | 0.89654685 | 0.00664018 | 0.07187925 |
| MSI2 | 1515.47305 | -0.5149068 | 0.00664444 | 0.07187925 |
| NCAPD3 | 495.490632 | -0.4398607 | 0.00663184 | 0.07187925 |
| PDE2A | 84.5137286 | 1.10285979 | 0.00663639 | 0.07187925 |
| TWIST2 | 85.3157495 | -1.0521586 | 0.00664272 | 0.07187925 |
| RNU6ATAC | 10.157866 | 1.23520238 | 0.00665588 | 0.07195873 |
| HORMAD1 | 2.36202051 | 1.3686604 | 0.0066713 | 0.07204093 |
| TPRXL | 8.08744902 | -1.1928687 | 0.00667167 | 0.07204093 |
| GPR19 | 3.33951177 | -1.3383385 | 0.00669881 | 0.07228963 |
| RLF | 546.507852 | 0.56751135 | 0.00671359 | 0.07240472 |
| NALCN-AS1 | 2.63608729 | 1.35996613 | 0.00672471 | 0.07248019 |
| FAM131A | 201.700396 | 0.8123818 | 0.00675256 | 0.07250355 |
| FAM134A | 1354.54853 | -0.368192 | 0.0067454 | 0.07250355 |
| FBLL1 | 19.2394899 | -1.23484 | 0.0067552 | 0.07250355 |
| MYOC | 1.72997171 | 1.39068877 | 0.00674622 | 0.07250355 |
| TMC4 | 95.3806337 | 1.13206521 | 0.00673428 | 0.07250355 |
| TRABD2A | 15.4216812 | -1.0676104 | 0.00674948 | 0.07250355 |
| TRIM59 | 89.3428754 | -1.0335043 | 0.00675573 | 0.07250355 |
| RIMS2 | 6.49587886 | -1.38569 | 0.00677058 | 0.07261864 |
| BAZ2A | 2611.2002 | -0.44271 | 0.00678616 | 0.07272746 |
| TNFSF13B | 81.1130181 | 0.76997422 | 0.006789 | 0.07272746 |
| CSNK2A3 | 11.4656862 | -1.1057021 | 0.0068011 | 0.0728128 |
| CHST6 | 6.4917891 | -1.1897552 | 0.00682713 | 0.07300254 |
| MYBPC2 | 15.7780267 | -1.3088914 | 0.00682359 | 0.07300254 |
| ANGPTL1 | 10.5167893 | -1.3827025 | 0.00685906 | 0.07321595 |
| CLN6 | 380.044317 | -0.5672359 | 0.00687059 | 0.07321595 |

| | | | | |
|---|---|---|---|---|
| FAM73A | 525.401744 | 0.4784359 | 0.00686531 | 0.07321595 |
| SLC2A13 | 185.27988 | 0.79147844 | 0.00685182 | 0.07321595 |
| STMN2 | 3.10385714 | -1.3896621 | 0.00686446 | 0.07321595 |
| SYMPK | 1154.12467 | -0.3510298 | 0.00687206 | 0.07321595 |
| CAP1 | 2228.07733 | 0.43871854 | 0.006885 | 0.07326509 |
| SNORA47 | 144.992718 | -0.8038545 | 0.0068835 | 0.07326509 |
| CD22 | 38.0405981 | -1.2608932 | 0.00689604 | 0.07330689 |
| FBXL19 | 342.696043 | -0.40624 | 0.00689727 | 0.07330689 |
| N4BP3 | 192.540667 | -0.9972669 | 0.00690974 | 0.07339512 |
| DDAH1 | 658.306134 | 0.89181048 | 0.00692171 | 0.07347788 |
| TMEFF2 | 3.06989736 | -1.3696019 | 0.00692639 | 0.07348328 |
| TRPV6 | 2.99630096 | -1.2318179 | 0.00693234 | 0.07350207 |
| RBM4B | 237.964997 | -0.314522 | 0.00694004 | 0.07353934 |
| LOC1001295 | 15.6084899 | 0.85657385 | 0.0069575 | 0.07367997 |
| MTR | 2279.06408 | -0.4132623 | 0.00697119 | 0.07373612 |
| PHC2 | 844.763143 | 0.67854146 | 0.00697053 | 0.07373612 |
| WFIKKN1 | 3.84945437 | -1.2752951 | 0.00697632 | 0.07374613 |
| C10orf85 | 2.02669293 | 1.37390366 | 0.00699328 | 0.07374813 |
| HNMT | 721.083848 | 0.69991166 | 0.00698714 | 0.07374813 |
| MYO3A | 121.977745 | -1.3066181 | 0.00698843 | 0.07374813 |
| SLC24A4 | 21.6598742 | 1.0941425 | 0.00699238 | 0.07374813 |
| YY1AP1 | 935.653986 | -0.3110162 | 0.00702491 | 0.0740373 |
| WFDC10B | 2.16401842 | 1.38153143 | 0.00704695 | 0.07422509 |
| RGS2 | 364.487017 | 1.11604499 | 0.00705636 | 0.0742797 |
| PSME3 | 1028.2267 | -0.3873644 | 0.00708571 | 0.074544 |
| ANAPC16 | 801.529816 | 0.50667458 | 0.00712887 | 0.0745605 |
| APOD | 2561.11528 | 1.34493362 | 0.00709186 | 0.0745605 |
| DKFZP586I14 | 243.549029 | 0.63993747 | 0.00712376 | 0.0745605 |
| FBXO43 | 8.2409028 | -1.1077351 | 0.00712334 | 0.0745605 |
| GSK3B | 1460.48401 | -0.2985407 | 0.00712264 | 0.0745605 |
| HIST2H2AC | 576.587367 | -0.4729899 | 0.00712197 | 0.0745605 |
| NEK11 | 116.819904 | 0.84870477 | 0.00712707 | 0.0745605 |
| SEC61G | 198.37667 | 0.61566205 | 0.00711761 | 0.0745605 |
| SETD1B | 915.850324 | -0.3953702 | 0.00711644 | 0.0745605 |
| XRCC2 | 62.2740623 | -0.9069593 | 0.00712966 | 0.0745605 |
| CXCR3 | 2.4690869 | 1.25358641 | 0.00713403 | 0.07456189 |
| GTF2IRD1 | 586.661702 | -0.5949058 | 0.00714839 | 0.07466752 |
| SWSAP1 | 30.9462481 | 0.65841211 | 0.007172 | 0.07486972 |
| MAL | 4.07708762 | -1.3733071 | 0.00718375 | 0.07490347 |
| SEPP1 | 3429.86545 | 0.873289 | 0.00718301 | 0.07490347 |
| GOLM1 | 1771.15419 | -0.5963552 | 0.00719107 | 0.07491947 |
| KIN | 266.199672 | 0.34762612 | 0.0072 | 0.07491947 |
| NCKAP5 | 133.342158 | 0.82041712 | 0.00719956 | 0.07491947 |
| UGT8 | 7.34085992 | -1.3588731 | 0.00720232 | 0.07491947 |
| YTHDF1 | 938.349134 | -0.5072065 | 0.00721737 | 0.07503163 |
| FRAT1 | 111.345698 | 0.892625 | 0.00722329 | 0.07504885 |
| SNAPC4 | 275.142855 | -0.4143946 | 0.00722815 | 0.07505501 |
| DPYD | 658.151311 | 0.80614371 | 0.00725658 | 0.07530575 |
| HLA-DQA1 | 362.607791 | 1.24692679 | 0.00731396 | 0.07585644 |
| SPN | 89.7027575 | 1.06549501 | 0.00732131 | 0.07588795 |

| | | | | |
|---|---|---|---|---|
| ERAL1 | 341.126496 | -0.3259454 | 0.0073296 | 0.07591108 |
| TNFRSF1B | 692.467589 | 0.75399358 | 0.00733217 | 0.07591108 |
| USP43 | 50.0868464 | -1.0280823 | 0.00733761 | 0.07592265 |
| PCNT | 1259.45603 | -0.5157463 | 0.00735042 | 0.07601048 |
| PTMA | 4515.25657 | -0.4510202 | 0.00736443 | 0.0760213 |
| ST6GALNAC4 | 356.019739 | -0.7474887 | 0.00736211 | 0.0760213 |
| TRIM38 | 797.731274 | 0.36543132 | 0.00736086 | 0.0760213 |
| DAPL1 | 44.5547071 | -1.375088 | 0.00738191 | 0.07602333 |
| ITGAX | 416.800742 | 0.95708409 | 0.00738057 | 0.07602333 |
| LRRC66 | 13.5859959 | 0.99498072 | 0.00737088 | 0.07602333 |
| RTCB | 556.417645 | 0.46526524 | 0.00738038 | 0.07602333 |
| COX8A | 1046.55534 | -0.4613345 | 0.00738814 | 0.07604294 |
| ZC3HAV1L | 242.16514 | -0.533992 | 0.00741562 | 0.07628116 |
| THAP3 | 88.9547193 | 0.49933557 | 0.00742424 | 0.07632517 |
| HRH2 | 26.2884063 | 1.00653559 | 0.00742941 | 0.07633368 |
| UBAP2 | 800.526155 | -0.5093192 | 0.0074365 | 0.07636196 |
| PPP5D1 | 8.36028369 | 1.06002587 | 0.00745969 | 0.07655536 |
| PHKB | 1581.52667 | -0.2486273 | 0.00747928 | 0.07671165 |
| SNORA22 | 91.4601122 | -0.9014758 | 0.00749196 | 0.076797 |
| AGAP1 | 981.878979 | -0.6289205 | 0.00750487 | 0.07683119 |
| IGF1 | 656.19493 | 1.21762476 | 0.0075084 | 0.07683119 |
| SLC50A1 | 279.539905 | -0.4904021 | 0.00750341 | 0.07683119 |
| AP2B1 | 4906.69735 | -0.420947 | 0.00755095 | 0.07700654 |
| CLSPN | 99.2796124 | -0.9061021 | 0.00755181 | 0.07700654 |
| EXOC3L1 | 18.2225992 | -0.8517377 | 0.00754821 | 0.07700654 |
| LRCH1 | 540.727939 | 0.94386298 | 0.0075351 | 0.07700654 |
| SIAE | 625.224355 | -0.6973814 | 0.00753678 | 0.07700654 |
| VPS4A | 992.212736 | -0.3558222 | 0.00754801 | 0.07700654 |
| GSTM5 | 127.50856 | 1.30779805 | 0.00756275 | 0.07707341 |
| CD19 | 2.00194291 | 1.3664171 | 0.00758995 | 0.07726113 |
| SHC4 | 273.00258 | 1.29300012 | 0.00758736 | 0.07726113 |
| LRRC24 | 39.3991708 | -0.6898899 | 0.00762504 | 0.07743905 |
| NPRL3 | 445.380552 | -0.5168202 | 0.00762186 | 0.07743905 |
| PPAPDC1A | 13.0121451 | 1.33254159 | 0.00761966 | 0.07743905 |
| ST8SIA4 | 318.592252 | 0.6874842 | 0.00761927 | 0.07743905 |
| CEP120 | 1145.50328 | 0.63001476 | 0.00766255 | 0.07768542 |
| SFRP1 | 1246.21992 | 1.34289952 | 0.00765898 | 0.07768542 |
| ZNF552 | 101.359538 | -0.4345508 | 0.00766107 | 0.07768542 |
| CLEC4E | 58.3287264 | 1.24811922 | 0.00768062 | 0.07782384 |
| RPS23 | 6159.9106 | 0.48475512 | 0.00770842 | 0.07806056 |
| TMEM132A | 293.960091 | -1.0659121 | 0.00771661 | 0.0780985 |
| CADPS2 | 1125.09816 | -0.9046582 | 0.00772116 | 0.07809962 |
| NDUFB2-AS1 | 6.24295045 | -0.9780856 | 0.0077492 | 0.07833815 |
| LRRC8A | 898.879648 | -0.4335187 | 0.00776225 | 0.07838003 |
| ZIC3 | 3.57793925 | -1.3677678 | 0.00775803 | 0.07838003 |
| OSBP | 1345.09887 | -0.3364541 | 0.00778612 | 0.07855951 |
| SEC16A | 3061.50191 | -0.396876 | 0.00778896 | 0.07855951 |
| RNF152 | 133.022328 | 1.1191809 | 0.00779578 | 0.07858328 |
| HTRA3 | 85.7421346 | -1.2049246 | 0.00780089 | 0.07858968 |
| TSTA3 | 308.407137 | -0.6239679 | 0.00781715 | 0.0787085 |

| | | | | |
|---|---|---|---|---|
| SLC22A20 | 9.06765585 | -1.0814901 | 0.0078242 | 0.0787344 |
| C1QTNF2 | 31.2511423 | 1.14869369 | 0.00786995 | 0.07897312 |
| DPF1 | 3.62336971 | -1.2745987 | 0.00787037 | 0.07897312 |
| FGD1 | 328.426605 | -0.4468967 | 0.00786895 | 0.07897312 |
| LAMA5 | 6392.39045 | -1.04763 | 0.00786443 | 0.07897312 |
| MPHOSPH6 | 104.383524 | 0.61250931 | 0.0078613 | 0.07897312 |
| BLOC1S6 | 966.662705 | 0.38008975 | 0.0078809 | 0.07902983 |
| C1orf61 | 6.60112108 | -1.3399154 | 0.00788501 | 0.07902983 |
| SULT1E1 | 46.9064246 | -1.3655629 | 0.00789634 | 0.07909831 |
| EBF3 | 261.419946 | 1.20634404 | 0.00793303 | 0.07942065 |
| GOLGA8N | 104.48881 | 0.71880309 | 0.0079693 | 0.07965506 |
| KIRREL2 | 6.34369895 | -1.3571089 | 0.00796171 | 0.07965506 |
| SNAI3 | 19.0466897 | 1.20328117 | 0.00797003 | 0.07965506 |
| SLC39A4 | 51.2174747 | -0.9393497 | 0.00797902 | 0.07969963 |
| FOXD2 | 985.867828 | -0.7040362 | 0.00800123 | 0.07985256 |
| SDCCAG3 | 323.487109 | -0.3889491 | 0.00800341 | 0.07985256 |
| STIP1 | 1039.55486 | -0.4937859 | 0.0080081 | 0.07985405 |
| KIAA1107 | 95.0899433 | 0.80434909 | 0.00802105 | 0.07993779 |
| WLS | 1641.20846 | 0.66990805 | 0.00805629 | 0.08024352 |
| LOC283731 | 3.82649162 | -1.3377782 | 0.00806903 | 0.08032494 |
| ZNF579 | 110.928098 | -0.597411 | 0.00811577 | 0.08074458 |
| SLAMF1 | 3.01500642 | 1.31262687 | 0.00812561 | 0.08079672 |
| C1orf198 | 1007.91842 | 0.72718818 | 0.00814756 | 0.08096927 |
| KRTAP5-10 | 2.89670533 | -1.2504201 | 0.00815649 | 0.08097997 |
| TLX1NB | 1.30583485 | 1.25932515 | 0.00815785 | 0.08097997 |
| HIC1 | 593.362307 | -0.6864376 | 0.0081648 | 0.08098283 |
| TACO1 | 181.921377 | -0.4827885 | 0.00816734 | 0.08098283 |
| ADAM20 | 89.4721456 | 0.71543662 | 0.00818912 | 0.08106166 |
| GLIS2 | 1186.00038 | -0.6665973 | 0.0081813 | 0.08106166 |
| OXCT1-AS1 | 7.8274801 | 1.09102863 | 0.00818859 | 0.08106166 |
| PDIA2 | 14.9532241 | 1.1735459 | 0.00821559 | 0.08127794 |
| B4GALT6 | 188.864157 | 0.93701003 | 0.00823802 | 0.08137546 |
| FAM157B | 1.7965365 | 1.29925748 | 0.00823932 | 0.08137546 |
| MTMR3 | 1127.46056 | 0.34116455 | 0.0082353 | 0.08137546 |
| KPNA5 | 253.096579 | 0.61969571 | 0.00824592 | 0.08139492 |
| AQP6 | 2.53211799 | -1.3245364 | 0.00825707 | 0.08145914 |
| CENPK | 50.3279916 | -1.0301507 | 0.00826169 | 0.08145914 |
| PI16 | 91.0604743 | 1.34990953 | 0.0082931 | 0.08172301 |
| AFAP1 | 3235.3442 | -0.6465041 | 0.00831173 | 0.08186073 |
| PSMD2 | 2140.87467 | -0.2986027 | 0.00833909 | 0.08208426 |
| BCL11A | 13.1524702 | -1.2515067 | 0.00840874 | 0.0826784 |
| CTSS | 922.399261 | 0.73459492 | 0.00840885 | 0.0826784 |
| SPOCK1 | 20.4803096 | -1.2054319 | 0.00844409 | 0.08297855 |
| C5orf45 | 158.642865 | 0.48442137 | 0.00845365 | 0.08300129 |
| RAD54L | 25.2271469 | -0.874106 | 0.00845584 | 0.08300129 |
| ADAMTS8 | 4.23374591 | -1.2876615 | 0.00847028 | 0.0830315 |
| CD93 | 1669.44188 | 0.72377207 | 0.00847308 | 0.0830315 |
| MNDA | 187.513684 | 0.88440434 | 0.00847297 | 0.0830315 |
| AQP5 | 33.5413778 | -1.3515927 | 0.00852513 | 0.08344852 |
| TLR5 | 129.703653 | 0.91485303 | 0.00852235 | 0.08344852 |

| | | | | |
|---|---|---|---|---|
| ECT2 | 283.98822 | -0.781238 | 0.00854807 | 0.08350859 |
| NLRC3 | 28.8700799 | 0.77852161 | 0.00854088 | 0.08350859 |
| SEPHS2 | 442.84727 | -0.6046046 | 0.00855025 | 0.08350859 |
| STAG3L2 | 393.629562 | -0.4820947 | 0.00854159 | 0.08350859 |
| PFKFB3 | 1095.32992 | 0.68190771 | 0.00857021 | 0.08365706 |
| COQ4 | 349.273436 | -0.3510392 | 0.00858218 | 0.08372742 |
| HIST1H3D | 205.543917 | -0.5778635 | 0.00859022 | 0.08374512 |
| SLC6A16 | 23.56193 | -1.0881179 | 0.00859352 | 0.08374512 |
| MOB3B | 191.764371 | 1.14393026 | 0.00860956 | 0.08382351 |
| TMEM44 | 136.046742 | -0.5835957 | 0.00861109 | 0.08382351 |
| UNG | 458.852563 | -0.4703865 | 0.00862775 | 0.08393922 |
| EIF4H | 2542.60041 | 0.26345735 | 0.00864249 | 0.08398972 |
| KIF21B | 170.101855 | 0.81043399 | 0.00863896 | 0.08398972 |
| GOPC | 775.073207 | 0.45935445 | 0.00865653 | 0.0840797 |
| CHRNB2 | 13.6945406 | -1.1283327 | 0.00868735 | 0.08422573 |
| MFSD6L | 1.5615883 | 1.29666276 | 0.00867949 | 0.08422573 |
| RACGAP1 | 298.266603 | -0.7297039 | 0.00868439 | 0.08422573 |
| TOP1 | 1834.46056 | -0.4056994 | 0.0086952 | 0.08422573 |
| USP32P1 | 458.292133 | -1.2897786 | 0.0086955 | 0.08422573 |
| FAM129B | 2373.61031 | -0.7306909 | 0.00870377 | 0.08424261 |
| METTL10 | 261.815124 | 0.49096096 | 0.00871293 | 0.08424261 |
| SLC7A5P2 | 311.113632 | -0.7085969 | 0.0087164 | 0.08424261 |
| ZIC4 | 293.497437 | -0.9547033 | 0.00871326 | 0.08424261 |
| GLYR1 | 1388.05145 | -0.2761638 | 0.00872684 | 0.08429716 |
| PCBP1 | 1854.6541 | -0.5438329 | 0.00873388 | 0.08431883 |
| PTH2R | 16.4102415 | -1.3110094 | 0.00875121 | 0.08443982 |
| MTF2 | 323.418175 | 0.49828425 | 0.00875934 | 0.08447192 |
| ADAMTS17 | 731.755755 | -1.0378369 | 0.00878086 | 0.08463309 |
| TMED7 | 1697.24227 | 0.40263344 | 0.00881687 | 0.08493357 |
| CCNB1 | 136.261762 | -0.8451117 | 0.00882867 | 0.08495422 |
| NAALADL2 | 138.503997 | 0.68015692 | 0.00882858 | 0.08495422 |
| FRA10AC1 | 187.08009 | 0.6010873 | 0.00887452 | 0.08526907 |
| PHACTR3 | 15.7575775 | -1.3109532 | 0.00887593 | 0.08526907 |
| ZC3H6 | 787.13698 | 0.52677322 | 0.00887457 | 0.08526907 |
| MSH4 | 24.374397 | 1.24713325 | 0.00889041 | 0.08536157 |
| RDM1 | 3.2391828 | -1.2834219 | 0.00889644 | 0.08537285 |
| DLGAP3 | 12.841439 | -0.8849434 | 0.00891796 | 0.08553264 |
| PVRL1 | 381.637036 | -0.7861457 | 0.00892347 | 0.0855389 |
| SLFN13 | 335.350902 | -0.8899567 | 0.00895245 | 0.08572321 |
| SORL1 | 3035.71862 | 1.06952883 | 0.00894893 | 0.08572321 |
| BSDC1 | 833.073056 | 0.42548249 | 0.00898319 | 0.08597082 |
| MCM7 | 750.278983 | -0.5111325 | 0.00899585 | 0.08599838 |
| SNX10 | 153.616538 | 0.64461621 | 0.00899103 | 0.08599838 |
| MRPL54 | 154.269287 | 0.49527471 | 0.00900599 | 0.08604853 |
| L3MBTL1 | 244.504355 | 0.54906386 | 0.00902569 | 0.08618995 |
| CTNND2 | 15.3003762 | -1.2736968 | 0.00903858 | 0.08621995 |
| MAOB | 26.8689368 | -1.2610371 | 0.00904109 | 0.08621995 |
| SCUBE3 | 733.938635 | -0.84299 | 0.00904844 | 0.08621995 |
| TAGLN3 | 2.63950269 | -1.2435348 | 0.00904577 | 0.08621995 |
| DOCK2 | 694.728173 | 0.78603255 | 0.00905638 | 0.08624894 |

| | | | | |
|---|---|---|---|---|
| TJP3 | 44.0041609 | -1.1412179 | 0.00906359 | 0.08627088 |
| ISY1 | 125.436635 | 0.42532774 | 0.00912104 | 0.08677075 |
| GPR126 | 276.694093 | 0.97527771 | 0.00912793 | 0.08678937 |
| FADS1 | 1182.50756 | -0.9346054 | 0.00917333 | 0.0871739 |
| CIDECP | 50.5251633 | -0.5634377 | 0.00919046 | 0.08724239 |
| PFDN2 | 174.667901 | -0.4907102 | 0.00919001 | 0.08724239 |
| HERC2P7 | 47.1901979 | -0.5599004 | 0.00920351 | 0.0872675 |
| MSTN | 17.8233495 | 0.93613515 | 0.00920799 | 0.0872675 |
| MYLK | 1397.31815 | 1.18158923 | 0.00920298 | 0.0872675 |
| MAP3K8 | 296.915721 | 0.69428878 | 0.00922595 | 0.0873907 |
| TRUB2 | 283.000817 | -0.4761209 | 0.00923707 | 0.08744894 |
| HNRNPLL | 545.387493 | 0.3874339 | 0.0092665 | 0.08763319 |
| MNT | 458.385302 | -0.4362841 | 0.0092657 | 0.08763319 |
| CALB1 | 55.3802302 | -1.3372605 | 0.00928143 | 0.08768806 |
| CXorf30 | 10.8770499 | -1.3350675 | 0.00928985 | 0.08768806 |
| MRAP2 | 126.592335 | 1.3355561 | 0.00928929 | 0.08768806 |
| SAP30BP | 909.955125 | -0.6214766 | 0.00929224 | 0.08768806 |
| GPNMB | 1155.58777 | 1.06298916 | 0.00930545 | 0.08772684 |
| MSS51 | 92.1673805 | 0.66182436 | 0.00930633 | 0.08772684 |
| SLC6A9 | 68.5587588 | -0.9949846 | 0.00933148 | 0.08791684 |
| KLHDC1 | 149.047834 | 0.7065104 | 0.00935108 | 0.08803905 |
| LRMP | 84.0325391 | 0.8398506 | 0.00935746 | 0.08803905 |
| ZBTB37 | 415.01171 | -0.3624504 | 0.00935947 | 0.08803905 |
| CATSPER2P1 | 25.023952 | -0.7792548 | 0.00942443 | 0.08855545 |
| SMIM14 | 642.774746 | 0.58541772 | 0.00942059 | 0.08855545 |
| CLPB | 433.961787 | -0.4142103 | 0.00945875 | 0.08863486 |
| FEZ2 | 674.230142 | 0.37286401 | 0.00946299 | 0.08863486 |
| IARS | 2597.69943 | -0.4533487 | 0.00944844 | 0.08863486 |
| PCDHB10 | 216.552312 | -0.9819181 | 0.00946312 | 0.08863486 |
| SCAND1 | 257.54916 | -0.4786299 | 0.00945834 | 0.08863486 |
| SEC22A | 250.394982 | 0.27047855 | 0.00945783 | 0.08863486 |
| KCTD2 | 819.709457 | -0.4183544 | 0.00947872 | 0.08868653 |
| TFEC | 243.715246 | 0.91099253 | 0.00947392 | 0.08868653 |
| HOXD13 | 11.2605109 | -1.2860945 | 0.00949335 | 0.08872902 |
| TRMT11 | 265.751446 | 0.51233345 | 0.009489 | 0.08872902 |
| EIF2B3 | 176.626493 | 0.59341514 | 0.00951491 | 0.08888328 |
| CRLF3 | 218.030425 | 0.50076702 | 0.00952796 | 0.08895793 |
| GADL1 | 5.35914178 | -1.3192235 | 0.00955342 | 0.08914836 |
| IGF2BP1 | 7.3617089 | -1.3316526 | 0.00957142 | 0.08922166 |
| TRPC6 | 35.7064511 | 1.0032315 | 0.00956891 | 0.08922166 |
| GALM | 183.624667 | 0.85428911 | 0.00959267 | 0.08934899 |
| GUK1 | 1166.13852 | -0.4041806 | 0.00959524 | 0.08934899 |
| CYP4Z1 | 88.5680969 | 0.98522166 | 0.00962941 | 0.08957234 |
| TSGA10 | 86.8851414 | 0.72635449 | 0.00962545 | 0.08957234 |
| LACC1 | 207.000162 | 0.61782274 | 0.00963983 | 0.08962189 |
| ULK2 | 679.691497 | 0.42920502 | 0.00964632 | 0.08963488 |
| NRCAM | 42.2055144 | -1.2105624 | 0.00965571 | 0.08967469 |
| RASA4B | 26.1088889 | 0.62958016 | 0.00969269 | 0.08992323 |
| TMEM175 | 389.06708 | -0.4553691 | 0.00968797 | 0.08992323 |
| ZNF709 | 157.704013 | 0.70949104 | 0.00969943 | 0.08993829 |

| | | | | |
|---|---|---|---|---|
| LINC00276 | 3.75064201 | 1.20784277 | 0.00972701 | 0.09014653 |
| DARS2 | 349.290839 | -0.6333252 | 0.00973936 | 0.09019509 |
| ZHX3 | 2578.78223 | 0.5797516 | 0.00974251 | 0.09019509 |
| GTF3C4 | 517.918237 | -0.3195609 | 0.00976933 | 0.09025341 |
| RPTOR | 1110.45962 | -0.3836327 | 0.009767 | 0.09025341 |
| SLC27A4 | 359.198924 | -0.4238999 | 0.0097629 | 0.09025341 |
| SRRD | 111.163421 | 0.42961768 | 0.00976278 | 0.09025341 |
| GPR174 | 4.62927119 | 1.10839929 | 0.00979358 | 0.09042996 |
| WDR83OS | 586.177275 | 0.41441455 | 0.00982524 | 0.09067466 |
| DLGAP1-AS1 | 79.1493969 | 0.64305101 | 0.00983864 | 0.09069063 |
| HOGA1 | 117.589582 | 0.94548182 | 0.00984243 | 0.09069063 |
| RAB8B | 1322.96586 | 0.6305523 | 0.00983366 | 0.09069063 |
| CASKIN2 | 703.627239 | -0.7805524 | 0.00985386 | 0.09074834 |
| CTD-2201I18 | 13.0738831 | 1.25956502 | 0.00986941 | 0.09084402 |
| C20orf197 | 16.2091515 | 1.014425 | 0.00987761 | 0.09087192 |
| NT5C2 | 1885.4198 | 0.58278399 | 0.00990685 | 0.09109327 |
| INSRR | 21.2773372 | -1.2275415 | 0.0099209 | 0.09113647 |
| SLC25A36 | 1329.03309 | -0.2130097 | 0.00992191 | 0.09113647 |
| GUSBP1 | 268.407154 | 0.44209335 | 0.009985 | 0.09166816 |
| ITGB1BP2 | 12.3987205 | 0.96962101 | 0.01000256 | 0.09168581 |
| NFAM1 | 131.597606 | 0.90117812 | 0.00999929 | 0.09168581 |
| WFIKKN2 | 33.596926 | -1.1810948 | 0.01000178 | 0.09168581 |
| CDK2 | 448.89343 | -0.5305259 | 0.01000805 | 0.09168834 |
| LOC283194 | 8.18574721 | 1.14573638 | 0.01004748 | 0.09192204 |
| SCYL1 | 1087.19291 | -0.3243741 | 0.01004924 | 0.09192204 |
| SLITRK6 | 107.481748 | -1.3187076 | 0.01004128 | 0.09192204 |
| RNF182 | 6.46914249 | -1.28002 | 0.01005986 | 0.09197139 |
| GABRB1 | 2.40867529 | -1.3185873 | 0.01007127 | 0.09202785 |
| NPHP3-ACAD | 110.391161 | 0.59948344 | 0.01009371 | 0.09210961 |
| RBPMS | 582.528367 | -0.6628947 | 0.01010028 | 0.09210961 |
| SGK494 | 177.807946 | -0.742496 | 0.01010116 | 0.09210961 |
| TRAPPC9 | 719.353097 | -0.3728201 | 0.01009847 | 0.09210961 |
| GRIK3 | 6.18211677 | -1.2867098 | 0.01013269 | 0.09230275 |
| MAP1S | 412.22877 | -0.4313726 | 0.01013284 | 0.09230275 |
| FAM227B | 42.0173594 | 0.55812735 | 0.01016208 | 0.09234712 |
| KLHDC3 | 930.972133 | 0.46675492 | 0.01016678 | 0.09234712 |
| SCAF1 | 897.905899 | -0.3778499 | 0.01014431 | 0.09234712 |
| SEPT9 | 1864.8579 | -0.6178233 | 0.01016643 | 0.09234712 |
| SFTPD | 4.55890954 | 1.30052897 | 0.0101552 | 0.09234712 |
| WASH2P | 228.707634 | 0.32184616 | 0.01016921 | 0.09234712 |
| CACTIN-AS1 | 3.269549 | 1.26311632 | 0.01017918 | 0.09238994 |
| MAT2A | 2125.07196 | -0.2968164 | 0.0101917 | 0.0924559 |
| DOCK10 | 633.678268 | 0.81421739 | 0.01020821 | 0.09251025 |
| EHD2 | 2104.9211 | -0.6436983 | 0.01020537 | 0.09251025 |
| CPLX2 | 41.6759835 | -1.2843813 | 0.01023052 | 0.09266467 |
| LOC1009965 | 11.0636019 | -1.2925667 | 0.01024017 | 0.09270435 |
| DKFZp779M( | 1.80227249 | 1.22945518 | 0.01029437 | 0.09295587 |
| GPR132 | 82.1197067 | 0.91900268 | 0.01029113 | 0.09295587 |
| INTS10 | 466.561986 | 0.44790115 | 0.01028452 | 0.09295587 |
| MIR497HG | 92.2121759 | 1.17496674 | 0.01028143 | 0.09295587 |

| | | | | |
|---|---|---|---|---|
| SH2D5 | 2.13265296 | -1.2656291 | 0.01029332 | 0.09295587 |
| DUSP2 | 37.1178142 | 1.1913886 | 0.01032811 | 0.09318031 |
| WWP2 | 971.062957 | 0.49516715 | 0.01032982 | 0.09318031 |
| ATP5G1 | 339.783801 | -0.4016904 | 0.01035167 | 0.09332952 |
| SMU1 | 1213.1633 | 0.26118731 | 0.01036404 | 0.09339315 |
| ATP5D | 483.65129 | -0.5771506 | 0.01037948 | 0.09346313 |
| PRRG3 | 11.2313071 | -1.3010998 | 0.01038243 | 0.09346313 |
| ZFP14 | 385.960028 | 0.48150248 | 0.01039122 | 0.09349439 |
| PKN3 | 138.442897 | -0.7911983 | 0.01040123 | 0.09353666 |
| AGBL3 | 22.839102 | 0.67526441 | 0.01041614 | 0.0935698 |
| BCL9 | 565.89901 | -0.56335 | 0.0104284 | 0.0935698 |
| COL8A1 | 4128.87281 | 1.2432255 | 0.01043422 | 0.0935698 |
| CTNNBIP1 | 286.493552 | 0.59969044 | 0.0104216 | 0.0935698 |
| FAM209B | 1.62886279 | 1.18017663 | 0.01043684 | 0.0935698 |
| LCP1 | 865.873621 | 0.88846622 | 0.01041262 | 0.0935698 |
| NUTF2 | 663.141085 | -0.4457137 | 0.01048337 | 0.09393912 |
| SNORA37 | 14.9144109 | 0.84058316 | 0.01050237 | 0.09406141 |
| OMG | 3.99499555 | -1.3152802 | 0.0105092 | 0.09407474 |
| STS | 494.077123 | 0.92121554 | 0.01051701 | 0.09409678 |
| PRPS2 | 292.904714 | -0.336753 | 0.01054059 | 0.0942598 |
| P2RY2 | 8.5396683 | 1.06877948 | 0.0105563 | 0.09435231 |
| CMKLR1 | 753.210278 | -1.1717778 | 0.01056978 | 0.09436855 |
| FIBIN | 2098.07476 | 1.2492112 | 0.01057958 | 0.09436855 |
| PPAP2B | 1602.65214 | 1.0070891 | 0.01056862 | 0.09436855 |
| SMOC2 | 1104.65737 | 1.1645175 | 0.01057532 | 0.09436855 |
| CCNC | 398.775093 | 0.50870935 | 0.01063712 | 0.09472702 |
| PBDC1 | 204.432539 | -0.4762585 | 0.0106355 | 0.09472702 |
| PTRH2 | 136.549145 | -0.5927044 | 0.01064131 | 0.09472702 |
| SRPX | 118.385247 | 1.31232844 | 0.01063272 | 0.09472702 |
| LOC1005060 | 11.6996876 | 1.04176154 | 0.01065594 | 0.09480929 |
| FGD3 | 113.520507 | 0.84712199 | 0.01066276 | 0.09482201 |
| ANKRD12 | 2012.35097 | 0.53194202 | 0.01070228 | 0.09483787 |
| FOXO4 | 163.011566 | 0.66908949 | 0.01069999 | 0.09483787 |
| ITGAM | 625.424429 | 0.90210399 | 0.01069544 | 0.09483787 |
| KLHL41 | 19.5811554 | 0.88142473 | 0.01068792 | 0.09483787 |
| LETM1 | 992.057375 | -0.4210587 | 0.01067469 | 0.09483787 |
| METTL1 | 90.4556564 | -0.5436944 | 0.01069129 | 0.09483787 |
| RCN3 | 344.239766 | 0.91686764 | 0.01069005 | 0.09483787 |
| ARPP21 | 2.48092624 | -1.3115684 | 0.01072889 | 0.09502572 |
| PPP6R1 | 983.591198 | -0.3698864 | 0.01076842 | 0.09532785 |
| ZNF420 | 191.940567 | 0.35822484 | 0.01079093 | 0.09547909 |
| C19orf24 | 168.987367 | -0.4160026 | 0.01081196 | 0.0956171 |
| ADAMTSL4-A | 90.6223746 | 0.76028893 | 0.01083475 | 0.09567432 |
| CCR5 | 60.1099779 | 0.99780015 | 0.01083251 | 0.09567432 |
| PITX1 | 44.8059028 | -1.2897383 | 0.01083356 | 0.09567432 |
| ANKAR | 221.579737 | 0.48696999 | 0.01087701 | 0.09598508 |
| C7orf76 | 2.73281527 | 1.17219638 | 0.01088086 | 0.09598508 |
| APOB | 143.79025 | 0.99439349 | 0.01089388 | 0.09605101 |
| KIF15 | 79.7250562 | -0.805695 | 0.01089925 | 0.09605101 |
| TUBB1 | 8.69275039 | 1.16033592 | 0.01091324 | 0.09612611 |

| | | | | |
|---|---|---|---|---|
| BSCL2 | 98.8387233 | -0.4663892 | 0.01092855 | 0.09621277 |
| NLRP1 | 349.563795 | 0.85735851 | 0.01094438 | 0.09630394 |
| HMGCS1 | 911.000882 | -0.7204714 | 0.01096331 | 0.09642232 |
| SLC26A6 | 751.588407 | -0.9098107 | 0.01097726 | 0.09649676 |
| DPF2 | 650.231858 | -0.2585779 | 0.01099916 | 0.09664097 |
| NUDT12 | 333.710983 | 0.65790589 | 0.01101175 | 0.09670331 |
| ZNF319 | 216.828932 | -0.3919418 | 0.0110513 | 0.09700217 |
| KCNQ2 | 1.480978 | -1.2563217 | 0.01106445 | 0.09706917 |
| C6orf226 | 34.3841844 | -0.7510572 | 0.01110301 | 0.09735892 |
| METTL3 | 372.741443 | 0.48863607 | 0.01111155 | 0.09738526 |
| MRPS11 | 216.124956 | -0.4585369 | 0.01111714 | 0.09738567 |
| RPL9 | 4924.86024 | 0.41211927 | 0.01112541 | 0.09740963 |
| HSPA5 | 7500.83159 | -0.7360764 | 0.01115369 | 0.09756013 |
| SYT12 | 13.337844 | -1.2829697 | 0.01115133 | 0.09756013 |
| CGNL1 | 2553.76464 | 1.02449688 | 0.01116843 | 0.09759199 |
| NEK10 | 8.66049861 | 0.96918089 | 0.01116798 | 0.09759199 |
| MED8 | 228.707734 | 0.54505333 | 0.0111752 | 0.09760264 |
| LOC642423 | 2.2050648 | -1.113341 | 0.01120546 | 0.09781836 |
| PCYOX1L | 165.946433 | -0.6661185 | 0.01122076 | 0.09785483 |
| PRPF3 | 910.366099 | -0.4682312 | 0.01121874 | 0.09785483 |
| GINS1 | 95.9678097 | -0.830902 | 0.01125066 | 0.09790827 |
| PPP1R14A | 10.7156895 | -1.0851736 | 0.01124798 | 0.09790827 |
| PTPRM | 2645.52257 | 0.60985929 | 0.01126029 | 0.09790827 |
| S100A10 | 3848.74361 | -0.5949333 | 0.01125241 | 0.09790827 |
| SLC7A5 | 379.476834 | -1.1028454 | 0.01125667 | 0.09790827 |
| ZRANB2 | 1238.77489 | 0.39719814 | 0.01124086 | 0.09790827 |
| DNAH9 | 33.5780757 | 1.01605752 | 0.01127773 | 0.09801146 |
| SLC29A2 | 16.2635598 | -1.2130864 | 0.01131126 | 0.09820585 |
| TMEM232 | 33.1005057 | 0.81704389 | 0.01130746 | 0.09820585 |
| ABI2 | 1589.28727 | -0.2600054 | 0.011365 | 0.09842849 |
| DDX42 | 2325.93893 | -0.2630001 | 0.0113579 | 0.09842849 |
| FAM182A | 4.24978078 | 1.28575251 | 0.01135063 | 0.09842849 |
| SETDB1 | 846.87377 | -0.3441052 | 0.01137048 | 0.09842849 |
| TP63 | 855.707961 | -1.2487262 | 0.0113567 | 0.09842849 |
| USP24 | 2333.569 | 0.44688153 | 0.01136918 | 0.09842849 |
| ALG14 | 67.0363986 | 0.61239694 | 0.01139107 | 0.09855823 |
| PURA | 1305.10431 | 0.4753986 | 0.0114063 | 0.09864152 |
| LRPPRC | 2367.36386 | -0.4401944 | 0.01142262 | 0.09868564 |
| SLC35F1 | 8.11396146 | -1.2337157 | 0.01141818 | 0.09868564 |
| ALMS1P | 4.22355277 | 1.07876514 | 0.01143995 | 0.09869065 |
| C16orf13 | 276.987338 | -0.4670433 | 0.01143106 | 0.09869065 |
| GRB7 | 15.2770871 | -1.0920194 | 0.01144565 | 0.09869065 |
| PRKG1 | 379.097124 | 1.01973672 | 0.0114456 | 0.09869065 |
| PARK2 | 87.986768 | 0.69044471 | 0.01146914 | 0.09884474 |
| SUGP1 | 339.47792 | -0.2726939 | 0.01147712 | 0.09886512 |
| KRTCAP2 | 643.855049 | -0.4516948 | 0.01150059 | 0.09901879 |
| EDN1 | 38.6798321 | 1.02343008 | 0.01151264 | 0.09902557 |
| SNORA10 | 8.4224426 | -1.0151493 | 0.01151161 | 0.09902557 |
| CLYBL | 87.9650955 | -0.5450727 | 0.01152859 | 0.09911436 |
| BRPF1 | 373.678976 | -0.3768595 | 0.01155155 | 0.09926323 |

| | | | | |
|---|---|---|---|---|
| TP53 | 759.585356 | -0.477851 | 0.01158129 | 0.09947017 |
| CTXN1 | 53.6242678 | -1.0128276 | 0.01159226 | 0.0995158 |
| LMBRD1 | 803.698176 | 0.62951337 | 0.01161732 | 0.0996378 |
| MANEA | 410.497381 | 0.65990363 | 0.0116178 | 0.0996378 |
| TSSC4 | 207.600728 | 0.41448553 | 0.01163209 | 0.09971172 |
| ANK3 | 543.83447 | 1.02748273 | 0.01167264 | 0.10001058 |
| HS3ST2 | 1.35087474 | -1.2867445 | 0.01169271 | 0.10003639 |
| LDB1 | 1146.34633 | -0.495794 | 0.01168734 | 0.10003639 |
| TP53INP2 | 980.326758 | -0.7036812 | 0.01168272 | 0.10003639 |
| C16orf86 | 30.0215993 | 1.05668027 | 0.01172378 | 0.10010743 |
| CRHR1 | 2.40582086 | 1.28750912 | 0.0117128 | 0.10010743 |
| DAAM1 | 407.477678 | 0.71983906 | 0.01172082 | 0.10010743 |
| SP4 | 268.044704 | 0.48340013 | 0.01172206 | 0.10010743 |
| TRIM7 | 24.2590231 | 1.01776631 | 0.01177229 | 0.10047283 |
| DMXL1 | 2253.31708 | 0.4510443 | 0.01179167 | 0.1005728 |
| MYBPC1 | 2.98903073 | 1.28358317 | 0.01179543 | 0.1005728 |
| SLED1 | 21.6166102 | 1.05589575 | 0.01181547 | 0.1006948 |
| CD3G | 4.97961134 | 1.1703156 | 0.01182555 | 0.10073196 |
| GYS1 | 1080.65506 | -0.478583 | 0.01185708 | 0.1008714 |
| PRNP | 1967.47208 | 0.49008355 | 0.01185913 | 0.1008714 |
| SLC29A4 | 41.2254333 | -1.1315046 | 0.01185871 | 0.1008714 |
| SCUBE2 | 48.0387352 | 1.08537123 | 0.01187641 | 0.10096956 |
| C2orf15 | 15.9640901 | -0.8641079 | 0.01190111 | 0.10108185 |
| ZNF695 | 10.3276014 | -1.1379469 | 0.01189966 | 0.10108185 |
| HLF | 3390.13925 | 0.71121899 | 0.01192658 | 0.10124929 |
| NOL11 | 556.46959 | -0.3417932 | 0.01194754 | 0.10137833 |
| LSM12 | 249.763551 | -0.4439958 | 0.01197356 | 0.10155011 |
| ITIH3 | 12.8339221 | 1.25339661 | 0.01198096 | 0.1015639 |
| PCDHB16 | 596.64816 | -1.0148205 | 0.01199704 | 0.10165119 |
| DNAJB4 | 483.055656 | 0.76837785 | 0.01202347 | 0.10174715 |
| MARCH1 | 346.371791 | 0.84089245 | 0.01202572 | 0.10174715 |
| TBC1D10C | 18.7998992 | 0.98745078 | 0.01202548 | 0.10174715 |
| POTEJ | 27.7853301 | -0.7765977 | 0.01204081 | 0.10182589 |
| MFGE8 | 727.972092 | 0.9082871 | 0.01208736 | 0.1021704 |
| RASSF7 | 328.126049 | -0.7837914 | 0.01209778 | 0.10220942 |
| C11orf44 | 2.81487725 | 1.2871382 | 0.01211221 | 0.10228214 |
| HCFC1 | 2397.99007 | -0.3282641 | 0.01215039 | 0.10245703 |
| ICA1L | 259.718095 | 0.72596262 | 0.01214458 | 0.10245703 |
| UBLCP1 | 419.857303 | 0.22117043 | 0.01213912 | 0.10245703 |
| ETV3 | 272.971867 | -0.3550517 | 0.0121669 | 0.1024992 |
| ZNF574 | 263.834691 | -0.3284222 | 0.01216705 | 0.1024992 |
| MIR181A1H( | 57.4126577 | -0.7753829 | 0.01219421 | 0.10267883 |
| COQ3 | 31.8957607 | 0.54423003 | 0.01220879 | 0.10273971 |
| SAMD3 | 6.12312816 | 1.12609853 | 0.01221312 | 0.10273971 |
| ASPG | 6.72131176 | 1.24148216 | 0.01225076 | 0.10297197 |
| DTX1 | 3.29223566 | -1.2478831 | 0.01225243 | 0.10297197 |
| SNHG10 | 65.8140844 | 0.51460863 | 0.01233001 | 0.103525 |
| ZYG11B | 1282.47491 | 0.49900043 | 0.01232704 | 0.103525 |
| LOC442028 | 35.3632189 | -1.2288942 | 0.01233605 | 0.10352631 |
| ADSS | 650.687267 | -0.3595628 | 0.01234731 | 0.1035714 |

| | | | | |
|---|---|---|---|---|
| HIST1H3I | 77.6256111 | -0.9198371 | 0.01236383 | 0.10366053 |
| CCDC124 | 301.113209 | -0.5010763 | 0.01239035 | 0.10383339 |
| GZMM | 1.97633887 | 1.22489555 | 0.01243837 | 0.10413654 |
| NOP14 | 613.04987 | -0.3684622 | 0.01243595 | 0.10413654 |
| FAM65A | 824.952635 | -0.3765384 | 0.01245505 | 0.10421816 |
| HIST1H4I | 26.5851307 | 0.93280888 | 0.01246887 | 0.10421816 |
| LOC1019270 | 3.82614715 | 1.16747416 | 0.01246103 | 0.10421816 |
| NFIA | 4419.28123 | 0.50548368 | 0.01247182 | 0.10421816 |
| NEIL3 | 17.8882922 | -0.903145 | 0.01251124 | 0.104498 |
| EIF4G1 | 5206.83667 | -0.3152885 | 0.01253735 | 0.10466631 |
| DHCR24 | 1192.26893 | -0.9644255 | 0.01257492 | 0.1049302 |
| TFAP4 | 86.2441319 | -0.750607 | 0.01260075 | 0.10509587 |
| PLSCR4 | 811.872572 | 0.71338444 | 0.01260844 | 0.1051102 |
| DLGAP4 | 1264.97398 | -0.6463967 | 0.01262056 | 0.10513613 |
| GRM7 | 36.0323409 | -1.2777433 | 0.01265067 | 0.10513613 |
| LRRC8C | 1232.74403 | 0.62961715 | 0.01264673 | 0.10513613 |
| MAP3K11 | 638.443469 | -0.4402532 | 0.01263805 | 0.10513613 |
| PABPC3 | 23.6396158 | -0.9476277 | 0.0126454 | 0.10513613 |
| SLC25A33 | 44.6416468 | 0.67550271 | 0.01265339 | 0.10513613 |
| WDSUB1 | 176.775913 | -0.4151742 | 0.01265113 | 0.10513613 |
| LRRC69 | 9.66141907 | 0.84703124 | 0.01266579 | 0.10518738 |
| TSPAN2 | 22.2643151 | -1.2022264 | 0.01267152 | 0.10518738 |
| SERTM1 | 144.6982 | 1.2793928 | 0.01268587 | 0.10525678 |
| ARAP3 | 170.45605 | 0.79681192 | 0.01279063 | 0.10583365 |
| CNPY4 | 286.323915 | 0.54291907 | 0.01277342 | 0.10583365 |
| DNPEP | 408.096581 | -0.3690933 | 0.01278053 | 0.10583365 |
| FGD2 | 320.285714 | 0.8365301 | 0.01277909 | 0.10583365 |
| GTF2B | 182.754677 | 0.50492289 | 0.01279149 | 0.10583365 |
| SEZ6 | 3.03019801 | -1.2206748 | 0.01276813 | 0.10583365 |
| ELF4 | 587.184075 | -0.5203067 | 0.01279999 | 0.10585415 |
| FASTKD1 | 281.999901 | -0.5247002 | 0.01281169 | 0.10590117 |
| COL7A1 | 365.299018 | -0.8526574 | 0.01282375 | 0.10595107 |
| IFI44 | 262.317596 | 0.65124767 | 0.01284766 | 0.10609872 |
| ZNF277 | 284.021546 | 0.35304326 | 0.01285398 | 0.10610111 |
| PHLDB1 | 2459.67734 | -0.4398601 | 0.01287025 | 0.10618556 |
| CPNE5 | 66.3061028 | 1.12831964 | 0.01288321 | 0.10624267 |
| FADD | 210.472432 | -0.3992807 | 0.01289751 | 0.10631074 |
| CFL1 | 3643.69941 | -0.3459104 | 0.01291184 | 0.10637904 |
| IBTK | 1418.76264 | 0.39605733 | 0.01293373 | 0.10640987 |
| NCOA6 | 1411.61294 | -0.4227224 | 0.01293071 | 0.10640987 |
| SCNN1D | 81.0540113 | 0.80531716 | 0.01292485 | 0.10640987 |
| CDPF1 | 63.1080039 | 0.48757096 | 0.01295794 | 0.10655918 |
| NUFIP2 | 4546.82794 | -0.2951646 | 0.01296923 | 0.10660219 |
| LEPR | 16039.4928 | 1.13460274 | 0.01298873 | 0.1067126 |
| LOC220729 | 418.006234 | -0.4598756 | 0.013003 | 0.10678005 |
| CLK4 | 431.552046 | 0.50422898 | 0.01302049 | 0.10687376 |
| ATAD5 | 155.22906 | -0.6035711 | 0.0130446 | 0.10699725 |
| P4HA2 | 1000.45007 | 0.6982131 | 0.0130477 | 0.10699725 |
| FAM26E | 321.117952 | 0.81058838 | 0.01308124 | 0.10718751 |
| KRT16 | 37.4048571 | -1.2735752 | 0.0130873 | 0.10718751 |

| | | | | |
|---|---|---|---|---|
| YDJC | 63.6388203 | -0.6138884 | 0.01308919 | 0.10718751 |
| KLHL31 | 27.6874593 | 0.88158593 | 0.01309676 | 0.10719961 |
| VAV2 | 510.529355 | -0.6368231 | 0.01312396 | 0.10737234 |
| ACAP1 | 26.7086243 | 0.8503598 | 0.01314126 | 0.107376 |
| FGF12 | 42.0837238 | 1.02709781 | 0.01314579 | 0.107376 |
| SPATA17 | 12.9576386 | -0.8726605 | 0.01314778 | 0.107376 |
| SSR4P1 | 19.7276751 | 0.94019903 | 0.01314883 | 0.107376 |
| PPP1R7 | 464.955348 | -0.4237314 | 0.01316284 | 0.1074405 |
| COMMD3 | 136.750367 | 0.46489079 | 0.01316996 | 0.10744881 |
| FAM102B | 404.871321 | 0.63820167 | 0.01319523 | 0.10760504 |
| KLHL29 | 1076.55604 | -1.102008 | 0.01320448 | 0.10763055 |
| KIF13A | 1442.11856 | 0.51628006 | 0.0132211 | 0.10771613 |
| ISPD | 63.8734177 | 0.67941747 | 0.01323969 | 0.10781765 |
| LOC286437 | 161.089393 | 0.4419421 | 0.01325314 | 0.10786094 |
| PIGO | 582.605954 | -0.363892 | 0.01325727 | 0.10786094 |
| ALG1L9P | 26.0601467 | 0.70938526 | 0.01328895 | 0.10806875 |
| ARHGEF2 | 1787.99027 | -0.4616076 | 0.01329562 | 0.10807277 |
| SBNO1 | 2033.24934 | -0.2988044 | 0.01330174 | 0.10807277 |
| NSUN5P2 | 159.901143 | 0.58077033 | 0.01332369 | 0.10815455 |
| TACR2 | 1.66965866 | 1.26210807 | 0.0133241 | 0.10815455 |
| AARS | 2290.84621 | -0.5328307 | 0.01337678 | 0.10842956 |
| CALN1 | 4.61157744 | -1.1921107 | 0.01340257 | 0.10842956 |
| NUDT9P1 | 11.4315562 | 1.02128096 | 0.01338204 | 0.10842956 |
| OR6W1P | 2.30199508 | -1.1913464 | 0.01340391 | 0.10842956 |
| SENCR | 3.33791416 | 1.02508474 | 0.01340149 | 0.10842956 |
| TBC1D19 | 341.360064 | 0.47599534 | 0.01336694 | 0.10842956 |
| TRIB1 | 316.84525 | 0.87521678 | 0.01339525 | 0.10842956 |
| TRIM61 | 26.4017129 | 0.58737515 | 0.01340729 | 0.10842956 |
| CAPS2 | 93.2605738 | 0.60842412 | 0.01344028 | 0.10864638 |
| CMTR1 | 781.961744 | 0.35645332 | 0.01346857 | 0.10868801 |
| HNRNPD | 1190.59673 | -0.497393 | 0.01346426 | 0.10868801 |
| PIEZO1 | 4006.42399 | -0.5267762 | 0.01347015 | 0.10868801 |
| VNN1 | 30.2810876 | 0.89516411 | 0.01346124 | 0.10868801 |
| ABCB4 | 33.5559559 | 0.84911103 | 0.01351035 | 0.10886256 |
| ACTG1 | 28071.0994 | -0.4047729 | 0.01350929 | 0.10886256 |
| ATP10B | 3.48652456 | -1.2419752 | 0.01350496 | 0.10886256 |
| TRAT1 | 3.62481835 | 1.16882959 | 0.01352039 | 0.1088936 |
| HN1L | 980.212124 | -0.4786807 | 0.01352702 | 0.10889714 |
| ERCC3 | 715.168043 | -0.2640257 | 0.01356048 | 0.10907656 |
| SGOL2 | 138.272914 | -0.6594302 | 0.01356171 | 0.10907656 |
| CLEC12A | 14.9363494 | 1.20218421 | 0.01356905 | 0.10908571 |
| SLC25A45 | 134.808263 | 0.68694282 | 0.0136117 | 0.10937865 |
| CEP162 | 260.994242 | 0.63611098 | 0.01362159 | 0.10940809 |
| ZNF426 | 374.97793 | 0.45002124 | 0.01367317 | 0.10977226 |
| ZBTB48 | 161.551934 | 0.47811598 | 0.01380871 | 0.1108099 |
| MED25 | 355.553707 | -0.3306966 | 0.01388499 | 0.11132039 |
| METTL13 | 375.697317 | -0.3695798 | 0.01388329 | 0.11132039 |
| TPM1 | 3502.53893 | 0.65335459 | 0.01390644 | 0.11144157 |
| EDAR | 6.26220872 | -1.2642539 | 0.01391534 | 0.11146211 |
| HDLBP | 9996.57967 | -0.3889842 | 0.01393159 | 0.11154149 |

| | | | | |
|---|---|---|---|---|
| CDH6 | 245.365006 | 1.08569767 | 0.01393877 | 0.11154824 |
| GIMAP2 | 96.5844368 | 0.70358902 | 0.01394906 | 0.1115798 |
| ERP44 | 989.139389 | -0.2541882 | 0.01395699 | 0.11159244 |
| FAM87A | 1.36964824 | 1.22229525 | 0.01398304 | 0.11174994 |
| ANKRD23 | 96.9699113 | -0.4573199 | 0.01400513 | 0.11187569 |
| KLK14 | 1.46445187 | -1.261703 | 0.01401651 | 0.11190972 |
| RORB | 1.72614072 | -1.2416578 | 0.01402212 | 0.11190972 |
| CYP1B1-AS1 | 28.4272791 | 1.1006298 | 0.0140299 | 0.11192104 |
| C10orf131 | 6.36538291 | 1.03689831 | 0.01406573 | 0.112156 |
| RPL34 | 2927.86885 | 0.45906791 | 0.01408742 | 0.11222726 |
| TLR8 | 52.1214509 | 0.96806396 | 0.01408537 | 0.11222726 |
| GCSH | 117.674334 | -0.6205266 | 0.01410353 | 0.11227955 |
| URB2 | 330.71244 | -0.4310364 | 0.01410675 | 0.11227955 |
| MAP3K9 | 181.077898 | -0.6856005 | 0.01413311 | 0.11243843 |
| CCDC147 | 6.53309278 | 1.05180332 | 0.01415059 | 0.11252659 |
| MROH9 | 2.49677848 | 1.18217481 | 0.01415795 | 0.11253423 |
| SKAP2 | 461.680458 | 0.67906221 | 0.01418526 | 0.1127004 |
| EML1 | 271.134284 | 0.67811396 | 0.01420917 | 0.11283577 |
| QPCT | 31.5268386 | 1.04707726 | 0.01421513 | 0.11283577 |
| DLEC1 | 99.105556 | 1.00218891 | 0.01422494 | 0.112838 |
| PLEKHF2 | 183.095843 | 0.35442768 | 0.01423111 | 0.112838 |
| TTBK1 | 6.62494902 | -1.1098188 | 0.01423465 | 0.112838 |
| HAPLN1 | 33.6846411 | -1.256739 | 0.01427339 | 0.11304322 |
| KRT10 | 239.435949 | -0.5994697 | 0.01426991 | 0.11304322 |
| SPTA1 | 3.49769645 | 1.10758314 | 0.01430062 | 0.1132079 |
| CALCR | 7.88014651 | 1.07772408 | 0.01431933 | 0.11330502 |
| NPEPPS | 1718.17291 | -0.3674811 | 0.01433327 | 0.1133643 |
| CENPM | 13.810056 | -0.9488749 | 0.01434784 | 0.11342854 |
| GPS1 | 1089.80612 | -0.2803623 | 0.01436639 | 0.11347321 |
| LPHN3 | 344.615058 | -1.2438929 | 0.01436012 | 0.11347321 |
| KIF22 | 255.348288 | -0.4725856 | 0.01439998 | 0.11363647 |
| PAM | 4232.58511 | 0.74480046 | 0.01439857 | 0.11363647 |
| GMFG | 80.4834946 | 0.71777067 | 0.01444567 | 0.11394589 |
| EPPK1 | 1044.10875 | -1.1441464 | 0.01446805 | 0.11399198 |
| FH | 549.138878 | -0.4871053 | 0.01448585 | 0.11399198 |
| LINC-PINT | 667.322094 | 0.5732055 | 0.01447162 | 0.11399198 |
| NFE2L2 | 2518.82983 | -0.499303 | 0.01448204 | 0.11399198 |
| NSFP1 | 3.43982201 | -0.9148012 | 0.01446539 | 0.11399198 |
| ST14 | 48.1829963 | -0.850542 | 0.0144904 | 0.11399198 |
| DLEU2L | 26.6992043 | 0.76997037 | 0.01451847 | 0.11416177 |
| GRIA4 | 3.01412661 | -1.2377456 | 0.01454361 | 0.11430837 |
| C9orf156 | 161.664825 | 0.29780757 | 0.01459967 | 0.11464655 |
| HSPB7 | 60.3476868 | 0.88817731 | 0.0145934 | 0.11464655 |
| B4GALNT3 | 20.222208 | 0.84579361 | 0.01464162 | 0.11487337 |
| SCARA3 | 4333.17705 | -0.7601584 | 0.01464162 | 0.11487337 |
| CD82 | 161.514609 | -0.9015279 | 0.01467058 | 0.11487355 |
| GNAI1 | 239.946798 | -0.7046352 | 0.01466788 | 0.11487355 |
| LINC00689 | 13.4211027 | -1.2530618 | 0.01467425 | 0.11487355 |
| P2RX1 | 7.26811683 | 1.10016983 | 0.01465516 | 0.11487355 |
| UBE2Q2P2 | 4.64547338 | -1.0373413 | 0.0146743 | 0.11487355 |

| | | | | |
|---|---|---|---|---|
| ZNF688 | 110.35104 | 0.43363575 | 0.01470263 | 0.11504413 |
| SNORA28 | 3.81580568 | 0.93418142 | 0.01471232 | 0.11506879 |
| CDK18 | 215.987998 | -0.7865878 | 0.01471922 | 0.11507155 |
| GDF15 | 164.41552 | -1.1582479 | 0.01473675 | 0.11514394 |
| LINC00174 | 207.434769 | -0.6082702 | 0.01474157 | 0.11514394 |
| ADC | 65.4148393 | 0.70893746 | 0.01477488 | 0.11519647 |
| DPEP1 | 8.27426572 | -1.2513772 | 0.01477511 | 0.11519647 |
| HIP1 | 805.513726 | 0.60782814 | 0.01476142 | 0.11519647 |
| LINC00861 | 7.75879575 | 0.93080005 | 0.01478104 | 0.11519647 |
| LOC1002881 | 13.0298617 | -1.1310317 | 0.01476097 | 0.11519647 |
| SHOX2 | 39.6118835 | -1.2270117 | 0.01480599 | 0.11533983 |
| C5orf27 | 1.67760537 | 1.25142983 | 0.01483306 | 0.11549209 |
| EIF3D | 1099.14014 | 0.42050089 | 0.01483867 | 0.11549209 |
| FCGR2A | 1078.17494 | 0.65380444 | 0.01484823 | 0.1155154 |
| KMO | 18.1280246 | 0.91558832 | 0.01485721 | 0.11553418 |
| CNDP1 | 4.0325359 | -1.2489277 | 0.01486933 | 0.1155773 |
| LOC1019270 | 15.0884918 | -1.2519334 | 0.01487663 | 0.11558301 |
| AP2M1 | 3356.31939 | -0.2196964 | 0.01490216 | 0.1156848 |
| PATL1 | 660.856971 | -0.4290482 | 0.01490289 | 0.1156848 |
| GATAD2A | 1009.48143 | -0.4256562 | 0.01491746 | 0.11574682 |
| HERC1 | 4893.26257 | 0.34731317 | 0.01493665 | 0.11584463 |
| COMMD2 | 617.795885 | 0.39798514 | 0.01498969 | 0.11594924 |
| HADH | 363.647013 | -0.3545237 | 0.01497118 | 0.11594924 |
| KIAA0247 | 3173.48115 | 0.64113004 | 0.01498406 | 0.11594924 |
| MVD | 250.582196 | -0.5977959 | 0.01495853 | 0.11594924 |
| ORC1 | 18.5123793 | -0.8136361 | 0.01497827 | 0.11594924 |
| SLC2A9 | 79.6319762 | 0.7763519 | 0.0149687 | 0.11594924 |
| LST1 | 1.76775754 | 1.20736856 | 0.01501892 | 0.11612434 |
| HEXB | 1279.61824 | 0.4233278 | 0.01504467 | 0.11627226 |
| ZFHX4 | 1145.46462 | -0.7391326 | 0.01505264 | 0.11628277 |
| CENPO | 214.892978 | -0.5410062 | 0.01511055 | 0.11637239 |
| FBXO4 | 125.386928 | 0.53076369 | 0.01508524 | 0.11637239 |
| NDUFA3 | 505.62272 | -0.4214873 | 0.01509958 | 0.11637239 |
| NEXN-AS1 | 9.78814837 | 1.03708387 | 0.01508712 | 0.11637239 |
| OR51B5 | 16.1320698 | 0.88862884 | 0.01509674 | 0.11637239 |
| SNORA61 | 5.16213449 | 0.92126932 | 0.01508004 | 0.11637239 |
| TOB2P1 | 11.9596795 | -1.0238819 | 0.01511023 | 0.11637239 |
| BOLA3 | 83.2231494 | -0.6109069 | 0.0151504 | 0.11656509 |
| LRRTM4 | 2.64830568 | -1.1853234 | 0.01515036 | 0.11656509 |
| NEAT1 | 26129.2731 | -0.6752856 | 0.01515545 | 0.11656509 |
| STAC2 | 11.1911642 | -1.1948699 | 0.01519301 | 0.11680288 |
| PCDHB9 | 275.324177 | -1.0218514 | 0.01521147 | 0.11689371 |
| IL6R | 365.900702 | 0.78521929 | 0.015224 | 0.11693897 |
| EIF3L | 2280.59498 | 0.52554215 | 0.0152325 | 0.11695319 |
| STAT5B | 1202.18454 | 0.39699136 | 0.01524179 | 0.1169734 |
| CCDC78 | 21.5450788 | -0.876187 | 0.01525624 | 0.11703231 |
| GSE1 | 1012.73337 | -0.5985577 | 0.01526568 | 0.11703231 |
| RPGRIP1 | 9.03574265 | 1.06270198 | 0.01526942 | 0.11703231 |
| LOC1001323 | 48.0001957 | 0.65234671 | 0.01529215 | 0.11715544 |
| MBD6 | 885.865038 | -0.4156389 | 0.01531403 | 0.11722099 |

| | | | | |
|---|---|---|---|---|
| SDHAP1 | 516.954413 | -0.5441823 | 0.01531199 | 0.11722099 |
| ABCB9 | 53.0105083 | -0.4793778 | 0.0153484 | 0.11738187 |
| ALYREF | 335.493172 | -0.3851224 | 0.01534423 | 0.11738187 |
| ASS1 | 3224.6212 | -0.715687 | 0.01536764 | 0.11747796 |
| TSPAN17 | 343.78064 | -0.5015222 | 0.01537627 | 0.11749288 |
| BHMT | 19.2833342 | 1.18855637 | 0.01543597 | 0.1175699 |
| FAM45B | 11.471693 | 0.64023592 | 0.01543366 | 0.1175699 |
| GNAI3 | 780.379574 | 0.4646368 | 0.01541985 | 0.1175699 |
| MYO5C | 931.810147 | -0.7295759 | 0.01540797 | 0.1175699 |
| POMGNT2 | 337.035203 | 0.53365382 | 0.01540488 | 0.1175699 |
| SENP6 | 1919.43057 | 0.46122914 | 0.015402 | 0.1175699 |
| SLC25A23 | 1470.81556 | -0.5247997 | 0.0154375 | 0.1175699 |
| ZNF818P | 200.625646 | 0.62610235 | 0.01543982 | 0.1175699 |
| SIRT1 | 480.543898 | 0.41353023 | 0.01544813 | 0.11758228 |
| ALB | 8.74818323 | 1.13081647 | 0.01545643 | 0.11759453 |
| ACKR2 | 27.0028024 | 1.0437618 | 0.01546506 | 0.11760932 |
| FAM76A | 152.107249 | 0.6168431 | 0.01549834 | 0.1178115 |
| CAPN1 | 1541.65555 | -0.384699 | 0.01551548 | 0.11785399 |
| NTRK1 | 3.05237524 | -1.0691841 | 0.01551846 | 0.11785399 |
| ZNHIT6 | 297.32681 | 0.35530311 | 0.01552403 | 0.11785399 |
| FBN2 | 3232.61463 | -0.9343474 | 0.01553566 | 0.11789138 |
| IFI6 | 632.30995 | 0.61240891 | 0.01556862 | 0.11809056 |
| C21orf58 | 84.5822832 | -0.618608 | 0.01559978 | 0.11817401 |
| NLRC4 | 45.3060286 | 0.81107778 | 0.01559726 | 0.11817401 |
| TNS3 | 2647.4907 | 0.90044845 | 0.01559559 | 0.11817401 |
| MGC16142 | 15.1843735 | 0.70266208 | 0.01560998 | 0.11820044 |
| CIRH1A | 367.855348 | -0.5182217 | 0.01563128 | 0.11826449 |
| UNC5A | 3.65386196 | -1.1910224 | 0.01563189 | 0.11826449 |
| HAUS1 | 145.935206 | 0.57528165 | 0.0156413 | 0.11828484 |
| LOC1005066 | 34.7666024 | 0.81821277 | 0.01571489 | 0.11873921 |
| LOC90784 | 292.880447 | -0.6566639 | 0.01571201 | 0.11873921 |
| TMIE | 3.14981364 | 1.12748332 | 0.015738 | 0.11883176 |
| UQCRC1 | 1280.10662 | -0.6087974 | 0.01574065 | 0.11883176 |
| RCAN1 | 488.134875 | 0.52190799 | 0.01576936 | 0.11899745 |
| AKAP3 | 10.1838475 | 0.97101711 | 0.01579433 | 0.1191348 |
| MICU3 | 149.599626 | 0.60431486 | 0.01580231 | 0.11914386 |
| GPC1 | 1224.94355 | -0.7739876 | 0.01583764 | 0.11935911 |
| C19orf66 | 328.962652 | 0.37682422 | 0.01586862 | 0.11951035 |
| MIR155HG | 22.1874183 | 0.98507548 | 0.0158713 | 0.11951035 |
| CCL19 | 2.39818569 | 1.21944974 | 0.01588628 | 0.11957198 |
| STAM2 | 993.125854 | 0.23640949 | 0.01589878 | 0.11961488 |
| DKFZp451B0 | 1.60015866 | 1.21888732 | 0.0159251 | 0.11964679 |
| NOTCH2NL | 1708.84794 | 0.41704837 | 0.01591843 | 0.11964679 |
| SHPK | 400.874133 | -0.4312795 | 0.01593023 | 0.11964679 |
| ZIC1 | 3995.73948 | -1.0414894 | 0.01592862 | 0.11964679 |
| ZBTB21 | 603.855811 | 0.60100489 | 0.01595503 | 0.11978186 |
| CHST1 | 56.6912046 | 0.81519604 | 0.0159734 | 0.11981757 |
| DDN | 5.32317992 | -1.1695367 | 0.01597289 | 0.11981757 |
| CCDC22 | 153.349388 | 0.28785008 | 0.01602175 | 0.11987348 |
| CCDC28B | 31.8521422 | -0.6362662 | 0.01600252 | 0.11987348 |

| | | | | |
|---|---|---|---|---|
| GNG3 | 2.10186931 | -1.2343705 | 0.01601898 | 0.11987348 |
| KRT4 | 30.2846316 | -1.184534 | 0.01600423 | 0.11987348 |
| LINC00412 | 9.67884405 | 0.76765392 | 0.01600286 | 0.11987348 |
| NFIL3 | 248.73075 | 0.96236282 | 0.01601567 | 0.11987348 |
| ERG | 192.599458 | 0.89829293 | 0.01605228 | 0.12005083 |
| HSCB | 40.5497933 | 0.46819328 | 0.01606245 | 0.12007589 |
| CHPT1 | 395.886944 | 0.67788495 | 0.01610232 | 0.12029546 |
| UNK | 791.942592 | -0.4122597 | 0.0161055 | 0.12029546 |
| RNF175 | 5.58120153 | 1.22046635 | 0.0161453 | 0.12043931 |
| SLC31A1 | 676.795471 | -0.5218087 | 0.0161333 | 0.12043931 |
| VSIG1 | 8.95449544 | -1.075749 | 0.0161402 | 0.12043931 |
| ADAM10 | 2090.079 | -0.3615527 | 0.01615877 | 0.12048364 |
| LYRM2 | 554.831832 | 0.60493979 | 0.01616495 | 0.12048364 |
| LOC284023 | 55.811801 | 0.5431662 | 0.01619259 | 0.12058747 |
| RRS1-AS1 | 323.45794 | -0.783172 | 0.01619001 | 0.12058747 |
| PNKD | 506.444471 | -0.5016499 | 0.0162513 | 0.12097352 |
| KLK10 | 93.2887591 | -1.1222467 | 0.01627154 | 0.12107289 |
| ANKRD13C | 534.644614 | 0.4366514 | 0.01628282 | 0.1211056 |
| GRTP1 | 91.3843747 | -0.6373269 | 0.01630718 | 0.12118431 |
| PINLYP | 77.5818182 | -0.4411063 | 0.01630485 | 0.12118431 |
| TXNL4B | 125.012111 | 0.6885896 | 0.01635273 | 0.12142679 |
| ZRANB1 | 1042.66994 | 0.35995898 | 0.01635361 | 0.12142679 |
| LDLRAP1 | 564.082293 | 0.72409681 | 0.01637046 | 0.12150057 |
| FAM188A | 403.906875 | 0.37908087 | 0.01640977 | 0.12174095 |
| ADAMTS5 | 1547.87796 | -1.2074387 | 0.01646376 | 0.12201464 |
| EVC2 | 238.902341 | 0.56826488 | 0.01648134 | 0.12201464 |
| LDLRAD2 | 11.17156 | 1.22597667 | 0.01647545 | 0.12201464 |
| PDPK1 | 1217.32719 | -0.3072523 | 0.01645617 | 0.12201464 |
| YIPF5 | 934.538213 | 0.50648096 | 0.01647608 | 0.12201464 |
| TMEM145 | 16.0594457 | -1.1090047 | 0.01649746 | 0.1220826 |
| LTBP2 | 12209.0176 | 1.06622671 | 0.01654737 | 0.1222976 |
| NHLRC4 | 20.4533166 | 0.92815726 | 0.01654214 | 0.1222976 |
| SNAI1 | 16.6488587 | 1.12507045 | 0.01653521 | 0.1222976 |
| CYB561D2 | 107.15457 | 0.43408637 | 0.01663565 | 0.12289842 |
| MTO1 | 285.419356 | 0.42852192 | 0.01666919 | 0.12304281 |
| PDZD4 | 1540.02841 | -0.5239095 | 0.01666762 | 0.12304281 |
| CHCHD5 | 100.149992 | -0.4675818 | 0.01669168 | 0.12315718 |
| SLMAP | 1179.40496 | 0.4923634 | 0.01673467 | 0.12342261 |
| GALC | 445.466678 | 0.50635299 | 0.01676693 | 0.12360869 |
| ID4 | 1574.74504 | -1.0611992 | 0.01679743 | 0.123678 |
| OVGP1 | 39.5638713 | 0.84079444 | 0.01679279 | 0.123678 |
| SCN4B | 600.794733 | 1.03577643 | 0.01679232 | 0.123678 |
| MRPS12 | 168.522291 | -0.4280847 | 0.01681222 | 0.12373512 |
| FRS2 | 1228.56547 | 0.20806862 | 0.01682771 | 0.12374554 |
| SNRPG | 253.00344 | -0.4178507 | 0.01682643 | 0.12374554 |
| PRICKLE3 | 77.6791809 | -0.4960801 | 0.01683744 | 0.12375029 |
| RPS18P9 | 56.3695793 | 0.64583328 | 0.01684242 | 0.12375029 |
| ATP13A1 | 880.520999 | -0.3524328 | 0.01686205 | 0.12379107 |
| GNG10 | 64.874661 | 0.40082127 | 0.01685739 | 0.12379107 |
| PPM1K | 537.927506 | 0.48653922 | 0.01688351 | 0.12389691 |

| | | | | |
|---|---|---|---|---|
| FN3KRP | 409.965049 | -0.4033351 | 0.01690867 | 0.12402985 |
| TMUB1 | 297.439901 | -0.4759638 | 0.01698572 | 0.12454307 |
| ANKRD31 | 30.8222985 | -0.9484377 | 0.0170068 | 0.12459376 |
| TNFRSF10C | 14.568077 | 0.92103523 | 0.01700533 | 0.12459376 |
| RHBDL3 | 88.9697933 | -0.8885354 | 0.01701973 | 0.12463658 |
| DHX33 | 422.509173 | -0.4161583 | 0.01705544 | 0.1247942 |
| HLA-DQB1 | 587.199442 | 1.20118235 | 0.01705205 | 0.1247942 |
| CADM2 | 10.4136585 | -1.2239324 | 0.01707108 | 0.12480476 |
| KLHL21 | 295.624705 | 0.5953951 | 0.01706957 | 0.12480476 |
| BTLA | 3.9608166 | 0.94320905 | 0.01711027 | 0.12503932 |
| WDR70 | 477.098621 | 0.32743792 | 0.01712498 | 0.12509484 |
| ADAMTS9-AS | 69.6413555 | 1.05003057 | 0.01718881 | 0.12540487 |
| GORAB | 169.20808 | 0.42737881 | 0.0171829 | 0.12540487 |
| RECK | 1379.9725 | -0.5241131 | 0.01717572 | 0.12540487 |
| MFAP5 | 1577.53 | 1.13750017 | 0.01722761 | 0.12563581 |
| CRABP1 | 6023.17308 | -1.1401613 | 0.01725974 | 0.12566619 |
| FGF13 | 8.86937171 | -1.0468769 | 0.01726182 | 0.12566619 |
| HBS1L | 721.081992 | 0.43583218 | 0.01725765 | 0.12566619 |
| PARM1 | 57.9631921 | 0.90084309 | 0.01725833 | 0.12566619 |
| POLD1 | 283.416759 | -0.4473143 | 0.01726749 | 0.12566619 |
| TTC38 | 176.211297 | 0.32878802 | 0.01729129 | 0.12578737 |
| ASTE1 | 123.27816 | 0.37436779 | 0.01734745 | 0.12589934 |
| CWF19L1 | 292.278705 | 0.38493006 | 0.01732526 | 0.12589934 |
| HTR7 | 18.9614089 | 1.01944344 | 0.01733589 | 0.12589934 |
| LAMTOR3 | 538.169836 | 0.34688776 | 0.01734271 | 0.12589934 |
| MED7 | 147.38885 | 0.36702661 | 0.01734963 | 0.12589934 |
| TBC1D26 | 1.69659189 | 1.1347752 | 0.017329 | 0.12589934 |
| DOCK11 | 455.862323 | 0.52660387 | 0.01741953 | 0.12606325 |
| EPHB3 | 108.615407 | 0.93519374 | 0.0174154 | 0.12606325 |
| FXR1 | 1835.64737 | -0.2688147 | 0.01738191 | 0.12606325 |
| PCDHGA1 | 146.406419 | -0.9278872 | 0.01742239 | 0.12606325 |
| PTK2 | 2149.64879 | -0.3061128 | 0.017409 | 0.12606325 |
| SAP25 | 90.4107356 | -0.6149695 | 0.01740765 | 0.12606325 |
| TRPC2 | 3.31925008 | 1.13480143 | 0.01740217 | 0.12606325 |
| HMHA1 | 580.71561 | 0.76978618 | 0.01747597 | 0.12623723 |
| LRRC37BP1 | 254.001929 | 0.3571886 | 0.01747963 | 0.12623723 |
| POTEE | 36.1149842 | -0.7634425 | 0.01746997 | 0.12623723 |
| PPTC7 | 359.92602 | -0.4939802 | 0.01748231 | 0.12623723 |
| ZNF664 | 2450.08196 | -0.4938254 | 0.01746687 | 0.12623723 |
| BTN3A1 | 365.524012 | 0.57604716 | 0.01749676 | 0.12623788 |
| CKLF | 54.7061044 | 0.64292406 | 0.01749047 | 0.12623788 |
| CCL26 | 77.9117202 | 1.08365428 | 0.01751516 | 0.12626709 |
| MYOZ1 | 41.2404142 | 1.15928768 | 0.01751347 | 0.12626709 |
| C21orf128 | 3.21191093 | -1.1878946 | 0.01754716 | 0.12628142 |
| GPR88 | 24.2211318 | 1.21953206 | 0.01753318 | 0.12628142 |
| RER1 | 858.520844 | 0.37737768 | 0.01754608 | 0.12628142 |
| TMEM150A | 153.598855 | 0.37882506 | 0.01753734 | 0.12628142 |
| ZFYVE16 | 1514.73254 | 0.38858027 | 0.01755305 | 0.12628142 |
| CUL3 | 1144.44518 | -0.2826773 | 0.01757687 | 0.12629783 |
| LOC441455 | 5.72410907 | 1.11819282 | 0.01757257 | 0.12629783 |

| | | | | |
|---|---|---|---|---|
| PIK3CD | 217.708388 | 0.76063099 | 0.01757272 | 0.12629783 |
| FRMD3 | 42.7695614 | 0.5524958 | 0.01761391 | 0.1264823 |
| SKA1 | 14.6382782 | -0.9325176 | 0.01761692 | 0.1264823 |
| ASH1L-AS1 | 57.2095834 | -0.5555873 | 0.01764282 | 0.12656229 |
| C1orf213 | 51.0773663 | 0.67027802 | 0.01764081 | 0.12656229 |
| ORC2 | 530.755438 | -0.2257503 | 0.01764965 | 0.12656229 |
| ISG20L2 | 372.478476 | -0.2999802 | 0.01766427 | 0.12661552 |
| SF3A2 | 494.863194 | -0.4485977 | 0.01768529 | 0.12671458 |
| HIST1H2BH | 142.710648 | -0.9846687 | 0.01773476 | 0.12681074 |
| KIF5A | 203.229145 | -1.1951528 | 0.01771641 | 0.12681074 |
| MGP | 3113.78849 | 0.79996454 | 0.01773146 | 0.12681074 |
| PLD3 | 5103.82038 | 0.70126558 | 0.01772836 | 0.12681074 |
| ZNF799 | 85.5289452 | 0.5945771 | 0.01773042 | 0.12681074 |
| CNTLN | 838.605205 | 0.41279858 | 0.01776362 | 0.12690649 |
| DOCK8 | 1134.03703 | 0.66060933 | 0.01776062 | 0.12690649 |
| EIF1B-AS1 | 17.9996719 | 0.82827248 | 0.0177698 | 0.12690649 |
| CTTNBP2 | 144.085813 | 1.02680209 | 0.01778183 | 0.12693533 |
| RUNX1-IT1 | 24.7889353 | 0.82649883 | 0.01778827 | 0.12693533 |
| AMZ1 | 5.37178341 | -1.1416163 | 0.017848 | 0.12728474 |
| LINC01105 | 6.12096281 | -1.2176078 | 0.0178517 | 0.12728474 |
| PCDHGB6 | 398.20236 | -0.8484708 | 0.01786466 | 0.12732553 |
| FER1L4 | 462.01982 | -0.9327065 | 0.01787252 | 0.12732998 |
| OTUD3 | 319.193637 | 0.56811154 | 0.01789239 | 0.12741992 |
| ADRM1 | 715.452496 | -0.4496354 | 0.01792085 | 0.12746775 |
| AGO2 | 706.622199 | -0.3838945 | 0.01792036 | 0.12746775 |
| B3GALT5 | 4.63391928 | -1.1667899 | 0.01790689 | 0.12746775 |
| KCNJ5 | 46.639109 | 0.76220326 | 0.01797256 | 0.12773228 |
| LIMD1-AS1 | 6.43559814 | 0.96278904 | 0.01796631 | 0.12773228 |
| STRN4 | 982.856253 | -0.2677645 | 0.01798458 | 0.12776604 |
| ATG4B | 635.563948 | -0.3732264 | 0.01800058 | 0.12782811 |
| SCARB2 | 2990.27891 | 0.35009122 | 0.01801461 | 0.12787609 |
| DLGAP1-AS2 | 12.3857047 | 0.75002685 | 0.01806245 | 0.1281261 |
| LINC00294 | 212.520957 | 0.44342822 | 0.0180644 | 0.1281261 |
| TBC1D10B | 607.005218 | -0.2957194 | 0.01808094 | 0.12819173 |
| FZD8 | 181.097157 | 0.70579283 | 0.01809783 | 0.12820815 |
| PTGIR | 6.36669564 | 1.07045238 | 0.01809417 | 0.12820815 |
| PMP2 | 16.3963299 | -1.1857319 | 0.01812709 | 0.12836373 |
| COX5B | 1038.30834 | -0.4849756 | 0.01813659 | 0.12837935 |
| FOPNL | 375.580776 | 0.32291037 | 0.0181483 | 0.12841054 |
| ZXDA | 227.941113 | 0.41703052 | 0.0181821 | 0.12859794 |
| BATF3 | 10.2438693 | 1.06146388 | 0.018209 | 0.1286847 |
| RASGRF2-AS | 2.46146715 | 1.21185304 | 0.01820244 | 0.1286847 |
| FGGY | 179.463029 | 0.49608873 | 0.0182425 | 0.12885987 |
| HAPLN3 | 69.5087713 | 0.99517931 | 0.01824843 | 0.12885987 |
| HHIPL1 | 81.0423643 | 0.79695421 | 0.01826818 | 0.12894752 |
| CTSC | 1310.77348 | -0.9046148 | 0.01827673 | 0.12895617 |
| ZNF407 | 830.473994 | 0.36349569 | 0.01828486 | 0.1289618 |
| LINC00467 | 50.7160623 | -0.6361161 | 0.01830535 | 0.12905453 |
| GGN | 5.34802273 | -0.9971445 | 0.0183304 | 0.12917936 |
| ATP5G3 | 1557.75168 | -0.5998609 | 0.0183411 | 0.12920301 |

| | | | | |
|---|---|---|---|---|
| RYK | 1109.58663 | 0.2783256 | 0.01834859 | 0.12920407 |
| GM2A | 1075.43973 | 0.32270765 | 0.01836734 | 0.12923263 |
| TNFAIP1 | 965.429219 | -0.3541293 | 0.01836611 | 0.12923263 |
| NEK7 | 1068.71028 | 0.32621972 | 0.01837622 | 0.12924337 |
| COQ7 | 319.227089 | 0.31970067 | 0.01840596 | 0.12935342 |
| NCAPD2 | 682.605805 | -0.3954637 | 0.01841014 | 0.12935342 |
| PCDHGB4 | 412.949842 | -0.8367136 | 0.01841393 | 0.12935342 |
| SIRT7 | 120.821191 | -0.4782833 | 0.01843579 | 0.12945532 |
| CIRBP | 4270.23654 | 0.47342875 | 0.01847439 | 0.1296746 |
| CEP57L1 | 161.719035 | 0.61520745 | 0.01849778 | 0.12973526 |
| GALNT4 | 13.5125667 | 0.72768225 | 0.01849573 | 0.12973526 |
| GOLGA2P7 | 97.8499178 | -0.6178131 | 0.01854276 | 0.12999889 |
| DLD | 1168.34532 | -0.4106832 | 0.01858264 | 0.13022654 |
| EVI5 | 909.140958 | 0.49112573 | 0.01859193 | 0.13023973 |
| BLACAT1 | 2.18744506 | -1.2084236 | 0.01863237 | 0.13047113 |
| RRP7B | 119.362232 | 0.50576098 | 0.01864075 | 0.13047787 |
| ZNF322 | 253.985889 | 0.4529534 | 0.01866415 | 0.13058969 |
| C20orf203 | 16.9625886 | 0.98861242 | 0.01868586 | 0.13065363 |
| PPP2R2C | 73.3034838 | -1.0757266 | 0.01868815 | 0.13065363 |
| CLK1 | 2311.83593 | 0.47991604 | 0.01870874 | 0.13069368 |
| CROCCP2 | 202.069261 | 0.61718554 | 0.01870678 | 0.13069368 |
| GGTA1P | 221.561618 | 0.78954218 | 0.01873617 | 0.13078142 |
| RPP25 | 81.4771471 | -1.0472373 | 0.01873214 | 0.13078142 |
| TNFRSF10A | 20.1251698 | 0.89360767 | 0.01875457 | 0.13085794 |
| UBR2 | 1627.01755 | 0.43485549 | 0.01877111 | 0.13092144 |
| DMBX1 | 12.7212476 | -1.2013785 | 0.01879392 | 0.13100603 |
| TMC3 | 4.85553803 | 1.19551678 | 0.01879814 | 0.13100603 |
| CCDC58 | 75.862956 | -0.4519992 | 0.01880718 | 0.13101714 |
| AGAP11 | 264.864654 | 0.87815612 | 0.01881869 | 0.13104542 |
| ADAMTS4 | 110.546578 | 1.03740159 | 0.01883883 | 0.13113377 |
| ZNF513 | 228.690349 | -0.3679572 | 0.01886964 | 0.1312963 |
| DDX41 | 669.645887 | -0.3128845 | 0.01888657 | 0.13131017 |
| TSIX | 16.5620458 | 1.18213852 | 0.0188835 | 0.13131017 |
| ZNRF1 | 146.536957 | -0.5354503 | 0.01889749 | 0.13133418 |
| TM6SF2 | 27.2971018 | -0.7804078 | 0.01890979 | 0.1313678 |
| MIER1 | 796.199396 | 0.43170769 | 0.01895837 | 0.13151124 |
| MMD2 | 1.42785939 | -1.1810182 | 0.01895617 | 0.13151124 |
| SCARNA10 | 4141.29921 | 0.42575583 | 0.01895169 | 0.13151124 |
| SEMA4G | 108.306124 | -0.7890921 | 0.01896782 | 0.13151124 |
| SLC35A3 | 391.772041 | 0.46908301 | 0.01896568 | 0.13151124 |
| PAX9 | 4.40823114 | -1.1808548 | 0.01901199 | 0.13171364 |
| SEMA3A | 62.2756074 | -1.1972194 | 0.01900774 | 0.13171364 |
| RPS2 | 8170.53671 | -0.4871434 | 0.01907989 | 0.13213202 |
| KIAA0226L | 90.6040974 | 0.82517593 | 0.01909007 | 0.13215045 |
| WBP4 | 240.802899 | 0.40501535 | 0.01910187 | 0.13218012 |
| RAD23B | 2922.47049 | -0.3010609 | 0.01911827 | 0.13224156 |
| C14orf132 | 327.314922 | 1.01144151 | 0.01914552 | 0.132378 |
| CHTOP | 627.00304 | -0.2472034 | 0.01915873 | 0.13241729 |
| FASLG | 1.58149491 | 1.19573955 | 0.01916669 | 0.13242032 |
| ABCC2 | 25.6058877 | 0.78279285 | 0.019215 | 0.13264097 |

| | | | | |
|---|---|---|---|---|
| ATP5J2 | 637.677347 | -0.3769351 | 0.01921012 | 0.13264097 |
| HIST1H2AH | 111.510033 | -0.7432187 | 0.01923633 | 0.13264097 |
| RRP7A | 374.757528 | 0.32485931 | 0.01922256 | 0.13264097 |
| SNX19 | 2855.25058 | -0.3111833 | 0.01923408 | 0.13264097 |
| C11orf53 | 3.84436301 | -1.2022426 | 0.01930989 | 0.13264208 |
| CAPN6 | 169.25765 | -1.1536852 | 0.01931618 | 0.13264208 |
| COMT | 553.824982 | 0.42791138 | 0.01932092 | 0.13264208 |
| DDX12P | 104.890152 | -0.6566637 | 0.01932049 | 0.13264208 |
| HIST1H2AG | 216.86582 | -0.8925885 | 0.01927683 | 0.13264208 |
| HIST1H3H | 127.286246 | -0.694482 | 0.01926712 | 0.13264208 |
| HPGD | 182.742463 | 1.08881976 | 0.01926657 | 0.13264208 |
| ICOS | 2.32270959 | 1.12847095 | 0.01931981 | 0.13264208 |
| NOTUM | 2.43845651 | -1.0761138 | 0.01924577 | 0.13264208 |
| RPPH1 | 88350.0298 | 0.3630559 | 0.01930818 | 0.13264208 |
| TMEM260 | 407.800665 | 0.46738151 | 0.01932698 | 0.13264208 |
| TMEM59 | 2087.19386 | 0.51827131 | 0.01925807 | 0.13264208 |
| MT1M | 11.821481 | 1.15917772 | 0.01934299 | 0.13270012 |
| AGRN | 4325.10607 | -0.6370926 | 0.01938757 | 0.13283925 |
| CIITA | 571.479516 | 0.86644262 | 0.01938167 | 0.13283925 |
| ETV7 | 18.1788379 | 0.89517105 | 0.01940889 | 0.13283925 |
| FAM212A | 33.9820786 | 0.93579343 | 0.01940565 | 0.13283925 |
| PRPF18 | 248.327115 | 0.37098454 | 0.01941613 | 0.13283925 |
| RABL3 | 263.633169 | 0.38689788 | 0.01941217 | 0.13283925 |
| TCP11 | 1.33381015 | 1.16483818 | 0.01940654 | 0.13283925 |
| OGDH | 2319.1214 | -0.5116648 | 0.01944721 | 0.1329485 |
| RP2 | 340.133793 | 0.38132556 | 0.01944617 | 0.1329485 |
| LURAP1L | 155.748661 | -1.0257479 | 0.01947774 | 0.13310548 |
| EPN2 | 619.397765 | -0.2873489 | 0.01950154 | 0.13320126 |
| JUP | 2481.25349 | -0.5996657 | 0.0195069 | 0.13320126 |
| TM2D1 | 250.068047 | 0.49393614 | 0.01957985 | 0.1336475 |
| COL2A1 | 2113.5285 | -1.1171761 | 0.01960285 | 0.13375258 |
| TNNI3 | 4.31224158 | -1.1725173 | 0.01961048 | 0.13375272 |
| SYT13 | 4.4903428 | -1.1547917 | 0.01962702 | 0.13381366 |
| GBP4 | 235.628734 | 0.75534924 | 0.01969446 | 0.13422142 |
| MORN2 | 82.1801757 | 0.65477723 | 0.01973776 | 0.13446444 |
| SIGLEC11 | 55.7525836 | 1.01498967 | 0.01975372 | 0.13452104 |
| MAPT | 37.5094298 | -0.9005335 | 0.0198922 | 0.13533995 |
| PHPT1 | 871.010425 | -0.3599938 | 0.01989705 | 0.13533995 |
| UBL4B | 78.2432099 | 1.17019545 | 0.01989365 | 0.13533995 |
| GIMAP1 | 102.06355 | 0.79500135 | 0.01990591 | 0.13534786 |
| OSCAR | 29.1761609 | 0.8293059 | 0.01995346 | 0.13556639 |
| ZNF790 | 145.365293 | 0.48365625 | 0.01995087 | 0.13556639 |
| CMPK2 | 101.343701 | 0.54732662 | 0.01997446 | 0.13565666 |
| NAGLU | 436.409366 | 0.52165421 | 0.01998276 | 0.13566043 |
| SCAMP1 | 1003.96194 | 0.4022738 | 0.01999044 | 0.13566043 |
| SNX2 | 1658.94676 | 0.50063094 | 0.02002603 | 0.13584958 |
| RPA2 | 377.589674 | 0.43611188 | 0.02003775 | 0.13587665 |
| THRB | 46.2296847 | 1.04260147 | 0.02009199 | 0.13619196 |
| NR2F2 | 2724.00323 | -0.9908574 | 0.02013377 | 0.13637004 |
| RTP1 | 1.28070757 | 1.18475832 | 0.02012709 | 0.13637004 |

| | | | | |
|---|---|---|---|---|
| AMER2 | 5.82384108 | -1.1938888 | 0.02014845 | 0.13641692 |
| EPB41L1 | 1777.44184 | -0.4802824 | 0.02017978 | 0.13656612 |
| TGM4 | 5.01426476 | -1.1895612 | 0.02018601 | 0.13656612 |
| ALPK3 | 278.001769 | 0.8905612 | 0.02023479 | 0.13678724 |
| CNTNAP3 | 449.574033 | -0.9272911 | 0.02022891 | 0.13678724 |
| EFNA2 | 11.2135371 | -0.9426364 | 0.02024202 | 0.13678724 |
| ABCA2 | 1133.59339 | -0.6623067 | 0.02027629 | 0.13691361 |
| ATG5 | 354.917825 | 0.39143227 | 0.02026988 | 0.13691361 |
| NELL1 | 56.952565 | -1.1909234 | 0.02031543 | 0.1370201 |
| PTPLB | 237.623248 | 0.57143284 | 0.0203148 | 0.1370201 |
| ZNF558 | 270.770498 | 0.38066499 | 0.02030488 | 0.1370201 |
| LSS | 1730.64353 | -0.5501576 | 0.02033324 | 0.1370463 |
| LTB4R2 | 53.9555084 | -0.6344587 | 0.0203349 | 0.1370463 |
| NRL | 6.7339895 | 0.68355218 | 0.02036267 | 0.13718094 |
| DUSP23 | 119.251354 | -0.5098406 | 0.02040896 | 0.13735956 |
| MYO1B | 319.959448 | -0.8406336 | 0.02042042 | 0.13735956 |
| QRICH2 | 206.207651 | 0.38265863 | 0.02039741 | 0.13735956 |
| SMPX | 1.45186379 | 1.00278783 | 0.02041882 | 0.13735956 |
| LOC1005065 | 599.131677 | 0.46756424 | 0.02048946 | 0.13774648 |
| WNT11 | 7.11154317 | -1.057469 | 0.02049361 | 0.13774648 |
| TMEM135 | 736.846847 | 0.40800202 | 0.0205097 | 0.13777375 |
| TRAPPC8 | 1103.50397 | 0.37506737 | 0.02051333 | 0.13777375 |
| SC5D | 776.977657 | -0.3830821 | 0.02052575 | 0.13780457 |
| AATK | 149.401996 | -0.7910415 | 0.02057393 | 0.13798365 |
| GPATCH3 | 104.319178 | 0.47584676 | 0.02057596 | 0.13798365 |
| HVCN1 | 143.356244 | 0.63699841 | 0.0205679 | 0.13798365 |
| TUBGCP3 | 617.596494 | -0.3496894 | 0.02059891 | 0.13808493 |
| MARK2 | 572.707845 | -0.3660357 | 0.02061567 | 0.13814459 |
| GSKIP | 91.4371207 | 0.49895405 | 0.02066233 | 0.13840453 |
| FLJ37201 | 25.4705171 | 0.77959017 | 0.02070175 | 0.13861585 |
| CEP72 | 63.0589905 | -0.4493509 | 0.02072392 | 0.13864931 |
| CRYM | 15.1845996 | -1.1826112 | 0.02073235 | 0.13864931 |
| MBLAC1 | 13.0605761 | 0.71410213 | 0.02073828 | 0.13864931 |
| PCSK9 | 24.8302562 | -1.130476 | 0.0207164 | 0.13864931 |
| ADAMTS14 | 10.318732 | -1.0446641 | 0.0207495 | 0.1386636 |
| AGAP3 | 892.781635 | -0.3789492 | 0.02076407 | 0.1386636 |
| KLC2 | 369.680981 | -0.4527857 | 0.02076362 | 0.1386636 |
| DUSP27 | 34.2636578 | 0.98986861 | 0.02080408 | 0.13883712 |
| GTF3C2 | 793.599615 | -0.3329293 | 0.02081084 | 0.13883712 |
| RBM15B | 1087.24527 | -0.3693117 | 0.02081373 | 0.13883712 |
| CCDC117 | 278.903307 | 0.41939427 | 0.0208406 | 0.13896367 |
| AKNA | 681.550614 | 0.45716503 | 0.02085712 | 0.13902111 |
| PTTG1 | 67.2064444 | -0.8854155 | 0.02087996 | 0.13912062 |
| ACKR1 | 6.99517371 | 1.11553294 | 0.02090343 | 0.13922427 |
| LTBP1 | 2609.06248 | 0.95084435 | 0.02091169 | 0.13922655 |
| NUPL2 | 231.969234 | 0.34274406 | 0.02091981 | 0.13922796 |
| IL13RA1 | 1643.59435 | 0.44590272 | 0.02093352 | 0.13924274 |
| UBTF | 2491.24674 | -0.319482 | 0.02093787 | 0.13924274 |
| ST8SIA3 | 52.5894541 | -1.1853199 | 0.02094843 | 0.13926036 |
| USP7 | 1899.04891 | -0.1478745 | 0.02096423 | 0.13931271 |

| | | | | |
|---|---|---|---|---|
| CABP1 | 2.96240915 | -1.1742599 | 0.0209911 | 0.13931454 |
| DOC2A | 33.8932638 | -0.9659116 | 0.02102218 | 0.13931454 |
| GFI1 | 7.38628728 | 0.8622671 | 0.0210437 | 0.13931454 |
| KDM2A | 2317.57699 | -0.2680049 | 0.02102788 | 0.13931454 |
| KNOP1 | 163.419734 | -0.4565234 | 0.0210315 | 0.13931454 |
| PICALM | 3262.32494 | 0.29412504 | 0.02099471 | 0.13931454 |
| PLLP | 31.9477573 | -0.921835 | 0.02099862 | 0.13931454 |
| PRKCA | 2990.08668 | -0.7244323 | 0.02101676 | 0.13931454 |
| SLC16A5 | 925.926778 | -0.8082879 | 0.02098009 | 0.13931454 |
| TRIM37 | 861.870872 | -0.4865002 | 0.02104223 | 0.13931454 |
| CHAMP1 | 300.645644 | -0.4955399 | 0.02108068 | 0.13950683 |
| ERMAP | 219.999567 | 0.61984344 | 0.02112068 | 0.13951607 |
| MSH6 | 946.029604 | -0.4290833 | 0.02112967 | 0.13951607 |
| MTMR9LP | 161.889609 | 0.98185723 | 0.02112281 | 0.13951607 |
| NOXRED1 | 7.08285056 | 0.8112119 | 0.02109816 | 0.13951607 |
| TRPV2 | 106.164722 | 0.65515944 | 0.02112013 | 0.13951607 |
| VAV1 | 181.1728 | 0.76569646 | 0.02110029 | 0.13951607 |
| RBP1 | 870.548966 | -0.9887752 | 0.02116584 | 0.1397025 |
| MAPK14 | 827.389015 | 0.37223468 | 0.02121025 | 0.13994312 |
| KIF26B | 34.011879 | 1.0115947 | 0.02124616 | 0.13997 |
| TCTEX1D1 | 3.29193443 | 1.0165559 | 0.02123317 | 0.13997 |
| ZBTB14 | 243.608424 | 0.35857244 | 0.02123418 | 0.13997 |
| ZNF566 | 253.098602 | 0.34429472 | 0.02123917 | 0.13997 |
| MFNG | 122.096569 | 0.63471498 | 0.02127239 | 0.14003792 |
| PRAC2 | 2.59163497 | -1.1161663 | 0.02126562 | 0.14003792 |
| HECTD4 | 5221.03483 | -0.3851433 | 0.02128902 | 0.140095 |
| RNF166 | 261.958076 | 0.49126321 | 0.02132142 | 0.1402557 |
| USP33 | 1110.76221 | 0.42360706 | 0.02134779 | 0.1403767 |
| RNF32 | 8.56184404 | -0.7770297 | 0.0213923 | 0.14061677 |
| EYA4 | 31.8207818 | -1.1743306 | 0.02140945 | 0.14067699 |
| LOC440311 | 1.55378968 | -1.1092299 | 0.02145756 | 0.1409396 |
| MXD3 | 97.8683955 | -0.4487267 | 0.02147322 | 0.1409396 |
| SNHG5 | 312.678066 | 0.75415509 | 0.02147346 | 0.1409396 |
| ABHD12B | 1.50412745 | -1.1796681 | 0.02149438 | 0.14102433 |
| EPN2-IT1 | 11.3517259 | -0.6565899 | 0.02151264 | 0.14109146 |
| CABYR | 15.6405737 | -1.010721 | 0.02156097 | 0.14121344 |
| CTSE | 1.49687832 | -1.1142887 | 0.02156222 | 0.14121344 |
| RASGRF2 | 1532.29275 | 0.76960736 | 0.02156252 | 0.14121344 |
| ZNF333 | 350.678956 | 0.3912263 | 0.02156335 | 0.14121344 |
| VIPR2 | 31.134779 | -1.1703198 | 0.02160478 | 0.14143211 |
| MB21D2 | 68.8107865 | 0.76914874 | 0.02161308 | 0.14143379 |
| TPBGL | 3.52678464 | -1.0970849 | 0.02169346 | 0.14190699 |
| SNORA34 | 39.5255362 | 0.81278162 | 0.02170211 | 0.14191079 |
| ATXN3 | 549.410449 | 0.44205285 | 0.02172925 | 0.14193004 |
| LOC145474 | 106.919097 | 0.78827882 | 0.02172545 | 0.14193004 |
| NQO1 | 887.098717 | -0.7329654 | 0.02172574 | 0.14193004 |
| MXD1 | 169.120691 | -0.5361008 | 0.02174021 | 0.14194887 |
| BBS10 | 410.010307 | 0.37059755 | 0.02177081 | 0.14204325 |
| SNORA23 | 216.465159 | -0.4335271 | 0.02177034 | 0.14204325 |
| LOC1019297 | 11.4790365 | 1.13079537 | 0.02179708 | 0.14216189 |

| | | | | |
|---|---|---|---|---|
| USP36 | 908.702588 | -0.4461883 | 0.02184222 | 0.1424035 |
| RHPN1-AS1 | 2.28034778 | -1.0214124 | 0.02186095 | 0.14247282 |
| ODF2 | 732.615776 | -0.3001716 | 0.0218776 | 0.14250572 |
| TIMM8A | 70.2656692 | -0.4150792 | 0.0218822 | 0.14250572 |
| LRRC42 | 301.259641 | 0.53035989 | 0.02192613 | 0.14268616 |
| ZFP37 | 118.339467 | 0.71909175 | 0.02191834 | 0.14268616 |
| MIA3 | 1922.4598 | -0.2734792 | 0.02195594 | 0.14282729 |
| RNA45S5 | 7239175.76 | -1.109938 | 0.02196784 | 0.14285185 |
| RPAP2 | 258.57948 | 0.41394935 | 0.02199274 | 0.14296091 |
| CLIC3 | 186.136485 | -0.9693712 | 0.02200137 | 0.14296419 |
| PIWIL4 | 44.2864992 | 0.54077244 | 0.02202376 | 0.14300404 |
| YIPF1 | 193.125417 | 0.50307635 | 0.02202321 | 0.14300404 |
| ANKRD46 | 285.00024 | 0.45730971 | 0.02205712 | 0.14300551 |
| PDCD1LG2 | 12.9429015 | 0.9217066 | 0.02205107 | 0.14300551 |
| PPIE | 288.725871 | 0.33864624 | 0.02206464 | 0.14300551 |
| PRMT9 | 306.994362 | 0.53912283 | 0.0220435 | 0.14300551 |
| SGK2 | 33.7989517 | 0.86168865 | 0.02206114 | 0.14300551 |
| FAM13C | 27.6955228 | -0.9225141 | 0.02208233 | 0.14306747 |
| GATAD1 | 977.547606 | 0.36175119 | 0.02210386 | 0.14315427 |
| LOC283683 | 30.6325717 | 1.04807838 | 0.02212244 | 0.14322181 |
| FAAH | 141.529287 | -0.8157932 | 0.02215803 | 0.1433995 |
| FBXO2 | 47.83577 | -1.0019384 | 0.02217223 | 0.14343861 |
| DENND6A | 756.556457 | 0.46324975 | 0.02227574 | 0.14400232 |
| EXOC6B | 609.293694 | -0.3338332 | 0.02226839 | 0.14400232 |
| AKR1A1 | 457.858532 | 0.41313073 | 0.02231739 | 0.14421857 |
| C1QTNF3 | 46.2268216 | 0.6684 | 0.02234237 | 0.14432696 |
| SH3BGRL2 | 380.395951 | 0.8680664 | 0.02235998 | 0.14438771 |
| SLC35B4 | 1147.66447 | -0.3732341 | 0.02239971 | 0.14459119 |
| MANEAL | 123.116597 | -0.9646923 | 0.02241702 | 0.14464978 |
| CD180 | 58.8601312 | 0.93057242 | 0.02242877 | 0.14467256 |
| EMC10 | 1644.02197 | -0.4841153 | 0.02243731 | 0.14467458 |
| GCLM | 207.941893 | 0.67644405 | 0.02247396 | 0.14479644 |
| TMEM97 | 326.920984 | -0.6096927 | 0.02247767 | 0.14479644 |
| TRAF2 | 122.555914 | -0.3635639 | 0.0224809 | 0.14479644 |
| RASSF1 | 219.499388 | 0.4790098 | 0.02250926 | 0.144873 |
| SLC16A13 | 31.8354518 | -0.7479789 | 0.02250476 | 0.144873 |
| PRR24 | 107.315749 | -0.3739479 | 0.02256105 | 0.14510016 |
| SLC25A18 | 5.24293249 | -1.146338 | 0.02255286 | 0.14510016 |
| CDKL3 | 47.1019481 | 0.55959794 | 0.02259659 | 0.1452756 |
| ANO4 | 55.1641555 | -1.1720195 | 0.02260938 | 0.14530469 |
| ANO7 | 33.0873216 | -0.7671802 | 0.02262325 | 0.14534073 |
| C12orf4 | 386.5365 | 0.35202006 | 0.02270974 | 0.14563045 |
| CHRNA5 | 15.7300343 | -1.0684798 | 0.02270122 | 0.14563045 |
| FTCD | 5.27121695 | -1.166937 | 0.02268978 | 0.14563045 |
| HOXC10 | 7.15884048 | -1.1039435 | 0.02268606 | 0.14563045 |
| STXBP1 | 550.37071 | -0.4403956 | 0.02270203 | 0.14563045 |
| WDR12 | 252.856675 | -0.3777361 | 0.02272192 | 0.14565547 |
| RAB9A | 190.923952 | 0.34813993 | 0.02273811 | 0.14570617 |
| ASIC4 | 3.91253504 | -1.1217688 | 0.02275612 | 0.14574644 |
| C6orf57 | 36.3525694 | 0.51557915 | 0.02283081 | 0.14574644 |

| | | | | |
|---|---|---|---|---|
| DSEL | 565.543822 | 0.70284065 | 0.02284383 | 0.14574644 |
| GAS2L2 | 3.54346705 | 1.10146742 | 0.02280225 | 0.14574644 |
| GTF2IRD2 | 83.8502202 | 0.49529087 | 0.02279137 | 0.14574644 |
| LOC1001283 | 58.349835 | -0.7533129 | 0.02280691 | 0.14574644 |
| SNORA14B | 4.66188067 | 0.94093988 | 0.02283932 | 0.14574644 |
| SUV39H1 | 93.1343228 | -0.4311541 | 0.02282637 | 0.14574644 |
| TOM1L1 | 387.214129 | -0.4187897 | 0.02281605 | 0.14574644 |
| UTRN | 8368.32818 | 0.4878453 | 0.02280177 | 0.14574644 |
| ZNF461 | 118.698223 | 0.48275432 | 0.02281377 | 0.14574644 |
| ZNF571 | 87.7497247 | 0.55711513 | 0.02283178 | 0.14574644 |
| PYHIN1 | 5.88551776 | 0.9850462 | 0.02285287 | 0.14575127 |
| KDM1A | 719.00567 | 0.38496498 | 0.02289942 | 0.14588311 |
| KIAA1919 | 363.66915 | 0.45647645 | 0.02290672 | 0.14588311 |
| LCMT2 | 262.440246 | -0.2948622 | 0.02288622 | 0.14588311 |
| SLC16A1-AS1 | 48.6828666 | 0.63148543 | 0.02289984 | 0.14588311 |
| PGAM5 | 353.092555 | -0.3866073 | 0.02293361 | 0.1460015 |
| GABRA2 | 2.39596876 | -1.1659968 | 0.02295177 | 0.14606426 |
| P2RY13 | 303.662961 | 0.88714096 | 0.02296864 | 0.14609903 |
| SLC25A10 | 62.3258938 | -0.6775806 | 0.02297384 | 0.14609903 |
| LINC01114 | 7.31567651 | 1.16860858 | 0.02300357 | 0.14618239 |
| MARK4 | 507.534155 | -0.459805 | 0.02300338 | 0.14618239 |
| CYB5RL | 76.24936 | 0.45423479 | 0.02301462 | 0.14619974 |
| ZNF628 | 95.4319927 | -0.5242396 | 0.02305015 | 0.14637263 |
| EBF1 | 666.668596 | 1.0391292 | 0.02309154 | 0.14651286 |
| VAPA | 1744.87672 | 0.4418301 | 0.02309722 | 0.14651286 |
| ZNF687 | 694.975096 | -0.3119419 | 0.02308602 | 0.14651286 |
| LOC284454 | 127.558221 | 0.80651349 | 0.02311455 | 0.14651706 |
| TOPBP1 | 818.273735 | -0.3110517 | 0.02311423 | 0.14651706 |
| CXCR4 | 584.255172 | 0.72972498 | 0.02315041 | 0.14663869 |
| TM6SF1 | 167.758218 | 0.79312131 | 0.02314511 | 0.14663869 |
| APP | 22567.5744 | -0.6767832 | 0.0231763 | 0.14665722 |
| BCAS4 | 122.53573 | -1.0003311 | 0.02317834 | 0.14665722 |
| FAM133CP | 249.403172 | 0.60558163 | 0.02316549 | 0.14665722 |
| ARSA | 403.186196 | 0.5279774 | 0.02323884 | 0.14686777 |
| KRT9 | 1.34669731 | -1.1644467 | 0.02322113 | 0.14686777 |
| LOC644762 | 2.05315684 | -1.1099228 | 0.02324502 | 0.14686777 |
| VPS9D1 | 278.243301 | -0.4634379 | 0.02323057 | 0.14686777 |
| AMMECR1 | 143.041848 | -0.4187597 | 0.02327301 | 0.14699181 |
| TBKBP1 | 294.998848 | -0.3626898 | 0.02331215 | 0.1471862 |
| CEP41 | 169.412785 | 0.44919586 | 0.02341916 | 0.14780875 |
| FLJ44087 | 13.5658354 | 1.11666973 | 0.0234994 | 0.14826201 |
| FBXO44 | 270.848594 | 0.35644903 | 0.02352392 | 0.14831031 |
| LZTFL1 | 353.592318 | 0.52593524 | 0.02351915 | 0.14831031 |
| EIF3G | 1035.5146 | 0.3681083 | 0.02355053 | 0.14842485 |
| TRMT13 | 218.381029 | 0.41701541 | 0.02358619 | 0.14859637 |
| NRGN | 29.2459566 | -1.0484839 | 0.02366882 | 0.14906357 |
| FAM43A | 605.45137 | -0.9276701 | 0.02368966 | 0.14914144 |
| C11orf1 | 162.445485 | -0.4916681 | 0.02370504 | 0.14918487 |
| MPO | 1.29029806 | 1.15486564 | 0.02376895 | 0.14953358 |
| IL20RB | 28.5726297 | -0.7587294 | 0.02379128 | 0.14962054 |

| | | | | |
|---|---|---|---|---|
| LOC284837 | 10.1599216 | 1.04159434 | 0.0238044 | 0.14964954 |
| NBPF14 | 1.33868242 | 1.11770941 | 0.02381515 | 0.14966364 |
| DKC1 | 492.482107 | -0.3271044 | 0.02382379 | 0.14966443 |
| BCAM | 697.903637 | -0.8670311 | 0.02385676 | 0.14981807 |
| COG5 | 1012.17638 | 0.26061391 | 0.02388437 | 0.14983254 |
| OCIAD2 | 81.6551468 | -0.8215368 | 0.02389598 | 0.14983254 |
| SGPP1 | 353.533717 | 0.55821062 | 0.02390165 | 0.14983254 |
| TLE1 | 188.46178 | 0.76038042 | 0.02389307 | 0.14983254 |
| TYMS | 281.937473 | -0.6943063 | 0.02387407 | 0.14983254 |
| LRRC25 | 145.701689 | 0.70435931 | 0.02391216 | 0.14984501 |
| TRNAU1AP | 124.542714 | 0.49741092 | 0.0239737 | 0.15017716 |
| VEGFB | 914.20998 | -0.5022457 | 0.02398939 | 0.15022189 |
| CD74 | 13997.8753 | 0.73587778 | 0.02400691 | 0.15027815 |
| ZZZ3 | 691.327552 | 0.42298675 | 0.02401686 | 0.15028691 |
| CA8 | 108.394455 | -1.0513055 | 0.02405843 | 0.15044 |
| SNORA71A | 44.4052054 | -0.8527776 | 0.0240533 | 0.15044 |
| RPGR | 93.182107 | 0.63333227 | 0.02411707 | 0.15075313 |
| UBE2Q2L | 6.07958101 | 0.97023336 | 0.0241417 | 0.15085348 |
| KRTAP5-AS1 | 1.65027619 | -1.1567699 | 0.0241839 | 0.15106271 |
| SPIN2B | 66.2032477 | -0.4401888 | 0.02419417 | 0.15106271 |
| ZNF25 | 403.212787 | 0.51488051 | 0.02420095 | 0.15106271 |
| RTCA | 259.84242 | 0.47556789 | 0.02420979 | 0.15106428 |
| LINC00887 | 129.536753 | -1.1190161 | 0.0242687 | 0.1513458 |
| TMEM119 | 241.85159 | 1.05755183 | 0.02427211 | 0.1513458 |
| FCHO2 | 1111.04489 | 0.33712406 | 0.02428129 | 0.15134936 |
| NXT2 | 118.164647 | 0.47734947 | 0.02429406 | 0.1513753 |
| ZDHHC17 | 1095.15507 | 0.29692156 | 0.02431378 | 0.15144452 |
| PPRC1 | 692.818752 | -0.4954754 | 0.02432501 | 0.15146084 |
| ZFY | 20.0408517 | -1.1381648 | 0.02433612 | 0.15147641 |
| DR1 | 552.322066 | 0.37583831 | 0.02440461 | 0.15184902 |
| CITED2 | 1438.56653 | 0.60448311 | 0.02443291 | 0.15197129 |
| PRKG2 | 319.209222 | -1.1545576 | 0.02444508 | 0.15198178 |
| RPS12 | 3929.33635 | 0.54889957 | 0.02445187 | 0.15198178 |
| LINC00526 | 19.4238322 | 0.59534329 | 0.0245125 | 0.15230479 |
| LAMA1 | 727.901864 | -1.083267 | 0.02452214 | 0.1523109 |
| CYP27A1 | 750.785532 | 0.39548505 | 0.02458733 | 0.15263099 |
| LOC727896 | 44.2935163 | 0.70086965 | 0.02459103 | 0.15263099 |
| KY | 2.36648907 | 1.14588203 | 0.02463472 | 0.15274716 |
| RPS15 | 2503.07766 | 0.35785588 | 0.02462268 | 0.15274716 |
| SH3BP5L | 387.513504 | -0.3107892 | 0.0246358 | 0.15274716 |
| PCSK7 | 847.433628 | -0.3152662 | 0.02466811 | 0.15289358 |
| AS3MT | 6.65510378 | 0.81791613 | 0.02469339 | 0.1529425 |
| GPR133 | 683.899119 | 0.97284803 | 0.02469273 | 0.1529425 |
| BMX | 8.10716473 | 1.0776649 | 0.02481937 | 0.1536687 |
| HYMAI | 107.414978 | 0.91529704 | 0.02484708 | 0.15378611 |
| CASC1 | 28.3036307 | 0.83588267 | 0.0248677 | 0.15378959 |
| RASGRP2 | 176.279748 | -0.8094588 | 0.02487387 | 0.15378959 |
| TNRC6C-AS1 | 198.030914 | -0.6664214 | 0.02487094 | 0.15378959 |
| ALKBH2 | 114.721509 | -0.4273725 | 0.02489514 | 0.15382719 |
| CHRNA4 | 288.17689 | -1.1523238 | 0.02489744 | 0.15382719 |

| | | | | |
|---|---|---|---|---|
| ZNF546 | 165.652415 | 0.39003261 | 0.02490939 | 0.15384696 |
| DIRAS1 | 77.7489031 | -1.0126152 | 0.02493539 | 0.15395351 |
| DYRK4 | 192.386222 | 0.37751744 | 0.02495369 | 0.15395839 |
| RGS4 | 53.6046751 | -1.1017794 | 0.0249454 | 0.15395839 |
| DNAL4 | 97.5988329 | 0.53405591 | 0.02501194 | 0.1542096 |
| PEX7 | 70.3003815 | 0.5956695 | 0.0250054 | 0.1542096 |
| CEACAM6 | 3.84850366 | -1.1457879 | 0.02504195 | 0.15423244 |
| FKBP11 | 142.410464 | -0.5649392 | 0.02504012 | 0.15423244 |
| ZNF512B | 1297.45608 | -0.5052564 | 0.02503787 | 0.15423244 |
| BBS2 | 813.156509 | 0.44257594 | 0.02507108 | 0.15435783 |
| DFFA | 295.021968 | 0.2633908 | 0.02510681 | 0.15437473 |
| GABRA5 | 2.23299568 | -1.1433536 | 0.02510823 | 0.15437473 |
| SPON1 | 83.7748093 | 1.02299939 | 0.02510893 | 0.15437473 |
| UBXN8 | 108.287951 | 0.37822544 | 0.02509999 | 0.15437473 |
| MSR1 | 1058.78578 | 0.72843027 | 0.02512199 | 0.15440108 |
| FAM212B | 76.9754028 | 0.70921322 | 0.02514672 | 0.15449908 |
| MTRNR2L3 | 4.75820798 | 0.99057467 | 0.0251707 | 0.15459242 |
| GIMAP4 | 355.441038 | 0.66622766 | 0.02518833 | 0.15464666 |
| CYP4X1 | 938.664431 | 0.75663067 | 0.02524846 | 0.15496178 |
| NDUFA13 | 1224.3029 | -0.3508613 | 0.02528273 | 0.15505052 |
| OR52N4 | 19.0507113 | 0.96210044 | 0.02528534 | 0.15505052 |
| SRSF2 | 2189.09344 | -0.2694978 | 0.02528937 | 0.15505052 |
| C17orf107 | 44.5371484 | 0.68707037 | 0.02536023 | 0.15518647 |
| CELF3 | 2.36237647 | -1.1357416 | 0.0253459 | 0.15518647 |
| CEP131 | 173.284962 | -0.4125684 | 0.02534825 | 0.15518647 |
| DNAH8 | 2.97277506 | 1.03698528 | 0.02536447 | 0.15518647 |
| GSTM3 | 570.017466 | 0.93505984 | 0.02532223 | 0.15518647 |
| POLR3GL | 275.808552 | 0.33226503 | 0.02535631 | 0.15518647 |
| PXDC1 | 378.715458 | 0.606238 | 0.02537556 | 0.15520032 |
| CBLN2 | 3.50396524 | -1.1271331 | 0.0254001 | 0.15520941 |
| LOC1002892 | 123.724851 | 0.42897855 | 0.02540352 | 0.15520941 |
| TNKS1BP1 | 2269.88849 | -0.3589662 | 0.02538798 | 0.15520941 |
| KCNMB1 | 29.7300776 | 0.82805919 | 0.02542954 | 0.15531446 |
| C21orf88 | 4.73498468 | -1.1310868 | 0.02545958 | 0.15539629 |
| WDR77 | 299.634799 | 0.3492198 | 0.02546061 | 0.15539629 |
| SCUBE1 | 561.792019 | 1.11784517 | 0.0255059 | 0.15561873 |
| TRIM22 | 3397.81618 | 0.41917242 | 0.0255311 | 0.1556645 |
| WIPF1 | 879.757898 | 0.38065349 | 0.02552741 | 0.1556645 |
| ZFPL1 | 254.34515 | -0.2996507 | 0.02556815 | 0.1558364 |
| C9orf169 | 5.74325543 | -0.9932948 | 0.02559334 | 0.15593588 |
| TCERG1L | 24.1384094 | -1.1434613 | 0.02563016 | 0.15610611 |
| EIF4E3 | 415.59589 | 0.62246327 | 0.02564533 | 0.15614444 |
| MCM3AP-AS | 89.8398167 | -0.8048922 | 0.02565909 | 0.15617418 |
| KCNJ1 | 1.66869134 | 1.04822814 | 0.02567347 | 0.15620768 |
| POGK | 872.557842 | -0.279723 | 0.02571025 | 0.15637734 |
| ZBED3 | 60.4511273 | -0.3853358 | 0.02572013 | 0.15638339 |
| TNFRSF10D | 213.630845 | 1.00292328 | 0.02574641 | 0.15648906 |
| FCGR1C | 30.8344835 | 0.91620667 | 0.02576576 | 0.1564985 |
| VWA3A | 9.44019557 | 0.87480668 | 0.02576374 | 0.1564985 |
| CACNA1D | 267.183799 | 1.11235995 | 0.02577625 | 0.1565082 |

| | | | | |
|---|---|---|---|---|
| HBP1 | 1237.41717 | 0.29896876 | 0.02579539 | 0.15656775 |
| UBA52 | 2931.52663 | 0.23771507 | 0.02580386 | 0.15656775 |
| ATM | 4043.91455 | 0.34079683 | 0.02581785 | 0.15659862 |
| ADRA1B | 51.1162006 | -0.8611527 | 0.02587593 | 0.1568968 |
| POLR2M | 98.4387645 | -0.4558602 | 0.02592032 | 0.15705765 |
| XRRA1 | 425.14664 | -0.6242673 | 0.02591835 | 0.15705765 |
| EMC7 | 566.697185 | -0.3870794 | 0.02593311 | 0.15708104 |
| COL9A3 | 6248.50479 | -0.9808974 | 0.02595748 | 0.15717452 |
| SCRIB | 891.784021 | -0.4183144 | 0.0259749 | 0.15722593 |
| CLMN | 714.271167 | -0.8084335 | 0.02599032 | 0.15726513 |
| GINS4 | 36.760441 | -0.6529448 | 0.02600208 | 0.15728217 |
| GPC6 | 6561.57161 | 0.57116247 | 0.02605301 | 0.15742782 |
| LOC1019295 | 7.29771753 | 1.01892043 | 0.0260406 | 0.15742782 |
| THEMIS2 | 424.468815 | 0.69801603 | 0.02605024 | 0.15742782 |
| RNF141 | 615.8538 | 0.47416249 | 0.02609814 | 0.15759224 |
| ZBTB5 | 482.191018 | -0.4446893 | 0.02609235 | 0.15759224 |
| FAM193A | 895.47397 | -0.3234247 | 0.02618395 | 0.15771084 |
| LOC1005062 | 13.9986733 | 0.83122506 | 0.0261434 | 0.15771084 |
| MRPS18C | 131.12973 | 0.34838 | 0.02613491 | 0.15771084 |
| POU5F2 | 40.7168004 | 0.54938247 | 0.02617115 | 0.15771084 |
| PUS1 | 130.095275 | -0.4051939 | 0.02615847 | 0.15771084 |
| TPT1-AS1 | 113.807328 | 0.4690803 | 0.02617637 | 0.15771084 |
| TRIM17 | 116.772804 | -0.7323088 | 0.02617893 | 0.15771084 |
| XYLT2 | 741.474434 | -0.4147394 | 0.0261895 | 0.15771084 |
| CNOT11 | 502.878054 | -0.2876135 | 0.02624729 | 0.15800111 |
| SERPINA1 | 298.376605 | 0.78651925 | 0.02625567 | 0.15800111 |
| IMPG2 | 100.794061 | 0.88688016 | 0.02628053 | 0.15809663 |
| BMPR1B | 2727.03093 | 1.07111363 | 0.0263257 | 0.158098 |
| CCDC88B | 255.936706 | 0.78636288 | 0.02630318 | 0.158098 |
| NOL4L | 765.960765 | -0.625692 | 0.02632077 | 0.158098 |
| PSIP1 | 1247.92096 | -0.5799651 | 0.02630986 | 0.158098 |
| SNCB | 3.87176405 | -1.1418219 | 0.0263065 | 0.158098 |
| IL23R | 4.07092125 | -1.1085575 | 0.02636111 | 0.15825662 |
| PPM1G | 862.85844 | -0.358852 | 0.02640125 | 0.1584423 |
| ZFP64 | 389.735405 | -0.4007204 | 0.02641005 | 0.1584423 |
| CYP2W1 | 11.0921481 | -1.1280987 | 0.0264436 | 0.15854289 |
| EIF5AL1 | 9.13049523 | -0.8427007 | 0.02644633 | 0.15854289 |
| STXBP3 | 488.77292 | 0.43334598 | 0.02645386 | 0.15854289 |
| CCNG1 | 3311.98823 | 0.44737807 | 0.02648987 | 0.15865061 |
| HPCA | 7.76732242 | -0.9691779 | 0.02648832 | 0.15865061 |
| MSL3 | 298.231517 | 0.37019851 | 0.02652114 | 0.15875414 |
| TRRAP | 2865.81864 | -0.344306 | 0.02652521 | 0.15875414 |
| NAA25 | 753.750742 | -0.2631373 | 0.02653889 | 0.158782 |
| HAUS2 | 350.305215 | 0.34815602 | 0.02659978 | 0.15897173 |
| QRSL1 | 223.784069 | 0.47774179 | 0.02660254 | 0.15897173 |
| TMEM68 | 186.468122 | 0.35971445 | 0.02660675 | 0.15897173 |
| ZNF317 | 554.586607 | 0.33993845 | 0.02660532 | 0.15897173 |
| PCDHGB8P | 247.892311 | -0.661721 | 0.02664922 | 0.15917141 |
| ABCC13 | 9.91086527 | -1.1199972 | 0.0266788 | 0.15926083 |
| PIBF1 | 531.853085 | 0.27493446 | 0.0266823 | 0.15926083 |

| | | | | |
|---|---|---|---|---|
| FHOD3 | 25.1216789 | 1.05159592 | 0.02671264 | 0.15938784 |
| S100A11 | 2528.49259 | -0.4666436 | 0.02674134 | 0.15950498 |
| PCDHGA11 | 406.266932 | -0.7044908 | 0.02680401 | 0.15982457 |
| WDR76 | 105.067775 | -0.6703798 | 0.02687693 | 0.16020507 |
| NINL | 1060.69833 | -0.645837 | 0.02689682 | 0.16026934 |
| FLI1 | 399.182654 | 0.60217416 | 0.02695677 | 0.16057218 |
| HHIP | 220.070093 | -1.1368159 | 0.02698575 | 0.16068245 |
| TMEM205 | 1267.06015 | -0.5715378 | 0.02699356 | 0.16068245 |
| ANP32E | 778.050563 | -0.3687404 | 0.02703845 | 0.16082334 |
| CXCL12 | 1151.4586 | -0.9802053 | 0.02704465 | 0.16082334 |
| NPDC1 | 844.466149 | -0.4467566 | 0.02702755 | 0.16082334 |
| BRSK2 | 19.4516827 | -0.8928752 | 0.02707842 | 0.1609697 |
| LOC1005063 | 42.2408272 | 0.92148527 | 0.0270994 | 0.1609917 |
| PPM1F | 557.030729 | 0.38807088 | 0.02710042 | 0.1609917 |
| BNIP3L | 2888.18182 | 0.42287329 | 0.02712887 | 0.16104711 |
| ELFN1 | 192.105627 | -1.0548402 | 0.02714089 | 0.16104711 |
| KCNE4 | 910.938561 | 0.8184689 | 0.02717426 | 0.16104711 |
| LOC1002893 | 3.65888864 | 0.77170552 | 0.02712611 | 0.16104711 |
| NNT | 2012.74394 | -0.44773 | 0.02714812 | 0.16104711 |
| PCNXL4 | 767.70695 | 0.4052282 | 0.02718299 | 0.16104711 |
| SLC44A1 | 2188.60549 | 0.71670672 | 0.02715878 | 0.16104711 |
| TMCC3 | 593.457182 | 0.55934362 | 0.02716564 | 0.16104711 |
| STX10 | 249.606799 | 0.29296826 | 0.02720694 | 0.1611347 |
| PTGER4 | 287.041525 | 0.80325511 | 0.02724066 | 0.16128012 |
| NOP16 | 129.626899 | -0.5198744 | 0.02728799 | 0.161506 |
| CD302 | 34.2334405 | -0.7118044 | 0.02731482 | 0.16159743 |
| CD3D | 6.44875466 | 1.0321086 | 0.02732182 | 0.16159743 |
| SURF4 | 3192.58501 | -0.3281438 | 0.02733959 | 0.16164819 |
| WDR11 | 1404.88891 | 0.42839271 | 0.02736569 | 0.16174812 |
| GPR65 | 100.592914 | 0.87769344 | 0.02740787 | 0.16184458 |
| INTS5 | 343.470111 | -0.334577 | 0.02740098 | 0.16184458 |
| TIMM50 | 368.787123 | -0.2898311 | 0.02740961 | 0.16184458 |
| MID1IP1-AS1 | 4.0315716 | -1.0406273 | 0.02742557 | 0.16188447 |
| SLC9A7P1 | 66.4332862 | 0.72254207 | 0.0274723 | 0.16210593 |
| GPR83 | 110.799199 | -1.061605 | 0.02752751 | 0.16237722 |
| ACTR3 | 2010.18188 | 0.19628702 | 0.0275817 | 0.16241852 |
| C10orf82 | 8.20135732 | 1.13258356 | 0.02756388 | 0.16241852 |
| DDX50 | 510.400789 | 0.40188679 | 0.02758221 | 0.16241852 |
| KIAA1456 | 13.5431182 | -1.0575453 | 0.02759399 | 0.16241852 |
| PCDHGB3 | 414.513982 | -0.7505127 | 0.02759059 | 0.16241852 |
| POC1B | 272.459075 | 0.34291534 | 0.02754732 | 0.16241852 |
| ZNF234 | 340.928081 | 0.32071836 | 0.02759915 | 0.16241852 |
| SMAP2 | 899.172315 | 0.70973784 | 0.02762711 | 0.16252875 |
| SEPT2 | 6654.54726 | -0.2399425 | 0.02767938 | 0.1627818 |
| ZNF410 | 422.99054 | 0.3810601 | 0.02774258 | 0.16309894 |
| HIST1H2BD | 530.098323 | -0.5058976 | 0.02778012 | 0.16323475 |
| LOC1001333 | 74.493895 | -0.6190639 | 0.02778424 | 0.16323475 |
| CD3E | 13.4113154 | 0.87031798 | 0.02779487 | 0.16324266 |
| PIANP | 18.4967091 | -1.062605 | 0.02780528 | 0.16324926 |
| CHN2 | 249.134431 | 0.96059354 | 0.02784764 | 0.16327737 |

| | | | | |
|---|---|---|---|---|
| ESCO1 | 489.537497 | 0.41468945 | 0.02784541 | 0.16327737 |
| LAT2 | 220.134347 | 0.70358483 | 0.02782845 | 0.16327737 |
| PRDX5 | 1532.10664 | -0.3608196 | 0.0278558 | 0.16327737 |
| SFT2D1 | 174.495408 | 0.41335699 | 0.02785647 | 0.16327737 |
| RAB40C | 274.767777 | -0.3915956 | 0.02788504 | 0.16339038 |
| BPTF | 4669.05615 | -0.2799034 | 0.02792201 | 0.16355251 |
| DUSP1 | 7492.72554 | 0.78348026 | 0.02797901 | 0.16365568 |
| ESM1 | 155.765628 | 1.06828597 | 0.02797515 | 0.16365568 |
| SNX29 | 1240.76013 | 0.47348331 | 0.02797685 | 0.16365568 |
| TMEM123 | 1814.10183 | 0.40987926 | 0.02798614 | 0.16365568 |
| ZNF787 | 220.876341 | -0.3492417 | 0.02797185 | 0.16365568 |
| HUWE1 | 9506.2104 | -0.2447647 | 0.02801601 | 0.16377588 |
| LOC1005075( | 22.1026642 | -0.5686124 | 0.02807752 | 0.16403754 |
| RPH3A | 3.9366871 | -1.1283025 | 0.02807942 | 0.16403754 |
| FLJ46906 | 10.8813784 | 1.00488173 | 0.02812255 | 0.16423497 |
| TGFB1 | 576.476332 | -0.5281176 | 0.02814227 | 0.16424108 |
| TRMU | 118.037643 | 0.40491109 | 0.02814208 | 0.16424108 |
| IGHMBP2 | 270.8365 | -0.2688781 | 0.0281891 | 0.16441036 |
| PILRA | 131.586353 | 0.72072985 | 0.02818997 | 0.16441036 |
| EOGT | 393.790787 | 0.61358677 | 0.0282498 | 0.16465011 |
| OSMR-AS1 | 7.91703707 | 1.01249963 | 0.02824186 | 0.16465011 |
| DOT1L | 576.888173 | -0.3354427 | 0.02828854 | 0.16482127 |
| SNAP91 | 71.0201676 | 0.97791056 | 0.02830237 | 0.16484726 |
| DNAJB6 | 1192.94204 | -0.2964111 | 0.02833047 | 0.16486459 |
| NAGA | 591.716232 | 0.37433655 | 0.02833347 | 0.16486459 |
| NFATC1 | 129.732068 | 0.58047043 | 0.02831591 | 0.16486459 |
| LBX1-AS1 | 3.679529 | -1.0968739 | 0.02835968 | 0.16490799 |
| SEMA3E | 3.91887352 | 1.09498066 | 0.02835654 | 0.16490799 |
| DPP3 | 380.302438 | -0.4115593 | 0.02841588 | 0.16512564 |
| MANBA | 533.875633 | 0.34471959 | 0.02841451 | 0.16512564 |
| METTL4 | 164.602419 | 0.50706147 | 0.02845029 | 0.16525399 |
| OVCA2 | 19.4184318 | -0.6708207 | 0.02846615 | 0.16525399 |
| PTPRN | 4.60730744 | -1.1257652 | 0.02846615 | 0.16525399 |
| SSTR5-AS1 | 1.83224796 | -1.1186978 | 0.02848901 | 0.1653321 |
| POLE2 | 23.4462566 | -0.7359222 | 0.02852734 | 0.16549995 |
| PODN | 622.082773 | 0.76903738 | 0.02854791 | 0.16556473 |
| SLC27A1 | 742.295981 | -0.4116786 | 0.02856847 | 0.16562935 |
| EID2B | 63.4597201 | 0.53289453 | 0.02868274 | 0.16612756 |
| RFXAP | 148.167664 | -0.3789686 | 0.02868054 | 0.16612756 |
| TIAF1 | 10.9690782 | 0.77165614 | 0.02867188 | 0.16612756 |
| ITM2C | 13078.7078 | -0.7935026 | 0.02869633 | 0.16615154 |
| CDC5L | 972.694789 | 0.33199143 | 0.02874702 | 0.16628087 |
| CXADRP2 | 3.28719766 | -1.1089757 | 0.02873715 | 0.16628087 |
| SNORA65 | 8.40709296 | -0.9584085 | 0.02873806 | 0.16628087 |
| ANAPC4 | 398.316195 | 0.26321212 | 0.02877808 | 0.16635974 |
| DSCAML1 | 13.352528 | -1.0753777 | 0.02877957 | 0.16635974 |
| ATP5L | 1058.48455 | -0.3328036 | 0.02892428 | 0.16683959 |
| CNOT6L | 1008.02742 | 0.33633741 | 0.02891346 | 0.16683959 |
| COX4I1 | 2023.5 | -0.4385468 | 0.02892139 | 0.16683959 |
| HMGB3 | 131.329332 | -0.6484016 | 0.02889481 | 0.16683959 |

| | | | | |
|---|---|---|---|---|
| LOC1019272 | 7.15939356 | 0.87269207 | 0.02892898 | 0.16683959 |
| RASL12 | 36.4137269 | 0.92471017 | 0.02891057 | 0.16683959 |
| RNF13 | 1301.47742 | 0.22774876 | 0.02888438 | 0.16683959 |
| LRP2BP | 60.521462 | 0.81551396 | 0.02893869 | 0.16684088 |
| NBPF1 | 989.623941 | 0.59966667 | 0.02894826 | 0.1668414 |
| WHAMMP1 | 136.833587 | 0.37760056 | 0.02898219 | 0.16698221 |
| PNMAL2 | 94.9021033 | 0.81580626 | 0.0290024 | 0.16704392 |
| B4GALT3 | 295.295686 | -0.3721747 | 0.02901529 | 0.16706348 |
| PIGK | 505.13983 | 0.37318197 | 0.02904211 | 0.16716322 |
| OTOG | 473.274579 | -1.119898 | 0.02905393 | 0.16717653 |
| LINC01234 | 14.1245046 | -1.061757 | 0.02906533 | 0.16718745 |
| GHR | 691.091394 | 0.60880065 | 0.02907958 | 0.16721474 |
| DEF6 | 174.986212 | 0.57853519 | 0.02916061 | 0.16762584 |
| PODXL | 4296.30291 | -0.7238078 | 0.0292076 | 0.16784114 |
| FAHD2A | 127.508966 | 0.42131716 | 0.02922801 | 0.16790356 |
| BET1 | 216.646252 | 0.25771761 | 0.02927019 | 0.16792648 |
| COX17 | 236.768026 | -0.4153693 | 0.02926576 | 0.16792648 |
| MFSD1 | 1012.67974 | 0.34185674 | 0.0292559 | 0.16792648 |
| PUS10 | 93.508515 | 0.43403127 | 0.0292596 | 0.16792648 |
| NSDHL | 188.753146 | -0.4487803 | 0.02931038 | 0.16810227 |
| MET | 3786.49178 | -0.9145013 | 0.02935257 | 0.16828933 |
| LINC00909 | 185.188974 | 0.50814188 | 0.02939889 | 0.16844509 |
| VAT1 | 1774.53022 | 0.52731957 | 0.02939558 | 0.16844509 |
| IL10 | 8.13968759 | 1.0691232 | 0.02940979 | 0.16845271 |
| CCDC40 | 143.391138 | 0.70099364 | 0.0295303 | 0.1690879 |
| MTRNR2L2 | 4035.9529 | -0.645655 | 0.0295725 | 0.16921934 |
| TMC8 | 132.888408 | 0.64000729 | 0.02957159 | 0.16921934 |
| TSPAN7 | 998.37661 | 0.99725953 | 0.0295925 | 0.1692787 |
| TMEM189 | 426.044526 | -0.5644965 | 0.02960248 | 0.16928077 |
| PRICKLE2 | 444.677456 | 0.70686218 | 0.02961451 | 0.16929452 |
| KCNG3 | 11.0582523 | -1.0844917 | 0.02964464 | 0.1693567 |
| TVP23C | 292.369093 | 0.47218982 | 0.0296365 | 0.1693567 |
| HAUS7 | 306.015746 | 0.53091429 | 0.02979227 | 0.17003443 |
| NTNG1 | 14.7366704 | -1.1142105 | 0.0297737 | 0.17003443 |
| TAS2R10 | 23.4643085 | 0.64988157 | 0.02978477 | 0.17003443 |
| C20orf196 | 20.7150415 | 0.66849375 | 0.02981209 | 0.17009235 |
| HIST1H2BF | 92.6572201 | -0.7292934 | 0.02983294 | 0.17010094 |
| UBQLN2 | 913.922869 | -0.302505 | 0.02982782 | 0.17010094 |
| RILPL1 | 318.924933 | -0.393818 | 0.02990284 | 0.17044423 |
| SUCLG1 | 629.383775 | -0.3641999 | 0.02994308 | 0.17061832 |
| XRCC4 | 109.345222 | 0.46406546 | 0.02996509 | 0.17068848 |
| INPP5E | 277.99569 | -0.3374869 | 0.03000134 | 0.1707843 |
| RASGRP1 | 26.7995425 | 0.86912732 | 0.02999801 | 0.1707843 |
| CCRN4L | 45.1436851 | -0.7614147 | 0.0300401 | 0.17083923 |
| GNPDA1 | 344.247953 | 0.30955905 | 0.03004563 | 0.17083923 |
| RNF180 | 246.898602 | 0.9234869 | 0.03002583 | 0.17083923 |
| ZNF587 | 1545.35482 | -0.3317432 | 0.03004983 | 0.17083923 |
| RELA | 831.711381 | -0.3298157 | 0.03006912 | 0.17089364 |
| PEF1 | 536.619055 | 0.40112052 | 0.03008207 | 0.17091201 |
| HIP1R | 338.627001 | -0.6438153 | 0.03011 | 0.17096026 |

| | | | | |
|---|---|---|---|---|
| TFIP11 | 283.15955 | 0.39011716 | 0.03010662 | 0.17096026 |
| TMSB15A | 3.64763739 | -0.987224 | 0.03012839 | 0.17100948 |
| DUOX1 | 173.23759 | 1.11073896 | 0.03014321 | 0.17101161 |
| NAA30 | 386.96055 | 0.37661969 | 0.03014821 | 0.17101161 |
| ZNF711 | 230.818554 | -0.813662 | 0.03016112 | 0.17102969 |
| CDK8 | 157.394771 | -0.435108 | 0.03018691 | 0.17106563 |
| CYP20A1 | 932.373623 | 0.27884846 | 0.0301859 | 0.17106563 |
| HDAC5 | 769.72435 | 0.34016595 | 0.03023691 | 0.17129379 |
| ALDH1B1 | 219.257299 | -0.6200088 | 0.03025211 | 0.17132475 |
| CD2BP2 | 823.19129 | 0.23560402 | 0.03030749 | 0.17158313 |
| CRNKL1 | 801.232045 | 0.32967752 | 0.03034933 | 0.17172187 |
| METTL25 | 122.073482 | 0.42272799 | 0.03035152 | 0.17172187 |
| PSENEN | 209.786636 | 0.34509128 | 0.03037458 | 0.17179704 |
| ACCS | 220.670213 | 0.58786464 | 0.03038734 | 0.17181398 |
| AMZ2 | 601.949723 | -0.2818465 | 0.03048662 | 0.17196491 |
| C19orf25 | 223.065895 | 0.42805391 | 0.03047761 | 0.17196491 |
| H6PD | 1658.42389 | 0.50826782 | 0.03049792 | 0.17196491 |
| LINC00612 | 6.99805088 | -0.8614238 | 0.03050202 | 0.17196491 |
| LOC644936 | 2.66246472 | 1.06217604 | 0.0304656 | 0.17196491 |
| NDUFS2 | 859.577747 | -0.4464326 | 0.03049117 | 0.17196491 |
| PUF60 | 901.996494 | -0.2671176 | 0.03047638 | 0.17196491 |
| SALL1 | 208.365536 | -1.0785112 | 0.03048636 | 0.17196491 |
| WDR78 | 90.1347772 | 0.87971756 | 0.03046039 | 0.17196491 |
| GFM1 | 844.680282 | -0.315205 | 0.03052135 | 0.17201877 |
| ARHGEF3 | 836.827019 | 0.80430297 | 0.03064004 | 0.1726324 |
| ASGR2 | 3.87258022 | 0.98763016 | 0.03065732 | 0.17266609 |
| RAB13 | 604.137322 | -0.3989106 | 0.03066565 | 0.17266609 |
| ESYT3 | 141.968844 | 0.91256672 | 0.03069444 | 0.17277284 |
| ARHGDIG | 23.3948858 | 0.9970966 | 0.03076915 | 0.17299132 |
| DMGDH | 173.029029 | 0.87560219 | 0.03074563 | 0.17299132 |
| SPDYE5 | 1.29350774 | 1.08653105 | 0.03077255 | 0.17299132 |
| WBP1L | 4773.91336 | 0.68206694 | 0.03077259 | 0.17299132 |
| VTN | 240.947162 | -0.8342588 | 0.03078351 | 0.17299742 |
| C19orf38 | 17.836551 | 0.84027668 | 0.03084129 | 0.1732668 |
| LYRM7 | 430.199442 | 0.47380661 | 0.03091072 | 0.17354598 |
| NCOR2 | 4192.57968 | -0.4276492 | 0.03090904 | 0.17354598 |
| ZDHHC22 | 2.45186915 | -1.0717136 | 0.03092205 | 0.17355421 |
| CACNA1B | 12.110647 | -0.9081087 | 0.030969 | 0.17376228 |
| ENO4 | 13.7807625 | 0.82952585 | 0.03099041 | 0.17382694 |
| TBXAS1 | 334.417678 | 0.7152917 | 0.03105799 | 0.17415046 |
| CILP2 | 52.3717697 | 1.06538786 | 0.03107177 | 0.17417221 |
| CLCN4 | 329.723817 | -0.5679338 | 0.0310876 | 0.17419725 |
| FAM71F1 | 3.1376057 | 1.03415403 | 0.03109604 | 0.17419725 |
| NTMT1 | 146.77866 | -0.2915894 | 0.03111467 | 0.17424609 |
| CD276 | 1170.14917 | -0.5967627 | 0.03114543 | 0.17436285 |
| LOC652276 | 85.0059607 | -0.4764863 | 0.03116345 | 0.17440821 |
| ANKS6 | 806.123298 | -0.436248 | 0.03123009 | 0.17441361 |
| ECM1 | 62.8492554 | -0.8872053 | 0.03130132 | 0.17441361 |
| GEN1 | 272.513514 | -0.5440266 | 0.03119013 | 0.17441361 |
| GRAMD4 | 283.546265 | 0.43792073 | 0.03125754 | 0.17441361 |

| | | | | |
|---|---|---|---|---|
| GTPBP10 | 423.784126 | 0.23817469 | 0.03126505 | 0.17441361 |
| HIST1H2BN | 140.681304 | -0.5825545 | 0.03130344 | 0.17441361 |
| IRF6 | 6.15154371 | 0.88946556 | 0.03121746 | 0.17441361 |
| ISCU | 876.125392 | 0.32981693 | 0.03129872 | 0.17441361 |
| LOC1002870 | 26.4423378 | 0.56295445 | 0.03120877 | 0.17441361 |
| MICAL2 | 1292.24519 | -0.5628925 | 0.03125521 | 0.17441361 |
| PIP4K2A | 457.155895 | 0.39182826 | 0.03132231 | 0.17441361 |
| RARRES3 | 188.781149 | 0.68022878 | 0.03124801 | 0.17441361 |
| RFX3 | 388.369445 | 0.50940111 | 0.03127838 | 0.17441361 |
| RFX7 | 824.340803 | -0.3817781 | 0.03130986 | 0.17441361 |
| SPCS2 | 518.43151 | -0.3386513 | 0.03132306 | 0.17441361 |
| STX12 | 748.963295 | 0.46785429 | 0.03131391 | 0.17441361 |
| LSM10 | 118.828753 | -0.5171565 | 0.03133444 | 0.17442178 |
| C9orf89 | 89.2909417 | -0.4393479 | 0.03134944 | 0.17445004 |
| KIF24 | 84.7203209 | -0.55177 | 0.03142888 | 0.1748368 |
| ME3 | 137.353629 | 0.56695763 | 0.03147715 | 0.17495662 |
| NRP2 | 3637.59713 | 0.86200226 | 0.03147981 | 0.17495662 |
| RBMX2 | 182.230601 | 0.31805964 | 0.03148026 | 0.17495662 |
| ABCC4 | 931.105552 | -0.96013 | 0.03156169 | 0.17529843 |
| SLC24A2 | 5.71164845 | -1.1042249 | 0.03155433 | 0.17529843 |
| BMP1 | 1151.79766 | -0.5437435 | 0.03159482 | 0.17542706 |
| CCR4 | 3.84773713 | 1.02757788 | 0.03161382 | 0.17547713 |
| LRRC58 | 1464.17826 | -0.2472066 | 0.03164093 | 0.17557222 |
| BAI3 | 48.6417289 | 0.94992779 | 0.03167559 | 0.17561387 |
| MAP2K3 | 343.765625 | -0.5134735 | 0.03166139 | 0.17561387 |
| PELO | 533.484203 | 0.46008963 | 0.03167839 | 0.17561387 |
| CD53 | 452.935995 | 0.67061235 | 0.03170097 | 0.17568368 |
| FBXO10 | 259.360952 | -0.4955109 | 0.03174361 | 0.17581187 |
| NUGGC | 3.90558258 | 1.00413896 | 0.03174409 | 0.17581187 |
| SERPINE1 | 306.074725 | 1.00183726 | 0.03175489 | 0.17581635 |
| TUBGCP4 | 352.381663 | -0.3368594 | 0.03178082 | 0.17590456 |
| DCP2 | 1396.74078 | 0.40081357 | 0.03187979 | 0.17639684 |
| ADM5 | 17.1412735 | -0.8457618 | 0.03190285 | 0.17641344 |
| LOC1005056 | 1.72240681 | 1.08174437 | 0.03189575 | 0.17641344 |
| RAB24 | 751.33496 | -0.5462763 | 0.03194533 | 0.17659281 |
| FRY | 707.948603 | 0.86877996 | 0.03197175 | 0.17668332 |
| SNORA31 | 4.52467172 | 0.72050402 | 0.03198619 | 0.17670761 |
| PCDH7 | 23.1111336 | -1.071513 | 0.03204555 | 0.17697996 |
| SCN2B | 182.000489 | 1.07959073 | 0.03207093 | 0.177009 |
| SNRNP40 | 297.750659 | 0.39320531 | 0.03206283 | 0.177009 |
| SSPO | 404.676972 | -0.6946385 | 0.03209044 | 0.17706113 |
| FCN1 | 10.0013606 | 0.99187656 | 0.03214176 | 0.17719196 |
| HIST1H2BB | 54.0646522 | -0.7670903 | 0.03214323 | 0.17719196 |
| TMEM156 | 31.4381538 | 1.01869706 | 0.03214437 | 0.17719196 |
| RAB14 | 1875.80057 | -0.2381738 | 0.03215875 | 0.17721566 |
| LRRC14 | 482.228933 | -0.3283456 | 0.03220615 | 0.17742128 |
| SYT15 | 378.871655 | 0.91814328 | 0.03224128 | 0.17755922 |
| OSTF1 | 245.924006 | 0.3484874 | 0.0322648 | 0.17763315 |
| DEPDC5 | 269.424119 | -0.3478477 | 0.03228715 | 0.17763534 |
| FBXO48 | 12.9345857 | 0.69341235 | 0.03228832 | 0.17763534 |

| | | | | |
|---|---|---|---|---|
| ZNF517 | 61.9434893 | -0.3837463 | 0.0322955 | 0.17763534 |
| LOC1001323 | 69.6990163 | 0.29728877 | 0.03239247 | 0.178113 |
| LYN | 402.699487 | 0.50799352 | 0.03242975 | 0.17825061 |
| MKRN3 | 4.24995195 | -1.0424549 | 0.03243776 | 0.17825061 |
| CRLF1 | 248.964644 | 1.03012065 | 0.03245443 | 0.17828651 |
| LIN9 | 76.309942 | -0.5225343 | 0.03247641 | 0.1783422 |
| LOC1004994 | 30.0456093 | 0.50349443 | 0.0325041 | 0.1783422 |
| NDFIP1 | 2192.6226 | 0.40233904 | 0.03251526 | 0.1783422 |
| PLXDC2 | 3154.35294 | 0.69834144 | 0.03250809 | 0.1783422 |
| ZFP91 | 1287.08299 | -0.2351078 | 0.03249996 | 0.1783422 |
| AGPAT9 | 64.6733205 | 0.77949422 | 0.03256409 | 0.17855437 |
| CTNND1 | 172.065796 | 0.27888455 | 0.03261324 | 0.17876812 |
| PCSK2 | 11.1163616 | -1.0961643 | 0.0326917 | 0.17911298 |
| RIN1 | 53.1149554 | -0.7976736 | 0.03269652 | 0.17911298 |
| LINC01184 | 240.921035 | 0.35769307 | 0.03272727 | 0.17920309 |
| MAPK1 | 1929.93216 | 0.25506157 | 0.03273334 | 0.17920309 |
| TOB1 | 2165.06113 | -0.6034116 | 0.03274796 | 0.1792273 |
| CTDSPL | 809.024127 | 0.63562442 | 0.03279206 | 0.17941283 |
| FAM228B | 117.273444 | 0.55137253 | 0.03280394 | 0.17942202 |
| HLA-DQA2 | 1.93300648 | 1.0196172 | 0.03283356 | 0.17952825 |
| RNF187 | 1260.21689 | -0.3202939 | 0.03285141 | 0.17957002 |
| CCSER1 | 66.246113 | -0.9081541 | 0.03287274 | 0.17963077 |
| COMMD6 | 447.819947 | 0.41128095 | 0.03292146 | 0.17982287 |
| NPW | 46.2574119 | -1.0619165 | 0.03292834 | 0.17982287 |
| CCDC11 | 9.89179026 | 0.79446971 | 0.03294903 | 0.17988001 |
| ABO | 37.1507997 | 1.02831065 | 0.03299886 | 0.1799194 |
| BAIAP2 | 447.725488 | -0.617979 | 0.0330483 | 0.1799194 |
| CCDC81 | 19.2079182 | 0.86590284 | 0.0330434 | 0.1799194 |
| CDHR2 | 3.20363096 | -0.9681638 | 0.03303407 | 0.1799194 |
| CLDN10 | 1.40248455 | -1.0721677 | 0.03304108 | 0.1799194 |
| DOPEY1 | 1033.41719 | 0.5325941 | 0.03303341 | 0.1799194 |
| LOC1027248 | 51.8760487 | 0.51298695 | 0.03299557 | 0.1799194 |
| SNRPA1 | 166.501535 | -0.496145 | 0.03302232 | 0.1799194 |
| TEAD4 | 128.262172 | -0.5721367 | 0.03301015 | 0.1799194 |
| ABI1 | 698.71873 | 0.25639172 | 0.03307471 | 0.18000745 |
| G3BP2 | 2209.21759 | -0.3533426 | 0.03308693 | 0.18001828 |
| C18orf25 | 558.110326 | 0.47547702 | 0.03314448 | 0.18026072 |
| IFNLR1 | 85.3819315 | 0.77819537 | 0.03319298 | 0.18026072 |
| KCNH8 | 2.04743478 | -1.0932273 | 0.03318963 | 0.18026072 |
| LOC1001293 | 415.367903 | -0.4100526 | 0.03318293 | 0.18026072 |
| PRDM11 | 487.865278 | -0.5375927 | 0.03317228 | 0.18026072 |
| YPEL3 | 427.022497 | 0.42326085 | 0.03316416 | 0.18026072 |
| CSDC2 | 135.867506 | -0.9461617 | 0.03322839 | 0.18036225 |
| GOLGA8M | 31.7201046 | 0.9422689 | 0.03323218 | 0.18036225 |
| KNTC1 | 580.388023 | -0.4726416 | 0.03327709 | 0.18055029 |
| COL23A1 | 44.760332 | 1.07264056 | 0.03335938 | 0.18074813 |
| NEO1 | 5202.04336 | -0.5467622 | 0.03336493 | 0.18074813 |
| TMEM151B | 9.77858524 | -0.9935044 | 0.03333651 | 0.18074813 |
| XAB2 | 533.88947 | 0.31885456 | 0.03332679 | 0.18074813 |
| ZMIZ1 | 3422.92943 | -0.5567616 | 0.03336251 | 0.18074813 |

| | | | | |
|---|---|---|---|---|
| LOC1009964 | 10.7234824 | 0.95626017 | 0.03338039 | 0.18077622 |
| ANKRD6 | 202.290736 | 0.65527321 | 0.03341383 | 0.18090159 |
| TECR | 954.703161 | -0.5489734 | 0.03344337 | 0.18100583 |
| ATG10 | 104.453903 | 0.47734184 | 0.03354416 | 0.1813325 |
| LOC1019287 | 20.2783723 | 0.8804373 | 0.03354497 | 0.1813325 |
| RABGAP1 | 1955.6251 | -0.255677 | 0.03353026 | 0.1813325 |
| SGMS1 | 741.496264 | 0.40037615 | 0.03353033 | 0.1813325 |
| CAMSAP1 | 868.927633 | -0.2932902 | 0.03362455 | 0.181353 |
| CEP85L | 421.316696 | 0.50329699 | 0.03357481 | 0.181353 |
| HEATR2 | 281.816632 | -0.3426524 | 0.03363124 | 0.181353 |
| HIST1H2BI | 126.67062 | -0.6202905 | 0.03360918 | 0.181353 |
| IL7R | 48.2315128 | 0.84489683 | 0.03361239 | 0.181353 |
| JMJD1C | 3772.04454 | 0.39092644 | 0.03358362 | 0.181353 |
| RPL5 | 4729.8575 | 0.41238728 | 0.03363116 | 0.181353 |
| TGIF2 | 371.323904 | -0.4883932 | 0.03358205 | 0.181353 |
| ALDH3A1 | 4.54017626 | -1.0376907 | 0.03364583 | 0.18137606 |
| CDK10 | 325.990868 | -0.3472637 | 0.03368981 | 0.1815575 |
| PLEKHA1 | 1108.19914 | 0.52746141 | 0.03371106 | 0.18161637 |
| EMBP1 | 13.8229289 | 0.77142541 | 0.03373858 | 0.1816534 |
| SEC1P | 3.8205024 | 0.98780588 | 0.03373639 | 0.1816534 |
| PLXNB3 | 29.1759803 | -0.8990754 | 0.03376365 | 0.18173274 |
| KANK4 | 21.3621067 | -1.0016096 | 0.03379338 | 0.18183713 |
| HS6ST1 | 768.545759 | -0.6083433 | 0.03380969 | 0.1818407 |
| SMAGP | 131.47637 | -0.9389578 | 0.03381472 | 0.1818407 |
| SRPRB | 848.727959 | -0.4258988 | 0.03383713 | 0.18190561 |
| KRTCAP3 | 8.42610799 | -0.8187264 | 0.03386578 | 0.18200401 |
| FAM155B | 12.7648467 | -0.7950002 | 0.03405004 | 0.18293835 |
| LRRC4B | 129.584642 | -1.0569176 | 0.03408689 | 0.18308042 |
| VWDE | 2.37069831 | -1.0831157 | 0.03412615 | 0.18323534 |
| MAGI2-AS3 | 1724.82006 | 0.37579095 | 0.03421856 | 0.18367543 |
| LMCD1-AS1 | 13.0897049 | 0.98280066 | 0.03424492 | 0.18376085 |
| HSPA7 | 340.107917 | 0.88503348 | 0.03433164 | 0.18382717 |
| NPIPB5 | 76.8335512 | -0.6000502 | 0.03434088 | 0.18382717 |
| PCDHGA6 | 633.870053 | -0.7647559 | 0.03429779 | 0.18382717 |
| PDIA3 | 5616.31562 | -0.360936 | 0.03432924 | 0.18382717 |
| PPP1R16A | 271.313049 | -0.3974664 | 0.03430658 | 0.18382717 |
| PSMB10 | 234.078427 | 0.43911056 | 0.03433302 | 0.18382717 |
| SYK | 593.574212 | 0.58962207 | 0.03430571 | 0.18382717 |
| ZNF7 | 305.422935 | -0.3026624 | 0.03432597 | 0.18382717 |
| ALKBH6 | 63.0773505 | -0.3576773 | 0.03437414 | 0.18394924 |
| PATL2 | 9.53630374 | 0.82083102 | 0.03440746 | 0.18407154 |
| LOC400927 | 12.2044679 | 0.60639757 | 0.03453293 | 0.18437055 |
| NCKIPSD | 573.664194 | -0.4279261 | 0.03448509 | 0.18437055 |
| NRBP1 | 1147.93452 | -0.3571549 | 0.03453047 | 0.18437055 |
| PIK3C2B | 2567.56678 | -0.5275716 | 0.03448167 | 0.18437055 |
| PRKD1 | 96.6698443 | 0.85757647 | 0.03454318 | 0.18437055 |
| RASSF2 | 5738.17722 | 0.80703378 | 0.03454118 | 0.18437055 |
| TMOD2 | 854.467629 | 0.50133768 | 0.03451156 | 0.18437055 |
| ZNF181 | 362.187515 | 0.28114218 | 0.0345472 | 0.18437055 |
| LINC00884 | 2.61937694 | 0.97881289 | 0.03455814 | 0.18437296 |

| | | | | |
|---|---|---|---|---|
| PARP1 | 2115.88668 | -0.3213802 | 0.03458727 | 0.18443928 |
| TMEM186 | 88.5775628 | -0.3714566 | 0.03459154 | 0.18443928 |
| MOK | 138.18863 | 0.63237057 | 0.03462027 | 0.18453651 |
| FOXD3 | 8.80665725 | -1.0088846 | 0.03464739 | 0.1846245 |
| WNK2 | 226.685623 | -1.0288439 | 0.03465777 | 0.1846245 |
| CTLA4 | 4.90382073 | 1.04291001 | 0.03467598 | 0.18466561 |
| SETD8 | 586.246464 | -0.3872122 | 0.03471362 | 0.1848101 |
| CHRDL1 | 49.5530635 | -0.9703618 | 0.03474019 | 0.18489561 |
| CCDC89 | 35.7866377 | -0.7258092 | 0.03480719 | 0.18511619 |
| LSR | 611.919003 | -0.7084122 | 0.03481321 | 0.18511619 |
| MAN1C1 | 2141.05434 | 0.62092849 | 0.03480688 | 0.18511619 |
| LOC1019283 | 4.94811841 | 0.98582517 | 0.03485229 | 0.18526802 |
| CENPT | 472.353609 | -0.2604288 | 0.0349085 | 0.18534275 |
| FAM134C | 1263.46729 | 0.29151826 | 0.03489426 | 0.18534275 |
| SMG5 | 1447.87688 | -0.2844759 | 0.03490681 | 0.18534275 |
| VPS26B | 1057.72619 | -0.2320113 | 0.03488452 | 0.18534275 |
| AKIP1 | 180.704204 | 0.38167609 | 0.03498198 | 0.18544966 |
| BRWD1-IT2 | 17.733317 | -0.6846713 | 0.03498219 | 0.18544966 |
| GPA33 | 2.08475576 | 0.95116835 | 0.03500244 | 0.18544966 |
| IGFL2 | 5.41940341 | -1.0758932 | 0.0349762 | 0.18544966 |
| SCNN1A | 291.786032 | -0.996062 | 0.03500034 | 0.18544966 |
| SYNE3 | 144.437534 | 0.67822363 | 0.03498329 | 0.18544966 |
| UNC5CL | 18.185967 | 0.63772298 | 0.03498209 | 0.18544966 |
| EIF4EBP1 | 158.941122 | -0.5889765 | 0.03501802 | 0.18547636 |
| ADM | 467.234036 | 0.985295 | 0.03505145 | 0.18548587 |
| CCR1 | 188.497248 | 0.75916809 | 0.03503652 | 0.18548587 |
| MST1P2 | 23.991054 | -0.87528 | 0.03504555 | 0.18548587 |
| CLEC2D | 328.666787 | 0.50246729 | 0.03508439 | 0.18559672 |
| LOC1005070 | 18.096686 | 0.78275172 | 0.0350935 | 0.18559672 |
| LOC1019272 | 1.44561409 | 1.03115828 | 0.03511501 | 0.18564827 |
| ULK4P3 | 4.3400452 | -1.0397339 | 0.035133 | 0.18564827 |
| WDFY4 | 441.952034 | 0.65805359 | 0.03513491 | 0.18564827 |
| LOC1001343 | 3.61084512 | 1.00439794 | 0.03515887 | 0.18571906 |
| METTL20 | 58.985435 | 0.51185428 | 0.0352085 | 0.1859254 |
| ADAR | 5694.80417 | -0.2339432 | 0.03531612 | 0.18604119 |
| AGPAT3 | 1577.5178 | -0.3754813 | 0.03541023 | 0.18604119 |
| AHCYL2 | 6250.16472 | 0.59189845 | 0.03534661 | 0.18604119 |
| FHOD1 | 603.5887 | -0.58773 | 0.03526197 | 0.18604119 |
| GFRA2 | 3.2630694 | -0.9678218 | 0.03526784 | 0.18604119 |
| KCNJ13 | 29.1411939 | -1.0802917 | 0.03529835 | 0.18604119 |
| LIPC | 8.59055215 | 1.01880773 | 0.03540885 | 0.18604119 |
| LRRC37A4P | 346.796044 | -0.547402 | 0.03538962 | 0.18604119 |
| OSGEP | 224.700335 | 0.39677302 | 0.03536374 | 0.18604119 |
| PAPSS1 | 959.74067 | -0.4196963 | 0.03526764 | 0.18604119 |
| PLEKHO1 | 354.145246 | 0.47580691 | 0.03538307 | 0.18604119 |
| SLC26A11 | 304.971097 | -0.3997851 | 0.03528016 | 0.18604119 |
| SNORA18 | 21.3464624 | -0.5773429 | 0.03537528 | 0.18604119 |
| TBC1D2B | 1682.05852 | 0.32960792 | 0.0353717 | 0.18604119 |
| UBE3C | 1589.09352 | -0.2071443 | 0.03535104 | 0.18604119 |
| VEGFC | 20.2826916 | 0.94943455 | 0.03535129 | 0.18604119 |

| | | | | |
|---|---|---|---|---|
| WIPI1 | 1501.30983 | -0.5031937 | 0.03530562 | 0.18604119 |
| HTR2C | 3.8762911 | 1.07390724 | 0.03543391 | 0.18611003 |
| LOC1005061 | 20.5565693 | -0.6485823 | 0.03545925 | 0.18618755 |
| CD163L1 | 90.5191424 | -0.873995 | 0.0355166 | 0.18637737 |
| CDC42SE1 | 1209.99462 | 0.26529007 | 0.035512 | 0.18637737 |
| PIEZO2 | 3223.89496 | -0.9625185 | 0.0356561 | 0.18705364 |
| ZC3H8 | 93.1745975 | -0.3685947 | 0.03567549 | 0.18709956 |
| SNX5 | 737.132049 | 0.37630545 | 0.03571727 | 0.18726284 |
| CEP95 | 510.124907 | -0.3600305 | 0.03576129 | 0.18728757 |
| ETNK1 | 1516.12565 | 0.38752123 | 0.03576458 | 0.18728757 |
| GPC5 | 15.340395 | -1.0298745 | 0.03573994 | 0.18728757 |
| PEX3 | 204.821614 | 0.40142228 | 0.03576096 | 0.18728757 |
| ATP5G2 | 1498.02183 | -0.3019617 | 0.03580742 | 0.18734461 |
| BEND6 | 64.2795737 | 0.854102 | 0.03580346 | 0.18734461 |
| TCTN3 | 796.761873 | 0.34393875 | 0.03579486 | 0.18734461 |
| ZNF124 | 334.043142 | 0.52823232 | 0.03582109 | 0.18736038 |
| CRIM1 | 5558.84356 | 0.59887114 | 0.03585256 | 0.18746923 |
| LACE1 | 58.9520415 | 0.45626276 | 0.03590375 | 0.18760501 |
| MRPS25 | 1069.80726 | -0.4958018 | 0.03589287 | 0.18760501 |
| SRPR | 2238.34882 | -0.2621241 | 0.03591052 | 0.18760501 |
| PJA2 | 3938.55036 | 0.41515933 | 0.03592133 | 0.18760573 |
| BEX1 | 40.8284228 | -1.0253153 | 0.03594589 | 0.18767829 |
| METRNL | 924.340845 | -0.6694701 | 0.03597199 | 0.18775886 |
| SEL1L3 | 461.305493 | -0.6703042 | 0.03606345 | 0.18818036 |
| CDK19 | 442.211633 | 0.40365334 | 0.03619635 | 0.18865 |
| DTX4 | 717.563842 | -0.6960138 | 0.03617718 | 0.18865 |
| FLVCR1 | 210.389679 | -0.4289433 | 0.03617897 | 0.18865 |
| SHPRH | 954.639107 | 0.32429424 | 0.03618962 | 0.18865 |
| TEX15 | 520.71065 | -1.0289284 | 0.0362207 | 0.18872099 |
| MRPL18 | 284.925036 | 0.3395863 | 0.03625829 | 0.18886094 |
| TNFRSF14 | 292.678056 | 0.52811084 | 0.03628548 | 0.1889466 |
| SLC2A14 | 11.9098947 | -0.9004853 | 0.03634252 | 0.18918758 |
| SPINK8 | 1.38016885 | -1.0238467 | 0.03646055 | 0.1897459 |
| PALD1 | 993.438053 | 0.61418123 | 0.03650101 | 0.18990026 |
| CD40 | 97.9579427 | 0.85570972 | 0.03652691 | 0.18994578 |
| ISLR2 | 26.1541354 | -0.9309332 | 0.03653136 | 0.18994578 |
| FMOD | 4295.79147 | 0.46203758 | 0.03657498 | 0.1900041 |
| ITGB1BP1 | 387.026199 | -0.3695507 | 0.03655948 | 0.1900041 |
| RPS9 | 3736.68306 | 0.34586845 | 0.03657089 | 0.1900041 |
| CASP1 | 162.300759 | 0.44816274 | 0.0367028 | 0.19021871 |
| FYB | 547.539411 | 0.66881356 | 0.03663676 | 0.19021871 |
| HAVCR2 | 342.653711 | 0.67575407 | 0.03668783 | 0.19021871 |
| LINC00858 | 2.62201988 | -0.9995042 | 0.03665299 | 0.19021871 |
| LPHN1 | 1147.6857 | -0.4912562 | 0.03670032 | 0.19021871 |
| PRDM1 | 170.978757 | 0.63388555 | 0.03669893 | 0.19021871 |
| SSBP2 | 934.240871 | 0.65419381 | 0.0366817 | 0.19021871 |
| TP53I11 | 5662.95749 | -0.8868852 | 0.0366775 | 0.19021871 |
| KDM7A | 518.572888 | 0.35628467 | 0.03674498 | 0.19029081 |
| LOC1019291 | 3.57611737 | -1.0309781 | 0.03672835 | 0.19029081 |
| RPL36A | 400.756731 | 0.42655701 | 0.03674917 | 0.19029081 |

| | | | | |
|---|---|---|---|---|
| TMEM181 | 714.491917 | 0.41252985 | 0.03680335 | 0.19051526 |
| ROCK1 | 1861.64557 | 0.37234502 | 0.03682973 | 0.19059577 |
| LINC01061 | 88.4439088 | -0.594709 | 0.03686814 | 0.19068234 |
| PAK6 | 61.2862124 | -0.7173172 | 0.03686003 | 0.19068234 |
| ERCC2 | 332.822459 | -0.3924757 | 0.03689657 | 0.19071722 |
| ZFP69 | 50.8172771 | 0.42558282 | 0.03689515 | 0.19071722 |
| CLEC11A | 185.206213 | 0.93737812 | 0.03691552 | 0.19075909 |
| GMPS | 809.526626 | -0.3017212 | 0.03697839 | 0.19102786 |
| PSMB1 | 846.625229 | 0.35208183 | 0.03701873 | 0.1911801 |
| BTK | 131.450085 | 0.71371677 | 0.037132 | 0.19123878 |
| BTNL9 | 907.867462 | -0.8134216 | 0.03713366 | 0.19123878 |
| CXCR2P1 | 2.51818085 | -1.0480211 | 0.03710621 | 0.19123878 |
| HERC6 | 186.173094 | 0.53319163 | 0.03714968 | 0.19123878 |
| KRCC1 | 321.932735 | 0.39121851 | 0.03707059 | 0.19123878 |
| LINC01237 | 6.36216893 | 0.92653321 | 0.03714719 | 0.19123878 |
| LOC339874 | 14.2684885 | 0.84332256 | 0.03710977 | 0.19123878 |
| RNF212 | 43.306654 | -1.0696572 | 0.03713409 | 0.19123878 |
| SIVA1 | 209.895123 | 0.362111 | 0.03712267 | 0.19123878 |
| SNX14 | 890.427165 | 0.40532332 | 0.03704379 | 0.19123878 |
| ZNF22 | 243.491106 | 0.47514452 | 0.03713442 | 0.19123878 |
| GFAP | 596.65589 | -1.0109724 | 0.03717437 | 0.19130989 |
| C19orf57 | 13.9797064 | -0.8037207 | 0.03720539 | 0.19141352 |
| INPP5J | 182.827158 | -0.7480139 | 0.03724318 | 0.19155193 |
| ZNF573 | 179.756017 | 0.38081161 | 0.03735213 | 0.1920561 |
| HIST1H3F | 110.239916 | -0.8302768 | 0.03737853 | 0.19207954 |
| SRM | 497.103201 | -0.4507003 | 0.03737114 | 0.19207954 |
| PIK3R5 | 322.3986 | 0.71146085 | 0.0373921 | 0.19209319 |
| ATG16L2 | 159.175521 | 0.54948493 | 0.03743812 | 0.19215082 |
| IER5 | 376.722586 | 0.52953455 | 0.03743261 | 0.19215082 |
| ITFG3 | 1106.0578 | -0.3834216 | 0.03744702 | 0.19215082 |
| SIRPG | 3.80664972 | 1.04729959 | 0.03744177 | 0.19215082 |
| PLA2G5 | 1.95817395 | 1.06880519 | 0.03746105 | 0.19216676 |
| ACKR4 | 23.7134881 | -0.9897853 | 0.03747901 | 0.19220286 |
| C1QTNF7 | 30.349341 | 1.05469514 | 0.03751831 | 0.19234832 |
| ANXA9 | 88.6771802 | -0.7580708 | 0.0375694 | 0.19240781 |
| ARHGEF15 | 51.9787338 | 0.76188331 | 0.03756091 | 0.19240781 |
| FAM150B | 6.08745422 | -1.0467131 | 0.03757248 | 0.19240781 |
| PTPRO | 89.1636402 | 0.70936765 | 0.03757367 | 0.19240781 |
| HCFC1R1 | 583.945741 | -0.5355552 | 0.03759634 | 0.19246788 |
| FEM1C | 748.831244 | 0.42619522 | 0.03761736 | 0.19251943 |
| CHRNE | 18.3514471 | 0.90316351 | 0.03763541 | 0.19255579 |
| ZNF846 | 410.953147 | 0.47334424 | 0.03769629 | 0.19281123 |
| PLP2 | 621.771973 | -0.582347 | 0.03773032 | 0.1928731 |
| TMSB10 | 7790.92344 | -0.3952657 | 0.03772693 | 0.1928731 |
| AIM1L | 2.02697742 | -1.0600831 | 0.03778653 | 0.19310434 |
| CLSTN3 | 729.185031 | -0.4840554 | 0.03783897 | 0.19326 |
| SERTAD2 | 355.601423 | 0.55142799 | 0.03783866 | 0.19326 |
| SS18L2 | 50.9534287 | 0.39340747 | 0.03787512 | 0.19338849 |
| PPP1R9B | 1020.09716 | -0.25523 | 0.03791877 | 0.19352499 |
| YJEFN3 | 29.2529156 | -0.7356673 | 0.03792386 | 0.19352499 |

| | | | | |
|---|---|---|---|---|
| TTC9C | 204.818086 | -0.2733101 | 0.03795935 | 0.19364993 |
| FDFT1 | 803.864426 | -0.4272115 | 0.03799103 | 0.19375535 |
| NMUR1 | 4.93244048 | 0.94330295 | 0.03805226 | 0.1940114 |
| RAD1 | 349.894418 | 0.30649101 | 0.03806939 | 0.19404249 |
| ITPR2 | 3466.37347 | 0.5452155 | 0.03808153 | 0.1940481 |
| TNK1 | 79.4288315 | -0.6580009 | 0.03810819 | 0.19412771 |
| LINC00599 | 1.55282855 | -1.0577873 | 0.03814221 | 0.19416884 |
| MRPS34 | 336.529499 | -0.3992467 | 0.03816041 | 0.19416884 |
| MSL2 | 874.089892 | -0.2133978 | 0.03815718 | 0.19416884 |
| RNF2 | 374.478447 | -0.2774843 | 0.03813977 | 0.19416884 |
| ARL14EP | 290.188357 | 0.37644949 | 0.03821643 | 0.19439765 |
| COPS4 | 423.786684 | 0.2706866 | 0.03824397 | 0.19448146 |
| MIR612 | 2.45674369 | -0.9080476 | 0.03827489 | 0.19452623 |
| YBX3P1 | 5.35506924 | -0.8142088 | 0.03826718 | 0.19452623 |
| FBXO31 | 697.857603 | -0.3360166 | 0.03830031 | 0.1945744 |
| THOC1 | 320.554841 | 0.39239815 | 0.03830649 | 0.1945744 |
| IMPACT | 590.06858 | 0.49194096 | 0.03834241 | 0.19470061 |
| SP140 | 28.8293111 | 0.70622314 | 0.03836329 | 0.19475043 |
| DCUN1D3 | 298.803865 | 0.57278271 | 0.03839786 | 0.19475731 |
| PIGW | 132.346769 | -0.5054355 | 0.03839381 | 0.19475731 |
| TRAPPC2P1 | 49.5933534 | 0.44282468 | 0.0383965 | 0.19475731 |
| RNF133 | 19.1947795 | -0.8359396 | 0.03843268 | 0.19487774 |
| IER3IP1 | 321.978911 | 0.43551356 | 0.03848658 | 0.19503861 |
| SYNJ1 | 1035.02015 | -0.4114843 | 0.03848358 | 0.19503861 |
| CARNS1 | 13.8435122 | -1.0035415 | 0.03852773 | 0.1951347 |
| SPATS2 | 367.119111 | -0.3832695 | 0.03851907 | 0.1951347 |
| ANKRD32 | 221.90063 | 0.70377801 | 0.03855308 | 0.19515828 |
| ARID3B | 83.4701112 | 0.4956576 | 0.03855458 | 0.19515828 |
| LBH | 817.286238 | 0.67426455 | 0.0385707 | 0.19518374 |
| IPO5P1 | 357.097072 | 0.48987869 | 0.03865176 | 0.19553766 |
| MYH15 | 14.9963976 | 0.78931809 | 0.03866694 | 0.19555824 |
| ESD | 771.429757 | 0.27718481 | 0.03872909 | 0.19581625 |
| FILIP1L | 312.102809 | 0.69037911 | 0.03880387 | 0.19608159 |
| WDR81 | 913.633928 | 0.44946622 | 0.0387938 | 0.19608159 |
| ITPKC | 464.644348 | 0.39796189 | 0.03886754 | 0.19634692 |
| IKZF1 | 268.075962 | 0.68619227 | 0.03894701 | 0.19657901 |
| PTOV1 | 1064.04471 | -0.2548773 | 0.03894595 | 0.19657901 |
| TPST1 | 194.569479 | 0.44862241 | 0.03894432 | 0.19657901 |
| FSCN1 | 1450.77759 | -0.3541503 | 0.03902696 | 0.19686958 |
| KIAA1045 | 10.3077974 | 0.90566796 | 0.03901671 | 0.19686958 |
| PLD4 | 180.17944 | 0.89529838 | 0.0391135 | 0.19724954 |
| PRDM6 | 934.255136 | 0.70291276 | 0.03914116 | 0.19729782 |
| ZNF75A | 289.554624 | -0.314458 | 0.0391455 | 0.19729782 |
| HCK | 241.198002 | 0.67995718 | 0.03917665 | 0.19734172 |
| SECTM1 | 47.9671826 | 0.71605141 | 0.03917512 | 0.19734172 |
| CENPI | 40.7872867 | -0.7448694 | 0.03925037 | 0.19754336 |
| MAP7 | 163.304385 | 0.7283244 | 0.03924305 | 0.19754336 |
| PDLIM2 | 424.878626 | 0.61133338 | 0.0392445 | 0.19754336 |
| LOC1005071 | 13.0656524 | 0.82543001 | 0.03926428 | 0.19755684 |
| NIPSNAP1 | 511.285001 | -0.5467711 | 0.03929101 | 0.1976348 |

| | | | | |
|---|---|---|---|---|
| C16orf52 | 357.487707 | 0.28672478 | 0.0393548 | 0.19784251 |
| ZNF582 | 58.3818456 | 0.50919731 | 0.03934481 | 0.19784251 |
| B4GALNT1 | 50.5937149 | -0.9139429 | 0.03939568 | 0.19793777 |
| CPVL | 356.54044 | 0.50924156 | 0.03943001 | 0.19793777 |
| GUSBP3 | 29.9899236 | 0.61464598 | 0.03939732 | 0.19793777 |
| PLAG1 | 37.2834674 | 0.82914771 | 0.03940803 | 0.19793777 |
| SMIM2-AS1 | 4.72221536 | 0.81403739 | 0.039429 | 0.19793777 |
| ENTPD1 | 788.365754 | 0.63712021 | 0.03949189 | 0.19819181 |
| CHD7 | 859.377125 | -0.5207748 | 0.03956934 | 0.19835415 |
| LOC1019278 | 10.8324413 | 0.82280997 | 0.03955701 | 0.19835415 |
| LRRC8E | 62.9977379 | -0.7297815 | 0.03954781 | 0.19835415 |
| SUMF2 | 1021.69228 | 0.34354617 | 0.03955814 | 0.19835415 |
| PGS1 | 477.707496 | -0.3031228 | 0.03960566 | 0.19847966 |
| ZNF33BP1 | 29.6457532 | 0.57112056 | 0.03962364 | 0.19851321 |
| CTSV | 141.051292 | -1.0205301 | 0.03969436 | 0.19881087 |
| MACROD2 | 174.46293 | 0.81374412 | 0.03973679 | 0.19896675 |
| SLC7A9 | 4.340495 | 0.76699562 | 0.03976851 | 0.19906891 |
| PCDHGB7 | 828.664311 | -0.6677716 | 0.03978847 | 0.19911213 |
| MAP2K5 | 331.813871 | -0.3649646 | 0.03982417 | 0.19923411 |
| CNGA1 | 3.59416362 | 1.04017236 | 0.0398564 | 0.199282 |
| LRIG1 | 1098.66431 | -0.5192601 | 0.03985531 | 0.199282 |
| ZNF791 | 482.939797 | 0.35026138 | 0.03988667 | 0.1993767 |
| NBEAL1 | 1290.33003 | 0.20768103 | 0.03994994 | 0.19963622 |
| NTRK2 | 4854.92701 | 0.92153875 | 0.04002171 | 0.19993805 |
| LRRC40 | 192.248117 | 0.36763715 | 0.04007432 | 0.20010685 |
| VWA5B2 | 3.02787087 | -1.0244629 | 0.04007825 | 0.20010685 |
| SPATC1L | 61.8216254 | -0.9630099 | 0.0401054 | 0.20018558 |
| GPATCH2L | 1431.17892 | 0.32911161 | 0.04011779 | 0.20019061 |
| CDC25C | 10.7524333 | -0.9018626 | 0.04013855 | 0.20023741 |
| COL25A1 | 31.389379 | 0.86566817 | 0.04018926 | 0.20031852 |
| GAS2L1 | 189.505871 | -0.4316743 | 0.0401669 | 0.20031852 |
| IGSF1 | 37.4910466 | -1.0130506 | 0.04020036 | 0.20031852 |
| MCTP2 | 203.838357 | -0.8551086 | 0.04019408 | 0.20031852 |
| TMEM80 | 164.370572 | 0.5301325 | 0.04022003 | 0.20035974 |
| PHF1 | 43.7094103 | 0.45140741 | 0.04026673 | 0.20053559 |
| ENOX2 | 141.870457 | 0.35848448 | 0.04029953 | 0.20058539 |
| LINC00189 | 67.1711399 | -0.8634809 | 0.04028992 | 0.20058539 |
| HYOU1 | 2663.96954 | -0.5076867 | 0.04036031 | 0.20077427 |
| OSBPL7 | 294.598689 | -0.3213228 | 0.0403545 | 0.20077427 |
| CLGN | 9.74773827 | -0.97356 | 0.04043858 | 0.20110678 |
| LINC01176 | 22.2705244 | 0.72249315 | 0.04047888 | 0.20121876 |
| METTL15 | 238.558946 | 0.33192088 | 0.04049542 | 0.20121876 |
| RIOK2 | 351.833665 | 0.33856949 | 0.04049067 | 0.20121876 |
| ZDHHC16 | 337.858745 | -0.3707761 | 0.04050701 | 0.20121952 |
| MAFF | 198.67175 | 0.94222981 | 0.04053002 | 0.20127696 |
| STAG3L4 | 215.032187 | -0.3435816 | 0.04054331 | 0.20128617 |
| ST6GALNAC6 | 908.089249 | 0.41437248 | 0.04058517 | 0.20143713 |
| YWHAG | 2649.87682 | -0.3276345 | 0.04063199 | 0.20161259 |
| SCARF2 | 469.177505 | -0.6313644 | 0.04068933 | 0.20184021 |
| ATP1B2 | 213.686419 | -0.7671026 | 0.04075684 | 0.20194727 |

| | | | | |
|---|---|---|---|---|
| C14orf39 | 66.2607985 | 0.92332212 | 0.04073672 | 0.20194727 |
| UBE2M | 568.692666 | -0.4767132 | 0.04074742 | 0.20194727 |
| VAC14 | 490.723425 | -0.3166752 | 0.04074943 | 0.20194727 |
| OLFML1 | 1548.569 | 0.72077527 | 0.04078123 | 0.20201121 |
| RRP8 | 181.778485 | 0.30816205 | 0.04080935 | 0.20209361 |
| ANXA2P2 | 84.9055383 | -0.6285371 | 0.04090243 | 0.20210465 |
| ARHGAP25 | 131.16387 | 0.62811384 | 0.0409035 | 0.20210465 |
| GZMK | 14.6854968 | 0.92189802 | 0.04085517 | 0.20210465 |
| KCNIP2-AS1 | 1.34402505 | 1.01314214 | 0.04084699 | 0.20210465 |
| MED30 | 84.2780918 | 0.46202261 | 0.04086984 | 0.20210465 |
| MEPCE | 719.146861 | -0.3136554 | 0.04084888 | 0.20210465 |
| NPIPA5 | 64.8092228 | -0.6445554 | 0.04086586 | 0.20210465 |
| SNHG1 | 384.596973 | -0.4159636 | 0.04090291 | 0.20210465 |
| CBX5 | 6673.13845 | -0.2688486 | 0.04097912 | 0.20238334 |
| CEP44 | 409.724237 | 0.311872 | 0.04102894 | 0.20238334 |
| IGSF11 | 103.806915 | -0.9194818 | 0.04102406 | 0.20238334 |
| MCCC2 | 830.790789 | -0.2670939 | 0.04101354 | 0.20238334 |
| NFIB | 2664.01937 | -0.5463586 | 0.04099494 | 0.20238334 |
| PGLS | 419.364404 | -0.2572831 | 0.04102049 | 0.20238334 |
| GNB4 | 1340.92935 | 0.62480642 | 0.04104207 | 0.20239136 |
| SHARPIN | 445.56554 | -0.3142374 | 0.04105789 | 0.20241262 |
| DIXDC1 | 1849.4715 | -0.5914427 | 0.04113976 | 0.20271727 |
| SLC19A3 | 2.71893955 | -1.0000729 | 0.04114273 | 0.20271727 |
| C6orf147 | 13.8344512 | -1.0039196 | 0.04118012 | 0.20283998 |
| CPA2 | 1.76519866 | -1.0415679 | 0.0411907 | 0.20283998 |
| RRAGB | 371.223174 | 0.22865023 | 0.04121968 | 0.20292589 |
| SLC35D2 | 289.893847 | 0.26303198 | 0.04125626 | 0.20301517 |
| TNFSF4 | 49.9102876 | 0.77561469 | 0.0412609 | 0.20301517 |
| LOC1005055 | 66.3744876 | 0.58237889 | 0.04127977 | 0.2030512 |
| C10orf128 | 27.1587372 | 0.69857167 | 0.04131336 | 0.20314094 |
| IRAK1BP1 | 145.702279 | 0.65000922 | 0.04132111 | 0.20314094 |
| SPIN1 | 2629.13408 | -0.2163335 | 0.04133799 | 0.20316715 |
| C2CD2L | 286.882481 | -0.3918371 | 0.04136041 | 0.20322056 |
| ZDHHC13 | 151.142284 | -0.5124243 | 0.04138483 | 0.20328379 |
| NEURL1 | 9.49779151 | -0.884286 | 0.04142052 | 0.20329465 |
| PIGM | 311.679059 | -0.3825376 | 0.04140083 | 0.20329465 |
| RNU12 | 61.1105406 | 0.4717217 | 0.04142172 | 0.20329465 |
| LOC1019295 | 3.43618835 | 0.94106362 | 0.04147629 | 0.20350571 |
| GAP43 | 387.233541 | -1.0023808 | 0.04154209 | 0.20373755 |
| PCDHB17 | 42.7006587 | -0.8695782 | 0.04154671 | 0.20373755 |
| ENPP2 | 437.1916 | 0.80549955 | 0.04155954 | 0.20374367 |
| SPAG9 | 6670.43394 | 0.32424099 | 0.04158114 | 0.20374593 |
| TAS2R5 | 67.8346287 | 0.56184709 | 0.04158317 | 0.20374593 |
| PXK | 257.618784 | 0.47434785 | 0.04163931 | 0.20396419 |
| HLA-DRB1 | 1622.18773 | 0.9562411 | 0.04165444 | 0.20398149 |
| COQ10A | 101.097408 | -0.4877027 | 0.04172046 | 0.20419114 |
| RPS8 | 6291.98911 | 0.43631857 | 0.04171402 | 0.20419114 |
| SNIP1 | 217.509164 | 0.4650503 | 0.04178565 | 0.20445331 |
| FAM58A | 119.48502 | -0.3122001 | 0.04189396 | 0.20492624 |
| CAMKV | 1.91865453 | -1.0422069 | 0.04195192 | 0.2051445 |

| | | | | |
|---|---|---|---|---|
| CST6 | 1.56131206 | 1.04288881 | 0.04197018 | 0.2051445 |
| RPL8 | 7907.55269 | -0.4688765 | 0.04197357 | 0.2051445 |
| FOXO3 | 2463.10359 | 0.65852541 | 0.04199737 | 0.20514681 |
| SNHG3 | 244.410045 | 0.40709368 | 0.04199188 | 0.20514681 |
| RHBDL1 | 23.4233454 | -0.6518578 | 0.04202516 | 0.20516862 |
| SS18L1 | 307.355 | -0.4197417 | 0.04201959 | 0.20516862 |
| C3orf70 | 237.197103 | 0.72376125 | 0.04207925 | 0.20531875 |
| PDCD6IPP2 | 33.600459 | 0.86187816 | 0.04207802 | 0.20531875 |
| LAIR1 | 544.05771 | 0.64573464 | 0.04210025 | 0.2053642 |
| FAM214A | 1044.65201 | -0.2808872 | 0.04215568 | 0.20557758 |
| WDR93 | 3.8038987 | -0.9305324 | 0.04225398 | 0.20594278 |
| ZNF8 | 343.191305 | -0.2679799 | 0.04224626 | 0.20594278 |
| DOK1 | 245.815661 | 0.41301397 | 0.04228109 | 0.20601782 |
| KDM5A | 2756.31688 | 0.36722296 | 0.04231398 | 0.206121 |
| BCKDHA | 432.187531 | -0.3512416 | 0.04234712 | 0.20622533 |
| ALK | 7.84815225 | -0.8491448 | 0.04237585 | 0.2063081 |
| TTC29 | 14.2544956 | -1.0429386 | 0.04239934 | 0.20632656 |
| ZNF292 | 1858.71895 | 0.35315931 | 0.0424031 | 0.20632656 |
| NAE1 | 460.049594 | 0.28573232 | 0.04244405 | 0.20641163 |
| RPS6KA2 | 1447.6195 | -0.664211 | 0.04243671 | 0.20641163 |
| NME6 | 92.6751731 | 0.35708107 | 0.04246225 | 0.20644305 |
| CRYGS | 13.7783077 | 0.64810003 | 0.0424931 | 0.20647888 |
| DNAJC27-AS | 54.5688105 | 0.68770842 | 0.04248382 | 0.20647888 |
| OLFM2 | 158.255725 | 0.82617685 | 0.04252311 | 0.20649918 |
| RPL23AP82 | 48.6288699 | 0.64435581 | 0.04254423 | 0.20649918 |
| ZNF414 | 94.8556422 | -0.5248648 | 0.04254266 | 0.20649918 |
| ZNF587B | 470.60024 | -0.3094507 | 0.04253275 | 0.20649918 |
| IRF2BP1 | 320.537326 | -0.2931055 | 0.04257198 | 0.20657685 |
| C11orf68 | 379.283272 | -0.2902473 | 0.04264097 | 0.20685459 |
| PDGFB | 324.25684 | 0.54894491 | 0.04272317 | 0.20719617 |
| LINC00482 | 18.1721238 | -0.967833 | 0.04274219 | 0.2072313 |
| FBRS | 618.303686 | -0.2764516 | 0.04279126 | 0.207296 |
| PELI2 | 134.152302 | 0.67930866 | 0.04279734 | 0.207296 |
| RGPD4 | 19.0637381 | 0.62770938 | 0.04281526 | 0.207296 |
| TM4SF1 | 693.847339 | 0.6757152 | 0.04281588 | 0.207296 |
| TRDMT1 | 277.450399 | 0.4013929 | 0.04277946 | 0.207296 |
| ZNF222 | 54.7680363 | 0.37285129 | 0.04282625 | 0.207296 |
| VTI1A | 537.307934 | 0.33909544 | 0.04283827 | 0.20729714 |
| BCL10 | 191.515632 | 0.41580355 | 0.04291929 | 0.20761552 |
| CYP2B7P | 5.21775599 | 0.95281129 | 0.04295102 | 0.20761552 |
| LOC1005061 | 1.77060596 | -1.0319549 | 0.04295426 | 0.20761552 |
| PCDH19 | 545.381495 | -0.9712846 | 0.04293063 | 0.20761552 |
| XRCC6 | 1794.1729 | 0.34222994 | 0.04296308 | 0.20761552 |
| ANXA7 | 1215.37512 | 0.30667641 | 0.04301878 | 0.20781073 |
| YRDC | 67.4619215 | 0.46988362 | 0.0430271 | 0.20781073 |
| POF1B | 9.32205757 | -1.0177637 | 0.04304566 | 0.20784329 |
| COX6B1 | 1145.27526 | -0.3692732 | 0.04307881 | 0.20794627 |
| NCF4 | 121.669426 | 0.61670529 | 0.04309441 | 0.20796452 |
| KLHDC2 | 618.646094 | 0.49046854 | 0.04315784 | 0.20821351 |
| DDX28 | 103.015097 | -0.3375738 | 0.04320852 | 0.20823971 |

| | | | | |
|---|---|---|---|---|
| MBD2 | 665.847739 | 0.51780114 | 0.04319661 | 0.20823971 |
| PCIF1 | 621.498508 | -0.3522432 | 0.04317835 | 0.20823971 |
| RAVER1 | 563.945441 | -0.3451771 | 0.04321063 | 0.20823971 |
| MAP3K7 | 1012.58601 | 0.34235278 | 0.04325808 | 0.20829719 |
| MATR3 | 5508.29544 | 0.2033866 | 0.04323612 | 0.20829719 |
| VPS4B | 769.83924 | 0.39569924 | 0.04325379 | 0.20829719 |
| HLA-DOA | 31.9452772 | 0.88927866 | 0.0432997 | 0.20844052 |
| HSF1 | 617.51626 | -0.3031775 | 0.04333377 | 0.20854747 |
| ZNF205-AS1 | 5.39184198 | 0.7851226 | 0.0433661 | 0.20864598 |
| C7orf55 | 47.0653205 | 0.4655182 | 0.04338434 | 0.20867668 |
| HHATL | 1.9639756 | -0.9931431 | 0.04339839 | 0.20868717 |
| LILRB5 | 50.2269842 | -1.0183371 | 0.04341539 | 0.20871185 |
| ZNF271 | 574.6136 | 0.34172032 | 0.04343396 | 0.2087441 |
| MTHFD1 | 694.888271 | -0.3621843 | 0.04349132 | 0.20879157 |
| PRIMPOL | 204.343615 | 0.32835354 | 0.04347132 | 0.20879157 |
| PRRT2 | 180.069009 | 0.77517286 | 0.04347975 | 0.20879157 |
| SON | 12477.3485 | -0.2604097 | 0.0434891 | 0.20879157 |
| POLE | 1266.15931 | -0.4085184 | 0.04356689 | 0.20909729 |
| RBM27 | 1025.68673 | 0.20854862 | 0.04359178 | 0.20915969 |
| EXD2 | 398.594819 | 0.44345658 | 0.04363915 | 0.20921601 |
| LIMCH1 | 1267.57674 | 0.59295778 | 0.0436392 | 0.20921601 |
| MAP7D3 | 286.47035 | 0.74819064 | 0.04362398 | 0.20921601 |
| CHMP4C | 63.8139986 | -1.0066276 | 0.04371303 | 0.20951288 |
| ITGA1 | 2205.04749 | 0.64475276 | 0.04375743 | 0.20966851 |
| ZSCAN26 | 300.803467 | 0.41084098 | 0.04381255 | 0.20987547 |
| CLDN23 | 4.42171329 | 0.82323064 | 0.04393858 | 0.21042041 |
| GDNF-AS1 | 1.40266401 | 1.01111075 | 0.04396118 | 0.21042041 |
| ISOC2 | 519.119862 | -0.3468724 | 0.04397416 | 0.21042041 |
| ZNF622 | 292.616657 | 0.38128575 | 0.04397192 | 0.21042041 |
| ADAMTSL3 | 5447.03969 | 0.78846594 | 0.04416914 | 0.21051511 |
| C16orf80 | 215.212012 | -0.3064481 | 0.04409171 | 0.21051511 |
| CAPN11 | 6.64521919 | 0.8665089 | 0.04410608 | 0.21051511 |
| EVPL | 4.83653382 | -0.9467417 | 0.04419402 | 0.21051511 |
| GALK2 | 364.236227 | 0.25661731 | 0.04409841 | 0.21051511 |
| GPR137 | 309.852347 | -0.362165 | 0.04412314 | 0.21051511 |
| HSBP1L1 | 93.5748549 | -0.5426809 | 0.04413983 | 0.21051511 |
| ISLR | 11026.8832 | -0.8432679 | 0.04410099 | 0.21051511 |
| LINC01140 | 4.54774345 | 0.74996337 | 0.04410463 | 0.21051511 |
| MMP28 | 191.346119 | 0.9214464 | 0.0441915 | 0.21051511 |
| NECAP2 | 504.457123 | 0.35087401 | 0.04403756 | 0.21051511 |
| NXN | 726.420093 | -0.6853449 | 0.04419857 | 0.21051511 |
| SHB | 702.610497 | -0.5887186 | 0.04409909 | 0.21051511 |
| SLC5A9 | 4.33198825 | 0.89745897 | 0.0441774 | 0.21051511 |
| SLC8A3 | 8.46459038 | 0.95549211 | 0.04420937 | 0.21051511 |
| UCKL1 | 398.43627 | -0.4079554 | 0.04414745 | 0.21051511 |
| ZCCHC4 | 140.785402 | 0.34790691 | 0.04409777 | 0.21051511 |
| ZFP36L2 | 3511.65583 | -0.4263456 | 0.04406199 | 0.21051511 |
| LACTB | 235.425954 | 0.45801418 | 0.04425843 | 0.21063469 |
| PCGF6 | 92.7532585 | 0.40135475 | 0.04425564 | 0.21063469 |
| GMPPA | 269.818778 | -0.3619197 | 0.04427888 | 0.21067502 |

| | | | | |
|---|---|---|---|---|
| DDX3X | 5586.63776 | 0.27846904 | 0.0443307 | 0.21080751 |
| LOC1022884 | 28.0537633 | -0.4464259 | 0.04431982 | 0.21080751 |
| MAPKAPK5-A | 139.468152 | 0.29306992 | 0.04440658 | 0.21111129 |
| SCRN3 | 402.651671 | -0.2367615 | 0.04448727 | 0.21143775 |
| ZNF878 | 3.80421563 | -0.8641188 | 0.04452292 | 0.21155004 |
| BCS1L | 229.18372 | -0.3163686 | 0.04460536 | 0.21159878 |
| DMC1 | 7.81423093 | 0.82957003 | 0.04457524 | 0.21159878 |
| KLK11 | 3.4260298 | -0.9420673 | 0.04459718 | 0.21159878 |
| LHX2 | 2.95436843 | -1.0326227 | 0.04454612 | 0.21159878 |
| MORC4 | 463.900728 | 0.43857299 | 0.04459525 | 0.21159878 |
| RBMS3 | 777.861994 | 0.87146955 | 0.04457922 | 0.21159878 |
| RFX1 | 203.999605 | -0.3420383 | 0.0448212 | 0.21256536 |
| VPS37B | 307.46994 | -0.243501 | 0.04489548 | 0.21286022 |
| DFNB31 | 178.989428 | -0.6071747 | 0.04495322 | 0.21295446 |
| MS4A6A | 797.819861 | 0.59865427 | 0.04495658 | 0.21295446 |
| PIGF | 153.03589 | 0.39190408 | 0.04496228 | 0.21295446 |
| RAB6A | 2210.79411 | -0.2169076 | 0.04496378 | 0.21295446 |
| NEK1 | 661.113544 | 0.31541937 | 0.0450349 | 0.21317648 |
| STXBP5-AS1 | 8.59625568 | 0.82712789 | 0.04503149 | 0.21317648 |
| UBE2O | 733.135037 | -0.2750967 | 0.04505869 | 0.21323171 |
| EMILIN1 | 932.739322 | 0.74700912 | 0.04519789 | 0.2134823 |
| KPNB1 | 3605.04605 | -0.2419704 | 0.0451669 | 0.2134823 |
| LINC00941 | 4.60473977 | -1.0052366 | 0.04519428 | 0.2134823 |
| LINC01279 | 393.612357 | 0.76172199 | 0.04514068 | 0.2134823 |
| PDHX | 610.689454 | -0.2862234 | 0.04517285 | 0.2134823 |
| PML | 1018.16551 | -0.3380339 | 0.04520873 | 0.2134823 |
| TRPM6 | 15.439791 | -0.95054 | 0.04512499 | 0.2134823 |
| ZNRF2P1 | 36.1002016 | -0.6011609 | 0.04515547 | 0.2134823 |
| DPY19L2P3 | 5.23384281 | 0.73528092 | 0.04523875 | 0.2135667 |
| IFT88 | 280.896526 | 0.44174581 | 0.04528123 | 0.2136576 |
| PRLR | 44.8075172 | 0.81384962 | 0.0452881 | 0.2136576 |
| TUBB3 | 125.840134 | -0.9400211 | 0.04529444 | 0.2136576 |
| ATP6AP1 | 1700.68728 | -0.2621404 | 0.04535325 | 0.21370579 |
| GOLGA6L9 | 18.8393414 | -0.707982 | 0.04532069 | 0.21370579 |
| PYGL | 691.800866 | 0.4745546 | 0.04534195 | 0.21370579 |
| ZNF784 | 70.7511118 | -0.5350495 | 0.04535229 | 0.21370579 |
| FSCN2 | 26.111017 | -0.7951798 | 0.04537721 | 0.21376144 |
| ARNT2 | 6606.36038 | -0.6353784 | 0.0454451 | 0.21399453 |
| PER3 | 1039.8286 | 0.56876497 | 0.04546319 | 0.21399453 |
| SNTG1 | 3.40655971 | -1.0257086 | 0.0454526 | 0.21399453 |
| HLTF | 1444.1811 | -0.318955 | 0.04549794 | 0.21402003 |
| ITGA3 | 754.942283 | -0.6784944 | 0.04550181 | 0.21402003 |
| OSGIN1 | 21.2311302 | 0.83587263 | 0.04551728 | 0.21402003 |
| PRKCE | 535.322573 | -0.4105464 | 0.04550639 | 0.21402003 |
| LOC1005075 | 5.57855028 | 0.73475395 | 0.04554435 | 0.21403289 |
| SNORA55 | 1.80055144 | 0.92080974 | 0.0455373 | 0.21403289 |
| DLG1 | 1418.6169 | -0.2107388 | 0.04557869 | 0.2140799 |
| LUZP2 | 31.5954281 | 1.02778328 | 0.0455685 | 0.2140799 |
| PRKD2 | 501.085234 | -0.3608381 | 0.04566177 | 0.21439387 |
| TRIP4 | 285.541522 | -0.2947488 | 0.04566992 | 0.21439387 |

| | | | | |
|---|---|---|---|---|
| KCTD20 | 1626.78781 | 0.34176898 | 0.04570901 | 0.21452015 |
| FAF1 | 545.827185 | 0.25128399 | 0.04573233 | 0.21457224 |
| SPNS1 | 372.204708 | -0.321728 | 0.04574451 | 0.21457224 |
| ARFGAP2 | 870.988287 | 0.28147645 | 0.04579295 | 0.21469311 |
| GPR183 | 158.068776 | 0.78908363 | 0.04579469 | 0.21469311 |
| HELLS | 200.736013 | -0.6255577 | 0.04585236 | 0.21490623 |
| DDX5 | 9955.5466 | -0.2852117 | 0.04587844 | 0.21491392 |
| ZNF397 | 366.826978 | 0.36977787 | 0.04587321 | 0.21491392 |
| LINC00173 | 31.9871132 | -0.857067 | 0.04595208 | 0.2151443 |
| RPL28 | 4346.07911 | -0.4790981 | 0.04594424 | 0.2151443 |
| CENPW | 25.0119182 | 0.71874682 | 0.04597999 | 0.21521767 |
| EXOSC4 | 113.878132 | -0.3717818 | 0.04600839 | 0.21523605 |
| LOC1019269 | 2.94679815 | 0.89971608 | 0.04599634 | 0.21523605 |
| BRSK1 | 165.144273 | -0.8074301 | 0.04603028 | 0.21528123 |
| MEA1 | 358.406587 | -0.3472748 | 0.04607662 | 0.21544065 |
| TNFRSF4 | 6.2607815 | 0.85181293 | 0.046102 | 0.21550202 |
| IGBP1 | 583.714652 | 0.32788079 | 0.04613159 | 0.21558305 |
| GOLGA1 | 591.458322 | -0.2394096 | 0.04617456 | 0.21566929 |
| TTC23L | 8.23212658 | 0.74729652 | 0.04616613 | 0.21566929 |
| ADNP | 2921.36577 | -0.3167354 | 0.04619332 | 0.21567211 |
| ASCC3 | 1697.04326 | 0.38938361 | 0.04623139 | 0.21567211 |
| FUCA1 | 425.359176 | 0.45605254 | 0.04622268 | 0.21567211 |
| GRIN2C | 8.19786901 | -0.7793268 | 0.04623585 | 0.21567211 |
| NPAT | 739.568078 | -0.1844768 | 0.04623647 | 0.21567211 |
| IP6K3 | 15.8518673 | -1.0217187 | 0.04626553 | 0.21571445 |
| LOC1019290 | 1.90028426 | -0.9928936 | 0.04627007 | 0.21571445 |
| FTO-IT1 | 30.5931919 | 0.59775614 | 0.04631816 | 0.21583341 |
| GPR37 | 12.638798 | -1.0219202 | 0.04632013 | 0.21583341 |
| NCR3LG1 | 845.481615 | -0.7935202 | 0.04633975 | 0.21586763 |
| CSF3R | 328.933752 | 0.71072675 | 0.04639227 | 0.21590422 |
| IKZF2 | 483.87179 | 0.43058946 | 0.04638909 | 0.21590422 |
| SELL | 68.0373487 | 0.66555565 | 0.04638915 | 0.21590422 |
| TMBIM4 | 906.587839 | 0.26490852 | 0.0463967 | 0.21590422 |
| PDSS2 | 272.022296 | 0.41004434 | 0.04641555 | 0.21593483 |
| HDAC1 | 425.837296 | 0.43461069 | 0.04649275 | 0.21617942 |
| LOC145783 | 141.879239 | -0.3930438 | 0.04652958 | 0.21617942 |
| LOC150776 | 417.295308 | -0.4847739 | 0.04651262 | 0.21617942 |
| NDUFS4 | 432.393813 | 0.327406 | 0.04651892 | 0.21617942 |
| SLC6A8 | 886.459679 | 0.608376 | 0.04650606 | 0.21617942 |
| ARHGAP5-AS | 31.7635397 | -0.6668815 | 0.04657828 | 0.21629145 |
| JUN | 3377.2183 | 0.57397501 | 0.04657491 | 0.21629145 |
| EIF3I | 864.261886 | 0.34717708 | 0.04661809 | 0.21639476 |
| FGD5P1 | 51.2515192 | -0.9000457 | 0.04662514 | 0.21639476 |
| LOC613037 | 27.6474392 | 0.63144798 | 0.04664044 | 0.21640871 |
| ATXN7L2 | 63.0591328 | 0.49419028 | 0.04671011 | 0.2166748 |
| C7orf63 | 69.4042251 | 0.49853293 | 0.04677224 | 0.21667712 |
| CIRBP-AS1 | 3.24086463 | -0.9963427 | 0.04678452 | 0.21667712 |
| ITGB2-AS1 | 55.447562 | 0.88170657 | 0.04676783 | 0.21667712 |
| SMYD3 | 103.401361 | -0.325786 | 0.04676453 | 0.21667712 |
| WDR4 | 192.995292 | -0.4179098 | 0.04678109 | 0.21667712 |

| | | | | |
|---|---|---|---|---|
| ZNF536 | 277.223014 | -0.7974539 | 0.04677311 | 0.21667712 |
| ASAP3 | 997.592836 | 0.54079254 | 0.04684256 | 0.21682189 |
| GPSM3 | 3.14511034 | -0.9617427 | 0.04684403 | 0.21682189 |
| PRDM8 | 7.12214672 | -0.9286355 | 0.04685276 | 0.21682189 |
| NFE2L3 | 73.1762727 | 0.69361853 | 0.04689738 | 0.21697129 |
| MTM1 | 229.697679 | 0.3481853 | 0.04697681 | 0.21728163 |
| SEPT7 | 2805.7521 | 0.3600778 | 0.04701283 | 0.21739106 |
| HHIPL2 | 5.97905307 | -0.959706 | 0.04706797 | 0.21758886 |
| ATL1 | 414.577594 | 0.6002632 | 0.04709602 | 0.21766134 |
| FRMD6-AS1 | 15.2515923 | 0.67525628 | 0.04713544 | 0.2177863 |
| GPRC5A | 331.170851 | -0.93567 | 0.04722019 | 0.21806361 |
| RAB11FIP1 | 1417.41966 | -0.565045 | 0.04722026 | 0.21806361 |
| NME1 | 298.069925 | -0.451558 | 0.04724767 | 0.21813292 |
| NBAS | 1796.7949 | 0.21042788 | 0.04733221 | 0.21844522 |
| ZSCAN12 | 373.206612 | -0.3373372 | 0.04734015 | 0.21844522 |
| LOC283440 | 2.00503247 | 0.98494154 | 0.04735591 | 0.21846065 |
| CXorf21 | 35.8754237 | 0.83243777 | 0.04739015 | 0.218504 |
| MBD1 | 743.948741 | 0.33000856 | 0.0473789 | 0.218504 |
| CHTF18 | 166.409093 | -0.4504592 | 0.04743414 | 0.21853497 |
| CNBP | 3084.20191 | 0.16511098 | 0.04741414 | 0.21853497 |
| GRM5 | 1.73741911 | -1.0124736 | 0.04742654 | 0.21853497 |
| CIR1 | 422.491165 | -0.1610436 | 0.04746232 | 0.21860753 |
| GALK1 | 149.7359 | -0.472466 | 0.0475043 | 0.21874364 |
| LYSMD3 | 538.825892 | 0.32809521 | 0.04752637 | 0.21878797 |
| LOC1005062 | 3.95578964 | 0.82833583 | 0.04755198 | 0.2188486 |
| CPD | 5665.75007 | -0.4528361 | 0.04764911 | 0.21891593 |
| JMJD1C-AS1 | 4.31097766 | -0.9458422 | 0.04761461 | 0.21891593 |
| MRPS28 | 125.348704 | 0.40325053 | 0.04764471 | 0.21891593 |
| PCBP2 | 6036.42724 | -0.4458549 | 0.04765373 | 0.21891593 |
| RAB40A | 7.71247537 | 0.69074169 | 0.04759325 | 0.21891593 |
| SH2D1A | 6.94914472 | 0.95079718 | 0.04761893 | 0.21891593 |
| TMEM17 | 39.9272047 | 0.47607458 | 0.04758095 | 0.21891593 |
| ALDH1L1-AS | 1.83065457 | 0.99731283 | 0.04767256 | 0.21894524 |
| UFL1 | 917.56176 | 0.38274036 | 0.04773609 | 0.21917978 |
| LIPA | 1201.48735 | 0.41393458 | 0.04777788 | 0.21931444 |
| ZNF490 | 428.69535 | 0.37253456 | 0.04781191 | 0.2194134 |
| SETBP1 | 3703.37648 | 0.51479741 | 0.04784088 | 0.21948905 |
| FOS | 5726.33899 | 0.77888962 | 0.04786561 | 0.21953335 |
| MAPKAP1 | 1082.90253 | -0.1836547 | 0.04787549 | 0.21953335 |
| RAD18 | 284.097956 | -0.3730212 | 0.04789004 | 0.21954282 |
| ARF1 | 4190.72588 | -0.2910688 | 0.04798816 | 0.2197895 |
| GPSM2 | 168.259662 | -0.7626666 | 0.04800633 | 0.2197895 |
| KLF7 | 1096.84387 | 0.36031196 | 0.04797823 | 0.2197895 |
| LOC729080 | 1.72827439 | -0.9592287 | 0.04799568 | 0.2197895 |
| SUN2 | 1007.96732 | 0.32687062 | 0.04797659 | 0.2197895 |
| CCKBR | 9.67967203 | -0.9182671 | 0.04802678 | 0.21980849 |
| IFNGR2 | 762.589131 | 0.30455368 | 0.0480588 | 0.21980849 |
| LY86 | 94.289478 | 0.704098 | 0.04805307 | 0.21980849 |
| RAB3IL1 | 1735.79462 | -0.3911935 | 0.04806046 | 0.21980849 |
| EPHA6 | 6.73039943 | -0.9937098 | 0.04809386 | 0.2199041 |

| | | | | |
|---|---|---|---|---|
| CCDC102B | 83.9158255 | 0.51025947 | 0.0481114 | 0.21992709 |
| EPCAM | 3.33462608 | -0.9643816 | 0.04813969 | 0.21994211 |
| STX7 | 1748.95473 | 0.35569387 | 0.04813612 | 0.21994211 |
| NCF2 | 270.283234 | 0.64128756 | 0.04815735 | 0.21996565 |
| MIR143HG | 36.2687276 | 0.83335697 | 0.04817615 | 0.21999442 |
| IFNAR2 | 395.422094 | -0.4324244 | 0.04825293 | 0.22028783 |
| TMEM240 | 12.1843445 | 0.73494358 | 0.04827766 | 0.22034356 |
| ULBP1 | 3.99654147 | 0.98987992 | 0.04831527 | 0.22045802 |
| BHMT2 | 518.324573 | 0.88581764 | 0.04840261 | 0.22063545 |
| CD37 | 128.021885 | 0.68381859 | 0.04839246 | 0.22063545 |
| DCX | 11.6763571 | -0.9408048 | 0.04840433 | 0.22063545 |
| GTF3C3 | 571.329414 | -0.2155252 | 0.04838523 | 0.22063545 |
| PEX6 | 379.816227 | 0.48720553 | 0.04845375 | 0.2207463 |
| TMEM214 | 1005.40394 | -0.3532856 | 0.04845132 | 0.2207463 |
| GOLGA2 | 1478.14733 | -0.2128786 | 0.04866241 | 0.2216395 |
| NDUFB3 | 343.656523 | -0.3216803 | 0.04870149 | 0.22176011 |
| C1orf95 | 12.6619062 | 0.77157532 | 0.04872637 | 0.22181597 |
| ACTR3C | 24.317666 | -0.5574402 | 0.04879771 | 0.22196837 |
| DOK5 | 144.191196 | 0.92467302 | 0.04879734 | 0.22196837 |
| PPP1R15A | 948.27234 | 0.59429631 | 0.0487896 | 0.22196837 |
| NOC3L | 399.707372 | 0.36904138 | 0.04883893 | 0.22209843 |
| DCDC5 | 16.1480911 | -0.90228 | 0.0488961 | 0.22223846 |
| FOXP3 | 3.15058091 | -0.9182525 | 0.04892205 | 0.22223846 |
| PKP2 | 3114.22944 | 0.75176682 | 0.04891793 | 0.22223846 |
| PTPN9 | 1207.77378 | -0.429032 | 0.04893289 | 0.22223846 |
| SNTB1 | 113.104982 | 0.71476926 | 0.04893276 | 0.22223846 |
| ABI3BP | 3918.98917 | 0.86310392 | 0.04900288 | 0.22246234 |
| CABIN1 | 1050.49439 | 0.27577317 | 0.04900748 | 0.22246234 |
| PABPC1L | 293.960745 | -0.613497 | 0.04903231 | 0.22251761 |
| RARS2 | 424.984279 | 0.34767012 | 0.04910318 | 0.22266744 |
| STT3A | 2062.76328 | -0.258614 | 0.04910012 | 0.22266744 |
| VAX2 | 11.1148561 | -1.002219 | 0.0491033 | 0.22266744 |
| IGDCC4 | 183.363909 | -0.8332516 | 0.04916216 | 0.22281949 |
| PIP5KL1 | 8.41753081 | -0.8147513 | 0.04915439 | 0.22281949 |
| MYO19 | 575.665715 | -0.46368 | 0.04921588 | 0.22300551 |
| SLC22A3 | 799.255822 | -0.7480609 | 0.04928026 | 0.2232397 |
| INTS4 | 356.797903 | -0.26577 | 0.04930172 | 0.22327939 |
| CBX7 | 410.597246 | 0.45014873 | 0.04937666 | 0.22348935 |
| LOC1001290 | 1335.36594 | -0.439945 | 0.0494224 | 0.22348935 |
| PRRC2B | 6647.81993 | -0.2518607 | 0.04937239 | 0.22348935 |
| PWWP2A | 469.696296 | 0.33478518 | 0.049423 | 0.22348935 |
| RDH16 | 3.79180445 | 0.88665047 | 0.04943701 | 0.22348935 |
| RFX2 | 153.027609 | 0.78879003 | 0.04943309 | 0.22348935 |
| SURF1 | 298.154738 | -0.2599644 | 0.04942142 | 0.22348935 |
| ARHGAP30 | 355.532427 | 0.62526562 | 0.04951148 | 0.22376846 |
| HRASLS5 | 42.8539959 | 1.00834537 | 0.04953818 | 0.22380008 |
| MIAT | 617.528335 | -0.7840879 | 0.04954392 | 0.22380008 |
| TIMM8B | 294.136273 | -0.3806866 | 0.04962156 | 0.22409327 |
| DHX58 | 246.870147 | 0.39770879 | 0.04967382 | 0.22427167 |
| DTYMK | 121.657804 | -0.4389273 | 0.04970322 | 0.22434685 |

| | | | | |
|---|---|---|---|---|
| ASF1A | 259.011308 | 0.41992363 | 0.04974818 | 0.22437706 |
| LBX1 | 1.59052284 | -0.9154071 | 0.04972273 | 0.22437706 |
| TNFRSF8 | 6.04640602 | 0.83516951 | 0.04974449 | 0.22437706 |
| RNPEPL1 | 537.124924 | -0.3615973 | 0.04976736 | 0.22440602 |
| ATP6V1B1 | 16.2489035 | -0.971931 | 0.04980181 | 0.22442742 |
| FLII | 2123.67544 | -0.2826066 | 0.0497983 | 0.22442742 |
| KCNMB3 | 137.737048 | -0.5266599 | 0.04982314 | 0.22442742 |
| MOB3A | 486.099836 | 0.2729282 | 0.0498131 | 0.22442742 |
| CLDN11 | 5740.14042 | -0.9116287 | 0.04986335 | 0.22455102 |
| KCNK10 | 15.9116983 | 0.98701631 | 0.04995534 | 0.22490773 |
