# Supplementary material for: Transcriptome signatures associated with meningioma progression
Source: Acta Neuropathol Commun. 2019 Apr 30;7:67. doi: 10.1186/s40478-019-0690-x (PMC6489307; doi:10.1186/s40478-019-0690-x)
Supplement: Supplementary file 6 — Table S5. List of significantly differentially expressed genes between grade II S. and II DN meningiomas, as identified by RNA-seq. (PDF 255 kb) [file 40478_2019_690_MOESM6_ESM.pdf]

Supplementary Table 5: Differentially expressed genes between GR II S. and GR II DN meningiomas

Gene	baseMean	log2FoldChar	lfcSE	pvalue	padj
LRP2	2.16E+02	3.05E+00	5.13E-01	2.71E-09	4.32E-05
GLDC	2.61E+02	-2.11E+00	3.67E-01	9.36E-09	5.36E-05
SLC6A12	5.19E+02	2.89E+00	5.05E-01	1.01E-08	5.36E-05
TMEM100	1.59E+03	-2.35E+00	4.50E-01	1.70E-07	6.76E-04
TMEM132B	6.26E+01	2.31E+00	4.48E-01	2.50E-07	7.96E-04
SLC26A7	7.45E+02	-2.63E+00	5.33E-01	7.95E-07	2.11E-03
LOC1019293	2.19E+01	-2.63E+00	5.42E-01	1.16E-06	2.41E-03
RABL3	1.92E+02	8.51E-01	1.76E-01	1.30E-06	2.41E-03
RET	1.72E+01	2.62E+00	5.42E-01	1.36E-06	2.41E-03
HECTD4	4.72E+03	8.05E-01	1.69E-01	1.90E-06	2.52E-03
LINC00478	3.16E+02	2.11E+00	4.43E-01	1.78E-06	2.52E-03
PIAS1	1.49E+03	8.08E-01	1.69E-01	1.83E-06	2.52E-03
SH3GL1P2	1.41E+01	-2.55E+00	5.42E-01	2.64E-06	3.24E-03
LOC1027235	2.15E+01	2.52E+00	5.44E-01	3.48E-06	3.70E-03
NNAT	3.01E+02	2.53E+00	5.45E-01	3.41E-06	3.70E-03
AOX1	2.07E+03	-1.97E+00	4.26E-01	3.88E-06	3.87E-03
HAAO	1.08E+02	-2.05E+00	4.48E-01	4.91E-06	4.61E-03
C5orf30	3.66E+02	1.81E+00	4.01E-01	6.41E-06	5.31E-03
CILP	1.15E+02	2.39E+00	5.30E-01	6.50E-06	5.31E-03
INF2	1.31E+03	1.20E+00	2.65E-01	6.66E-06	5.31E-03
CX3CR1	6.78E+02	-2.12E+00	4.76E-01	8.21E-06	6.05E-03
GABRE	1.43E+03	1.94E+00	4.36E-01	8.64E-06	6.05E-03
SLC6A13	1.13E+03	2.33E+00	5.24E-01	8.72E-06	6.05E-03
LHFPL2	4.94E+03	1.31E+00	2.98E-01	1.14E-05	7.27E-03
SUSD5	7.85E+01	2.30E+00	5.25E-01	1.14E-05	7.27E-03
SNAPC1	7.85E+01	1.16E+00	2.68E-01	1.42E-05	8.71E-03
HEY1	2.34E+02	1.16E+00	2.68E-01	1.48E-05	8.72E-03
CA9	4.63E+01	2.27E+00	5.33E-01	2.09E-05	1.19E-02
IFI27	2.93E+02	1.71E+00	4.04E-01	2.25E-05	1.24E-02
LOC653602	1.32E+01	2.02E+00	4.80E-01	2.47E-05	1.31E-02
ADAM19	3.33E+02	1.58E+00	3.76E-01	2.78E-05	1.43E-02
ITSN1	1.58E+03	9.20E-01	2.20E-01	2.94E-05	1.46E-02
ILDR2	6.88E+01	2.14E+00	5.14E-01	3.24E-05	1.52E-02
THSD4	2.68E+04	-1.31E+00	3.15E-01	3.20E-05	1.52E-02
MAB21L1	3.99E+01	-2.15E+00	5.19E-01	3.58E-05	1.63E-02
SYNJ2BP	9.61E+02	8.05E-01	1.95E-01	3.79E-05	1.68E-02
DLGAP2	3.92E+01	-2.21E+00	5.39E-01	3.95E-05	1.70E-02
TRIM22	2.65E+03	7.13E-01	1.74E-01	4.04E-05	1.70E-02
PDLIM2	2.54E+02	-1.01E+00	2.49E-01	4.65E-05	1.90E-02
ENPEP	9.02E+02	1.75E+00	4.30E-01	4.88E-05	1.95E-02
BAIAP2	3.91E+02	1.05E+00	2.62E-01	5.85E-05	2.28E-02
ATP1A2	5.59E+03	2.17E+00	5.43E-01	6.31E-05	2.39E-02
FGF7	3.36E+01	-1.98E+00	4.99E-01	7.45E-05	2.77E-02
CECR1	2.38E+02	-1.55E+00	3.94E-01	8.50E-05	2.85E-02
DLEU7-AS1	1.66E+01	-2.09E+00	5.32E-01	8.57E-05	2.85E-02
ISG15	8.98E+01	1.24E+00	3.16E-01	8.00E-05	2.85E-02
PGM1	5.54E+02	-1.33E+00	3.38E-01	8.34E-05	2.85E-02
RPRM	8.22E+01	-2.07E+00	5.26E-01	8.20E-05	2.85E-02
ANKRD20A1	8.16E+01	-2.13E+00	5.43E-01	8.91E-05	2.90E-02
TLR1	2.77E+02	-1.16E+00	3.00E-01	1.05E-04	3.37E-02

CNRIP1	2.80E+02	-1.09E+00	2.83E-01	1.24E-04	3.82E-02
ZNF189	9.52E+02	1.05E+00	2.74E-01	1.24E-04	3.82E-02
C21orf15	1.45E+02	-2.08E+00	5.45E-01	1.33E-04	3.99E-02
COL18A1	2.22E+03	1.82E+00	4.79E-01	1.44E-04	4.24E-02
C2orf40	4.95E+02	-1.71E+00	4.54E-01	1.62E-04	4.50E-02
MGC16025	1.04E+02	-1.29E+00	3.44E-01	1.72E-04	4.50E-02
REC8	1.09E+02	1.81E+00	4.80E-01	1.65E-04	4.50E-02
RHOV	4.63E+00	2.03E+00	5.41E-01	1.70E-04	4.50E-02
SLC16A9	8.01E+02	-1.54E+00	4.07E-01	1.59E-04	4.50E-02
TMEM144	5.47E+01	-1.35E+00	3.59E-01	1.64E-04	4.50E-02
TMEM171	4.98E+00	1.94E+00	5.16E-01	1.70E-04	4.50E-02
LAD1	5.68E+00	2.04E+00	5.43E-01	1.77E-04	4.55E-02
ASPM	1.76E+02	1.33E+00	3.57E-01	1.87E-04	4.59E-02
DHRS12	1.14E+02	8.99E-01	2.40E-01	1.82E-04	4.59E-02
GAL3ST4	2.54E+02	-9.95E-01	2.66E-01	1.87E-04	4.59E-02
EBF2	1.72E+02	2.03E+00	5.44E-01	1.92E-04	4.64E-02
PLXNC1	5.31E+03	1.64E+00	4.43E-01	2.09E-04	4.84E-02
SLC9A9	2.97E+02	-1.07E+00	2.90E-01	2.08E-04	4.84E-02
TRAF5	1.86E+03	1.54E+00	4.15E-01	2.09E-04	4.84E-02
GALNT18	1.62E+02	1.45E+00	3.92E-01	2.16E-04	4.86E-02
PDLIM3	2.27E+01	1.95E+00	5.26E-01	2.16E-04	4.86E-02
SORBS2	1.27E+02	1.70E+00	4.60E-01	2.25E-04	4.98E-02
NALCN	1.89E+03	6.87E-01	1.87E-01	2.34E-04	5.11E-02
ITPRIPL1	3.45E+01	-1.56E+00	4.27E-01	2.44E-04	5.19E-02
LINC00869	5.52E+01	-1.15E+00	3.13E-01	2.42E-04	5.19E-02
C16orf86	1.74E+01	-1.70E+00	4.66E-01	2.64E-04	5.54E-02
ADM	2.67E+02	1.64E+00	4.51E-01	2.73E-04	5.65E-02
H19	1.52E+04	-1.98E+00	5.45E-01	2.86E-04	5.71E-02
LYNX1	6.87E+02	-1.53E+00	4.21E-01	2.81E-04	5.71E-02
OR52N4	1.12E+01	1.75E+00	4.84E-01	2.86E-04	5.71E-02
LINC00886	7.71E+01	-1.63E+00	4.50E-01	2.92E-04	5.75E-02
KLRG2	7.80E+01	1.96E+00	5.42E-01	2.99E-04	5.82E-02
DZANK1	3.96E+01	1.38E+00	3.82E-01	3.18E-04	5.84E-02
FAM46C	1.18E+02	1.59E+00	4.43E-01	3.18E-04	5.84E-02
PAPLN	1.94E+03	1.80E+00	4.99E-01	3.18E-04	5.84E-02
RANBP3L	3.55E+02	1.86E+00	5.14E-01	3.05E-04	5.84E-02
TCHH	1.33E+01	1.92E+00	5.33E-01	3.11E-04	5.84E-02
CBLL1	6.20E+02	9.85E-01	2.75E-01	3.42E-04	6.13E-02
SYPL2	3.54E+01	-1.50E+00	4.18E-01	3.42E-04	6.13E-02
IMPDH1	3.90E+02	1.23E+00	3.46E-01	3.59E-04	6.23E-02
ITGA1	1.61E+03	1.33E+00	3.74E-01	3.70E-04	6.23E-02
MAP1LC3B	1.28E+03	-5.25E-01	1.47E-01	3.58E-04	6.23E-02
PLA2G6	2.68E+02	-1.00E+00	2.82E-01	3.64E-04	6.23E-02
RCSD1	8.51E+02	-1.20E+00	3.38E-01	3.69E-04	6.23E-02
ZMYND8	1.82E+03	9.31E-01	2.62E-01	3.71E-04	6.23E-02
EFCAB11	5.38E+01	1.26E+00	3.54E-01	3.85E-04	6.31E-02
EXPH5	1.31E+02	-1.69E+00	4.77E-01	3.88E-04	6.31E-02
GINS1	7.42E+01	1.15E+00	3.25E-01	3.80E-04	6.31E-02
OR5P2	2.82E+00	1.92E+00	5.42E-01	3.98E-04	6.35E-02
PLA2G7	5.81E+01	-1.90E+00	5.38E-01	3.97E-04	6.35E-02
NUAK2	1.13E+02	1.24E+00	3.51E-01	4.11E-04	6.43E-02
SAV1	5.06E+02	1.05E+00	2.97E-01	4.15E-04	6.43E-02
SRPRB	7.37E+02	-9.90E-01	2.80E-01	4.09E-04	6.43E-02

CCDC102A	1.52E+02	-1.12E+00	3.20E-01	4.41E-04	6.64E-02
KIF20B	2.19E+02	8.96E-01	2.55E-01	4.39E-04	6.64E-02
SYNE2	4.41E+03	1.38E+00	3.92E-01	4.40E-04	6.64E-02
CALM1	3.03E+03	9.36E-01	2.67E-01	4.54E-04	6.70E-02
MARVELD3	8.11E+01	1.91E+00	5.45E-01	4.50E-04	6.70E-02
POLR1D	4.33E+02	5.64E-01	1.61E-01	4.66E-04	6.82E-02
LRP8	1.52E+02	1.62E+00	4.63E-01	4.72E-04	6.84E-02
NANOS1	4.20E+01	1.62E+00	4.63E-01	4.82E-04	6.93E-02
AKR1B10	7.02E+00	1.90E+00	5.45E-01	5.01E-04	7.04E-02
COBLL1	6.85E+02	-1.46E+00	4.20E-01	5.16E-04	7.04E-02
GPR135	3.21E+01	1.12E+00	3.21E-01	5.09E-04	7.04E-02
RASSF3	4.75E+02	1.24E+00	3.55E-01	4.95E-04	7.04E-02
SFRP5	6.76E+00	1.89E+00	5.45E-01	5.13E-04	7.04E-02
SLC4A7	6.58E+02	-1.05E+00	3.01E-01	5.05E-04	7.04E-02
MOB3B	9.28E+01	1.69E+00	4.86E-01	5.21E-04	7.05E-02
UBE2QL1	7.39E+01	1.69E+00	4.89E-01	5.30E-04	7.10E-02
CLMP	6.78E+02	-1.26E+00	3.65E-01	5.36E-04	7.13E-02
ANKRD46	2.04E+02	-7.40E-01	2.14E-01	5.57E-04	7.26E-02
CHRNA4	1.72E+02	1.85E+00	5.37E-01	5.53E-04	7.26E-02
COMMD7	2.82E+02	6.86E-01	1.99E-01	5.68E-04	7.26E-02
DYNC1H1	9.80E+03	5.38E-01	1.56E-01	5.62E-04	7.26E-02
RALGPS2	2.51E+02	1.41E+00	4.09E-01	5.69E-04	7.26E-02
IFIT3	3.22E+02	8.96E-01	2.60E-01	5.79E-04	7.33E-02
SYCE1	4.07E+01	1.86E+00	5.41E-01	5.88E-04	7.38E-02
ENC1	2.69E+03	1.65E+00	4.80E-01	6.03E-04	7.46E-02
FAM49A	1.82E+02	1.70E+00	4.96E-01	6.00E-04	7.46E-02
ABLIM2	4.15E+01	1.53E+00	4.46E-01	6.12E-04	7.51E-02
HLCS	6.41E+02	1.10E+00	3.22E-01	6.20E-04	7.54E-02
HCAR1	1.46E+02	1.61E+00	4.72E-01	6.29E-04	7.60E-02
PNMA1	6.90E+02	9.98E-01	2.93E-01	6.58E-04	7.89E-02
OXER1	3.33E+01	-1.69E+00	4.97E-01	6.68E-04	7.95E-02
AJUBA	6.15E+02	9.98E-01	2.94E-01	6.97E-04	8.24E-02
CD28	3.46E+01	1.84E+00	5.45E-01	7.08E-04	8.30E-02
FKBP3	3.36E+02	9.74E-01	2.88E-01	7.18E-04	8.36E-02
PCP4L1	1.83E+01	1.83E+00	5.42E-01	7.27E-04	8.41E-02
FAM150B	1.07E+01	1.83E+00	5.45E-01	7.71E-04	8.81E-02
PRTFDC1	1.18E+02	-1.07E+00	3.19E-01	7.73E-04	8.81E-02
CRYZL1	2.28E+02	5.83E-01	1.74E-01	7.85E-04	8.88E-02
FAM129A	2.88E+03	1.72E+00	5.12E-01	7.92E-04	8.89E-02
HSPB2	1.66E+02	-8.24E-01	2.46E-01	8.00E-04	8.93E-02
JAG2	2.35E+02	1.35E+00	4.03E-01	8.09E-04	8.96E-02
ELFN1	2.41E+02	-1.71E+00	5.11E-01	8.16E-04	8.98E-02
TBX5	1.28E+01	1.72E+00	5.14E-01	8.22E-04	8.98E-02
CCDC85C	3.60E+02	1.19E+00	3.57E-01	8.42E-04	9.14E-02
ARHGEF39	4.50E+01	1.43E+00	4.29E-01	8.48E-04	9.14E-02
TEX15	5.09E+02	-1.73E+00	5.20E-01	8.66E-04	9.27E-02
CPPED1	5.31E+02	1.45E+00	4.37E-01	9.05E-04	9.50E-02
FOXC2	1.66E+03	-9.15E-01	2.75E-01	8.96E-04	9.50E-02
STPG2	1.13E+01	-1.42E+00	4.29E-01	9.05E-04	9.50E-02
FLJ36777	3.21E+00	-1.80E+00	5.44E-01	9.28E-04	9.61E-02
RCOR2	4.53E+00	1.71E+00	5.15E-01	9.26E-04	9.61E-02
LOC1019272	1.26E+01	-1.78E+00	5.39E-01	9.46E-04	9.73E-02
FOXM1	1.02E+02	8.37E-01	2.54E-01	9.61E-04	9.78E-02

NMRK1	7.31E+01	-9.20E-01	2.79E-01	9.63E-04	9.78E-02
ADAM20	6.21E+01	1.22E+00	3.69E-01	9.94E-04	9.97E-02
PPARGC1A	1.22E+03	-1.62E+00	4.93E-01	9.90E-04	9.97E-02
IL20RA	1.28E+01	-1.64E+00	4.98E-01	1.02E-03	1.01E-01
PCCA	1.03E+03	9.96E-01	3.03E-01	1.02E-03	1.02E-01
EIF5	1.83E+03	7.08E-01	2.16E-01	1.04E-03	1.02E-01
SH3D21	1.13E+02	1.51E+00	4.59E-01	1.04E-03	1.02E-01
CADM3	3.25E+01	-1.70E+00	5.18E-01	1.05E-03	1.02E-01
COL4A4	3.47E+02	1.78E+00	5.44E-01	1.08E-03	1.03E-01
DTNA	1.01E+02	-1.16E+00	3.55E-01	1.08E-03	1.03E-01
EDA	3.37E+02	1.35E+00	4.13E-01	1.07E-03	1.03E-01
BTBD11	4.20E+02	1.77E+00	5.41E-01	1.09E-03	1.04E-01
SULF1	5.57E+03	-1.69E+00	5.18E-01	1.10E-03	1.04E-01
DDX26B	3.54E+02	1.48E+00	4.53E-01	1.13E-03	1.06E-01
SLC24A4	1.15E+01	1.39E+00	4.25E-01	1.13E-03	1.06E-01
HS6ST1	5.66E+02	1.05E+00	3.22E-01	1.15E-03	1.07E-01
BEND6	4.30E+01	-1.50E+00	4.61E-01	1.17E-03	1.08E-01
CSNK2A3	1.26E+01	1.63E+00	5.03E-01	1.19E-03	1.08E-01
FAM222B	4.96E+02	6.75E-01	2.09E-01	1.24E-03	1.08E-01
FMO1	4.24E+02	-1.64E+00	5.08E-01	1.23E-03	1.08E-01
HSPBAP1	7.40E+01	-9.54E-01	2.95E-01	1.22E-03	1.08E-01
MME	1.19E+01	1.61E+00	4.96E-01	1.19E-03	1.08E-01
NSG1	8.17E+01	1.71E+00	5.30E-01	1.23E-03	1.08E-01
RRP1B	6.79E+02	6.67E-01	2.06E-01	1.24E-03	1.08E-01
SMOC1	2.75E+03	1.61E+00	4.99E-01	1.24E-03	1.08E-01
SSTR2	2.96E+03	-1.43E+00	4.43E-01	1.23E-03	1.08E-01
ZNF738	2.19E+02	1.04E+00	3.21E-01	1.20E-03	1.08E-01
COCH	1.02E+02	1.75E+00	5.43E-01	1.26E-03	1.09E-01
CPED1	3.81E+02	-1.60E+00	4.97E-01	1.25E-03	1.09E-01
IREB2	1.50E+03	3.94E-01	1.23E-01	1.33E-03	1.12E-01
PCBP3	5.53E+01	1.59E+00	4.95E-01	1.31E-03	1.12E-01
ZNF718	1.92E+02	7.96E-01	2.48E-01	1.32E-03	1.12E-01
CYP4F3	3.39E+01	1.74E+00	5.44E-01	1.35E-03	1.14E-01
VEZT	1.41E+03	6.54E-01	2.04E-01	1.36E-03	1.14E-01
ARG2	8.84E+01	1.67E+00	5.22E-01	1.40E-03	1.15E-01
MTUS2	3.71E+01	1.72E+00	5.38E-01	1.41E-03	1.15E-01
OLFM4	6.36E+01	1.57E+00	4.93E-01	1.41E-03	1.15E-01
PRMT2	1.71E+03	5.83E-01	1.83E-01	1.41E-03	1.15E-01
SOD1	1.39E+03	5.41E-01	1.69E-01	1.40E-03	1.15E-01
SOX6	6.23E+01	-1.73E+00	5.42E-01	1.44E-03	1.17E-01
CKMT1A	1.09E+01	-1.73E+00	5.44E-01	1.48E-03	1.19E-01
FBXO34	2.57E+02	9.00E-01	2.83E-01	1.48E-03	1.19E-01
MAL2	4.74E+02	-1.69E+00	5.33E-01	1.49E-03	1.19E-01
NHS	6.19E+02	1.06E+00	3.35E-01	1.49E-03	1.19E-01
PGBD5	2.65E+01	1.28E+00	4.04E-01	1.50E-03	1.19E-01
PDXK	4.35E+03	9.03E-01	2.85E-01	1.51E-03	1.19E-01
STOX1	4.45E+01	1.63E+00	5.13E-01	1.54E-03	1.21E-01
TREX1	1.16E+02	-7.40E-01	2.34E-01	1.59E-03	1.25E-01
FBP1	4.06E+01	-1.35E+00	4.28E-01	1.60E-03	1.25E-01
CARD11	1.69E+02	-1.16E+00	3.68E-01	1.64E-03	1.26E-01
CDH4	1.40E+02	1.71E+00	5.43E-01	1.64E-03	1.26E-01
COL4A3	2.26E+02	1.71E+00	5.43E-01	1.65E-03	1.26E-01
FHL3	2.16E+02	1.30E+00	4.15E-01	1.66E-03	1.26E-01

TLR6	1.52E+02	-1.13E+00	3.57E-01	1.63E-03	1.26E-01
ALDH2	4.30E+03	-1.04E+00	3.30E-01	1.68E-03	1.27E-01
C12orf65	1.41E+02	6.50E-01	2.07E-01	1.71E-03	1.27E-01
KCNJ2	6.49E+02	-1.58E+00	5.02E-01	1.72E-03	1.27E-01
KLF6	2.24E+03	1.09E+00	3.47E-01	1.71E-03	1.27E-01
MIR4697HG	2.29E+02	1.23E+00	3.92E-01	1.71E-03	1.27E-01
RNF144A	2.88E+02	1.43E+00	4.56E-01	1.72E-03	1.27E-01
AKR1C3	2.98E+02	1.35E+00	4.32E-01	1.73E-03	1.27E-01
KNSTRN	9.17E+01	7.23E-01	2.31E-01	1.79E-03	1.31E-01
APCDD1	1.43E+03	-1.55E+00	4.96E-01	1.82E-03	1.32E-01
EHF	3.03E+02	1.46E+00	4.68E-01	1.83E-03	1.32E-01
SCFD1	5.33E+02	7.86E-01	2.52E-01	1.81E-03	1.32E-01
ATP1B1	2.01E+03	-1.17E+00	3.76E-01	1.87E-03	1.33E-01
CYP3A5	5.33E+02	-1.32E+00	4.23E-01	1.86E-03	1.33E-01
EPSTI1	7.93E+01	1.06E+00	3.39E-01	1.87E-03	1.33E-01
PELO	3.92E+02	8.45E-01	2.72E-01	1.90E-03	1.34E-01
SLC2A1	6.28E+02	1.30E+00	4.20E-01	1.90E-03	1.34E-01
MFAP5	6.77E+02	-1.63E+00	5.26E-01	1.93E-03	1.35E-01
SERPINB10	1.09E+01	-1.69E+00	5.45E-01	1.94E-03	1.36E-01
AMOTL1	3.23E+03	1.16E+00	3.73E-01	1.97E-03	1.37E-01
ZFAND1	3.20E+02	-6.73E-01	2.18E-01	1.97E-03	1.37E-01
CIAPIN1	3.35E+02	-8.69E-01	2.81E-01	2.00E-03	1.38E-01
RHOQ	1.37E+03	-8.03E-01	2.60E-01	1.99E-03	1.38E-01
MOV10L1	6.84E+00	1.64E+00	5.32E-01	2.02E-03	1.38E-01
FANCM	1.86E+02	9.65E-01	3.13E-01	2.03E-03	1.38E-01
FAM162B	5.62E+01	-1.68E+00	5.45E-01	2.05E-03	1.39E-01
CRYGN	1.16E+02	-1.48E+00	4.81E-01	2.11E-03	1.41E-01
MESTIT1	1.40E+01	-1.64E+00	5.35E-01	2.10E-03	1.41E-01
WDR25	1.02E+02	1.01E+00	3.28E-01	2.11E-03	1.41E-01
DPP10	2.38E+01	1.66E+00	5.42E-01	2.13E-03	1.42E-01
PRPF39	2.84E+02	6.74E-01	2.20E-01	2.13E-03	1.42E-01
KIF4A	3.93E+01	1.10E+00	3.58E-01	2.17E-03	1.43E-01
MAP3K13	4.42E+02	-6.22E-01	2.03E-01	2.17E-03	1.43E-01
ZNF521	4.05E+02	1.67E+00	5.45E-01	2.18E-03	1.43E-01
PDS5B	1.16E+03	6.16E-01	2.01E-01	2.20E-03	1.44E-01
PDPR	1.31E+03	-8.24E-01	2.69E-01	2.23E-03	1.45E-01
TDP1	1.15E+02	8.07E-01	2.64E-01	2.26E-03	1.47E-01
ZCCHC14	9.43E+02	-7.48E-01	2.45E-01	2.30E-03	1.48E-01
RPS10P7	2.25E+01	-1.16E+00	3.82E-01	2.31E-03	1.49E-01
GPAT2	9.98E+00	1.65E+00	5.45E-01	2.38E-03	1.51E-01
TFE3	9.28E+02	4.33E-01	1.42E-01	2.36E-03	1.51E-01
TRIM5	9.14E+02	7.39E-01	2.43E-01	2.37E-03	1.51E-01
DECR1	5.06E+02	-1.20E+00	3.97E-01	2.40E-03	1.52E-01
KLF12	1.40E+03	6.86E-01	2.26E-01	2.43E-03	1.53E-01
NCOA3	2.36E+03	6.93E-01	2.29E-01	2.43E-03	1.53E-01
ARHGAP5	3.11E+03	7.26E-01	2.40E-01	2.45E-03	1.53E-01
CTPS2	3.35E+02	6.48E-01	2.14E-01	2.48E-03	1.54E-01
MOXD1	4.63E+00	1.65E+00	5.45E-01	2.51E-03	1.56E-01
KTN1	2.87E+03	8.59E-01	2.84E-01	2.52E-03	1.56E-01
LGI3	5.54E+01	1.55E+00	5.12E-01	2.56E-03	1.58E-01
CR1	1.92E+01	1.62E+00	5.39E-01	2.59E-03	1.58E-01
MACC1	2.00E+01	-1.24E+00	4.10E-01	2.59E-03	1.58E-01
VRK1	7.20E+01	9.34E-01	3.10E-01	2.60E-03	1.58E-01

GADL1	1.61E+01	-1.64E+00	5.44E-01	2.64E-03	1.58E-01
LOC1005066	8.66E+01	-1.63E+00	5.43E-01	2.62E-03	1.58E-01
PAN3	1.07E+03	5.34E-01	1.78E-01	2.64E-03	1.58E-01
SCNN1G	4.48E+00	1.52E+00	5.04E-01	2.62E-03	1.58E-01
GNG7	2.68E+02	-1.12E+00	3.72E-01	2.65E-03	1.58E-01
ACKR3	8.94E+02	-1.40E+00	4.69E-01	2.81E-03	1.61E-01
DGCR9	1.29E+01	-1.42E+00	4.73E-01	2.72E-03	1.61E-01
ERO1L	8.83E+02	9.51E-01	3.18E-01	2.79E-03	1.61E-01
FAM91A1	3.11E+03	-1.27E+00	4.25E-01	2.79E-03	1.61E-01
GPC5	1.53E+01	-1.62E+00	5.40E-01	2.74E-03	1.61E-01
HDAC9	3.95E+02	-1.32E+00	4.41E-01	2.79E-03	1.61E-01
LOC283299	6.93E+00	1.62E+00	5.40E-01	2.74E-03	1.61E-01
LOC654841	1.16E+01	-1.41E+00	4.71E-01	2.81E-03	1.61E-01
MTMR2	8.90E+02	1.08E+00	3.62E-01	2.79E-03	1.61E-01
SGK223	5.37E+02	-1.61E+00	5.37E-01	2.75E-03	1.61E-01
SLC39A14	8.40E+02	-1.43E+00	4.76E-01	2.72E-03	1.61E-01
SNRNP35	1.73E+02	3.91E-01	1.30E-01	2.71E-03	1.61E-01
ADCY10P1	3.97E+01	-1.07E+00	3.60E-01	2.83E-03	1.61E-01
GLI3	6.33E+02	-1.07E+00	3.58E-01	2.89E-03	1.61E-01
LEPREL1	1.76E+02	-1.53E+00	5.12E-01	2.86E-03	1.61E-01
LOC1019295	9.29E+00	-1.62E+00	5.42E-01	2.88E-03	1.61E-01
LUM	3.45E+02	-1.62E+00	5.45E-01	2.90E-03	1.61E-01
MBNL1-AS1	5.73E+01	1.04E+00	3.50E-01	2.90E-03	1.61E-01
POP5	1.44E+02	-5.35E-01	1.79E-01	2.87E-03	1.61E-01
ZNF740	5.14E+02	4.67E-01	1.57E-01	2.89E-03	1.61E-01
IFI6	3.96E+02	1.07E+00	3.58E-01	2.93E-03	1.62E-01
GLIS2	9.64E+02	-6.52E-01	2.19E-01	2.95E-03	1.63E-01
ADCY1	1.56E+02	1.40E+00	4.77E-01	3.24E-03	1.63E-01
CHST2	1.71E+02	1.37E+00	4.61E-01	3.04E-03	1.63E-01
CKMT1B	8.51E+00	-1.61E+00	5.45E-01	3.18E-03	1.63E-01
COPZ2	9.09E+02	-7.41E-01	2.51E-01	3.16E-03	1.63E-01
CSE1L	1.09E+03	5.95E-01	2.02E-01	3.15E-03	1.63E-01
CSTB	8.60E+02	1.07E+00	3.62E-01	3.10E-03	1.63E-01
DPY19L1	9.06E+02	-7.37E-01	2.50E-01	3.20E-03	1.63E-01
EPN3	2.64E+01	-1.60E+00	5.40E-01	3.02E-03	1.63E-01
ERV3-1	6.97E+02	-7.88E-01	2.67E-01	3.20E-03	1.63E-01
ESM1	8.16E+01	1.46E+00	4.92E-01	3.11E-03	1.63E-01
EXOC5	8.49E+02	8.22E-01	2.78E-01	3.14E-03	1.63E-01
GOLGA6L9	1.63E+01	1.20E+00	4.07E-01	3.25E-03	1.63E-01
HLA-H	1.62E+01	1.22E+00	4.14E-01	3.21E-03	1.63E-01
IRF9	3.67E+02	9.05E-01	3.08E-01	3.26E-03	1.63E-01
KIAA0922	5.32E+02	1.48E+00	5.02E-01	3.24E-03	1.63E-01
KIAA1324	8.64E+00	1.58E+00	5.37E-01	3.22E-03	1.63E-01
LINC01152	4.33E+01	-1.56E+00	5.26E-01	3.07E-03	1.63E-01
LMO4	2.54E+03	-1.34E+00	4.54E-01	3.27E-03	1.63E-01
LRIG3	5.44E+02	1.08E+00	3.65E-01	3.07E-03	1.63E-01
LSAMP	5.53E+01	-1.61E+00	5.42E-01	3.01E-03	1.63E-01
LTBP1	1.41E+03	-1.45E+00	4.91E-01	3.23E-03	1.63E-01
MPP5	4.44E+02	8.61E-01	2.91E-01	3.11E-03	1.63E-01
RHEB	9.15E+02	-7.36E-01	2.50E-01	3.19E-03	1.63E-01
RTKN	2.38E+02	-7.59E-01	2.57E-01	3.19E-03	1.63E-01
SDPR	9.01E+02	1.14E+00	3.85E-01	3.14E-03	1.63E-01
STK38	1.15E+03	6.95E-01	2.35E-01	3.12E-03	1.63E-01

STXBP6	3.51E+02	1.47E+00	4.97E-01	3.04E-03	1.63E-01
TSC22D1-AS1	2.68E+01	1.25E+00	4.24E-01	3.17E-03	1.63E-01
UGT8	1.83E+01	-1.59E+00	5.42E-01	3.25E-03	1.63E-01
ZBTB42	5.05E+01	1.20E+00	4.06E-01	3.06E-03	1.63E-01
CFL2	5.88E+02	6.20E-01	2.11E-01	3.30E-03	1.64E-01
GNG4	1.51E+02	1.52E+00	5.18E-01	3.29E-03	1.64E-01
SEMA4G	9.05E+01	1.17E+00	3.99E-01	3.30E-03	1.64E-01
ARHGEF7	1.62E+03	8.91E-01	3.04E-01	3.42E-03	1.67E-01
ERAP2	5.08E+02	1.50E+00	5.10E-01	3.40E-03	1.67E-01
IFIT1	3.70E+02	1.06E+00	3.64E-01	3.41E-03	1.67E-01
LYSMD4	1.51E+02	8.73E-01	2.98E-01	3.41E-03	1.67E-01
TNNI3	5.56E+00	1.60E+00	5.45E-01	3.40E-03	1.67E-01
ST8SIA1	2.14E+01	-1.53E+00	5.23E-01	3.43E-03	1.67E-01
DENND5B	3.12E+02	7.19E-01	2.46E-01	3.44E-03	1.67E-01
SLC27A6	8.01E+01	-1.59E+00	5.43E-01	3.47E-03	1.68E-01
PTH2R	1.94E+01	-1.59E+00	5.45E-01	3.50E-03	1.69E-01
ANGPTL7	1.00E+02	1.54E+00	5.27E-01	3.53E-03	1.69E-01
KCNK10	1.00E+01	-1.58E+00	5.43E-01	3.53E-03	1.69E-01
CDK10	2.82E+02	-5.27E-01	1.81E-01	3.55E-03	1.70E-01
ABHD12	4.92E+02	8.22E-01	2.83E-01	3.61E-03	1.71E-01
FAM189A2	3.76E+01	1.28E+00	4.40E-01	3.63E-03	1.71E-01
GK5	1.18E+03	9.44E-01	3.24E-01	3.60E-03	1.71E-01
METTL25	8.91E+01	7.84E-01	2.69E-01	3.63E-03	1.71E-01
PF4	4.25E+00	1.49E+00	5.12E-01	3.63E-03	1.71E-01
TNFSF10	3.73E+02	-1.32E+00	4.56E-01	3.70E-03	1.74E-01
ABCC3	6.91E+01	-1.37E+00	4.73E-01	3.73E-03	1.74E-01
ABHD10	4.22E+02	-4.71E-01	1.62E-01	3.73E-03	1.74E-01
MMP14	2.57E+03	7.96E-01	2.75E-01	3.81E-03	1.77E-01
MED7	1.04E+02	5.72E-01	1.98E-01	3.83E-03	1.77E-01
MED6	1.96E+02	8.67E-01	3.00E-01	3.88E-03	1.79E-01
MGC16275	1.72E+01	1.05E+00	3.63E-01	3.89E-03	1.79E-01
ZNF33B	4.66E+02	9.54E-01	3.30E-01	3.87E-03	1.79E-01
CCDC40	9.17E+01	-8.95E-01	3.10E-01	3.91E-03	1.79E-01
SLC12A5	1.41E+01	-1.36E+00	4.73E-01	3.91E-03	1.79E-01
TATDN2	9.70E+02	-6.05E-01	2.10E-01	3.93E-03	1.79E-01
SIDT1	8.92E+01	-1.51E+00	5.25E-01	3.95E-03	1.80E-01
CDC42SE2	6.13E+02	5.95E-01	2.07E-01	4.00E-03	1.81E-01
TLR10	2.82E+01	-1.48E+00	5.15E-01	4.00E-03	1.81E-01
ALX4	7.99E+02	-1.46E+00	5.09E-01	4.04E-03	1.82E-01
GRK5	2.69E+02	1.07E+00	3.74E-01	4.06E-03	1.82E-01
PIM2	9.66E+01	-6.63E-01	2.31E-01	4.06E-03	1.82E-01
SUCLG2-AS1	4.05E+01	-1.02E+00	3.55E-01	4.08E-03	1.82E-01
ZNF430	1.85E+02	6.85E-01	2.39E-01	4.08E-03	1.82E-01
DOCK10	3.71E+02	-1.02E+00	3.55E-01	4.11E-03	1.82E-01
FAM171B	9.50E+02	-9.57E-01	3.34E-01	4.11E-03	1.82E-01
ATP11C	7.80E+02	1.30E+00	4.53E-01	4.18E-03	1.84E-01
ADAM20P1	5.42E+01	1.25E+00	4.37E-01	4.24E-03	1.86E-01
ADAM33	7.30E+02	1.26E+00	4.39E-01	4.24E-03	1.86E-01
MT1M	4.78E+00	-1.55E+00	5.44E-01	4.29E-03	1.88E-01
ZIC1	3.63E+03	-1.39E+00	4.86E-01	4.29E-03	1.88E-01
LOXL2	1.86E+02	1.32E+00	4.63E-01	4.31E-03	1.88E-01
GRM3	1.44E+01	-1.55E+00	5.44E-01	4.37E-03	1.89E-01
TUBB1	1.20E+01	1.54E+00	5.41E-01	4.37E-03	1.89E-01

WDFY3-AS2	4.16E+01	-1.18E+00	4.13E-01	4.37E-03	1.89E-01
G2E3	2.60E+02	7.02E-01	2.47E-01	4.41E-03	1.90E-01
RPS2	6.24E+03	-6.68E-01	2.34E-01	4.41E-03	1.90E-01
ARHGAP26	7.80E+02	1.27E+00	4.45E-01	4.47E-03	1.92E-01
DLG5-AS1	6.51E+00	1.47E+00	5.16E-01	4.48E-03	1.92E-01
NT5DC3	1.62E+03	1.42E+00	5.00E-01	4.49E-03	1.92E-01
ANKS1A	1.27E+03	7.28E-01	2.56E-01	4.53E-03	1.93E-01
INPP1	1.68E+02	-6.83E-01	2.41E-01	4.57E-03	1.94E-01
SEL1L	1.87E+03	6.55E-01	2.31E-01	4.58E-03	1.94E-01
SLC5A3	1.19E+03	1.05E+00	3.70E-01	4.59E-03	1.94E-01
TGFB2	2.34E+03	-1.30E+00	4.57E-01	4.62E-03	1.94E-01
OR56B1	5.56E+00	1.40E+00	4.93E-01	4.63E-03	1.94E-01
BMP5	2.79E+03	-1.54E+00	5.43E-01	4.69E-03	1.95E-01
PNMA3	9.41E+01	-1.09E+00	3.84E-01	4.67E-03	1.95E-01
SLC7A7	3.73E+02	1.46E+00	5.17E-01	4.69E-03	1.95E-01
SPSB1	1.02E+02	1.29E+00	4.56E-01	4.69E-03	1.95E-01
CEP170B	5.85E+02	7.95E-01	2.81E-01	4.72E-03	1.95E-01
RGS4	3.03E+01	1.50E+00	5.29E-01	4.71E-03	1.95E-01
DZIP3	6.20E+02	-4.92E-01	1.74E-01	4.75E-03	1.96E-01
FMR1	9.51E+02	5.81E-01	2.06E-01	4.77E-03	1.96E-01
MAP4K5	5.81E+02	6.94E-01	2.46E-01	4.79E-03	1.96E-01
RBM25	1.46E+03	7.76E-01	2.75E-01	4.80E-03	1.96E-01
SLC38A2	1.66E+04	-8.38E-01	2.97E-01	4.82E-03	1.97E-01
ADAMTS16	7.52E+00	1.49E+00	5.30E-01	4.85E-03	1.97E-01
NUDT4	9.14E+01	1.18E+00	4.20E-01	4.86E-03	1.97E-01
CRIPAK	2.42E+02	1.00E+00	3.56E-01	4.95E-03	1.97E-01
DLG2	1.27E+02	-1.25E+00	4.43E-01	4.90E-03	1.97E-01
FBLN5	8.19E+02	1.07E+00	3.82E-01	4.92E-03	1.97E-01
GID8	8.56E+02	7.36E-01	2.62E-01	4.94E-03	1.97E-01
GOLGA5	5.15E+02	6.67E-01	2.37E-01	4.90E-03	1.97E-01
JADE3	2.76E+02	-5.75E-01	2.04E-01	4.90E-03	1.97E-01
ZNF827	2.93E+02	-9.25E-01	3.29E-01	4.94E-03	1.97E-01
CARD6	2.26E+02	-6.93E-01	2.47E-01	5.02E-03	2.00E-01
ZNF536	2.05E+02	1.21E+00	4.32E-01	5.08E-03	2.02E-01
CTU2	9.97E+01	-6.16E-01	2.20E-01	5.13E-03	2.02E-01
MBTPS2	4.35E+02	4.81E-01	1.72E-01	5.12E-03	2.02E-01
PAIP1	5.62E+02	3.18E-01	1.14E-01	5.13E-03	2.02E-01
RTEL1	1.40E+01	1.15E+00	4.12E-01	5.16E-03	2.03E-01
HAUS6	3.15E+02	4.78E-01	1.71E-01	5.18E-03	2.03E-01
BRAF	4.17E+02	-3.80E-01	1.36E-01	5.22E-03	2.03E-01
PLBD1	4.27E+02	-1.16E+00	4.17E-01	5.21E-03	2.03E-01
WARS	5.79E+02	8.04E-01	2.88E-01	5.22E-03	2.03E-01
PLP1	3.36E+02	-1.52E+00	5.45E-01	5.26E-03	2.04E-01
HCN2	5.95E+01	-1.44E+00	5.18E-01	5.30E-03	2.05E-01
SCN2A	4.10E+01	-1.52E+00	5.45E-01	5.31E-03	2.05E-01
ZNF117	1.07E+03	-6.51E-01	2.34E-01	5.37E-03	2.07E-01
ARHGAP29	1.51E+03	1.19E+00	4.27E-01	5.43E-03	2.09E-01
FCF1	3.03E+02	6.88E-01	2.48E-01	5.44E-03	2.09E-01
MEF2C	1.57E+03	-1.02E+00	3.68E-01	5.45E-03	2.09E-01
ERMN	2.57E+01	-1.51E+00	5.44E-01	5.50E-03	2.09E-01
RNF19A	1.12E+03	-6.23E-01	2.24E-01	5.49E-03	2.09E-01
ZNF714	2.81E+02	1.10E+00	3.98E-01	5.51E-03	2.09E-01
ATXN1	2.25E+03	8.88E-01	3.20E-01	5.55E-03	2.10E-01

GDF10	2.70E+01	-1.50E+00	5.41E-01	5.58E-03	2.11E-01
MTURN	1.25E+03	-9.35E-01	3.38E-01	5.60E-03	2.11E-01
RAPGEF4	4.79E+02	1.02E+00	3.67E-01	5.62E-03	2.11E-01
TRPC6	4.65E+01	1.43E+00	5.17E-01	5.62E-03	2.11E-01
NRDE2	2.35E+02	7.87E-01	2.84E-01	5.65E-03	2.11E-01
USP10	6.83E+02	-5.28E-01	1.91E-01	5.67E-03	2.12E-01
IGFBP6	1.81E+03	1.33E+00	4.79E-01	5.69E-03	2.12E-01
ITGB8	1.93E+03	-1.18E+00	4.29E-01	5.73E-03	2.13E-01
SRP54	4.63E+02	6.76E-01	2.45E-01	5.72E-03	2.13E-01
BAIAP2L1	3.17E+01	1.44E+00	5.23E-01	5.84E-03	2.13E-01
DIRAS1	7.00E+01	-1.47E+00	5.31E-01	5.78E-03	2.13E-01
EPB41L5	1.84E+03	1.21E+00	4.38E-01	5.82E-03	2.13E-01
ERICH2	5.54E+00	1.42E+00	5.16E-01	5.84E-03	2.13E-01
GSTM4	1.55E+02	-9.59E-01	3.47E-01	5.77E-03	2.13E-01
LINC00570	4.64E+00	-1.49E+00	5.42E-01	5.82E-03	2.13E-01
SRRM3	3.24E+01	-1.46E+00	5.31E-01	5.80E-03	2.13E-01
PPP2R5A	2.79E+02	7.74E-01	2.81E-01	5.87E-03	2.14E-01
LINC00494	1.06E+01	1.34E+00	4.87E-01	5.88E-03	2.14E-01
TFAP4	6.78E+01	-1.02E+00	3.72E-01	5.98E-03	2.17E-01
CDK5RAP1	2.49E+02	6.60E-01	2.41E-01	6.07E-03	2.19E-01
MET	3.43E+03	-1.14E+00	4.14E-01	6.09E-03	2.19E-01
NOTCH3	3.94E+03	1.06E+00	3.87E-01	6.09E-03	2.19E-01
SATB1	2.10E+02	-1.04E+00	3.77E-01	6.10E-03	2.19E-01
USP6	1.36E+01	-1.42E+00	5.18E-01	6.05E-03	2.19E-01
ARHGAP35	2.84E+03	6.87E-01	2.51E-01	6.25E-03	2.21E-01
EMC2	3.28E+02	-5.55E-01	2.03E-01	6.25E-03	2.21E-01
GOT2	8.33E+02	-6.34E-01	2.32E-01	6.24E-03	2.21E-01
KATNAL2	1.49E+02	-1.26E+00	4.61E-01	6.23E-03	2.21E-01
PLCL1	1.74E+02	1.11E+00	4.06E-01	6.26E-03	2.21E-01
RHBDL1	2.07E+01	-1.19E+00	4.36E-01	6.25E-03	2.21E-01
TRDMT1	2.01E+02	7.20E-01	2.63E-01	6.21E-03	2.21E-01
ATP5S	8.87E+01	7.80E-01	2.86E-01	6.42E-03	2.23E-01
CRTC3	5.87E+02	6.71E-01	2.46E-01	6.38E-03	2.23E-01
IGSF9B	3.41E+02	1.03E+00	3.78E-01	6.37E-03	2.23E-01
NAPB	2.87E+02	6.93E-01	2.54E-01	6.43E-03	2.23E-01
PSMA3	2.96E+02	6.85E-01	2.51E-01	6.34E-03	2.23E-01
RCAN1	3.41E+02	7.73E-01	2.84E-01	6.42E-03	2.23E-01
TREM2	1.92E+02	-1.16E+00	4.26E-01	6.43E-03	2.23E-01
CACNA2D3	1.18E+03	-1.19E+00	4.38E-01	6.54E-03	2.24E-01
CEND1	9.57E+01	-1.48E+00	5.45E-01	6.56E-03	2.24E-01
CHCHD5	8.58E+01	-5.77E-01	2.12E-01	6.54E-03	2.24E-01
ENTPD6	1.13E+03	9.64E-01	3.54E-01	6.47E-03	2.24E-01
FKBP4	5.64E+02	-8.92E-01	3.28E-01	6.52E-03	2.24E-01
LOC1019271	1.09E+01	1.13E+00	4.16E-01	6.52E-03	2.24E-01
PARP14	1.78E+03	3.35E-01	1.23E-01	6.47E-03	2.24E-01
SUPT20H	5.30E+02	4.69E-01	1.73E-01	6.56E-03	2.24E-01
TRMT44	2.01E+02	5.97E-01	2.19E-01	6.49E-03	2.24E-01
KRT8	6.50E+01	1.21E+00	4.45E-01	6.61E-03	2.25E-01
ALG1L9P	1.65E+01	1.05E+00	3.87E-01	6.72E-03	2.25E-01
C20orf196	1.44E+01	1.07E+00	3.93E-01	6.68E-03	2.25E-01
FAM174B	1.31E+02	1.29E+00	4.74E-01	6.69E-03	2.25E-01
IFNAR1	1.45E+03	4.67E-01	1.72E-01	6.71E-03	2.25E-01
OCLN	2.19E+02	1.05E+00	3.85E-01	6.68E-03	2.25E-01

SPR	7.87E+01	-9.90E-01	3.65E-01	6.73E-03	2.25E-01
STC2	2.47E+02	1.47E+00	5.43E-01	6.67E-03	2.25E-01
ZNF133	2.21E+02	6.93E-01	2.56E-01	6.73E-03	2.25E-01
KLHDC8B	2.45E+02	8.36E-01	3.09E-01	6.75E-03	2.25E-01
HGF	4.40E+01	-1.19E+00	4.41E-01	6.77E-03	2.25E-01
LSM14B	4.41E+02	7.66E-01	2.83E-01	6.81E-03	2.26E-01
MPP3	8.41E+01	1.07E+00	3.97E-01	6.82E-03	2.26E-01
GAA	7.32E+02	-7.01E-01	2.59E-01	6.84E-03	2.26E-01
ANK2	4.80E+03	-1.46E+00	5.39E-01	6.93E-03	2.27E-01
CIITA	3.44E+02	-1.19E+00	4.42E-01	6.97E-03	2.27E-01
CLIC4	3.66E+03	9.39E-01	3.47E-01	6.89E-03	2.27E-01
IQGAP3	1.24E+02	8.81E-01	3.26E-01	6.95E-03	2.27E-01
OIT3	3.83E+00	1.42E+00	5.28E-01	6.94E-03	2.27E-01
PPP1R1B	1.41E+02	1.47E+00	5.45E-01	6.97E-03	2.27E-01
PYGB	2.27E+03	9.25E-01	3.43E-01	6.95E-03	2.27E-01
TSR2	3.54E+02	5.79E-01	2.14E-01	6.90E-03	2.27E-01
ID2	8.00E+02	-7.59E-01	2.82E-01	7.00E-03	2.27E-01
NKD2	2.06E+01	-1.46E+00	5.41E-01	7.07E-03	2.28E-01
NOTCH4	1.52E+01	1.45E+00	5.39E-01	7.06E-03	2.28E-01
P4HA2	6.67E+02	8.05E-01	2.99E-01	7.07E-03	2.28E-01
ZNF436	4.21E+02	1.06E+00	3.95E-01	7.07E-03	2.28E-01
VPS4A	8.03E+02	-4.80E-01	1.78E-01	7.10E-03	2.28E-01
HEBP1	4.01E+02	-5.86E-01	2.18E-01	7.18E-03	2.29E-01
LINC00987	2.23E+01	-1.46E+00	5.41E-01	7.17E-03	2.29E-01
PLXNA4	7.75E+02	-1.16E+00	4.31E-01	7.13E-03	2.29E-01
PRDX4	3.49E+02	7.74E-01	2.88E-01	7.15E-03	2.29E-01
WWP1	9.07E+02	-8.42E-01	3.13E-01	7.17E-03	2.29E-01
FGD4	1.12E+03	8.52E-01	3.17E-01	7.24E-03	2.29E-01
PWAR1	4.37E+01	1.17E+00	4.37E-01	7.24E-03	2.29E-01
SMEK1	8.22E+02	6.61E-01	2.46E-01	7.21E-03	2.29E-01
GAMT	6.14E+01	-8.58E-01	3.20E-01	7.26E-03	2.29E-01
CDC20	2.37E+01	1.07E+00	4.01E-01	7.44E-03	2.33E-01
CHODL	7.05E+01	-1.46E+00	5.44E-01	7.40E-03	2.33E-01
DNAJC10	1.86E+03	-5.23E-01	1.95E-01	7.42E-03	2.33E-01
SLCO4A1	4.30E+01	1.37E+00	5.11E-01	7.44E-03	2.33E-01
WDR86	1.01E+03	-8.54E-01	3.19E-01	7.42E-03	2.33E-01
ECSIT	1.92E+02	-8.83E-01	3.30E-01	7.50E-03	2.34E-01
AMT	1.50E+02	-7.32E-01	2.74E-01	7.59E-03	2.34E-01
CCDC7	2.81E+02	8.49E-01	3.18E-01	7.60E-03	2.34E-01
CDC16	8.42E+02	5.19E-01	1.94E-01	7.54E-03	2.34E-01
LRP12	2.68E+02	-1.19E+00	4.45E-01	7.55E-03	2.34E-01
PCNX	2.03E+03	7.17E-01	2.68E-01	7.57E-03	2.34E-01
TRAF7	6.63E+02	-5.47E-01	2.05E-01	7.56E-03	2.34E-01
GBP5	3.80E+01	-1.36E+00	5.11E-01	7.65E-03	2.35E-01
PDE3A	5.08E+02	1.38E+00	5.18E-01	7.65E-03	2.35E-01
TENM2	4.43E+03	-1.41E+00	5.28E-01	7.64E-03	2.35E-01
ACSM3	3.87E+01	-1.27E+00	4.78E-01	7.75E-03	2.36E-01
CHCHD10	7.76E+01	-1.17E+00	4.41E-01	7.74E-03	2.36E-01
USP31	7.47E+02	1.00E+00	3.76E-01	7.72E-03	2.36E-01
ZNF829	9.27E+01	9.26E-01	3.48E-01	7.74E-03	2.36E-01
GSG2	1.51E+01	1.08E+00	4.05E-01	7.80E-03	2.36E-01
HNMT	4.97E+02	-6.94E-01	2.61E-01	7.81E-03	2.36E-01
SOX2-OT	2.37E+01	-1.43E+00	5.38E-01	7.78E-03	2.36E-01

CDC42EP2	3.04E+02	7.37E-01	2.77E-01	7.84E-03	2.37E-01
GSTM1	3.40E+01	-1.43E+00	5.39E-01	7.86E-03	2.37E-01
PAPOLA	1.79E+03	5.19E-01	1.95E-01	7.87E-03	2.37E-01
CPZ	1.70E+03	1.19E+00	4.49E-01	7.92E-03	2.38E-01
CNTN2	3.82E+01	-1.44E+00	5.41E-01	7.98E-03	2.39E-01
CUL1	7.99E+02	-3.81E-01	1.44E-01	7.99E-03	2.39E-01
PACS2	8.75E+02	6.84E-01	2.59E-01	8.14E-03	2.41E-01
PDGFRL	2.21E+02	-1.29E+00	4.86E-01	8.10E-03	2.41E-01
PTPN21	5.82E+02	8.20E-01	3.10E-01	8.11E-03	2.41E-01
SSBP2	6.78E+02	1.00E+00	3.79E-01	8.08E-03	2.41E-01
STK35	9.30E+02	1.08E+00	4.08E-01	8.14E-03	2.41E-01
USP46	3.65E+02	5.69E-01	2.15E-01	8.11E-03	2.41E-01
LOC1019273	2.75E+00	1.38E+00	5.21E-01	8.21E-03	2.42E-01
PTPRA	1.31E+03	7.12E-01	2.69E-01	8.21E-03	2.42E-01
SYCP2L	3.74E+01	-1.32E+00	5.00E-01	8.19E-03	2.42E-01
PDLIM7	8.68E+02	8.69E-01	3.29E-01	8.32E-03	2.44E-01
LAMA5	5.61E+03	1.01E+00	3.82E-01	8.34E-03	2.45E-01
YBX3	3.79E+03	-7.35E-01	2.79E-01	8.38E-03	2.45E-01
MRAP2	1.11E+01	1.44E+00	5.45E-01	8.41E-03	2.46E-01
ATG2B	1.02E+03	7.49E-01	2.84E-01	8.44E-03	2.46E-01
KPTN	3.93E+01	9.58E-01	3.64E-01	8.48E-03	2.47E-01
ATP7B	6.61E+02	1.31E+00	4.98E-01	8.52E-03	2.47E-01
BNIP3	2.00E+02	1.18E+00	4.47E-01	8.55E-03	2.47E-01
GBP2	2.10E+02	-9.85E-01	3.75E-01	8.56E-03	2.47E-01
PCDH18	5.11E+02	1.14E+00	4.35E-01	8.53E-03	2.47E-01
PRSS23	2.99E+02	1.21E+00	4.62E-01	8.54E-03	2.47E-01
NGLY1	2.51E+02	-5.98E-01	2.28E-01	8.58E-03	2.47E-01
AKAP13	8.75E+03	7.06E-01	2.69E-01	8.72E-03	2.47E-01
ARL8B	8.81E+02	-4.20E-01	1.60E-01	8.67E-03	2.47E-01
HEATR5A	1.06E+03	9.80E-01	3.74E-01	8.77E-03	2.47E-01
KCNJ8	5.33E+02	-1.43E+00	5.43E-01	8.63E-03	2.47E-01
LOC1005067	2.41E+01	-9.89E-01	3.77E-01	8.65E-03	2.47E-01
PKHD1L1	1.02E+01	-1.42E+00	5.42E-01	8.76E-03	2.47E-01
PLCH1	2.42E+01	-1.43E+00	5.45E-01	8.77E-03	2.47E-01
RNF145	1.40E+03	8.02E-01	3.06E-01	8.71E-03	2.47E-01
SRBD1	3.53E+02	-6.91E-01	2.63E-01	8.65E-03	2.47E-01
TENM3	7.34E+02	-1.39E+00	5.31E-01	8.68E-03	2.47E-01
TTYH2	2.82E+02	7.90E-01	3.01E-01	8.77E-03	2.47E-01
ZDHHC23	8.05E+01	-1.35E+00	5.16E-01	8.70E-03	2.47E-01
OGDHL	5.95E+02	-1.34E+00	5.12E-01	8.80E-03	2.48E-01
F2RL1	5.78E+01	1.37E+00	5.24E-01	8.82E-03	2.48E-01
MARCH3	6.97E+01	1.35E+00	5.17E-01	8.90E-03	2.50E-01
LINC01268	1.75E+01	-1.35E+00	5.17E-01	8.93E-03	2.50E-01
PCDHGA6	5.69E+02	8.44E-01	3.23E-01	8.95E-03	2.50E-01
LINC00842	1.18E+02	1.31E+00	5.03E-01	8.98E-03	2.50E-01
TLR7	1.21E+02	-1.14E+00	4.36E-01	9.04E-03	2.52E-01
LHFP	9.87E+02	1.28E+00	4.92E-01	9.06E-03	2.52E-01
TIFA	8.90E+01	-9.09E-01	3.48E-01	9.09E-03	2.52E-01
ZFYVE1	2.98E+02	7.06E-01	2.71E-01	9.09E-03	2.52E-01
ZNF837	1.49E+01	-1.01E+00	3.89E-01	9.11E-03	2.52E-01
DNM1P35	1.63E+01	-1.17E+00	4.49E-01	9.20E-03	2.53E-01
TNFAIP8	2.33E+02	-7.57E-01	2.91E-01	9.18E-03	2.53E-01
UVSSA	4.66E+02	7.69E-01	2.95E-01	9.20E-03	2.53E-01

WDR20	2.38E+02	6.33E-01	2.43E-01	9.19E-03	2.53E-01
DDX31	2.68E+02	7.18E-01	2.76E-01	9.23E-03	2.53E-01
LOC1001304	9.83E+00	1.37E+00	5.28E-01	9.23E-03	2.53E-01
MLH3	7.60E+02	6.41E-01	2.46E-01	9.26E-03	2.53E-01
PCYT1B	5.20E+01	-1.37E+00	5.28E-01	9.26E-03	2.53E-01
EML5	1.12E+02	1.00E+00	3.85E-01	9.31E-03	2.53E-01
GYPC	4.30E+02	-9.25E-01	3.56E-01	9.30E-03	2.53E-01
KIF21B	1.05E+02	-8.57E-01	3.30E-01	9.32E-03	2.53E-01
LOC90246	2.97E+01	-1.42E+00	5.45E-01	9.35E-03	2.53E-01
URI1	9.74E+02	4.16E-01	1.60E-01	9.39E-03	2.54E-01
ATP10D	1.11E+03	9.52E-01	3.67E-01	9.41E-03	2.54E-01
ARSD	6.30E+02	-5.77E-01	2.22E-01	9.46E-03	2.55E-01
PTPN9	8.73E+02	5.29E-01	2.04E-01	9.48E-03	2.55E-01
IRF1	2.30E+02	-7.52E-01	2.90E-01	9.50E-03	2.55E-01
PPBP	1.85E+01	1.37E+00	5.28E-01	9.52E-03	2.55E-01
KCNQ3	3.04E+02	-1.05E+00	4.05E-01	9.55E-03	2.55E-01
ROM1	2.23E+01	-1.10E+00	4.24E-01	9.55E-03	2.55E-01
ALG14	4.66E+01	8.75E-01	3.38E-01	9.61E-03	2.56E-01
HNRNPA1P3	1.66E+01	1.36E+00	5.27E-01	9.63E-03	2.56E-01
RAB11A	2.45E+03	6.39E-01	2.47E-01	9.64E-03	2.56E-01
SYT15	1.86E+02	1.22E+00	4.70E-01	9.61E-03	2.56E-01
ADAM22	1.70E+02	-1.31E+00	5.06E-01	9.78E-03	2.58E-01
NANP	1.59E+02	5.54E-01	2.14E-01	9.75E-03	2.58E-01
NDRG4	3.34E+01	-1.33E+00	5.13E-01	9.77E-03	2.58E-01
OSBP2	4.20E+01	1.09E+00	4.22E-01	9.75E-03	2.58E-01
TMX4	7.28E+02	6.69E-01	2.59E-01	9.88E-03	2.60E-01
N4BP3	1.60E+02	1.10E+00	4.26E-01	9.89E-03	2.60E-01
BCL2L1	1.64E+03	6.86E-01	2.66E-01	9.98E-03	2.60E-01
DNA2	5.56E+01	7.67E-01	2.98E-01	9.94E-03	2.60E-01
MOBP	4.49E+01	-1.37E+00	5.32E-01	9.98E-03	2.60E-01
NYAP1	4.51E+01	1.24E+00	4.81E-01	9.93E-03	2.60E-01
SCARA5	9.82E+03	-1.36E+00	5.27E-01	1.00E-02	2.60E-01
SEC23A	1.22E+03	7.78E-01	3.02E-01	1.00E-02	2.60E-01
SLC24A2	1.46E+01	-1.38E+00	5.37E-01	1.00E-02	2.60E-01
HSH2D	7.48E+00	1.24E+00	4.83E-01	1.01E-02	2.61E-01
EFCAB1	2.25E+01	-1.40E+00	5.44E-01	1.01E-02	2.61E-01
PPP1R16B	1.42E+02	1.03E+00	4.01E-01	1.01E-02	2.62E-01
MAOB	2.58E+01	-1.39E+00	5.41E-01	1.02E-02	2.63E-01
MAPRE1	1.46E+03	5.38E-01	2.10E-01	1.03E-02	2.65E-01
ERMARD	1.64E+02	6.48E-01	2.53E-01	1.03E-02	2.65E-01
GMFB	6.16E+02	6.05E-01	2.36E-01	1.04E-02	2.65E-01
KLHL3	4.57E+02	1.07E+00	4.17E-01	1.04E-02	2.65E-01
STXBP4	3.38E+02	-8.10E-01	3.16E-01	1.03E-02	2.65E-01
PIGU	2.23E+02	6.88E-01	2.68E-01	1.04E-02	2.65E-01
ADAT1	2.76E+02	-5.86E-01	2.29E-01	1.04E-02	2.66E-01
AP4S1	1.02E+02	1.12E+00	4.39E-01	1.04E-02	2.66E-01
ARSK	7.21E+01	7.93E-01	3.10E-01	1.05E-02	2.67E-01
TRAPPC10	1.23E+03	6.73E-01	2.63E-01	1.05E-02	2.67E-01
GAB2	5.16E+02	-6.85E-01	2.68E-01	1.06E-02	2.68E-01
TMSB4Y	4.84E+00	1.21E+00	4.73E-01	1.06E-02	2.68E-01
TNNC2	6.32E+00	1.25E+00	4.90E-01	1.06E-02	2.69E-01
FBLL1	2.52E+01	-1.35E+00	5.30E-01	1.07E-02	2.71E-01
GPC5-AS1	1.08E+02	-1.37E+00	5.36E-01	1.08E-02	2.71E-01

LINC00948	7.99E+00	-1.35E+00	5.29E-01	1.08E-02	2.71E-01
ZNF488	2.17E+01	1.27E+00	4.98E-01	1.08E-02	2.71E-01
CDC42BPB	2.39E+03	8.94E-01	3.51E-01	1.09E-02	2.71E-01
DNASE1L3	4.04E+02	-1.36E+00	5.34E-01	1.09E-02	2.71E-01
MEIS2	8.50E+01	1.06E+00	4.17E-01	1.09E-02	2.71E-01
NDUFAF4	1.11E+02	-1.01E+00	3.95E-01	1.09E-02	2.71E-01
PCDHAC2	9.33E+01	1.28E+00	5.02E-01	1.09E-02	2.71E-01
UQCRC2	1.35E+03	-8.51E-01	3.34E-01	1.09E-02	2.71E-01
BRF1	2.75E+02	7.31E-01	2.87E-01	1.09E-02	2.72E-01
BDKRB2	8.00E+01	1.38E+00	5.42E-01	1.10E-02	2.72E-01
LGALS12	3.21E+00	1.39E+00	5.45E-01	1.10E-02	2.72E-01
PLAGL2	3.39E+02	7.78E-01	3.06E-01	1.10E-02	2.72E-01
RNU6ATAC	4.57E+00	1.38E+00	5.45E-01	1.10E-02	2.72E-01
SRSF5	2.09E+03	7.28E-01	2.86E-01	1.10E-02	2.72E-01
TFF3	2.36E+01	1.39E+00	5.45E-01	1.10E-02	2.72E-01
KIF26B	1.97E+01	-1.27E+00	5.00E-01	1.11E-02	2.72E-01
SNTG2	1.71E+01	1.31E+00	5.18E-01	1.13E-02	2.78E-01
CHSY1	1.83E+03	1.15E+00	4.53E-01	1.14E-02	2.78E-01
MCC	6.09E+02	1.29E+00	5.09E-01	1.14E-02	2.78E-01
MT3	4.97E+01	-1.33E+00	5.26E-01	1.14E-02	2.78E-01
AP1G2	5.06E+02	9.65E-01	3.81E-01	1.14E-02	2.78E-01
C11orf70	3.70E+01	-9.25E-01	3.66E-01	1.14E-02	2.78E-01
ASXL2	1.03E+03	-5.07E-01	2.01E-01	1.17E-02	2.79E-01
BLOC1S2	1.70E+02	5.48E-01	2.17E-01	1.15E-02	2.79E-01
CLN6	2.91E+02	3.71E-01	1.47E-01	1.15E-02	2.79E-01
COX16	5.91E+01	7.07E-01	2.80E-01	1.15E-02	2.79E-01
DBNDD2	9.64E+01	1.09E+00	4.34E-01	1.17E-02	2.79E-01
E2F1	1.81E+02	8.84E-01	3.50E-01	1.16E-02	2.79E-01
EPS8	6.95E+02	1.15E+00	4.55E-01	1.16E-02	2.79E-01
GABARAPL1	1.93E+03	-7.75E-01	3.07E-01	1.16E-02	2.79E-01
GPRC5B	2.56E+02	1.23E+00	4.88E-01	1.15E-02	2.79E-01
IER5	2.59E+02	7.79E-01	3.09E-01	1.16E-02	2.79E-01
LINC00852	3.98E+01	-1.01E+00	4.00E-01	1.17E-02	2.79E-01
PCBP4	3.74E+02	-7.74E-01	3.06E-01	1.16E-02	2.79E-01
RAB3D	1.33E+03	9.35E-01	3.70E-01	1.15E-02	2.79E-01
RAPGEF5	6.76E+02	1.12E+00	4.44E-01	1.17E-02	2.79E-01
TMEM251	6.20E+01	8.65E-01	3.43E-01	1.17E-02	2.79E-01
UACA	3.40E+03	7.80E-01	3.09E-01	1.17E-02	2.79E-01
UBE2H	1.76E+03	7.34E-01	2.91E-01	1.17E-02	2.79E-01
KCNK13	1.69E+01	-1.23E+00	4.89E-01	1.18E-02	2.80E-01
ARID2	1.67E+03	4.38E-01	1.74E-01	1.19E-02	2.80E-01
DFNB31	1.84E+02	-9.53E-01	3.79E-01	1.19E-02	2.80E-01
MRPS25	9.13E+02	-8.27E-01	3.29E-01	1.19E-02	2.80E-01
CAPG	4.04E+02	-8.42E-01	3.35E-01	1.19E-02	2.81E-01
RRM2	1.09E+02	8.40E-01	3.34E-01	1.20E-02	2.81E-01
KIAA1328	2.01E+02	6.88E-01	2.74E-01	1.20E-02	2.82E-01
VSIG10L	7.22E+01	1.23E+00	4.89E-01	1.20E-02	2.82E-01
CHMP4A	1.82E+02	8.05E-01	3.20E-01	1.20E-02	2.82E-01
DTX2	1.43E+02	-5.46E-01	2.18E-01	1.21E-02	2.82E-01
KLF3	2.64E+03	-8.51E-01	3.39E-01	1.21E-02	2.82E-01
TMEM237	4.54E+02	-8.13E-01	3.24E-01	1.21E-02	2.82E-01
C9orf78	4.12E+02	-3.15E-01	1.26E-01	1.23E-02	2.84E-01
FOLR1	4.16E+01	-1.35E+00	5.40E-01	1.23E-02	2.84E-01

MOCOS	2.57E+01	-1.26E+00	5.01E-01	1.22E-02	2.84E-01
SYK	4.07E+02	-7.52E-01	3.00E-01	1.22E-02	2.84E-01
CCNK	3.20E+02	7.95E-01	3.18E-01	1.23E-02	2.85E-01
CFI	9.89E+02	9.77E-01	3.90E-01	1.23E-02	2.85E-01
USP27X	2.04E+01	8.54E-01	3.41E-01	1.24E-02	2.86E-01
GAS6-AS2	2.72E+01	1.33E+00	5.34E-01	1.24E-02	2.86E-01
MFSD1	7.57E+02	-4.95E-01	1.98E-01	1.24E-02	2.86E-01
NKAPL	1.97E+01	1.36E+00	5.44E-01	1.25E-02	2.86E-01
TGM1	2.34E+02	8.91E-01	3.56E-01	1.25E-02	2.86E-01
SPAG1	5.32E+01	-1.13E+00	4.51E-01	1.25E-02	2.87E-01
ID4	1.74E+03	-1.09E+00	4.36E-01	1.25E-02	2.87E-01
POLR3F	1.64E+02	6.78E-01	2.72E-01	1.26E-02	2.87E-01
AKT1	1.32E+03	6.85E-01	2.75E-01	1.27E-02	2.89E-01
CIPC	3.50E+02	7.33E-01	2.94E-01	1.27E-02	2.89E-01
EHD2	1.84E+03	7.44E-01	2.98E-01	1.27E-02	2.89E-01
NQO1	5.86E+02	8.11E-01	3.25E-01	1.27E-02	2.89E-01
TRAF3IP3	2.18E+01	-1.13E+00	4.52E-01	1.27E-02	2.89E-01
AKAP12	1.93E+04	1.15E+00	4.62E-01	1.28E-02	2.90E-01
SEMA5A	6.04E+02	1.23E+00	4.93E-01	1.28E-02	2.90E-01
B4GALT5	1.03E+03	7.95E-01	3.20E-01	1.29E-02	2.91E-01
POMT2	1.35E+02	5.70E-01	2.29E-01	1.29E-02	2.91E-01
POLE2	1.86E+01	1.12E+00	4.49E-01	1.30E-02	2.92E-01
TBC1D20	7.15E+02	7.46E-01	3.00E-01	1.30E-02	2.92E-01
TMTC2	3.41E+01	1.01E+00	4.07E-01	1.30E-02	2.92E-01
ZNF706	3.61E+02	-5.62E-01	2.26E-01	1.30E-02	2.92E-01
PSMA6	4.44E+02	5.89E-01	2.37E-01	1.32E-02	2.95E-01
ABCA2	1.03E+03	-7.55E-01	3.05E-01	1.33E-02	2.95E-01
CCSER2	1.09E+03	6.16E-01	2.49E-01	1.32E-02	2.95E-01
CNKSR2	1.93E+01	1.35E+00	5.44E-01	1.33E-02	2.95E-01
LOC613037	1.50E+01	9.98E-01	4.03E-01	1.32E-02	2.95E-01
MTERFD1	1.26E+02	-6.45E-01	2.60E-01	1.33E-02	2.95E-01
PARP3	2.47E+02	-1.01E+00	4.06E-01	1.33E-02	2.95E-01
SRGAP1	6.31E+02	1.02E+00	4.11E-01	1.33E-02	2.95E-01
UGCG	2.59E+02	9.18E-01	3.71E-01	1.32E-02	2.95E-01
ZNF410	2.93E+02	6.37E-01	2.58E-01	1.34E-02	2.96E-01
ANO1	4.72E+01	1.18E+00	4.78E-01	1.34E-02	2.96E-01
MNAT1	2.28E+02	7.97E-01	3.22E-01	1.35E-02	2.96E-01
SLC39A3	1.77E+02	-7.23E-01	2.92E-01	1.34E-02	2.96E-01
STRN3	4.82E+02	8.07E-01	3.26E-01	1.34E-02	2.96E-01
APCDD1L-AS	4.12E+00	1.24E+00	5.03E-01	1.35E-02	2.96E-01
ENDOU	1.59E+01	1.22E+00	4.96E-01	1.36E-02	2.98E-01
FBF1	2.24E+02	-4.91E-01	1.99E-01	1.36E-02	2.98E-01
SIAH3	3.77E+00	1.34E+00	5.45E-01	1.36E-02	2.98E-01
FFAR4	9.52E+00	-1.29E+00	5.23E-01	1.36E-02	2.98E-01
ACO2	1.20E+03	-7.59E-01	3.08E-01	1.37E-02	2.99E-01
ADAMTS17	7.32E+02	1.15E+00	4.67E-01	1.37E-02	2.99E-01
SVIL	1.82E+03	1.15E+00	4.68E-01	1.37E-02	2.99E-01
THAP2	4.96E+01	-6.60E-01	2.68E-01	1.37E-02	2.99E-01
CDK2	3.69E+02	-4.34E-01	1.76E-01	1.38E-02	2.99E-01
ZC3H12C	5.80E+02	5.04E-01	2.05E-01	1.38E-02	2.99E-01
SLC38A10	1.38E+03	-5.37E-01	2.18E-01	1.38E-02	2.99E-01
CTGLF12P	2.27E+01	-1.27E+00	5.15E-01	1.39E-02	3.00E-01
ABCC1	3.07E+02	1.14E+00	4.63E-01	1.40E-02	3.00E-01

ABI3BP	2.23E+03	-1.24E+00	5.06E-01	1.41E-02	3.00E-01
FAM179A	2.99E+01	-1.16E+00	4.71E-01	1.40E-02	3.00E-01
FAM84A	9.46E+01	1.23E+00	5.02E-01	1.41E-02	3.00E-01
JAKMIP3	4.76E+01	1.05E+00	4.29E-01	1.39E-02	3.00E-01
LAMA3	6.82E+01	1.29E+00	5.25E-01	1.40E-02	3.00E-01
MMP15	2.03E+02	1.01E+00	4.09E-01	1.40E-02	3.00E-01
OGDH	2.32E+03	-7.28E-01	2.96E-01	1.40E-02	3.00E-01
WDR89	1.08E+02	6.26E-01	2.55E-01	1.40E-02	3.00E-01
ZNF534	7.39E+00	1.33E+00	5.41E-01	1.41E-02	3.00E-01
ZNF771	3.68E+01	-9.07E-01	3.69E-01	1.41E-02	3.00E-01
CCDC77	7.63E+01	8.02E-01	3.27E-01	1.41E-02	3.01E-01
HYAL1	8.82E+01	-1.32E+00	5.37E-01	1.42E-02	3.01E-01
ZSCAN12P1	4.50E+01	-1.32E+00	5.37E-01	1.42E-02	3.01E-01
CA13	4.41E+01	-1.24E+00	5.07E-01	1.42E-02	3.02E-01
FOXD3	3.55E+01	-1.29E+00	5.25E-01	1.43E-02	3.02E-01
PRRT2	1.00E+02	-1.01E+00	4.14E-01	1.43E-02	3.02E-01
MCU	4.26E+02	9.93E-01	4.06E-01	1.44E-02	3.03E-01
PLTP	2.56E+03	1.05E+00	4.28E-01	1.44E-02	3.03E-01
TPH1	1.20E+01	1.23E+00	5.01E-01	1.44E-02	3.03E-01
CAV2	8.02E+02	-1.09E+00	4.48E-01	1.45E-02	3.04E-01
CSTF1	3.61E+02	5.52E-01	2.26E-01	1.45E-02	3.04E-01
ZFYVE26	8.76E+02	6.29E-01	2.57E-01	1.45E-02	3.04E-01
FAM46B	4.19E+00	1.33E+00	5.43E-01	1.45E-02	3.04E-01
MASTL	1.53E+02	5.75E-01	2.36E-01	1.46E-02	3.05E-01
RNF31	5.31E+02	5.86E-01	2.40E-01	1.46E-02	3.05E-01
ZC3H18	4.58E+02	-3.42E-01	1.40E-01	1.46E-02	3.05E-01
IFIT5	3.64E+02	5.74E-01	2.35E-01	1.47E-02	3.05E-01
MYL3	6.19E+00	-1.31E+00	5.36E-01	1.47E-02	3.05E-01
PSEN1	8.92E+02	7.01E-01	2.88E-01	1.48E-02	3.07E-01
AXL	9.66E+02	-9.21E-01	3.78E-01	1.48E-02	3.07E-01
SEMA3A	1.58E+01	1.32E+00	5.43E-01	1.49E-02	3.07E-01
SH2D4A	6.86E+01	-1.18E+00	4.86E-01	1.48E-02	3.07E-01
TMTC1	4.82E+03	9.34E-01	3.84E-01	1.49E-02	3.07E-01
ZBED5-AS1	5.11E+01	-9.29E-01	3.81E-01	1.49E-02	3.07E-01
ETNK1	1.20E+03	-5.72E-01	2.35E-01	1.49E-02	3.07E-01
TRAF3	5.01E+02	5.51E-01	2.26E-01	1.49E-02	3.08E-01
DLG5	9.98E+02	6.30E-01	2.59E-01	1.50E-02	3.08E-01
KLF7	8.22E+02	5.64E-01	2.32E-01	1.50E-02	3.08E-01
PHGDH	1.23E+02	9.74E-01	4.01E-01	1.51E-02	3.09E-01
CDH6	1.55E+02	1.21E+00	5.00E-01	1.52E-02	3.09E-01
INHBA	1.62E+01	1.20E+00	4.92E-01	1.52E-02	3.09E-01
LMF1	2.70E+02	-6.65E-01	2.74E-01	1.52E-02	3.09E-01
LOC1019269	1.17E+02	1.00E+00	4.12E-01	1.51E-02	3.09E-01
SLC9A8	4.20E+02	6.10E-01	2.51E-01	1.52E-02	3.09E-01
SLCO1A2	7.21E+00	-1.31E+00	5.39E-01	1.51E-02	3.09E-01
TRABD2B	5.52E+02	-9.43E-01	3.88E-01	1.52E-02	3.09E-01
GALNT3	7.88E+01	-1.32E+00	5.45E-01	1.54E-02	3.12E-01
LYPD6B	8.81E+01	-1.29E+00	5.34E-01	1.54E-02	3.13E-01
ATP8B5P	6.03E+00	1.12E+00	4.64E-01	1.55E-02	3.13E-01
GPR65	5.84E+01	-1.18E+00	4.89E-01	1.56E-02	3.13E-01
HBA1	1.24E+02	1.29E+00	5.32E-01	1.55E-02	3.13E-01
PAM	2.89E+03	6.84E-01	2.83E-01	1.56E-02	3.13E-01
PTPN1	7.55E+02	6.73E-01	2.78E-01	1.55E-02	3.13E-01

SLC25A32	2.61E+02	-5.07E-01	2.10E-01	1.56E-02	3.13E-01
SMG1P7	2.72E+01	-1.11E+00	4.57E-01	1.56E-02	3.13E-01
GADD45A	1.02E+02	1.12E+00	4.62E-01	1.57E-02	3.14E-01
FKBP9	2.21E+03	-5.74E-01	2.38E-01	1.57E-02	3.15E-01
GPATCH2L	1.00E+03	5.62E-01	2.33E-01	1.57E-02	3.15E-01
SLC39A9	1.38E+03	6.20E-01	2.57E-01	1.58E-02	3.15E-01
EXD2	2.70E+02	6.86E-01	2.84E-01	1.58E-02	3.15E-01
GNLY	6.31E+00	1.27E+00	5.28E-01	1.58E-02	3.16E-01
GGT5	1.60E+02	1.19E+00	4.92E-01	1.59E-02	3.16E-01
RNF115	4.11E+02	4.92E-01	2.04E-01	1.59E-02	3.17E-01
ARHGEF17	1.99E+03	8.91E-01	3.70E-01	1.61E-02	3.17E-01
COQ9	5.87E+02	-8.79E-01	3.65E-01	1.61E-02	3.17E-01
EFEMP1	6.54E+03	-1.07E+00	4.44E-01	1.60E-02	3.17E-01
KLF3-AS1	1.94E+02	-7.34E-01	3.05E-01	1.61E-02	3.17E-01
MKL2	2.08E+03	9.68E-01	4.02E-01	1.61E-02	3.17E-01
PNPO	5.33E+02	-5.74E-01	2.38E-01	1.60E-02	3.17E-01
SYNGR3	2.78E+01	-1.28E+00	5.31E-01	1.60E-02	3.17E-01
IL17RA	6.35E+02	-8.71E-01	3.62E-01	1.61E-02	3.17E-01
IQUB	2.77E+01	-1.11E+00	4.60E-01	1.61E-02	3.17E-01
DOCK9	1.63E+03	1.01E+00	4.22E-01	1.62E-02	3.18E-01
ERCC1	7.47E+02	-5.41E-01	2.25E-01	1.62E-02	3.18E-01
PRKCH	5.68E+02	6.71E-01	2.79E-01	1.62E-02	3.18E-01
DNAL1	3.14E+02	8.18E-01	3.40E-01	1.62E-02	3.18E-01
PLEKHO1	2.25E+02	-6.97E-01	2.90E-01	1.63E-02	3.18E-01
PTDSS1	8.19E+02	-6.33E-01	2.64E-01	1.63E-02	3.18E-01
DLEU7	4.58E+00	-1.28E+00	5.32E-01	1.64E-02	3.18E-01
EVI2A	1.13E+02	-1.09E+00	4.54E-01	1.64E-02	3.18E-01
KLRD1	4.21E+01	-1.28E+00	5.33E-01	1.64E-02	3.18E-01
MYCBP2	3.37E+03	4.50E-01	1.88E-01	1.64E-02	3.18E-01
SLC6A17	1.28E+01	-1.31E+00	5.45E-01	1.64E-02	3.18E-01
TOX4	8.91E+02	6.19E-01	2.58E-01	1.64E-02	3.18E-01
YY1	6.88E+02	5.13E-01	2.14E-01	1.65E-02	3.19E-01
S100A14	8.73E+00	1.30E+00	5.41E-01	1.65E-02	3.19E-01
ARX	1.40E+01	-1.28E+00	5.36E-01	1.66E-02	3.19E-01
IFI27L2	8.23E+01	9.99E-01	4.17E-01	1.66E-02	3.19E-01
LOC1019275	2.82E+01	-1.30E+00	5.43E-01	1.66E-02	3.20E-01
CABLES1	1.22E+02	1.15E+00	4.81E-01	1.66E-02	3.20E-01
CD84	4.65E+02	-9.76E-01	4.08E-01	1.67E-02	3.21E-01
SUMO3	8.36E+02	4.66E-01	1.95E-01	1.67E-02	3.21E-01
SEZ6L2	5.06E+02	-1.15E+00	4.82E-01	1.67E-02	3.21E-01
SETD3	7.62E+02	7.62E-01	3.19E-01	1.68E-02	3.23E-01
ITPRIP	4.19E+02	9.81E-01	4.11E-01	1.69E-02	3.24E-01
OTUB2	8.47E+00	1.10E+00	4.61E-01	1.70E-02	3.25E-01
SYT6	1.71E+01	-1.22E+00	5.13E-01	1.70E-02	3.25E-01
AGFG2	4.32E+02	6.71E-01	2.81E-01	1.71E-02	3.25E-01
NAV3	5.63E+01	-1.27E+00	5.33E-01	1.71E-02	3.25E-01
CASP6	7.85E+01	7.58E-01	3.18E-01	1.71E-02	3.26E-01
LINC00176	4.87E+01	1.05E+00	4.39E-01	1.72E-02	3.26E-01
SDCBP	3.32E+03	-6.92E-01	2.90E-01	1.72E-02	3.26E-01
FASTKD1	2.44E+02	-7.47E-01	3.14E-01	1.73E-02	3.27E-01
KCNB1	2.14E+02	1.18E+00	4.96E-01	1.73E-02	3.28E-01
RFC3	1.15E+02	7.23E-01	3.04E-01	1.73E-02	3.28E-01
CDH3	2.92E+02	-1.05E+00	4.43E-01	1.74E-02	3.28E-01

HCG27	6.55E+00	-1.27E+00	5.32E-01	1.74E-02	3.28E-01
PLCG1	2.20E+03	6.13E-01	2.58E-01	1.74E-02	3.28E-01
STAG3L5P	2.89E+01	7.88E-01	3.31E-01	1.74E-02	3.28E-01
FAM65A	6.88E+02	-4.21E-01	1.77E-01	1.75E-02	3.29E-01
TMOD3	3.69E+03	8.01E-01	3.38E-01	1.77E-02	3.32E-01
ELOVL2	2.34E+02	-1.10E+00	4.65E-01	1.78E-02	3.33E-01
RAB14	1.59E+03	-3.61E-01	1.53E-01	1.79E-02	3.35E-01
SLC7A5	2.48E+02	-1.09E+00	4.61E-01	1.79E-02	3.35E-01
SLC44A3	4.30E+01	-1.21E+00	5.11E-01	1.80E-02	3.35E-01
C14orf169	1.61E+02	7.34E-01	3.10E-01	1.81E-02	3.37E-01
SPATA18	1.03E+02	-1.28E+00	5.40E-01	1.81E-02	3.37E-01
CSDC2	1.07E+02	1.02E+00	4.31E-01	1.83E-02	3.38E-01
DGCR5	7.68E+01	-9.38E-01	3.97E-01	1.82E-02	3.38E-01
ERH	5.56E+02	6.46E-01	2.73E-01	1.82E-02	3.38E-01
FAM92A1	1.95E+02	-6.84E-01	2.90E-01	1.83E-02	3.38E-01
MICU2	5.73E+02	4.75E-01	2.01E-01	1.82E-02	3.38E-01
MIS18BP1	4.33E+02	4.86E-01	2.06E-01	1.82E-02	3.38E-01
PSMB10	1.59E+02	-6.56E-01	2.78E-01	1.82E-02	3.38E-01
RTN1	8.47E+01	-9.95E-01	4.22E-01	1.83E-02	3.38E-01
ATAD5	1.12E+02	6.26E-01	2.65E-01	1.83E-02	3.38E-01
APBB1IP	1.78E+02	-1.02E+00	4.35E-01	1.85E-02	3.40E-01
NGDN	1.49E+02	7.33E-01	3.11E-01	1.85E-02	3.40E-01
SLC35E3	1.61E+02	6.37E-01	2.71E-01	1.85E-02	3.40E-01
GNE	4.87E+02	-5.63E-01	2.39E-01	1.86E-02	3.40E-01
PTH1R	4.90E+01	-1.12E+00	4.75E-01	1.86E-02	3.40E-01
RBM20	2.26E+01	1.26E+00	5.34E-01	1.85E-02	3.40E-01
COMMD10	1.38E+02	6.09E-01	2.59E-01	1.87E-02	3.41E-01
SCNN1A	2.80E+02	1.12E+00	4.75E-01	1.87E-02	3.41E-01
ZNF268	4.23E+02	6.27E-01	2.67E-01	1.87E-02	3.41E-01
MDGA1	4.06E+02	9.64E-01	4.10E-01	1.87E-02	3.41E-01
SCIN	9.87E+01	-1.20E+00	5.11E-01	1.88E-02	3.41E-01
SNTB1	1.17E+02	1.21E+00	5.16E-01	1.88E-02	3.41E-01
CRYAB	4.27E+02	-8.86E-01	3.77E-01	1.88E-02	3.41E-01
LHX4	4.86E+00	1.26E+00	5.35E-01	1.89E-02	3.43E-01
TTC39B	2.72E+02	-5.74E-01	2.45E-01	1.90E-02	3.44E-01
COL4A1	2.90E+03	1.13E+00	4.83E-01	1.90E-02	3.45E-01
CRISPLD2	8.56E+01	1.27E+00	5.43E-01	1.91E-02	3.45E-01
BCKDHB	3.51E+02	-5.66E-01	2.42E-01	1.94E-02	3.45E-01
CAMKK1	7.74E+01	1.12E+00	4.79E-01	1.93E-02	3.45E-01
ITCH	1.32E+03	5.03E-01	2.15E-01	1.92E-02	3.45E-01
LINC00087	4.60E+01	1.11E+00	4.74E-01	1.94E-02	3.45E-01
LINC01132	6.66E+00	1.16E+00	4.94E-01	1.92E-02	3.45E-01
MED29	8.62E+02	3.87E-01	1.66E-01	1.93E-02	3.45E-01
OR51I1	1.38E+01	1.16E+00	4.96E-01	1.94E-02	3.45E-01
PDLIM1	4.06E+02	1.20E+00	5.12E-01	1.92E-02	3.45E-01
PROS1	1.95E+03	-9.08E-01	3.88E-01	1.92E-02	3.45E-01
RARRES1	5.25E+00	1.27E+00	5.42E-01	1.93E-02	3.45E-01
SNHG17	9.21E+01	-6.22E-01	2.66E-01	1.93E-02	3.45E-01
SURF6	3.51E+02	-3.99E-01	1.70E-01	1.91E-02	3.45E-01
TAX1BP3	8.64E+01	6.35E-01	2.72E-01	1.94E-02	3.45E-01
ZDHHC20	7.06E+02	4.64E-01	1.98E-01	1.93E-02	3.45E-01
QPRT	2.25E+02	-1.08E+00	4.64E-01	1.96E-02	3.48E-01
UCN	5.78E+00	-1.25E+00	5.37E-01	1.96E-02	3.48E-01

WDR19	5.43E+02	-7.84E-01	3.36E-01	1.96E-02	3.48E-01
GPAM	2.36E+02	6.61E-01	2.83E-01	1.97E-02	3.49E-01
KLC1	1.15E+03	6.55E-01	2.81E-01	1.97E-02	3.49E-01
WDR37	4.02E+02	5.20E-01	2.23E-01	1.97E-02	3.49E-01
BAALC	1.22E+02	-1.27E+00	5.43E-01	1.98E-02	3.49E-01
ATP6V1E2	1.00E+01	-1.15E+00	4.94E-01	1.99E-02	3.51E-01
CXCL12	8.22E+02	1.23E+00	5.30E-01	2.01E-02	3.52E-01
IFI27L1	2.78E+01	8.83E-01	3.80E-01	2.01E-02	3.52E-01
LOC1019270	4.52E+01	7.55E-01	3.25E-01	2.01E-02	3.52E-01
NRG4	4.42E+01	-1.21E+00	5.22E-01	2.01E-02	3.52E-01
PDZRN4	1.03E+01	-1.23E+00	5.30E-01	2.00E-02	3.52E-01
RBM3	1.81E+03	4.77E-01	2.05E-01	2.01E-02	3.52E-01
SUPT16H	1.29E+03	6.31E-01	2.72E-01	2.02E-02	3.52E-01
TBCK	7.36E+02	-5.28E-01	2.27E-01	2.00E-02	3.52E-01
TRIM6	1.85E+02	9.74E-01	4.19E-01	2.01E-02	3.52E-01
SLC19A2	2.49E+02	-8.55E-01	3.68E-01	2.02E-02	3.52E-01
NFATC2	2.37E+02	-7.62E-01	3.28E-01	2.02E-02	3.52E-01
ZNF358	4.29E+02	-5.97E-01	2.57E-01	2.02E-02	3.52E-01
C10orf111	3.34E+00	1.26E+00	5.42E-01	2.03E-02	3.53E-01
STXBP5L	8.79E+00	-1.25E+00	5.39E-01	2.03E-02	3.53E-01
ATL1	2.99E+02	8.61E-01	3.71E-01	2.05E-02	3.54E-01
C9orf106	1.68E+01	1.01E+00	4.36E-01	2.04E-02	3.54E-01
ESR2	2.93E+01	-1.20E+00	5.19E-01	2.05E-02	3.54E-01
C15orf37	8.15E+00	-1.06E+00	4.58E-01	2.05E-02	3.55E-01
PCNA	4.95E+02	6.24E-01	2.70E-01	2.06E-02	3.56E-01
NDUFA8	2.67E+02	-6.67E-01	2.88E-01	2.07E-02	3.57E-01
HLA-DRB5	1.43E+02	-1.24E+00	5.36E-01	2.07E-02	3.57E-01
NXN	6.84E+02	9.03E-01	3.90E-01	2.07E-02	3.57E-01
PRKG1	2.18E+02	-1.03E+00	4.44E-01	2.07E-02	3.57E-01
TLR5	8.23E+01	-1.01E+00	4.38E-01	2.09E-02	3.59E-01
HBA2	3.34E+02	1.23E+00	5.31E-01	2.09E-02	3.59E-01
RASA1	9.34E+02	4.15E-01	1.80E-01	2.09E-02	3.59E-01
C16orf58	8.52E+02	-5.36E-01	2.32E-01	2.10E-02	3.59E-01
CHTF8	6.40E+02	-4.37E-01	1.89E-01	2.10E-02	3.59E-01
MPP6	6.24E+02	9.54E-01	4.14E-01	2.10E-02	3.59E-01
OLFML2B	2.16E+03	-9.10E-01	3.94E-01	2.10E-02	3.59E-01
AMER2	1.70E+01	-1.24E+00	5.36E-01	2.11E-02	3.59E-01
LOC1002681	5.72E+00	1.16E+00	5.04E-01	2.11E-02	3.59E-01
SBF2	1.41E+03	4.90E-01	2.12E-01	2.11E-02	3.59E-01
TMED8	6.75E+01	6.81E-01	2.95E-01	2.11E-02	3.59E-01
C15orf52	5.67E+02	7.84E-01	3.41E-01	2.12E-02	3.60E-01
CD59	5.64E+03	7.51E-01	3.26E-01	2.12E-02	3.60E-01
MAP3K10	1.07E+02	5.21E-01	2.26E-01	2.13E-02	3.60E-01
MYD88	3.62E+02	-5.46E-01	2.37E-01	2.13E-02	3.60E-01
MYOF	1.48E+03	6.97E-01	3.02E-01	2.12E-02	3.60E-01
CAMK2D	1.44E+03	9.80E-01	4.28E-01	2.20E-02	3.62E-01
CEP55	4.26E+01	8.58E-01	3.74E-01	2.19E-02	3.62E-01
CISH	3.20E+01	-9.12E-01	3.97E-01	2.15E-02	3.62E-01
DLG3	4.74E+02	8.92E-01	3.88E-01	2.17E-02	3.62E-01
DYNLRB1	8.60E+02	6.91E-01	3.01E-01	2.18E-02	3.62E-01
ELL3	2.37E+01	1.00E+00	4.37E-01	2.16E-02	3.62E-01
EMILIN1	6.18E+02	-1.03E+00	4.48E-01	2.18E-02	3.62E-01
FAM81A	2.06E+01	-1.07E+00	4.68E-01	2.18E-02	3.62E-01

FBXO46	1.81E+02	4.32E-01	1.89E-01	2.18E-02	3.62E-01
ISM1	6.27E+01	1.08E+00	4.70E-01	2.17E-02	3.62E-01
KIAA1683	2.05E+02	1.17E+00	5.08E-01	2.15E-02	3.62E-01
LINC01272	4.04E+00	1.22E+00	5.32E-01	2.19E-02	3.62E-01
MOAP1	2.96E+02	7.39E-01	3.22E-01	2.19E-02	3.62E-01
NMNAT2	4.25E+03	-9.72E-01	4.24E-01	2.18E-02	3.62E-01
PSD4	6.23E+02	-6.02E-01	2.62E-01	2.15E-02	3.62E-01
PTAFR	1.79E+02	-1.06E+00	4.63E-01	2.17E-02	3.62E-01
PTGER3	5.78E+00	1.23E+00	5.37E-01	2.20E-02	3.62E-01
RGS20	7.34E+00	-1.23E+00	5.38E-01	2.18E-02	3.62E-01
RIMKLA	2.77E+01	1.22E+00	5.29E-01	2.16E-02	3.62E-01
SLC25A12	5.19E+02	-7.59E-01	3.30E-01	2.14E-02	3.62E-01
SSBP3	5.51E+02	7.90E-01	3.44E-01	2.15E-02	3.62E-01
SYTL4	8.24E+01	1.20E+00	5.23E-01	2.16E-02	3.62E-01
TPGS2	4.64E+02	6.40E-01	2.79E-01	2.19E-02	3.62E-01
YLPM1	1.60E+03	6.28E-01	2.73E-01	2.16E-02	3.62E-01
ZFX	1.56E+03	6.32E-01	2.76E-01	2.18E-02	3.62E-01
ZNF429	1.86E+02	6.71E-01	2.92E-01	2.17E-02	3.62E-01
GALNT5	2.94E+01	-1.23E+00	5.38E-01	2.20E-02	3.62E-01
SNX25	9.45E+02	8.55E-01	3.74E-01	2.20E-02	3.62E-01
CXCL2	7.46E+00	-1.25E+00	5.44E-01	2.21E-02	3.62E-01
INPP5A	2.43E+02	7.95E-01	3.47E-01	2.21E-02	3.62E-01
WHAMMP2	8.88E+01	7.38E-01	3.22E-01	2.21E-02	3.62E-01
G6PD	7.84E+02	9.35E-01	4.09E-01	2.22E-02	3.62E-01
PARD6B	1.24E+02	1.13E+00	4.92E-01	2.22E-02	3.62E-01
PYROXD1	2.54E+02	6.48E-01	2.83E-01	2.22E-02	3.62E-01
PHLDB2	5.91E+03	-8.42E-01	3.68E-01	2.22E-02	3.62E-01
ATP6V1D	4.05E+02	5.88E-01	2.57E-01	2.23E-02	3.62E-01
CHST1	3.33E+01	1.00E+00	4.39E-01	2.23E-02	3.62E-01
CMTM8	3.36E+01	9.85E-01	4.31E-01	2.23E-02	3.62E-01
CYP3A4	3.01E+00	-1.23E+00	5.37E-01	2.23E-02	3.62E-01
ACD	1.25E+02	-6.58E-01	2.89E-01	2.26E-02	3.63E-01
AKAP5	1.20E+01	1.05E+00	4.61E-01	2.28E-02	3.63E-01
BAG1	5.94E+02	-8.47E-01	3.72E-01	2.26E-02	3.63E-01
C3orf18	1.70E+02	-9.21E-01	4.05E-01	2.28E-02	3.63E-01
CCND1	1.94E+04	8.47E-01	3.71E-01	2.24E-02	3.63E-01
CD209	4.27E+01	1.24E+00	5.45E-01	2.28E-02	3.63E-01
CLEC10A	8.05E+00	-1.24E+00	5.45E-01	2.26E-02	3.63E-01
CRMP1	1.45E+02	-1.11E+00	4.86E-01	2.28E-02	3.63E-01
FAM118A	2.73E+02	-1.09E+00	4.78E-01	2.25E-02	3.63E-01
HSPA12A	1.50E+02	1.08E+00	4.75E-01	2.26E-02	3.63E-01
IPO5P1	2.79E+02	5.34E-01	2.35E-01	2.28E-02	3.63E-01
IRF8	1.33E+02	-9.95E-01	4.37E-01	2.29E-02	3.63E-01
JAG1	4.37E+03	9.34E-01	4.09E-01	2.24E-02	3.63E-01
LOC1019297	9.34E+00	-1.08E+00	4.73E-01	2.28E-02	3.63E-01
MTMR12	9.40E+02	4.21E-01	1.84E-01	2.26E-02	3.63E-01
NOG	4.15E+00	1.19E+00	5.22E-01	2.26E-02	3.63E-01
NRG1	6.45E+01	-1.24E+00	5.44E-01	2.26E-02	3.63E-01
PIGH	1.44E+02	6.70E-01	2.94E-01	2.27E-02	3.63E-01
PREB	3.51E+02	-5.71E-01	2.51E-01	2.29E-02	3.63E-01
RMI2	2.54E+01	8.52E-01	3.74E-01	2.28E-02	3.63E-01
SCRN2	2.28E+02	-5.40E-01	2.38E-01	2.29E-02	3.63E-01
USH2A	3.22E+01	-1.24E+00	5.45E-01	2.26E-02	3.63E-01

ZNF287	1.42E+02	4.77E-01	2.10E-01	2.28E-02	3.63E-01
ZNF493	9.98E+02	7.78E-01	3.41E-01	2.27E-02	3.63E-01
ZNF629	7.58E+02	-3.70E-01	1.62E-01	2.24E-02	3.63E-01
LOC1005061	7.91E+00	-1.23E+00	5.39E-01	2.30E-02	3.64E-01
EXOSC6	2.25E+02	-4.95E-01	2.18E-01	2.30E-02	3.64E-01
LOC1019295	9.07E+00	1.24E+00	5.45E-01	2.31E-02	3.65E-01
FAT4	1.34E+04	-1.12E+00	4.92E-01	2.31E-02	3.65E-01
SLC6A1	1.05E+03	1.22E+00	5.38E-01	2.31E-02	3.65E-01
C1orf204	2.07E+01	-8.99E-01	3.96E-01	2.33E-02	3.65E-01
CDCA2	7.69E+01	1.14E+00	5.03E-01	2.32E-02	3.65E-01
MAP10	6.82E+01	1.12E+00	4.95E-01	2.33E-02	3.65E-01
OVCH1	1.63E+01	1.16E+00	5.11E-01	2.33E-02	3.65E-01
PLAUR	6.87E+01	-8.07E-01	3.56E-01	2.33E-02	3.65E-01
TMEM53	1.88E+01	9.65E-01	4.25E-01	2.33E-02	3.65E-01
ZBTB1	6.22E+02	6.22E-01	2.74E-01	2.34E-02	3.66E-01
RASL11A	2.14E+01	9.42E-01	4.16E-01	2.34E-02	3.66E-01
HSPH1	1.17E+03	-7.06E-01	3.11E-01	2.35E-02	3.66E-01
HSPA5	7.00E+03	-7.25E-01	3.20E-01	2.35E-02	3.67E-01
ZIC4	2.33E+02	-9.94E-01	4.39E-01	2.35E-02	3.67E-01
ZNF843	6.83E+00	-1.10E+00	4.85E-01	2.36E-02	3.67E-01
C5AR1	2.09E+02	-9.33E-01	4.12E-01	2.36E-02	3.68E-01
KYNU	2.85E+01	-9.08E-01	4.01E-01	2.36E-02	3.68E-01
GLIDR	6.24E+01	-9.42E-01	4.16E-01	2.37E-02	3.68E-01
MSRA	7.79E+01	-6.64E-01	2.94E-01	2.37E-02	3.68E-01
SMCO4	2.24E+01	-9.90E-01	4.38E-01	2.37E-02	3.68E-01
FAM35DP	1.51E+01	9.45E-01	4.18E-01	2.38E-02	3.68E-01
HSD17B2	1.57E+02	9.85E-01	4.36E-01	2.38E-02	3.68E-01
HECTD1	2.99E+03	6.35E-01	2.81E-01	2.39E-02	3.69E-01
TASP1	1.50E+02	5.39E-01	2.38E-01	2.39E-02	3.69E-01
SOS2	1.16E+03	7.53E-01	3.33E-01	2.39E-02	3.69E-01
MAPK1IP1L	1.49E+03	6.37E-01	2.82E-01	2.41E-02	3.70E-01
POFUT1	1.31E+03	5.36E-01	2.38E-01	2.40E-02	3.70E-01
SERTAD2	2.39E+02	-7.01E-01	3.11E-01	2.40E-02	3.70E-01
UBR7	4.17E+02	5.25E-01	2.33E-01	2.41E-02	3.70E-01
LIMK1	3.49E+02	-5.63E-01	2.49E-01	2.41E-02	3.71E-01
BLNK	1.12E+02	-9.21E-01	4.09E-01	2.42E-02	3.72E-01
CXCL1	3.80E+00	1.17E+00	5.21E-01	2.43E-02	3.72E-01
HRSP12	5.05E+01	-6.69E-01	2.97E-01	2.43E-02	3.72E-01
NT5DC1	3.03E+02	-5.92E-01	2.63E-01	2.43E-02	3.72E-01
AKR7A3	9.48E+00	9.95E-01	4.42E-01	2.44E-02	3.72E-01
SLC10A4	5.69E+00	1.23E+00	5.45E-01	2.44E-02	3.72E-01
ARID4A	5.12E+02	6.52E-01	2.90E-01	2.45E-02	3.73E-01
TRAPPC6B	3.66E+02	7.72E-01	3.43E-01	2.45E-02	3.73E-01
RECK	1.25E+03	-7.18E-01	3.19E-01	2.45E-02	3.73E-01
GREB1	7.11E+02	1.19E+00	5.30E-01	2.47E-02	3.75E-01
HEPACAM2	1.08E+01	-1.17E+00	5.20E-01	2.47E-02	3.75E-01
PPP1R3E	1.30E+02	7.16E-01	3.19E-01	2.48E-02	3.76E-01
RNFT1	1.55E+02	-6.15E-01	2.74E-01	2.49E-02	3.77E-01
ATP6V0E2-A	8.15E+01	-9.66E-01	4.31E-01	2.49E-02	3.78E-01
CDCA4	1.01E+02	7.78E-01	3.48E-01	2.51E-02	3.79E-01
NDUFB9	3.45E+02	-6.18E-01	2.76E-01	2.51E-02	3.79E-01
SV2B	1.90E+01	-1.20E+00	5.38E-01	2.51E-02	3.79E-01
TTC3	4.98E+03	4.44E-01	1.98E-01	2.51E-02	3.79E-01

CTSS	5.99E+02	-8.43E-01	3.76E-01	2.52E-02	3.80E-01
SALL1	1.94E+02	-1.22E+00	5.44E-01	2.52E-02	3.80E-01
KIAA0391	4.69E+02	6.13E-01	2.74E-01	2.53E-02	3.81E-01
ASXL1	1.57E+03	4.97E-01	2.23E-01	2.56E-02	3.81E-01
ATR	8.63E+02	-5.33E-01	2.39E-01	2.57E-02	3.81E-01
BCAS4	8.55E+01	1.13E+00	5.05E-01	2.55E-02	3.81E-01
C19orf26	1.62E+01	-1.21E+00	5.42E-01	2.55E-02	3.81E-01
CASC15	4.94E+01	-1.21E+00	5.43E-01	2.58E-02	3.81E-01
CNNM3	3.51E+02	-4.59E-01	2.06E-01	2.58E-02	3.81E-01
DHX29	6.57E+02	3.60E-01	1.61E-01	2.55E-02	3.81E-01
DOHH	5.24E+01	-7.08E-01	3.18E-01	2.58E-02	3.81E-01
ERAP1	1.13E+03	-6.10E-01	2.74E-01	2.58E-02	3.81E-01
FAT3	6.84E+02	-1.15E+00	5.18E-01	2.59E-02	3.81E-01
HLA-C	1.33E+03	-6.18E-01	2.76E-01	2.55E-02	3.81E-01
IL1RAP	3.35E+02	-8.05E-01	3.61E-01	2.57E-02	3.81E-01
LNX2	5.82E+02	6.63E-01	2.97E-01	2.54E-02	3.81E-01
MCAM	2.08E+03	1.04E+00	4.64E-01	2.58E-02	3.81E-01
MTA1	6.92E+02	7.21E-01	3.23E-01	2.57E-02	3.81E-01
PNMA6A	8.09E+00	1.11E+00	4.97E-01	2.54E-02	3.81E-01
POLN	4.02E+01	6.54E-01	2.93E-01	2.55E-02	3.81E-01
SLC2A4RG	5.28E+02	6.95E-01	3.11E-01	2.56E-02	3.81E-01
STYX	2.85E+02	5.53E-01	2.47E-01	2.54E-02	3.81E-01
TSPYL1	2.07E+03	-5.69E-01	2.55E-01	2.57E-02	3.81E-01
ZNF180	1.26E+02	6.07E-01	2.72E-01	2.58E-02	3.81E-01
ZNFX1	1.75E+03	5.90E-01	2.64E-01	2.57E-02	3.81E-01
ZFYVE21	3.21E+02	8.30E-01	3.73E-01	2.59E-02	3.81E-01
FAM184A	4.85E+01	-1.10E+00	4.96E-01	2.60E-02	3.82E-01
ALS2CL	2.84E+02	-9.98E-01	4.49E-01	2.63E-02	3.84E-01
AP5S1	2.44E+02	7.32E-01	3.29E-01	2.62E-02	3.84E-01
C14orf166	6.04E+02	6.18E-01	2.78E-01	2.62E-02	3.84E-01
CERS6	7.96E+02	3.81E-01	1.71E-01	2.62E-02	3.84E-01
CHD8	1.70E+03	6.34E-01	2.85E-01	2.63E-02	3.84E-01
DCDC5	2.21E+01	-1.11E+00	5.00E-01	2.64E-02	3.84E-01
DCUN1D2	2.65E+02	6.63E-01	2.99E-01	2.64E-02	3.84E-01
DOK4	3.12E+02	-5.28E-01	2.38E-01	2.63E-02	3.84E-01
LBX2	9.40E+00	-1.21E+00	5.45E-01	2.64E-02	3.84E-01
MAX	5.45E+02	5.68E-01	2.56E-01	2.64E-02	3.84E-01
NPY6R	1.14E+01	-1.19E+00	5.36E-01	2.62E-02	3.84E-01
THAP11	1.77E+02	-6.03E-01	2.71E-01	2.63E-02	3.84E-01
TMEM102	1.87E+01	-1.13E+00	5.11E-01	2.64E-02	3.84E-01
TULP1	5.89E+00	-1.21E+00	5.44E-01	2.63E-02	3.84E-01
MGAT2	4.19E+02	7.13E-01	3.21E-01	2.65E-02	3.84E-01
YJEFN3	2.55E+01	-9.90E-01	4.46E-01	2.64E-02	3.84E-01
WNT2B	8.97E+01	1.19E+00	5.37E-01	2.66E-02	3.85E-01
SMOX	3.17E+02	1.06E+00	4.78E-01	2.67E-02	3.86E-01
CHCHD4	1.17E+02	-7.07E-01	3.19E-01	2.68E-02	3.86E-01
CORO1C	2.23E+03	5.46E-01	2.46E-01	2.68E-02	3.86E-01
HES4	1.83E+02	9.53E-01	4.30E-01	2.67E-02	3.86E-01
IFT172	5.71E+02	-6.56E-01	2.96E-01	2.67E-02	3.86E-01
PEX5L	7.48E+01	-1.21E+00	5.45E-01	2.68E-02	3.86E-01
PPP2R5E	4.78E+02	6.04E-01	2.73E-01	2.68E-02	3.86E-01
TNRC18P1	2.17E+01	1.02E+00	4.59E-01	2.68E-02	3.86E-01
MND1	1.10E+01	1.15E+00	5.21E-01	2.69E-02	3.87E-01

ATP2C1	1.27E+03	-4.71E-01	2.13E-01	2.70E-02	3.87E-01
TRMT12	1.26E+02	-6.81E-01	3.08E-01	2.70E-02	3.87E-01
PIGG	9.59E+02	5.78E-01	2.61E-01	2.70E-02	3.87E-01
GNGT2	1.56E+01	-1.07E+00	4.85E-01	2.71E-02	3.89E-01
NAA30	2.79E+02	5.94E-01	2.69E-01	2.72E-02	3.89E-01
ZNF93	7.70E+01	8.44E-01	3.82E-01	2.72E-02	3.89E-01
SYT2	5.44E+00	1.20E+00	5.44E-01	2.72E-02	3.89E-01
FAM78B	2.42E+01	-1.19E+00	5.41E-01	2.73E-02	3.89E-01
MYLK4	2.61E+02	-1.20E+00	5.43E-01	2.74E-02	3.90E-01
SHANK3	4.89E+02	1.10E+00	5.01E-01	2.74E-02	3.90E-01
ABCA7	3.68E+02	-6.54E-01	2.96E-01	2.75E-02	3.91E-01
LILRA5	4.01E+00	1.20E+00	5.45E-01	2.75E-02	3.91E-01
LINC01057	1.61E+01	-1.05E+00	4.77E-01	2.76E-02	3.91E-01
RHOT2	6.27E+02	-4.75E-01	2.15E-01	2.75E-02	3.91E-01
DCAF5	1.17E+03	6.96E-01	3.16E-01	2.76E-02	3.91E-01
PCMTD2	9.47E+02	4.26E-01	1.93E-01	2.76E-02	3.91E-01
ACSM5	2.16E+01	-1.13E+00	5.13E-01	2.77E-02	3.91E-01
FRA10AC1	1.27E+02	7.63E-01	3.46E-01	2.77E-02	3.91E-01
PEX1	3.49E+02	-3.96E-01	1.80E-01	2.77E-02	3.91E-01
CDADC1	9.32E+01	5.34E-01	2.43E-01	2.78E-02	3.92E-01
DEPDC1B	2.14E+01	1.18E+00	5.38E-01	2.78E-02	3.92E-01
IKZF4	9.65E+01	8.25E-01	3.75E-01	2.79E-02	3.92E-01
PRKRA	2.75E+02	-3.73E-01	1.69E-01	2.78E-02	3.92E-01
SORCS1	8.54E+01	-1.19E+00	5.42E-01	2.79E-02	3.92E-01
SUGT1	3.43E+02	4.10E-01	1.87E-01	2.78E-02	3.92E-01
THBD	2.55E+02	1.16E+00	5.30E-01	2.79E-02	3.92E-01
MAMLD1	7.70E+01	1.05E+00	4.76E-01	2.79E-02	3.92E-01
PHLDB3	5.92E+01	-8.26E-01	3.76E-01	2.79E-02	3.92E-01
NID1	2.06E+03	9.87E-01	4.49E-01	2.80E-02	3.92E-01
EPHA7	3.02E+03	-1.12E+00	5.09E-01	2.80E-02	3.92E-01
REL	5.39E+02	-6.43E-01	2.93E-01	2.80E-02	3.92E-01
RGCC	1.68E+02	1.10E+00	5.00E-01	2.81E-02	3.92E-01
ADNP	2.39E+03	4.54E-01	2.07E-01	2.81E-02	3.92E-01
CHURC1	6.87E+02	6.96E-01	3.17E-01	2.81E-02	3.92E-01
HACL1	1.96E+02	-5.34E-01	2.43E-01	2.82E-02	3.92E-01
TCERG1	1.05E+03	4.32E-01	1.97E-01	2.82E-02	3.92E-01
PROSER2	2.34E+02	8.80E-01	4.01E-01	2.83E-02	3.94E-01
BCL2L2	5.07E+02	7.49E-01	3.42E-01	2.84E-02	3.94E-01
MEOX2	1.78E+03	-1.05E+00	4.78E-01	2.84E-02	3.94E-01
NPW	5.65E+01	-1.19E+00	5.41E-01	2.85E-02	3.94E-01
RANBP10	4.33E+02	-5.80E-01	2.65E-01	2.84E-02	3.94E-01
STX16	8.85E+02	5.61E-01	2.56E-01	2.85E-02	3.94E-01
TAT	2.94E+00	9.91E-01	4.52E-01	2.85E-02	3.94E-01
USP16	7.24E+02	4.47E-01	2.04E-01	2.85E-02	3.94E-01
AACS	4.40E+02	5.42E-01	2.48E-01	2.91E-02	3.95E-01
ATP2B3	1.05E+01	1.19E+00	5.45E-01	2.91E-02	3.95E-01
COL4A2	2.16E+03	1.06E+00	4.85E-01	2.90E-02	3.95E-01
CPEB2	7.39E+02	9.84E-01	4.50E-01	2.87E-02	3.95E-01
EDN1	2.22E+01	1.02E+00	4.67E-01	2.91E-02	3.95E-01
ERMP1	2.20E+03	-8.16E-01	3.73E-01	2.88E-02	3.95E-01
FAM195A	8.76E+01	-9.58E-01	4.39E-01	2.89E-02	3.95E-01
FGL2	1.07E+04	-1.12E+00	5.12E-01	2.88E-02	3.95E-01
GPR137C	1.09E+02	8.63E-01	3.94E-01	2.87E-02	3.95E-01

IL21R	7.64E+00	-1.16E+00	5.29E-01	2.88E-02	3.95E-01
LOC1019285	7.61E+00	1.15E+00	5.25E-01	2.89E-02	3.95E-01
MIPOL1	9.29E+01	9.39E-01	4.30E-01	2.90E-02	3.95E-01
MOCS2	4.52E+02	5.46E-01	2.49E-01	2.86E-02	3.95E-01
PAWR	5.57E+02	8.41E-01	3.85E-01	2.88E-02	3.95E-01
PGM5	2.26E+02	-1.11E+00	5.07E-01	2.91E-02	3.95E-01
POLR2A	1.90E+03	5.94E-01	2.72E-01	2.87E-02	3.95E-01
SH3BP1	9.88E+01	-9.42E-01	4.31E-01	2.89E-02	3.95E-01
SOGA1	1.41E+03	8.04E-01	3.67E-01	2.87E-02	3.95E-01
TSPAN9	5.80E+02	9.84E-01	4.50E-01	2.89E-02	3.95E-01
USO1	1.25E+03	4.00E-01	1.83E-01	2.91E-02	3.95E-01
WRN	5.03E+02	-7.05E-01	3.23E-01	2.90E-02	3.95E-01
YWHAB	2.71E+03	5.17E-01	2.37E-01	2.88E-02	3.95E-01
ZNF708	6.87E+02	5.84E-01	2.67E-01	2.89E-02	3.95E-01
CDC14B	4.13E+02	-5.64E-01	2.59E-01	2.92E-02	3.95E-01
DDRGK1	4.33E+02	5.76E-01	2.64E-01	2.92E-02	3.95E-01
MARK3	7.19E+02	6.72E-01	3.08E-01	2.93E-02	3.95E-01
TMEM161B-/	2.26E+01	-8.29E-01	3.80E-01	2.93E-02	3.95E-01
TP63	1.11E+03	-1.19E+00	5.44E-01	2.93E-02	3.96E-01
EOGT	2.68E+02	8.22E-01	3.77E-01	2.94E-02	3.97E-01
ZNF568	2.51E+02	6.27E-01	2.88E-01	2.94E-02	3.97E-01
AIFM1	3.31E+02	-5.07E-01	2.33E-01	2.98E-02	3.97E-01
AKAP1	8.41E+02	-6.00E-01	2.76E-01	2.99E-02	3.97E-01
ARPP19	2.34E+03	5.01E-01	2.30E-01	2.97E-02	3.97E-01
BRWD1	2.65E+03	3.90E-01	1.79E-01	2.95E-02	3.97E-01
C19orf68	4.38E+01	6.57E-01	3.03E-01	3.00E-02	3.97E-01
CCDC153	8.23E+00	1.13E+00	5.19E-01	2.97E-02	3.97E-01
DGCR6	2.56E+01	-1.03E+00	4.72E-01	2.99E-02	3.97E-01
EGFL6	9.78E+02	-1.13E+00	5.21E-01	3.00E-02	3.97E-01
FAM167A	4.55E+02	-1.09E+00	5.01E-01	2.96E-02	3.97E-01
FPR2	3.33E+00	1.18E+00	5.45E-01	2.99E-02	3.97E-01
GATA3	5.34E+01	-1.17E+00	5.37E-01	2.97E-02	3.97E-01
GLDN	1.09E+02	-1.18E+00	5.44E-01	2.99E-02	3.97E-01
LINC01278	1.44E+02	5.72E-01	2.63E-01	2.99E-02	3.97E-01
LOC1026064	1.42E+01	-1.04E+00	4.76E-01	2.97E-02	3.97E-01
MB21D1	2.38E+01	-9.05E-01	4.17E-01	3.00E-02	3.97E-01
MED14	1.11E+03	4.86E-01	2.24E-01	2.99E-02	3.97E-01
NBPF3	7.43E+01	-8.71E-01	4.02E-01	3.00E-02	3.97E-01
PDE4B	1.30E+02	-8.25E-01	3.79E-01	2.97E-02	3.97E-01
PSMC1	3.50E+02	4.40E-01	2.03E-01	2.98E-02	3.97E-01
PVRL3	7.41E+02	-6.64E-01	3.06E-01	3.00E-02	3.97E-01
RALGAPB	1.90E+03	5.90E-01	2.72E-01	3.00E-02	3.97E-01
RCOR1	4.26E+02	7.57E-01	3.49E-01	3.00E-02	3.97E-01
VSIG4	6.07E+02	-9.49E-01	4.38E-01	3.00E-02	3.97E-01
ZC3H13	1.42E+03	5.35E-01	2.46E-01	3.01E-02	3.97E-01
PPAP2C	3.10E+01	-1.04E+00	4.80E-01	3.01E-02	3.97E-01
MS4A14	6.01E+01	-1.01E+00	4.67E-01	3.01E-02	3.97E-01
PXYLP1	1.03E+02	7.20E-01	3.32E-01	3.02E-02	3.97E-01
UBE2D2	6.26E+02	3.71E-01	1.71E-01	3.02E-02	3.97E-01
YPEL4	4.58E+00	1.18E+00	5.45E-01	3.02E-02	3.97E-01
KAT2B	1.20E+03	-6.05E-01	2.79E-01	3.03E-02	3.98E-01
ULK2	4.90E+02	5.15E-01	2.38E-01	3.03E-02	3.98E-01
LEPR	8.17E+03	-1.10E+00	5.06E-01	3.05E-02	4.00E-01

VAMP1	2.14E+02	5.96E-01	2.76E-01	3.05E-02	4.00E-01
DPM1	2.23E+02	5.00E-01	2.31E-01	3.06E-02	4.00E-01
HYMAI	6.64E+01	1.05E+00	4.85E-01	3.06E-02	4.00E-01
CTBP2	1.39E+03	5.41E-01	2.50E-01	3.08E-02	4.03E-01
BLCAP	1.65E+03	7.48E-01	3.47E-01	3.08E-02	4.03E-01
BMS1P4	9.69E+01	4.42E-01	2.05E-01	3.09E-02	4.04E-01
PPM1A	9.70E+02	6.42E-01	2.98E-01	3.10E-02	4.04E-01
C4orf36	4.88E+00	1.04E+00	4.86E-01	3.14E-02	4.04E-01
CINP	1.39E+02	6.46E-01	3.00E-01	3.14E-02	4.04E-01
DIP2B	3.45E+03	-5.77E-01	2.68E-01	3.12E-02	4.04E-01
DNM3	2.38E+02	9.31E-01	4.32E-01	3.13E-02	4.04E-01
FBLN7	2.61E+01	-1.13E+00	5.23E-01	3.11E-02	4.04E-01
KIAA0247	2.12E+03	7.26E-01	3.37E-01	3.13E-02	4.04E-01
LAIR1	3.47E+02	-8.17E-01	3.80E-01	3.14E-02	4.04E-01
LAT2	1.46E+02	-8.97E-01	4.17E-01	3.13E-02	4.04E-01
LOC1019268	3.95E+01	1.12E+00	5.22E-01	3.14E-02	4.04E-01
MCM8	1.08E+02	6.44E-01	2.99E-01	3.13E-02	4.04E-01
NEFH	3.15E+01	1.17E+00	5.44E-01	3.14E-02	4.04E-01
PNMT	7.65E+00	1.12E+00	5.18E-01	3.12E-02	4.04E-01
POLR1B	5.46E+02	-6.13E-01	2.85E-01	3.14E-02	4.04E-01
SLC9A3R1	8.64E+02	8.50E-01	3.95E-01	3.13E-02	4.04E-01
SLIRP	1.30E+02	5.97E-01	2.77E-01	3.11E-02	4.04E-01
SVIP	1.78E+02	5.17E-01	2.40E-01	3.14E-02	4.04E-01
ZMYND15	4.86E+01	-7.72E-01	3.58E-01	3.13E-02	4.04E-01
ZNF780A	3.75E+02	3.70E-01	1.72E-01	3.11E-02	4.04E-01
TAC3	6.80E+01	-1.16E+00	5.40E-01	3.15E-02	4.04E-01
L3HYPDH	9.73E+01	6.51E-01	3.03E-01	3.15E-02	4.04E-01
PXN	4.01E+03	8.59E-01	3.99E-01	3.16E-02	4.04E-01
RGS5	5.31E+03	9.85E-01	4.58E-01	3.15E-02	4.04E-01
ARFRP1	3.10E+02	5.99E-01	2.79E-01	3.18E-02	4.05E-01
CD74	9.10E+03	-9.01E-01	4.20E-01	3.18E-02	4.05E-01
EPB41L2	5.04E+03	-7.88E-01	3.67E-01	3.18E-02	4.05E-01
HEY2	6.07E+01	1.07E+00	4.98E-01	3.18E-02	4.05E-01
IFRD2	3.38E+02	-6.52E-01	3.04E-01	3.19E-02	4.05E-01
IGSF21	1.01E+02	-1.04E+00	4.84E-01	3.18E-02	4.05E-01
LOC1001323	3.45E+01	7.41E-01	3.45E-01	3.18E-02	4.05E-01
PURG	1.19E+02	-1.10E+00	5.12E-01	3.19E-02	4.05E-01
SLC16A6	6.13E+02	1.05E+00	4.91E-01	3.18E-02	4.05E-01
WWC1	2.92E+02	-9.98E-01	4.65E-01	3.17E-02	4.05E-01
COL4A3BP	1.11E+03	7.72E-01	3.60E-01	3.20E-02	4.06E-01
DET1	1.46E+02	5.04E-01	2.35E-01	3.20E-02	4.06E-01
LOC1019286	3.84E+00	1.16E+00	5.41E-01	3.20E-02	4.06E-01
PDRG1	9.71E+01	6.57E-01	3.06E-01	3.20E-02	4.06E-01
LINC00664	3.59E+01	1.14E+00	5.35E-01	3.22E-02	4.08E-01
MMP24	3.71E+01	1.09E+00	5.11E-01	3.24E-02	4.09E-01
OR51B2	6.59E+00	1.12E+00	5.25E-01	3.24E-02	4.09E-01
SYTL5	9.72E+01	-1.16E+00	5.44E-01	3.24E-02	4.09E-01
TECPR2	4.96E+02	5.94E-01	2.77E-01	3.24E-02	4.09E-01
TSPYL2	2.31E+03	7.78E-01	3.64E-01	3.25E-02	4.09E-01
C14orf28	3.97E+01	7.69E-01	3.60E-01	3.25E-02	4.09E-01
ZFPM2	4.71E+01	-1.06E+00	4.95E-01	3.25E-02	4.09E-01
PIGT	1.89E+03	5.11E-01	2.39E-01	3.26E-02	4.10E-01
FKBP1A	1.08E+03	5.78E-01	2.71E-01	3.27E-02	4.10E-01

KCNJ9	3.53E+00	-1.16E+00	5.45E-01	3.27E-02	4.10E-01
RAE1	2.08E+02	5.14E-01	2.41E-01	3.27E-02	4.10E-01
SDC4	6.34E+02	7.61E-01	3.56E-01	3.26E-02	4.10E-01
ZNF516	7.96E+02	-6.93E-01	3.25E-01	3.27E-02	4.10E-01
C3orf14	4.73E+01	-7.79E-01	3.65E-01	3.28E-02	4.10E-01
FLJ31306	6.69E+02	6.25E-01	2.93E-01	3.28E-02	4.11E-01
RAB15	5.31E+01	9.38E-01	4.40E-01	3.29E-02	4.11E-01
ATP8B1	7.61E+02	8.90E-01	4.18E-01	3.31E-02	4.12E-01
CSF1R	2.33E+03	-8.68E-01	4.07E-01	3.31E-02	4.12E-01
DPEP2	1.75E+01	-1.14E+00	5.35E-01	3.32E-02	4.12E-01
FAM129B	1.94E+03	7.36E-01	3.45E-01	3.31E-02	4.12E-01
IMPAD1	3.13E+03	-5.26E-01	2.47E-01	3.32E-02	4.12E-01
KCNE4	6.00E+02	-9.77E-01	4.58E-01	3.30E-02	4.12E-01
PAFAH2	1.01E+02	-5.46E-01	2.56E-01	3.32E-02	4.12E-01
RNA5-8S5	1.21E+03	5.02E-01	2.36E-01	3.32E-02	4.12E-01
INTS9	1.19E+02	-5.63E-01	2.64E-01	3.32E-02	4.12E-01
LBX2-AS1	4.64E+01	-1.12E+00	5.24E-01	3.33E-02	4.13E-01
PTER	1.35E+02	-8.61E-01	4.04E-01	3.33E-02	4.13E-01
BASP1	2.73E+02	9.04E-01	4.25E-01	3.34E-02	4.13E-01
IL17RC	3.27E+02	-5.72E-01	2.69E-01	3.36E-02	4.15E-01
LIPT1	3.89E+01	-6.47E-01	3.04E-01	3.35E-02	4.15E-01
NCK1-AS1	2.22E+01	-9.06E-01	4.26E-01	3.36E-02	4.15E-01
ZNF239	3.69E+01	7.57E-01	3.56E-01	3.36E-02	4.15E-01
CDS2	2.05E+03	7.29E-01	3.43E-01	3.37E-02	4.15E-01
NDUFB10	4.13E+02	-5.35E-01	2.52E-01	3.36E-02	4.15E-01
TMEM50B	1.23E+03	4.78E-01	2.25E-01	3.37E-02	4.15E-01
U2AF1	4.26E+02	3.95E-01	1.86E-01	3.37E-02	4.15E-01
HTATSF1P2	5.39E+01	-1.10E+00	5.19E-01	3.38E-02	4.15E-01
STX11	4.17E+01	-6.23E-01	2.94E-01	3.38E-02	4.16E-01
CCDC14	7.75E+02	-6.28E-01	2.96E-01	3.39E-02	4.16E-01
CD47	1.62E+03	-3.78E-01	1.78E-01	3.39E-02	4.16E-01
MIR503HG	1.97E+01	1.13E+00	5.33E-01	3.39E-02	4.16E-01
EMR1	9.70E+00	-1.09E+00	5.15E-01	3.40E-02	4.16E-01
FARP1	8.10E+02	9.36E-01	4.42E-01	3.40E-02	4.16E-01
LPGAT1	1.45E+03	8.06E-01	3.80E-01	3.40E-02	4.16E-01
ZNF512B	1.08E+03	6.50E-01	3.07E-01	3.41E-02	4.17E-01
AAK1	2.00E+03	-4.23E-01	1.99E-01	3.42E-02	4.17E-01
IQSEC3	1.23E+02	1.15E+00	5.42E-01	3.41E-02	4.17E-01
MIOS	2.94E+02	-3.37E-01	1.59E-01	3.42E-02	4.17E-01
RRN3P1	6.61E+01	-6.70E-01	3.17E-01	3.42E-02	4.17E-01
CDKN3	1.99E+01	9.94E-01	4.69E-01	3.43E-02	4.17E-01
RELL2	7.09E+01	-9.45E-01	4.47E-01	3.43E-02	4.17E-01
CNTNAP2	1.32E+01	-1.12E+00	5.30E-01	3.46E-02	4.18E-01
GFOD1	9.80E+01	9.58E-01	4.53E-01	3.45E-02	4.18E-01
MIB1	1.86E+03	7.06E-01	3.34E-01	3.46E-02	4.18E-01
MTO1	2.20E+02	-5.63E-01	2.66E-01	3.45E-02	4.18E-01
NT5C2	1.32E+03	6.55E-01	3.10E-01	3.46E-02	4.18E-01
PABPN1	2.12E+02	6.77E-01	3.20E-01	3.46E-02	4.18E-01
PDP1	8.74E+02	-5.32E-01	2.52E-01	3.45E-02	4.18E-01
PFKL	1.70E+03	7.48E-01	3.54E-01	3.46E-02	4.18E-01
ROBO3	7.71E+01	-1.03E+00	4.88E-01	3.45E-02	4.18E-01
C9orf69	2.40E+02	-5.68E-01	2.69E-01	3.47E-02	4.18E-01
NOL6	6.50E+02	-4.37E-01	2.07E-01	3.47E-02	4.18E-01

FAM117B	1.85E+02	4.38E-01	2.07E-01	3.48E-02	4.19E-01
KIF11	1.43E+02	7.69E-01	3.64E-01	3.49E-02	4.21E-01
BIN1	5.47E+02	-6.05E-01	2.87E-01	3.50E-02	4.21E-01
DICER1	1.58E+03	6.50E-01	3.08E-01	3.51E-02	4.21E-01
GPRIN2	2.01E+01	1.14E+00	5.39E-01	3.51E-02	4.21E-01
HLA-A	9.15E+02	1.01E+00	4.78E-01	3.51E-02	4.21E-01
MSC	3.53E+01	-1.13E+00	5.37E-01	3.50E-02	4.21E-01
RAD17	3.54E+02	4.04E-01	1.92E-01	3.50E-02	4.21E-01
SLC31A1	5.40E+02	-7.06E-01	3.35E-01	3.51E-02	4.21E-01
ANKRD20A5I	1.35E+02	-1.15E+00	5.44E-01	3.52E-02	4.21E-01
FAM96B	2.68E+02	-4.14E-01	1.97E-01	3.53E-02	4.22E-01
HES6	3.86E+01	8.21E-01	3.90E-01	3.53E-02	4.22E-01
KIAA1731	5.70E+02	4.77E-01	2.27E-01	3.53E-02	4.22E-01
CLDN7	8.58E+01	1.14E+00	5.40E-01	3.54E-02	4.23E-01
SSTR1	3.15E+00	9.96E-01	4.74E-01	3.55E-02	4.23E-01
TTC7B	1.97E+02	6.16E-01	2.93E-01	3.55E-02	4.23E-01
WDHD1	1.06E+02	5.70E-01	2.71E-01	3.55E-02	4.23E-01
COLCA2	6.37E+01	-1.11E+00	5.26E-01	3.55E-02	4.23E-01
ATP2B1	9.05E+02	-4.58E-01	2.18E-01	3.56E-02	4.24E-01
C16orf80	1.63E+02	-3.97E-01	1.89E-01	3.57E-02	4.24E-01
KIF17	8.99E+00	1.02E+00	4.86E-01	3.57E-02	4.24E-01
PCOLCE2	9.36E+01	-1.13E+00	5.36E-01	3.58E-02	4.24E-01
TAGLN3	6.28E+00	-1.14E+00	5.45E-01	3.59E-02	4.25E-01
CBR3	2.88E+01	1.08E+00	5.14E-01	3.61E-02	4.26E-01
FBXO31	6.08E+02	-4.65E-01	2.22E-01	3.61E-02	4.26E-01
LOC1005060	6.43E+00	1.07E+00	5.12E-01	3.61E-02	4.26E-01
MOGS	4.80E+02	-5.10E-01	2.43E-01	3.61E-02	4.26E-01
NEB	3.36E+02	-9.36E-01	4.46E-01	3.60E-02	4.26E-01
RABGEF1	4.73E+02	-4.01E-01	1.91E-01	3.61E-02	4.26E-01
TMC6	5.96E+02	7.97E-01	3.80E-01	3.60E-02	4.26E-01
XIAP	1.25E+03	2.83E-01	1.35E-01	3.61E-02	4.26E-01
YAF2	5.31E+02	5.53E-01	2.64E-01	3.62E-02	4.26E-01
ARRDC5	9.46E+00	-1.13E+00	5.40E-01	3.63E-02	4.26E-01
COX10-AS1	6.83E+01	-6.74E-01	3.22E-01	3.62E-02	4.26E-01
NAA50	1.24E+03	-3.92E-01	1.87E-01	3.63E-02	4.26E-01
RUFY2	4.59E+02	5.26E-01	2.51E-01	3.63E-02	4.27E-01
NPAS2	1.33E+02	7.18E-01	3.43E-01	3.64E-02	4.27E-01
EDN3	2.00E+02	1.12E+00	5.34E-01	3.65E-02	4.27E-01
LRRTM3	3.33E+00	1.14E+00	5.44E-01	3.65E-02	4.27E-01
N4BP2L2	2.01E+03	4.14E-01	1.98E-01	3.66E-02	4.27E-01
SLC38A6	2.76E+02	7.19E-01	3.44E-01	3.66E-02	4.27E-01
SNAPC2	9.21E+01	-5.68E-01	2.72E-01	3.65E-02	4.27E-01
TMEM57	4.68E+02	6.54E-01	3.13E-01	3.66E-02	4.27E-01
WDSUB1	1.50E+02	-5.13E-01	2.45E-01	3.64E-02	4.27E-01
GABARAPL2	6.32E+02	-5.48E-01	2.62E-01	3.67E-02	4.28E-01
NXNL2	1.77E+01	-8.85E-01	4.24E-01	3.67E-02	4.28E-01
ANKRD32	1.43E+02	8.28E-01	3.97E-01	3.68E-02	4.29E-01
ABR	1.42E+03	6.19E-01	2.97E-01	3.70E-02	4.30E-01
ALDH16A1	2.28E+02	-4.57E-01	2.19E-01	3.71E-02	4.30E-01
ANP32A-IT1	5.65E+01	7.11E-01	3.41E-01	3.70E-02	4.30E-01
C12orf57	5.03E+02	-5.30E-01	2.54E-01	3.71E-02	4.30E-01
CHORDC1	2.79E+02	-4.65E-01	2.23E-01	3.70E-02	4.30E-01
DHRS2	7.24E+01	1.12E+00	5.37E-01	3.70E-02	4.30E-01

TPD52L2	1.54E+03	6.69E-01	3.21E-01	3.71E-02	4.30E-01
SOAT2	3.29E+00	1.13E+00	5.42E-01	3.72E-02	4.30E-01
DISP1	2.24E+03	-8.52E-01	4.09E-01	3.72E-02	4.30E-01
CPE	7.91E+03	1.13E+00	5.44E-01	3.74E-02	4.31E-01
HLA-DRB3	5.46E+01	-1.12E+00	5.37E-01	3.73E-02	4.31E-01
KIFC2	4.82E+02	6.28E-01	3.01E-01	3.73E-02	4.31E-01
NAGA	4.25E+02	-5.21E-01	2.50E-01	3.73E-02	4.31E-01
SATB2	8.87E+01	8.22E-01	3.95E-01	3.73E-02	4.31E-01
TYROBP	3.53E+02	-8.16E-01	3.92E-01	3.74E-02	4.31E-01
EVI2B	1.72E+02	-9.51E-01	4.57E-01	3.74E-02	4.31E-01
PLA2G4A	8.98E+01	-8.31E-01	4.00E-01	3.74E-02	4.31E-01
RIPK4	2.98E+02	-1.08E+00	5.19E-01	3.75E-02	4.31E-01
CDIPT	5.28E+02	-3.98E-01	1.91E-01	3.75E-02	4.31E-01
MRPL52	1.24E+02	6.81E-01	3.27E-01	3.75E-02	4.31E-01
CCDC125	2.77E+02	4.77E-01	2.29E-01	3.76E-02	4.31E-01
FAM210B	9.22E+02	4.03E-01	1.94E-01	3.76E-02	4.31E-01
FXYD7	1.18E+01	1.12E+00	5.41E-01	3.78E-02	4.31E-01
LOC1005064	1.60E+01	1.04E+00	4.99E-01	3.77E-02	4.31E-01
LOC642236	9.80E+00	1.13E+00	5.43E-01	3.77E-02	4.31E-01
LOC730102	8.57E+01	-7.21E-01	3.47E-01	3.76E-02	4.31E-01
RUSC1	4.78E+02	5.89E-01	2.83E-01	3.77E-02	4.31E-01
ZBTB37	3.32E+02	4.06E-01	1.96E-01	3.77E-02	4.31E-01
DSCAM	5.59E+00	-1.13E+00	5.45E-01	3.79E-02	4.31E-01
HSPA4L	4.14E+02	-8.15E-01	3.93E-01	3.78E-02	4.31E-01
SLC24A3	9.96E+02	1.08E+00	5.20E-01	3.78E-02	4.31E-01
SMS	2.04E+02	5.56E-01	2.68E-01	3.78E-02	4.31E-01
EYA1	1.56E+03	-1.11E+00	5.36E-01	3.79E-02	4.31E-01
TTC13	1.79E+02	4.45E-01	2.14E-01	3.79E-02	4.31E-01
MIS18A	8.44E+01	5.59E-01	2.69E-01	3.80E-02	4.32E-01
TVP23B	2.99E+02	-4.05E-01	1.95E-01	3.80E-02	4.32E-01
CAPZA2	1.20E+03	-3.20E-01	1.54E-01	3.81E-02	4.32E-01
VPS28	5.26E+02	-4.42E-01	2.13E-01	3.82E-02	4.32E-01
CACNA1H	2.61E+02	9.78E-01	4.72E-01	3.83E-02	4.33E-01
CHMP4B	1.05E+03	5.80E-01	2.80E-01	3.83E-02	4.33E-01
EIF2S1	5.52E+02	4.68E-01	2.26E-01	3.83E-02	4.33E-01
FAM83E	8.79E+00	1.13E+00	5.44E-01	3.82E-02	4.33E-01
PVRL4	4.01E+00	1.06E+00	5.10E-01	3.83E-02	4.33E-01
TSEN15	2.06E+02	6.04E-01	2.92E-01	3.83E-02	4.33E-01
ARFGEF2	1.54E+03	4.66E-01	2.25E-01	3.84E-02	4.33E-01
CYP2S1	1.78E+01	-9.31E-01	4.50E-01	3.84E-02	4.33E-01
IL17RE	3.09E+01	-1.08E+00	5.22E-01	3.84E-02	4.33E-01
CDC25B	1.90E+03	6.90E-01	3.33E-01	3.85E-02	4.34E-01
CECR6	4.26E+01	-1.02E+00	4.92E-01	3.86E-02	4.34E-01
ENAH	5.72E+03	7.17E-01	3.47E-01	3.86E-02	4.34E-01
ANGPTL1	5.20E+01	-1.09E+00	5.27E-01	3.88E-02	4.36E-01
AFF1	4.21E+03	6.35E-01	3.07E-01	3.89E-02	4.36E-01
DISP2	3.51E+02	8.68E-01	4.20E-01	3.89E-02	4.36E-01
KLHL28	3.07E+02	6.05E-01	2.93E-01	3.89E-02	4.36E-01
TENC1	3.26E+03	4.91E-01	2.38E-01	3.89E-02	4.36E-01
TTC9	1.91E+02	7.67E-01	3.71E-01	3.89E-02	4.36E-01
LRRC73	7.92E+00	-1.01E+00	4.89E-01	3.90E-02	4.36E-01
ATP11A	9.43E+02	5.32E-01	2.58E-01	3.91E-02	4.37E-01
ARL4C	1.39E+02	1.04E+00	5.04E-01	3.92E-02	4.37E-01

C14orf2	3.14E+02	5.72E-01	2.78E-01	3.94E-02	4.37E-01
CD1D	1.01E+01	1.00E+00	4.85E-01	3.92E-02	4.37E-01
DDX24	1.44E+03	6.06E-01	2.94E-01	3.94E-02	4.37E-01
HLA-F	1.46E+01	1.11E+00	5.40E-01	3.93E-02	4.37E-01
HSPB6	2.31E+01	1.06E+00	5.17E-01	3.93E-02	4.37E-01
MAVS	3.75E+03	6.27E-01	3.04E-01	3.92E-02	4.37E-01
NEURL1B	1.51E+02	9.37E-01	4.55E-01	3.93E-02	4.37E-01
TDRD6	4.98E+01	-9.70E-01	4.71E-01	3.94E-02	4.37E-01
ZSWIM3	7.57E+01	5.54E-01	2.69E-01	3.93E-02	4.37E-01
CORO2B	3.19E+02	1.08E+00	5.26E-01	3.96E-02	4.38E-01
EDC4	7.27E+02	-3.52E-01	1.71E-01	3.96E-02	4.38E-01
MORC3	7.09E+02	4.51E-01	2.19E-01	3.96E-02	4.38E-01
OR52H1	3.05E+01	7.95E-01	3.86E-01	3.95E-02	4.38E-01
RAB11FIP1	1.09E+03	7.16E-01	3.48E-01	3.96E-02	4.38E-01
SLC44A1	1.63E+03	-7.89E-01	3.83E-01	3.96E-02	4.38E-01
TMEM161B	2.17E+02	3.93E-01	1.91E-01	3.96E-02	4.38E-01
SPATA6L	2.78E+01	-9.82E-01	4.77E-01	3.97E-02	4.38E-01
TSIX	8.76E+00	1.11E+00	5.39E-01	3.97E-02	4.38E-01
ADAM21	4.53E+00	1.09E+00	5.31E-01	3.99E-02	4.38E-01
C21orf58	6.47E+01	5.96E-01	2.90E-01	3.98E-02	4.38E-01
DKK2	2.83E+03	-1.06E+00	5.16E-01	3.99E-02	4.38E-01
FLNA	1.39E+04	6.08E-01	2.96E-01	3.99E-02	4.38E-01
PPP4R4	1.41E+01	1.08E+00	5.25E-01	3.99E-02	4.38E-01
TMUB2	3.94E+02	-3.39E-01	1.65E-01	3.99E-02	4.38E-01
TPM1	2.45E+03	7.12E-01	3.47E-01	3.99E-02	4.38E-01
AKT2	1.75E+03	3.70E-01	1.81E-01	4.05E-02	4.40E-01
ANO6	4.96E+03	7.79E-01	3.80E-01	4.06E-02	4.40E-01
ASPHD1	3.38E+00	-1.11E+00	5.42E-01	4.04E-02	4.40E-01
ATXN3	3.80E+02	6.28E-01	3.06E-01	4.03E-02	4.40E-01
ELMSAN1	9.72E+02	7.13E-01	3.48E-01	4.06E-02	4.40E-01
FAM179B	5.38E+02	5.57E-01	2.72E-01	4.06E-02	4.40E-01
GPR161	5.33E+02	6.84E-01	3.34E-01	4.06E-02	4.40E-01
LOC1025462	1.78E+01	-8.77E-01	4.28E-01	4.05E-02	4.40E-01
MINOS1P1	1.27E+02	5.74E-01	2.80E-01	4.06E-02	4.40E-01
MMRN1	1.92E+02	1.11E+00	5.43E-01	4.06E-02	4.40E-01
NMNAT3	2.49E+01	-1.11E+00	5.41E-01	4.04E-02	4.40E-01
PBX2	8.22E+00	1.08E+00	5.25E-01	4.05E-02	4.40E-01
PDIA4	1.68E+03	-5.22E-01	2.55E-01	4.06E-02	4.40E-01
S100A9	7.05E+01	1.08E+00	5.28E-01	4.06E-02	4.40E-01
TBC1D24	3.89E+02	7.02E-01	3.42E-01	4.03E-02	4.40E-01
TGM4	7.30E+00	1.10E+00	5.38E-01	4.06E-02	4.40E-01
TMEM159	1.90E+02	-6.87E-01	3.35E-01	4.03E-02	4.40E-01
TXNIP	1.74E+04	-8.62E-01	4.21E-01	4.06E-02	4.40E-01
VAPB	1.36E+03	4.97E-01	2.43E-01	4.04E-02	4.40E-01
ZNF335	4.51E+02	5.43E-01	2.65E-01	4.03E-02	4.40E-01
PRND	4.43E+00	1.11E+00	5.44E-01	4.07E-02	4.40E-01
FAM109B	1.25E+02	-7.05E-01	3.45E-01	4.08E-02	4.41E-01
ACTR10	3.80E+02	6.21E-01	3.04E-01	4.09E-02	4.41E-01
CCDC93	8.88E+02	-4.74E-01	2.32E-01	4.09E-02	4.41E-01
CERS5	5.47E+02	3.76E-01	1.84E-01	4.08E-02	4.41E-01
NSUN6	1.37E+02	4.02E-01	1.97E-01	4.09E-02	4.41E-01
CNKSR3	3.34E+02	9.53E-01	4.66E-01	4.10E-02	4.41E-01
DHFR	1.85E+02	5.72E-01	2.80E-01	4.12E-02	4.41E-01

FAM86C1	4.64E+01	-6.71E-01	3.29E-01	4.12E-02	4.41E-01
GOLGA8R	4.54E+01	-6.27E-01	3.07E-01	4.12E-02	4.41E-01
HMOX1	1.82E+02	-8.21E-01	4.02E-01	4.12E-02	4.41E-01
MRPS9	1.87E+02	-5.27E-01	2.58E-01	4.10E-02	4.41E-01
NADK2	5.23E+02	5.40E-01	2.64E-01	4.12E-02	4.41E-01
PRADC1	1.23E+02	-7.39E-01	3.62E-01	4.11E-02	4.41E-01
PSMD7	4.67E+02	-4.07E-01	1.99E-01	4.10E-02	4.41E-01
RGL1	8.68E+02	-6.46E-01	3.17E-01	4.12E-02	4.41E-01
TIPARP	1.53E+03	9.69E-01	4.75E-01	4.11E-02	4.41E-01
WDR18	1.72E+02	-6.31E-01	3.09E-01	4.11E-02	4.41E-01
AGAP1	8.46E+02	6.70E-01	3.28E-01	4.14E-02	4.42E-01
DGKB	2.93E+01	9.65E-01	4.73E-01	4.14E-02	4.42E-01
RAPGEF1	1.14E+03	6.45E-01	3.16E-01	4.14E-02	4.42E-01
GPM6A	4.41E+01	-1.11E+00	5.42E-01	4.14E-02	4.42E-01
LINC00282	2.02E+01	1.11E+00	5.43E-01	4.15E-02	4.42E-01
PRELID2	6.68E+01	9.02E-01	4.42E-01	4.15E-02	4.43E-01
HRASLS5	1.09E+01	1.10E+00	5.42E-01	4.16E-02	4.43E-01
RSAD2	6.34E+01	7.28E-01	3.57E-01	4.16E-02	4.43E-01
IQCC	2.59E+01	8.41E-01	4.13E-01	4.19E-02	4.45E-01
PRRT3	8.77E+01	-6.88E-01	3.38E-01	4.19E-02	4.45E-01
TCF25	9.38E+02	-4.01E-01	1.97E-01	4.20E-02	4.45E-01
GPN3	1.35E+02	-4.40E-01	2.16E-01	4.20E-02	4.46E-01
TLR4	7.84E+02	5.72E-01	2.82E-01	4.20E-02	4.46E-01
HIVEP3	1.41E+02	-6.40E-01	3.15E-01	4.21E-02	4.46E-01
ZNF850	1.20E+02	8.32E-01	4.10E-01	4.21E-02	4.46E-01
SALL2	4.75E+02	6.89E-01	3.39E-01	4.22E-02	4.46E-01
RPL23AP32	6.11E+01	6.93E-01	3.41E-01	4.22E-02	4.46E-01
RPP25L	9.83E+01	-6.65E-01	3.28E-01	4.22E-02	4.46E-01
PHLDA1	9.70E+02	1.07E+00	5.27E-01	4.23E-02	4.46E-01
FAM177A1	3.26E+02	7.07E-01	3.49E-01	4.24E-02	4.47E-01
VTI1B	4.41E+02	6.12E-01	3.02E-01	4.24E-02	4.47E-01
SFT2D2	2.56E+02	4.35E-01	2.14E-01	4.25E-02	4.48E-01
CBFA2T2	1.31E+03	4.55E-01	2.24E-01	4.26E-02	4.48E-01
NR2C1	7.11E+02	5.57E-01	2.75E-01	4.25E-02	4.48E-01
ZNF704	2.22E+03	-7.71E-01	3.80E-01	4.26E-02	4.48E-01
TXNRD3	1.82E+02	-4.77E-01	2.35E-01	4.27E-02	4.49E-01
GSAP	4.93E+02	-6.68E-01	3.30E-01	4.28E-02	4.50E-01
KCNC4	2.41E+02	9.77E-01	4.83E-01	4.28E-02	4.50E-01
BMP8A	1.05E+01	1.10E+00	5.44E-01	4.30E-02	4.50E-01
CREB3L1	1.80E+02	1.07E+00	5.30E-01	4.30E-02	4.50E-01
HSD11B1L	3.62E+01	-9.15E-01	4.52E-01	4.30E-02	4.50E-01
LINC00908	7.44E+00	-1.06E+00	5.25E-01	4.30E-02	4.50E-01
PLAGL1	5.16E+02	9.03E-01	4.46E-01	4.30E-02	4.50E-01
SYS1	5.71E+02	5.41E-01	2.67E-01	4.30E-02	4.50E-01
TMCO3	1.09E+03	5.23E-01	2.58E-01	4.29E-02	4.50E-01
NAMPT	2.35E+03	-7.34E-01	3.63E-01	4.31E-02	4.50E-01
OAS3	5.21E+02	6.59E-01	3.26E-01	4.31E-02	4.50E-01
GJC1	3.17E+02	8.63E-01	4.27E-01	4.32E-02	4.50E-01
AURKA	4.68E+01	7.70E-01	3.81E-01	4.33E-02	4.51E-01
PLA2G16	3.29E+02	6.45E-01	3.19E-01	4.32E-02	4.51E-01
ADRA1A	2.74E+01	-1.07E+00	5.29E-01	4.35E-02	4.51E-01
ALPK1	3.38E+02	-4.97E-01	2.46E-01	4.36E-02	4.51E-01
ARF6	7.33E+02	5.39E-01	2.67E-01	4.37E-02	4.51E-01

ARL5A	1.55E+03	-5.30E-01	2.62E-01	4.34E-02	4.51E-01
BACE1	1.15E+03	-5.44E-01	2.70E-01	4.38E-02	4.51E-01
CNPY3	5.74E+02	-4.42E-01	2.19E-01	4.37E-02	4.51E-01
CTSO	7.37E+02	-6.62E-01	3.28E-01	4.36E-02	4.51E-01
HIBADH	3.58E+02	-6.13E-01	3.04E-01	4.35E-02	4.51E-01
MED12L	1.00E+02	8.59E-01	4.26E-01	4.37E-02	4.51E-01
MEIS3P1	2.92E+02	-5.63E-01	2.79E-01	4.37E-02	4.51E-01
MGA	1.60E+03	4.28E-01	2.12E-01	4.33E-02	4.51E-01
PLBD2	4.03E+02	8.44E-01	4.18E-01	4.33E-02	4.51E-01
POSTN	6.29E+02	1.10E+00	5.45E-01	4.38E-02	4.51E-01
RASGRF2	1.06E+03	8.78E-01	4.35E-01	4.35E-02	4.51E-01
SETDB2	2.94E+02	4.65E-01	2.30E-01	4.35E-02	4.51E-01
TDRD3	4.84E+02	3.81E-01	1.89E-01	4.36E-02	4.51E-01
ZFHX4	1.10E+03	-7.15E-01	3.54E-01	4.36E-02	4.51E-01
KCNJ2-AS1	2.82E+01	-9.48E-01	4.70E-01	4.39E-02	4.51E-01
MDGA2	1.29E+02	-1.09E+00	5.41E-01	4.39E-02	4.51E-01
SHROOM2	1.29E+02	9.97E-01	4.95E-01	4.39E-02	4.52E-01
TNFRSF10D	1.09E+02	1.02E+00	5.09E-01	4.40E-02	4.53E-01
URB1	2.42E+03	6.10E-01	3.03E-01	4.41E-02	4.53E-01
UNG	3.79E+02	-3.54E-01	1.76E-01	4.41E-02	4.53E-01
NXPH4	5.18E+02	8.22E-01	4.08E-01	4.42E-02	4.53E-01
RSPH4A	1.36E+01	-1.03E+00	5.11E-01	4.42E-02	4.53E-01
CADPS2	1.30E+03	-8.79E-01	4.37E-01	4.44E-02	4.53E-01
GRHL1	2.57E+02	9.71E-01	4.83E-01	4.44E-02	4.53E-01
SERTAD4	7.28E+01	-1.03E+00	5.12E-01	4.43E-02	4.53E-01
SPIRE2	8.40E+01	-8.18E-01	4.07E-01	4.43E-02	4.53E-01
ZFP14	2.81E+02	5.33E-01	2.65E-01	4.43E-02	4.53E-01
BAX	2.66E+02	4.76E-01	2.37E-01	4.45E-02	4.54E-01
TTLL5	4.04E+02	5.66E-01	2.82E-01	4.45E-02	4.54E-01
FAM155A	5.84E+01	1.05E+00	5.22E-01	4.46E-02	4.55E-01
PVR	3.80E+02	7.20E-01	3.59E-01	4.46E-02	4.55E-01
FAM229B	1.72E+02	-7.78E-01	3.88E-01	4.47E-02	4.55E-01
ADAMTSL4-A	6.15E+01	6.28E-01	3.13E-01	4.50E-02	4.57E-01
PNN	8.95E+02	4.99E-01	2.49E-01	4.50E-02	4.57E-01
SLC8A3	5.74E+00	1.08E+00	5.41E-01	4.50E-02	4.57E-01
C20orf194	9.86E+02	7.00E-01	3.49E-01	4.51E-02	4.57E-01
IFT140	5.03E+02	-6.56E-01	3.27E-01	4.51E-02	4.57E-01
MAP2	2.70E+02	1.02E+00	5.11E-01	4.51E-02	4.57E-01
YAP1	3.24E+03	4.67E-01	2.33E-01	4.50E-02	4.57E-01
FAM217B	3.80E+02	4.93E-01	2.46E-01	4.52E-02	4.58E-01
ZNF792	1.35E+02	7.74E-01	3.86E-01	4.52E-02	4.58E-01
LRRC16B	1.81E+01	9.48E-01	4.73E-01	4.52E-02	4.58E-01
GPR132	4.87E+01	-9.35E-01	4.67E-01	4.53E-02	4.58E-01
CRB2	1.43E+02	9.52E-01	4.76E-01	4.55E-02	4.59E-01
DDX56	5.66E+02	-4.69E-01	2.35E-01	4.57E-02	4.59E-01
HBB	1.13E+03	1.07E+00	5.33E-01	4.55E-02	4.59E-01
LINC00880	3.30E+00	1.01E+00	5.07E-01	4.57E-02	4.59E-01
NTHL1	7.31E+01	-6.72E-01	3.36E-01	4.56E-02	4.59E-01
PPP2R3C	1.03E+02	6.58E-01	3.29E-01	4.56E-02	4.59E-01
RNF180	1.48E+02	1.01E+00	5.07E-01	4.55E-02	4.59E-01
SARDH	8.47E+01	-1.02E+00	5.08E-01	4.56E-02	4.59E-01
SEMA3B	2.14E+03	-7.22E-01	3.62E-01	4.57E-02	4.59E-01
SRC	4.79E+02	6.21E-01	3.11E-01	4.55E-02	4.59E-01

UBXN2B	7.60E+02	-5.26E-01	2.63E-01	4.57E-02	4.59E-01
WBP4	1.84E+02	5.28E-01	2.64E-01	4.56E-02	4.59E-01
RBL1	1.30E+02	5.67E-01	2.84E-01	4.58E-02	4.59E-01
MKI67	5.83E+02	7.30E-01	3.66E-01	4.59E-02	4.59E-01
NCOA6	1.24E+03	5.22E-01	2.61E-01	4.58E-02	4.59E-01
EBAG9	3.51E+02	-6.05E-01	3.03E-01	4.60E-02	4.60E-01
RNF138P1	3.81E+02	8.08E-01	4.05E-01	4.60E-02	4.60E-01
GHR	4.73E+02	6.90E-01	3.46E-01	4.63E-02	4.63E-01
ADAM9	2.29E+03	-7.22E-01	3.62E-01	4.63E-02	4.63E-01
C5orf51	5.91E+02	3.46E-01	1.74E-01	4.63E-02	4.63E-01
CWF19L1	2.03E+02	5.19E-01	2.61E-01	4.66E-02	4.63E-01
EDIL3	1.15E+02	1.02E+00	5.15E-01	4.66E-02	4.63E-01
EIF3G	7.99E+02	-4.85E-01	2.44E-01	4.66E-02	4.63E-01
GBP1P1	2.79E+00	-1.08E+00	5.45E-01	4.66E-02	4.63E-01
GNAS	9.37E+03	3.86E-01	1.94E-01	4.66E-02	4.63E-01
KDM5A	2.10E+03	5.70E-01	2.86E-01	4.64E-02	4.63E-01
LINC00158	3.85E+00	1.08E+00	5.45E-01	4.66E-02	4.63E-01
LOC1019274	3.63E+01	5.95E-01	2.99E-01	4.66E-02	4.63E-01
LOC286189	1.37E+03	-1.08E+00	5.41E-01	4.65E-02	4.63E-01
NIPAL2	3.57E+02	-6.99E-01	3.51E-01	4.65E-02	4.63E-01
PCDH1	1.95E+02	9.72E-01	4.88E-01	4.66E-02	4.63E-01
CRELD2	1.49E+02	-6.41E-01	3.23E-01	4.67E-02	4.63E-01
RHBDF2	4.25E+02	-8.16E-01	4.10E-01	4.68E-02	4.63E-01
DAGLA	9.93E+01	6.40E-01	3.22E-01	4.69E-02	4.64E-01
DNAJC9	1.83E+02	4.47E-01	2.25E-01	4.69E-02	4.64E-01
ST8SIA4	2.10E+02	-7.37E-01	3.71E-01	4.69E-02	4.64E-01
TCEAL8	5.08E+02	3.22E-01	1.62E-01	4.69E-02	4.64E-01
LRRC8E	4.86E+01	-7.57E-01	3.81E-01	4.70E-02	4.64E-01
DCAF4	9.65E+01	7.29E-01	3.67E-01	4.71E-02	4.65E-01
EVA1C	2.31E+02	8.46E-01	4.26E-01	4.71E-02	4.65E-01
SETBP1	2.48E+03	6.21E-01	3.13E-01	4.71E-02	4.65E-01
SEC14L1P1	9.48E+01	5.40E-01	2.72E-01	4.72E-02	4.65E-01
LOC642852	4.25E+02	6.92E-01	3.49E-01	4.72E-02	4.65E-01
IL6	6.82E+00	-1.08E+00	5.45E-01	4.73E-02	4.65E-01
SLC38A1	4.11E+03	-1.03E+00	5.17E-01	4.74E-02	4.65E-01
SPPL2A	8.13E+02	-3.46E-01	1.74E-01	4.74E-02	4.65E-01
ZC4H2	3.85E+02	4.88E-01	2.46E-01	4.74E-02	4.65E-01
SIK1	1.75E+03	-9.50E-01	4.79E-01	4.74E-02	4.65E-01
EEPD1	1.51E+02	8.83E-01	4.46E-01	4.75E-02	4.65E-01
TANGO6	2.31E+02	-4.02E-01	2.03E-01	4.75E-02	4.65E-01
CLEC17A	5.12E+00	-1.07E+00	5.43E-01	4.76E-02	4.66E-01
CTR9	9.08E+02	-3.30E-01	1.66E-01	4.76E-02	4.66E-01
FAM21EP	4.37E+00	1.07E+00	5.43E-01	4.76E-02	4.66E-01
TMEM158	3.00E+01	1.07E+00	5.41E-01	4.76E-02	4.66E-01
PPP1R14A	1.03E+01	-1.07E+00	5.39E-01	4.78E-02	4.67E-01
CYP39A1	8.34E+01	-1.01E+00	5.09E-01	4.79E-02	4.68E-01
ZNF581	1.46E+02	4.47E-01	2.26E-01	4.80E-02	4.69E-01
SPATA2L	5.34E+01	-8.01E-01	4.05E-01	4.81E-02	4.69E-01
LCP2	2.27E+02	-6.63E-01	3.36E-01	4.82E-02	4.69E-01
SLX4IP	7.55E+01	4.91E-01	2.48E-01	4.82E-02	4.70E-01
FAM160B2	5.77E+02	-4.79E-01	2.43E-01	4.83E-02	4.70E-01
BTK	8.21E+01	-8.41E-01	4.26E-01	4.87E-02	4.71E-01
CDH24	3.24E+02	6.49E-01	3.29E-01	4.84E-02	4.71E-01

DOK1	1.81E+02	-5.93E-01	3.00E-01	4.85E-02	4.71E-01
GDA	4.86E+00	1.05E+00	5.31E-01	4.85E-02	4.71E-01
LOC284412	1.25E+01	1.03E+00	5.23E-01	4.86E-02	4.71E-01
MMP21	6.30E+00	-1.07E+00	5.44E-01	4.86E-02	4.71E-01
N4BP2L2-IT2	6.10E+02	5.09E-01	2.58E-01	4.85E-02	4.71E-01
NCKAP1L	4.67E+02	-7.67E-01	3.89E-01	4.86E-02	4.71E-01
NID2	4.08E+03	9.13E-01	4.63E-01	4.84E-02	4.71E-01
PRPH2	1.05E+02	-7.72E-01	3.92E-01	4.86E-02	4.71E-01
RASL10B	1.33E+02	1.06E+00	5.37E-01	4.85E-02	4.71E-01
ANKRD36BP2	1.00E+01	1.07E+00	5.44E-01	4.88E-02	4.71E-01
CALB1	3.53E+02	-1.03E+00	5.22E-01	4.88E-02	4.71E-01
CBS	3.06E+02	7.92E-01	4.02E-01	4.89E-02	4.71E-01
CHAF1B	5.00E+01	8.25E-01	4.19E-01	4.89E-02	4.71E-01
FOXP1	5.60E+03	-6.02E-01	3.06E-01	4.89E-02	4.71E-01
GIPR	2.87E+00	-1.04E+00	5.29E-01	4.89E-02	4.71E-01
KARS	8.98E+02	-4.85E-01	2.46E-01	4.88E-02	4.71E-01
SEMA5B	3.71E+01	9.90E-01	5.03E-01	4.89E-02	4.71E-01
TSC22D2	8.77E+02	-4.22E-01	2.14E-01	4.89E-02	4.71E-01
LEPREL2	5.26E+02	-6.89E-01	3.50E-01	4.90E-02	4.71E-01
EFNA2	7.66E+00	-1.04E+00	5.27E-01	4.91E-02	4.72E-01
DLEC1	6.98E+01	-9.48E-01	4.82E-01	4.92E-02	4.72E-01
MOCS3	1.25E+02	5.42E-01	2.75E-01	4.92E-02	4.72E-01
TENM1	3.03E+01	1.02E+00	5.20E-01	4.92E-02	4.72E-01
ZWINT	7.19E+01	7.43E-01	3.78E-01	4.92E-02	4.72E-01
ADORA3	2.82E+02	-9.19E-01	4.68E-01	4.93E-02	4.72E-01
CDS1	1.04E+02	8.53E-01	4.34E-01	4.93E-02	4.72E-01
OTUD5	6.62E+02	2.49E-01	1.27E-01	4.93E-02	4.72E-01
WBP5	6.11E+02	6.30E-01	3.21E-01	4.94E-02	4.72E-01
ST6GALNAC4	3.39E+02	-7.39E-01	3.76E-01	4.94E-02	4.72E-01
ANKRD23	8.39E+01	-5.21E-01	2.65E-01	4.96E-02	4.73E-01
CEP290	9.03E+02	4.44E-01	2.26E-01	4.96E-02	4.73E-01
FOXN3	2.67E+03	8.21E-01	4.18E-01	4.96E-02	4.73E-01
ZNF334	4.20E+02	5.22E-01	2.66E-01	4.96E-02	4.73E-01
PTGES	1.84E+02	-9.80E-01	4.99E-01	4.97E-02	4.74E-01
A1BG	7.81E+00	-1.05E+00	5.35E-01	4.99E-02	4.74E-01
PARP11	1.43E+02	7.20E-01	3.67E-01	5.00E-02	4.74E-01
QSOX2	2.37E+02	-5.64E-01	2.88E-01	4.99E-02	4.74E-01
SPRYD7	1.22E+02	6.35E-01	3.24E-01	4.99E-02	4.74E-01
TRDN	9.02E+00	-9.99E-01	5.09E-01	5.00E-02	4.74E-01
